# Supplementary material for: Tunable enantioselective electrocatalytic functionalization of unactivated alkenes
Source: Nat Commun. 2026 Jan 2;17:1372. doi: 10.1038/s41467-025-68123-3 (PMC12877130; doi:10.1038/s41467-025-68123-3)
Supplement: Supplementary file 1 — Supplementary Information [file 41467_2025_68123_MOESM1_ESM.pdf]

Supplementary Information

Tunable Enantioselective Electrocatalytic Functionalization of Unactivated Alkenes

Tian Xie[1], Minghao Liu[1], Jiayin Zhang[1], Lingzi Peng[1], Chang Guo[1,2]*

[1]Hefei National Research Center for Physical Sciences at the Microscale and Department of Chemistry, University of Science and Technology of China, Hefei, 230026, China.
[2]State Key Laboratory of Coordination Chemistry, School of Chemistry and Chemical Engineering, Nanjing University, Nanjing, 210093, China

*Corresponding author. Email: guochang@ustc.edu.cn

CONTENTS:

1. General information S1

2. Synthesis and characterization of substrate S2

3. Photographic guide for electrochemical reactions S9

4. Optimization of the reaction conditions..S10

5. Synthesis and characterization of products..S15

6. Synthetic applications..S37

7. Cyclic voltammetry studies S45

8. Mechanistic studies S48

9. X-ray crystallography data S51

10. Computational details...S53

11. NMR spectra S65

12. HPLC traces S198

13. References...S272

1. General information

Unless otherwise noted, all reagents were purchased from commercial suppliers and used without further purification. Cyclic voltammograms were recorded on a CHI 760E potentiostat. ^{1}H NMR and ^{13}C NMR spectra were recorded at 25 ºC on Bruker Advance 400 M NMR spectrometers, Bruker Advance 500 M NMR spectrometers, and JEOL 600 MHz spectrometers. High-resolution mass spectral analysis (HRMS) was performed on a Waters XEVO G2 Q-TOF. Optical rotations were determined at 589 nm (sodium D line) by using a Perkin-Elmer-343 polarimeter (1 dm path length cell). The measurement of enantiomeric excesses was performed on a Waters Alliance (2998, Photodiode Array Detector). Chiralpak IC, IE, and IG columns were purchased from Daicel Chemical Industries, Ltd.

2. Synthesis and characterization of substrate

Synthesis of benzoxazolyl acetates

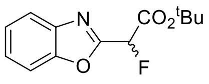

1a

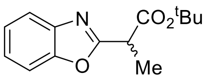

1b

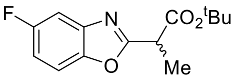

1c

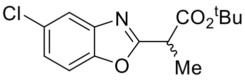

1d

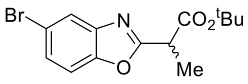

1e

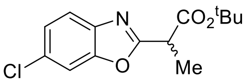

1f

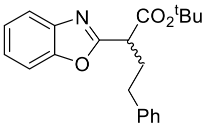

1g

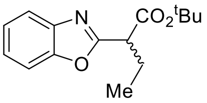

1h

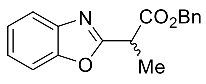

1i

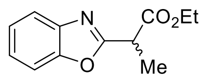

1j

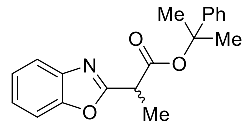

1k

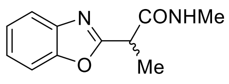

1l

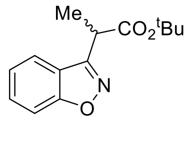

1m

1a-1m were prepared according to literature procedures[1,2].

Method A:

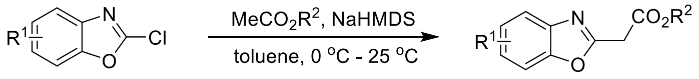

S1

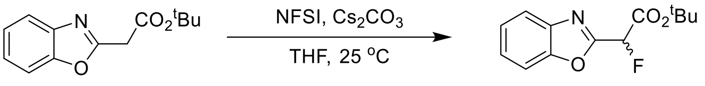

S1 **1a**

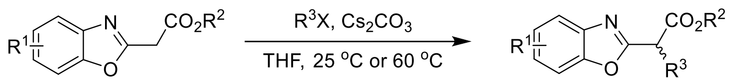

S1 **1b, 1d- 1i, 1k**

tert-Butyl acetate (40 mmol) was added dropwise to a solution of NaHMDS (2 M in THF, 40 mmol) in dry toluene (40 mL) at 0 °C. After stirring at 0 °C for 30 minutes, 2-chlorobenzoxazole (20 mmol) was added. The mixture was gradually heated to 25 °C and stirred continuously for 3 hours. After that, it was quenched by the addition of saturated aqueous NH_4Cl solution. The organic layer was separated, and the aqueous layer was extracted with EtOAc. The combined organic layers were washed with brine, dried over $MgSO_4$, filtered, and concentrated in vacuo. The crude product was purified by flash chromatography to afford the desired product **S1**.

N-fluorobenzenesulfonimide (15 mmol) was added to a solution of cesium carbonate (15 mmol) and

S1 (10 mmol) in THF (20 mL). The mixture was stirred at 25 °C until the reaction was complete (monitored by TLC). The solid was separated by filtration. The filtrate was subsequently concentrated under reduced pressure. The resulting crude mixture was subsequently subjected to purification via flash column chromatography, affording the desired product **1a**.

To a solution of cesium carbonate (15 mmol) and **S1** (10 mmol) in THF (20 mL), alkyl bromide or alkyl iodide (15 mmol) was added dropwise. The mixture was stirred at 25 °C or 60 °C until the reaction was complete (monitored by TLC). The solid was separated by filtration. The filtrate was subsequently concentrated under reduced pressure, resulting in a crude mixture. This crude mixture was then purified via flash column chromatography, and the desired products **1b**, **1d**-**1i**, and **1k** were obtained.

Method B:

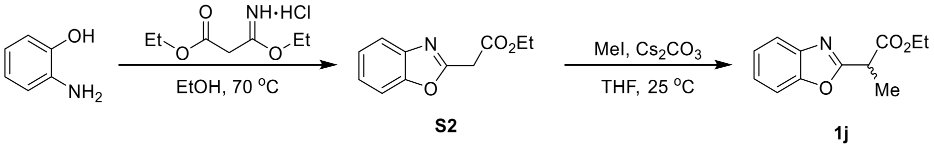

A mixture of 1-amino-2-hydroxybenzene (50 mmol) and ethyl 3-ethoxy-3-iminopropanoate hydrochloride (50 mmol) in anhydrous ethanol (100 mL) was stirred for 4 h at 70 °C. The solvent was removed under reduced pressure. The residue was dissolved in DCM, and filtered. The filtrate was concentrated under reduced pressure. The crude product was purified by flash chromatography to afford the desired product **S2**. Iodomethane (15 mmol) was added dropwise to a solution of cesium carbonate (15 mmol) and **S2** (10 mmol) in THF (20 mL). The mixture was stirred at 25 °C until the reaction was complete (monitored by TLC). The solid was filtered out. The filtrate was subsequently concentrated under reduced pressure. After concentration, the resulting crude mixture was purified via flash column chromatography, and the desired product **1j** was successfully obtained.

Method C:

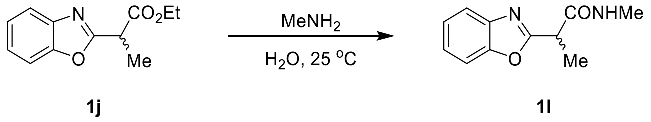

A mixture of **1j** (10 mmol) and methylamine (50 mmol, 40 wt.% in H_2O) in ethanol (5 mL) was stirred for 6 h at 25 °C until the reaction was complete (monitored by TLC). The aqueous layer was extracted with DCM (20 mL × 3). The combined organic layers were dried over anhydrous $MgSO_4$, filtered, and concentrated in vacuo. The crude mixture was purified by flash column chromatography to give the desired product **1l**.

Method D:

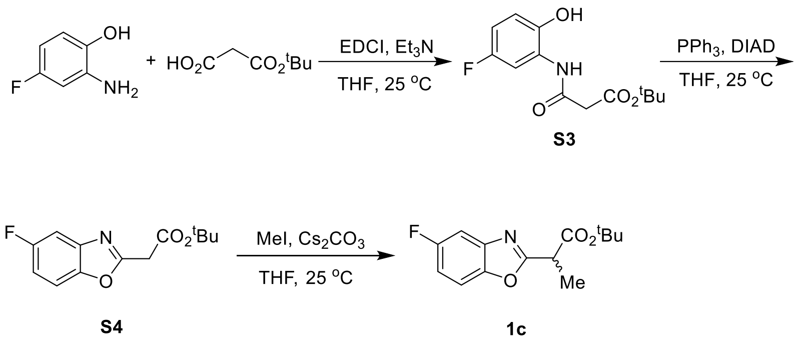

A mixture of 2-amino-4-fluorophenol (20 mmol), 3-(*tert*-butoxy)-3-oxopropanoic acid (24 mmol), EDCI (30 mmol), and Et_3N (30 mmol) in THF (50 mL) was stirred for 12 h at 25 °C until the reaction was complete (monitored by TLC). The solvent was removed under reduced pressure. The crude product was purified by flash chromatography to afford product **S3**. DIAD (22 mmol) was added dropwise to a solution of **S3** (10 mmol) and PPh_3 (22 mmol) in THF (60 mL). The mixture was stirred at 25 °C until the reaction was complete (monitored by TLC). The solid was filtered off, and the filtrate was concentrated under reduced pressure. The crude mixture was purified by flash column chromatography to obtain the desired product **S4**.

Iodomethane (7.5 mmol) was added dropwise to a solution of cesium carbonate (7.5 mmol) and **S4** (5 mmol) in THF (20 mL). The mixture was stirred at 25 °C until the reaction was complete (monitored by TLC). The solid was filtered off, and the filtrate was concentrated under reduced pressure. The crude mixture was purified by flash column chromatography to give desired product **1c**.

Method E:

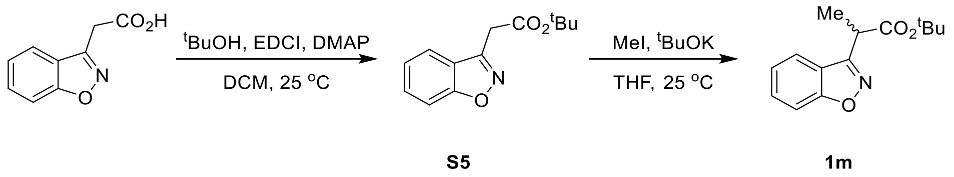

A mixture of 1,2-benzisoxazole-3-acetic acid (40 mmol), EDCI (48 mmol), DMAP (8 mmol), and *tert*-butanol (200 mmol) in DCM (100 mL) was stirred for 12 h at 25 °C until the reaction was complete (monitored by TLC). The solvent was removed under reduced pressure. The crude product was purified by flash chromatography to afford product **S5**.

Iodomethane (24 mmol) was added dropwise to a solution of potassium *tert*-butoxide (24 mmol) and **S5** (20 mmol) in THF (60 mL). The mixture was stirred at 25 °C until the reaction was complete (monitored by TLC). The solid was filtered off, and the filtrate was concentrated under reduced pressure. The crude mixture was purified by flash column chromatography to obtain the desired product **1m**.

Synthesis of alkenes

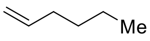

2a

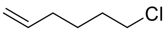

2b

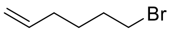

2c

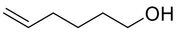

2d

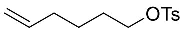

2e

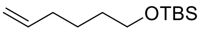

2f

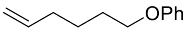

2g

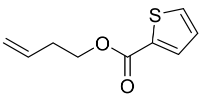

2h

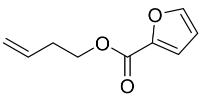

2i

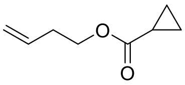

2j

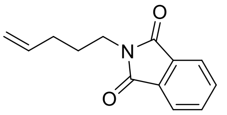

2k

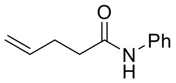

2l

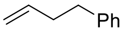

2m

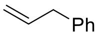

2n

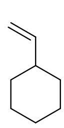
2o

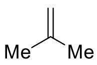

2p

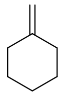
2q

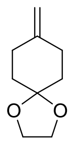

2r

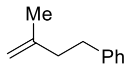

2s

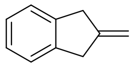
2t

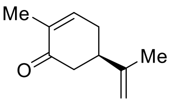

2u

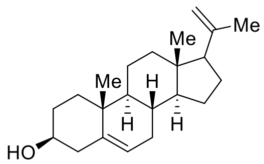

2v

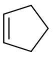
2w

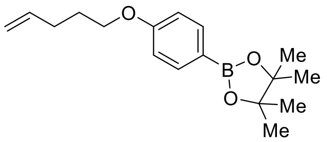

2x

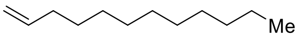

2y

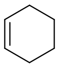
2z

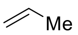

2aa

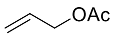

2ab

2a-2d, **2m-2q**, **2u**, **2w**, **2y**, **2z**, and **2aa-2ab** were purchased from commercial suppliers and used without further purification.

2e-2l, **2r-2t**, **2v**, and **2x** were prepared according to literature procedures[3-6].

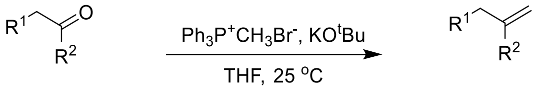

2r-2t and **2v**

Under an Ar atmosphere, a mixture of methyl triphenylphosphonium bromide (36 mmol), KO^{t}Bu (36 mmol), and dry THF (40 mL) was stirred for 2 h at 25 °C. After that, a solution of aldehyde or ketone (30 mmol) in THF (10 mL) was added dropwise and stirred for 16 h. A solution of saturated NH_4Cl (20 mL) was added to quench the reaction, and the aqueous layer was extracted with EtOAc (30 mL × 3). The combined organic layers were dried over anhydrous $MgSO_4$, filtered, and concentrated in vacuo. The residue was chromatographed on silica gel to obtain the alkenes **2r-2t**, and **2v**.

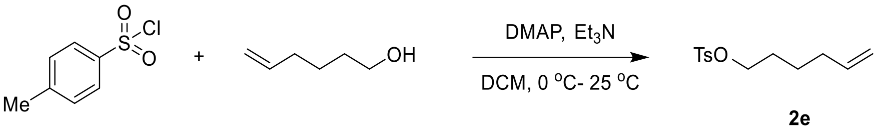

2e

To a solution of hex-5-en-1-ol (20 mmol), DMAP (1 mmol) and Et_3N (26 mmol) in DCM (40 mL) was added TsCl (22 mmol) at 0 °C, and the mixture was stirred for 12 h at 25 °C (monitored by TLC). After completion, the reaction mixture was diluted with DCM, washed with brine, dried over $MgSO_4$, filtered, and concentrated in vacuo. The residue was chromatographed on silica gel to obtain the alkene **2e**.

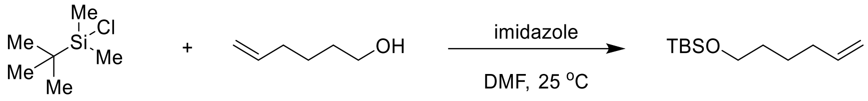

2f

TBSCl (22 mmol) was added to a solution of hex-5-en-1-ol (20 mmol) and imidazole (22 mmol) in DMF at 25 °C, and the mixture was stirred for 12 h (monitored by TLC). The reaction was quenched with water and extracted with diethyl ether. The combined organic layers were washed with saturated NaCl 3 times, dried with $MgSO_4$, filtered, and concentrated in vacuo. The residue was chromatographed on silica gel to obtain alkene **2f**.

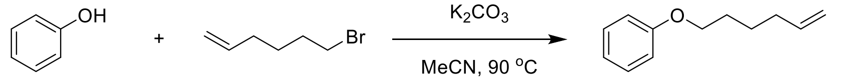

2g

K_2CO_3 (36 mmol) was added to a solution of phenol (30 mmol) and 6-bromohex-1-ene (36 mmol) in MeCN (50 mL) at 25 °C, and the mixture was stirred for 6 h at 90 °C in an oil bath (monitored by TLC). After being cooled to 25 °C, the solvent was removed under reduced pressure. The residue was chromatographed on silica gel to obtain alkene **2g**.

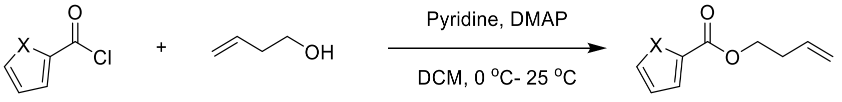

X = S, **2h**
X = O, **2i**

3-Buten-1-ol (20 mmol), pyridine (40 mmol), DMAP (2 mmol), and DCM (60 mL) was added to a round bottom flask, and then, acyl chloride (24 mmol) was added dropwise at 0 °C. The mixture was stirred at 25 °C for 12 h. The combined organic layer was washed with brine, dried over $MgSO_4$, filtered, and concentrated in vacuo. The residue was chromatographed on silica gel to obtaom alkenes **2h** and **2i**.

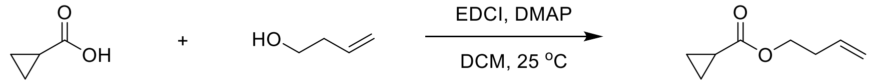

2j

To a solution of cyclopropanecarboxylic acid (20 mmol), EDCI (26 mmol) and DMAP (2 mmol) in DCM (60 ml) was added but-3-en-1-ol (24 mmol) at 25 °C, and the mixture was stirred for 12 h (monitored by TLC). The solvent was removed under reduced pressure. The residue was chromatographed on silica gel to obtain alkene **2j**.

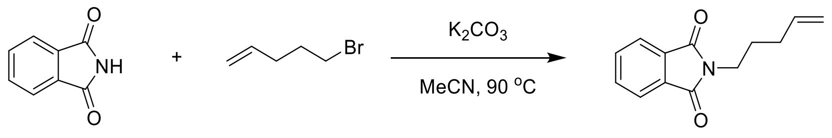

2k

K_2CO_3 (36 mmol) was added to a solution of phthalimide (30 mmol) and 5-bromopent-1-ene (36 mmol) in MeCN (50 mL) at 25 °C, and the mixture was stirred for 6 h at 90 °C in an oil bath (monitored by TLC). After being cooled to 25 °C, the solvent was removed under reduced pressure. The residue was chromatographed on silica gel to obtain alkene **2k**.

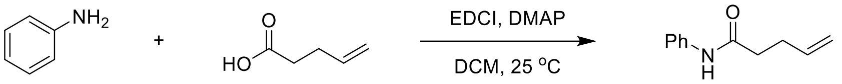

2l

Aniline (24 mmol) was added to a solution of 4-pentenoic acid (20 mmol), EDCI (26 mmol), and DMAP (2 mmol) in DCM (60 ml) at 25 °C, and the mixture was stirred for 12 h (monitored by TLC). The solvent was removed under reduced pressure. The residue was chromatographed on silica gel to obtain alkene **2l**.

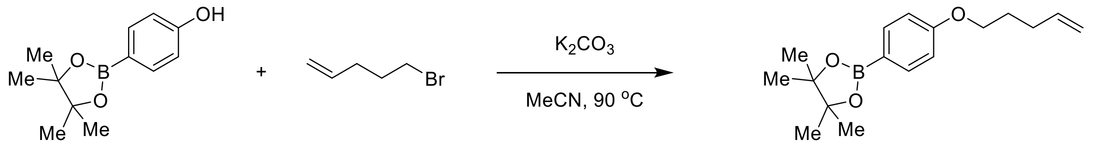

2x

K_2CO_3 (36 mmol) was added to a solution of 4-Hydroxyphenylboronic acid pinacol ester (30 mmol) and 5-bromopent-1-ene (36 mmol) in MeCN (50 mL) at 25 °C, and the mixture was stirred for 6 h at 90 °C in an oil bath (monitored by TLC). After being cooled to 25 °C, the solvent was removed under reduced pressure. The residue was chromatographed on silica gel to obtain alkene **2x**.

The data for **1a-1b**, **1g**, **1i-1l, 2e-2i, 2k-2n, 2r-2t, 2v,** and **2x** were in agreement with those previously reported.

***Tert*-butyl 2-(5-fluorobenzo[*d*]oxazol-2-yl)propanoate (1c)**

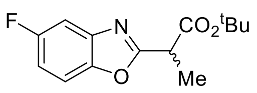

^{1}H NMR (600 MHz, CDCl$_3$) δ 7.45–7.36 (m, 2H), 7.04 (dd, *J* = 9.1, 2.7 Hz, 1H), 4.01 (q, *J* = 7.3 Hz, 1H), 1.68 (d, *J* = 7.4 Hz, 3H), 1.43 (s, 9H). **^{13}C NMR (151 MHz, CDCl$_3$)** δ 169.47, 166.49, 160.04 (d, *J* = 240.3 Hz), 147.35, 141.96 (d, *J* = 13.1 Hz), 112.73 (d, *J* = 26.3 Hz), 110.98 (d, *J* = 10.1 Hz), 106.63 (d, *J* = 25.6 Hz), 82.53, 41.83, 28.00, 15.11. **^{19}F NMR (565 MHz, CDCl$_3$)** δ -118.00. **ESI-MS: calculated [$C_{14}H_{16}FNO_3$ + Na]$^+$: 288.1006, found: 288.1003.**

***Tert*-butyl 2-(5-chlorobenzo[*d*]oxazol-2-yl)propanoate (1d)**

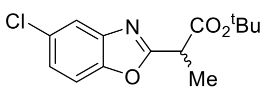

^{1}H NMR (600 MHz, CDCl$_3$) δ 7.69 (d, *J* = 2.1 Hz, 1H), 7.43 (d, *J* = 8.6 Hz, 1H), 7.30 (dd, *J* = 8.6, 2.1 Hz, 1H), 4.02 (q, *J* = 7.3 Hz, 1H), 1.68 (d, *J* = 7.3 Hz, 3H), 1.43 (s, 9H). **^{13}C NMR (151 MHz, CDCl$_3$)** δ 169.37, 166.11, 149.63, 142.34, 129.93, 125.41, 120.20, 111.43, 82.57, 41.82, 28.02, 15.11. **ESI-MS: calculated [$C_{14}H_{16}ClNO_3$ + Na]$^+$: 304.0711, found: 304.0710.**

***Tert*-butyl 2-(5-bromobenzo[*d*]oxazol-2-yl)propanoate (1e)**

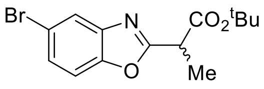

^{1}H NMR (600 MHz, CDCl$_3$) δ 7.84 (d, *J* = 1.9 Hz, 1H), 7.43 (dd, *J* = 8.6, 1.9 Hz, 1H), 7.38 (d, *J* = 8.6 Hz, 1H), 4.01 (q, *J* = 7.3 Hz, 1H), 1.68 (d, *J* = 7.3 Hz, 3H), 1.43 (s, 9H). **^{13}C NMR (151 MHz, CDCl$_3$)** δ 169.33, 165.92, 150.03, 142.81, 128.11, 123.19, 117.16, 111.93, 82.56, 41.78, 28.01, 15.10. **ESI-MS: calculated [$C_{14}H_{16}BrNO_3$ + Na]$^+$: 348.0206, found: 348.0205.**

***Tert*-butyl 2-(6-chlorobenzo[*d*]oxazol-2-yl)propanoate (1f)**

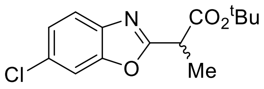

^{1}H NMR (600 MHz, CDCl$_3$) δ 7.61 (d, *J* = 8.5 Hz, 1H), 7.52 (d, *J* = 1.9 Hz, 1H), 7.30 (dd, *J* = 8.5, 2.0 Hz, 1H), 4.01 (q, *J* = 7.3 Hz, 1H), 1.68 (d, *J* = 7.3 Hz, 3H), 1.43 (s, 9H). **^{13}C NMR (151 MHz, CDCl$_3$)** δ 169.38, 165.38, 151.21, 139.98, 130.76, 125.13, 120.67, 111.37, 82.55, 41.76, 28.02, 15.11. **ESI-MS: calculated [$C_{14}H_{16}ClNO_3$ + Na]$^+$: 304.0711, found: 304.0713.**

***Tert*-butyl 2-(benzo[*d*]oxazol-2-yl)butanoate (1h)**

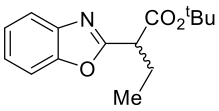

^{1}H NMR (600 MHz, CDCl$_3$) δ 7.75–7.68 (m, 1H), 7.54–7.48 (m, 1H), 7.35–7.29 (m, 2H), 3.84 (t, *J* = 7.6 Hz, 1H), 2.27–2.13 (m, 2H), 1.44 (s, 9H), 1.03 (t, *J* = 7.4 Hz, 3H). **^{13}C NMR (151 MHz, CDCl$_3$)** δ 169.03, 163.86, 151.03, 141.23, 124.99, 124.36, 120.16, 110.70, 82.32, 49.08, 28.07, 23.80, 12.08. **ESI-MS: calculated [$C_{15}H_{19}NO_3$ + Na]$^+$: 284.1257, found: 284.1261.**

***Tert*-butyl 2-(benzo[*d*]isoxazol-3-yl)propanoate (1m)**

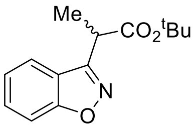

^{1}H NMR (600 MHz, CDCl$_3$) δ 7.73 (d, *J* = 8.0 Hz, 1H), 7.58–7.48 (m, 2H), 7.27 (t, *J* = 7.4 Hz, 1H), 4.15 (q, *J* = 7.3 Hz, 1H), 1.66 (d, *J* = 7.4 Hz, 3H), 1.37 (s, 9H). **^{13}C NMR (151 MHz, CDCl$_3$)** δ 170.82, 163.45, 157.56, 129.82, 123.35, 122.15, 120.49, 110.03, 81.96, 39.15, 27.91, 15.75. **ESI-MS: calculated [$C_{14}H_{17}NO_3$ + Na]$^+$: 270.1101, found: 270.1094.**

3. Photographic guide for electrochemical reactions

3.1. Overview of the materials used

From left to right: 1) Electrochemical cell. 2) Carbon felt (1.5 cm × 1.5 cm × 3 mm) anode and platinum plate (1.0 cm × 1.0 cm × 0.2 mm) cathode. (Purchased from Tianjin AIDAhengsheng Science-Technology Development Co., Ltd.).

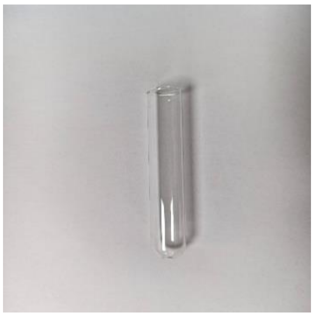

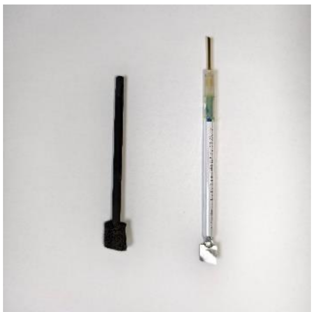

3.2. Assembling the cell

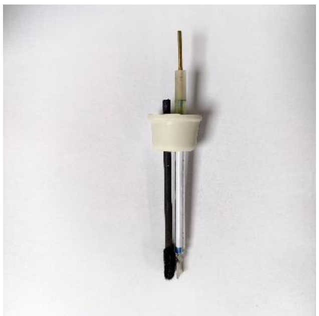

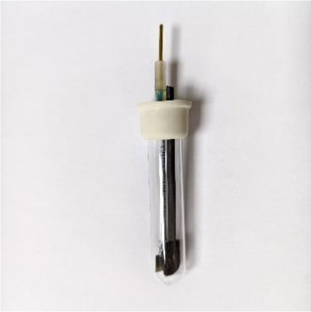

3.3. Electrolysis

The potentiostat was purchased from Shanghai Xin Rui Instrument Co., Ltd.

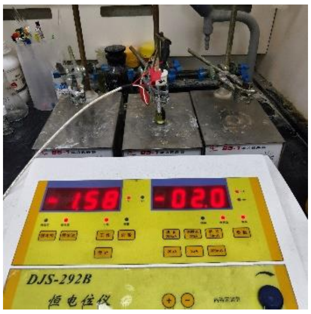

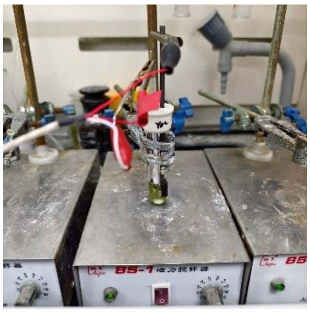

4. Optimization of the reaction conditions

Table S1. Survey of chiral ligands for dehydrogenative alkenylation.[a]

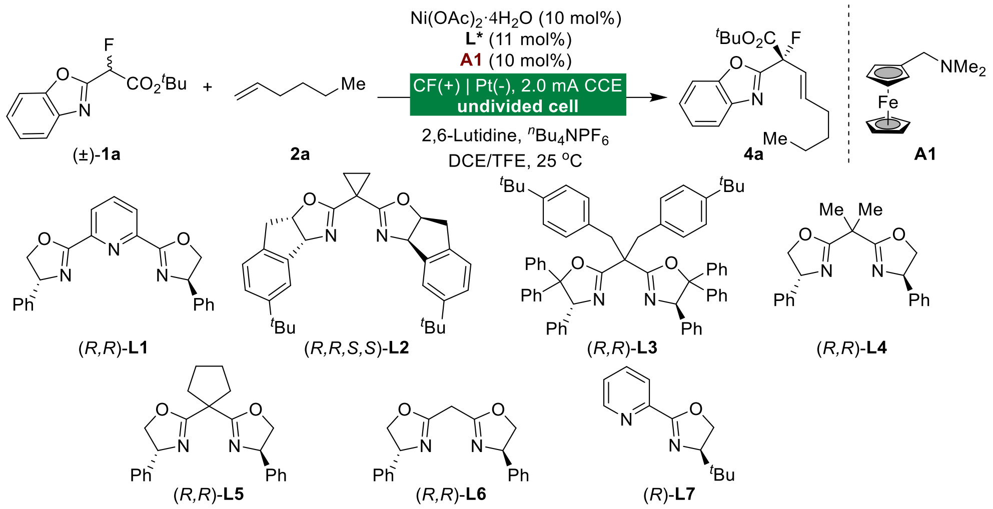

entry	L*	Results of **4a**
1	**L1**	11% yield, 60% e.e.
2	**L2**	27% yield, 40% e.e.
3	**L3**	37% yield, 67% e.e.
4	**L4**	58% yield, 88% e.e.
5	**L5**	72% yield, 92% e.e.
6	**L6**	40% yield, 88% e.e.
7	**L7**	20% yield, 42% e.e.

[a]Reactions were performed by using racemic benzoxazolyl acetate **1a** (0.1 mmol), alkene **2a** (0.5 mmol), $Ni(OAc)_2 \cdot 4H_2O$ (10 mol%), **L*** (11 mol%), **A1** (10 mol%), 2,6-Lutidine (0.1 mmol), $^nBu_4NPF_6$ (0.1 M), DCE (0.5 mL) and TFE (2.5 mL) at 25 °C under constant-current conditions (2.0 mA) in an undivided cell. The enantiomeric excess (e.e.) was analyzed via high-performance liquid chromatography (HPLC). TFE, 2,2,2-trifluoroethanol; CF, carbon felt.

Table S2. Survey of additives for dehydrogenative allylation.[a]

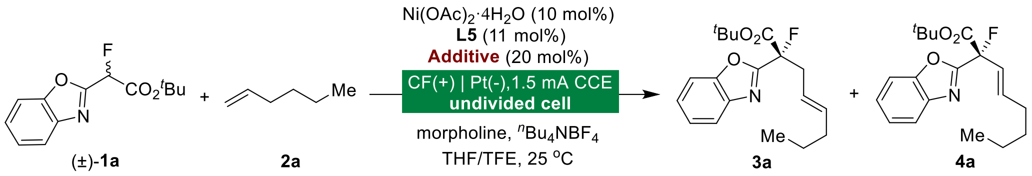

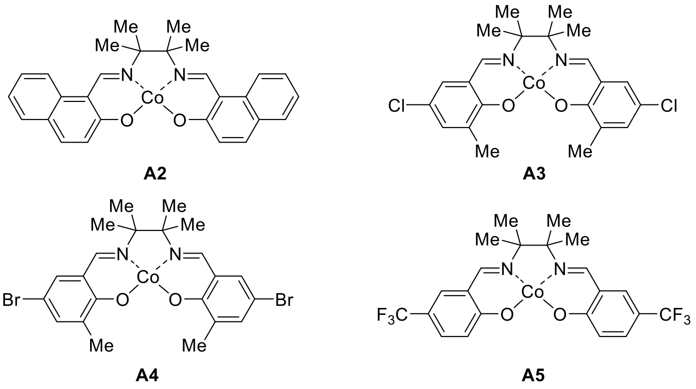

entry	**Additive**	Results of **3a**	Results of **4a**
1	**A2**	11% yield, 74% e.e.	11% yield, 76% e.e.
2	**A3**	32% yield, 85% e.e.	5% yield, 86% e.e.
3	**A4**	28% yield, 86% e.e.	7% yield, 88% e.e.
4	**A5**	74% yield, 92% e.e.	7% yield, 92% e.e.

[a]Reactions were performed by using racemic benzoxazolyl acetate **1a** (0.1 mmol), alkene **2a** (0.5 mmol), $Ni(OAc)_2·4H_2O$ (10 mol%), **L5** (11 mol%), additive (20 mol%), morpholine (0.1 mmol), $^nBu_4NBF_4$ (0.1 M), THF (0.5 mL) and TFE (2.5 mL) at 25 °C under constant-current conditions (2.0 mA) in an undivided cell. The enantiomeric excess (e.e.) was analyzed via high-performance liquid chromatography (HPLC). TFE, 2,2,2-trifluoroethanol; CF, carbon felt.

Table S3. Survey of the HAT reagents for hydroalkylation.[a]

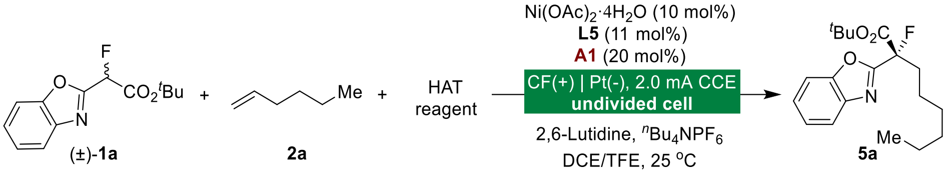

entry	HAT reagent	Results of **5a**
1	1,4-cyclohexadiene (BDE = 77 kcal/mol)	nd
2	$(MeO)_3SiH$ (BDE(calc) = 96.3 kcal/mol)	nd
3	Ph_2SiH_2 (BDE = 90.6 kcal/mol)	nd
4	Ph_3SiH (BDE = 88.7 kcal/mol)	nd
5	$(TMS)_3Si$-H (BDE = 79 kcal/mol)	56% yield, 92% e.e.

[a]Reactions were performed by using racemic benzoxazolyl acetate **1a** (0.1 mmol), alkene **2a** (0.5 mmol), $Ni(OAc)_2·4H_2O$ (10 mol%), **L5** (11 mol%), **A1** (10 mol%), HAT reagent (0.5 mmol), 2,6-Lutidine (0.1 mmol), $^nBu_4NPF_6$ (0.1 M), DCE (0.5 mL) and TFE (2.5 mL) at 25 °C under constant-current conditions (2.0 mA) in an undivided cell. The enantiomeric excess (e.e.) was analyzed via high-performance liquid chromatography (HPLC).

Table S4. Survey of solvents for hydroalkylation.[a]

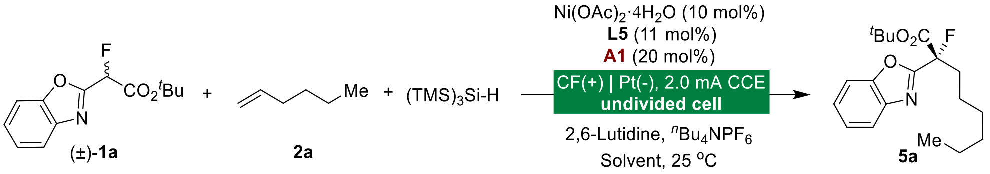

entry	Solvent	Results of **4a**	Results of **5a**
1	DCE:TFE = 1:5	28% yield, 92% e.e.	56% yield, 92% e.e.
2	THF:TFE = 1:5	18% yield, 92% e.e.	54% yield, 92% e.e.
3	MeOH:TFE = 1:5	12% yield, 92% e.e.	60% yield, 93% e.e.
4	TFE	40% yield, 92% e.e.	40% yield, 92% e.e.

[a]Reactions were performed by using racemic benzoxazolyl acetate **1a** (0.1 mmol), alkene **2a** (0.5 mmol), $Ni(OAc)_2·4H_2O$ (10 mol%), **L5** (11 mol%), **A1** (10 mol%), $(TMS)_3Si$-H (0.5 mmol), 2,6-Lutidine (0.1 mmol), $^nBu_4NPF_6$ (0.1 M) and solvent (3.0 mL) at 25 °C under constant-current conditions (2.0 mA) in an undivided cell. TFE, 2,2,2-trifluoroethanol; CF, carbon felt.

Table S5. Survey of constructing relate tertiary stereocenters.[a]

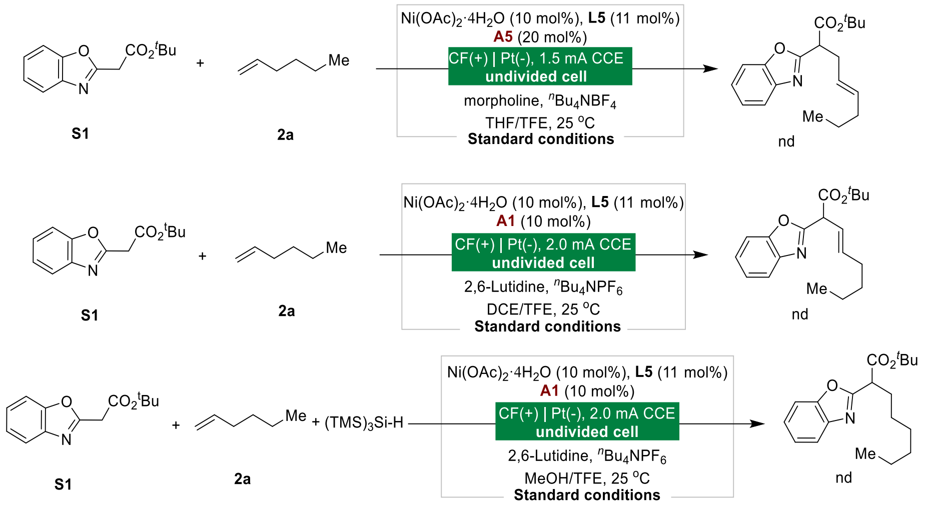

[a]Reactions were performed under the standard conditions.

Table S6. Survey of solvents for dehydrogenative alkenylation.[a]

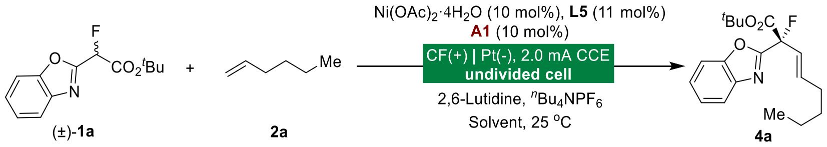

entry	Solvent	Results of **4a**
1	DCE/TFE=0.5/2.5	72% yield, 92% e.e.
2	DCE	n.r.
3	TFE	44% yield, 92% e.e.

[a]Reactions were performed by using racemic benzoxazolyl acetate **1a** (0.1 mmol), alkene **2a** (0.5 mmol), $Ni(OAc)_2 \cdot 4H_2O$ (10 mol%), **L5** (11 mol%), **A1** (10 mol%), 2,6-Lutidine (0.1 mmol), $^nBu_4NPF_6$ (0.1 M), and solvent (3.0 mL) at 25 °C under constant-current conditions (2.0 mA) in an undivided cell. The enantiomeric excess (e.e.) was analyzed via high-performance liquid chromatography (HPLC). TFE, 2,2,2-trifluoroethanol; CF, carbon felt.

Table S7. Survey of bases for dehydrogenative alkenylation.[a]

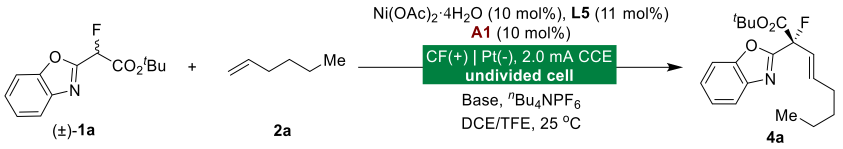

entry	Base	Results of **4a**
1	2,6-Lutidine	72% yield, 92% e.e.
2	morpholine	20% yield, 92% e.e.

[a]Reactions were performed by using racemic benzoxazolyl acetate **1a** (0.1 mmol), alkene **2a** (0.5 mmol), $Ni(OAc)_2{\cdot}4H_2O$ (10 mol%), **L5** (11 mol%), **A1** (10 mol%),base (0.1 mmol), $^{n}Bu_4NPF_6$ (0.1 M), DCE (0.5 mL) and TFE (2.5 mL) at 25 °C under constant-current conditions (2.0 mA) in an undivided cell. The enantiomeric excess (e.e.) was analyzed via high-performance liquid chromatography (HPLC). TFE, 2,2,2-trifluoroethanol; CF, carbon felt.

Table S8. Survey of bases for dehydrogenative allylation.[a]

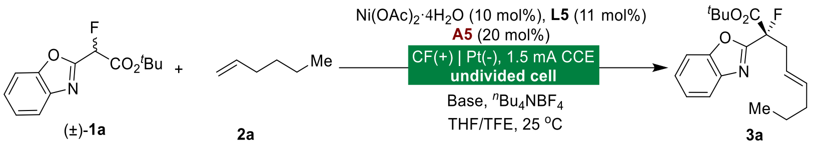

entry	Base	Results of **3a**
1	morpholine	74% yield, 92% e.e.
2	2,6-Lutidine	42% yield, 92% e.e.

[a]Reactions were performed by using racemic benzoxazolyl acetate **1a** (0.1 mmol), alkene **2a** (0.5 mmol), $Ni(OAc)_2{\cdot}4H_2O$ (10 mol%), **L5** (11 mol%), **A5** (20 mol%), base (0.1 mmol), $^{n}Bu_4NBF_4$ (0.1 M), THF (0.5 mL) and TFE (2.5 mL) at 25 °C under constant-current conditions (2.0 mA) in an undivided cell. The enantiomeric excess (e.e.) was analyzed via high-performance liquid chromatography (HPLC). TFE, 2,2,2-trifluoroethanol; CF, carbon felt.

5. Synthesis and characterization of products

Synthetic methods for the chiral nickel catalysts: Under an Ar atmosphere, a mixture of $Ni(OAc)_2·4H_2O$ (1 mmol), **L5** (1.1 mmol), acetonitrile (3 mL), and MeOH (3 mL) was stirred at room temperature for 4 hours. After the solvent the removed, the residue was dissolved in dichloromethane and evaporated to dryness. The resulting preformed nickel complex can be used directly for the subsequent reaction.

General procedure A: Synthesis of product 3

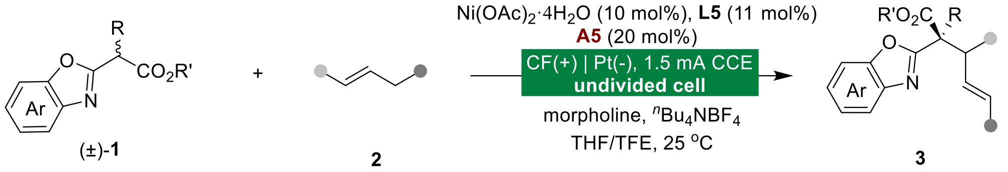

A 10 mL flask equipped with a magnetic stir bar was charged with **1** (0.1 mmol), nickel complex (0.01 mmol), **A5** (0.02 mmol), and $^nBu_4NBF_4$ (0.3 mmol). The flask was equipped with a carbon felt (1.5 cm × 1.5 cm × 3 mm) as the anode and a platinum plate (1.0 cm × 1.0 cm × 0.2 mm) as the cathode. The reaction mixture was degassed via vacuum evacuation and backfilled with argon three times. A solution of **2** (0.5 mmol) and morpholine (0.1 mmol) in THF (0.5 mL) and TFE (2.5 mL) were subsequently added under argon. Constant current (1.5 mA) electrolysis was carried out at 25 °C for 8 h until complete consumption of the starting material (monitored by TLC). The solvent was removed under reduced pressure. The residue was purified by silica gel chromatography to afford desired product **3**.

General procedure B: Synthesis of product 4

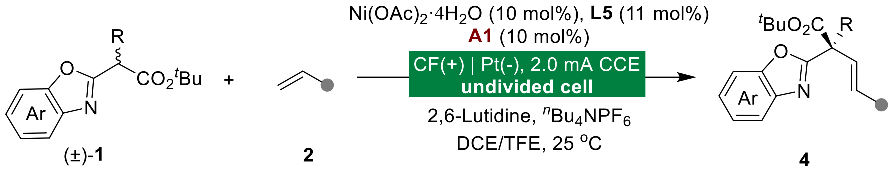

A 10 mL flask equipped with a magnetic stir bar was charged with **1** (0.1 mmol), nickel complex (0.01 mmol), Cp_2Fe-CH_2NMe_2 (**A1**, 0.01 mmol) and $^nBu_4NPF_6$ (0.3 mmol). The flask was equipped with a carbon felt (1.5 cm × 1.5 cm × 3 mm) as the anode and platinum plate (1.0 cm × 1.0 cm × 0.2 mm) as the cathode. The reaction mixture was degassed via vacuum evacuation and backfilled with argon three times. A solution of **2** (0.5 mmol) and 2,6-Lutidine (0.1 mmol) in DCE (0.5 mL) and TFE (2.5 mL) was subsequently added under argon. Constant current (2.0 mA) electrolysis was carried out at 25 °C for 6 h until complete consumption of the starting material (monitored by TLC). The solvent was removed under reduced pressure. The residue was purified by silica gel chromatography to afford desired product **4**.

General procedure C: Synthesis of product 5

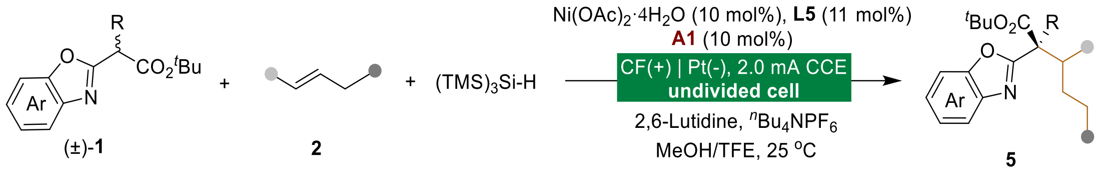

A 10 mL flask equipped with a magnetic stir bar was charged with **1** (0.1 mmol), nickel complex (0.01 mmol), $Cp_2Fe-CH_2NMe_2$ (**A1**, 0.01 mmol) and $^nBu_4NPF_6$ (0.3 mmol). The flask was equipped with a carbon felt (1.5 cm × 1.5 cm × 3 mm) as the anode and platinum plate (1.0 cm × 1.0 cm × 0.2 mm) as the cathode. The reaction mixture was degassed via vacuum evacuation and backfilled with argon three times. A solution of **2** (2.0 mmol), $(TMS)_3SiH$ (0.5 mmol), and 2,6-Lutidine (0.1 mmol) in MeOH (0.5 mL) and TFE (2.5 mL) were subsequently added under argon. Constant current (2.0 mA) electrolysis was carried out at 25 °C for 8 h until complete consumption of the starting material (monitored by TLC). The solvent was removed under reduced pressure. The residue was purified by silica gel chromatography to afford desired product **5**.

***Tert*-butyl (*R*,*E*)-2-(benzo[*d*]oxazol-2-yl)-2-fluorooct-4-enoate (3a)**

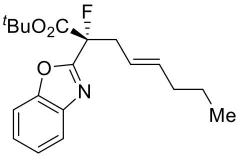

The title compound was prepared according to general procedure A using **1a** (0.1 mmol) and **2a** (0.5 mmol). The crude reaction mixture was purified by flash column chromatography to afford the title compound (petroleum ether/ethyl acetate, 25:1). (74% yield, *E*:*Z* = 6:1). **^{1}H NMR (400 MHz, $CDCl_3$)** δ 7.83–7.74 (m, 1H), 7.62–7.53 (m, 1H), 7.45 – 7.34 (m, 2H), 5.76 – 5.62 (m, 1H), 5.59–5.47 (m, 1H), 3.39–3.07 (m, 2H), 2.06 – 1.94 (m, 2H), 1.47 (s, 9H), 1.41–1.32 (m, 2H), 0.87 (t, *J* = 7.4 Hz, 3H). **^{13}C NMR (151 MHz, $CDCl_3$)** δ 165.32 (d, *J* = 27.5 Hz), 160.77 (d, *J* = 25.4 Hz), 150.95, 140.55, 137.24, 126.27, 124.98, 121.06, 120.62 (d, *J* = 2.8 Hz), 111.25, 91.79 (d, *J* = 190.6 Hz), 84.33, 38.68 (d, *J* = 21.3 Hz), 34.81, 28.04, 22.38, 13.75. **^{19}F NMR (376 MHz, $CDCl_3$)** δ -158.18. **ESI-MS: calculated [$C_{19}H_{24}FNO_3$ + Na]$^+$: 356.1632, found: 356.1626.** $[\alpha]^{20}_D$ = -15.21 (c = 7.97, CH_2Cl_2). The product was analyzed by HPLC to determine the enantiomeric excess: 92% e.e. (CHIRALPAK IG, hexane/*i*-PrOH = 99/1, detector: 254 nm, T = 25 °C, flow rate: 1 mL/min), t_1(minor) = 8.26 min, t_2(major) = 9.34 min.

***Tert*-butyl (*R*,*E*)-2-(benzo[*d*]oxazol-2-yl)-8-chloro-2-fluorooct-4-enoate (3b)**

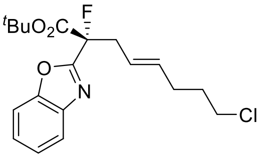

The title compound was prepared according to general procedure A using **1a** (0.1 mmol) and **2b** (0.5 mmol). The crude reaction mixture was purified by flash column chromatography to afford the title compound (petroleum ether/ethyl acetate, 25:1). (76% yield, *E*:*Z* = 6:1). **^{1}H NMR (500 MHz, $CDCl_3$)** δ 7.82–7.76 (m, 1H), 7.60–7.55 (m, 1H), 7.45–7.35 (m, 2H), 5.72–5.54 (m, 2H), 3.50 (t, *J* = 6.6 Hz, 2H), 3.35–3.10 (m, 2H), 2.23–2.14 (m, 2H), 1.89–1.78 (m, 2H), 1.47 (s, 9H). **^{13}C NMR (126 MHz, $CDCl_3$)** δ 165.25 (d, *J* = 27.3 Hz), 160.58 (d, *J* = 25.3 Hz), 150.92, 140.49, 135.24, 126.33, 125.02, 122.20 (d, *J* = 3.0 Hz), 121.06, 111.25, 91.62 (d, *J* = 191.1 Hz), 84.47, 44.31, 38.56 (d, *J* = 21.5 Hz), 31.91, 29.75, 28.03.**^{19}F NMR (471 MHz, $CDCl_3$)** δ -158.16. **ESI-MS: calculated [$C_{19}H_{23}ClFNO_3$ + H]$^+$: 368.1423, found: 368.1424.** $[\alpha]^{20}_D$ = -12.90 (c = 8.37, CH_2Cl_2). The product was analyzed by HPLC to determine the enantiomeric excess: 92% e.e. (CHIRALPAK IG, hexane/*i*-PrOH = 98/2, detector: 252.2 nm, T = 25 °C, flow rate: 1 mL/min), t_1(minor) = 10.31 min, t_2(major) = 11.43 min.

***Tert*-butyl (*R*,*E*)-2-(benzo[*d*]oxazol-2-yl)-8-bromo-2-fluorooct-4-enoate (3c)**

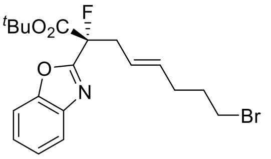

The title compound was prepared according to general procedure A using **1a** (0.1 mmol) and **2c** (0.5 mmol). The crude reaction mixture was purified by flash column chromatography to afford the title compound (petroleum ether/ethyl acetate, 25:1). (74% yield, *E*:*Z* = 6:1). **^{1}H NMR (500 MHz, $CDCl_3$)** δ 7.82–7.76 (m, 1H), 7.60–7.55 (m, 1H), 7.46–7.35 (m, 2H), 5.72–5.56 (m, 2H), 3.37 (t, *J* = 6.7 Hz, 2H), 3.29–3.10 (m, 2H), 2.24–2.14 (m, 2H), 1.95–1.85 (m, 2H), 1.47 (s, 9H). **^{13}C NMR (126 MHz, $CDCl_3$)** δ 165.24 (d, *J* = 27.4 Hz), 160.57 (d, *J* = 25.4 Hz), 150.92, 140.49, 135.06, 126.33, 125.02, 122.30 (d, *J* = 2.8 Hz), 121.06, 111.25, 91.61 (d, *J* = 191.0 Hz), 84.48, 38.56 (d, *J* = 21.5 Hz), 33.10, 32.02, 30.99, 28.04. **^{19}F NMR (471 MHz, $CDCl_3$)** δ -158.17. **ESI-MS: calculated [$C_{19}H_{23}BrFNO_3$ + H]$^+$: 412.0918, found: 412.0913.** $[\alpha]^{20}_{D}$ = -7.39 (c = 9.13, CH_2Cl_2). The product was analyzed by HPLC to determine the enantiomeric excess: 91% e.e. (CHIRALPAK IG, hexane/*i*-PrOH = 95/5, detector: 254 nm, T = 25 °C, flow rate: 1 mL/min), t_1(minor) = 7.02 min, t_2(major) = 7.68 min.

***Tert*-butyl (*R*,*E*)-2-(benzo[*d*]oxazol-2-yl)-2-fluoro-8-hydroxyoct-4-enoate (3d)**

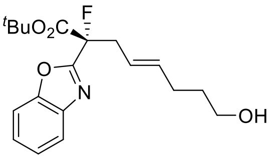

The title compound was prepared according to general procedure A using **1a** (0.1 mmol) and **2d** (0.5 mmol). The crude reaction mixture was purified by flash column chromatography to afford the title compound (petroleum ether/ethyl acetate, 2:1). (55% yield, *E*:*Z* = 8:1). **^{1}H NMR (400 MHz, $CDCl_3$)** δ 7.84–7.74 (m, 1H), 7.61–7.54 (m, 1H), 7.47–7.34 (m, 2H), 5.78–5.66 (m, 1H), 5.65–5.50 (m, 1H), 3.62 (t, *J* = 6.5 Hz, 2H), 3.35–3.07 (m, 2H), 2.13 (q, *J* = 7.2 Hz, 2H), 1.67 (s, 1H), 1.66–1.59 (m, 2H), 1.47 (s, 9H). **^{13}C NMR (101 MHz, $CDCl_3$)** δ 165.31 (d, *J* = 27.5 Hz), 160.64 (d, *J* = 25.3 Hz), 150.92, 140.49, 136.52, 126.32, 125.02, 121.27 (d, *J* = 3.0 Hz), 121.06, 111.24, 91.68 (d, *J* = 190.6 Hz), 84.46, 62.35, 38.57 (d, *J* = 21.5 Hz), 32.03, 29.06, 28.04. **^{19}F NMR (376 MHz, $CDCl_3$)** δ -158.07. **ESI-MS: calculated [$C_{19}H_{24}FNO_4$ + Na]$^+$: 372.1582, found: 372.1577.** $[\alpha]^{20}_{D}$ = -11.23 (c = 2.67, CH_2Cl_2). The product was analyzed by HPLC to determine the enantiomeric excess: 93% e.e. (CHIRALPAK IG, hexane/*i*-PrOH = 90/10, detector: 233 nm, T = 25 °C, flow rate: 1 mL/min), t_1(minor) = 13.46 min, t_2(major) = 15.71 min.

***Tert*-butyl (*R*,*E*)-2-(benzo[*d*]oxazol-2-yl)-2-fluoro-8-(tosyloxy)oct-4-enoate (3e)**

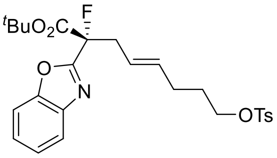

The title compound was prepared according to general procedure A using **1a** (0.1 mmol) and **2e** (0.5 mmol). The crude reaction mixture was purified by flash column chromatography to afford the title compound (petroleum ether/ethyl acetate, 10:1). (64% yield, *E*:*Z* = 5:1). **^{1}H NMR (400 MHz, $CDCl_3$)** δ 7.82–7.74 (m, 3H), 7.61–7.54 (m, 1H), 7.44–7.37 (m, 2H), 7.36–7.31 (m, 2H), 5.63–5.42 (m, 2H), 4.09–3.93 (m, 2H), 3.27–3.01 (m, 2H), 2.44 (s, 3H), 2.06 (q, *J* = 7.1 Hz, 2H), 1.79–1.64 (m, 2H), 1.45 (s, 9H). **^{13}C NMR (101 MHz, $CDCl_3$)** δ 165.17 (d, *J* = 27.5 Hz), 160.52 (d, *J* = 25.3 Hz), 150.91, 144.87, 140.48, 134.95, 133.24, 129.99, 128.02, 126.35, 125.03, 122.28 (d, *J* = 3.0 Hz), 121.06, 111.24, 91.53 (d, *J* = 190.9 Hz), 84.46, 69.82, 38.47 (d, *J* = 21.5 Hz), 28.44, 28.41, 28.02, 21.76. **^{19}F NMR (376 MHz, $CDCl_3$)** δ -158.14. **ESI-MS: calculated [$C_{26}H_{30}FNO_6S$ + Na]$^+$: 526.1670, found: 526.1674.** $[\alpha]^{20}_{D}$ = -7.44 (c = 6.07, CH_2Cl_2). The product was analyzed by HPLC to determine the enantiomeric excess: 91% e.e. (CHIRALPAK IG, hexane/*i*-PrOH = 70/30, detector: 254 nm, T = 25 °C, flow rate: 1 mL/min), t_1(minor) = 16.34 min, t_2(major) = 20.00 min.

***Tert*-butyl (*R*,*E*)-2-(benzo[*d*]oxazol-2-yl)-8-((*tert*-butyldimethylsilyl)oxy)-2-fluorooct-4-enoate (3f)**

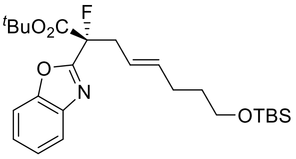

The title compound was prepared according to general procedure A using **1a** (0.1 mmol) and **2f** (0.5 mmol). The crude reaction mixture was purified by flash column chromatography to afford the title compound (petroleum ether/ethyl acetate, 25:1). (78% yield, *E*:*Z* = 9:1). **^{1}H NMR (400 MHz, $CDCl_3$)** δ 7.83–7.76 (m, 1H), 7.61 – 7.54 (m, 1H), 7.45–7.32 (m, 2H), 5.77–5.64 (m, 1H), 5.61–5.49 (m, 1H), 3.58 (t, *J* = 6.4 Hz, 2H), 3.33–3.07 (m, 2H), 2.09 (q, *J* = 7.2 Hz, 2H), 1.63–1.52 (m, 2H), 1.47 (s, 9H), 0.88 (s, 9H), 0.03 (s, 6H). **^{13}C NMR (151 MHz, $CDCl_3$)** δ 165.29 (d, *J* = 27.5 Hz), 160.71 (d, *J* = 25.2 Hz), 150.94, 140.52, 136.89, 126.29, 124.99, 121.07, 120.81 (d, *J* = 2.9 Hz), 111.26, 91.74 (d, *J* = 190.5 Hz), 84.36, 62.57, 38.65 (d, *J* = 21.2 Hz), 32.33, 29.04, 28.05, 26.09, 18.46, -5.16. **^{19}F NMR (376 MHz, $CDCl_3$)** δ -158.14. **ESI-MS: calculated [$C_{25}H_{38}FNO_4Si$ + Na]$^+$: 486.2446, found: 468.2447.** $[\alpha]^{20}_D$ = -11.58 (c = 22.93, CH_2Cl_2). The product was analyzed by HPLC to determine the enantiomeric excess: 89% e.e. (CHIRALPAK IG, hexane/*i*-PrOH = 98/2, detector: 254 nm, T = 25 °C, flow rate: 1 mL/min), t_1(minor) = 4.43 min, t_2(major) = 4.83 min.

***Tert*-butyl (*R*,*E*)-2-(benzo[*d*]oxazol-2-yl)-2-fluoro-8-phenoxyoct-4-enoate (3g)**

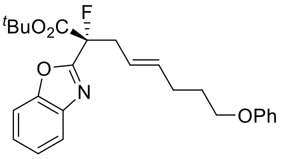

The title compound was prepared according to general procedure A using **1a** (0.1 mmol) and **2g** (0.5 mmol). The crude reaction mixture was purified by flash column chromatography to afford the title compound (petroleum ether/ethyl acetate, 25:1). (55% yield, *E*:*Z* = 5:1). **^{1}H NMR (400 MHz, $CDCl_3$)** δ 7.81–7.74 (m, 1H), 7.59–7.52 (m, 1H), 7.44–7.32 (m, 2H), 7.30–7.20 (m, 2H), 6.95–6.83 (m, 3H), 5.80–5.67 (m, 1H), 5.65–5.53 (m, 1H), 3.92 (t, *J* = 6.4 Hz, 2H), 3.36–3.06 (m, 2H), 2.22 (q, *J* = 7.2 Hz, 2H), 1.92–1.79 (m, 2H), 1.45 (s, 9H). **^{13}C NMR (151 MHz, $CDCl_3$)** δ 165.27 (d, *J* = 27.5 Hz), 160.62 (d, *J* = 25.3 Hz), 159.08, 150.91, 140.47, 136.21, 129.53, 126.31, 125.00, 121.49 (d, *J* = 3.0 Hz), 121.05, 120.65, 114.60, 111.25, 91.66 (d, *J* = 190.4 Hz), 84.42, 67.02, 38.58 (d, *J* = 21.6 Hz), 29.15, 28.78, 28.01. **^{19}F NMR (376 MHz, $CDCl_3$)** δ -158.12. **ESI-MS: calculated [$C_{25}H_{28}FNO_4$ + H]$^+$: 426.2075, found: 426.2076.** $[\alpha]^{20}_D$ = -13.40 (c = 3.03, CH_2Cl_2). The product was analyzed by HPLC to determine the enantiomeric excess: 92% e.e. (CHIRALPAK IG, hexane/*i*-PrOH = 98/2, detector: 254 nm, T = 25 °C, flow rate: 1 mL/min), t_1(minor) = 15.17 min, t_2(major) = 17.76 min.

(*R*,*E*)-5-(benzo[*d*]oxazol-2-yl)-6-(*tert*-butoxy)-5-fluoro-6-oxohex-2-en-1-yl thiophene-2-carboxylate (3h)

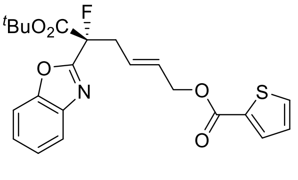

The title compound was prepared according to general procedure A using **1a** (0.1 mmol) and **2h** (0.5 mmol). The crude reaction mixture was purified by flash column chromatography to afford the title compound (petroleum ether/ethyl acetate, 25:1). (54% yield, *E*:*Z* > 20:1). **^{1}H NMR (600 MHz, $CDCl_3$)** δ 7.81–7.76 (m, 2H), 7.60–7.53 (m, 2H), 7.45–7.36 (m, 2H), 7.09 (dd, *J* = 5.0, 3.7 Hz, 1H), 6.02–5.91 (m, 2H), 4.83–4.73 (m, 2H), 3.39–3.21 (m, 2H), 1.47 (s, 9H). **^{13}C NMR (151 MHz, $CDCl_3$)** δ 165.01 (d, *J* = 27.4 Hz), 162.00, 160.28 (d, *J* = 24.8 Hz), 150.96, 140.44, 133.71, 133.66, 132.64, 130.26, 127.87, 126.44, 126.25 (d, *J* = 3.1 Hz), 125.08, 121.11, 111.30, 91.28 (d, *J* = 191.3 Hz), 84.78, 65.03, 38.24 (d, *J* = 21.6 Hz), 28.02. **^{19}F NMR (376 MHz, $CDCl_3$)** δ -157.49. **ESI-MS: calculated [$C_{22}H_{22}FNO_5S$ + Na]$^+$: 454.1095, found: 454.1090.** $[\alpha]^{20}_D$ = -9.38 (c = 10.10, CH_2Cl_2). The product was analyzed by HPLC to determine the enantiomeric excess: 89% e.e.

(CHIRALPAK IG, hexane/*i*-PrOH = 80/20, detector: 254 nm, T = 25 °C, flow rate: 1 mL/min), t_1(minor) = 9.50 min, t_2(major) = 11.31 min.

(*R*,*E*)-5-(benzo[*d*]oxazol-2-yl)-6-(*tert*-butoxy)-5-fluoro-6-oxohex-2-en-1-yl furan-2-carboxylate (3i)

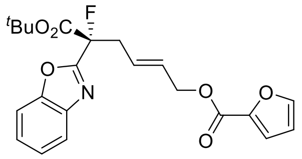

The title compound was prepared according to general procedure A using **1a** (0.1 mmol) and **2i** (0.5 mmol). The crude reaction mixture was purified by flash column chromatography to afford the title compound (petroleum ether/ethyl acetate, 25:1). (55% yield, *E*:*Z* > 20:1). **^{1}H NMR (400 MHz, $CDCl_3$)** δ 7.83–7.74 (m, 1H), 7.60–7.55 (m, 2H), 7.46–7.35 (m, 2H), 7.16 (dd, *J* = 3.6, 0.8 Hz, 1H), 6.50 (dd, *J* = 3.5, 1.7 Hz, 1H), 6.05–5.88 (m, 2H), 4.84–4.72 (m, 2H), 3.41–3.18 (m, 2H), 1.47 (s, 9H). **^{13}C NMR (151 MHz, $CDCl_3$)** 165.01 (d, *J* = 27.4 Hz), 160.27 (d, *J* = 25.1 Hz), 158.46, 150.95, 146.52, 144.62, 140.44, 130.07, 126.63 (d, *J* = 3.1 Hz), 126.44, 125.09, 121.10, 118.22, 111.98, 111.29, 91.27 (d, *J* = 191.7 Hz), 84.80, 64.79, 38.21 (d, *J* = 21.6 Hz), 27.98. **^{19}F NMR (376 MHz, $CDCl_3$)** δ -157.50. **ESI-MS: calculated [$C_{22}H_{22}FNO_6$ + Na]$^+$: 438.1323, found: 438.132.** $[\alpha]^{20}_D$ = -11.27 (c = 6.47, CH_2Cl_2). The product was analyzed by HPLC to determine the enantiomeric excess: 90% e.e. (CHIRALPAK IG, hexane/*i*-PrOH = 80/20, detector: 254 nm, T = 25 °C, flow rate: 1 mL/min), t_1(minor) = 11.31 min, t_2(major) = 14.25 min.

(*R*,*E*)-5-(benzo[*d*]oxazol-2-yl)-6-(*tert*-butoxy)-5-fluoro-6-oxohex-2-en-1-yl cyclopropanecarboxylate (3j)

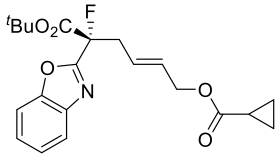

The title compound was prepared according to general procedure A using **1a** (0.1 mmol) and **2j** (0.5 mmol). The crude reaction mixture was purified by flash column chromatography to afford the title compound (petroleum ether/ethyl acetate, 25:1). (65% yield, *E*:*Z* > 20:1). **^{1}H NMR (400 MHz, $CDCl_3$)** δ 7.83–7.76 (m, 1H), 7.61–7.53 (m, 1H), 7.44–7.36 (m, 2H), 5.97–5.78 (m, 2H), 4.62–4.49 (m, 2H), 3.38–3.15 (m, 2H), 1.66–1.54 (m, 1H), 1.48 (s, 9H), 1.01–0.95 (m, 2H), 0.89–0.80 (m, 2H). **^{13}C NMR (151 MHz, $CDCl_3$)** 174.66, 165.03 (d, *J* = 27.5 Hz), 160.32 (d, *J* = 24.8 Hz), 150.96, 140.46, 130.61, 126.44, 125.70 (d, *J* = 2.9 Hz), 125.09, 121.11, 111.30, 91.31 (d, *J* = 191.4 Hz), 84.73, 64.44, 38.22 (d, *J* = 21.5 Hz), 28.01, 12.93, 8.60. **^{19}F NMR (376 MHz, $CDCl_3$)** δ -157.53. **ESI-MS: calculated [$C_{21}H_{24}FNO_5$ + Na]$^+$: 412.1531, found: 412.1530.** $[\alpha]^{20}_D$ = -10.83 (c = 6.53, CH_2Cl_2). The product was analyzed by HPLC to determine the enantiomeric excess: 90% e.e. (CHIRALPAK IG, hexane/*i*-PrOH = 80/20, detector: 254 nm, T = 25 °C, flow rate: 1 mL/min), t_1(minor) = 6.25 min, t_2(major) = 7.24 min.

***Tert*-butyl (*R*,*E*)-2-(benzo[*d*]oxazol-2-yl)-7-(1,3-dioxoisoindolin-2-yl)-2-fluorohept-4-enoate (3k)**

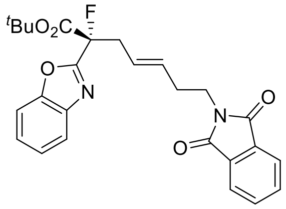

The title compound was prepared according to general procedure A using **1a** (0.1 mmol) and **2k** (0.5 mmol). The crude reaction mixture was purified by flash column chromatography to afford the title compound (petroleum ether/ethyl acetate, 4:1). (72% yield, *E*:*Z* = 5:1). **^{1}H NMR (400 MHz, $CDCl_3$)** δ 7.85–7.75 (m, 3H), 7.73–7.67 (m, 2H), 7.61–7.51 (m, 1H), 7.45–7.33 (m, 2H), 5.77–5.56 (m, 2H), 3.81–3.64 (m, 2H), 3.32–3.04 (m, 2H), 2.50–2.36 (m, 2H), 1.44 (s, 9H). **^{13}C NMR (151 MHz, $CDCl_3$)** δ 168.38, 165.18 (d, *J* = 27.5 Hz), 160.49 (d, *J* = 25.2 Hz), 150.92, 140.48, 134.02, 132.70, 132.21, 126.31, 125.00, 123.95 (d, *J* = 2.9 Hz), 123.34, 121.07, 111.25, 91.40 (d, *J* = 191.2 Hz), 84.49, 38.53 (d, *J* = 21.6 Hz), 37.43, 31.85, 28.00. **^{19}F NMR**

(376 MHz, $CDCl_3$) δ -158.16. **ESI-MS: calculated [$C_{26}H_{25}FN_2O_5$ + H]$^+$: 465.1820, found: 465.1817.** $[\alpha]^{20}_D$ = -2.16 (c = 9.30, CH_2Cl_2). The product was analyzed by HPLC to determine the enantiomeric excess: 90% e.e. (CHIRALPAK IE, hexane/*i*-PrOH = 85/15, detector: 254 nm, T = 25 °C, flow rate: 1 mL/min), t_1(major) = 23.13 min, t_2(minor) = 25.13 min.

***Tert*-butyl (*R*,*E*)-2-(benzo[*d*]oxazol-2-yl)-2-fluoro-7-oxo-7-(phenylamino)hept-4-enoate (3l)**

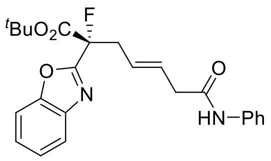

The title compound was prepared according to general procedure A using **1a** (0.1 mmol) and **2l** (0.5 mmol). The crude reaction mixture was purified by flash column chromatography to afford the title compound (petroleum ether/ethyl acetate, 4:1). (52% yield, *E*:*Z* > 20:1). **^{1}H NMR (400 MHz, $CDCl_3$)** δ 7.78–7.72 (m, 1H), 7.60 (s, 1H), 7.59–7.55 (m, 3H), 7.47–7.34 (m, 2H), 7.32–7.27 (m, 2H), 7.13–7.05 (m, 1H), 6.00–5.79 (m, 2H), 3.47–2.96 (m, 4H), 1.45 (s, 9H). **^{13}C NMR (151 MHz, $CDCl_3$)** δ 168.49, 165.33 (d, *J* = 27.6 Hz), 160.15 (d, *J* = 25.1 Hz), 150.93, 140.36, 137.99, 129.75, 129.01, 127.70 (d, *J* = 3.5 Hz), 126.56, 125.17, 124.42, 121.14, 120.05, 111.29, 91.30 (d, *J* = 190.3 Hz), 85.13, 41.85, 38.23 (d, *J* = 21.6 Hz), 27.99. **^{19}F NMR (376 MHz, $CDCl_3$)** δ -156.47. **ESI-MS: calculated [$C_{24}H_{25}FN_2O_4$ + Na]$^+$: 447.1691, found: 447.1689.** $[\alpha]^{20}_D$ = -13.27 (c = 5.37, CH_2Cl_2). The product was analyzed by HPLC to determine the enantiomeric excess: 90% e.e. (CHIRALPAK IG, hexane/*i*-PrOH = 80/20, detector: 254 nm, T = 25 °C, flow rate: 1 mL/min), t_1(minor) = 9.75 min, t_2(major) = 10.79 min.

***Tert*-butyl (*R*,*E*)-2-(benzo[*d*]oxazol-2-yl)-2-fluoro-6-phenylhex-4-enoate (3m)**

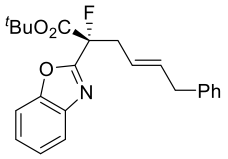

The title compound was prepared according to general procedure A using **1a** (0.1 mmol) and **2m** (0.5 mmol). The crude reaction mixture was purified by flash column chromatography to afford the title compound (petroleum ether/ethyl acetate, 25:1). (78% yield, *E*:*Z* > 20:1). **^{1}H NMR (400 MHz, $CDCl_3$)** δ 7.83–7.74 (m, 1H), 7.59–7.54 (m, 1H), 7.39 (tt, *J* = 7.4, 5.8 Hz, 2H), 7.27–7.21 (m, 2H), 7.19–7.12 (m, 3H), 5.92–5.81 (m, 1H), 5.70–5.58 (m, 1H), 3.41–3.33 (m, 2H), 3.33–3.12 (m, 2H), 1.42 (s, 9H). **^{13}C NMR (151 MHz, $CDCl_3$)** δ 165.23 (d, *J* = 27.4 Hz), 160.60 (d, *J* = 25.4 Hz), 150.92, 140.49, 139.91, 135.76, 128.66, 128.52, 126.30, 126.20, 125.00, 122.14 (d, *J* = 2.9 Hz), 121.05, 111.25, 91.69 (d, *J* = 190.8 Hz), 84.47, 39.16, 38.52 (d, *J* = 21.5 Hz), 27.96. **^{19}F NMR (376 MHz, $CDCl_3$)** δ -158.07. **ESI-MS: calculated [$C_{23}H_{24}FNO_3$ + H]$^+$: 382.1813, found: 382.1806.** $[\alpha]^{20}_D$ = -13.11 (c = 7.97, CH_2Cl_2). The product was analyzed by HPLC to determine the enantiomeric excess: 91% e.e. (CHIRALPAK IG, hexane/*i*-PrOH = 98/2, detector: 254 nm, T = 25 °C, flow rate: 1 mL/min), t_1(minor) = 10.35 min, t_2(major) = 11.65 min.

***Tert*-butyl (*R*,*E*)-2-(benzo[*d*]oxazol-2-yl)-2-fluoro-5-phenylpent-4-enoate (3n)**

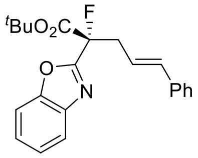

The title compound was prepared according to general procedure A using **1a** (0.1 mmol) and **2n** (0.5 mmol). The crude reaction mixture was purified by flash column chromatography to afford the title compound (petroleum ether/ethyl acetate, 25:1). (63% yield, *E*:*Z* > 20:1). **^{1}H NMR (400 MHz, $CDCl_3$)** δ 7.85–7.77 (m, 1H), 7.64–7.56 (m, 1H), 7.46–7.39 (m, 2H), 7.39–7.35 (m, 2H), 7.33–7.28 (m, 2H), 7.26–7.21 (m, 1H), 6.63 (d, *J* = 15.8 Hz, 1H), 6.31 (ddd, *J* = 15.8, 8.2, 6.3 Hz, 1H), 3.54–3.31 (m, 2H), 1.45 (s, 9H). **^{13}C NMR (126 MHz, $CDCl_3$)** δ 165.22 (d, *J* = 27.4 Hz), 160.49 (d, *J* = 25.1 Hz), 150.98, 140.50, 136.81, 135.83, 128.69, 127.86, 126.52, 126.41, 125.07, 121.12, 120.66 (d, *J* = 2.9 Hz), 111.32, 91.66 (d, *J* =

191.4 Hz), 84.66, 39.03 (d, *J* = 21.3 Hz), 28.03. **^{19}F NMR (376 MHz, $CDCl_3$)** δ -157.18. **ESI-MS: calculated [$C_{22}H_{22}FNO_3$ + H]$^+$: 368.1656, found: 368.1651.** $[\alpha]^{20}_D$ = -30.49 (c = 9.93, CH_2Cl_2). The product was analyzed by HPLC to determine the enantiomeric excess: 91% e.e. (CHIRALPAK IG, hexane/*i*-PrOH = 98/2, detector: 254 nm, T = 25 °C, flow rate: 1 mL/min), t_1(minor) = 12.95 min, t_2(major) = 14.63 min.

***Tert*-butyl (*R*)-2-(benzo[*d*]oxazol-2-yl)-4-cyclohexylidene-2-fluorobutanoate (3o)**

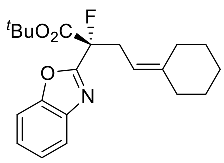

The title compound was prepared according to general procedure A using **1a** (0.1 mmol) and **2o** (0.5 mmol). The crude reaction mixture was purified by flash column chromatography to afford the title compound (petroleum ether/ethyl acetate, 25:1). (66% yield). **^{1}H NMR (500 MHz, $CDCl_3$)** δ 7.82–7.77 (m, 1H), 7.60–7.55 (m, 1H), 7.44–7.33 (m, 2H), 5.26–5.20 (m, 1H), 3.44–3.03 (m, 2H), 2.30 – 2.23 (m, 1H), 2.19–2.05 (m, 3H), 1.58 – 1.49 (m, 6H), 1.47 (s, 9H). **^{13}C NMR (151 MHz, $CDCl_3$)** δ 165.58 (d, *J* = 27.4 Hz), 160.96 (d, *J* = 25.5 Hz), 150.94, 145.72, 140.56, 126.24, 124.97, 121.04, 111.36 (d, *J* = 2.7 Hz), 111.24, 92.01 (d, *J* = 190.9 Hz), 84.27, 37.42, 33.42, 33.28, 29.26, 27.99, 27.75, 26.85. **^{19}F NMR (471 MHz, $CDCl_3$)** δ -158.61. **ESI-MS: calculated [$C_{21}H_{26}FNO_3$ + H]$^+$: 360.1969, found: 360.1968.** $[\alpha]^{20}_D$ = -8.11 (c = 6.70, CH_2Cl_2). The product was analyzed by HPLC to determine the enantiomeric excess: 89% e.e. (CHIRALPAK IG, hexane/*i*-PrOH = 98/2, detector: 254 nm, T = 25 °C, flow rate: 1 mL/min), t_1(minor) = 7.10 min, t_2(major) = 7.91 min.

***Tert*-butyl (*R*)-2-(benzo[*d*]oxazol-2-yl)-2-fluoro-4-methylpent-4-enoate (3p)**

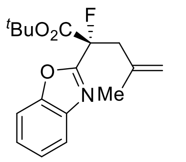

The title compound was prepared according to general procedure A using **1a** (0.1 mmol) and **2p** (0.5 mmol). The crude reaction mixture was purified by flash column chromatography to afford the title compound (petroleum ether/ethyl acetate, 25:1). (89% yield). **^{1}H NMR (400 MHz, $CDCl_3$)** δ 7.83–7.76 (m, 1H), 7.61–7.54 (m, 1H), 7.46–7.34 (m, 2H), 5.01–4.92 (m, 2H), 3.38–3.18 (m, 2H), 1.88 (q, *J* = 1.4 Hz, 3H), 1.47 (s, 9H). **^{13}C NMR (151 MHz, $CDCl_3$)** δ 165.17 (d, *J* = 27.5 Hz), 160.89 (d, *J* = 25.3 Hz), 150.93, 140.48, 138.68, 126.32, 125.02, 121.06, 116.70, 111.26, 92.24 (d, *J* = 193.6 Hz), 84.52, 42.62 (d, *J* = 20.3 Hz), 27.99, 23.80 (d, *J* = 3.0 Hz). **^{19}F NMR (376 MHz, $CDCl_3$)** δ -156.52. **ESI-MS: calculated [$C_{17}H_{20}FNO_3$ + Na]$^+$: 328.1319, found: 328.1320.** $[\alpha]^{20}_D$ = -15.42 (c = 10.43, CH_2Cl_2). The product was analyzed by HPLC to determine the enantiomeric excess: 91% e.e. (CHIRALPAK IG, hexane/*i*-PrOH = 98/2, detector: 254 nm, T = 25 °C, flow rate: 1 mL/min), t_1(minor) = 6.58 min, t_2(major) = 8.44 min.

***Tert*-butyl (*R*)-2-(benzo[*d*]oxazol-2-yl)-3-(cyclohex-1-en-1-yl)-2-fluoropropanoate (3q)**

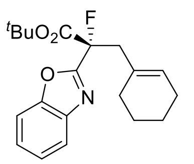

The title compound was prepared according to general procedure A using **1a** (0.1 mmol) and **2q** (0.5 mmol). The crude reaction mixture was purified by flash column chromatography to afford the title compound (petroleum ether/ethyl acetate, 25:1). (88% yield). **^{1}H NMR (400 MHz, $CDCl_3$)** δ 7.82–7.75 (m, 1H), 7.61–7.54 (m, 1H), 7.44–7.33 (m, 2H), 5.66 (s, 1H), 3.29–3.08 (m, 2H), 2.29–2.15 (m, 1H), 2.05–1.92 (m, 3H), 1.70–1.52 (m, 4H), 1.46 (s, 9H). **^{13}C NMR (151 MHz, $CDCl_3$)** δ 165.31 (d, *J* = 27.6 Hz), 161.15 (d, *J* = 25.3 Hz), 150.93, 140.54, 131.27, 127.95, 126.23, 124.95, 121.04, 111.24, 92.77 (d, *J* = 193.1 Hz), 84.19, 43.37 (d, *J* = 20.2 Hz), 29.49 (d, *J* = 2.9 Hz), 28.01, 25.56, 22.99, 22.10. **^{19}F NMR (376 MHz, $CDCl_3$)** δ -154.76. **ESI-MS: calculated [$C_{20}H_{24}FNO_3$ + H]$^+$: 346.1813, found: 346.1808.** $[\alpha]^{20}_D$ = -36.74 (c = 10.13, CH_2Cl_2). The product was analyzed by HPLC to determine the enantiomeric excess:

90% e.e. (CHIRALPAK IG, hexane/*i*-PrOH = 98/2, detector: 254 nm, T = 25 °C, flow rate: 1 mL/min), t_1(minor) = 9.32 min, t_2(major) = 11.60 min.

***Tert*-butyl (*R*)-2-(benzo[*d*]oxazol-2-yl)-2-fluoro-3-(1,4-dioxaspiro[4.5]dec-7-en-8-yl)propanoate (3r)**

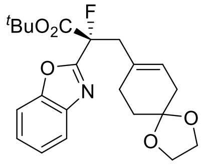

The title compound was prepared according to general procedure A using **1a** (0.1 mmol) and **2r** (0.5 mmol). The crude reaction mixture was purified by flash column chromatography to afford the title compound (petroleum ether/ethyl acetate, 10:1). (82% yield). **^{1}H NMR (400 MHz, $CDCl_3$)** δ 7.84 – 7.73 (m, 1H), 7.63–7.51 (m, 1H), 7.45–7.33 (m, 2H), 5.62–5.55 (m, 1H), 3.97 (s, 4H), 3.39–3.11 (m, 2H), 2.59–2.42 (m, 1H), 2.35–2.17 (m, 3H), 1.77 (t, *J* = 6.4 Hz, 2H), 1.48 (s, 9H). **^{13}C NMR (151 MHz, $CDCl_3$)** δ 165.25 (d, *J* = 27.0 Hz), 160.96 (d, *J* = 25.3 Hz), 150.95, 140.52, 131.01, 126.28, 125.19, 124.99, 121.07, 111.27, 107.63, 92.65 (d, *J* = 193.9 Hz), 84.46, 64.50, 64.45, 42.19 (d, *J* = 20.3 Hz), 35.96, 31.33, 28.52 (d, *J* = 3.4 Hz), 27.98. **^{19}F NMR (376 MHz, $CDCl_3$)** δ -154.91. **ESI-MS: calculated [$C_{22}H_{26}FNO_5$ + H]$^+$: 404.1868, found: 404.1862.** $[\alpha]^{20}_D$ = -23.25 (c = 10.63, CH_2Cl_2). The product was analyzed by HPLC to determine the enantiomeric excess: 85% e.e. (CHIRALPAK IG, hexane/*i*-PrOH = 80/20, detector: 254 nm, T = 25 °C, flow rate: 1 mL/min), t_1(minor) = 7.10 min, t_2(major) = 7.79 min.

***Tert*-butyl (*R*)-2-(benzo[*d*]oxazol-2-yl)-2-fluoro-4-methylene-6-phenylhexanoate (3s)**

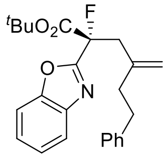

The title compound was prepared according to general procedure A using **1a** (0.1 mmol) and **2s** (0.5 mmol). The crude reaction mixture was purified by flash column chromatography to afford the title compound (petroleum ether/ethyl acetate, 25:1). (75% yield). **^{1}H NMR (400 MHz, $CDCl_3$)** δ 7.84–7.77 (m, 1H), 7.64–7.54 (m, 1H), 7.47–7.35 (m, 2H), 7.34–7.23 (m, 2H), 7.23–7.13 (m, 3H), 5.06 (s, 1H), 5.02 (s, 1H), 3.39–3.22 (m, 2H), 2.90–2.72 (m, 2H), 2.49 (t, *J* = 8.1 Hz, 2H), 1.46 (s, 9H). **^{13}C NMR (101 MHz, $CDCl_3$)** δ 165.15 (d, *J* = 27.6 Hz), 160.86 (d, *J* = 25.0 Hz), 150.94, 141.90, 141.87, 140.47, 128.53, 128.45, 126.36, 125.97, 125.06, 121.08, 116.11, 111.29, 92.36 (d, *J* = 193.7 Hz), 84.60, 40.98 (d, *J* = 20.2 Hz), 38.68 (d, *J* = 2.6 Hz), 34.31, 28.01. **^{19}F NMR (376 MHz, $CDCl_3$)** δ -156.46. **ESI-MS: calculated [$C_{24}H_{26}FNO_3$ + H]$^+$: 396.1969, found: 396.1971.** $[\alpha]^{20}_D$ = -13.80 (c = 11.47, CH_2Cl_2). The product was analyzed by HPLC to determine the enantiomeric excess: 91% e.e. (CHIRALPAK IG, hexane/*i*-PrOH = 99/1, detector: 254 nm, T = 25 °C, flow rate: 1 mL/min), t_1(minor) = 13.75 min, t_2(major) = 15.14 min.

***Tert*-butyl (*R*)-2-(benzo[*d*]oxazol-2-yl)-2-fluoro-3-(1*H*-inden-2-yl)propanoate (3t)**

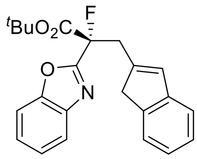

The title compound was prepared according to the general procedure A using **1a** (0.1 mmol) and **2t** (0.5 mmol). The crude reaction mixture was purified by flash column chromatography to afford the title compound (petroleum ether/ethyl acetate, 25:1). (86% yield). **^{1}H NMR (400 MHz, $CDCl_3$)** δ 7.86–7.79 (m, 1H), 7.64–7.57 (m, 1H), 7.42 (ddd, *J* = 6.9, 4.9, 1.7 Hz, 3H), 7.32 (dt, *J* = 7.5, 1.1 Hz, 1H), 7.26–7.21 (m, 1H), 7.16 (td, *J* = 7.4, 1.3 Hz, 1H), 6.80 (s, 1H), 3.87–3.67 (m, 2H), 3.61 (d, *J* = 22.9 Hz, 1H), 3.43 (d, *J* = 22.9 Hz, 1H), 1.41 (s, 9H). **^{13}C NMR (151 MHz, $CDCl_3$)** 165.20 (d, *J* = 27.2 Hz), 160.58 (d, *J* = 25.2 Hz), 151.00, 144.58, 143.88, 140.79, 140.51, 131.99, 126.41, 126.39, 125.09, 124.65, 123.60, 121.12, 120.72, 111.31, 91.94 (d, *J* = 193.1 Hz), 84.71, 42.33, 36.95 (d, *J* = 21.4 Hz), 27.91. **^{19}F NMR (376 MHz, $CDCl_3$)** δ -155.77. **ESI-MS: calculated [$C_{23}H_{22}FNO_3$ + Na]$^+$: 402.1476, found: 402.1472.** $[\alpha]^{20}_D$ = -44.53 (c = 11.87, CH_2Cl_2). The product was analyzed by HPLC to determine the enantiomeric

excess: 94% e.e. (CHIRALPAK IE, hexane/*i*-PrOH = 98/2, detector: 254 nm, T = 25 °C, flow rate: 1 mL/min), t_1(major) = 12.34 min, t_2(minor) = 13.39 min.

***Tert*-butyl (*R*)-2-(benzo[*d*]oxazol-2-yl)-2-fluoro-4-((*R*)-4-methyl-5-oxocyclohex-3-en-1-yl)pent-4-enoate (3u)**

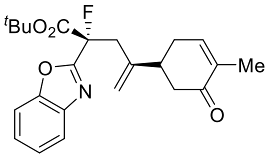

The title compound was prepared according to general procedure A using **1a** (0.1 mmol) and **2u** (0.5 mmol). The crude reaction mixture was purified by flash column chromatography to afford the title compound (petroleum ether/ethyl acetate, 10:1). (60% yield). **^{1}H NMR (500 MHz, $CDCl_3$)** δ 7.81–7.74 (m, 1H), 7.62–7.55 (m, 1H), 7.46–7.34 (m, 2H), 6.78–6.70 (m, 1H), 5.18 (s, 1H), 5.05 (s, 1H), 3.43–3.20 (m, 2H), 2.97–2.87 (m, 1H), 2.71–2.56 (m, 2H), 2.40 (dd, *J* = 16.0, 12.9 Hz, 1H), 2.30–2.14 (m, 1H), 1.78 (s, 3H), 1.46 (s, 9H). **^{13}C NMR (126 MHz, $CDCl_3$)** δ 199.50, 164.91 (d, *J* = 27.0 Hz), 160.62 (d, *J* = 25.3 Hz), 150.94, 144.23, 144.03, 140.39, 135.71, 126.47, 125.13, 121.08, 115.58, 111.29, 92.30 (d, *J* = 193.9 Hz), 84.83, 43.21, 40.78 (d, *J* = 2.7 Hz), 39.44 (d, *J* = 20.1 Hz), 31.79, 28.01, 15.79. **^{19}F NMR (471 MHz, $CDCl_3$)** δ -155.89. **ESI-MS: calculated [$C_{23}H_{26}FNO_4$ + Na]$^+$: 422.1738, found: 422.1747.** $[\alpha]^{20}_{D}$ = -35.83 (c = 5.43, CH_2Cl_2).

***Tert*-butyl (*R*)-2-(benzo[*d*]oxazol-2-yl)-2-fluoro-4-((3*S*,8*R*,9*S*,10*R*,13*S*,14*S*,17*S*)-3-hydroxy-10,13-dimethyl-2,3,4,7,8,9,10,11,12,13,14,15,16,17-tetradecahydro-1*H*-cyclopenta[*a*]phenanthren-17-yl)pent-4-enoate (3v)**

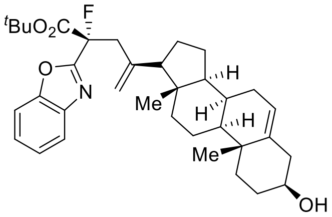

The title compound was prepared according to general procedure A using **1a** (0.1 mmol) and **2v** (0.5 mmol). The crude reaction mixture was purified by flash column chromatography to afford the title compound (petroleum ether/ethyl acetate, 1:1). (70% yield). **^{1}H NMR (400 MHz, $CDCl_3$)** δ 7.84–7.74 (m, 1H), 7.61–7.54 (m, 1H), 7.45–7.33 (m, 2H), 5.38–5.32 (m, 1H), 5.19 (s, 1H), 5.06 (s, 1H), 3.57–3.47 (m, 1H), 3.42–3.22 (m, 2H), 2.41–2.16 (m, 3H), 2.06–1.93 (m, 1H), 1.91–1.72 (m, 5H), 1.71–1.50 (m, 5H), 1.46 (s, 9H), 1.39–1.06 (m, 5H), 1.00 (s, 3H), 0.97–0.89 (m, 1H), 0.61 (s, 3H). **^{13}C NMR (151 MHz, $CDCl_3$)** δ 165.15 (d, *J* = 27.3 Hz), 161.11 (d, *J* = 25.1 Hz), 150.90, 142.04, 140.91, 140.43, 126.31, 124.99, 121.69, 121.04, 115.54, 111.25, 92.78 (d, *J* = 194.1 Hz), 84.42, 71.85, 56.69, 55.64, 50.29, 43.24, 42.61, 42.48, 42.39, 38.86, 37.35, 36.65, 32.49, 31.81 (d, *J* = 19.1 Hz), 28.06, 26.14, 24.18, 21.29, 19.56, 12.93. **^{19}F NMR (376 MHz, $CDCl_3$)** δ -154.86. **ESI-MS: calculated [$C_{35}H_{46}FNO_4$ + H]$^+$: 564.3484, found: 564.3475.** $[\alpha]^{20}_{D}$ = -42.99 (c = 7.27, CH_2Cl_2).

***Tert*-butyl (2*R*)-2-(benzo[*d*]oxazol-2-yl)-2-(cyclopent-2-en-1-yl)-2-fluoroacetate (3w)**

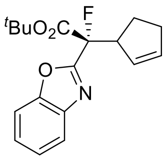

The title compound was prepared according to general procedure A using **1a** (0.1 mmol) and **2w** (0.5 mmol). The crude reaction mixture was purified by flash column chromatography to afford the title compound (petroleum ether/ethyl acetate, 25:1). (70% yield, d.r. = 1:1). **^{1}H NMR (400 MHz, $CDCl_3$)** δ 7.86–7.74 (m, 1H), 7.63–7.51 (m, 1H), 7.45–7.33 (m, 2H), 6.03–5.98 (m, 1H), 5.84–5.78 (m, 1H), 4.10–4.02 (m, 1H), 2.55–2.43 (m, 2H), 2.30–2.19 (m, 1H), 2.09–1.95 (m, 1H), 1.47 (s, 9H). **^{13}C NMR (151 MHz, $CDCl_3$)** δ 165.37 (d, *J* = 27.5 Hz), 160.67 (d, *J* = 28.8 Hz), 150.84, 140.61, 135.45, 127.00 (d, *J* = 2.9 Hz), 126.14, 124.92, 121.02, 111.22, 93.33 (d, *J* = 193.4 Hz), 84.43, 51.28 (d, *J* = 2.9 Hz), 32.27, 27.96, 24.25 (d, *J* = 80.9 Hz). **^{19}F NMR (376 MHz, $CDCl_3$)** δ -168.45. **ESI-MS: calculated [$C_{18}H_{22}FNO_3$ + H]$^+$: 318.1500,**

found: 318.1495. $[\alpha]^{20}_D$ = -4.45 (c = 7.47, CH_2Cl_2). The major product was analyzed by HPLC to determine the enantiomeric excess: 93% e.e. (CHIRALPAK IC, hexane/*i*-PrOH = 98/2, detector: 254 nm, T = 25 °C, flow rate: 1 mL/min), t_1(minor) = 8.55 min, t_2(major) = 12.00 min. The minor product was analyzed by HPLC to determine the enantiomeric excess: 87% e.e. (CHIRALPAK IC, hexane/*i*-PrOH = 98/2, detector: 254 nm, T = 25 °C, flow rate: 1 mL/min), t_1(minor) = 7.61 min, t_2(major) = 9.24 min.

***Tert*-butyl (*S*)-2-(benzo[*d*]oxazol-2-yl)-3-(1*H*-inden-2-yl)-2-methylpropanoate (3x)**

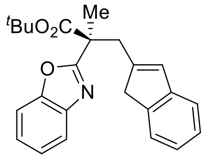

The title compound was prepared according to general procedure A using **1b** (0.1 mmol) and **2t** (1.5 mmol). The crude reaction mixture was purified by flash column chromatography to afford the title compound (petroleum ether/ethyl acetate, 25:1). (77% yield). **^{1}H NMR (600 MHz, $CDCl_3$)** δ 7.79–7.73 (m, 1H), 7.59–7.52 (m, 1H), 7.40–7.33 (m, 2H), 7.30–7.24 (m, 2H), 7.20 (td, *J* = 7.4, 1.1 Hz, 1H), 7.09 (td, *J* = 7.4, 1.2 Hz, 1H), 6.59 (s, 1H), 3.53–3.34 (m, 2H), 3.22 (d, *J* = 22.8 Hz, 1H), 3.03 (d, *J* = 22.8 Hz, 1H), 1.74 (s, 3H), 1.41 (s, 9H). **^{13}C NMR (151 MHz, $CDCl_3$)** δ 171.41, 167.34, 150.92, 144.81, 143.97, 143.52, 141.03, 131.36, 126.33, 125.13, 124.44, 124.26, 123.46, 120.40, 120.27, 110.67, 82.45, 49.88, 41.94, 38.27, 27.91, 21.37. **ESI-MS: calculated [$C_{24}H_{25}NO_3$ + H]$^+$: 376.1907, found: 376.1906.** $[\alpha]^{20}_D$ = +81.44 (c = 10.63, CH_2Cl_2). The product was analyzed by HPLC to determine the enantiomeric excess: 92% e.e. (CHIRALPAK IG, hexane/*i*-PrOH = 95/5, detector: 254 nm, T = 25 °C, flow rate: 1 mL/min), t_1(major) = 5.73 min, t_2(minor) = 6.12 min.

***Tert*-butyl (*S*)-2-(5-fluorobenzo[*d*]oxazol-2-yl)-3-(1*H*-inden-2-yl)-2-methylpropanoate (3y)**

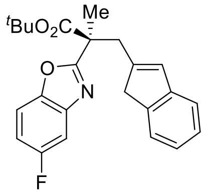

The title compound was prepared according to general procedure A using **1c** (0.1 mmol) and **2t** (1.5 mmol). The crude reaction mixture was purified by flash column chromatography to afford the title compound (petroleum ether/ethyl acetate, 25:1). (57% yield). **^{1}H NMR (400 MHz, $CDCl_3$)** δ 7.67 (dd, *J* = 8.7, 4.9 Hz, 1H), 7.33–7.23 (m, 3H), 7.23–7.17 (m, 1H), 7.16–7.05 (m, 2H), 6.58 (s, 1H), 3.45 (d, *J* = 14.4 Hz, 1H), 3.38 (d, *J* = 14.4 Hz, 1H), 3.21 (d, *J* = 22.6 Hz, 1H), 3.00 (d, *J* = 22.6 Hz, 1H), 1.72 (s, 3H), 1.41 (s, 9H). **^{13}C NMR (151 MHz, $CDCl_3$)** δ 171.28, 167.93 (d, *J* = 3.5 Hz), 160.69 (d, *J* = 244.0 Hz), 150.84 (d, *J* = 14.6 Hz), 144.77, 143.76, 143.48, 137.27, 131.45, 126.38, 124.33, 123.47, 120.56 (d, *J* = 10.1 Hz), 120.44, 112.47 (d, *J* = 24.6 Hz), 98.76 (d, *J* = 28.1 Hz), 82.58, 49.94, 41.95, 38.21, 27.92, 21.35. **^{19}F NMR (376 MHz, $CDCl_3$)** δ -115.26. **ESI-MS: calculated [$C_{24}H_{24}FNO_3$ +H]$^+$: 394.1813, found: 394.1809.** $[\alpha]^{20}_D$ = +79.25 (c = 10.63, CH_2Cl_2). The e.e. value of **3y** is determined by chiral HPLC analysis of derivatives **3y'**.

(*S*)-2-(5-fluorobenzo[*d*]oxazol-2-yl)-3-(1*H*-inden-2-yl)-2-methylpropanal (3y')

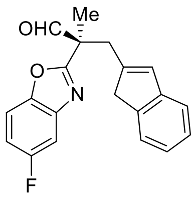

DIBAL-H (1.0 M in toluene, 0.15 mmol) was added dropwise to a solution of **3y** (0.1 mmol) in DCM (1.0 mL) via a syringe at -78 °C. The reaction mixture was stirred at -78 °C for 10 min until the reaction was complete (monitored by TLC). A saturated NH_4Cl solution (5 mL) and HCl (1 M, 1 mL) were added to quench the reaction and the aqueous layer was extracted with DCM (5 mL × 3). The combined organic layers were dried over anhydrous $MgSO_4$, filtered, and concentrated in vacuo. The crude reaction mixture was purified by flash column chromatography to afford the title compound (petroleum ether/ethyl acetate, 20:1). **^{1}H NMR (400 MHz, $CDCl_3$)** δ 9.89 (s, 1H), 7.68–7.60

(m, 1H), 7.27–7.23 (m, 2H), 7.22–7.13 (m, 2H), 7.12–7.02 (m, 2H), 6.51 (s, 1H), 3.37 (d, *J* = 14.6 Hz, 1H), 3.20 (d, *J* = 14.6 Hz, 1H), 3.10 (d, *J* = 22.6 Hz, 1H), 2.98 (d, *J* = 22.5 Hz, 1H), 1.62 (s, 3H). **^{13}C NMR (151 MHz, $CDCl_3$)** δ 198.39, 165.95 (d, *J* = 3.5 Hz), 160.84 (d, *J* = 245.0 Hz), 150.84 (d, *J* = 14.8 Hz), 144.53, 143.30, 142.51, 137.23, 131.76, 126.47, 124.55, 123.52, 120.71 (d, *J* = 10.1 Hz), 120.59, 112.91 (d, *J* = 24.8 Hz), 99.00 (d, *J* = 28.2 Hz), 53.33, 42.04, 36.57, 18.32. **^{19}F NMR (376 MHz, $CDCl_3$)** δ -114.44. **ESI-MS: calculated [$C_{20}H_{16}FNO_2$ + Na]$^+$: 322.1238, found: 322.1247.** $[\alpha]^{20}_D$ = +32.11 (c = 1.47, CH_2Cl_2). The product was analyzed by HPLC to determine the enantiomeric excess: 95% e.e. (CHIRALPAK IG, hexane/*i*-PrOH = 80/20, detector: 254 nm, T = 25 °C, flow rate: 1 mL/min), t_1(major) = 5.50 min, t_2(minor) = 5.83 min.

***Tert*-butyl (*S*)-2-(5-chlorobenzo[*d*]oxazol-2-yl)-3-(1*H*-inden-2-yl)-2-methylpropanoate (3z)**

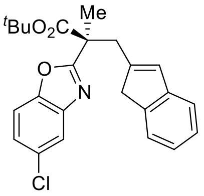

The title compound was prepared according to general procedure A using **1d** (0.1 mmol) and **2t** (1.5 mmol). The crude reaction mixture was purified by flash column chromatography to afford the title compound (petroleum ether/ethyl acetate, 25:1). (80% yield). **^{1}H NMR (600 MHz, $CDCl_3$)** δ 7.72 (d, *J* = 2.1 Hz, 1H), 7.50–7.44 (m, 1H), 7.33 (dd, *J* = 8.6, 2.1 Hz, 1H), 7.30–7.28 (m, 1H), 7.26–7.24 (m, 1H), 7.20 (td, *J* = 7.5, 1.1 Hz, 1H), 7.10 (td, *J* = 7.4, 1.2 Hz, 1H), 6.57 (s, 1H), 3.51–3.32 (m, 2H), 3.20 (d, *J* = 22.7 Hz, 1H), 3.00 (d, *J* = 22.5 Hz, 1H), 1.72 (s, 3H), 1.40 (s, 9H). **^{13}C NMR (151 MHz, $CDCl_3$)** δ 171.18, 168.81, 149.50, 144.74, 143.69, 143.47, 142.18, 131.49, 129.97, 126.40, 125.53, 124.36, 123.50, 120.47, 120.34, 111.43, 82.66, 50.02, 41.94, 38.22, 27.91, 21.32. **ESI-MS: calculated [$C_{24}H_{24}ClNO_3$ + H]$^+$: 410.1517, found: 410.1514.** $[\alpha]^{20}_D$ = +54.29 (c = 13.27, CH_2Cl_2). The product was analyzed by HPLC to determine the enantiomeric excess: 87% e.e. (CHIRALPAK IG, hexane/*i*-PrOH = 98/2, detector: 254 nm, T = 25 °C, flow rate: 0.5 mL/min), t_1(major) = 13.87 min, t_2(minor) = 14.94 min..

***Tert*-butyl (*S*)-2-(5-bromobenzo[*d*]oxazol-2-yl)-3-(1*H*-inden-2-yl)-2-methylpropanoate (3aa)**

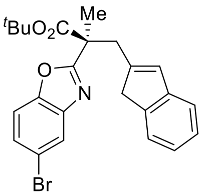

The title compound was prepared according to general procedure A using **1e** (0.1 mmol) and **2t** (1.5 mmol). The crude reaction mixture was purified by flash column chromatography to afford the title compound (petroleum ether/ethyl acetate, 25:1). (77% yield). **^{1}H NMR (600 MHz, $CDCl_3$)** δ 7.89 (s, 1H), 7.51–7.46 (m, 1H), 7.43 (dt, *J* = 8.4, 1.8 Hz, 1H), 7.29 (d, *J* = 7.4 Hz, 1H), 7.27–7.24 (m, 1H), 7.20 (td, *J* = 7.6, 2.5 Hz, 1H), 7.10 (td, *J* = 7.5, 1.9 Hz, 1H), 6.57 (s, 1H), 3.45 (d, *J* = 14.5 Hz, 1H), 3.38 (d, *J* = 14.7 Hz, 1H), 3.20 (d, *J* = 22.5 Hz, 1H), 3.00 (d, *J* = 22.5 Hz, 1H), 1.72 (s, 3H), 1.40 (s, 9H). **^{13}C NMR (151 MHz, $CDCl_3$)** δ 171.15, 168.62, 149.91, 144.72, 143.66, 143.45, 142.65, 131.49, 128.23, 126.39, 124.36, 123.49, 123.34, 120.46, 117.22, 111.93, 82.66, 50.00, 41.94, 38.23, 27.91, 21.33. **ESI-MS: calculated [$C_{24}H_{24}BrNO_3$ + H]$^+$: 454.1012, found: 454.1005.** $[\alpha]^{20}_D$ = +46.71 (c = 11.63, CH_2Cl_2). The product was analyzed by HPLC to determine the enantiomeric excess: 90% e.e. (CHIRALPAK IE, hexane/*i*-PrOH = 98/2, detector: 254 nm, T = 25 °C, flow rate: 1 mL/min), t_1(major) = 6.17 min, t_2(minor) = 6.60 min.

***Tert*-butyl (*S*)-2-(6-chlorobenzo[*d*]oxazol-2-yl)-3-(1*H*-inden-2-yl)-2-methylpropanoate (3ab)**

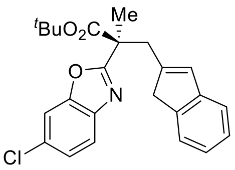

The title compound was prepared according to general procedure A using **1f** (0.1 mmol) and **2t** (1.5 mmol). The crude reaction mixture was purified by flash column chromatography to afford the title compound (petroleum ether/ethyl acetate, 25:1). (80% yield). **^{1}H NMR (600 MHz, $CDCl_3$)** δ 7.65 (d, *J* = 8.4 Hz,

1H), 7.58–7.55 (m, 1H), 7.34 (dt, *J* = 8.6, 1.4 Hz, 1H), 7.29 (d, *J* = 7.4 Hz, 1H), 7.27–7.24 (m, 1H), 7.20 (t, *J* = 7.4 Hz, 1H), 7.09 (t, *J* = 7.4 Hz, 1H), 6.57 (s, 1H), 3.45 (d, *J* = 14.5 Hz, 1H), 3.37 (d, *J* = 14.5 Hz, 1H), 3.20 (d, *J* = 22.6 Hz, 1H), 2.99 (d, *J* = 22.6 Hz, 1H), 1.72 (s, 3H), 1.40 (s, 9H). **^{13}C NMR (151 MHz, $CDCl_3$)** δ 171.19, 168.07, 151.09, 144.74, 143.67, 143.47, 139.79, 131.50, 130.84, 126.39, 125.23, 124.36, 123.49, 120.82, 120.47, 111.37, 82.67, 49.97, 41.94, 38.21, 27.92, 21.35. **ESI-MS: calculated [$C_{24}H_{24}ClNO_3$ + H]$^+$: 410.1517, found: 410.1524.** $[\alpha]^{20}_D$ = +101.40 (c = 10.83, CH_2Cl_2). The product was analyzed by HPLC to determine the enantiomeric excess: 92% e.e. (CHIRALPAK IG, hexane/*i*-PrOH = 99/1, detector: 254 nm, T = 25 °C, flow rate: 1 mL/min), t_1(major) = 11.07 min, t_2(minor) = 12.35 min.

Benzyl (*S*)-2-(benzo[*d*]oxazol-2-yl)-3-(1*H*-inden-2-yl)-2-methylpropanoate (3ac)

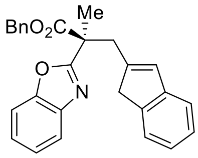

The title compound was prepared according to general procedure A using **1i** (0.1 mmol) and **2t** (1.5 mmol). The crude reaction mixture was purified by flash column chromatography to afford the title compound (petroleum ether/ethyl acetate, 25:1). (48% yield). **^{1}H NMR (600 MHz, $CDCl_3$)** δ 7.80–7.73 (m, 1H), 7.55–7.50 (m, 1H), 7.40–7.35 (m, 2H), 7.31–7.26 (m, 3H), 7.26–7.19 (m, 5H), 7.09 (td, *J* = 7.3, 1.3 Hz, 1H), 6.54 (s, 1H), 5.25–5.17 (m, 2H), 3.49 (s, 2H), 3.11 (d, *J* = 22.8 Hz, 1H), 2.95 (d, *J* = 22.5 Hz, 1H), 1.78 (s, 3H). **^{13}C NMR (151 MHz, $CDCl_3$)** δ 172.14, 166.72, 150.97, 144.71, 143.53, 143.44, 140.98, 135.44, 131.65, 128.63, 128.44, 128.24, 126.35, 125.35, 124.60, 124.35, 123.49, 120.50, 120.38, 110.82, 67.56, 49.32, 41.82, 38.36, 21.34. **ESI-MS: calculated [$C_{27}H_{23}NO_3$ + Na]$^+$: 432.1570, found: 432.1572.** $[\alpha]^{20}_D$ = +37.19 (c = 5.00, CH_2Cl_2). The product was analyzed by HPLC to determine the enantiomeric excess: 86% e.e. (CHIRALPAK IG, hexane/*i*-PrOH = 92/8, detector: 241 nm, T = 25 °C, flow rate: 1 mL/min), t_1(major) = 9.96 min, t_2(minor) = 10.60 min.

Ethyl (*S*)-2-(benzo[*d*]oxazol-2-yl)-3-(1*H*-inden-2-yl)-2-methylpropanoate (3ad)

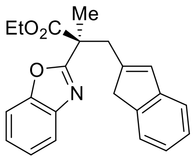

The title compound was prepared according to general procedure A using **1j** (0.1 mmol) and **2t** (1.5 mmol). The crude reaction mixture was purified by flash column chromatography to afford the title compound (petroleum ether/ethyl acetate, 25:1). (71% yield). **^{1}H NMR (600 MHz, $CDCl_3$)** δ 7.80–7.73 (m, 1H), 7.59–7.52 (m, 1H), 7.41–7.33 (m, 2H), 7.31–7.24 (m, 2H), 7.20 (td, *J* = 7.4, 1.1 Hz, 1H), 7.10 (td, *J* = 7.4, 1.2 Hz, 1H), 6.60 (s, 1H), 4.23 (qd, *J* = 7.1, 1.8 Hz, 2H), 3.53–3.44 (m, 2H), 3.20 (d, *J* = 22.7 Hz, 1H), 3.03 (d, *J* = 22.7 Hz, 1H), 1.78 (s, 3H), 1.21 (t, *J* = 7.1 Hz, 3H). **^{13}C NMR (151 MHz, $CDCl_3$)** δ 172.39, 166.85, 150.95, 144.74, 143.59, 143.49, 140.99, 131.55, 126.36, 125.28, 124.55, 124.34, 123.48, 120.47, 120.35, 110.81, 62.08, 49.19, 41.86, 38.38, 21.37, 14.15. **ESI-MS: calculated [$C_{22}H_{21}NO_3$ + H]$^+$: 348.1594, found: 348.1596.** $[\alpha]^{20}_D$ = +76.51 (c = 7.90, CH_2Cl_2). The product was analyzed by HPLC to determine the enantiomeric excess: 90% e.e. (CHIRALPAK IG, hexane/*i*-PrOH = 95/5, detector: 254 nm, T = 25 °C, flow rate: 1 mL/min), t_1(major) = 9.05 min, t_2(minor) = 11.60 min.

2-Phenylpropan-2-yl (*S*)-2-(benzo[*d*]oxazol-2-yl)-3-(1*H*-inden-2-yl)-2-methylpropanoate (3ae)

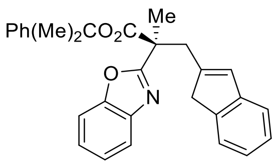

The title compound was prepared according to general procedure A using **1k** (0.1 mmol) and **2t** (1.5 mmol). The crude reaction mixture was purified by flash column chromatography to afford the title compound (petroleum ether/ethyl acetate, 25:1). (59% yield). **^{1}H NMR (600 MHz, $CDCl_3$)** δ 7.83–7.76 (m, 1H), 7.61–7.55 (m, 1H), 7.43–7.35 (m, 2H), 7.27–7.18 (m, 8H),

7.09 (td, J = 7.4, 1.3 Hz, 1H), 6.56 (s, 1H), 3.49 (d, J = 14.7 Hz, 1H), 3.40 (d, J = 14.5 Hz, 1H), 3.16 (d, J = 22.7 Hz, 1H), 2.98 (d, J = 22.7 Hz, 1H), 1.78 (s, 3H), 1.73 (s, 3H), 1.72 (s, 3H). **^{13}C NMR (151 MHz, $CDCl_3$)** δ 170.61, 167.09, 150.96, 145.05, 144.80, 143.81, 143.53, 141.07, 131.45, 128.34, 127.36, 126.35, 125.31, 124.59, 124.35, 124.29, 123.49, 120.45, 120.37, 110.72, 83.54, 49.73, 41.97, 38.10, 28.43, 28.15, 21.15. **ESI-MS: calculated [$C_{29}H_{27}NO_3$ + Na]$^+$: 460.1883, found: 460.1880.** $[\alpha]^{20}_D$ = +77.29 (c = 8.13, CH_2Cl_2). The product was analyzed by HPLC to determine the enantiomeric excess: 88% e.e. (CHIRALPAK IG, hexane/*i*-PrOH = 98/2, detector: 254 nm, T = 25 °C, flow rate: 0.5 mL/min), t_1(major) = 21.97 min, t_2(minor) = 23.71 min.

(*S*)-2-(benzo[*d*]oxazol-2-yl)-3-(1*H*-inden-2-yl)-N,2-dimethylpropanamide (3af)

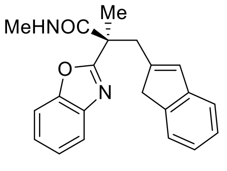

The title compound was prepared according to general procedure A using **1l** (0.1 mmol) and **2t** (1.5 mmol). The crude reaction mixture was purified by flash column chromatography to afford the title compound (petroleum ether/ethyl acetate, 10:1). (56% yield). **^{1}H NMR (600 MHz, $CDCl_3$)** δ 8.17 (q, J = 4.7 Hz, 1H), 7.73–7.67 (m, 1H), 7.61–7.55 (m, 1H), 7.43–7.34 (m, 2H), 7.25 (d, J = 8.1 Hz, 1H), 7.20–7.13 (m, 2H), 7.06 (td, J = 7.2, 1.7 Hz, 1H), 6.42 (s, 1H), 3.54 (d, J = 14.3 Hz, 1H), 3.33 (d, J = 14.2 Hz, 1H), 3.11–3.00 (m, 2H), 2.89 (d, J = 4.6 Hz, 3H), 1.78 (s, 3H). **^{13}C NMR (151 MHz, $CDCl_3$)** δ 171.73, 168.20, 150.35, 144.88, 144.47, 143.43, 140.19, 130.51, 126.25, 125.57, 124.84, 124.19, 123.44, 120.45, 120.02, 110.93, 49.59, 41.68, 40.58, 26.89, 24.39. **ESI-MS: calculated [$C_{21}H_{20}N_2O_2$ + H]$^+$: 333.1598, found: 333.1599.** $[\alpha]^{20}_D$ = -41.42 (c = 6.13, CH_2Cl_2). The product was analyzed by HPLC to determine the enantiomeric excess: 85% e.e. (CHIRALPAK IG, hexane/*i*-PrOH = 70/30, detector: 254 nm, T = 25 °C, flow rate: 1 mL/min), t_1(major) = 6.23 min, t_2(minor) = 7.00 min.

***Tert*-butyl (*S*)-2-(benzo[*d*]oxazol-2-yl)-3-(cyclohex-1-en-1-yl)-2-methylpropanoate (3ag)**

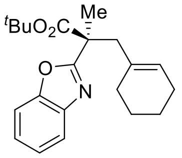

The title compound was prepared according to general procedure A using **1b** (0.1 mmol) and **2q** (2 mmol). The crude reaction mixture was purified by flash column chromatography to afford the title compound (petroleum ether/ethyl acetate, 25:1). (59% yield). **^{1}H NMR (600 MHz, $CDCl_3$)** δ 7.75–7.69 (m, 1H), 7.54–7.48 (m, 1H), 7.35–7.29 (m, 2H), 5.45 (s, 1H), 2.93 (d, J = 13.7 Hz, 1H), 2.83 (d, J = 13.8 Hz, 1H), 1.95–1.92 (m, 2H), 1.78–1.70 (m, 1H), 1.65 (s, 3H), 1.58–1.50 (m, 1H), 1.50–1.42 (m, 4H), 1.38 (s, 9H). **^{13}C NMR (151 MHz, $CDCl_3$)** δ 171.89, 168.03, 150.85, 141.09, 132.98, 127.11, 124.93, 124.27, 120.15, 110.59, 82.08, 49.44, 45.04, 29.25, 27.93, 25.54, 23.00, 22.14, 21.18. **ESI-MS: calculated [$C_{21}H_{27}NO_3$ + H]$^+$: 342.2064, found: 342.2063.** $[\alpha]^{20}_D$ = -50.89 (c = 3.63, CH_2Cl_2). The product was analyzed by HPLC to determine the enantiomeric excess: 91% e.e. (CHIRALPAK IE, hexane/*i*-PrOH = 98/2, detector: 254 nm, T = 25 °C, flow rate: 1 mL/min), t_1(minor) = 5.86 min, t_2(major) = 6.30 min.

***Tert*-butyl (*S*)-2-(benzo[*d*]oxazol-2-yl)-2-methyl-4-methylene-6-phenylhexanoate (3ah)**

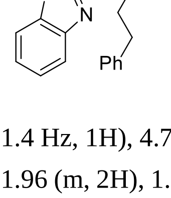

The title compound was prepared according to general procedure A using **1b** (0.1 mmol) and **2s** (2 mmol). The crude reaction mixture was purified by flash column chromatography to afford the title compound (petroleum ether/ethyl acetate, 25:1). (79% yield). **^{1}H NMR (500 MHz, $CDCl_3$)** δ 7.78–7.71 (m, 1H), 7.54–7.48 (m, 1H), 7.39–7.29 (m, 2H), 7.21–7.15 (m, 2H), 7.14–7.10 (m, 1H), 7.00–6.94 (m, 2H), 4.88 (q, J = 1.4 Hz, 1H), 4.76 (s, 1H), 3.09 (d, J = 14.1 Hz, 1H), 2.95 (d, J = 14.2 Hz, 1H), 2.74–2.60 (m, 2H), 2.13–1.96 (m, 2H), 1.72 (s, 3H), 1.38 (s, 9H). **^{13}C NMR (151 MHz, $CDCl_3$)** δ 171.62, 167.60, 150.86, 144.00,

141.84, 141.02, 128.34, 128.31, 125.82, 125.08, 124.41, 120.23, 115.31, 110.65, 82.34, 49.36, 42.35, 38.63, 34.47, 27.91, 21.19. **ESI-MS: calculated [$C_{25}H_{29}NO_3$ + H]$^+$: 392.2220, found: 392.2214.** $[\alpha]^{20}_D$ = -40.15 (c = 10.30, CH_2Cl_2). The product was analyzed by HPLC to determine the enantiomeric excess: 89% e.e. (CHIRALPAK IG, hexane/*i*-PrOH = 98/2, detector: 254 nm, T = 25 °C, flow rate: 0.5 mL/min), t_1(minor) = 10.83 min, t_2(major) = 11.71 min.

***Tert*-butyl (*S*)-2-((1*H*-inden-2-yl)methyl)-2-(benzo[*d*]oxazol-2-yl)butanoate (3ai)**

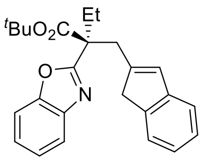

The title compound was prepared according to general procedure A using **1h** (0.1 mmol) and **2t** (1.5 mmol). The crude reaction mixture was purified by flash column chromatography to afford the title compound (petroleum ether/ethyl acetate, 25:1). (75% yield). **^{1}H NMR (600 MHz, $CDCl_3$)** δ 7.79–7.73 (m, 1H), 7.57–7.51 (m, 1H), 7.39–7.33 (m, 2H), 7.28–7.22 (m, 2H), 7.19 (td, *J* = 7.4, 1.1 Hz, 1H), 7.08 (td, *J* = 7.4, 1.2 Hz, 1H), 6.57 (s, 1H), 3.50 – 3.41 (m, 2H), 3.16 (d, *J* = 22.6 Hz, 1H), 3.01 (d, *J* = 22.6 Hz, 1H), 2.26 (m, 2H), 1.39 (s, 9H), 0.97 (t, *J* = 7.5 Hz, 3H). **^{13}C NMR (151 MHz, $CDCl_3$)** δ 170.64, 166.89, 150.81, 144.84, 144.04, 143.50, 140.94, 131.00, 126.31, 125.11, 124.41, 124.22, 123.44, 120.36, 120.30, 110.66, 82.38, 54.01, 41.78, 34.96, 27.98, 26.90, 8.77. **ESI-MS: calculated [$C_{25}H_{27}NO_3$ + Na]$^+$: 412.1883, found: 412.1880.** $[\alpha]^{20}_D$ = + 55.27 (c = 8.53, CH_2Cl_2). The product was analyzed by HPLC to determine the enantiomeric excess: 87% e.e. (CHIRALPAK IG, hexane/*i*-PrOH = 98/2, detector: 254 nm, T = 25 °C, flow rate: 1 mL/min), t_1(major) = 7.30 min, t_2(minor) = 9.50 min.

***Tert*-butyl (*S*)-2-((1*H*-inden-2-yl)methyl)-2-(benzo[*d*]oxazol-2-yl)-4-phenylbutanoate (3aj)**

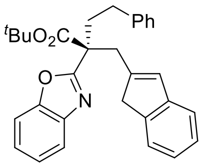

The title compound was prepared according to general procedure A using **1g** (0.1 mmol) and **2t** (1.5 mmol). The crude reaction mixture was purified by flash column chromatography to afford the title compound (petroleum ether/ethyl acetate, 25:1). (64% yield). **^{1}H NMR (400 MHz, $CDCl_3$)** δ 7.73–7.63 (m, 1H), 7.51–7.42 (m, 1H), 7.33–7.24 (m, 2H), 7.19–7.11 (m, 5H), 7.10–7.05 (m, 3H), 7.00 (td, *J* = 7.4, 1.3 Hz, 1H), 6.55 (s, 1H), 3.47 (s, 2H), 3.09 (d, *J* = 23.0 Hz, 1H), 2.92 (d, *J* = 22.6 Hz, 1H), 2.70–2.34 (m, 4H), 1.34 (s, 9H). **^{13}C NMR (101 MHz, $CDCl_3$)** δ 170.47, 166.54, 150.78, 144.71, 143.78, 143.49, 141.32, 140.91, 131.24, 128.51, 128.50, 126.33, 126.14, 125.21, 124.49, 124.30, 123.47, 120.44, 120.35, 110.70, 82.66, 53.52, 41.70, 35.91, 35.81, 30.84, 28.01. **ESI-MS: calculated [$C_{31}H_{31}NO_3$ + H]$^+$: 466.2377, found: 466.2381.** $[\alpha]^{20}_D$ = +27.94 (c = 11.23, CH_2Cl_2). The product was analyzed by HPLC to determine the enantiomeric excess: 88% e.e. (CHIRALPAK IG, hexane/*i*-PrOH = 98/2, detector: 254 nm, T = 25 °C, flow rate: 1 mL/min), t_1(minor) = 8.49 min, t_2(major) = 11.72 min.

***Tert*-butyl (*S*)-2-(benzo[*d*]isoxazol-3-yl)-3-(1*H*-inden-2-yl)-2-methylpropanoate (3ak)**

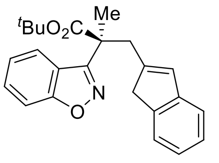

The title compound was prepared according to general procedure A using **1m** (0.1 mmol) and **2t** (1.5 mmol). The crude reaction mixture was purified by flash column chromatography to afford the title compound (petroleum ether/ethyl acetate, 25:1). (69% yield). **^{1}H NMR (600 MHz, $CDCl_3$)** δ 7.73 (dt, *J* = 8.0, 1.0 Hz, 1H), 7.64–7.58 (m, 1H), 7.60–7.50 (m, 1H), 7.33–7.23 (m, 3H), 7.20 (td, *J* = 7.4, 1.1 Hz, 1H), 7.09 (td, *J* = 7.4, 1.2 Hz, 1H), 6.58 (s, 1H), 3.55 (d, *J* = 14.3 Hz, 1H), 3.40 (d, *J* = 14.7 Hz, 1H), 3.27–3.04 (m, 2H), 1.75 (s, 3H), 1.32 (s, 9H). **^{13}C NMR (126 MHz, $CDCl_3$)** δ 172.60, 163.62, 160.27, 144.87, 144.47, 143.65, 131.37, 129.77, 126.33, 124.25, 123.47, 123.37, 122.47, 120.41, 120.39, 110.30, 82.34, 48.64, 42.39, 38.39, 27.88, 22.16. **ESI-MS: calculated [$C_{24}H_{25}NO_3$ + H]$^+$: 376.1907, found: 376.1898.**

$[\alpha]^{20}_{D}$ = -25.73 (c = 8.47, CH_2Cl_2). The product was analyzed by HPLC to determine the enantiomeric excess: 88% e.e. (CHIRALPAK IG, hexane/*i*-PrOH = 98/2, detector: 254 nm, T = 25 °C, flow rate: 1 mL/min), t_1(minor) = 9.10 min, t_2(major) = 9.83 min.

***Tert*-butyl (*R*,*E*)-2-(benzo[*d*]oxazol-2-yl)-2-fluorooct-3-enoate (4a)**

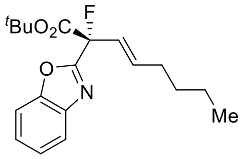

The title compound was prepared according to general procedure B using **1a** (0.1 mmol) and **2a** (0.5 mmol). The crude reaction mixture was purified by flash column chromatography to afford the title compound (petroleum ether/ethyl acetate, 25:1). (72% yield). **^{1}H NMR (400 MHz, $CDCl_3$)** δ 7.85–7.75 (m, 1H), 7.63–7.53 (m, 1H), 7.46–7.33 (m, 2H), 6.27–6.10 (m, 2H), 2.31–2.16 (m, 2H), 1.47 (s, 9H), 1.48–1.37 (m, 2H), 1.41–1.28 (m, 2H), 0.91 (t, *J* = 7.2 Hz, 3H). **^{13}C NMR (151 MHz, $CDCl_3$)** δ 165.07 (d, *J* = 28.9 Hz), 160.76 (d, *J* = 25.5 Hz), 150.99, 140.53, 136.65 (d, *J* = 8.9 Hz), 126.33, 124.98, 122.36 (d, *J* = 18.9 Hz), 121.16, 111.24, 90.16 (d, *J* = 190.5 Hz), 84.63, 32.06, 30.79, 27.94, 22.25, 13.99. **^{19}F NMR (376 MHz, $CDCl_3$)** δ -152.52. **ESI-MS: calculated [$C_{19}H_{24}FNO_3$ + Na]$^+$: 356.1632, found: 356.1640.** $[\alpha]^{20}_{D}$ = -22.41 (c = 7.43, CH_2Cl_2). The product was analyzed by HPLC to determine the enantiomeric excess: 92% e.e. (CHIRALPAK IG, hexane/*i*-PrOH = 98/2, detector: 254 nm, T = 25 °C, flow rate: 1 mL/min), t_1(minor) = 6.99 min, t_2(major) = 8.63 min.

***Tert*-butyl (*R*,*E*)-2-(benzo[d]oxazol-2-yl)-8-chloro-2-fluorooct-3-enoate (4b)**

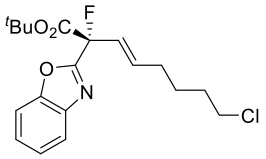

The title compound was prepared according to general procedure B using **1a** (0.1 mmol) and **2b** (0.5 mmol). The crude reaction mixture was purified by flash column chromatography to afford the title compound (petroleum ether/ethyl acetate, 25:1). (66% yield). **^{1}H NMR (500 MHz, $CDCl_3$)** δ 7.83–7.77 (m, 1H), 7.62–7.55 (m, 1H), 7.45–7.35 (m, 2H), 6.28–6.14 (m, 2H), 3.55 (t, *J* = 6.6 Hz, 2H), 2.33–2.23 (m, 2H), 1.89–1.78 (m, 2H), 1.70–1.59 (m, 2H), 1.48 (s, 9H). **^{13}C NMR (126 MHz, $CDCl_3$)** δ 164.94 (d, *J* = 28.6 Hz), 160.60 (d, *J* = 25.7 Hz), 151.00, 140.52, 135.47 (d, *J* = 9.2 Hz), 126.39, 125.02, 123.20 (d, *J* = 18.9 Hz), 121.17, 111.26, 90.09 (d, *J* = 191.2 Hz), 84.79, 44.85, 32.05, 31.56, 27.97, 25.94. **^{19}F NMR (471 MHz, $CDCl_3$)** δ -153.09. **ESI-MS: calculated [$C_{19}H_{23}ClFNO_3$ + Na]$^+$: 390.1243, found: 390.1244.** $[\alpha]^{20}_{D}$ = -19.74 (c = 7.77, CH_2Cl_2). The product was analyzed by HPLC to determine the enantiomeric excess: 91% e.e. (CHIRALPAK IG, hexane/*i*-PrOH = 95/5, detector: 254 nm, T = 25 °C, flow rate: 1 mL/min), t_1(minor) = 8.10 min, t_2(major) = 9.90 min.

***Tert*-butyl (*R*,*E*)-2-(benzo[d]oxazol-2-yl)-8-bromo-2-fluorooct-3-enoate (4c)**

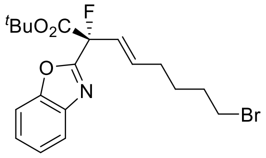

The title compound was prepared according to general procedure B using **1a** (0.1 mmol) and **2c** (0.5 mmol). The crude reaction mixture was purified by flash column chromatography to afford the title compound (petroleum ether/ethyl acetate, 25:1). (62% yield). **^{1}H NMR (500 MHz, $CDCl_3$)** δ 7.84–7.77 (m, 1H), 7.61–7.55 (m, 1H), 7.45–7.35 (m, 2H), 6.26–6.14 (m, 2H), 3.42 (t, *J* = 6.7 Hz, 2H), 2.33–2.21 (m, 2H), 1.96 – 1.86 (m, 2H), 1.68–1.54 (m, 2H), 1.48 (s, 9H). **^{13}C NMR (126 MHz, $CDCl_3$)** δ 164.93 (d, *J* = 28.7 Hz), 160.60 (d, *J* = 25.6 Hz), 151.00, 140.53, 135.40 (d, *J* = 9.2 Hz), 126.39, 125.02, 123.25 (d, *J* = 19.0 Hz), 121.18, 111.26, 90.09 (d, *J* = 191.1 Hz), 84.80, 33.49, 32.22, 31.45, 27.98, 27.22. **^{19}F NMR (471 MHz, $CDCl_3$)** δ -153.11. **ESI-MS: calculated [$C_{19}H_{23}BrFNO_3$ + Na]$^+$: 434.0738, found: 434.0743.** $[\alpha]^{20}_{D}$ = -19.79 (c = 8.57, CH_2Cl_2). The product

was analyzed by HPLC to determine the enantiomeric excess: 91% e.e. (CHIRALPAK IG, hexane/*i*-PrOH = 95/5, detector: 254 nm, T = 25 °C, flow rate: 1 mL/min), t_1(minor) = 8.46 min, t_2(major) = 10.35 min.

***Tert*-butyl (*R*,*E*)-2-(benzo[*d*]oxazol-2-yl)-2-fluoro-8-hydroxyoct-3-enoate (4d)**

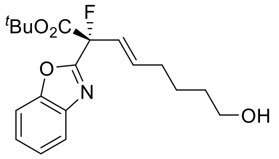

The title compound was prepared according to general procedure B using **1a** (0.1 mmol) and **2d** (0.5 mmol). The crude reaction mixture was purified by flash column chromatography to afford the title compound (petroleum ether/ethyl acetate, 1:1). (76% yield). **^{1}H NMR (500 MHz, $CDCl_3$)** δ 7.82–7.75 (m, 1H), 7.60–7.53 (m, 1H), 7.44–7.34 (m, 2H), 6.26–6.13 (m, 2H), 3.67 (t, *J* = 6.2 Hz, 2H), 2.31–2.23 (m, 2H), 1.66–1.53 (m, 4H), 1.47 (s, 9H). **^{13}C NMR (126 MHz, $CDCl_3$)** δ 164.99 (d, *J* = 28.8 Hz), 160.71 (d, *J* = 25.6 Hz), 151.00, 140.50, 136.06 (d, *J* = 9.2 Hz), 126.37, 125.01, 122.85 (d, *J* = 19.0 Hz), 121.15, 111.24, 90.14 (d, *J* = 190.9 Hz), 84.73, 62.71, 32.24, 32.04, 27.96, 24.84. **^{19}F NMR (471 MHz, $CDCl_3$)** δ -152.79. **ESI-MS: calculated [$C_{19}H_{24}FNO_4$ + Na]$^+$: 372.1582, found: 372.1588.** $[\alpha]^{20}_D$ = -20.90 (c = 6.83, CH_2Cl_2). The product was analyzed by HPLC to determine the enantiomeric excess: 92% e.e. (CHIRALPAK IG, hexane/*i*-PrOH = 90/10, detector: 254 nm, T = 25 °C, flow rate: 1 mL/min), t_1(minor) = 16.18 min, t_2(major) = 17.26 min.

***Tert*-butyl (*R*,*E*)-2-(benzo[*d*]oxazol-2-yl)-2-fluoro-8-(tosyloxy)oct-3-enoate (4e)**

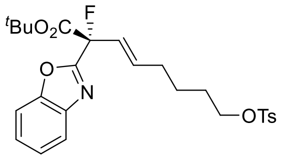

The title compound was prepared according to general procedure B using **1a** (0.1 mmol) and **2e** (0.5 mmol). The crude reaction mixture was purified by flash column chromatography to afford the title compound (petroleum ether/ethyl acetate, 15:1). (62% yield). **^{1}H NMR (500 MHz, $CDCl_3$)** δ 7.83–7.76 (m, 3H), 7.60–7.55 (m, 1H), 7.44–7.36 (m, 2H), 7.34 (d, *J* = 8.1 Hz, 2H), 6.20–6.09 (m, 2H), 4.04 (t, *J* = 6.4 Hz, 2H), 2.44 (s, 3H), 2.22–2.14 (m, 2H), 1.73–1.65 (m, 2H), 1.54–1.48 (m, 2H), 1.47 (s, 9H). **^{13}C NMR (151 MHz, $CDCl_3$)** δ 164.88 (d, *J* = 28.8 Hz), 160.52 (d, *J* = 25.4 Hz), 150.97, 144.92, 140.46, 135.14 (d, *J* = 9.3 Hz), 133.15, 130.00, 128.02, 126.42, 125.04, 123.22 (d, *J* = 18.9 Hz), 121.15, 111.26, 90.02 (d, *J* = 191.2 Hz), 84.83, 70.29, 31.55, 28.31, 27.94, 24.51, 21.76. **^{19}F NMR (471 MHz, $CDCl_3$)** δ -153.17. **ESI-MS: calculated [$C_{26}H_{30}FNO_6S$ + Na]$^+$: 526.1670, found: 526.1661.** $[\alpha]^{20}_D$ = -12.98 (c = 9.17, CH_2Cl_2). The product was analyzed by HPLC to determine the enantiomeric excess: 91% e.e. (CHIRALPAK IG, hexane/*i*-PrOH = 70/30, detector: 254 nm, T = 25 °C, flow rate: 1 mL/min), t_1(minor) = 20.09 min, t_2(major) = 30.14 min.

***Tert*-butyl (*R*,*E*)-2-(benzo[*d*]oxazol-2-yl)-8-((*tert*-butyldimethylsilyl)oxy)-2-fluorooct-3-enoate (4f)**

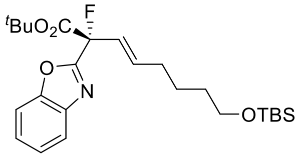

The title compound was prepared according to general procedure B using **1a** (0.1 mmol) and **2f** (0.5 mmol). The crude reaction mixture was purified by flash column chromatography to afford the title compound (petroleum ether/ethyl acetate, 25:1). (58% yield). **^{1}H NMR (600 MHz, $CDCl_3$)** δ 7.82–7.77 (m, 1H), 7.60–7.55 (m, 1H), 7.44–7.35 (m, 2H), 6.25–6.13 (m, 2H), 3.62 (t, *J* = 6.1 Hz, 2H), 2.29–2.20 (m, 2H), 1.59–1.51 (m, 4H), 1.47 (s, 9H), 0.88 (s, 9H), 0.04 (s, 6H). **^{13}C NMR (151 MHz, $CDCl_3$)** δ 165.03 (d, *J* = 28.9 Hz), 160.71 (d, *J* = 25.6 Hz), 150.99, 140.54, 136.36 (d, *J* = 9.0 Hz), 126.33, 124.97, 122.61 (d, *J* = 19.1 Hz), 121.15, 111.24, 90.15 (d, *J* = 190.7 Hz), 84.65, 63.02, 32.36, 32.16, 27.95, 26.10, 25.06, 18.49, -5.15. **^{19}F NMR (376 MHz, $CDCl_3$)** δ -152.71. **ESI-MS: calculated [$C_{25}H_{38}FNO_4Si$ + Na]$^+$: 486.2446, found: 486.2446.** $[\alpha]^{20}_D$ = -18.46 (c = 6.53,

CH_2Cl_2). The product was analyzed by HPLC to determine the enantiomeric excess: 90% e.e. (CHIRALPAK IG, hexane/*i*-PrOH = 98/2, detector: 243 nm, T = 25 °C, flow rate: 1 mL/min), t_1(minor) = 4.94 min, t_2(major) = 5.65 min.

***Tert*-butyl (*R*,*E*)-2-(benzo[*d*]oxazol-2-yl)-2-fluoro-8-phenoxyoct-3-enoate (4g)**

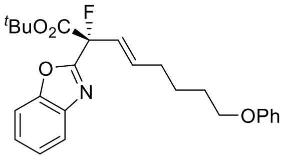

The title compound was prepared according to general procedure B using **1a** (0.1 mmol) and **2g** (0.5 mmol). The crude reaction mixture was purified by flash column chromatography to afford the title compound (petroleum ether/ethyl acetate, 25:1). (60% yield). **^{1}H NMR (400 MHz, $CDCl_3$)** δ 7.84–7.75 (m, 1H), 7.63–7.53 (m, 1H), 7.46–7.34 (m, 2H), 7.32–7.23 (m, 2H), 6.97–6.86 (m, 3H), 6.31–6.15 (m, 2H), 3.98 (t, *J* = 6.3 Hz, 2H), 2.37–2.27 (m, 2H), 1.90–1.79 (m, 2H), 1.74–1.61 (m, 2H), 1.48 (s, 9H). **^{13}C NMR (151 MHz, $CDCl_3$)** δ 164.99 (d, *J* = 28.8 Hz), 160.65 (d, *J* = 25.4 Hz), 159.11, 150.98, 140.50, 135.93 (d, *J* = 9.0 Hz), 129.56, 126.37, 125.00, 122.89 (d, *J* = 18.9 Hz), 121.16, 120.67, 114.56, 111.26, 90.12 (d, *J* = 190.8 Hz), 84.75, 67.54, 32.07, 28.85, 27.94, 25.26. **^{19}F NMR (376 MHz, $CDCl_3$)** δ -152.75. **ESI-MS: calculated $[C_{25}H_{28}FNO_4 + Na]^+$: 448.1895, found: 448.1894.** $[\alpha]^{20}_{D}$ = -19.54 (c = 7.90, CH_2Cl_2). The product was analyzed by HPLC to determine the enantiomeric excess: 90% e.e. (CHIRALPAK IG, hexane/*i*-PrOH = 95/5, detector: 254 nm, T = 25 °C, flow rate: 1 mL/min), t_1(minor) = 10.66 min, t_2(major) = 13.96 min.

(*R*,*E*)-5-(benzo[d]oxazol-2-yl)-6-(*tert*-butoxy)-5-fluoro-6-oxohex-3-en-1-yl thiophene-2-carboxylate (4h)

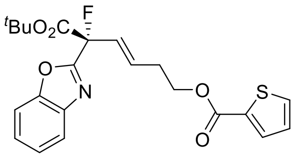

The title compound was prepared according to general procedure B using **1a** (0.1 mmol) and **2h** (0.5 mmol). The crude reaction mixture was purified by flash column chromatography to afford the title compound (petroleum ether/ethyl acetate, 25:1). (58% yield). **^{1}H NMR (400 MHz, $CDCl_3$)** δ 7.82–7.75 (m, 2H), 7.60–7.48 (m, 2H), 7.45–7.33 (m, 2H), 7.06 (dd, *J* = 4.9, 3.8 Hz, 1H), 6.40–6.21 (m, 2H), 4.42 (t, *J* = 6.5 Hz, 2H), 2.74–2.64 (m, 2H), 1.45 (s, 9H). **^{13}C NMR (151 MHz, $CDCl_3$)** δ 164.68 (d, *J* = 28.2 Hz), 162.22, 160.39 (d, *J* = 25.5 Hz), 151.00, 140.52, 133.78, 133.66, 132.57, 131.05 (d, *J* = 9.9 Hz), 127.85, 126.42, 125.38 (d, *J* = 19.2 Hz), 125.04, 121.17, 111.27, 90.04 (d, *J* = 192.0 Hz), 84.93, 63.58, 31.80, 27.92. **^{19}F NMR (376 MHz, $CDCl_3$)** δ -154.61. **ESI-MS: calculated $[C_{22}H_{22}FNO_5S + Na]^+$: 454.1095, found: 454.1089.** $[\alpha]^{20}_{D}$ =-16.61 (c = 5.70, CH_2Cl_2). The product was analyzed by HPLC to determine the enantiomeric excess: 90% e.e. (CHIRALPAK IG, hexane/*i*-PrOH = 80/20, detector: 254 nm, T = 25 °C, flow rate: 1 mL/min), t_1(minor) = 11.22 min, t_2(major) = 12.81 min.

(*R*,*E*)-5-(benzo[*d*]oxazol-2-yl)-6-(*tert*-butoxy)-5-fluoro-6-oxohex-3-en-1-yl furan-2-carboxylate (4i)

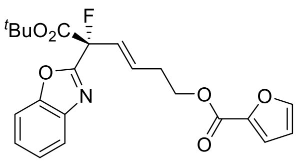

The title compound was prepared according to general procedure B using **1a** (0.1 mmol) and **2i** (0.5 mmol). The crude reaction mixture was purified by flash column chromatography to afford the title compound (petroleum ether/ethyl acetate, 25:1). (54% yield). **^{1}H NMR (400 MHz, $CDCl_3$)** δ 7.82–7.75 (m, 1H), 7.60–7.53 (m, 2H), 7.46–7.34 (m, 2H), 7.19 (dd, *J* = 3.5, 0.9 Hz, 1H), 6.48 (dd, *J* = 3.5, 1.8 Hz, 1H), 6.38 6.22 (m, 2H), 4.43 (t, *J* = 6.6 Hz, 2H), 2.74–2.64 (m, 2H), 1.46 (s, 9H). **^{13}C NMR (151 MHz, $CDCl_3$)** δ 164.68 (d, *J* = 28.6 Hz), 160.36 (d, *J* = 25.7 Hz), 158.70, 151.00, 146.52, 144.66, 140.50, 130.92 (d, *J* = 9.9 Hz), 126.44, 125.41 (d, *J* = 19.0 Hz), 125.06,

121.16, 118.24, 111.95, 111.27, 90.01 (d, J = 192.2 Hz), 84.94, 63.38, 31.74, 27.91. **^{19}F NMR (376 MHz, CDCl$_3$)** δ -154.61. **ESI-MS: calculated [$C_{22}H_{22}FNO_6$ + Na]$^+$: 438.1323, found: 438.1327.** $[\alpha]^{20}_D$ = -16.79 (c = 4.63, CH_2Cl_2). The product was analyzed by HPLC to determine the enantiomeric excess: 90% e.e. (CHIRALPAK IG, hexane/*i*-PrOH = 80/20, detector: 254 nm, T = 25 °C, flow rate: 1 mL/min), t_1(minor) = 12.03 min, t_2(major) = 14.30 min.

(*R*,*E*)-5-(benzo[*d*]oxazol-2-yl)-6-(*tert*-butoxy)-5-fluoro-6-oxohex-3-en-1-yl cyclopropanecarboxylate (4j)

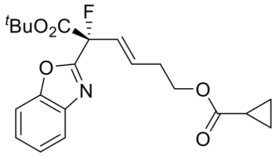

The title compound was prepared according to general procedure B using **1a** (0.1 mmol) and **2j** (0.5 mmol). The crude reaction mixture was purified by flash column chromatography to afford the title compound (petroleum ether/ethyl acetate, 25:1). (53% yield). **^{1}H NMR (400 MHz, CDCl$_3$)** δ 7.83–7.74 (m, 1H), 7.61–7.53 (m, 1H), 7.46–7.34 (m, 2H), 6.35–6.15 (m, 2H), δ 4.19 (t, J = 6.6 Hz, 2H), 2.62–2.53 (m, 2H), 1.64–1.53 (m, 1H), 1.48 (s, 9H), 1.05–0.94 (m, 2H), 0.89–0.77 (m, 2H). **^{13}C NMR (151 MHz, CDCl$_3$)** δ 174.92, 164.73 (d, J = 28.4 Hz), 160.39 (d, J = 25.4 Hz), 150.99, 140.48, 131.44 (d, J = 9.5 Hz), 126.45, 125.07, 125.02 (d, J = 18.9 Hz), 121.16, 111.26, 90.00 (d, J = 191.6 Hz), 84.92, 63.02, 31.73, 27.94, 13.00, 8.60. **^{19}F NMR (376 MHz, CDCl$_3$)** δ -154.29. **ESI-MS: calculated [$C_{21}H_{24}FNO_5$ + Na]$^+$: 412.1531, found: 412.1536.** $[\alpha]^{20}_D$ = -18.54 (c = 4.07, CH_2Cl_2). The product was analyzed by HPLC to determine the enantiomeric excess: 90% e.e. (CHIRALPAK IG, hexane/*i*-PrOH = 80/20, detector: 254 nm, T = 25 °C, flow rate: 1 mL/min), t_1(minor) = 7.82 min, t_2(major) = 8.47 min.

***Tert*-butyl (*R*,*E*)-2-(benzo[*d*]oxazol-2-yl)-2-fluoro-7-(4-(4,4,5,5-tetramethyl-1,3,2-dioxaborolan-2-yl)phenoxy)hept-3-enoate (4k)**

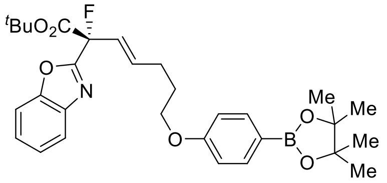

The title compound was prepared according to general procedure B using **1a** (0.1 mmol) and **2x** (0.5 mmol). The crude reaction mixture was purified by flash column chromatography to afford the title compound (petroleum ether/ethyl acetate, 10:1). (61% yield). **^{1}H NMR (400 MHz, CDCl$_3$)** δ 7.82–7.78 (m, 1H), 7.73 (d, J = 8.5 Hz, 2H), 7.59–7.54 (m, 1H), 7.45–7.34 (m, 2H), 6.89 (d, J = 8.5 Hz, 2H), 6.32–6.16 (m, 2H), 4.03 (t, J = 6.3 Hz, 2H), 2.48–2.38 (m, 2H), 2.03–1.92 (m, 2H), 1.45 (s, 9H), 1.33 (s, 12H). **^{13}C NMR (101 MHz, CDCl$_3$)** δ 164.90 (d, J = 28.7 Hz), 161.66, 160.57 (d, J = 25.5 Hz), 150.97, 140.50, 136.64, 135.05 (d, J = 9.3 Hz), 126.38, 125.01, 123.34 (d, J = 19.0 Hz), 121.18, 113.96, 111.26, 90.09 (d, J = 190.9 Hz), 84.80, 83.66, 66.74, 28.83, 28.30, 27.94, 24.99. **^{19}F NMR (376 MHz, CDCl$_3$)** δ -153.55. **ESI-MS: calculated [$C_{30}H_{37}BFNO_6$ + Na]$^+$: 560.2590, found: 560.2586.** $[\alpha]^{20}_D$ = -15.67 (c = 10.40, CH_2Cl_2). The product was analyzed by HPLC to determine the enantiomeric excess: 91% e.e. (CHIRALPAK IG, hexane/*i*-PrOH = 80/20, detector: 254 nm, T = 25 °C, flow rate: 1 mL/min), t_1(minor) = 6.21 min, t_2(major) = 11.12 min.

***Tert*-butyl (*R*,*E*)-2-(benzo[*d*]oxazol-2-yl)-7-(1,3-dioxoisoindolin-2-yl)-2-fluorohept-3-enoate (4l)**

The title compound was prepared according to general procedure B using **1a** (0.1 mmol) and **2k** (0.5 mmol). The crude reaction mixture was purified by flash column chromatography to afford the title

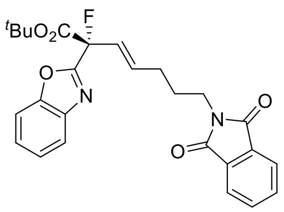

compound (petroleum ether/ethyl acetate, 4:1). (60% yield). **^{1}H NMR (400 MHz, CDCl$_3$)** δ 7.87–7.82 (m, 2H), 7.81–7.77 (m, 1H), 7.75–7.66 (m, 2H), 7.59–7.54 (m, 1H), 7.45–7.32 (m, 2H), 6.30–6.15 (m, 2H), 3.74 (t, *J* = 7.1 Hz, 2H), 2.35–2.25 (m, 2H), 1.93–1.81 (m, 2H), 1.47 (s, 9H). **^{13}C NMR (151 MHz, CDCl$_3$)** δ 168.50, 164.85 (d, *J* = 28.7 Hz), 160.56 (d, *J* = 25.3 Hz), 150.98, 140.52, 134.60 (d, *J* = 9.3 Hz), 134.08, 132.24, 126.36, 124.99, 123.42 (d, *J* = 18.7 Hz), 123.38, 121.18, 111.25, 90.05 (d, *J* = 191.0 Hz), 84.82, 37.58, 29.76, 27.95, 27.70. **^{19}F NMR (376 MHz, CDCl$_3$)** δ -153.31. **ESI-MS: calculated [$C_{26}H_{25}FN_2O_5$ + Na]$^+$: 487.1640, found: 487.1647.** $[\alpha]^{20}_{D}$ = -13.93 (c = 6.97, CH_2Cl_2). The product was analyzed by HPLC to determine the enantiomeric excess: 91% e.e. (CHIRALPAK IC, hexane/*i*-PrOH = 70/30, detector: 254 nm, T = 25 °C, flow rate: 1 mL/min), t_1(major) = 12.23 min, t_2(minor) = 16.02 min.

***Tert*-butyl (*R*,*E*)-2-(benzo[*d*]oxazol-2-yl)-2-fluoro-6-phenylhex-3-enoate (4m)**

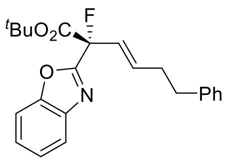

The title compound was prepared according to general procedure B using **1a** (0.1 mmol) and **2m** (0.5 mmol). The crude reaction mixture was purified by flash column chromatography to afford the title compound (petroleum ether/ethyl acetate, 25:1). (74% yield). **^{1}H NMR (500 MHz, CDCl$_3$)** δ 7.80–7.75 (m, 1H), 7.57–7.52 (m, 1H), 7.42–7.32 (m, 2H), 7.28–7.21 (m, 2H), 7.19–7.12 (m, 3H), 6.32–6.10 (m, 2H), 2.83–2.70 (m, 2H), 2.57–2.49 (m, 2H), 1.42 (s, 9H). **^{13}C NMR (151 MHz, CDCl$_3$)** δ 164.88 (d, *J* = 28.4 Hz), 160.64 (d, *J* = 25.8 Hz), 150.97, 141.22, 140.50, 135.39 (d, *J* = 9.3 Hz), 128.57, 128.53, 126.36, 126.14, 125.00, 123.09 (d, *J* = 19.3 Hz), 121.14, 111.24, 90.12 (d, *J* = 190.8 Hz), 84.75, 35.07, 34.05, 27.93. **^{19}F NMR (471 MHz, CDCl$_3$)** δ -152.95. **ESI-MS: calculated [$C_{23}H_{24}FNO_3$ + Na]$^+$: 404.1632, found: 404.1634.** $[\alpha]^{20}_{D}$ = -26.57 (c = 7.73, CH_2Cl_2). The product was analyzed by HPLC to determine the enantiomeric excess: 90% e.e. (CHIRALPAK IG, hexane/*i*-PrOH = 98/2, detector: 254 nm, T = 25 °C, flow rate: 1 mL/min), t_1(minor) = 10.97 min, t_2(major) = 13.25 min.

***Tert*-butyl (*R*,*E*)-2-(benzo[*d*]oxazol-2-yl)-2-fluoro-5-phenylpent-3-enoate (4n)**

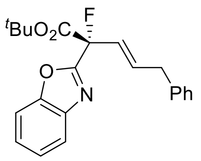

The title compound was prepared according to general procedure B using **1a** (0.1 mmol) and **2n** (0.5 mmol). The crude reaction mixture was purified by flash column chromatography to afford the title compound (petroleum ether/ethyl acetate, 25:1). (64% yield). **^{1}H NMR (500 MHz, CDCl$_3$)** δ 7.84–7.78 (m, 1H), 7.61–7.55 (m, 1H), 7.45–7.35 (m, 2H), 7.34–7.28 (m, 2H), 7.26–7.19 (m, 3H), 6.43–6.33 (m, 1H), 6.32–6.22 (m, 1H), 3.57 (dd, *J* = 6.7, 2.3 Hz, 2H), 1.47 (s, 9H). **^{13}C NMR (126 MHz, CDCl$_3$)** δ 164.85 (d, *J* = 28.8 Hz), 160.48 (d, *J* = 25.4 Hz), 150.99, 140.51, 138.90, 134.71 (d, *J* = 8.9 Hz), 128.76, 128.69, 126.58, 126.41, 125.03, 124.02 (d, *J* = 19.3 Hz), 121.18, 111.28, 90.10 (d, *J* = 191.3 Hz), 84.83, 38.57, 27.93. **^{19}F NMR (471 MHz, CDCl$_3$)** δ -153.05. **ESI-MS: calculated [$C_{22}H_{22}FNO_3$ + Na]$^+$: 390.1476, found: 390.1476.** $[\alpha]^{20}_{D}$ = -19.33 (c = 5.70, CH_2Cl_2). The product was analyzed by HPLC to determine the enantiomeric excess: 90% e.e. (CHIRALPAK IG, hexane/*i*-PrOH = 98/2, detector: 254 nm, T = 25 °C, flow rate: 1 mL/min), t_1(minor) = 11.45 min, t_2(major) = 14.68 min.

***Tert*-butyl (*R*,*E*)-2-(benzo[*d*]oxazol-2-yl)-2-fluorotetradec-3-enoate (4o)**

The title compound was prepared according to general procedure B using **1a** (0.1 mmol) and **2y** (0.5 mmol). The crude reaction mixture was purified by flash column chromatography to afford the title

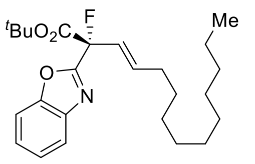

compound (petroleum ether/ethyl acetate, 25:1). (65% yield). **^{1}H NMR (500 MHz, $CDCl_3$)** δ 7.83–7.77 (m, 1H), 7.60–7.55 (m, 1H), 7.44–7.34 (m, 2H), 6.26–6.12 (m, 2H), 2.26–2.17 (m, 2H), 1.48 (s, 9H), 1.53–1.45 (m, 1H), 1.37–1.19 (m, 15H), 0.88 (t, *J* = 6.9 Hz, 3H). **^{13}C NMR (151 MHz, $CDCl_3$)** δ 165.08 (d, *J* = 28.9 Hz), 160.76 (d, *J* = 25.8 Hz), 150.98, 140.50, 136.71 (d, *J* = 9.3 Hz), 126.34, 124.99, 122.29 (d, *J* = 18.9 Hz), 121.15, 111.25, 90.16 (d, *J* = 190.6 Hz), 84.66, 32.39, 32.03, 29.74, 29.57, 29.47, 29.21, 28.69, 27.94, 22.82, 14.27. **^{19}F NMR (471 MHz, $CDCl_3$)** δ -152.35. **ESI-MS: calculated [$C_{25}H_{36}FNO_3$ + H]$^+$: 418.2752, found: 418.2757.** $[\alpha]^{20}_D$ = -22.19 (c = 4.73, CH_2Cl_2). The product was analyzed by HPLC to determine the enantiomeric excess: 91% e.e. (CHIRALPAK IG, hexane/*i*-PrOH = 99/1, detector: 243 nm, T = 25 °C, flow rate: 0.7 mL/min), t_1(minor) = 9.01 min, t_2(major) = 9.99 min.

***Tert*-butyl (*S*,*E*)-2-(benzo[*d*]isoxazol-3-yl)-2-methyloct-3-enoate (4p)**

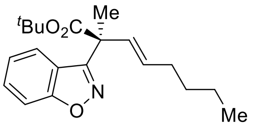

The title compound was prepared according to general procedure B using **1m** (0.1 mmol) and **2a** (2 mmol). The crude reaction mixture was purified by flash column chromatography to afford the title compound (petroleum ether/ethyl acetate, 25:1). (64% yield). **^{1}H NMR (600 MHz, $CDCl_3$)** δ 7.60–7.53 (m, 2H), 7.53–7.47 (m, 1H), 7.27–7.22 (m, 1H), 6.13 (dt, *J* = 15.8, 1.4 Hz, 1H), 5.48 (dt, *J* = 15.8, 6.9 Hz, 1H), 2.07 (qd, *J* = 7.0, 1.4 Hz, 2H), 1.82 (s, 3H), 1.34 (s, 9H), 1.30–1.22 (m, 4H), 0.86 (t, *J* = 7.2 Hz, 3H). **^{13}C NMR (151 MHz, $CDCl_3$)** δ 171.88, 163.44, 160.22, 132.29, 129.96, 129.51, 123.10, 122.87, 120.74, 110.08, 82.12, 50.14, 32.35, 31.31, 27.86, 23.07, 22.20, 13.99. **ESI-MS: calculated [$C_{20}H_{27}NO_3$ + Na]$^+$: 352.1883, found: 352.1882.** $[\alpha]^{20}_D$ = +8.33 (c = 7.43, CH_2Cl_2). The product was analyzed by HPLC to determine the enantiomeric excess: 92% e.e. (CHIRALPAK IC, hexane/*i*-PrOH = 98/2, detector: 230 nm, T = 25 °C, flow rate: 1 mL/min), t_1(minor) = 5.30 min, t_2(major) = 5.86 min.

***Tert*-butyl (*R*)-2-(benzo[*d*]oxazol-2-yl)-2-fluorooctanoate (5a)**

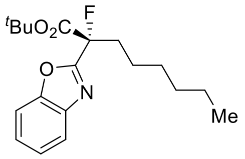

The title compound was prepared according to general procedure C using **1a** (0.1 mmol) and **2a** (2 mmol). The crude reaction mixture was purified by flash column chromatography to afford the title compound (petroleum ether/ethyl acetate, 25:1). (60% yield). **^{1}H NMR (400 MHz, $CDCl_3$)** δ 7.83–7.74 (m, 1H), 7.61–7.54 (m, 1H), 7.45–7.34 (m, 2H), 2.59–2.37 (m, 2H), 1.70–1.59 (m, 1H), 1.48 (s, 9H), 1.42 (m, 3H), 1.32 (m, 4H), 0.91 – 0.87 (m, 3H). **^{13}C NMR (151 MHz, $CDCl_3$)** δ 165.85 (d, *J* = 27.7 Hz), 161.03 (d, *J* = 25.4 Hz), 150.88, 140.48, 126.25, 124.97, 121.03, 111.25, 92.29 (d, *J* = 189.8 Hz), 84.39, 35.13 (d, *J* = 21.7 Hz), 31.57, 29.12, 27.98, 22.74 (d, *J* = 2.6 Hz), 22.62, 14.18. **^{19}F NMR (376 MHz, $CDCl_3$)** δ -159.35. **ESI-MS: calculated [$C_{19}H_{26}FNO_3$ + Na]$^+$: 358.1789, found: 358.1797.** $[\alpha]^{20}_D$ = -14.85 (c = 5.83, CH_2Cl_2). The product was analyzed by HPLC to determine the enantiomeric excess: 93% e.e. (CHIRALPAK IG, hexane/*i*-PrOH = 98/2, detector: 254 nm, T = 25 °C, flow rate: 1 mL/min), t_1(minor) = 6.02 min, t_2(major) = 6.77 min.

***Tert*-butyl (*R*)-2-(benzo[*d*]oxazol-2-yl)-8-((*tert*-butyldimethylsilyl)oxy)-2-fluorooctanoate (5b)**

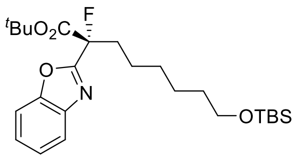

The title compound was prepared according to general procedure C using **1a** (0.1 mmol) and **2f** (2 mmol). The crude reaction mixture was purified by flash column chromatography to afford the title compound (petroleum ether/ethyl acetate, 25:1). (68% yield). **^{1}H NMR (500 MHz, $CDCl_3$)** δ

7.81–7.76 (m, 1H), 7.61–7.53 (m, 1H), 7.44–7.34 (m, 2H), 3.60 (t, *J* = 6.5 Hz, 2H), 2.58–2.40 (m, 2H), 1.71–1.59 (m, 1H), 1.57–1.50 (m, 2H), 1.48 (s, 9H), 1.46–1.34 (m, 5H), 0.89 (s, 9H), 0.04 (s, 6H). **^{13}C NMR (126 MHz, $CDCl_3$)** δ 165.84 (d, *J* = 27.6 Hz), 161.04 (d, *J* = 25.7 Hz), 150.92, 140.55, 126.24, 124.96, 121.04, 111.24, 92.29 (d, *J* = 189.8 Hz), 84.37, 63.25, 35.13 (d, *J* = 21.6 Hz), 32.79, 29.29, 27.99, 26.12, 25.65, 22.86 (d, *J* = 2.7 Hz), 18.50, -5.13. **^{19}F NMR (471 MHz, $CDCl_3$)** δ -159.31. **ESI-MS: calculated [$C_{25}H_{40}FNO_4Si$ + Na]$^+$: 488.2603, found: 488.2614.** $[\alpha]^{20}_D$ = -10.81 (c = 9.50, CH_2Cl_2). The product was analyzed by HPLC to determine the enantiomeric excess: 92% e.e. (CHIRALPAK IG, hexane/*i*-PrOH = 98/2, detector: 254 nm, T = 25 °C, flow rate: 0.5 mL/min), t_1(minor) = 9.27 min, t_2(major) = 10.07 min.

***Tert*-butyl (*R*)-2-(benzo[*d*]oxazol-2-yl)-2-fluoro-8-phenoxyoctanoate (5c)**

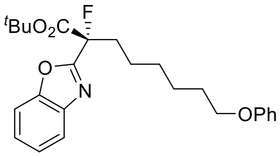

The title compound was prepared according to general procedure C using **1a** (0.1 mmol) and **2g** (2 mmol). The crude reaction mixture was purified by flash column chromatography to afford the title compound (petroleum ether/ethyl acetate, 25:1). (65% yield). **^{1}H NMR (500 MHz, $CDCl_3$)** δ 7.82–7.77 (m, 1H), 7.60–7.55 (m, 1H), 7.45–7.37 (m, 2H), 7.31–7.24 (m, 2H), 6.96–6.87 (m, 3H), 3.96 (t, *J* = 6.4 Hz, 2H), 2.61–2.43 (m, 2H), 1.80 (p, *J* = 6.5 Hz, 2H), 1.75–1.66 (m, 1H), 1.62–1.50 (m, 5H), 1.49 (s, 9H). **^{13}C NMR (126 MHz, $CDCl_3$)** δ 165.82 (d, *J* = 27.6 Hz), 160.96 (d, *J* = 25.7 Hz), 159.17, 150.90, 140.50, 129.54, 126.27, 124.99, 121.04, 120.62, 114.58, 111.25, 92.25 (d, *J* = 190.1 Hz), 84.45, 67.77, 35.06 (d, *J* = 21.6 Hz), 29.26, 29.24, 27.99, 25.95, 22.80 (d, *J* = 2.5 Hz).**^{19}F NMR (471 MHz, $CDCl_3$)** δ -159.34. **ESI-MS: calculated [$C_{25}H_{30}FNO_4$ + Na]$^+$: 450.2051, found: 450.2055.** $[\alpha]^{20}_D$ = -9.76 (c = 6.30, CH_2Cl_2). The product was analyzed by HPLC to determine the enantiomeric excess: 92% e.e. (CHIRALPAK IG, hexane/*i*-PrOH = 95/5, detector: 254 nm, T = 25 °C, flow rate: 1 mL/min), t_1(minor) = 9.70 min, t_2(major) = 11.06 min.

***Tert*-butyl (*R*)-5-acetoxy-2-(benzo[*d*]oxazol-2-yl)-2-fluoropentanoate (5d)**

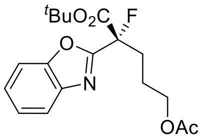

The title compound was prepared according to general procedure C using **1a** (0.1 mmol) and **2ab** (2 mmol). The crude reaction mixture was purified by flash column chromatography to afford the title compound (petroleum ether/ethyl acetate, 25:1). (60% yield).**^{1}H NMR (400 MHz, $CDCl_3$)** δ 7.86–7.71 (m, 1H), 7.63–7.53 (m, 1H), 7.46–7.34 (m, 2H), 4.24–4.09 (m, 2H), 2.71–2.46 (m, 2H), 2.06 (s, 3H), 2.03–1.94 (m, 1H), 1.90–1.75 (m, 1H), 1.49 (s, 9H). **^{13}C NMR (151 MHz, $CDCl_3$)** δ 171.16, 165.49 (d, *J* = 27.5 Hz), 160.51 (d, *J* = 25.3 Hz), 150.89, 140.40, 126.41, 125.08, 121.05, 111.28, 91.83 (d, *J* = 190.7 Hz), 84.77, 63.76, 31.93 (d, *J* = 21.7 Hz), 27.96, 22.49 (d, *J* = 3.1 Hz), 21.08. **^{19}F NMR (376 MHz, $CDCl_3$)** δ -159.34. **ESI-MS: calculated [$C_{18}H_{22}FNO_5$ + Na]$^+$: 374.1374, found: 374.1383.** $[\alpha]^{20}_D$ = -15.76 (c = 7.40, CH_2Cl_2). The product was analyzed by HPLC to determine the enantiomeric excess: 90% e.e. (CHIRALPAK IG, hexane/*i*-PrOH = 95/5, detector: 254 nm, T = 25 °C, flow rate: 1 mL/min), t_1(minor) = 10.78 min, t_2(major) = 11.70 min.

***Tert*-butyl (*R*)-2-(benzo[*d*]oxazol-2-yl)-2-fluoro-6-phenylhexanoate (5e)**

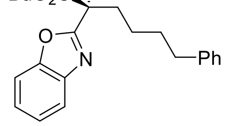

The title compound was prepared according to general procedure C using **1a** (0.1 mmol) and **2m** (2 mmol). The crude reaction mixture was purified by flash column chromatography to afford the title compound (petroleum ether/ethyl acetate, 25:1). (75% yield). **^{1}H NMR (500 MHz, $CDCl_3$)** δ 7.84–7.77 (m, 1H),

7.62–7.54 (m, 1H), 7.45–7.35 (m, 2H), 7.31–7.24 (m, 2H), 7.21–7.14 (m, 3H), 2.72–2.63 (m, 2H), 2.61–2.46 (m, 2H), 1.85–1.66 (m, 3H), 1.56–1.49 (m, 1H), 1.45 (s, 9H). **^{13}C NMR (151 MHz, $CDCl_3$)** δ 165.76 (d, *J* = 27.5 Hz), 160.96 (d, *J* = 25.3 Hz), 150.92, 142.16, 140.52, 128.54, 128.47, 126.28, 125.92, 124.99, 121.04, 111.26, 92.22 (d, *J* = 190.1 Hz), 84.45, 35.63, 35.01 (d, *J* = 21.6 Hz), 31.19, 27.93, 22.45 (d, *J* = 2.7 Hz). **^{19}F NMR (471 MHz, $CDCl_3$)** δ -159.05. **ESI-MS: calculated [$C_{23}H_{26}FNO_3$ + Na]$^+$: 406.1789, found: 406.1794.** $[\alpha]^{20}_D$ = -12.98 (c = 8.33, CH_2Cl_2). The product was analyzed by HPLC to determine the enantiomeric excess: 91% e.e. (CHIRALPAK IG, hexane/*i*-PrOH = 98/2, detector: 271 nm, T = 25 °C, flow rate: 1 mL/min), t_1(minor) = 9.72 min, t_2(major) = 11.58 min.

***Tert*-butyl (*R*)-2-(benzo[d]oxazol-2-yl)-2-cyclopentyl-2-fluoroacetate (5f)**

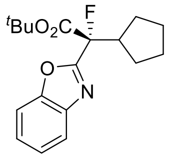

The title compound was prepared according to general procedure C using **1a** (0.1 mmol) and **2w** (2 mmol). The crude reaction mixture was purified by flash column chromatography to afford the title compound (petroleum ether/ethyl acetate, 25:1). (76% yield). **^{1}H NMR (500 MHz, $CDCl_3$)** δ 7.83–7.76 (m, 1H), 7.61–7.52 (m, 1H), 7.43–7.33 (m, 2H), 3.26–3.09 (m, 1H), 2.02–1.90 (m, 1H), 1.87–1.78 (m, 1H), 1.78–1.56 (m, 6H), 1.47 (s, 9H). **^{13}C NMR (126 MHz, $CDCl_3$)** δ 165.81 (d, *J* = 27.7 Hz), 161.17 (d, *J* = 26.4 Hz), 150.87, 140.70, 126.03, 124.88, 120.99, 111.19, 93.83 (d, *J* = 193.5 Hz), 84.26, 44.34 (d, *J* = 21.5 Hz), 27.98, 26.98 (d, *J* = 3.4 Hz), 26.46 (d, *J* = 2.6 Hz), 26.08, 26.04. **^{19}F NMR (471 MHz, $CDCl_3$)** δ -172.87. **ESI-MS: calculated [$C1_8H_{22}FNO_3$ + Na]$^+$: 342.1476, found: 342.1480.** $[\alpha]^{20}_D$ = -7.67 (c = 7.07, CH_2Cl_2). The product was analyzed by HPLC to determine the enantiomeric excess: 94% e.e. (CHIRALPAK IG, hexane/*i*-PrOH = 98/2, detector: 240 nm, T = 25 °C, flow rate: 1 mL/min), t_1(minor) = 7.45 min, t_2(major) = 8.73 min.

***Tert*-butyl (*R*)-2-(benzo[*d*]oxazol-2-yl)-2-cyclohexyl-2-fluoroacetate (5g)**

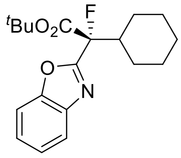

The title compound was prepared according to general procedure C using **1a** (0.1 mmol) and **2z** (2 mmol). The crude reaction mixture was purified by flash column chromatography to afford the title compound (petroleum ether/ethyl acetate, 25:1). (56% yield). **^{1}H NMR (500 MHz, $CDCl_3$)** δ 7.83–7.75 (m, 1H), 7.62–7.54 (m, 1H), 7.43–7.33 (m, 2H), 2.71–2.56 (m, 1H), 1.87–1.66 (m, 5H), 1.47 (s, 9H), 1.43–1.30 (m, 4H), 1.21–1.14 (m, 1H). **^{13}C NMR (126 MHz, $CDCl_3$)** δ 165.51 (d, *J* = 26.9 Hz), 160.68 (d, *J* = 26.8 Hz), 150.83, 140.71, 125.90, 124.88, 120.91, 111.18, 95.26 (d, *J* = 194.9 Hz), 84.38, 43.42 (d, *J* = 20.6 Hz), 28.02, 26.45 (d, *J* = 2.2 Hz), 26.30 (d, *J* = 3.2 Hz), 26.23, 26.04, 26.01. **^{19}F NMR (471 MHz, $CDCl_3$)** δ -172.39. **ESI-MS: calculated [$C_{19}H_{24}FNO_3$ + Na]$^+$: 356.1636, found: 356.1636.** $[\alpha]^{20}_D$ = -7.36 (c = 4.20, CH_2Cl_2). The product was analyzed by HPLC to determine the enantiomeric excess: 85% e.e. (CHIRALPAK IG, hexane/*i*-PrOH = 98/2, detector: 240 nm, T = 25 °C, flow rate: 1 mL/min), t_1(minor) = 7.55 min, t_2(major) = 9.70 min.

***Tert*-butyl (*R*)-2-(benzo[*d*]oxazol-2-yl)-3-(2,3-dihydro-1*H*-inden-2-yl)-2-fluoropropanoate (5h)**

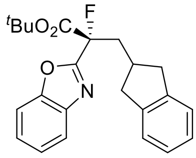

The title compound was prepared according to general procedure C using **1a** (0.1 mmol) and **2t** (2 mmol). The crude reaction mixture was purified by flash column chromatography to afford the title compound (petroleum ether/ethyl acetate, 25:1). (79% yield). **^{1}H NMR (400 MHz, $CDCl_3$)** δ 7.84–7.76 (m, 1H), 7.65–7.56 (m, 1H), 7.47–7.36 (m, 2H), 7.22–7.18 (m, 2H), 7.17–7.12 (m, 2H), 3.25–3.09 (m, 2H), 2.91–2.69 (m, 5H), 1.50 (s, 9H). **^{13}C NMR (101 MHz, $CDCl_3$)** δ 165.82 (d, *J* = 27.7 Hz), 160.99 (d, *J* = 25.5

Hz), 150.89, 143.28, 142.76, 140.42, 126.43, 126.36, 125.06, 124.45, 124.31, 121.03, 111.26, 92.48 (d, *J* = 191.4 Hz), 84.69, 40.12 (d, *J* = 20.6 Hz), 39.94, 39.86 (d, *J* = 2.2 Hz), 36.32, 28.00. **^{19}F NMR (376 MHz, CDCl$_3$)** δ -158.74. **ESI-MS: calculated [$C_{23}H_{24}FNO_3$ + Na]$^+$: 404.1632, found: 404.1634.** $[\alpha]^{20}_D$ = -19.94 (c = 8.80, CH_2Cl_2). The product was analyzed by HPLC to determine the enantiomeric excess: 93% e.e. (CHIRALPAK IG, hexane/*i*-PrOH = 95/5, detector: 254 nm, T = 25 °C, flow rate: 1 mL/min), t_1(minor) = 8.79 min, t_2(major) = 9.64 min.

***Tert*-butyl (*S*)-2-(benzo[*d*]oxazol-2-yl)-3-(2,3-dihydro-1*H*-inden-2-yl)-2-methylpropanoate (5i)**

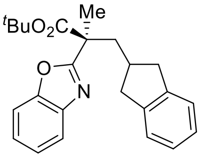

The title compound was prepared according to general procedure C using **1b** (0.1 mmol) and **2t** (2 mmol). The crude reaction mixture was purified by flash column chromatography to afford the title compound (petroleum ether/ethyl acetate, 25:1). (66% yield). **^{1}H NMR (500 MHz, CDCl$_3$)** δ 7.78–7.71 (m, 1H), 7.55 7.48 (m, 1H), 7.39–7.30 (m, 2H), 7.17–7.11 (m, 1H), 7.11–7.04 (m, 3H), 3.04 (dd, *J* = 15.3, 6.4 Hz, 1H), 2.86–2.78 (m, 1H), 2.72–2.64 (m, 1H), 2.60–2.43 (m, 4H), 1.80 (s, 3H), 1.41 (s, 9H). **^{13}C NMR (126 MHz, CDCl$_3$)** δ 171.89, 167.85, 150.92, 143.27, 143.15, 141.08, 126.27, 125.06, 124.38, 124.28, 124.25, 120.21, 110.63, 82.24, 49.78, 42.09, 40.57, 39.96, 37.33, 27.97, 21.58. **ESI-MS: calculated [$C_{24}H_{27}NO_3$ + Na]$^+$: 400.1883, found: 400.1887.** $[\alpha]^{20}_D$ = -15.79 (c = 6.13, CH_2Cl_2). The product was analyzed by HPLC to determine the enantiomeric excess: 98% e.e. (CHIRALPAK IE, hexane/*i*-PrOH = 98/2, detector: 254 nm, T = 25 °C, flow rate: 1 mL/min), t_1(major) = 7.13 min, t_2(minor) = 8.48 min.

6. Synthetic applications

6.1 Ni/Co catalyzed electrocatalytic dehydrogenative allylation with propylene gas

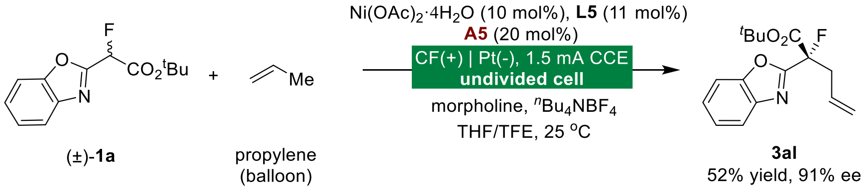

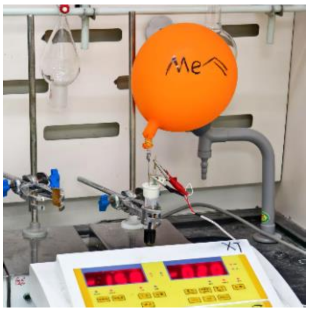

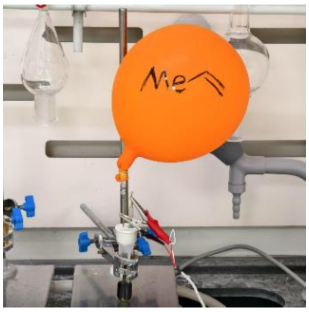

A 10 mL flask equipped with carbon felt (1.5 cm × 1.5 cm × 3 mm) as the anode and platinum plate (1.0 cm × 1.0 cm × 0.2 mm) as the cathode was degassed via vacuum evacuation and the flask was injected with propylene through a balloon. A solution of **1a** (0.1 mmol), nickel complex (0.01 mmol), **A5** (0.02 mmol), morpholine (0.1 mmol) and $^nBu_4NBF_4$ in THF (0.5 mL) and TFE (2.5 mL) were subsequently added. Constant current (1.5 mA) electrolysis was carried out at 25 °C for 24 h (a new balloon containing propylene needs to be replaced every 4 h) until complete consumption of the starting material (monitored by TLC). The solvent was removed under reduced pressure. The residue was purified by silica gel chromatography to afford desired product **3al**.

6.2 Scale-up experiment

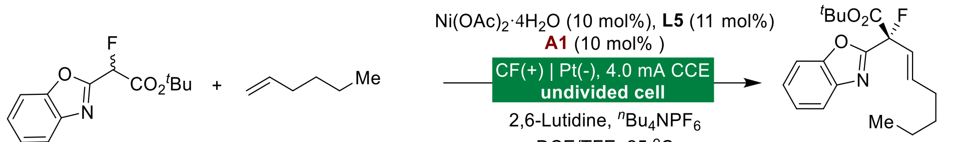

(±)-**1a** **2a** **4a**, 1.26 g
62% yield, 92% e.e.

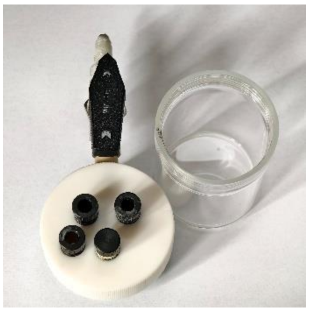
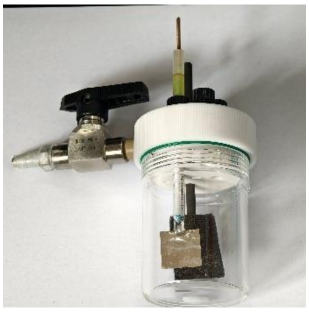
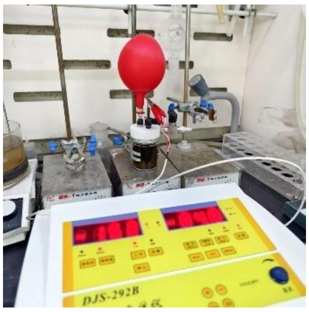

A 100 mL undivided electrochemical cell equipped with a magnetic stir bar was charged with **1a** (6 mmol), nickel complex (0.6 mmol), Cp_2Fe-CH_2NMe_2 **A1** (0.6 mmol), and $^{n}Bu_4NPF_6$ (9 mmol). The flask was equipped with a carbon felt (2.5 cm × 3.5 cm × 3 mm) as the anode and platinum plate (2.0 cm × 2.0 cm × 0.2 mm) as the cathode. The reaction mixture was degassed via vacuum evacuation and backfilled with argon three times. A solution of **2a** (30 mmol) and 2,6-Lutidine (6 mmol) in DCE (15 mL) and TFE (75 mL) was subsequently added under argon. Constant current (4.0 mA) electrolysis was carried out at 25 °C for 3 d until complete consumption of the starting material (monitored by TLC). The solvent was removed under reduced pressure. The residue was purified by silica gel chromatography to afford desired product **4a**.

6.3 Functionalization of enantioenriched products 3a and 3x

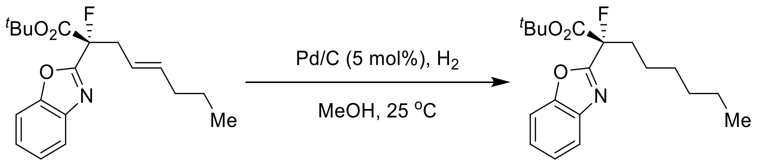

3a **6**

The reaction flask with a hydrogen balloon was charged with **3a** (0.1 mmol), Pd/C (palladium on activated carbon, 10%; wetted with ca. 55% water) (0.005 mmol, 50 wt%), and dry MeOH (1.0 mL). After stirring at 25 °C for 1 h, the starting material was consumed completely (monitored by TLC). Pd/C was removed through a short silica gel column and concentrated in vacuo, and the residue was purified by silica gel chromatography to afford the desired product **6** (petroleum ether/ethyl acetate, 25:1).

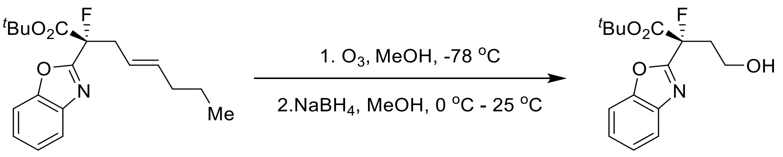

3a **7**

A solution of **3a** (0.2 mmol) in dry MeOH (2 mL) was cooled to -78 °C. Ozone was bubbled through

the solution until a blue color persisted, followed by the addition of oxygen gas to remove the excess of ozone. PPh_3 (0.3 mmol) was added, and the mixture was warmed to 25 °C and stirred for an additional 0.5 h. $NaBH_4$ (0.3 mmol) was added at 0 °C. The mixture was allowed to warm to 25 °C and stirred for 1 h until the reaction was complete (monitored by TLC). The reaction was quenched with H_2O, and diluted with EtOAc. The aqueous phase was separated and extracted twice with EtOAc. The combined organic phase was dried over anhydrous $MgSO_4$ and concentrated in vacuo. The crude products were purified by flash column chromatography to afford **7** (petroleum ether/ethyl acetate, 4:1).

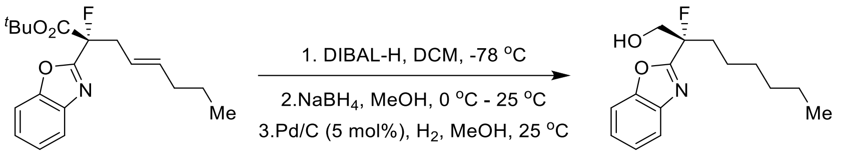

3a **8**

DIBAL-H (1.0 M in toluene, 0.3 mmol) was added dropwise to a solution of **3a** (0.2 mmol) in DCM (2.0 mL) via a syringe at -78 °C. The reaction mixture was stirred at -78 °C for 10 min until the reaction was complete (monitored by TLC). A saturated NH_4Cl solution (5 mL) and HCl (1 M, 1 mL) were added to quench the reaction and the aqueous layer was extracted with DCM (5 mL × 3). The combined organic layers were dried over anhydrous $MgSO_4$, filtered, and concentrated in vacuo. The residue was dissolved in MeOH (2 mL) and $NaBH_4$ (0.3 mmol) was added at 0 °C. The mixture was allowed to warm to 25 °C and stirred for 1 h until the reaction was complete (monitored by TLC). The reaction was quenched with H_2O and diluted with EtOAc. The aqueous phase was separated and extracted twice with EtOAc. The combined organic phase dried over anhydrous $MgSO_4$ and concentrated in vacuo. The residue was dissolved in dry MeOH (1 mL) and Pd/C (0.01 mmol) was added. The resulting mixture was stirred at 25 °C in an atmosphere of hydrogen gas until the reaction was complete (monitoring by TLC). Pd/C was removed through a short silica gel column and concentrated in vacuo, and the residue was purified by silica gel chromatography to afford the desired product **8** (petroleum ether/ethyl acetate, 4:1).

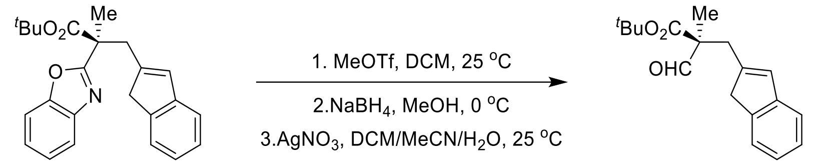

3x **9**

Under an argon atmosphere, a mixture composed of **3x** (0.2 mmol), 200 mg of 4 Å molecular sieves, and 2 mL of dry dichloromethane (DCM) was stirred at 25 °C for 10 minutes. Subsequently, 1 mmol of methyl trifluoromethanesulfonate (MeOTf) was added to the mixture. The mixture was stirred for 6 h until the reaction was complete (monitored by TLC). The molecular sieves were removed through a short silica gel column and concentrated in vacuo. The residue was dissolved in MeOH (2 mL), and $NaBH_4$ (0.3 mmol) was added at 0 °C. The mixture was stirred for 2 min until the reaction was complete (monitored by TLC). The reaction was quenched with H_2O, and diluted with EtOAc. The aqueous phase was separated and extracted twice with EtOAc. The combined organic phase was dried over anhydrous $MgSO_4$ and concentrated in vacuo. The residue was dissolved in MeCN/DCM/H_2O (1.5 mL/0.5 mL/0.3 mL) and $AgNO_3$ (0.6 mmol) was added. The mixture was stirred at 50 °C for 24 h until the reaction was complete (monitored by TLC). Then, the mixture was diluted with 1 M phosphate buffer at pH 7 (5 mL). After stirring for 30 min, the reaction mixture was extracted twice with EtOAc. The combined organic

phase was dried over anhydrous $MgSO_4$ and concentrated in vacuo. The crude products were purified by flash column chromatography to afford **9** (petroleum ether/ethyl acetate, 15:1).

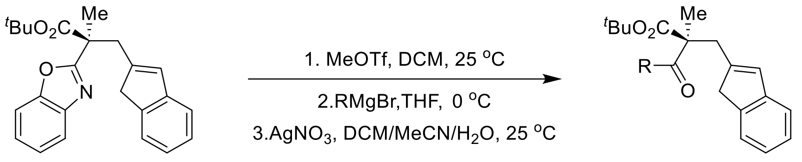

3x

R = Me, **10**
R = Ph, **11**

Under an argon atmosphere, a mixture composed of **3x** (0.2 mmol), 200 mg of 4 Å molecular sieves powder, and 2 mL of dry dichloromethane (DCM) was stirred at 25 °C for 10 minutes. Subsequently, 1 mmol of methyl trifluoromethanesulfonate (MeOTf) was added to the mixture. The mixture was stirred for 6 h until the reaction was complete (monitored by TLC). The molecular sieves were removed through a short silica gel column and concentrated in vacuo. The residue was dissolved in THF (2 mL) and RMgBr (0.3 mmol) was added at 0 °C. The mixture was stirred for 2 min until the reaction was complete (monitored by TLC). The reaction was quenched with saturated NH_4Cl solution, and diluted with EtOAc. The aqueous phase was separated and extracted twice with EtOAc. The combined organic phase was dried over anhydrous $MgSO_4$ and concentrated in vacuo. The residue was dissolved in MeCN/DCM/H_2O (1.5 mL/0.5 mL/0.3 mL) and $AgNO_3$ (0.6 mmol) was added. The mixture was stirred at 50 °C for 12 h until the reaction was complete (monitored by TLC). Then the mixture was diluted with 1 M phosphate buffer at pH 7 (5 mL). After stirring for 30 min, the reaction mixture was extracted twice with EtOAc. The combined organic phase was dried over anhydrous $MgSO_4$ and concentrated in vacuo. The crude products were purified by flash column chromatography to afford **10** (petroleum ether/ethyl acetate, 25:1) and **11** (petroleum ether/ethyl acetate, 25:1).

6.4 Synthesis of (*S*)-SYK inhibitor

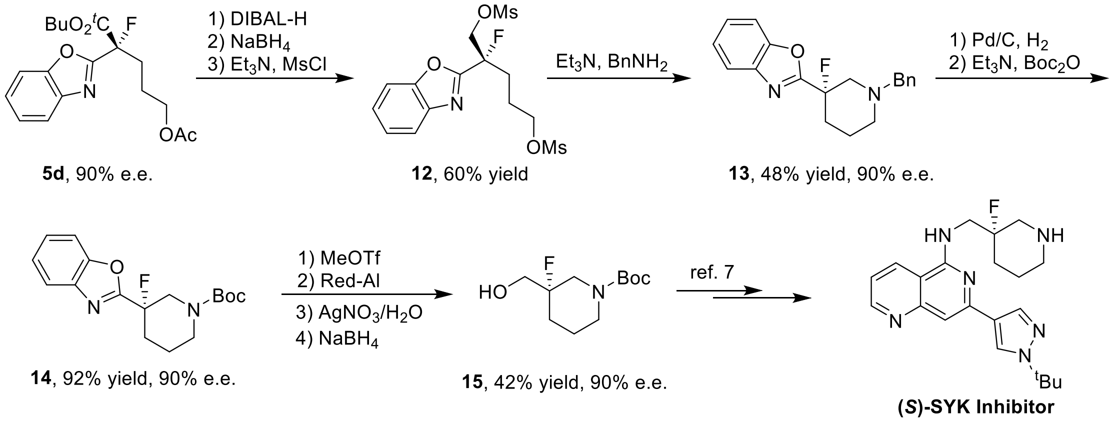

5d, 90% e.e. **12**, 60% yield **13**, 48% yield, 90% e.e. **14**, 92% yield, 90% e.e. **15**, 42% yield, 90% e.e. **(*S*)-SYK Inhibitor**

DIBAL-H (1.0 M in toluene, 6 mmol) was added dropwise to a solution of **5d** (2 mmol) in DCM (20 mL) via a syringe at -78 °C. The reaction mixture was stirred at -78 °C for 20 min until the reaction was complete (monitored by TLC). A saturated NH_4Cl solution (10 mL) and HCl (1 M, 20 mL) were added to quench the reaction, and the aqueous layer was extracted with DCM (20 mL × 3). The combined organic layers were dried over anhydrous $MgSO_4$, filtered, and concentrated in vacuo. The residue was dissolved in MeOH (20 mL) and $NaBH_4$ (6 mmol) was added at 0 °C. The mixture was allowed to warm

to 25 ºC and stirred for 6 h until the reaction was complete (monitored by TLC). The reaction was quenched with H_2O, and diluted with EtOAc. The aqueous phase was separated and extracted twice with EtOAc. The combined organic phase was dried over anhydrous $MgSO_4$ and concentrated in vacuo. The residue was dissolved in DCM (20 mL). Et_3N (8 mmol) and MsCl (8 mmol) were added at 0 ºC. The mixture was allowed to warm to 25 ºC and stirred for 3 h until the reaction was complete (monitored by TLC). The solvent was removed under reduced pressure. The residue was purified by silica gel chromatography to afford the desired product **12** (petroleum ether/ethyl acetate, 4:1).

Under an Ar atmosphere, a mixture of **12** (1.2 mmol), Et_3N (3.6 mmol), $BnNH_2$ (12 mmol) and dry 1,4-dioxane (50 mL) was stirred for 24 h at 100 ºC. The solvent was removed under reduced pressure. The residue was purified by silica gel chromatography to afford the desired product **13** (petroleum ether/ethyl acetate, 1:1).

The reaction flask with a hydrogen balloon was charged with **13** (0.5 mmol), Pd/C (palladium on activated carbon, 10%; wetted with ca. 55% water) (0.025 mmol, 50 wt%), and dry EtOH (5.0 mL). After stirring at 60 ºC for 4 h, the starting material was consumed completely (monitored by TLC). Pd/C was removed through a short silica gel column and concentrated in vacuo. The residue was dissolved in DCM (5 mL). Et_3N (2 mmol) and Boc_2O (1.5 mmol) were added at 0 ºC. The mixture was allowed to warm to 25 ºC and stirred for 3 h until the reaction was complete (monitored by TLC). The solvent was removed under reduced pressure. The residue was purified by silica gel chromatography to afford the desired product **14** (petroleum ether/ethyl acetate, 10:1).

Under an argon atmosphere, a mixture was prepared by combining 0.4 mmol of compound 14, 200 mg of powdered 4 Å molecular sieves, and 2 mL of dry dichloromethane (DCM). This mixture was then stirred at 25 °C for 10 minutes. After that, 2 mmol of methyl trifluoromethanesulfonate (MeOTf) was added to the stirred mixture. The mixture was stirred for 4 h until the reaction was complete (monitored by TLC). The molecular sieves were removed through a short silica gel column and concentrated in vacuo. The residue was dissolved in THF (2 mL) and Red-Al (3.5 M in toluene, 0.7 mmol) was added at 0 ºC. The mixture was stirred for 2 min until the reaction was complete (monitored by TLC). The reaction was quenched with saturated NH_4Cl solution, and diluted with EtOAc. The aqueous phase was separated and extracted twice with EtOAc. The combined organic phase was dried over anhydrous $MgSO_4$ and concentrated in vacuo. The residue was dissolved in MeCN/DCM/H_2O (1.5 mL/0.5 mL/0.3 mL) and $AgNO_3$ (1.2 mmol) was added. The mixture was stirred at 50 ºC for 12 h until the reaction was complete (monitored by TLC). Then, the mixture was diluted with 1 M phosphate buffer at pH 7 (5 mL). After stirring for 30 min, the reaction mixture was extracted twice with EtOAc. The combined organic phase was dried over anhydrous $MgSO_4$ and concentrated in vacuo. The residue was dissolved in MeOH (5 mL) and $NaBH_4$ (1.2 mmol) was added at 0 ºC. The mixture was allowed to warm to 25 ºC and stirred for 1 h until the reaction was complete (monitored by TLC). The reaction was quenched with H_2O, and diluted with EtOAc. The aqueous phase was separated and extracted twice with EtOAc. The combined organic phase was dried over anhydrous $MgSO_4$ and concentrated in vacuo. The residue was purified by silica gel chromatography to afford the desired product **15** (petroleum ether/ethyl acetate, 10:1). According to the literature[7], **15** can be converted into the (*S*)-SYK inhibitor.

***Tert*-butyl (*R*)-2-(benzo[*d*]oxazol-2-yl)-2-fluoropent-4-enoate (3al)**

The title compound was prepared according to general procedure A using **1a** (0.1 mmol) under a propylene atmosphere (0.5 mmol). The crude reaction mixture was purified by flash column

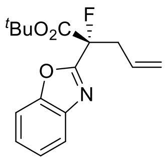

chromatography to afford the title compound (petroleum ether/ethyl acetate, 25:1). (52% yield). **^{1}H NMR (400 MHz, CDCl$_3$)** δ 7.86–7.73 (m, 1H), 7.61–7.52 (m, 1H), 7.47–7.34 (m, 2H), 6.01–5.86 (m, 1H), 5.35–5.20 (m, 2H), 3.40–3.10 (m, 2H), 1.47 (s, 9H). **^{13}C NMR (151 MHz, CDCl$_3$)** δ 165.17 (d, *J* = 27.5 Hz), 160.54 (d, *J* = 25.2 Hz), 150.97, 140.51, 129.39 (d, *J* = 3.1 Hz), 126.36, 125.04, 121.09, 121.07, 111.27, 91.40 (d, *J* = 191.1 Hz), 84.58, 39.67 (d, *J* = 21.5 Hz), 28.02. **^{19}F NMR (376 MHz, CDCl$_3$)** δ -157.99. **ESI-MS: calculated [$C_{16}H_{18}FNO_3$ + Na]$^+$: 314.1163, found: 314.1172.** $[\alpha]^{20}_D$ = -12.96 (c = 5.83, CH_2Cl_2). The product was analyzed by HPLC to determine the enantiomeric excess: 91% e.e. (CHIRALPAK IG, hexane/*i*-PrOH = 98/20 detector: 254 nm, T = 25 °C, flow rate: 1 mL/min), t_1(minor) = 6.76 min, t_2(major) = 7.98 min.

Tert-butyl (_R_)-2-(benzo[_d_]oxazol-2-yl)-2-fluoro-4-hydroxybutanoate (7)

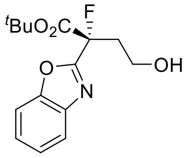

^{1}H NMR (400 MHz, CDCl$_3$) δ 7.82–7.75 (m, 1H), 7.62–7.53 (m, 1H), 7.47–7.34 (m, 2H), 3.99 (t, *J* = 5.4 Hz, 2H), 2.95–2.60 (m, 2H), 1.48 (s, 9H). **^{13}C NMR (151 MHz, CDCl$_3$)** δ 165.93 (d, *J* = 27.5 Hz), 160.78 (d, *J* = 25.1 Hz), 150.92, 139.99, 126.61, 125.19, 121.02, 111.33, 91.03 (d, *J* = 189.3 Hz), 84.89, 57.16 (d, *J* = 4.5 Hz), 37.89 (d, *J* = 20.9 Hz), 27.91. **^{19}F NMR (376 MHz, CDCl$_3$)** δ -157.36. **ESI-MS: calculated [$C_{15}H_{18}FNO_4$ + Na]$^+$: 318.1112, found: 318.1118.** $[\alpha]^{20}_D$ = -2.20 (c = 5.20, CH_2Cl_2). The product was analyzed by HPLC to determine the enantiomeric excess: 92% e.e. (CHIRALPAK IG, hexane/*i*-PrOH = 92/8, detector: 254 nm, T = 25 °C, flow rate: 1 mL/min), t_1(major)= 12.23 min, t_2(minor) = 13.29 min.

(_S_)-2-(benzo[_d_]oxazol-2-yl)-2-fluorooctan-1-ol (8)

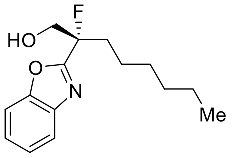

^{1}H NMR (400 MHz, CDCl$_3$) δ 7.81–7.71 (m, 1H), 7.62–7.55 (m, 1H), 7.44–7.33 (m, 2H), 4.26–4.05 (m, 2H), 2.68 (t, *J* = 7.1 Hz, 1H), 2.26–2.08 (m, 2H), 1.50–1.39 (m, 1H), 1.34–1.22 (m, 6H), 0.89–0.81 (m, 3H). **^{13}C NMR (151 MHz, CDCl$_3$)** δ 163.54 (d, *J* = 25.9 Hz), 150.65, 140.38, 125.87, 124.97, 120.58, 111.22, 95.72 (d, *J* = 178.3 Hz), 66.14 (d, *J* = 24.5 Hz), 34.90 (d, *J* = 22.5 Hz), 31.61, 29.37, 23.00 (d, *J* = 4.4 Hz), 22.63, 14.16. **^{19}F NMR (376 MHz, CDCl$_3$)** δ -166.56. **ESI-MS: calculated [$C_{15}H_{20}FNO_2$ + H]$^+$: 266.1551, found: 266.1547.** $[\alpha]^{20}_D$ = -6.81 (c = 4.77, CH_2Cl_2). The product was analyzed by HPLC to determine the enantiomeric excess: 92% e.e. (CHIRALPAK IG, hexane/*i*-PrOH = 92/8, detector: 254 nm, T = 25 °C, flow rate: 1 mL/min), t_1(minor) = 7.56 min, t_2(major) = 8.27 min.

Tert-butyl (_S_)-2-((1_H_-inden-2-yl)methyl)-2-methyl-3-oxopropanoate (9)

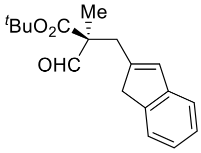

^{1}H NMR (500 MHz, CDCl$_3$) δ 9.78 (s, 1H), 7.37 (dd, *J* = 7.3, 0.9 Hz, 1H), 7.29–7.26 (m, 1H), 7.22 (td, *J* = 7.4, 1.1 Hz, 1H), 7.13 (td, *J* = 7.4, 1.3 Hz, 1H), 6.58 (s, 1H), 3.32–3.26 (m, 2H), 3.06 (d, *J* = 14.6 Hz, 1H), 2.97 (d, *J* = 14.5 Hz, 1H), 1.45 (s, 9H), 1.32 (s, 3H). **^{13}C NMR (126 MHz, CDCl$_3$)** δ 199.83, 171.17, 144.77, 143.77, 143.44, 131.06, 126.46, 124.42, 123.51, 120.46, 82.70, 58.52, 42.40, 35.76, 28.08, 17.95. **ESI-MS: calculated [$C_{18}H_{22}O_3$ + Na]$^+$: 309.1461, found: 309.1469.** $[\alpha]^{20}_D$ = -1.84 (c = 3.40, CH_2Cl_2). The product was analyzed by HPLC to determine the enantiomeric excess: 92% e.e. (CHIRALPAK IG, hexane/*i*-PrOH = 98/2, detector: 254 nm, T = 25 °C, flow rate: 1 mL/min), t_1(minor) = 7.39 min, t_2(major) = 9.30 min.

***Tert*-butyl (*S*)-2-((1*H*-inden-2-yl)methyl)-2-methyl-3-oxobutanoate (10)**

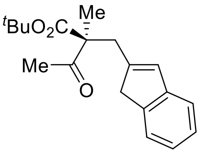

^{1}H NMR (500 MHz, $CDCl_3$) δ 7.36 (dd, *J* = 7.3, 0.9 Hz, 1H), 7.29–7.26 (m, 1H), 7.22 (td, *J* = 7.4, 1.1 Hz, 1H), 7.12 (td, *J* = 7.4, 1.3 Hz, 1H), 6.56 (s, 1H), 3.30–3.25 (m, 2H), 3.10 (d, *J* = 14.8 Hz, 1H), 2.98 (d, *J* = 14.8 Hz, 1H), 2.21 (s, 3H), 1.44 (s, 9H), 1.35 (s, 3H). **^{13}C NMR (126 MHz, $CDCl_3$)** δ 205.56, 171.93, 144.91, 144.74, 143.58, 130.74, 126.40, 124.27, 123.48, 120.36, 82.26, 60.73, 42.38, 36.37, 27.93, 26.33, 19.61. **ESI-MS: calculated [$C_{19}H_{24}O_3$ + Na]$^+$: 323.1618, found: 323.1620.** $[\alpha]^{20}_{D}$ = -9.11 (c = 3.33, CH_2Cl_2). The product was analyzed by HPLC to determine the enantiomeric excess: 92% e.e. (CHIRALPAK IG, hexane/*i*-PrOH = 98/2, detector: 254 nm, T = 25 °C, flow rate: 1 mL/min), t_1(minor) = 7.60 min, t_2(major) = 8.29 min.

***Tert*-butyl (*S*)-2-((1*H*-inden-2-yl)methyl)-2-methyl-3-oxo-3-phenylpropanoate (11)**

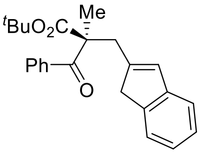

^{1}H NMR (500 MHz, $CDCl_3$) δ 7.99–7.88 (m, 2H), 7.57–7.51 (m, 1H), 7.48–7.42 (m, 2H), 7.34 (dt, *J* = 7.3, 0.9 Hz, 1H), 7.29–7.24 (m, 1H), 7.21 (td, *J* = 7.5, 1.1 Hz, 1H), 7.11 (td, *J* = 7.4, 1.2 Hz, 1H), 6.56 (s, 1H), 3.35–3.18 (m, 4H), 1.59 (s, 3H), 1.24 (s, 9H). **^{13}C NMR (126 MHz, $CDCl_3$)** δ 197.89, 173.02, 144.85, 144.71, 143.83, 135.97, 132.74, 131.29, 128.91, 128.52, 126.29, 124.20, 123.44, 120.32, 82.36, 58.29, 42.36, 38.49, 27.65, 21.95. **ESI-MS: calculated [$C_{24}H_{26}O_3$ + Na]$^+$: 385.1774, found: 385.1777.** $[\alpha]^{20}_{D}$ = 20.87 (c = 11.40, CH_2Cl_2). The product was analyzed by HPLC to determine the enantiomeric excess: 92% e.e. (CHIRALPAK IC, hexane/*i*-PrOH = 98/2, detector: 254 nm, T = 25 °C, flow rate: 1 mL/min), t_1(minor) = 6.97 min, t_2(major) = 9.40 min.

(*S*)-2-(benzo[*d*]oxazol-2-yl)-2-fluoropentane-1,5-diyl dimethanesulfonate (12)

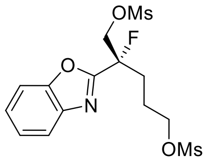

^{1}H NMR (600 MHz, $CDCl_3$) δ 7.77–7.70 (m, 1H), 7.63–7.56 (m, 1H), 7.46–7.36 (m, 2H), 4.76 (dd, *J* = 19.4, 1.5 Hz, 2H), 4.25 (t, *J* = 6.1 Hz, 2H), 3.06 (s, 3H), 2.99 (s, 3H), 2.45–2.33 (m, 2H), 2.00–1.90 (m, 1H), 1.90–1.81 (m, 1H). **^{13}C NMR (151 MHz, $CDCl_3$)** δ 160.42 (d, *J* = 25.4 Hz), 150.77, 140.24, 126.45, 125.31, 120.79, 111.31, 93.08 (d, *J* = 184.7 Hz), 70.24 (d, *J* = 24.7 Hz), 68.83, 37.97, 37.47, 30.53 (d, *J* = 22.4 Hz), 23.01 (d, *J* = 4.8 Hz). **^{19}F NMR (471 MHz, $CDCl_3$)** δ -163.85. **ESI-MS: calculated [$C_{14}H_{18}FNO_7S_2$ + H]$^+$: 396.0581, found: 396.0585.** $[\alpha]^{20}_{D}$ =-6.95 (c = 3.03, CH_2Cl_2).

(*S*)-2-(1-benzyl-3-fluoropiperidin-3-yl)benzo[*d*]oxazole (13)

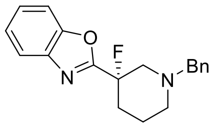

^{1}H NMR (500 MHz, $CDCl_3$) δ 7.82–7.77 (m, 1H), 7.60–7.52 (m, 1H), 7.44–7.35 (m, 2H), 7.34–7.23 (m, 5H), 3.68 (d, *J* = 13.4 Hz, 1H), 3.63 (d, *J* = 13.3 Hz, 1H), 3.17–3.01 (m, 2H), 2.71–2.63 (m, 1H), 2.49–2.35 (m, 2H), 2.31–2.18 (m, 1H), 2.08–1.99 (m, 1H), 1.91–1.81 (m, 1H). **^{13}C NMR (151 MHz, $CDCl_3$)** δ 163.91 (d, *J* = 24.4 Hz), 150.68, 140.69, 137.54, 129.11, 128.33, 127.29, 125.92, 124.76, 120.72, 111.14, 90.05 (d, *J* = 176.4 Hz), 62.53, 58.75 (d, *J* = 24.7 Hz), 52.73, 32.32 (d, *J* = 21.7 Hz), 21.86 (d, *J* = 4.2 Hz). **ESI-MS: calculated [$C_{19}H_{19}FN_2O$ + H]$^+$: 311.1554, found: 3111.155**6. $[\alpha]^{20}_{D}$ = -40.29 (c = 2.30, CH_2Cl_2). The product was analyzed by HPLC to determine the enantiomeric excess: 90% e.e. (CHIRALPAK IG, hexane/*i*-PrOH = 98/2, detector: 254 nm, T = 25 °C, flow rate: 1 mL/min), t_1(minor) = 8.10 min, t_2(major) = 8.68 min.

***Tert*-butyl (*S*)-3-(benzo[*d*]oxazol-2-yl)-3-fluoropiperidine-1-carboxylate (14)**

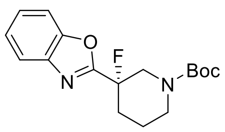

^{1}H NMR (500 MHz, $CDCl_3$) δ 7.76 (d, *J* = 7.7 Hz, 1H), 7.58 (d, *J* = 7.8 Hz, 1H), 7.44–7.33 (m, 2H), 4.60–4.17 (m, 1H), 4.00–3.47 (m, 2H), 3.21–3.01 (m, 1H), 2.47–2.19 (m, 2H), 2.05–1.89 (m, 1H), 1.84–1.68 (m, 1H), 1.42 (s, 9H). **^{13}C NMR (151 MHz, $CDCl_3$)** δ 163.08 (d, *J* = 23.9 Hz), 154.80, 150.72, 140.54, 126.11, 124.94, 120.75, 111.17, 88.84 (d, *J* = 180.2 Hz), 80.29, 50.46 (d, *J* = 26.2 Hz), 42.90, 32.82 (d, *J* = 23.0 Hz), 28.38, 20.88. **^{19}F NMR (565 MHz, CDCl3)** δ -158.04. **ESI-MS: calculated [$C_{17}H_{21}FN_2O_3$ + Na]$^+$: 343.1428, found: 343.1431.** $[\alpha]^{20}_D$ = -46.00 (c = 0.63, CH_2Cl_2). The product was analyzed by HPLC to determine the enantiomeric excess: 90% e.e. (CHIRALPAK IG, hexane/*i*-PrOH = 95/5, detector: 245 nm, T = 25 °C, flow rate: 1 mL/min), t_1(minor) = 10.68 min, t_2(major) = 11.73 min.

***Tert*-butyl (*S*)-3-fluoro-3-(hydroxymethyl)piperidine-1-carboxylate (15)**

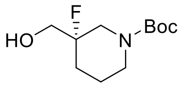

^{1}H NMR (600 MHz, DMSO-*D_6*) δ 5.04 (t, *J* = 6.0 Hz, 1H), 4.03–3.73 (m, 2H), 3.49 – 3.36 (m, 2H), 3.08–2.71 (m, 2H), 1.77–1.67 (m, 1H), 1.64–1.51 (m, 2H), 1.49–1.43 (m, 1H), 1.38 (s, 9H). **^{13}C NMR (151 MHz, DMSO-*D_6*)** δ 154.30, 93.44 (d, *J* = 175.6 Hz), 78.67, 64.80 (d, *J* = 23.5 Hz), 47.93 (dd, *J* = 178.6, 23.6 Hz), 43.06 (d, *J* = 158.8 Hz), 29.39 (d, *J* = 21.9 Hz), 28.08, 20.40 (d, *J* = 40.0 Hz). **^{19}F NMR (565 MHz, DMSO-*D_6*)** δ -165.81. **ESI-MS: calculated [$C_{11}H_{20}FNO_3$ + Na]$^+$: 256.1319, found: 256.1328.** $[\alpha]^{20}_D$ = -5.26 (c = 1.43, CH_2Cl_2). The e.e. value of **15** was determined by chiral HPLC analysis of the *p*-nitrobenzoate derivative **15'.**

***Tert*-butyl (*S*)-3-fluoro-3-(((4-nitrobenzoyl)oxy)methyl)piperidine-1-carboxylate (15')**

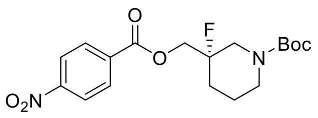

Et_3N (0.2 mmol) and 4-nitrobenzoyl chloride (0.3 mmol) were added to a solution of **15** (0.1 mmol) in DCM (1 mL). The reaction mixture was stirred for 1 h until the reaction was complete (monitored by TLC). The solvent was removed under reduced pressure. The residue was purified by silica gel chromatography to afford the desired product **15'**. **^{1}H NMR (600 MHz, $CDCl_3$)** δ 8.30 (d, *J* = 8.5 Hz, 2H), 8.25 (d, *J* = 8.5 Hz, 2H), 4.52–4.35 (m, 2H), 4.04–3.85 (m, 1H), 3.79–3.67 (m, 1H), 3.39–3.30 (m, 1H), 3.19–3.13 (m, 1H), 2.03–1.65 (m, 3H), 1.64–1.56 (m, 1H), 1.44 (s, 9H). **^{13}C NMR (151 MHz, $CDCl_3$)** δ 164.27, 154.91, 150.86, 135.02, 131.04, 123.80, 91.18 (d, *J* = 179.0 Hz), 80.30, 67.39, 48.44 (d, *J* = 169.1 Hz), 43.67 (d, *J* = 152.8 Hz), 30.86 (d, *J* = 21.7 Hz), 28.46, 21.15 (d, *J* = 38.6 Hz). **^{19}F NMR (565 MHz, $CDCl_3$)** δ -164.42. **ESI-MS: calculated [$C_{18}H_{23}FN_2O_6$ + Na]$^+$: 405.1432, found: 405.1426.** The product was analyzed by HPLC to determine the enantiomeric excess: 90% e.e. (CHIRALPAK IG, hexane/*i*-PrOH = 90/10, detector: 254 nm, T = 25 °C, flow rate: 1 mL/min), t_1(major) = 17.53 min, t_2(minor) = 18.99 min.

7. Cyclic voltammetry studies

Unless otherwise noted, the cyclic voltammograms were recorded on a CHI 760E instrument using a glassy carbon disk working electrode (diameter, 3 mm), a Pt wire auxiliary electrode, an SCE reference electrode, and a scan rate of 100 mV/s. The onset potential determined at the point where the current deviates from zero[8].

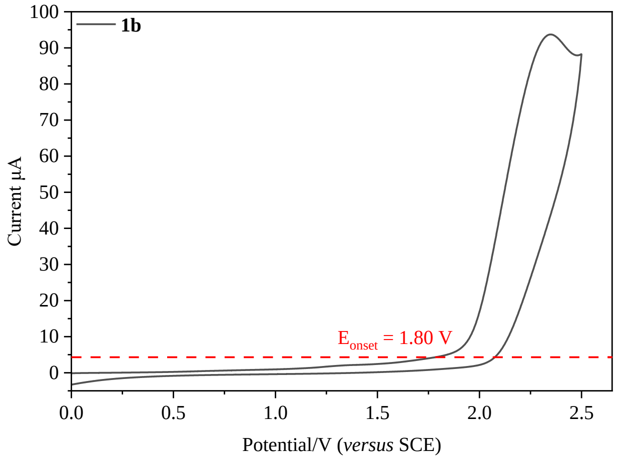

Figure S1. Cyclic voltammogram of **1b** (10 mM) in an electrolyte of $^{n}Bu_4NPF_6$ (0.1 M) with DCE (3.0 mL). The onset potential of **1b** was measured at approximately +1.80 V (versus SCE).

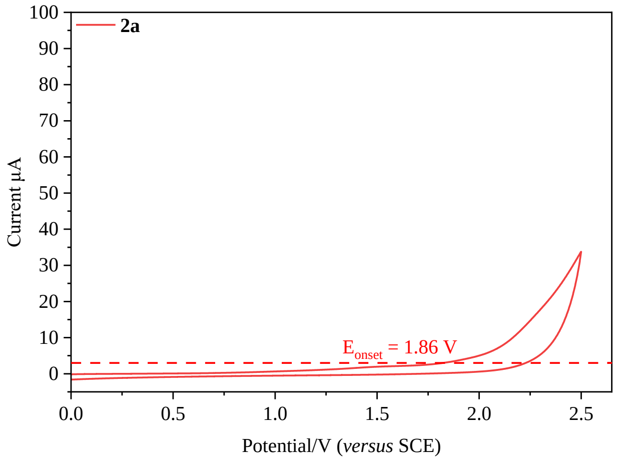

Figure S2. Cyclic voltammogram of **2a** (10 mM) in an electrolyte of $^{n}Bu_4NPF_6$ (0.1 M) with DCE (3.0 mL). The onset potential of **2a** was measured at approximately +1.86 V (versus SCE).

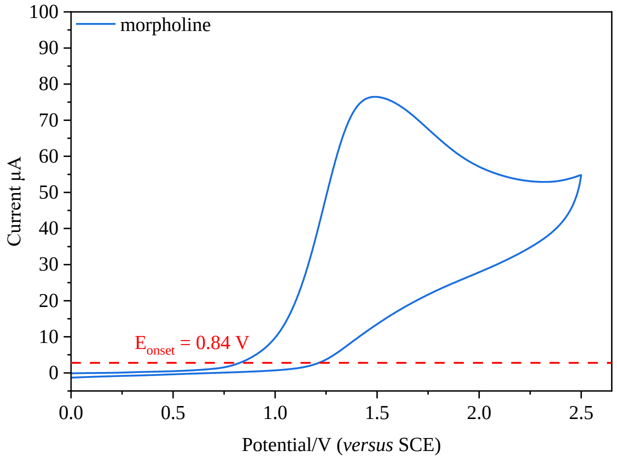

Figure S3. Cyclic voltammogram of morpholine (10 mM) in an electrolyte of ^{n}Bu$_4$NPF$_6$ (0.1 M) with DCE (3.0 mL). The onset potential of morpholine was measured at approximately +0.84 V (versus SCE).

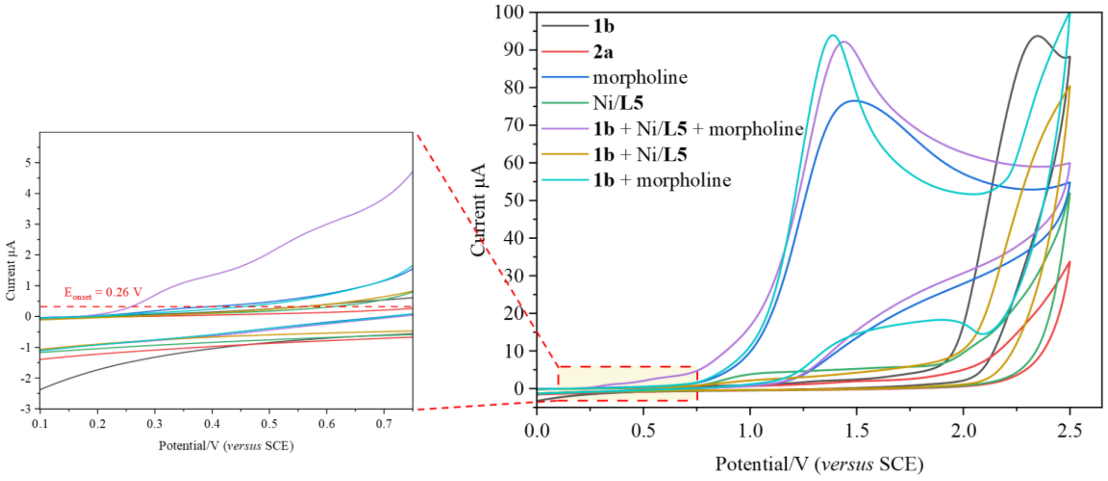

Figure S4. Cyclic voltammogram of [**1b** (10 mM) + morpholine (10 mM) + Ni-**L5** (2 mM)] in an electrolyte of ^{n}Bu$_4$NPF$_6$ (0.1 M) with DCE (3.0 mL). The onset potential of the nickel-bound enolate intermediate was measured at approximately +0.26 V (*versus* SCE).

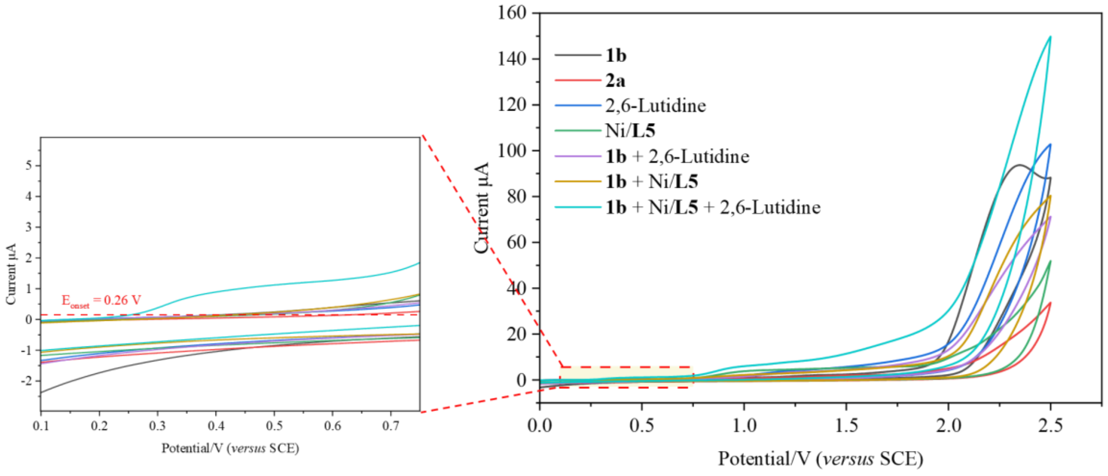

Figure S5. Cyclic voltammogram of [**1b** (10 mM) + 2,6-Lutidine (10 mM) + Ni-**L5** (2 mM)] in an electrolyte of ^{n}Bu$_4$NPF$_6$ (0.1 M) with DCE (3.0 mL). The onset potential of the nickel-bound enolate intermediate was measured at approximately +0.26 V (*versus* SCE).

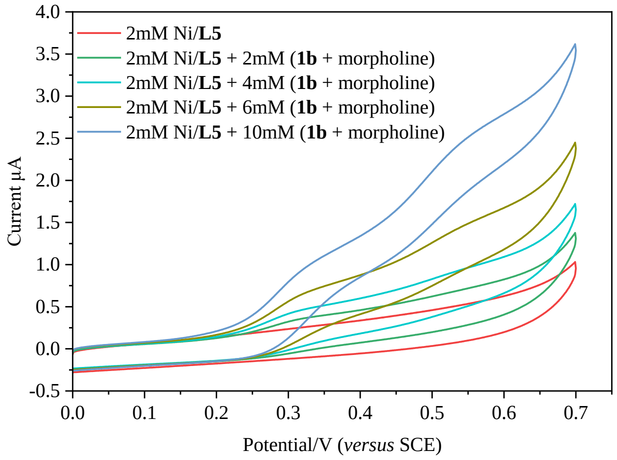

Figure S6. Titration of **1b** and morpholine (2 mM to 10 mM) to Ni-**L5** (2 mM) monitored by cyclic voltammetry in an electrolyte of $^{n}Bu_4NPF_6$ (0.1 M) with DCE (3.0 mL). The current response increased with increasing concentrations of **1b** and morpholine.

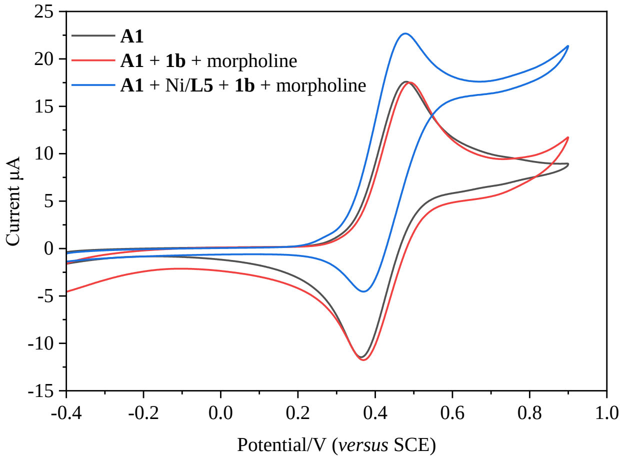

Figure S7. Cyclic voltammogram of [**A1** (1 mM) + Ni-**L5** (1 mM) + **1b** (10 mM) + morpholine (10 mM)] in an electrolyte of $^{n}Bu_4NPF_6$ (0.1 M) with DCE (3.0 mL).

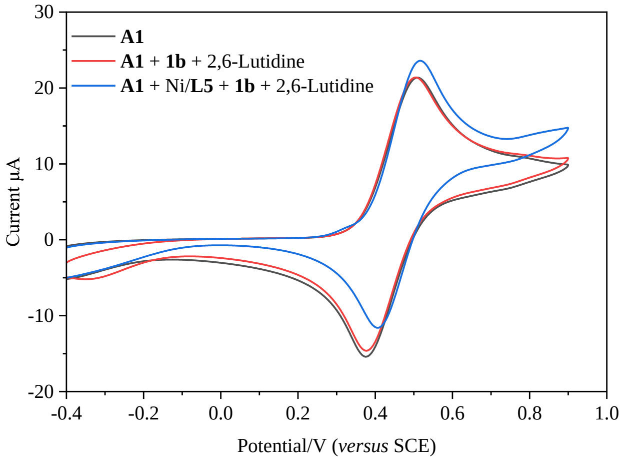

Figure S8. Cyclic voltammogram of [**A1** (1 mM) + Ni-**L5** (1 mM) + **1b** (10 mM) + 2,6-Lutidine (10 mM)] in an electrolyte of $^{n}Bu_4NPF_6$ (0.1 M) with DCE (3.0 mL).

8. Mechanistic studies

Deuteration experiment

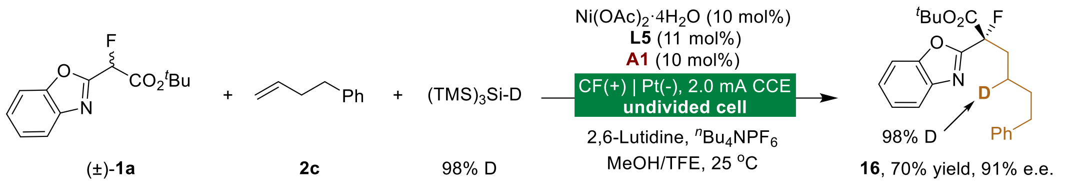

A 10 mL flask equipped with a magnetic stir bar was charged with **1a** (0.1 mmol), nickel complex (0.01 mmol), $Cp_2Fe\text{-}CH_2NMe_2$ (**A1**, 0.01 mmol) and $^nBu_4NPF_6$ (0.3 mmol). The flask was equipped with a carbon felt (1.5 cm × 1.5 cm × 3 mm) as the anode and a platinum plate (1.0 cm × 1.0 cm × 0.2 mm) as the cathode. The reaction mixture was degassed via vacuum evacuation and backfilled with argon three times. A solution of **2c** (2.0 mmol), $(TMS)_3SiD$ (98% D) (0.5 mmol), and 2,6-Lutidine (0.1 mmol) in MeOH (0.5 mL) and TFE (2.5 mL) were subsequently added under argon. Constant current (2.0 mA) electrolysis was carried out at 25 °C for 8 h until complete consumption of the starting material (monitored by TLC). The solvent was removed under reduced pressure. The residue was purified by silica gel chromatography to afford the desired product **16**.

Radical clock experiments

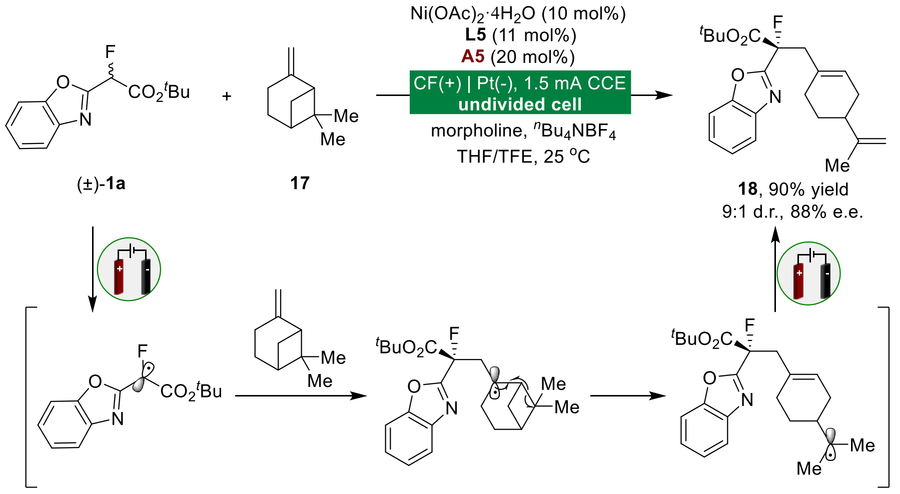

A 10 mL flask equipped with a magnetic stir bar was charged with **1a** (0.1 mmol), nickel complex (0.01 mmol), **A5** (0.02 mmol), and $^nBu_4NBF_4$ (0.3 mmol). The flask was equipped with a carbon felt (1.5 cm × 1.5 cm × 3 mm) as the anode and a platinum plate (1.0 cm × 1.0 cm × 0.2 mm) as the cathode. The reaction mixture was degassed via vacuum evacuation and backfilled with argon three times. A solution of **17** (0.5 mmol) and morpholine (0.1 mmol) in THF (0.5 mL) and TFE (2.5 mL) were subsequently added under argon. Constant current (1.5 mA) electrolysis was carried out at 25 °C for 8 h until complete consumption of the starting material (monitored by TLC). The solvent was removed under reduced pressure. The residue was purified by silica gel chromatography to afford the desired product **18**.

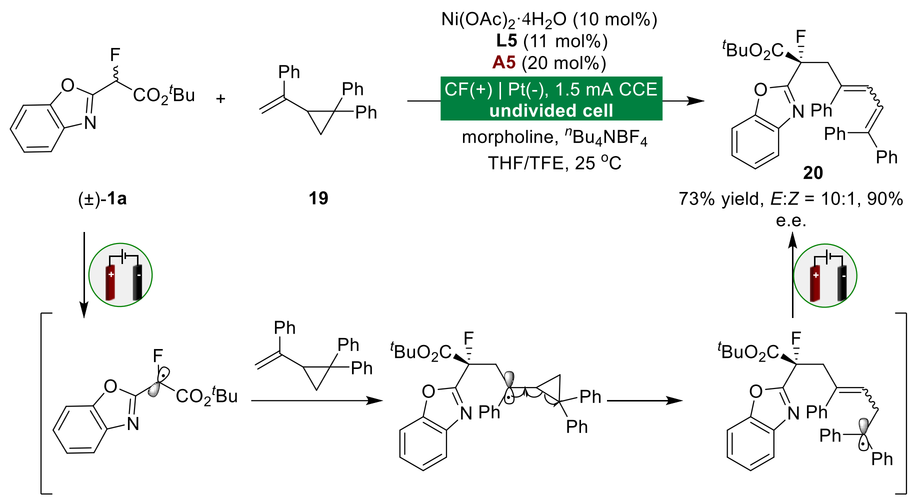

A 10 mL flask equipped with a magnetic stir bar was charged with **1a** (0.1 mmol), nickel complex (0.01 mmol), **A5** (0.02 mmol), and $^{n}Bu_4NBF_4$ (0.3 mmol). The flask was equipped with a carbon felt (1.5 cm × 1.5 cm × 3 mm) as the anode and a platinum plate (1.0 cm × 1.0 cm × 0.2 mm) as the cathode. The reaction mixture was degassed via vacuum evacuation and backfilled with argon three times. A solution of **19** (0.5 mmol) and morpholine (0.1 mmol) in THF (0.5 mL) and TFE (2.5 mL) were subsequently added under argon. Constant current (1.5 mA) electrolysis was carried out at 25 ºC for 8 h until complete consumption of the starting material (monitored by TLC). The solvent was removed under reduced pressure. The residue was purified by silica gel chromatography to afford the desired product **20**.

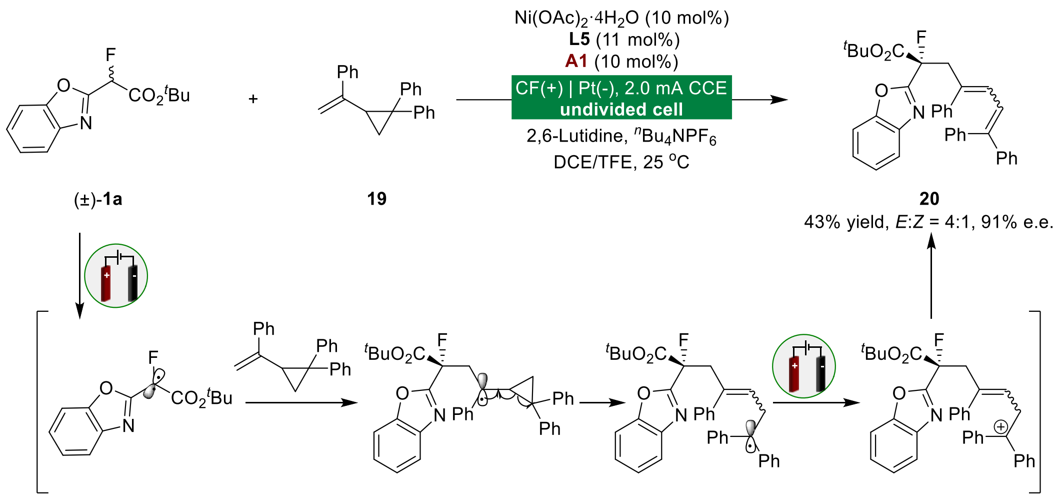

A 10 mL flask equipped with a magnetic stir bar was charged with **1a** (0.1 mmol), **19** (0.5 mmol), nickel complex (0.01 mmol), **A1** (0.01 mmol), and $^{n}Bu_4NPF_6$ (0.3 mmol). The flask was equipped with a carbon felt (1.5 cm × 1.5 cm × 3 mm) as the anode and platinum plate (1.0 cm × 1.0 cm × 0.2 mm) as the cathode. The reaction mixture was degassed via vacuum evacuation and backfilled with argon three times. A solution of 2,6-lutidine (0.1 mmol) in DCE (0.5 mL) and TFE (2.5 mL) was subsequently added under argon. Constant current (2.0 mA) electrolysis was carried out at 25 ºC for 6 h until complete consumption of the starting material (monitored by TLC). The solvent was removed under reduced pressure. The residue was purified by silica gel chromatography to afford the desired product **20.**

***Tert*-butyl (2*R*)-2-(benzo[*d*]oxazol-2-yl)-2-fluoro-6-phenylhexanoate-3-*d* (16)**

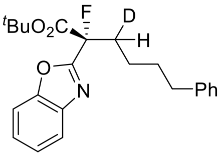

petroleum ether/ethyl acetate, 25:1, **^{1}H NMR (500 MHz, $CDCl_3$)** δ 7.83–7.76 (m, 1H), 7.60–7.55 (m, 1H), 7.45–7.35 (m, 2H), 7.31–7.24 (m, 2H), 7.21–7.14 (m, 3H), 2.73–2.63 (m, 2H), 2.60–2.44 (m, 2H), 1.84–1.64 (m, 3H), 1.45 (s, 9H). **^{13}C NMR (151 MHz, $CDCl_3$)** δ 165.77 (d, *J* = 27.6 Hz), 160.97 (d, *J* = 25.6 Hz), 150.92, 142.17, 140.52, 128.54, 128.47, 126.28, 125.92, 125.00, 121.05, 111.26, 92.23 (d, *J* = 190.2 Hz), 84.45, 35.61, 34.92 (d, *J* = 21.7 Hz), 31.09, 27.94, 22.31 – 21.90 (m). **^{19}F NMR (471 MHz, $CDCl_3$)** δ -159.06. **ESI-MS: calculated [$C_{23}H_{25}DFNO_3$ + Na]$^+$: 407.1852, found: 407.1857.** $[\alpha]^{20}_{D}$ = -12.38 (c = 6.47, CH_2Cl_2). The product was analyzed by HPLC to determine the enantiomeric excess: 91% e.e. (CHIRALPAK IG, hexane/*i*-PrOH = 98/2, detector: 254 nm, T = 25 °C, flow rate: 1 mL/min), t_1(minor) = 11.30 min, t_2(major) = 13.51 min.

***Tert*-butyl (2*R*)-2-(benzo[*d*]oxazol-2-yl)-2-fluoro-3-(4-(prop-1-en-2-yl)cyclohex-1-en-1-yl)propanoate (18)**

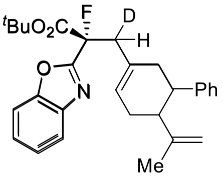

petroleum ether/ethyl acetate, 20:1, **^{1}H NMR (400 MHz, $CDCl_3$)** δ 7.84–7.74 (m, 1H), 7.63–7.53 (m, 1H), 7.45–7.34 (m, 2H), 5.72–5.65 (m, 1H), 4.74–4.66 (m, 2H), 3.33–3.09 (m, 2H), 2.42–2.26 (m, 1H), 2.19–2.04 (m, 3H), 2.03–1.91 (m, 1H), 1.87–1.76 (m, 1H), 1.72 (s, 3H), 1.54–1.37 (m, 1H), 1.46 (s, 9H). **^{13}C NMR (101 MHz, $CDCl_3$)** δ 165.19 (d, *J* = 27.9 Hz), 161.01 (d, *J* = 25.2 Hz), 150.90, 149.89, 140.48, 131.10, 127.57, 126.27, 124.98, 121.03, 111.25, 108.75, 92.77 (d, *J* = 192.9 Hz), 84.24, 43.20 (d, *J* = 20.3 Hz), 40.84, 30.99, 30.21, 30.19, 28.01, 20.89. **^{19}F NMR (376 MHz, $CDCl_3$)** δ -154.85. **ESI-MS: calculated [$C_{23}H_{28}FNO_3$ + H]$^+$: 386.2126, found: 386.2119.** $[\alpha]^{20}_{D}$ = -68.42 (c = 8.70, CH_2Cl_2). The product was analyzed by HPLC to determine the enantiomeric excess: 88% e.e. (CHIRALPAK IG, hexane/*i*-PrOH = 98/2, detector: 254 nm, T = 25 °C, flow rate: 1 mL/min), t_1(minor) = 8.90 min, t_2(major) = 9.96 min.

***Tert*-butyl (*R*)-2-(benzo[*d*]oxazol-2-yl)-2-fluoro-4,7,7-triphenylhepta-4,6-dienoate (20)**

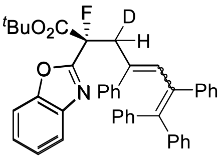

petroleum ether/ethyl acetate, 20:1, **^{1}H NMR (500 MHz, $CDCl_3$)** δ 7.81–7.74 (m, 1H), 7.53–7.46 (m, 1H), 7.40–7.35 (m, 4H), 7.33–7.24 (m, 8H), 7.23–7.19 (m, 4H), 7.16–7.09 (m, 2H), 6.62 (d, *J* = 11.6 Hz, 1H), 4.14–4.02 (m, 2H), 1.23 (s, 9H). **^{13}C NMR (126 MHz, $CDCl_3$)** δ 165.01 (d, *J* = 27.1 Hz), 160.89 (d, *J* = 25.5 Hz), 150.88, 145.19, 142.75, 142.72, 140.42, 139.75, 134.04, 132.03, 130.76, 128.30, 128.28, 128.07, 127.70, 127.65, 127.28, 127.05, 126.23, 124.95, 124.48 (d, *J* = 2.4 Hz), 120.98, 111.18, 91.73 (d, *J* = 195.2 Hz), 84.62, 35.25 (d, *J* = 21.5 Hz), 27.59. **^{19}F NMR (471 MHz, $CDCl_3$)** δ -155.68. **ESI-MS: calculated [$C_{36}H_{32}FNO_3$ + H]$^+$: 546.2439, found: 546.2433.** $[\alpha]^{20}_{D}$ = -7.15 (c = 9.63, CH_2Cl_2). The product was analyzed by HPLC to determine the enantiomeric excess: 90% e.e. (CHIRALPAK IC, hexane/*i*-PrOH = 98/2, detector: 254 nm, T = 25 °C, flow rate: 1 mL/min), t_1(minor) = 7.23 min, t_2(major) = 8.44 min.

9. X-ray crystallography data

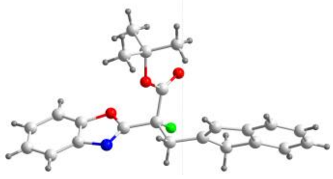

3t
(CCDC 2430133)

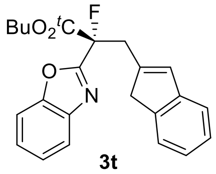

3t

Chemical formula	$C_{23}H_{22}FNO_3$
Formula weight	379.41
Space group	P 21 21 21
Z	4
a, Å	5.9987(1)
b, Å	16.2643(3)
c, Å	21,1634(4)
α, °	90.000
β, °	90.000
γ, °	90.000
V, $Å^3$	2064.80(6)

0.1 mmol of the sample (**3t** or **4l**) was recrystallized from dichloromethane/petroleum ether, affording a solid with an enantiomeric excess of >99%. The optically pure sample was dissolved in 1.8 mL of dichloromethane, and 0.6 mL of petroleum ether was added dropwise with shaking. The solution was then filtered through a membrane filter into a 3 mL vial. The vial was covered with filter paper containing small pores and left to stand at 25 °C until crystals formed. The crystals were collected for X-ray diffraction analysis. The diffraction data were processed and the structure was solved using standard methods with the SHELX suite. Absolute configuration was assigned based on Flack parameter refinement.

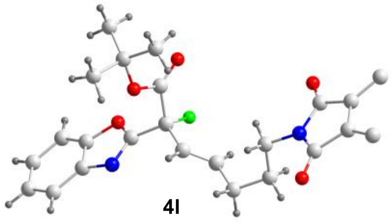

4l
(CCDC 2430132)

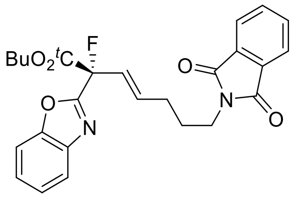

4l

Chemical formula	$C_{26}H_{25}FNO_5$
Formula weight	464.48
Space group	P 21 21 21
Z	4
a, Å	6.2772(1)
b, Å	10.6974(2)
c, Å	36.1669(8)
α, °	90.000
β, °	90.000
γ, °	90.000
V, Å^3	2428.60(8)

10. Computational details

Computational methods

All calculations were performed with Gaussian 16, Rev. C01 package[9]. The B3LYP functional[10], associated with the Grimme dispersion correction D3(BJ)[11] was used for geometry optimization of all structures. The SDD basis set (including related effective core potential)[12] was employed on Ni and Co and the 6-31G(d) basis set[13] was employed on other elements. Harmonic frequency calculations at the same level were performed to validate each structure as either a minimum or a transition state and to gain the thermal correction to Gibbs free energy at 298.15 K via Grimme's QRRHO[14] by Shermo (Version 2.5)[15]. Single-point energy calculations were conducted with the wB97X-D functional[16], def2-TZVP basis set[17], and SMD implicit solvation model[18]. Since the solvent parameters for the mixed solvent were not available under Gaussian 16, the full solvent parameters are defined by weighted averaging of the SMD parameters of pure solvents on the basis of the volume ratio. Unless otherwise stated, the mix solvent THF:TFE = 1:5 was used in alignment with the optimized experimental conditiod for the allylation reaction (Eps = 23.509, EpsInf = 1.717, HbondAcidity = 0.475, HbondBasicity = 0.288, SurfaceTensionAtInterface = 41.59, CarbonAromaticity = 0.0 and ElectronegativeHalogenicity = 0.417). 3D diagrams of the molecules were generated via CYLview20[19].

Discussion on spin states of cobalt and nickel species

We have considered the possible spin states of cobalt and nickel species present in the system. The spin-state changes occurring along the reaction pathway involve well-characterized species whose spin state are either documented in prior studies or can be reliably assigned via theoretical analysis[20-23]. Specifically, six-coordinate octahedral nickel (II) complexes adopt a high-spin configuration; cobalt centers coordinated by planar quadridentate ligands (such as Salen) favor a low-spin state; and the carbon radical generated during the reaction exhibits weak coupling with nickel, making the spin-parallel configuration slightly more stable than the spin-antiparallel (broken symmetry) configuration.

To further validate the chosen spin states, we computed the Gibbs free energies difference between the chosen spin state and another plausible spin state for the cobalt and nickel complexes (**Table S9**). Given that accurately predicting spin-state energy differences remains challenging within DFT, we performed single-point calculations using the TPSSh-D3(BJ)[11,24] and MN15L[25] functionals—selected based on benchmark studies in the literature[26,27]—instead of the wB97X-D functional. In all cases, the energy differences between the spin states employed in our study and other plausible spin states were positive, confirming that our choice correspond to the most stable spin states.

Table S9. Validation of the chosen spin states for the cobalt and nickel complexes.[a]

Entry	Complexes	The chosen spin state to another plausible spin state	ΔG(TPSSh-D3(BJ) Single-Point)/(kcal/mol)	ΔG(MN15L Single-Point) /(kcal/mol)
1	**$[Ni^{II}]^{+}OAc^{-}$**	t → s	15.3	21.8
2	**INT1**	t → s	14.1	20.4
3	**INT2**	t → s	15.3	22.2
4	**INT3**	q → d(BS)	1.0	2.1
5	**INT4**	q → d(BS)	0.4	0.4
6	**$[Co^{II}]$**	d → q	6.9	0.5
7	**INT6**	s → t	17.8	15.6
8	**$[Co^{I}]^{-}$**	s → t	7.0	2.1

a. Spin states are annotated as superscripts: s (singlet), d (doublet), t (triplet), q (quartet), and BS (broken symmetry).

The spin states of the transition states were logically deduced from those of the adjacent intermediates. For TS3, which corresponds to the hydrogen atom transfer (HAT) step, the reactants are two radical species, while the product is a closed-shell system. For such electron pairing processes, employing the broken-symmetry (BS) approach in DFT calculations is both appropriate and necessary[28].

Discussion of the electrochemical oxidation process

Values of electrochemical processes were calculated according to suggestions from Konezny, S.J. et al[29]. We use **A1**$^{+}$/ **A1** as a link between experiments and calculations. The potentials are obtained according to the following equations:

$$E_{calc}(vs.\,\boldsymbol{A1}^{+}/\boldsymbol{A1}) = E_{calc}(abs) - E_{calc,\boldsymbol{A1}^{+}/\boldsymbol{A1}}(abs)$$

$$E_{exp}(vs.\,\boldsymbol{A1}^{+}/\boldsymbol{A1}) = E_{exp}(vs.\,SCE) - E_{exp,\boldsymbol{A1}^{+}/\boldsymbol{A1}}(vs.\,SCE)$$

Thus, the calculated potentials $E_{calc}(vs.\,\boldsymbol{A1}^{+}/\boldsymbol{A1})$ and experimental potentials $E_{exp}(vs.\,\boldsymbol{A1}^{+}/\boldsymbol{A1})$ are comparable. Furthermore, the calculated potentials can be converted to the experimental scale referenced against the SCE, allowing for direct comparison with experimental values:

$$E_{calc}(vs.\,SCE) = E_{calc}(abs) - E_{calc,\boldsymbol{A1}^{+}/\boldsymbol{A1}}(abs) + E_{exp,\boldsymbol{A1}^{+}/\boldsymbol{A1}}(vs.\,SCE)$$

The oxidation potential of **A1** was measured to be 0.42 V (vs. SCE) by cyclic voltammetry (Fig. 6a, right). Next, the Gibbs free energy of electrochemical processes can be calculated using:

$$\Delta G = -nFE = -nF\left[E_{exp}^{external}(vs.\,SCE) - E_{calc}(vs.\,SCE)\right]$$

where $E_{exp}^{external}$ is the external potential used in our reaction systems.

Table S10. Calculated oxidation potentials.

Reduced form	Oxidized form	E_{calc}(vs. SCE)/V
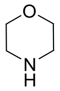<br>**morpholine**	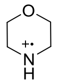<br>**morpholine^{+}**	1.35
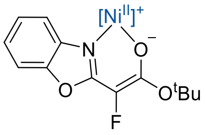	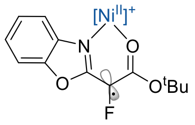	0.70
**INT2^{t}**	**INT3^{q}**	
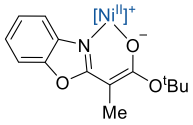	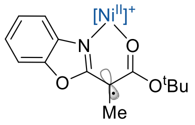	0.55
**INT2-1b^{t}**	**INT3-1b^{q}**	

The external potential was set to +0.26 V vs. SCE, corresponding to the experimentally measured onset potential for the nickel-bound enolate species. Thus, the energy profile shows an overall barrier of 21.4 kcal/mol at this potential (**Fig. 7a**), which falls within the expected range for room-temperature reactions (21 kcal/mol) and aligns well with our experimental observations.

Discussion of the radical addition process and the origin of enantioselectivity

Given the feasibility of dissociative ligand exchange in this catalytic system, four possible coordination modes of the nickel-bound α-carbonyl radical were identified (**Figure S9**). Among these, the structure of **INT3-1**^{q} lies only 1.0 kcal/mol higher in energy than the minimum-energy structure **INT3**^{q}, yet it has the opposite face selectivity. Consequently, two additional competing pathways may contribute to the formation of the minor stereoisomer: (i) **TS2-*Re*'**^{q}, involving the reaction of **INT3**^{q} with the alkene on the *Re* face *(unfavor)*, and (ii) **TS2-*Re***^{q}, where **INT3-1**^{q} reacts with the alkene on the *Re* face *(favor)*. Speciafically, **TS2-*Re***^{q} is significantly more favorable than **TS2-*Re*'**^{q}.

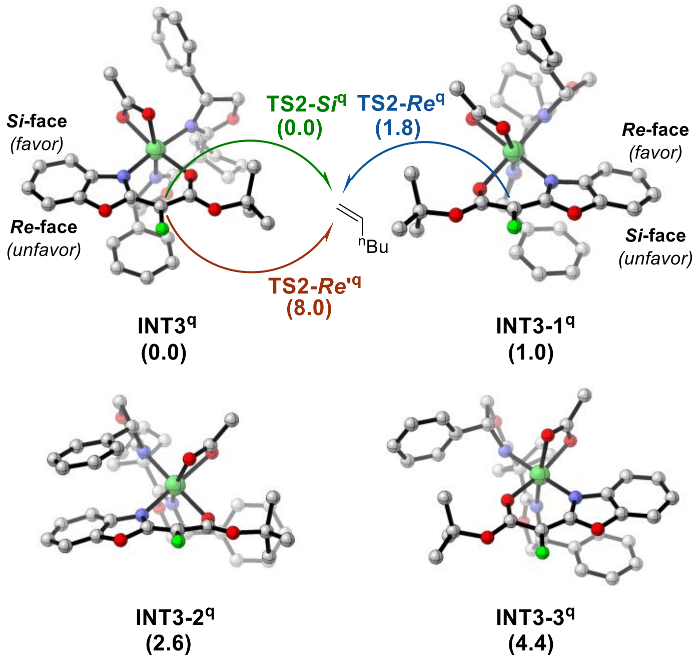

Figure S9. Relative Gibbs free energy of four possible coordination modes of nickel-bound α-carbonyl radicals and their corresponding transition states in kcal/mol.

To elucidate the origin of enantioselectivity of the radical addition process, we performed distortion-interaction analysis and energy decomposition analysis on the enantioselectivity-determining transition states **TS2-*Si***^{q} and **TS2-*Re***^{q} using sobEDA[30] method. Due to the incompatibility of the sobEDA method with the wB97X-D functional, the B3LYP-D3(BJ) functional was employed, as recommended in the original sobEDA article[30]. Initial distortion–interaction analysis (**Figure S10**, **a**) revealed that the transition state energy difference originates primarily from the distortion energy of the nickel-bound radical specie (**Fragment 1**, 2.9 kcal/mol), whereas both the distortion energy of **2a** (hexene, **Fragment 2**, -0.9 kcal/mol) and interaction energy (-0.6 kcal/mol) disfavor enantioselectivity. This suggests that the origin of enantioselectivity is likely governed by the conformation of the nickel-bound radical specie.

Further distortion-interaction analysis (**Figure S10**, **a**) focusing on the nickel-bound radical specie (**Fragment 1**) indicated that the energy difference arises mainly from the distortion energy of the nickel catalyst (**Fragment 3**, 1.4 kcal/mol), followed by contributions from the distortion energy of benzoxazolyl acetate radical (**Fragment 4**, 0.7 kcal/mol) and interaction energy (0.9 kcal/mol). The result implies that the energy difference of the nickel-bound radical specie stem from distinct benzoxazolyl acetate substrate coordination modes, leading to varied steric repulsion and consequently significant nickel catalyst distortion coupled with benzoxazolyl acetate substrate distortion.

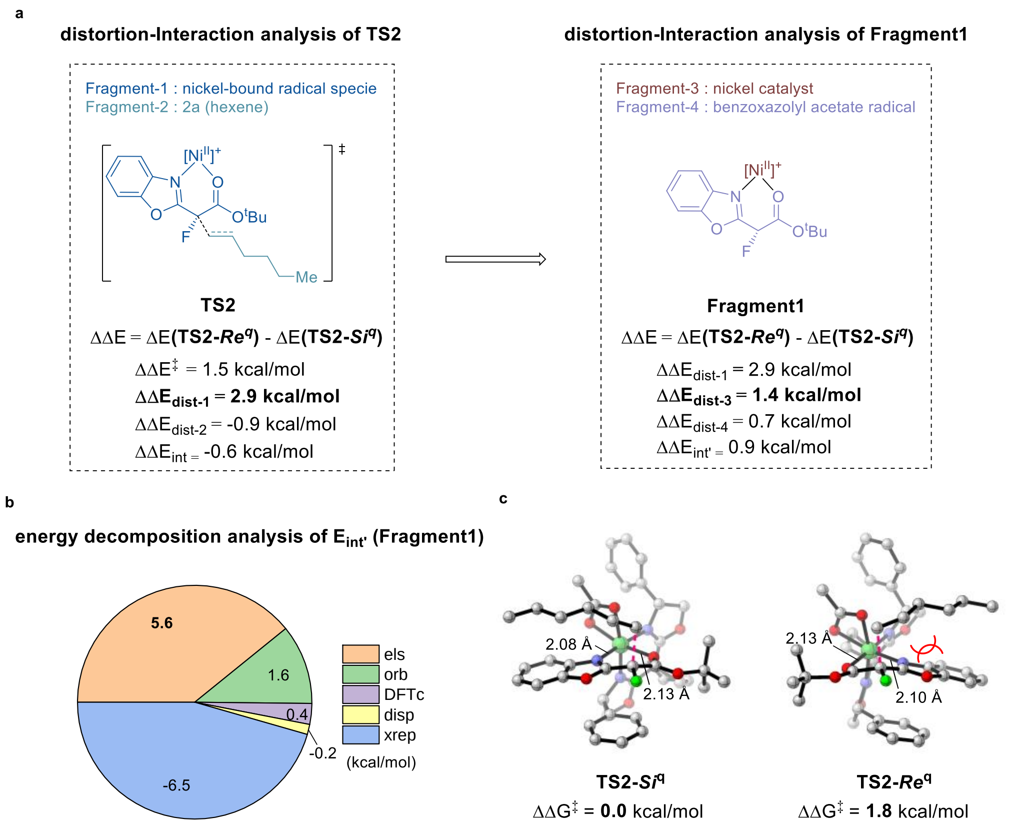

Figure S10. Analyses on the enantioselectivity-determining transition states **TS2-*Si*^{q}** and **TS2-*Re*^{q}** **a**, Distortion-interaction analysis. **b**, Energy decomposition analysis. **c**, Structural analysis. E_{dist}, distortion energy; E_{els}, electrostatic energy; E_{orb}, orbital interaction energy; E_{xrep}, exchange-repulsion energy; E_{DFTc}, correlation energy; E_{disp}, dispersion energy.

Since interaction energy ($E_{\mathrm{int'}}$ = 0.9 kcal/mol) contributes non-negligibly to the energy difference, we conducted additional energy decomposition analysis (**Figure S10**, **b**), which identified electrostatic interactions as the dominant component (5.6 kcal/mol), with orbital interactions playing a minor role (1.6 kcal/mol). Consistent with our earlier findings[20], the two nickel-bound radical specie exhibit a noticeable difference in N–Ni bond lengths (slightly longer in the **TS2-*Re*^{q}** complex, **TS2-*Si*^{q}**: 2.08Å; **TS2-*Re*^{q}**: 2.10Å), whereas the O–Ni distances remain similar (**TS2-*Si*^{q}**: 2.13Å; **TS2-*Re*^{q}**: 2.13Å). The coordination difference correlates well with the larger electrostatic energy difference and smaller orbital interaction energy difference. We thus attribute the interaction energy difference of the nickel-bound radical specie to the weaker N–Ni coordination in **TS2-*Re*^{q}**.

Structural analysis suggests that, under the **TS2-*Re*^{q}** coordination mode, steric repulsion occurs between the phenyl ring of the benzoxazolyl acetate substrate and the BOX ligand (**Figure S10**, **c**). This structural distinction consistently accounts for the greater distortion energy difference of the nickel catalyst and the smaller distortion energy difference of the benzoxazolyl acetate substrate in **TS2-*Re*^{q}**. Furthermore, it leads to impaired contact between the coordinating N atom (adjacent to the phenyl substituent) and the Ni center, thereby resulting in a weaker N–Ni bond, whereas the O–Ni bond remains unaffected.

Integrating the results from distortion-interaction analysis, energy decomposition analysis, and

structural analysis, we conclude that the enantioselectivity arises from energy differences induced by distinct substrate coordination modes. In particular, the coordination in **TS2-*Re***^{q} introduces steric repulsion between the phenyl substituent of the benzoxazolyl acetate substrate and the BOX ligand, resulting in a disfavored transition state.

Discussion of the radical termination process

In addition to the HAT mechanism, β-H elimination is also a possible radical termination pathway (**Figure S11**). However, DFT calculations revealed that the energy of the transition state of β-H elimination (**TS3-3a'**^{s}) is significantly higher than that of the HAT mechanism (**TS3-3a**$^{s(BS)}$), and thus the β-H elimination mechanism can be subsequently ruled out.

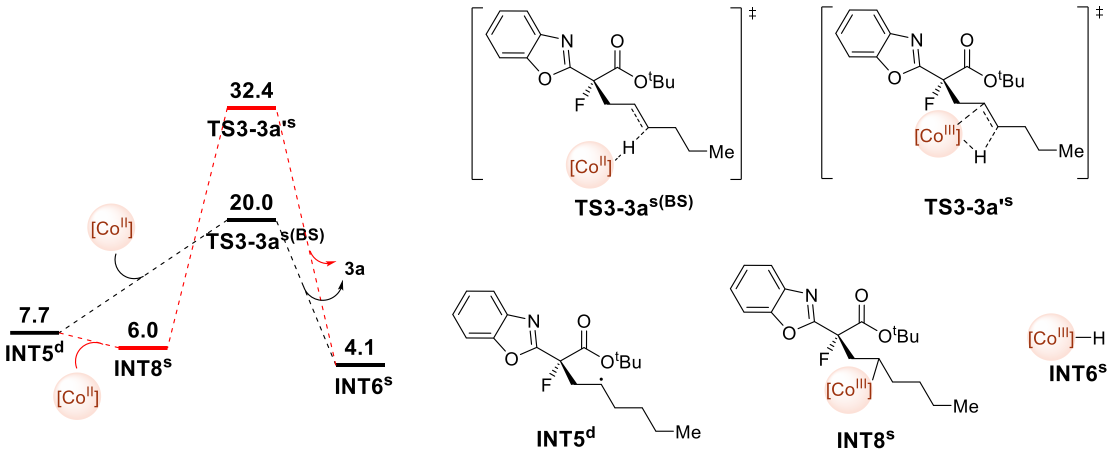

Figure S11. Gibbs free energy profile of possible radical termination mechanisms in kcal/mol. Black line: HAT mechanism; red line: β-H elimination mechanism.

Calculation of the bond dissociation energy (BDE)

Bond dissociation energy (BDE) were calculated using the following equation in gas phase in order to compare with the experiment value:

$$BDE(A-B) = H(A\cdot) + H(B\cdot) - H(A-B)$$

Table S11. Theoretical calculation results of bond dissociation energies.

Species	Bond	BDE/(kcal/mol)
**$(MeO)_3$Si-H**	Si–H	96.3
**A2-H**	Co–H	35.4
**A3-H**	Co–H	35.2
**A4-H**	Co–H	35.5
**A5-H**	Co–H	36.3

Discussion on chemoselectivity of the alkenylation reaction

Our investigation reveals that the alkenylation pathway, mediated by the **A1** mediator, proceeds through an oxidation–radical addition–elimination mechanism. Following radical addition, the resulting alkyl radical is oxidized to a carbocation intermediate, which is stabilized by 2,6-lutidine[31]. Subsequent base-promoted elimination then produces the alkenylation product (**Figure S12**).

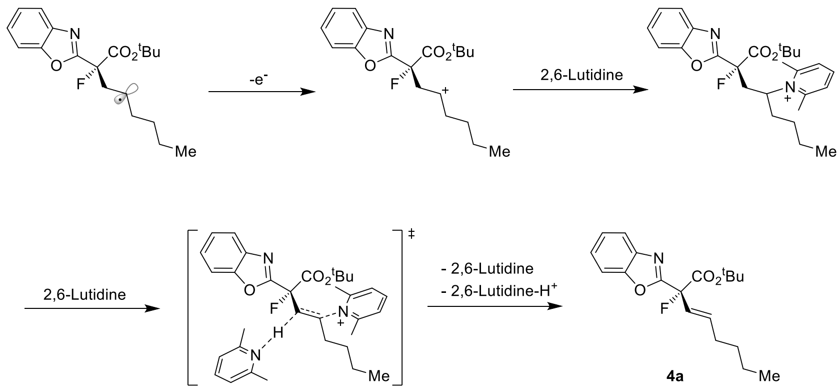

Figure S12. Plausible reaction mechanism of the alkenylation reaction.

To elucidate the chemoselectivity, we computed the key transition states leading to both alkenylation and allylation products. In alignment with the optimized experimental conditions, we employed DCE:TFE = 1:5 as solvent for the alkenylation reaction (Only TS5, Eps = 23.959, EpsInf = 1.736, HbondAcidity = 0.492, HbondBasicity = 0.227, SurfaceTensionAtInterface = 42.66, CarbonAromaticity = 0.0 and ElectronegativeHalogenicity = 0.5). DFT results clearly indicate a pronounced energetic preference for the alkenylation pathway (**TS5-4a^s, Figure S13**). This selectivity can be attributed to the enhanced acidity of the hydrogen atom situated near the three electron-withdrawing groups—the benzoxazole, ester, and fluorine substituents—which facilitates its elimination and directs the reaction toward alkenylated product **3a**.

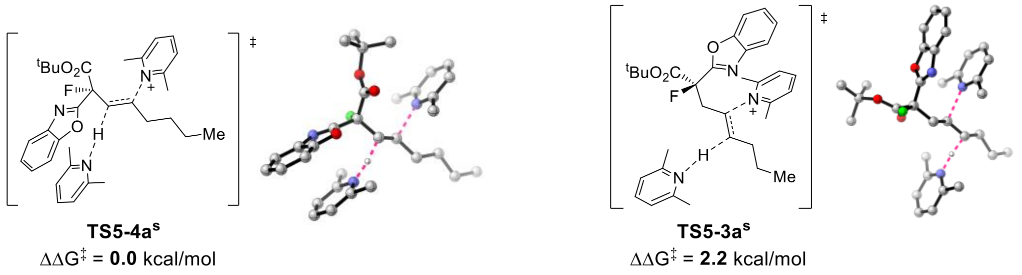

Figure S13. Structures and relative Gibbs free energies of the key transition states in the alkenylation reaction.

The use of a Co catalysts leads to allylation, due to steric hindrance influencing HAT, where the cobalt catalyst selectively abstracts the less hindered distal hydrogen atom, leading to the observed allylation product **4a** (**Fig. 7c**). These computational insights now provide a quantitative basis for the divergence between the two pathways: the alkenylation route is governed by the relative acidity of specific C–H bonds and their propensity for elimination, while the allylation pathway is controlled by sterically modulated hydrogen atom transfer.

Discussion on Z/E selectivity of the allylation reaction and the alkenylation reaction

To elucidate the origin of the stereochemical outcome, we computed the key transition states governing the formation of *E*- and *Z*-alkenes in both the allylation and alkenylation pathways (**Figure S14**). For the allylation reaction, the energy difference between transition states **TS3-3a**$^{s(BS)}$ (*E*-pathway) and **TS3-3a-*Z***$^{s(BS)}$ (*Z*-pathway) is relatively small ($\Delta\Delta G^{\ddagger}$ = 0.4 kcal/mol), corresponding to an E/Z ratio of approximately 6:1, which aligns well with our experimental observations. In contrast, for the alkenylation pathway, a significantly larger energy difference ($\Delta\Delta G^{\ddagger}$ = 4.6 kcal/mol) is observed between **TS5-4a**^{s} (*E*-pathway) and **TS5-4a-*Z***^{s} (*Z*-pathway). This substantial barrier rationalizes why the *Z*-isomer was not detected experimentally in the alkenylation products. Structurally, the bulky 2,6-lutidine base introduces considerable steric hindrance that disproportionately destabilizes the transition state leading to the *Z*-alkene, particularly in the alkenylation mechanism where the geometry around the forming double bond is more constrained. In comparison, the more planar and open coordination environment of the Co(salen) catalyst in the allylation pathway results in a lower energetic penalty for *Z*-isomer formation.

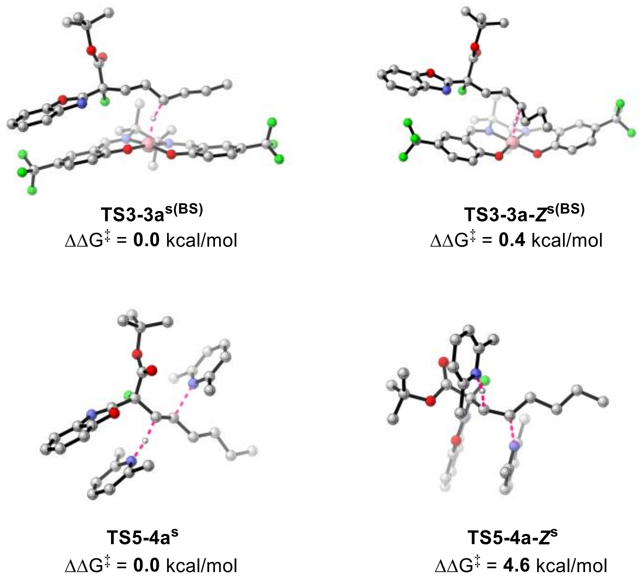

Figure S14. Structures and relative Gibbs free energies of the key transition states leading to the *Z*/*E* products in the allylation reaction and the alkenylation reaction.

Table of energies

Table S12. Thermal correction to Gibbs free energies (TCG), Single-point energies in THF:TFE = 1:5 solution (E_{sol}), and Gibbs free energies (G) of the allylation reaction calculations in Hartree.

Entry	TCG	E_{sol}	G
**[Co^{II}]^{d}**	0.340346	-3092.535024	-3092.191666
**[Ni^{II}]$^{+}$(OAc^{-})^{t}**	0.474117	-3116.467030	-3115.989901
**A1**	0.232448	-1824.165086	-1823.929626
**A1^{+}**	0.233170	-1824.000054	-1823.763872
**1a**	0.211474	-884.163400	-883.948914
**2a**	0.134916	-235.862750	-235.724822
**3a**	0.349152	-1118.838061	-1118.485897
**INT1^{t}**	0.671185	-3771.983577	-3771.309380
**INT2^{t}**	0.656021	-3771.516285	-3770.857252
**INT2-1b^{t}**	0.692100	-3711.593177	-3710.898065
**INT3^{q}**	0.657803	-3771.342454	-3770.681639
**INT3-1b^{q}**	0.693572	-3711.423960	-3710.727376
**INT3-1^{q}**	0.657075	-3771.340078	-3770.679991
**INT3-2^{q}**	0.657216	-3771.337758	-3770.677530
**INT3-3^{q}**	0.657065	-3771.334626	-3770.674549
**INT4^{q}**	0.819383	-4007.233152	-4006.410757
**INT5^{d}**	0.359330	-1119.404262	-1119.041920
**INT6^{s}**	0.348171	-3093.104514	-3092.753331
**INT7^{s}**	0.480325	-3380.945272	-3380.461935
**INT8^{s}**	0.734867	-4211.974077	-4211.236198
**morpholine**	0.107820	-287.829453	-287.718622
**morpholine^{+}**	0.106608	-287.628420	-287.518800
**morpholine-H^{+}**	0.122695	-288.298548	-288.172841
**morpholine-HOAc**	0.163438	-516.963793	-516.797343
**TS1^{t}**	0.798189	-4059.819460	-4059.018259
**TS2-*Re*^{q}**	0.818636	-4007.206738	-4006.385090
**TS2-*Re*'^{q}**	0.816697	-4007.194975	-4006.375266
**TS2-*Si*^{q}**	0.817620	-4007.208656	-4006.388025
**TS3-3a$^{s(BS)}$**	0.725253	-4211.942097	-4211.213832
**TS-3a-*Z*$^{s(BS)}$**	0.723293	-4211.939432	-4211.213127
**TS3-3a'^{s}**	0.727266	-4211.924498	-4211.194220
**TS3-4a$^{s(BS)}$**	0.724202	-4211.937521	-4211.210307
**TS4^{s}**	0.477951	-3380.939914	-3380.458951

Table S13. Thermal correction to Gibbs free energies (TCG), Single-point energies in DCE:TFE = 1:5 solution (E_{sol}), and Gibbs free energies (G) of the alkenylation reaction calculations in Hartree.

Entry	TCG	E_{sol}	G
**TS5-3a^{s}**	0.635898	-1773.146007	-1772.507097
**TS5-4a^{s}**	0.637103	-1773.150658	-1772.510543
**TS5-4a-*Z*^{s}**	0.638400	-1773.144658	-1772.503246

Table S14. Thermal correction to Gibbs free energies (TCG), Single-point energies in THF:TFE = 1:5 solution (E_{sol}) of spin state validation in Hartree.

Entry	TCG	E_{sol}(TPSSh-D3(BJ) Single-Point)	E_{sol}(MN15L Single-Point)
Low-energy spin states			
**$[Ni^{II}]^{+}(OAc^{-})^{t}$**	0.474117	-3117.138433	-3115.459267
**INT1^{t}**	0.671185	-3772.932375	-3770.626833
**INT2^{t}**	0.656021	-3772.468108	-3770.170091
**INT3^{q}**	0.657803	-3772.300924	-3770.004133
**INT4^{q}**	0.819383	-4008.289147	-4005.704405
**$[Co^{II}]^{d}$**	0.340346	-3093.195675	-3091.528091
**INT6^{s}**	0.348171	-3093.769786	-3092.095372
**$([Co^{I}]^{-})^{s}$**	0.334799	-3093.290458	-3091.628297
High-energy spin states			
**$[Ni^{II}]^{+}(OAc^{-})^{s}$**	0.474742	-3117.114697	-3115.425146
**INT1^{s}**	0.671776	-3772.910499	-3770.594848
**INT2^{s}**	0.656636	-3772.444379	-3770.135346
**INT3$^{d(BS)}$**	0.658208	-3772.299705	-3770.001234
**INT4$^{d(BS)}$**	0.820010	-4008.289136	-4005.704370
**$[Co^{II}]^{q}$**	0.335094	-3093.179432	-3091.522114
**INT6^{t}**	0.342865	-3093.736049	-3092.065149
**$([Co^{I}]^{-})^{t}$**	0.330842	-3093.275357	-3091.620990

Table S15. Thermal correction to enthalpy (TCH), Single-point energies in gas phase (E_{gas}), and enthalpy (H) of BDE calculations in Hartree.

Entry	TCH	E_{gas}	H
**H·**	0.002360	-0.502666	-0.500306
**$(MeO)_3Si$-H**	0.149960	-635.654063	-635.504103
**$(MeO)_3Si$·**	0.140392	-634.990628	-634.850236
**A2-H**	0.517038	-2726.134199	-2725.617162
**A2**	0.508496	-2725.568927	-2725.060432
**A3-H**	0.459946	-3416.716940	-3416.256994
**A3**	0.451427	-3416.152062	-3415.700636
**A4-H**	0.459731	-7644.725135	-7644.265404
**A4**	0.451279	-7644.159748	-7643.708469
**A5-H**	0.434497	-3093.068061	-3092.633564
**A5**	0.426471	-3092.501886	-3092.075415

11. NMR spectra

^{1}**H NMR** spectrum of **3a** (The product was isolated as a 6:1 mixture of E/Z isomers)

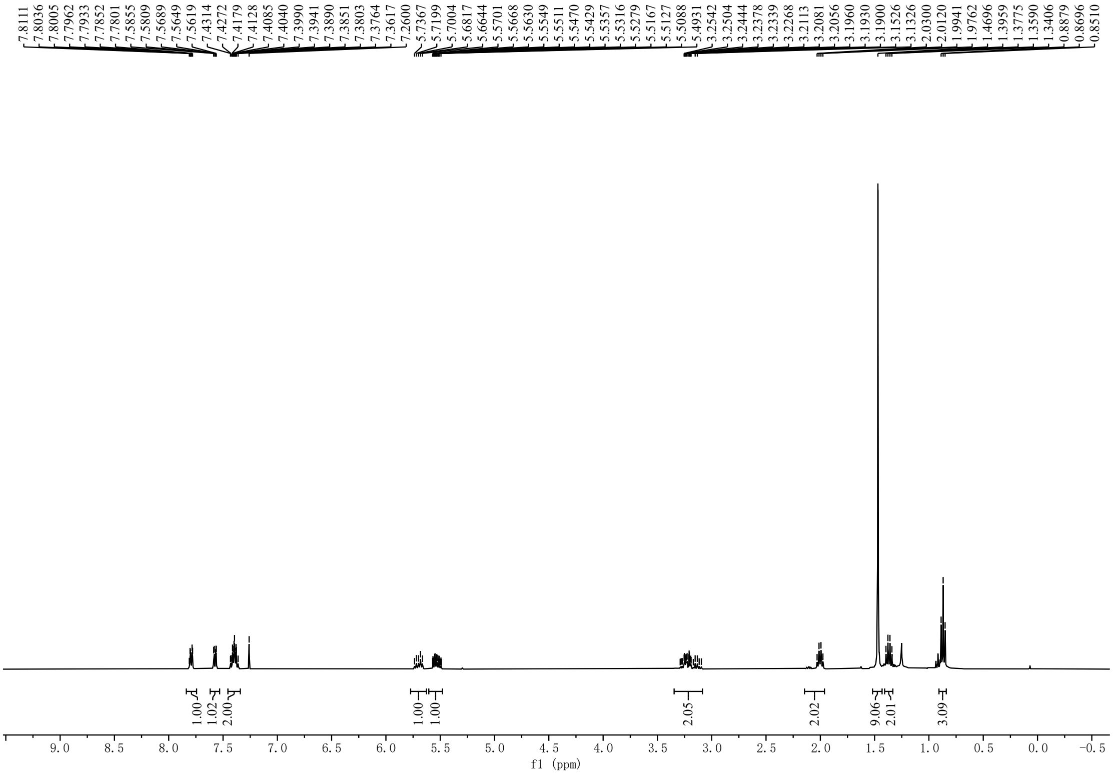

^{13}**C NMR** spectrum of **3a**

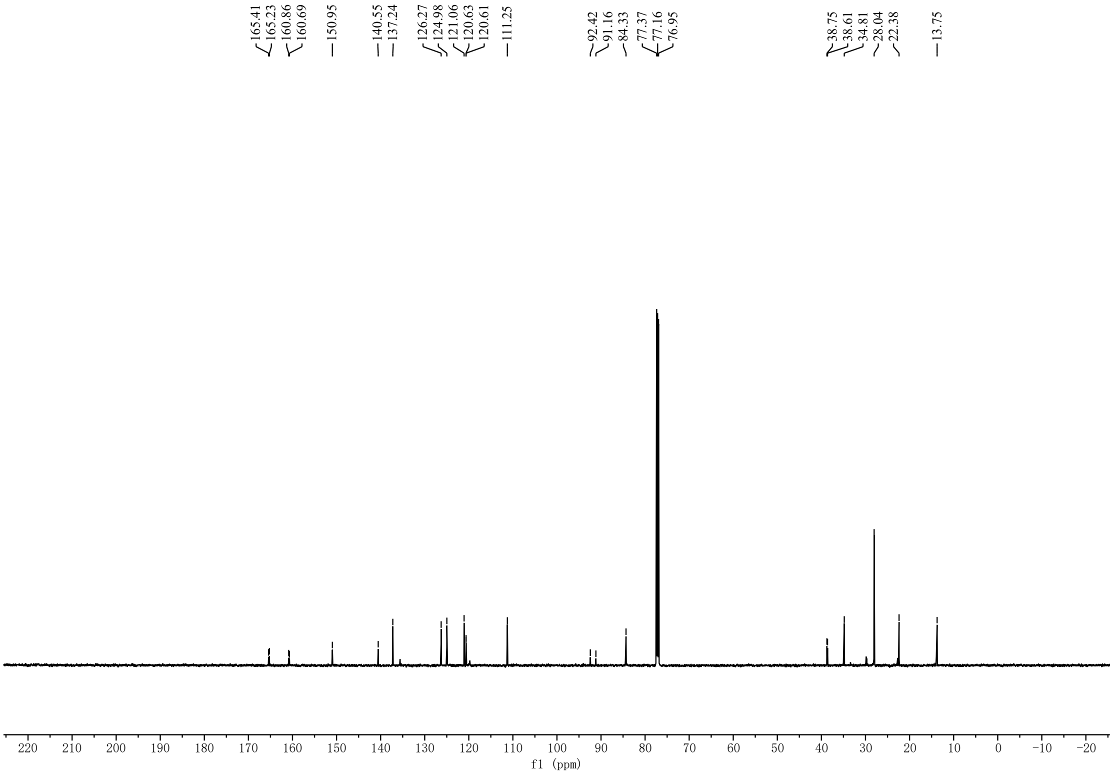

^{19}F NMR spectrum of **3a**

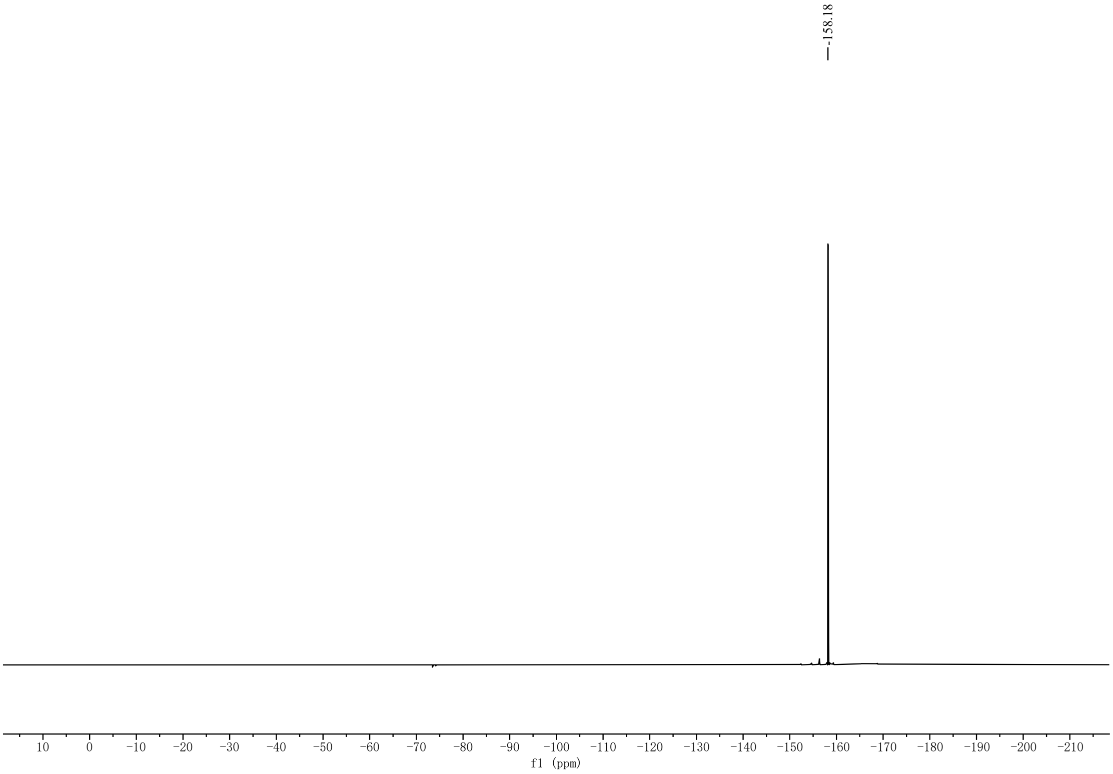

^{1}H NMR spectrum of **3b** (The product was isolated as a 6:1 mixture of E/Z isomers)

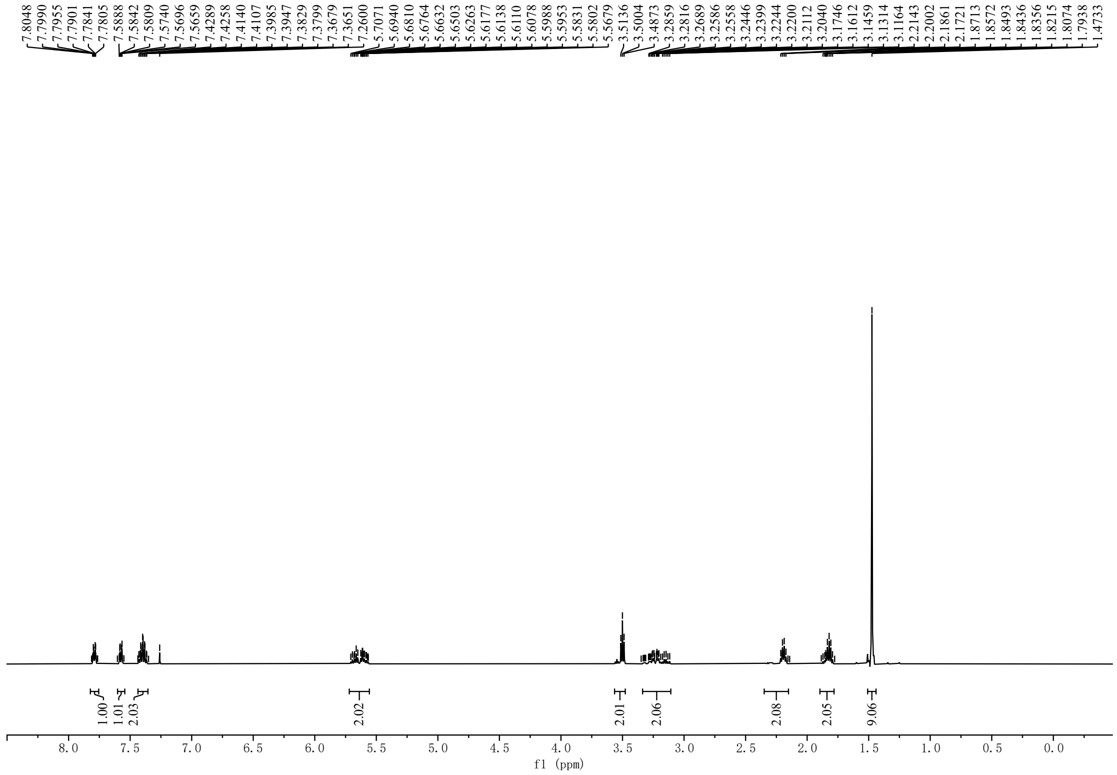

^{13}C NMR spectrum of **3b**

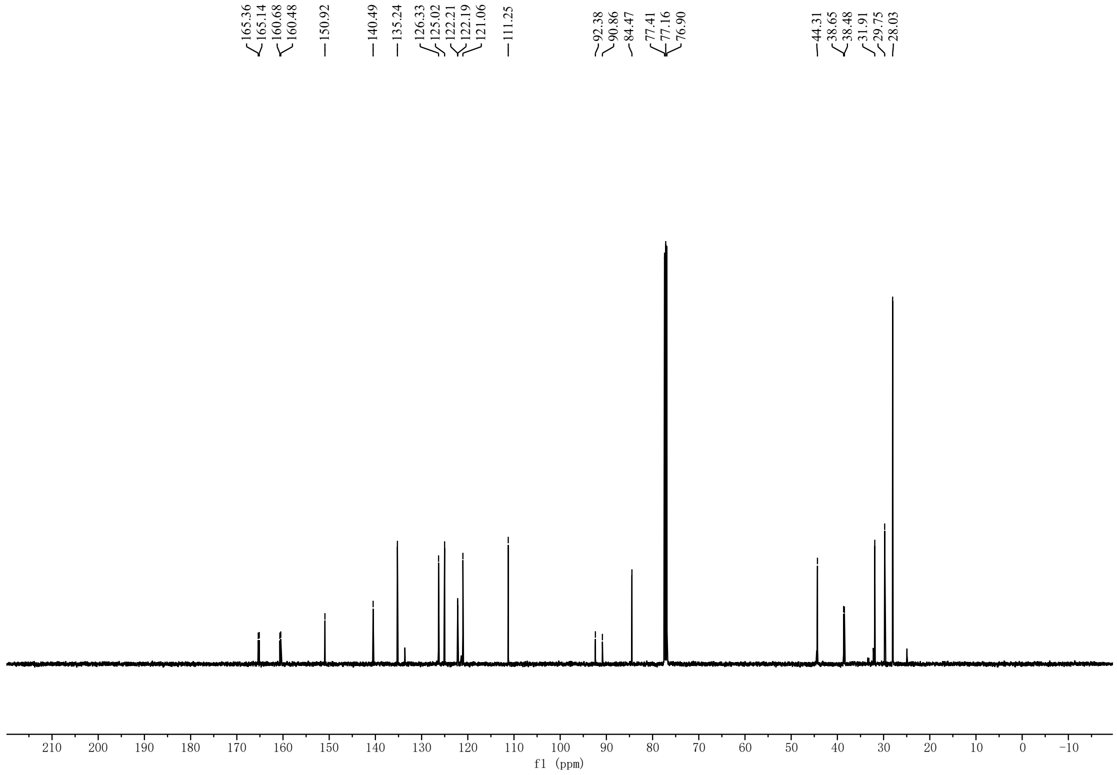

^{19}F NMR spectrum of **3b**

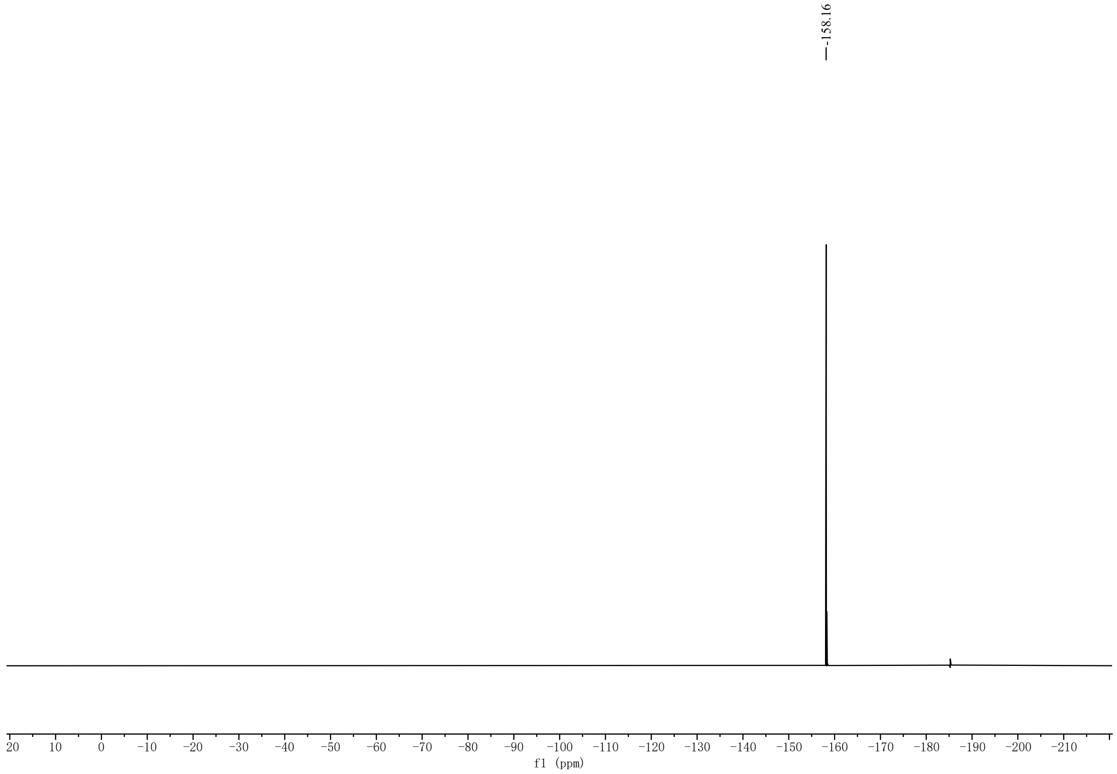

^{1}H NMR spectrum of **3c** (The product was isolated as a 6:1 mixture of E/Z isomers)

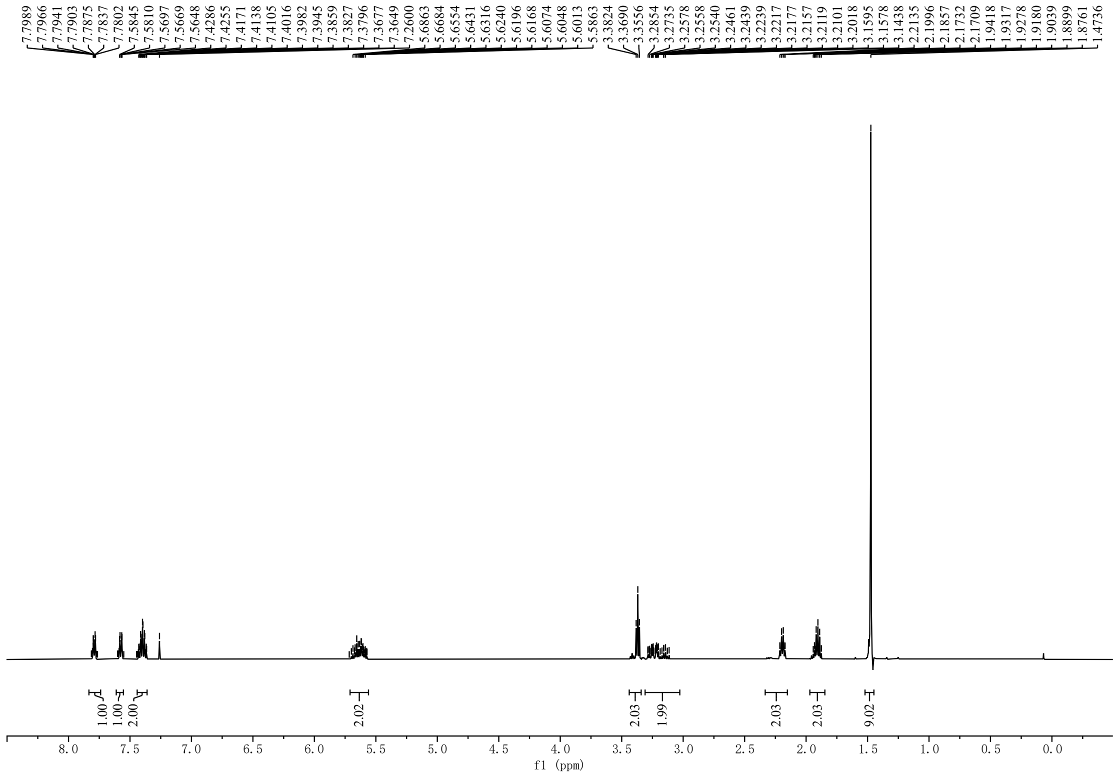

^{13}C NMR spectrum of **3c**

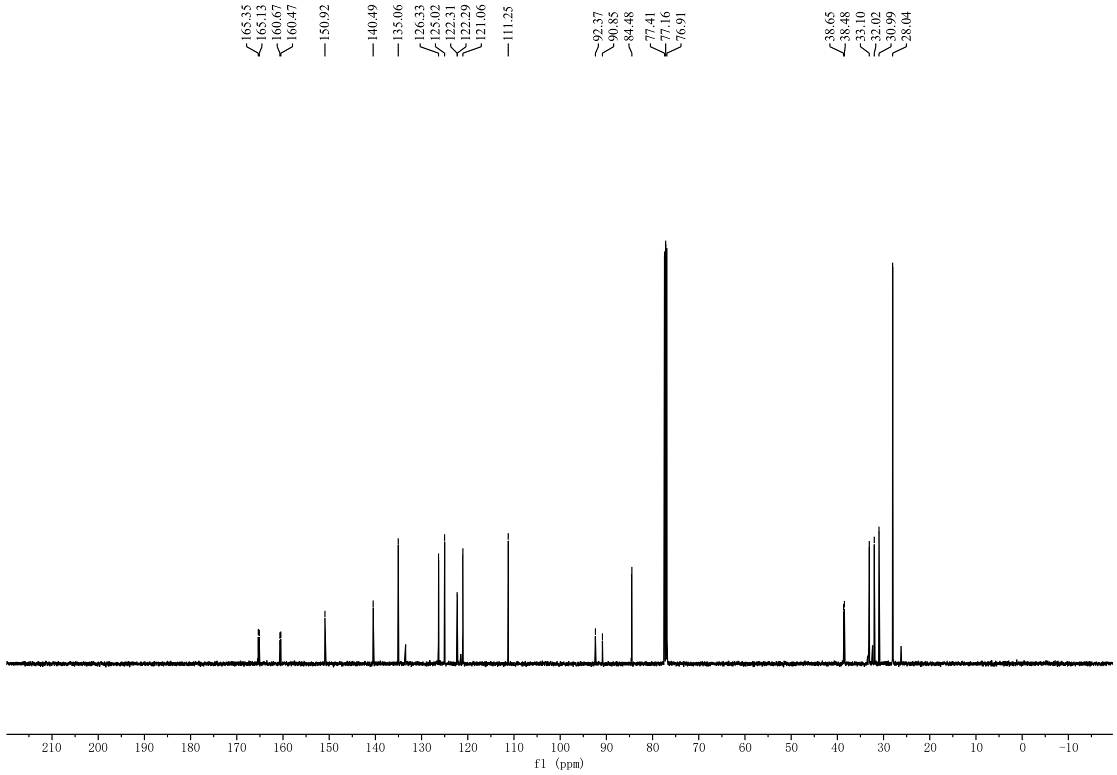

^{19}F NMR spectrum of **3c**

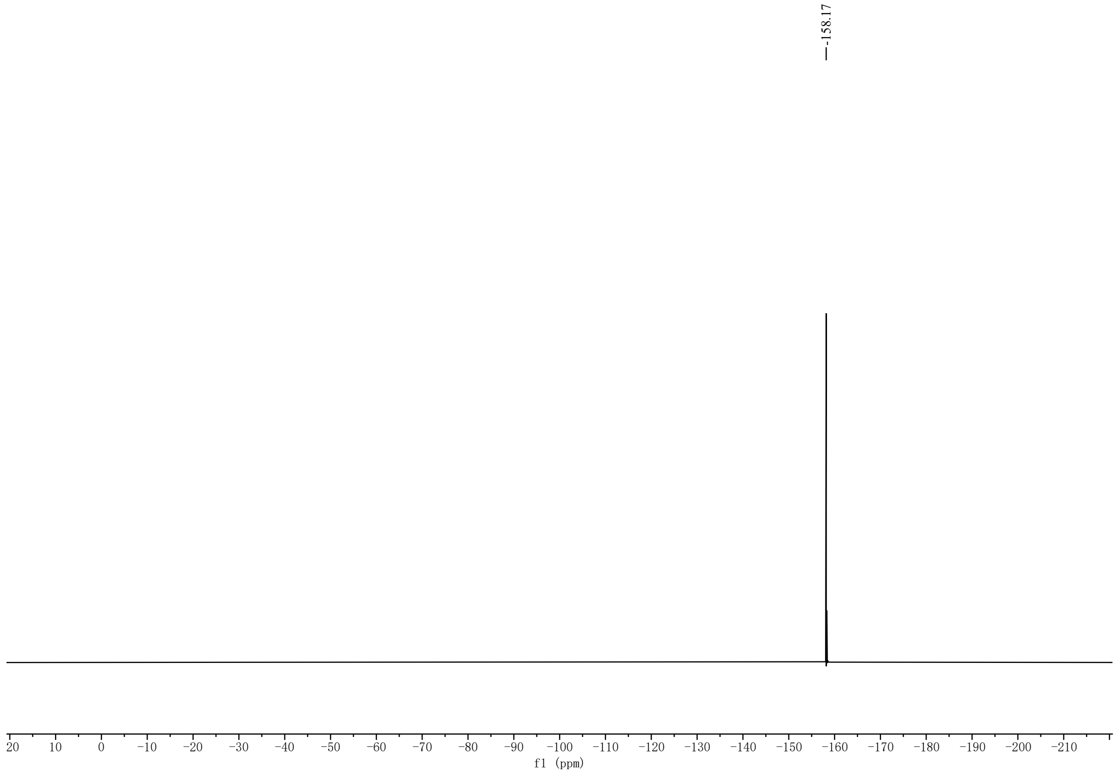

^{1}H NMR spectrum of 3d (The product was isolated as a 8:1 mixture of E/Z isomers)

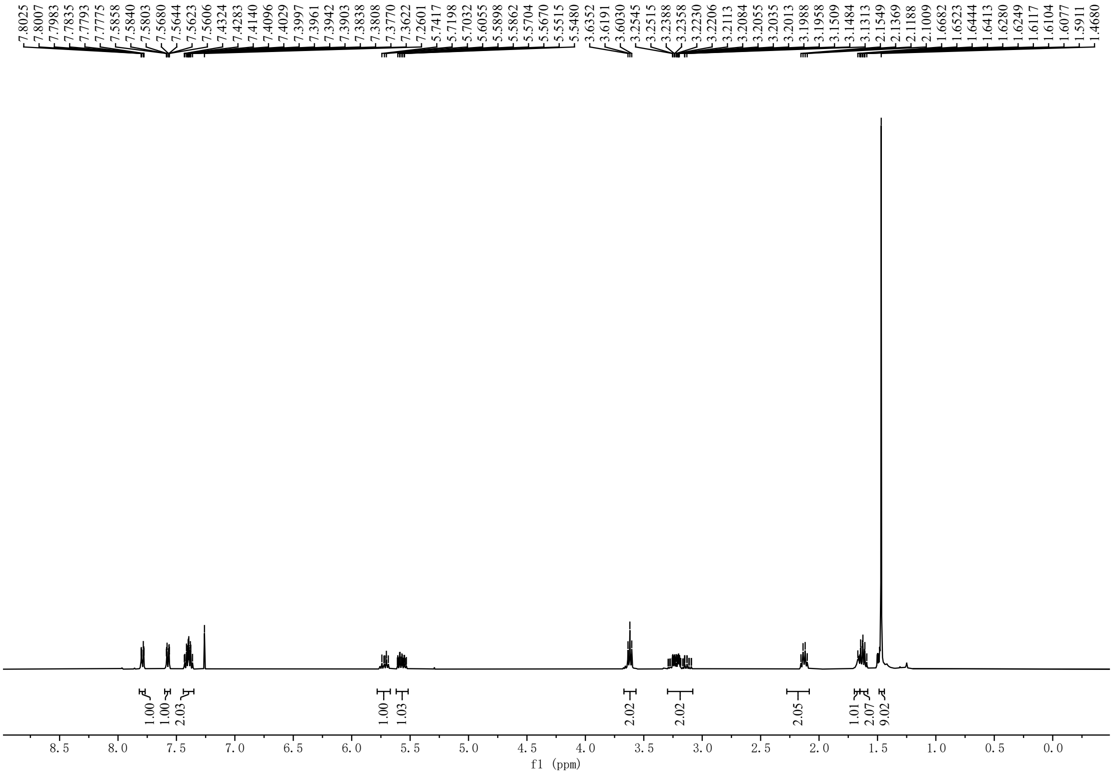

^{13}C NMR spectrum of 3d

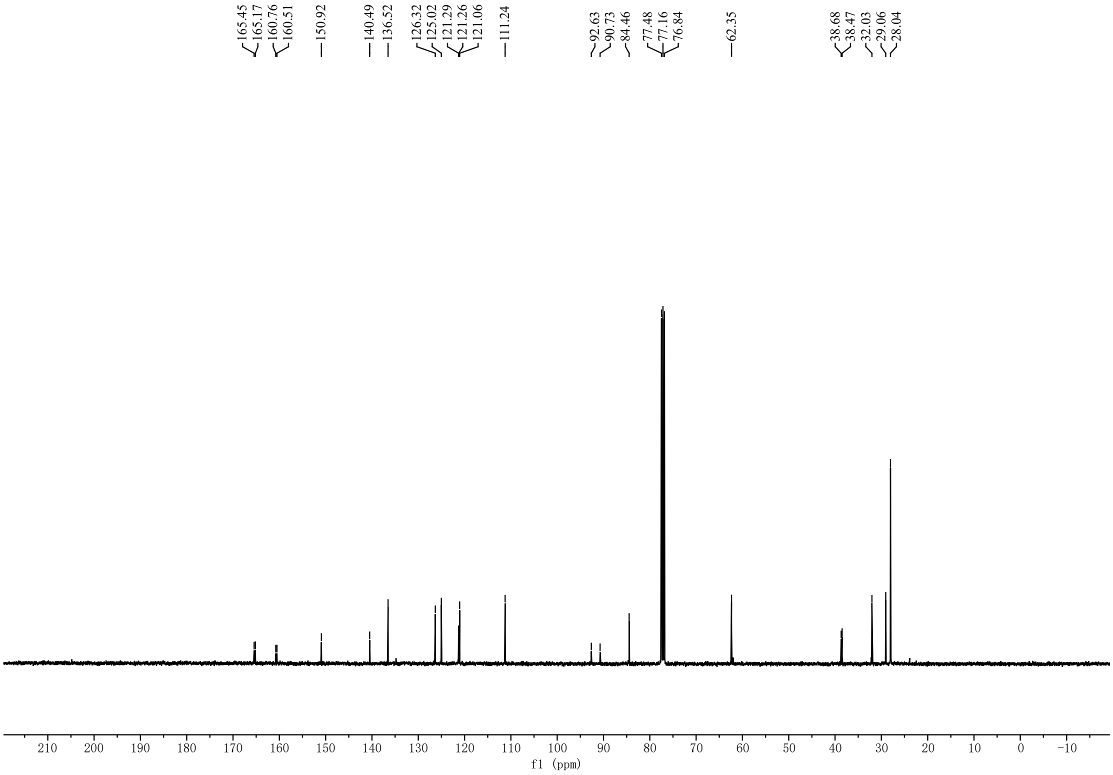

^{19}F NMR spectrum of **3d**

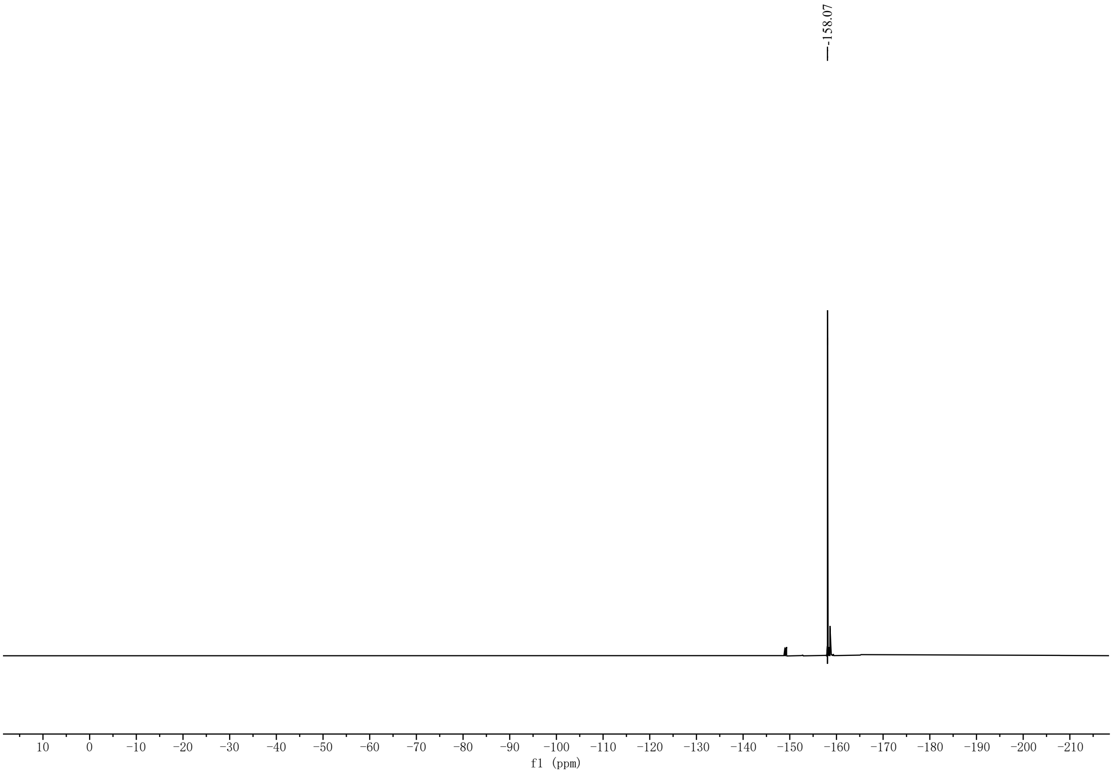

^{1}H NMR spectrum of **3e** (The product was isolated as a 5:1 mixture of E/Z isomers)

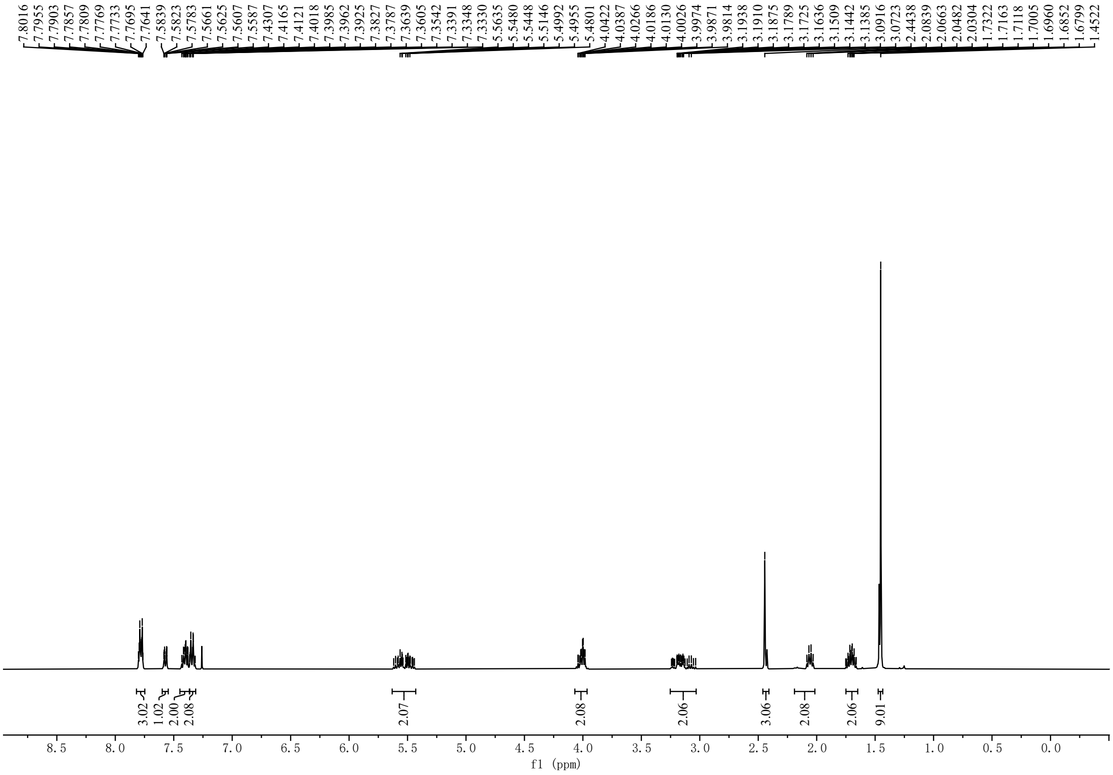

^{13}C NMR spectrum of **3e**

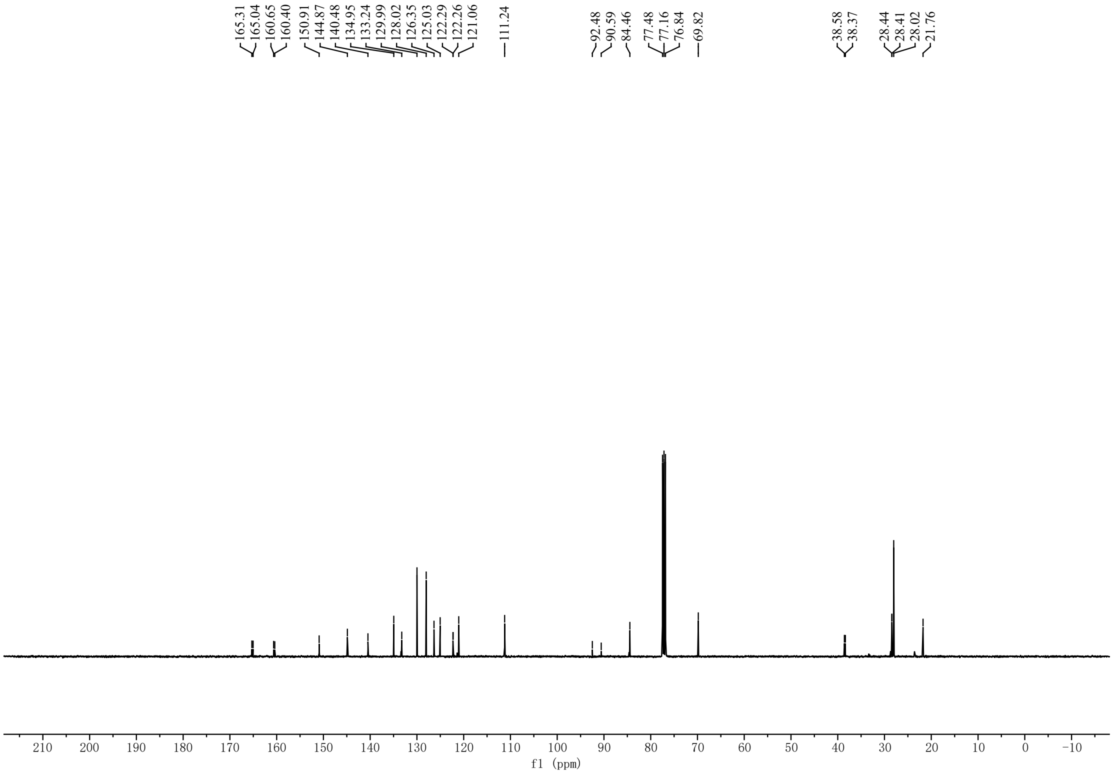

^{19}F NMR spectrum of **3e**

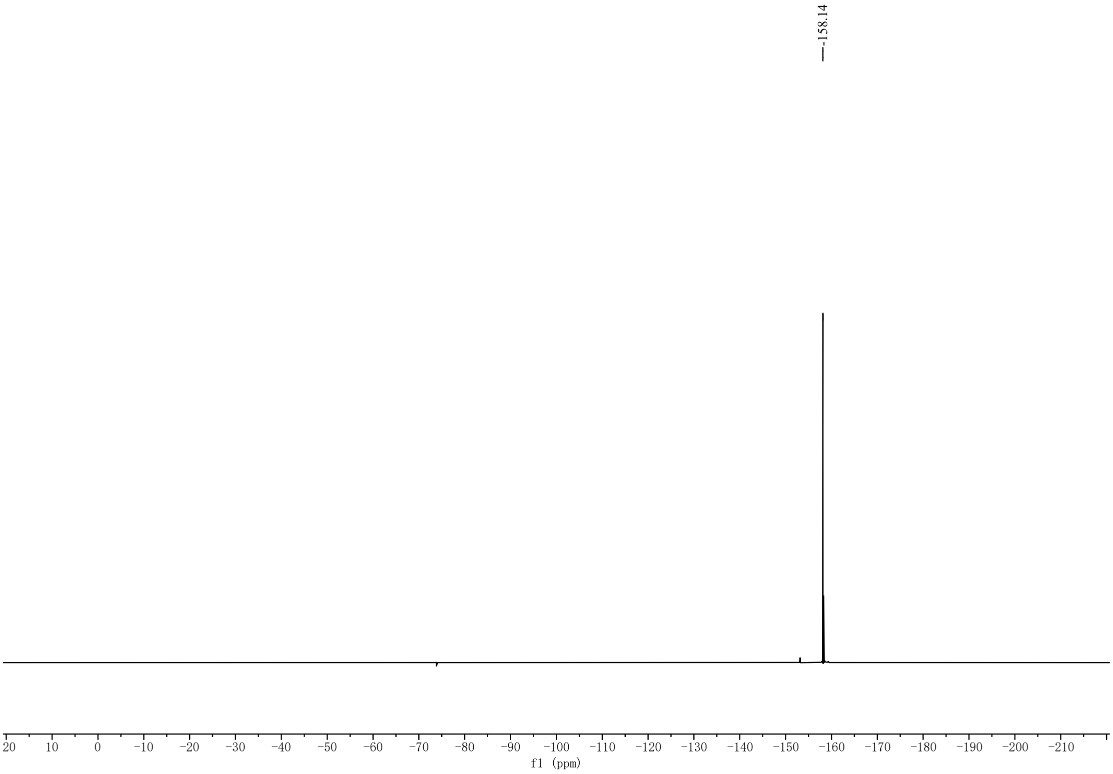

[1]**H NMR** spectrum of **3f** (The product was isolated as a 9:1 mixture of E/Z isomers)

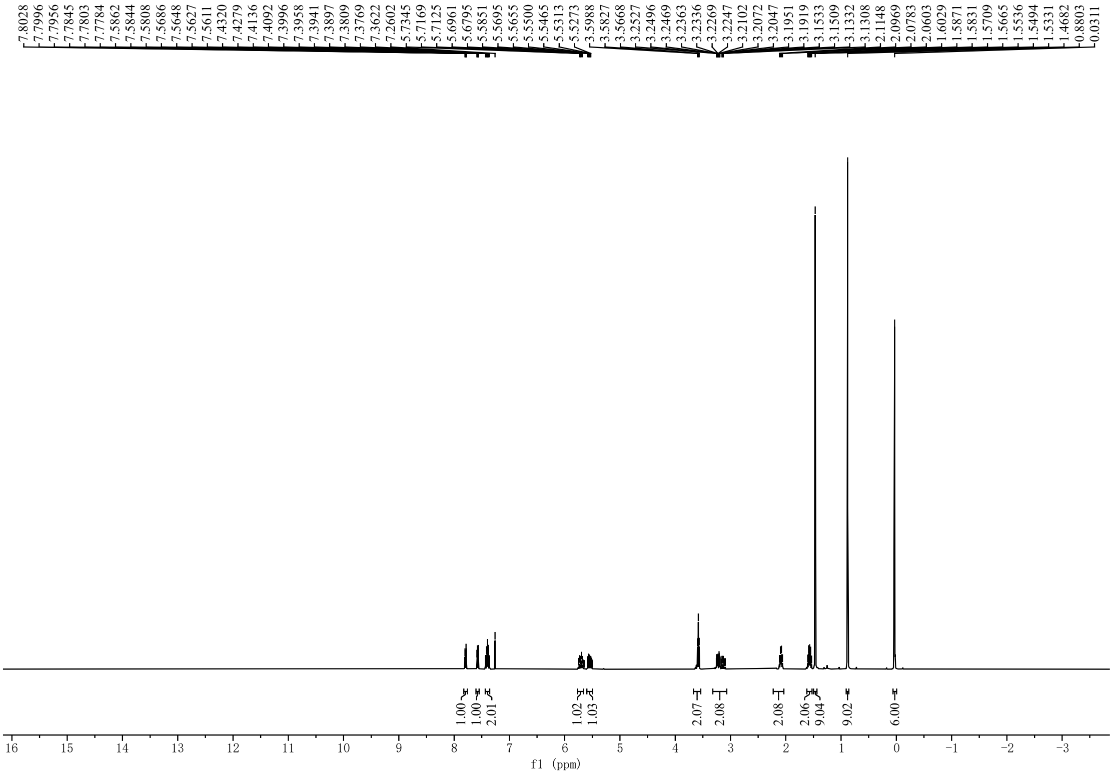

[13]**C NMR** spectrum of **3f**

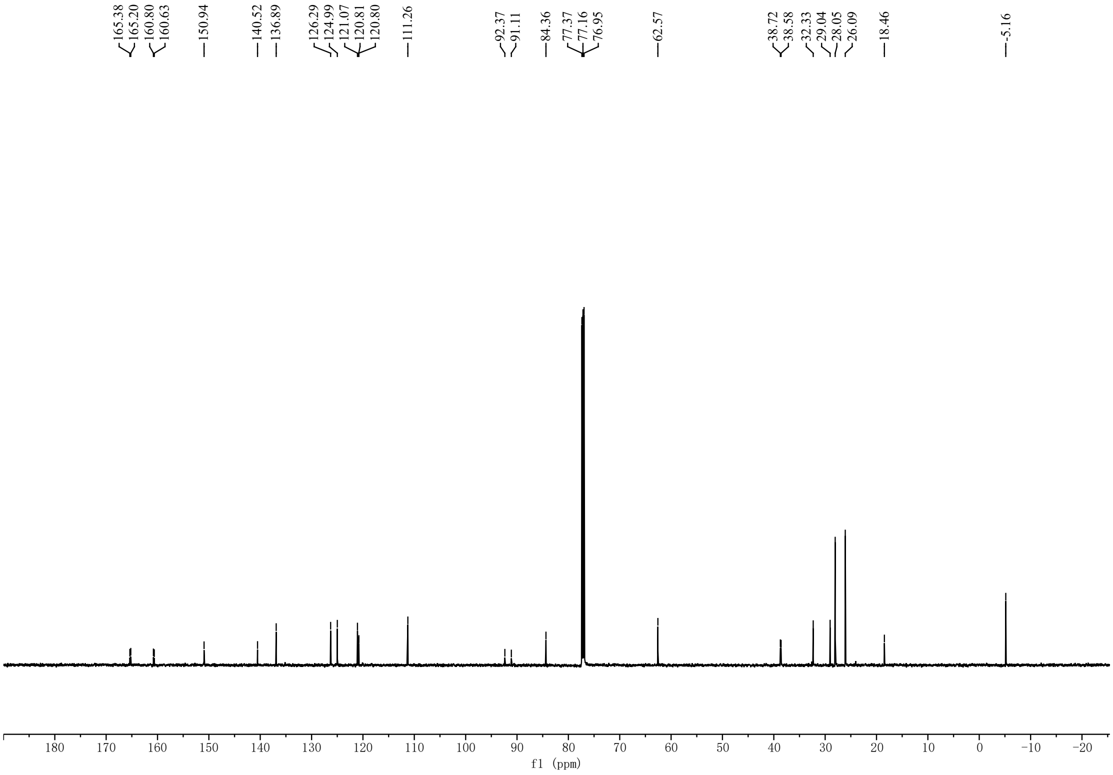

^{19}**F NMR** spectrum of **3f**

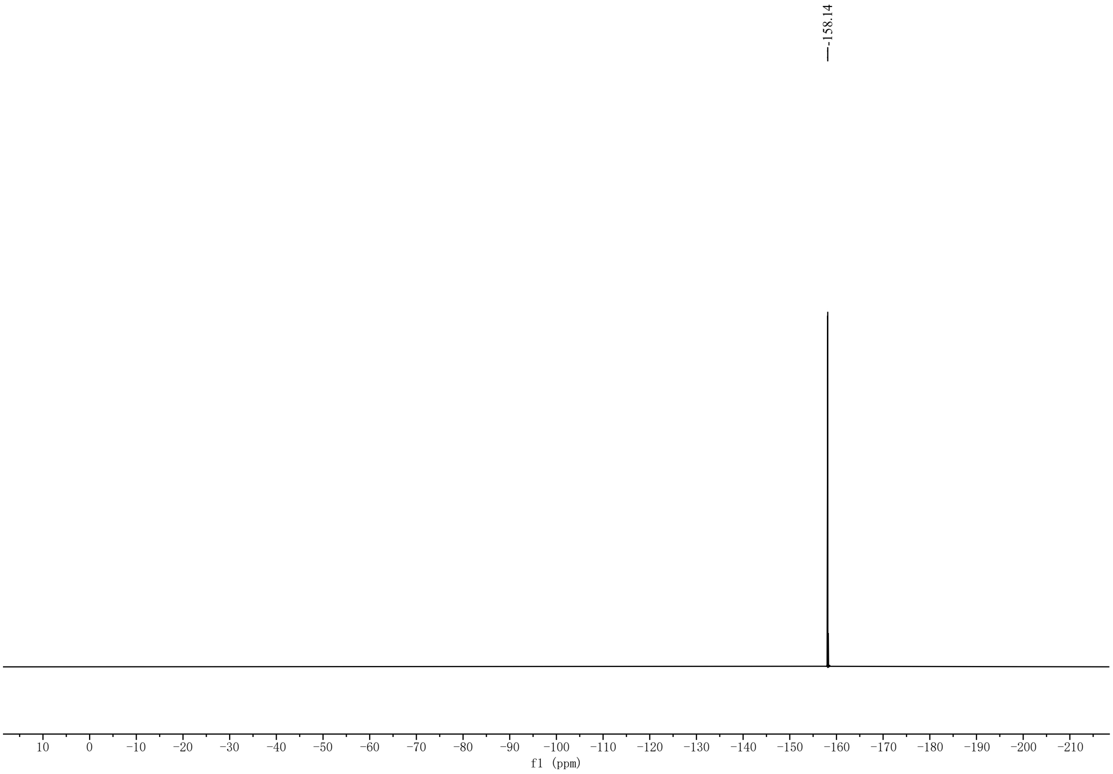

^{1}H NMR spectrum of **3g** (The product was isolated as a 5:1 mixture of E/Z isomers)

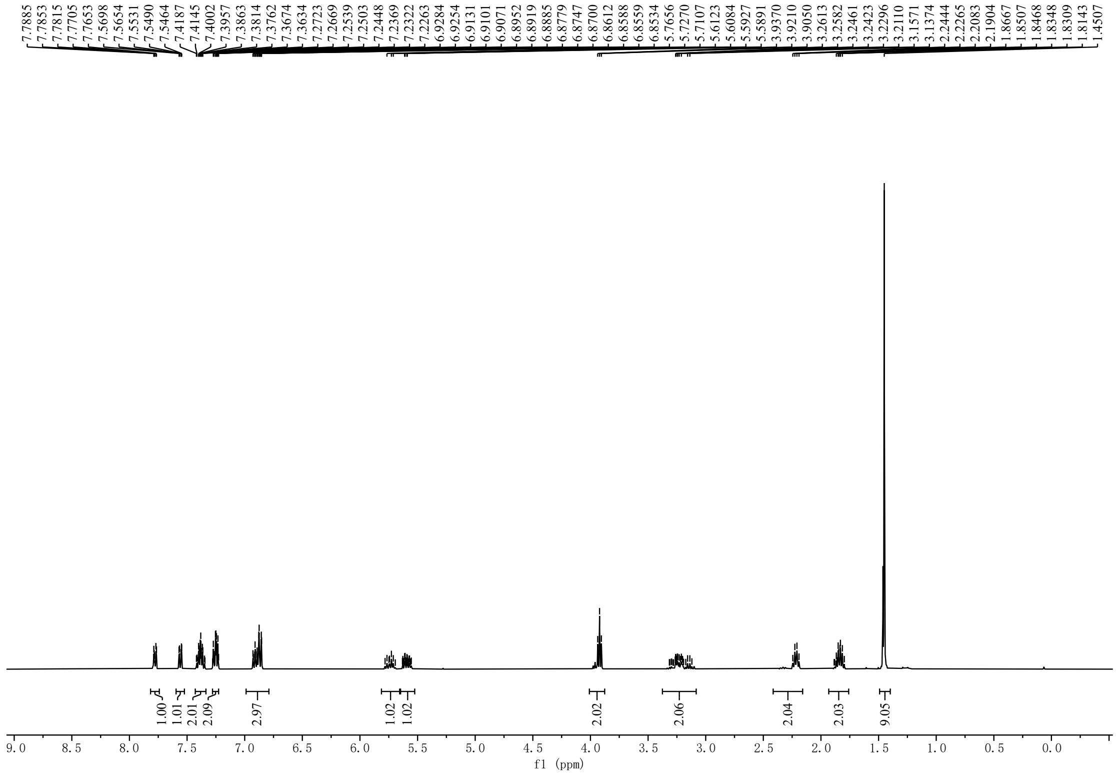

^{13}C NMR spectrum of **3g**

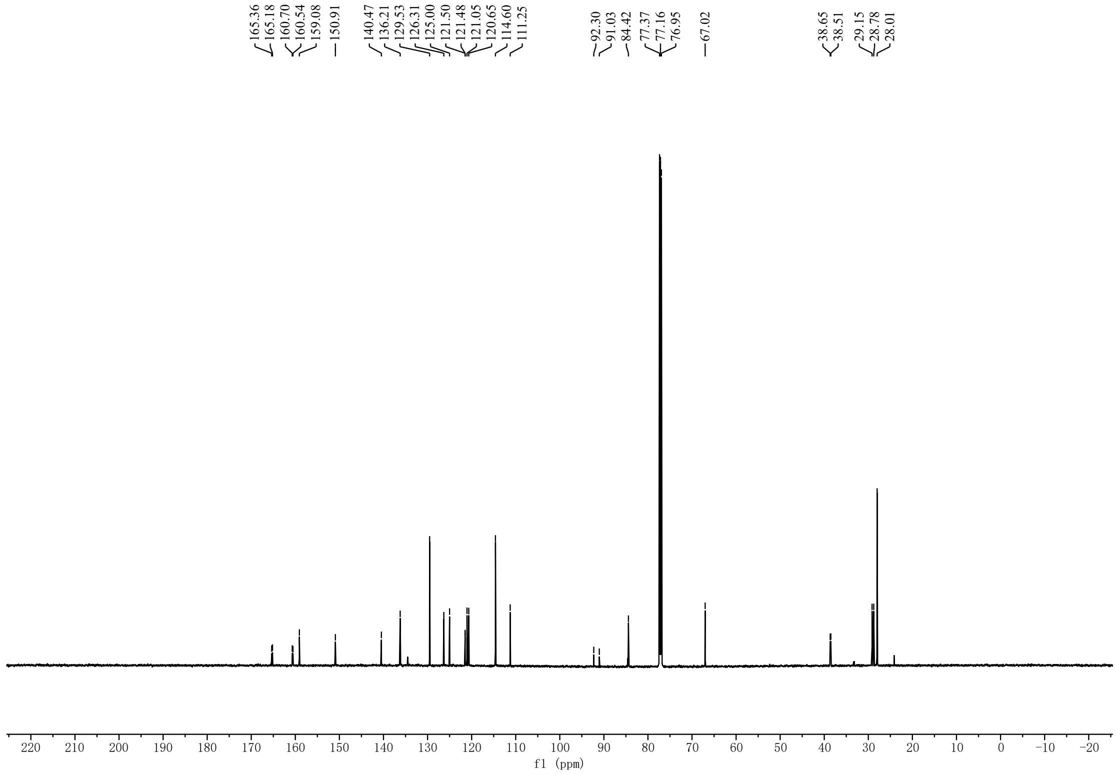

^{19}F NMR spectrum of **3g**

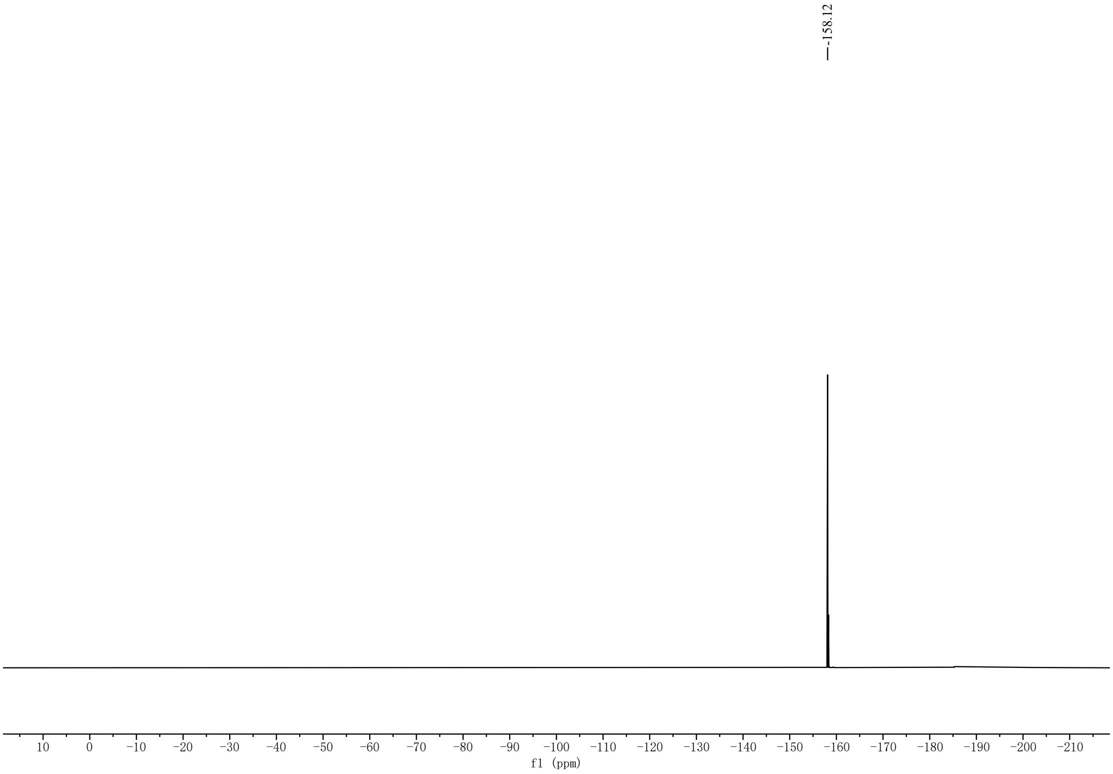

^{1}H NMR spectrum of **3h**

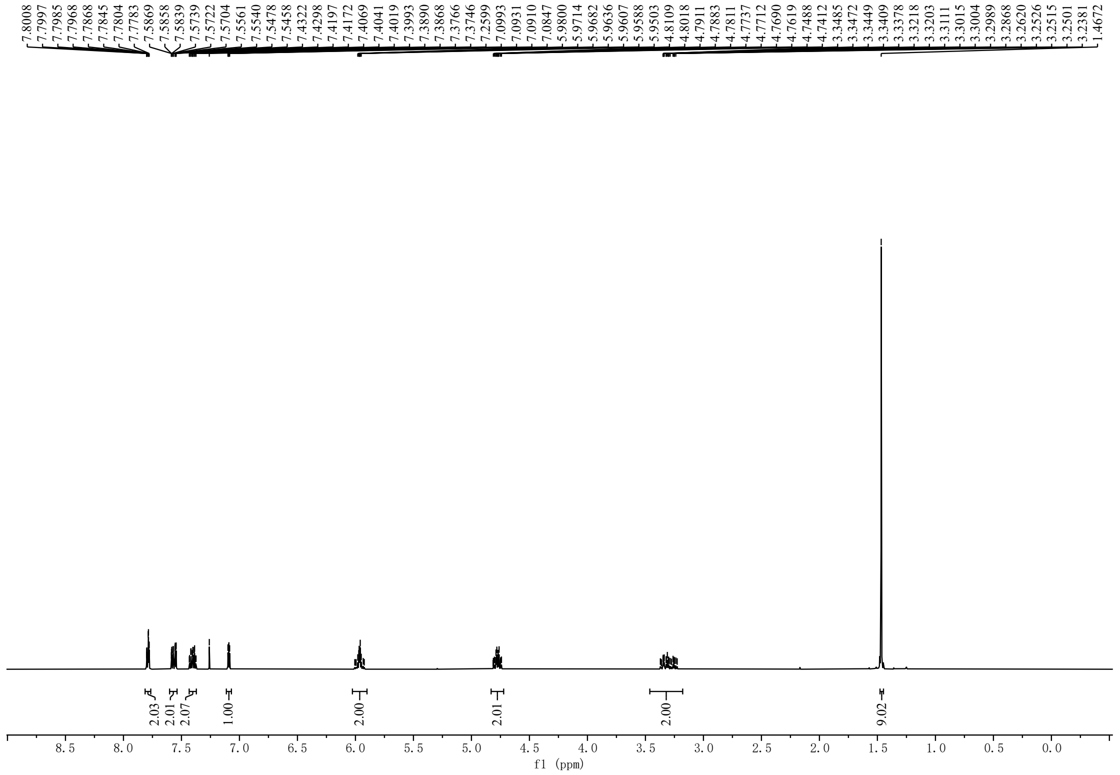

^{13}C NMR spectrum of **3h**

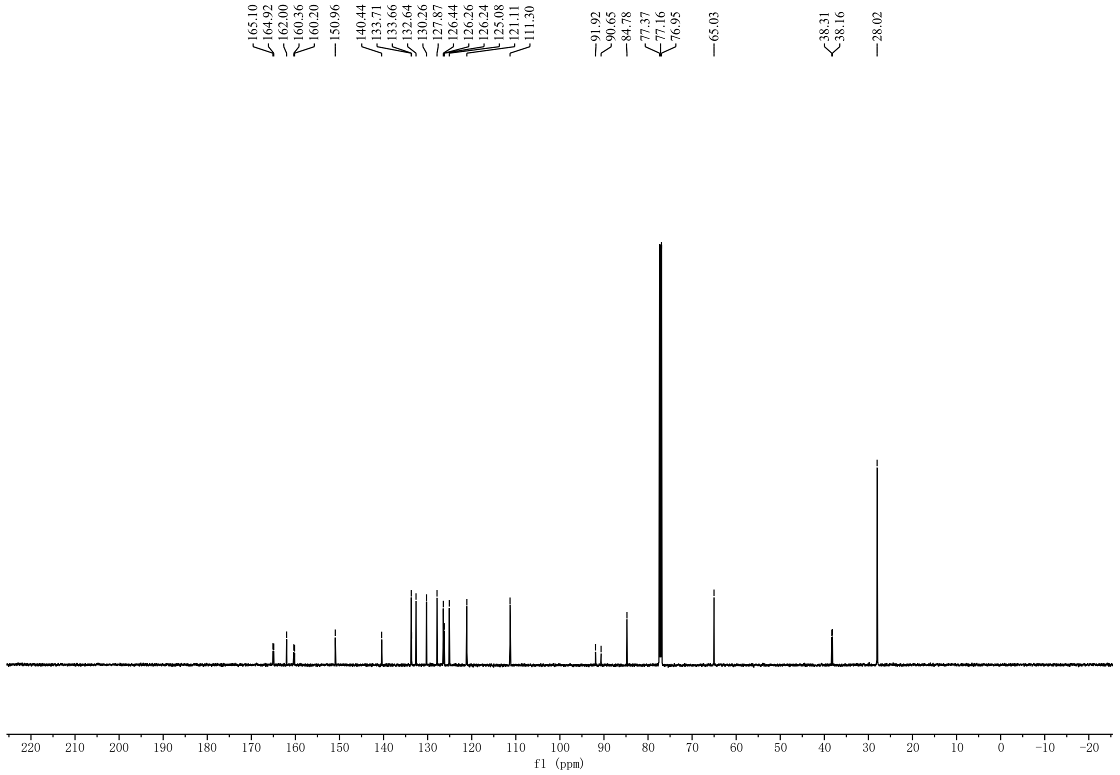

^{19}F NMR spectrum of **3h**

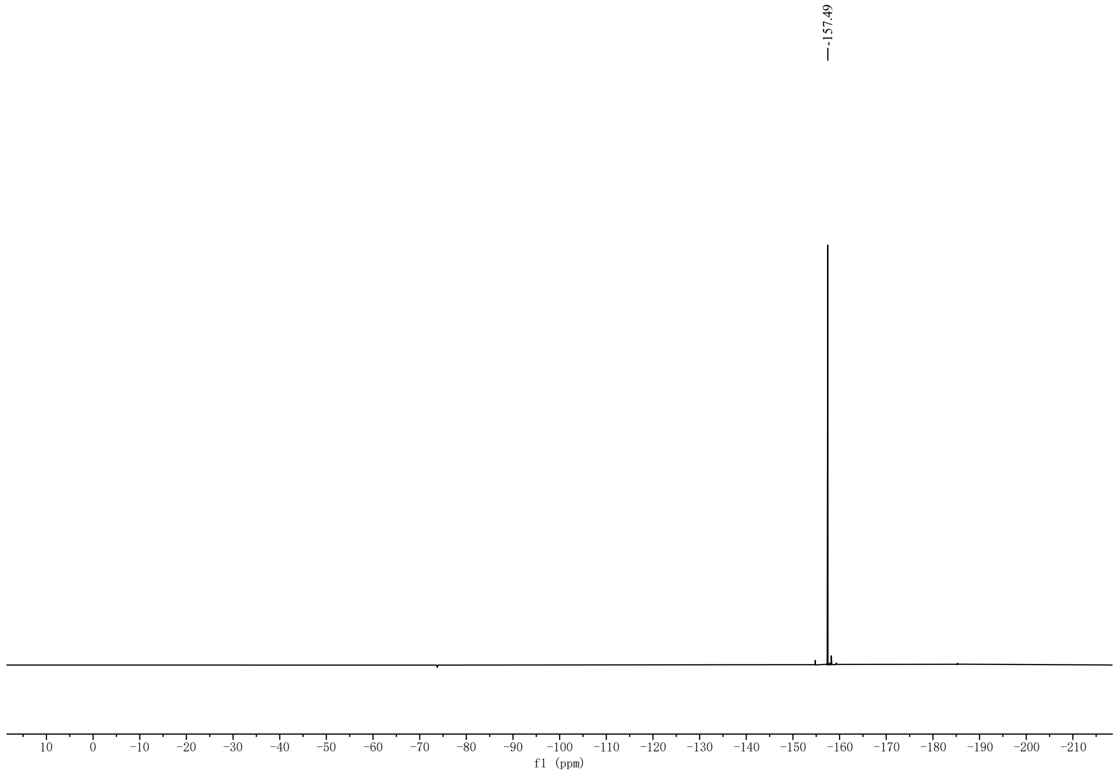

^{1}H NMR spectrum of **3i**

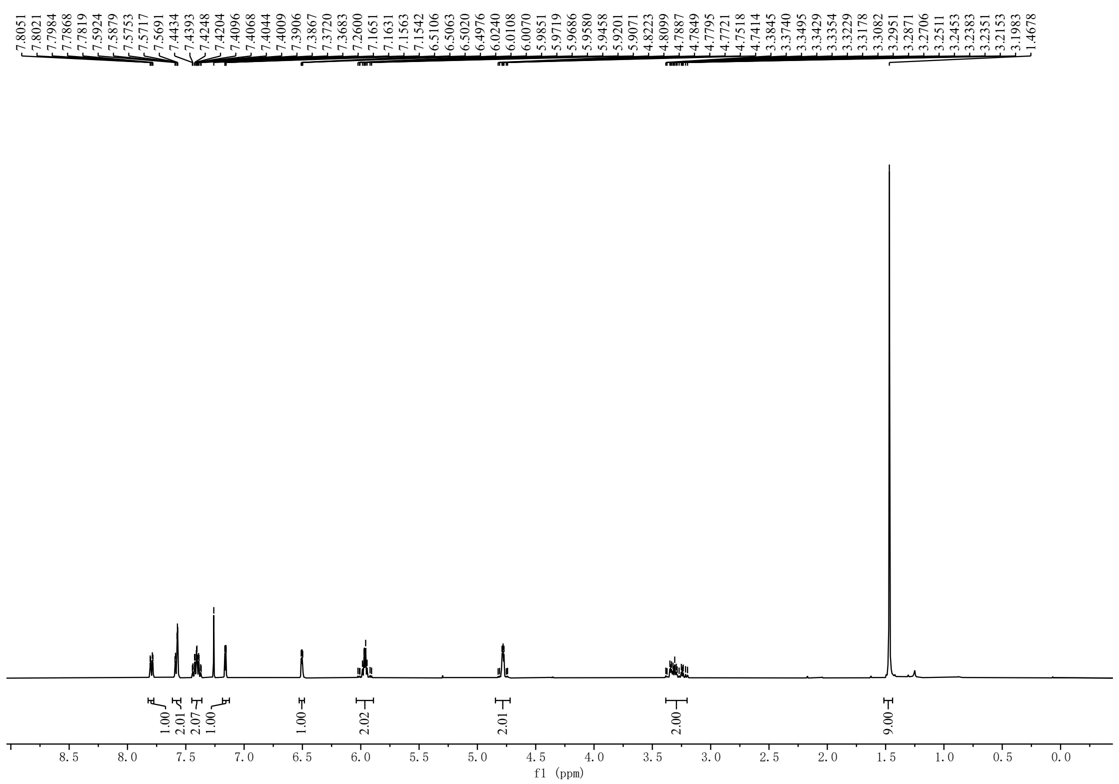

^{13}C NMR spectrum of **3i**

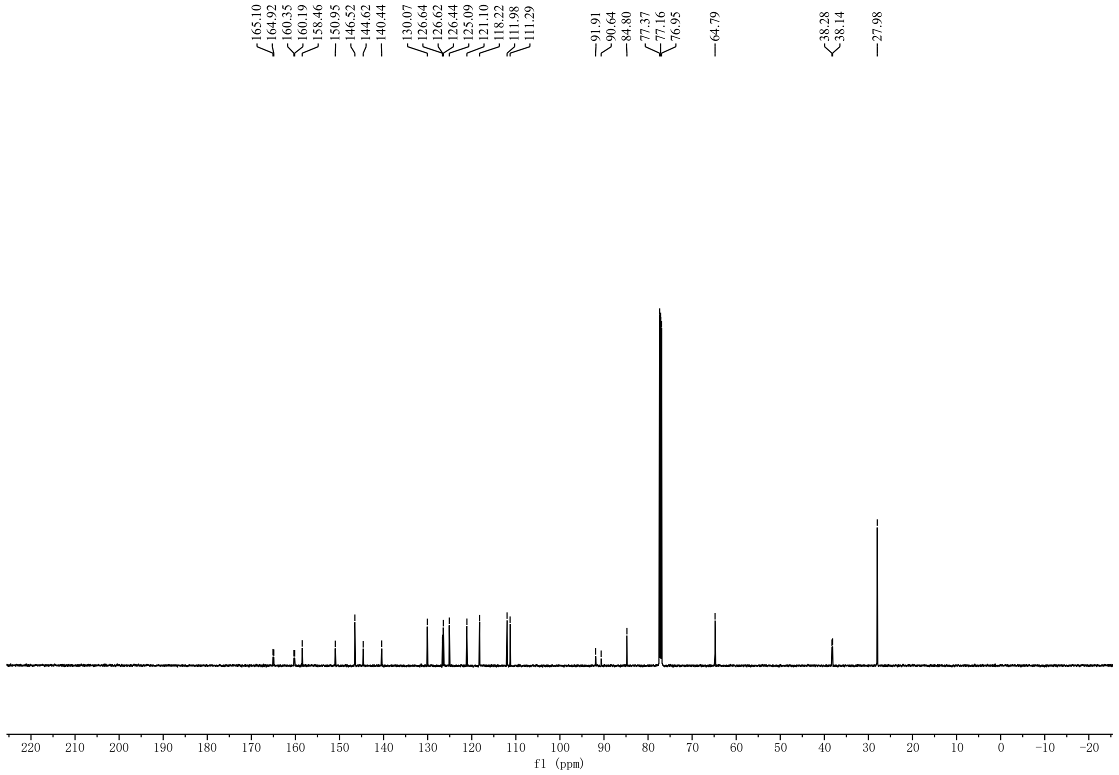

^{19}F NMR spectrum of **3i**

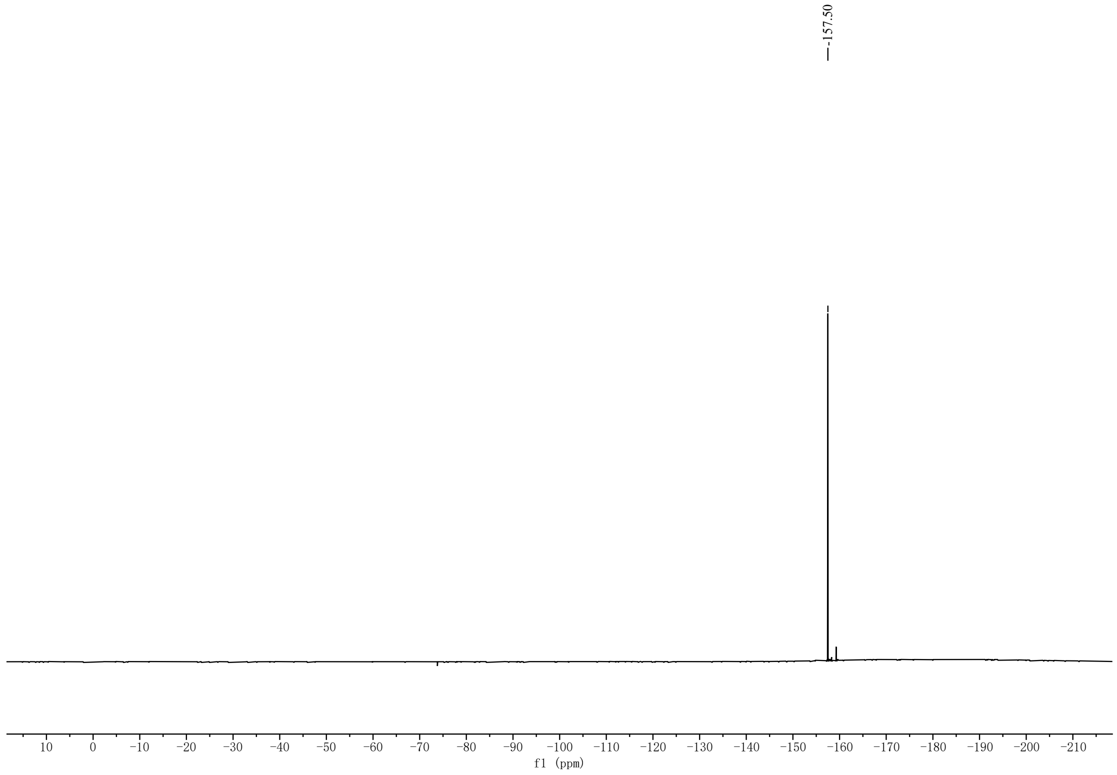

^{1}H NMR spectrum of **3j**

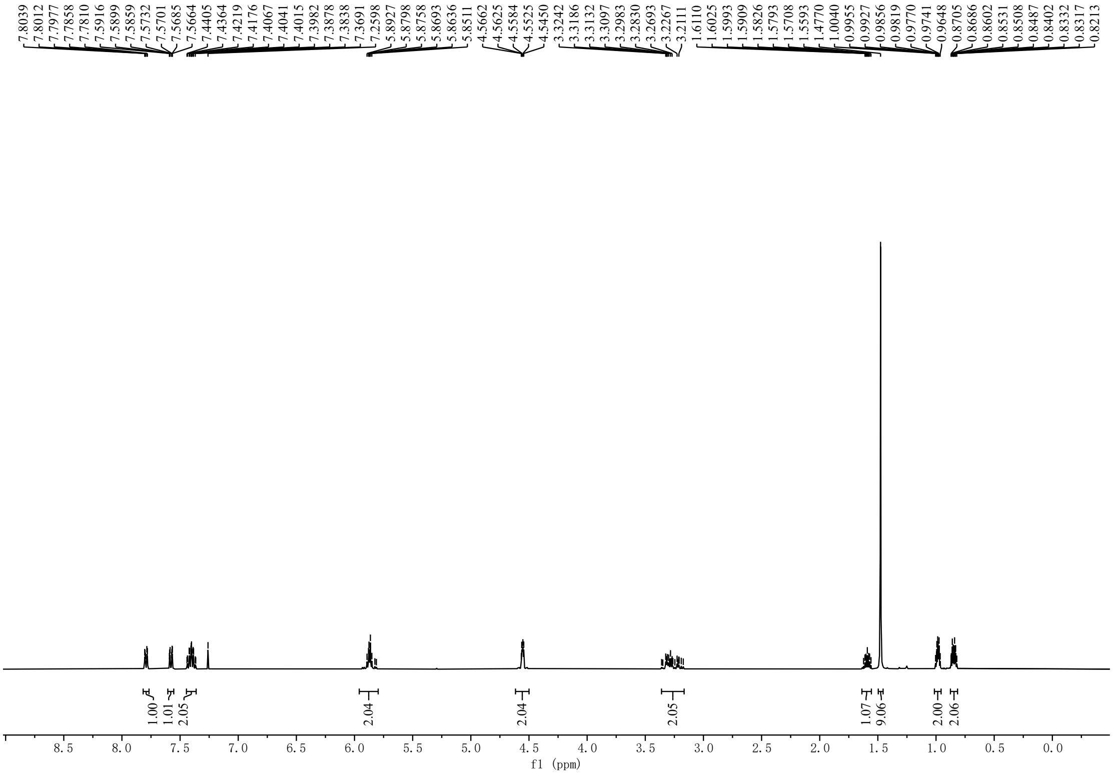

^{13}C NMR spectrum of **3j**

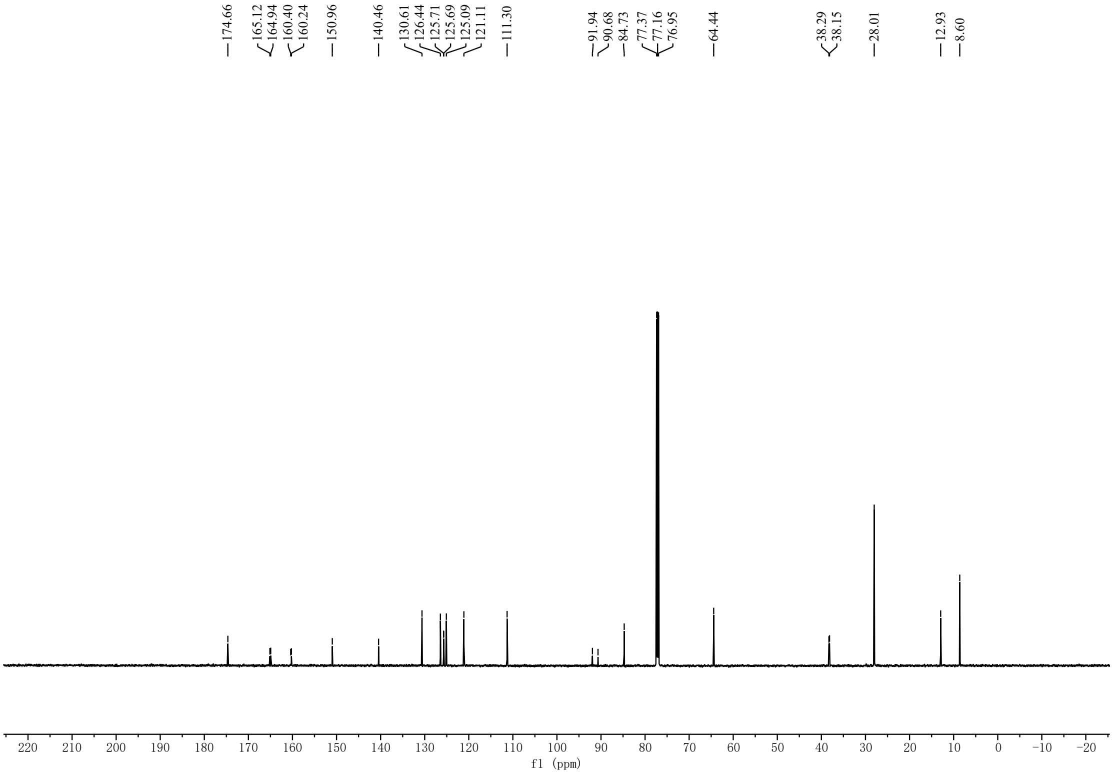

^{19}F NMR spectrum of **3j**

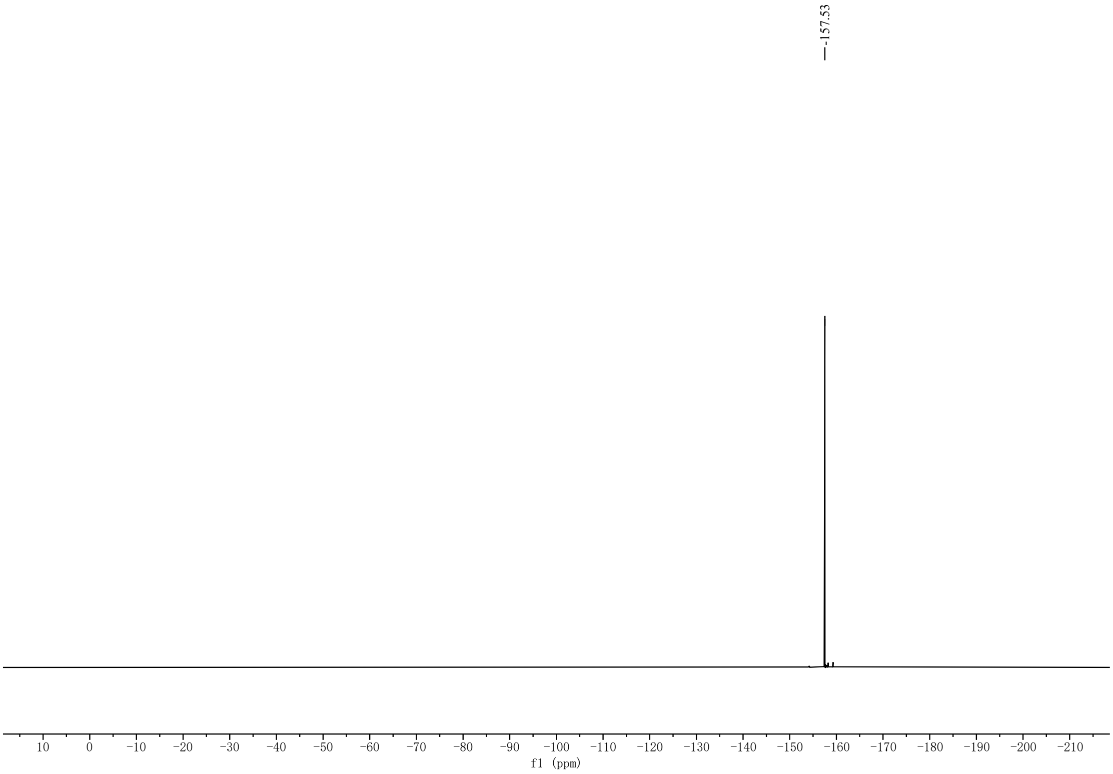

^{1}H NMR spectrum of **3k** (The product was isolated as a 5:1 mixture of E/Z isomers)

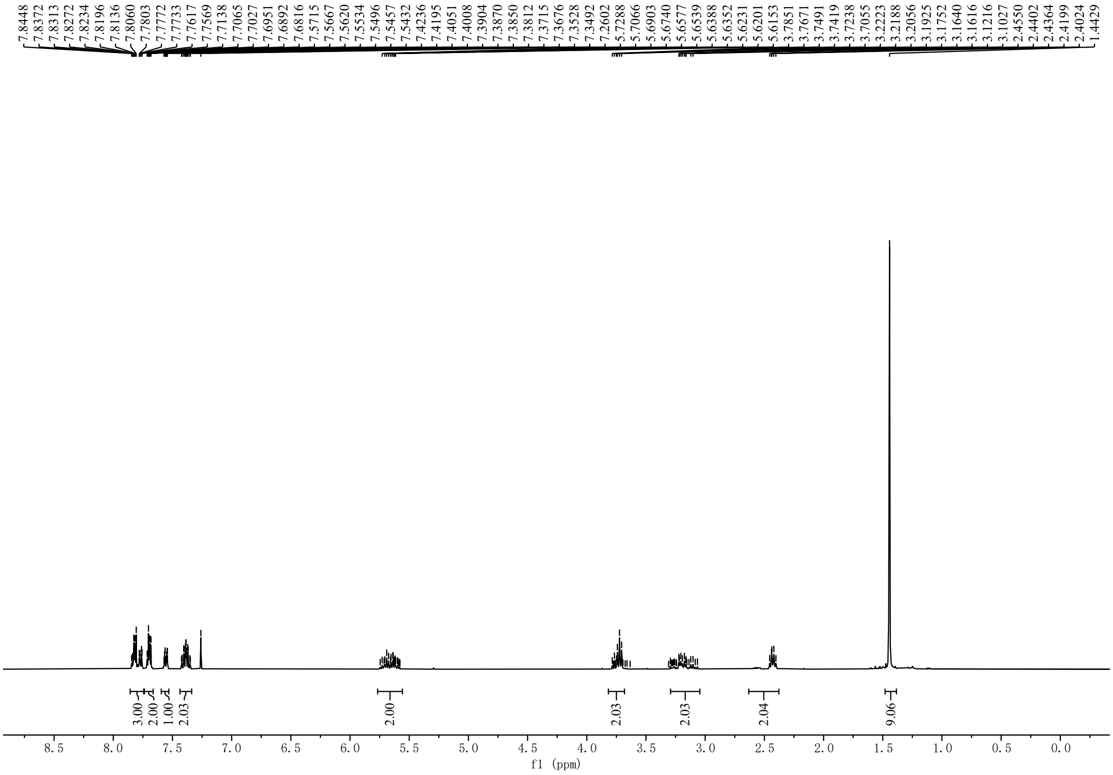

^{13}C NMR spectrum of **3k**

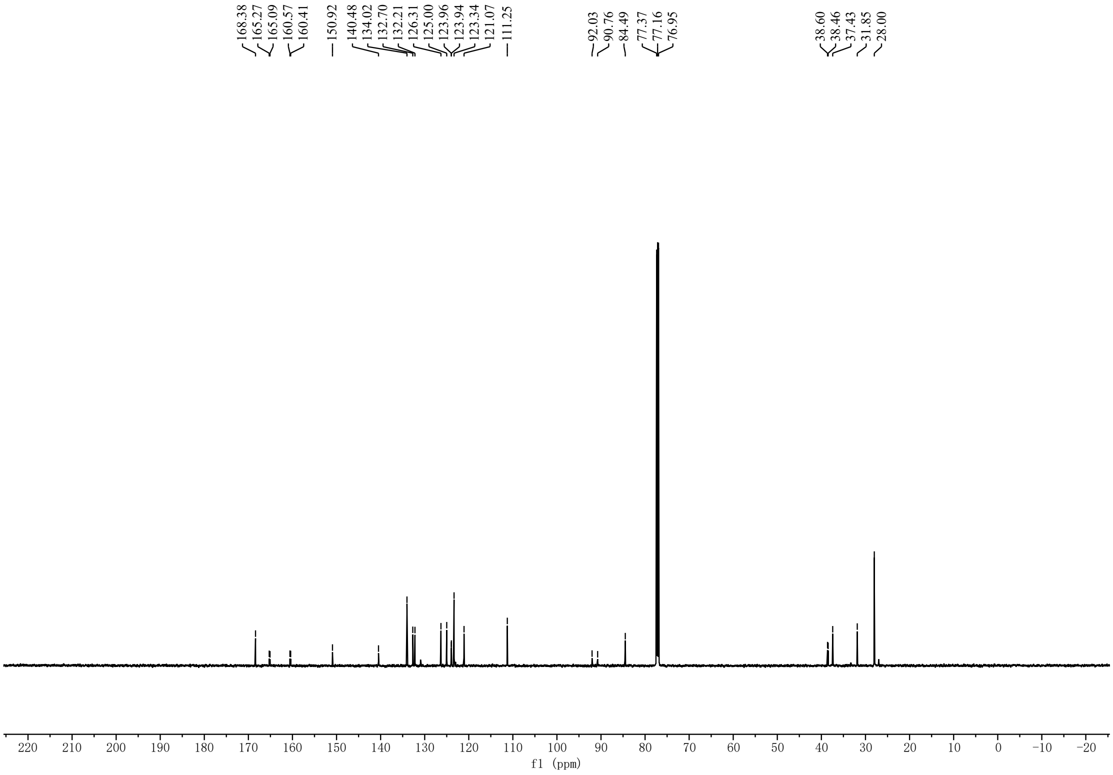

^{19}F NMR spectrum of **3k**

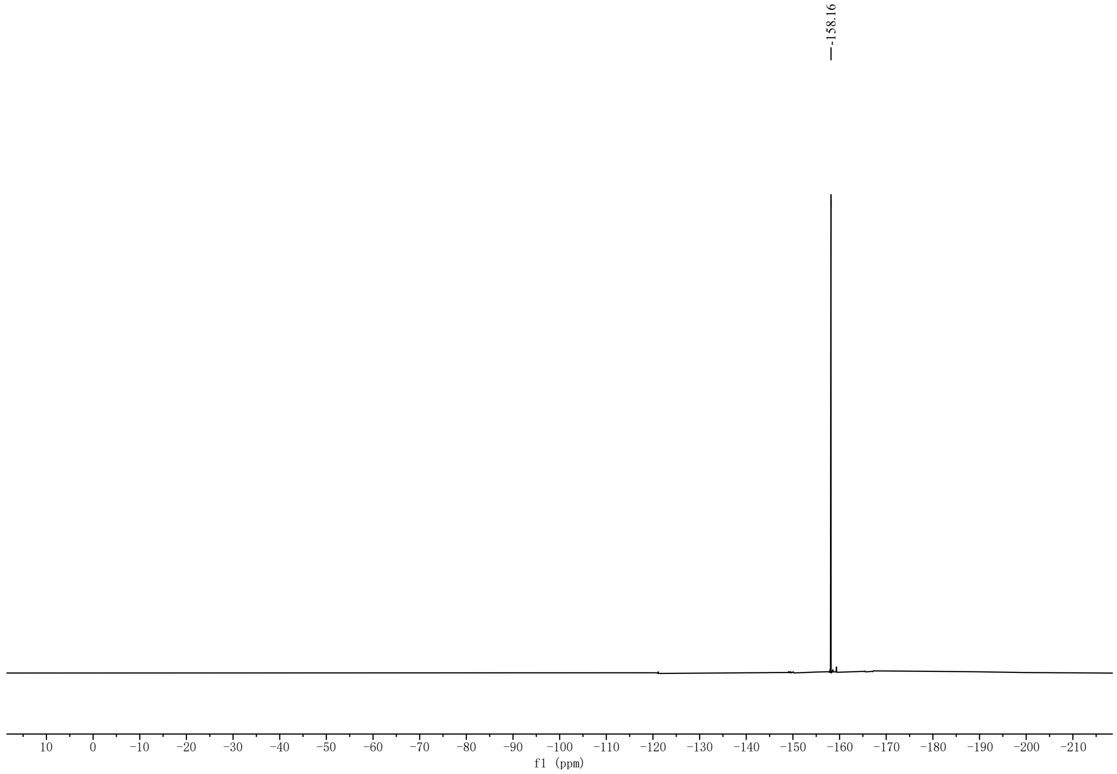

^{1}H NMR spectrum of **3l**

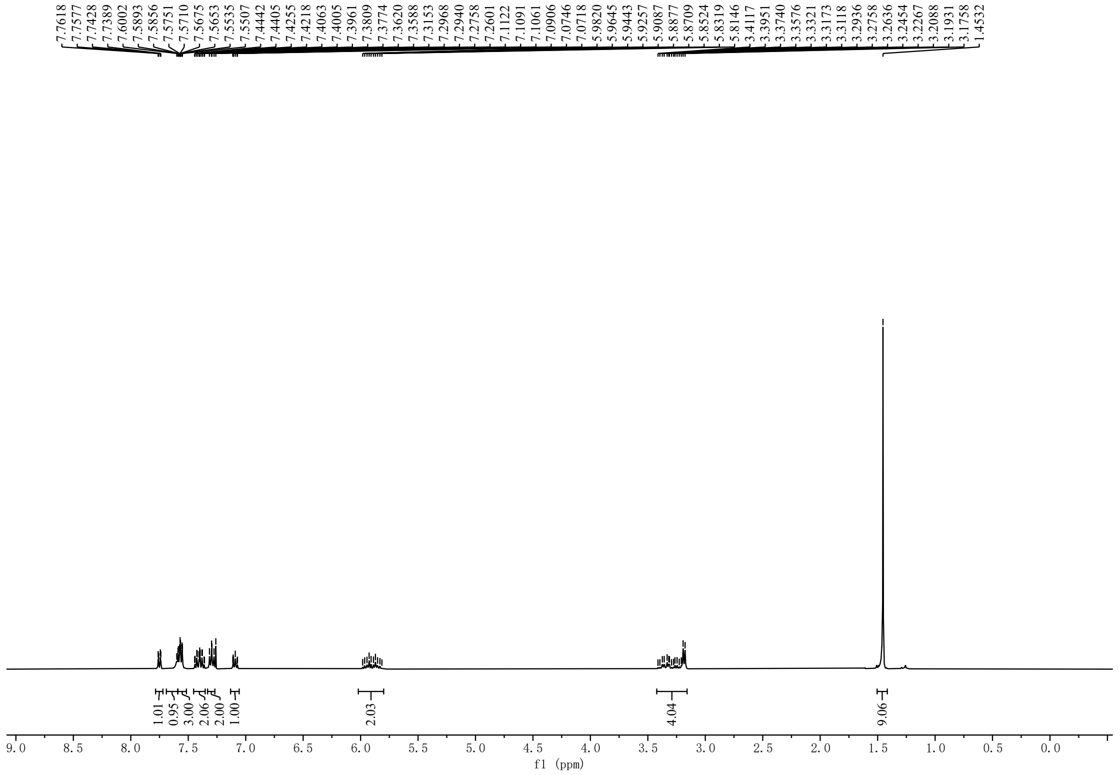

^{13}C NMR spectrum of **3l**

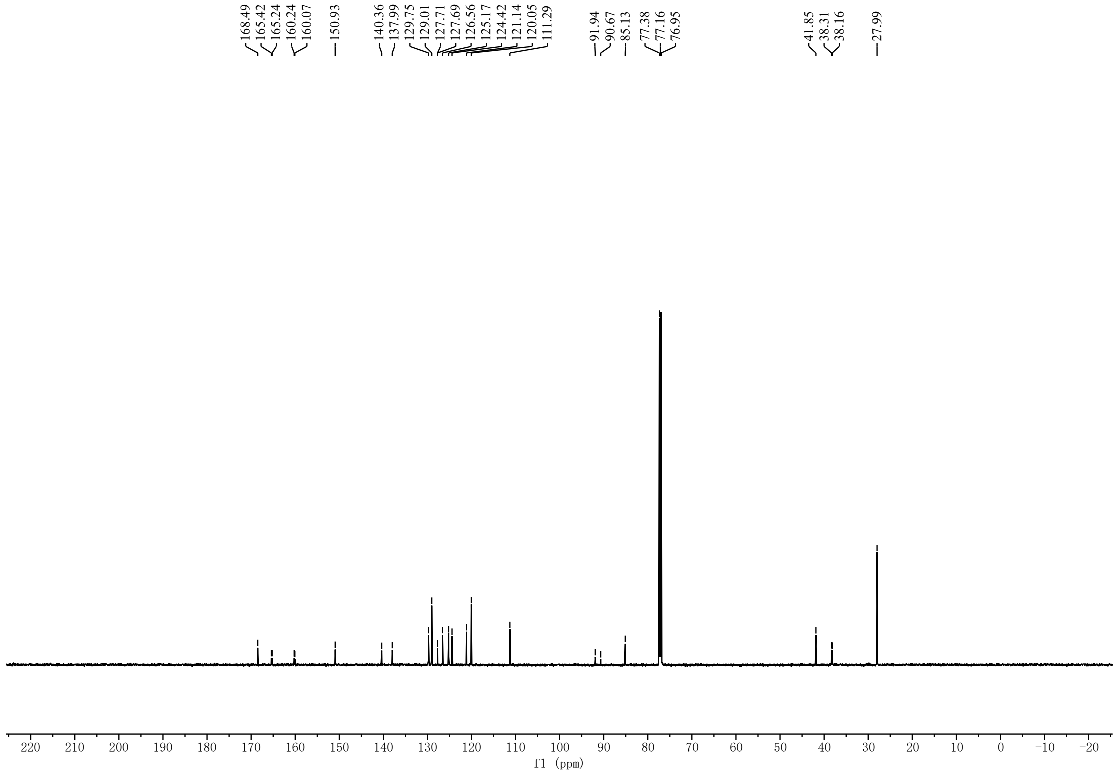

^{19}F NMR spectrum of **3l**

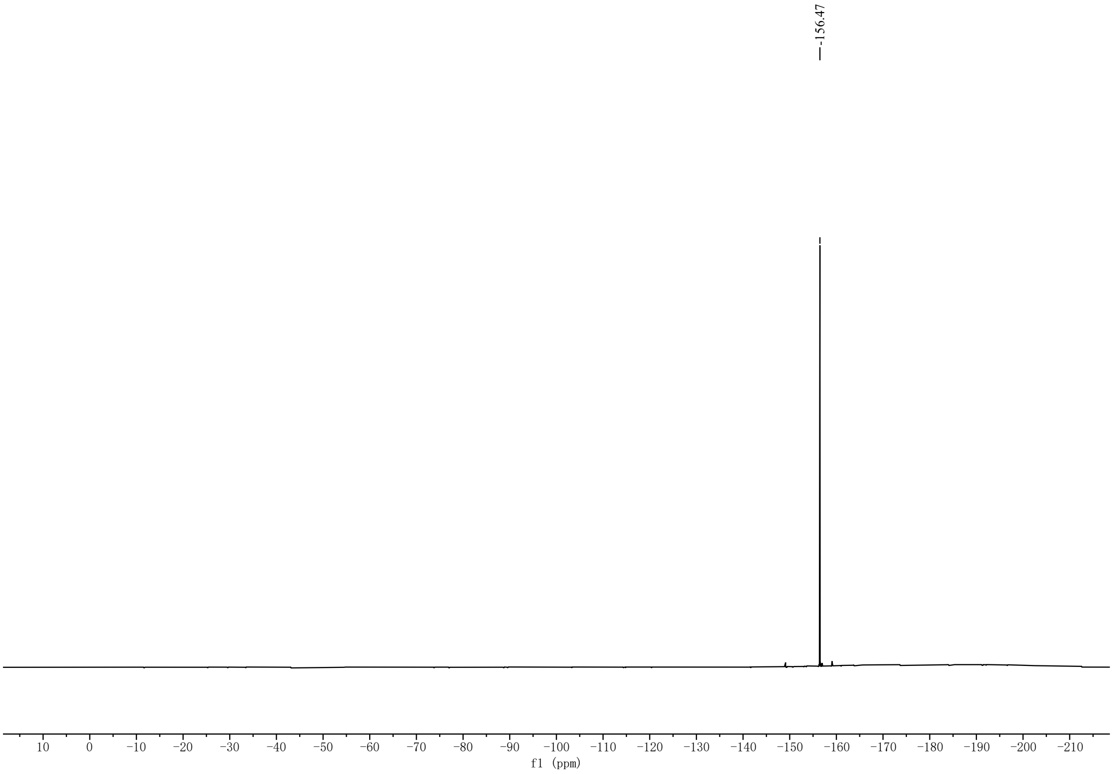

^{1}H NMR spectrum of **3m**

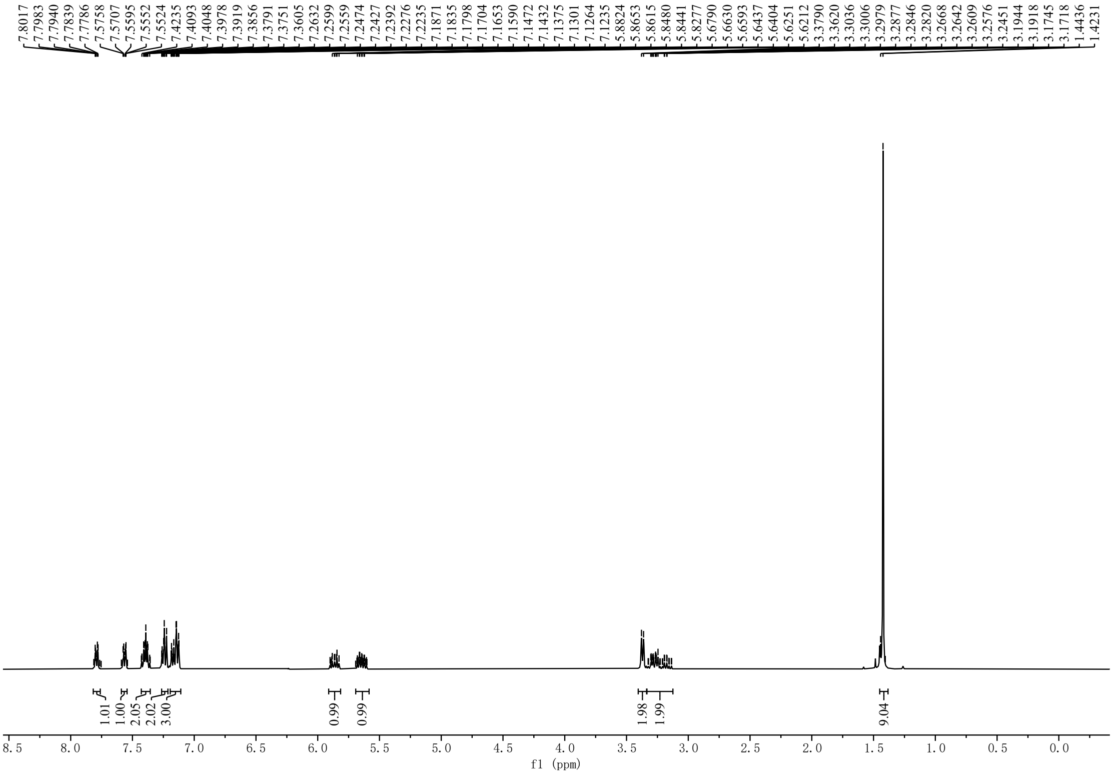

^{13}C NMR spectrum of **3m**

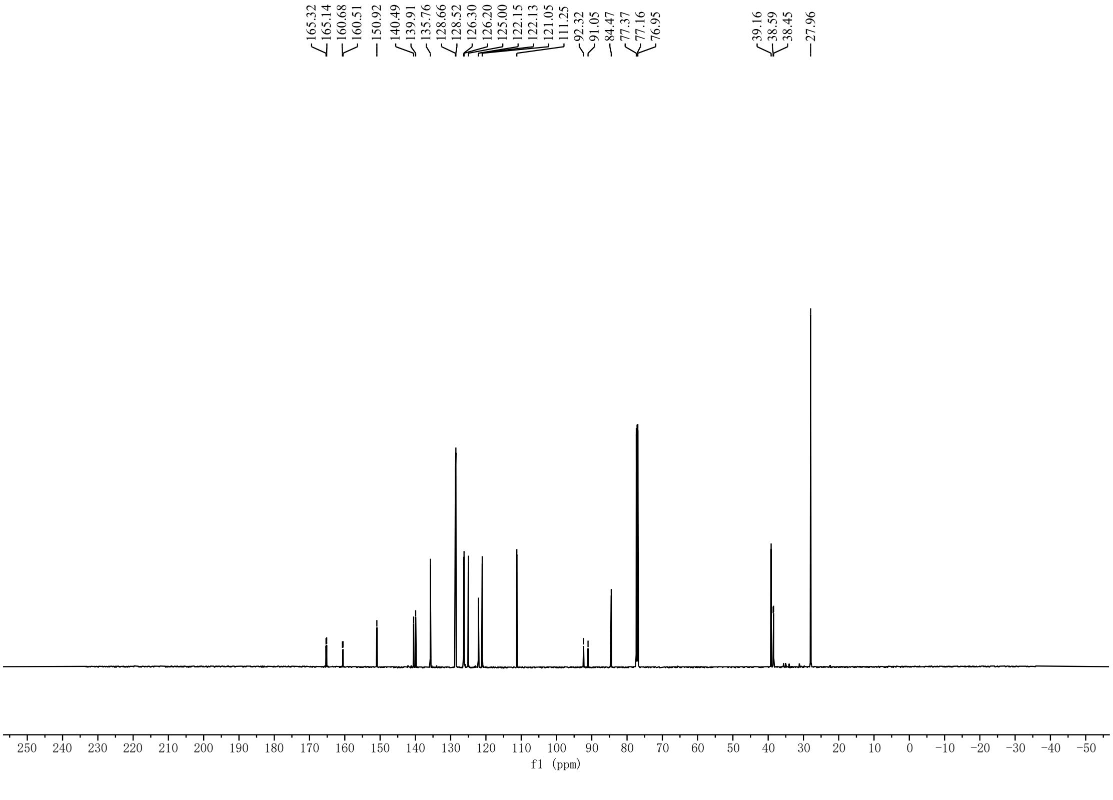

^{19}F NMR spectrum of **3m**

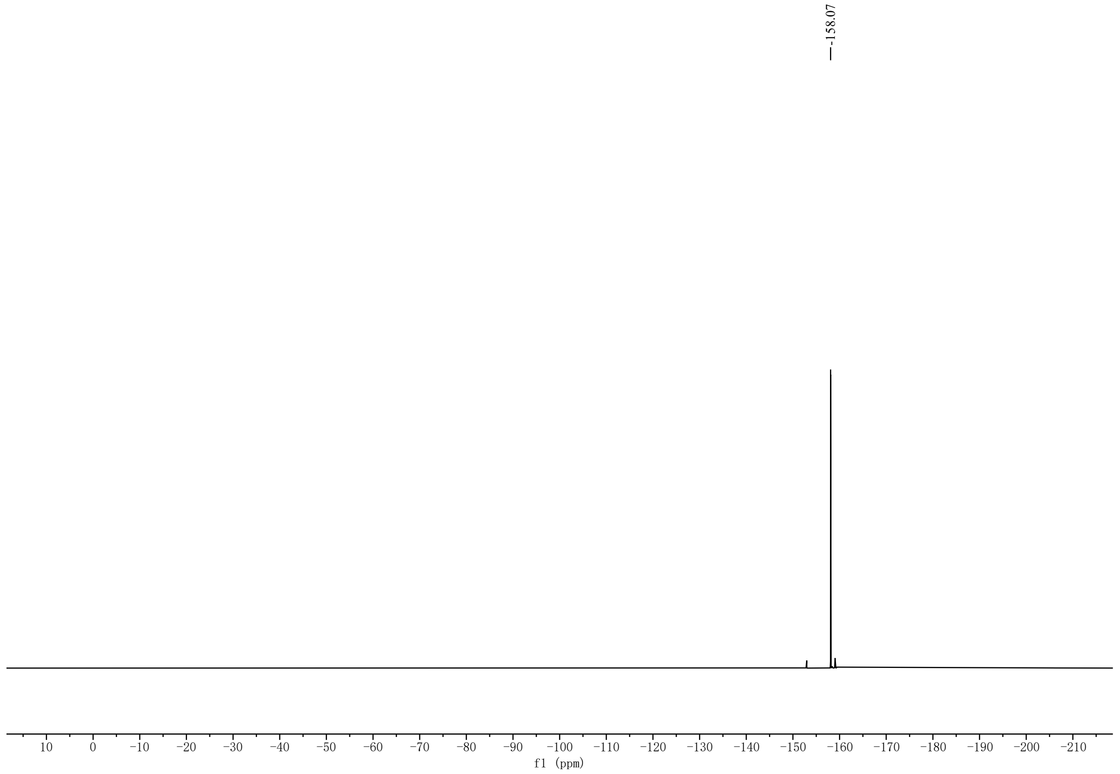

^{1}H NMR spectrum of **3n**

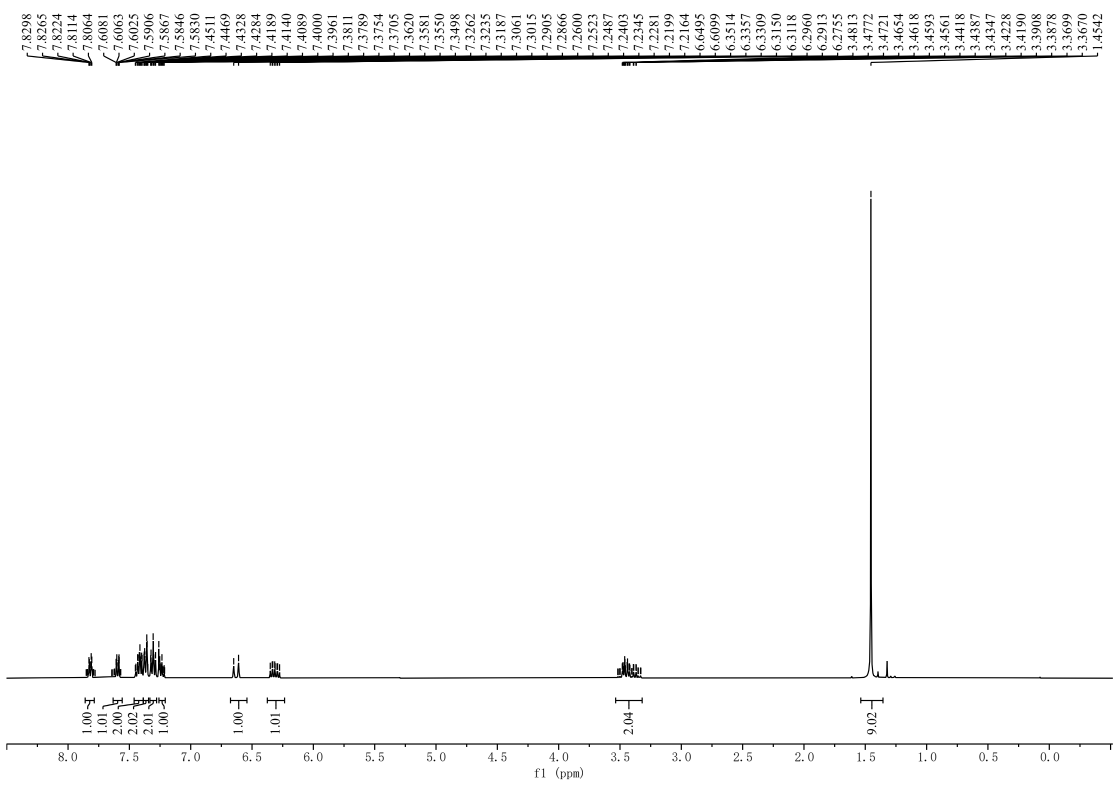

^{13}C NMR spectrum of **3n**

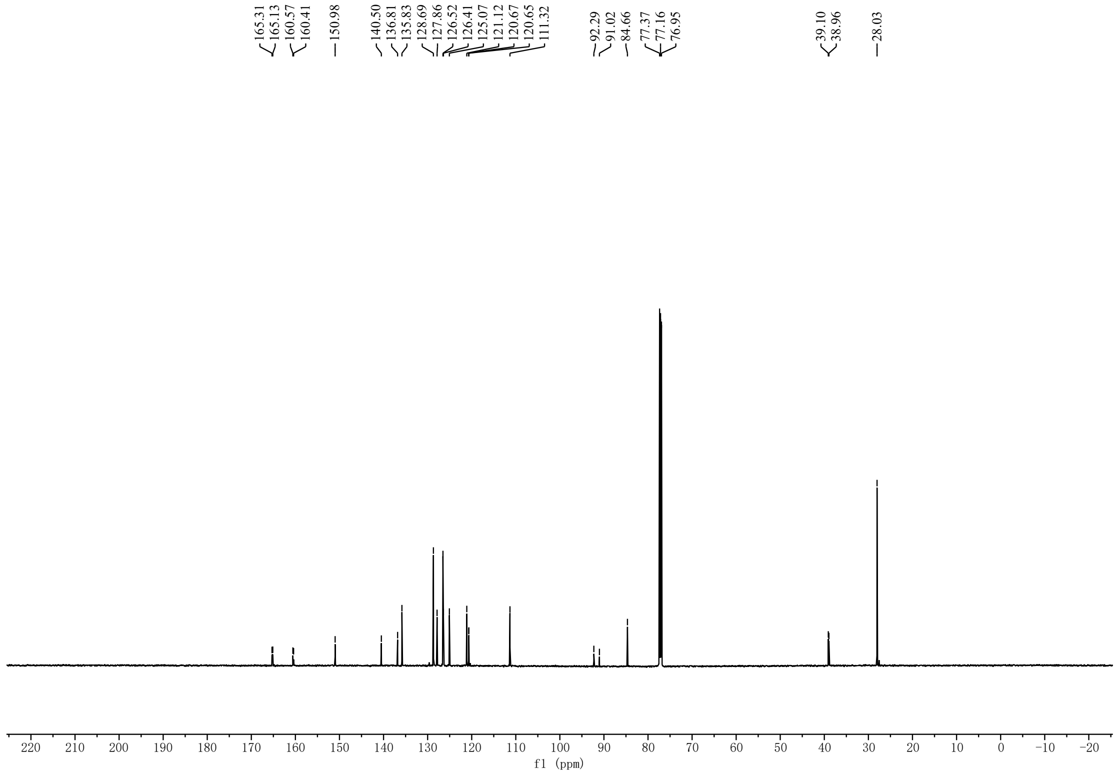

^{19}F NMR spectrum of **3n**

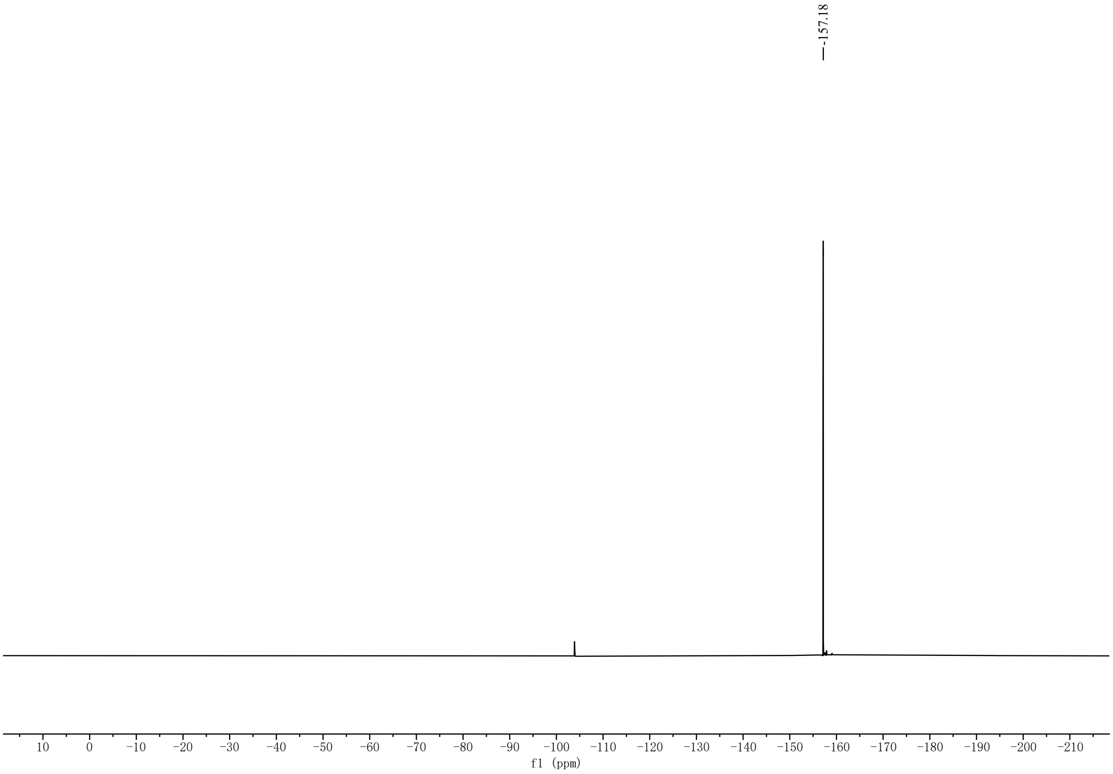

^{1}H NMR spectrum of **3o**

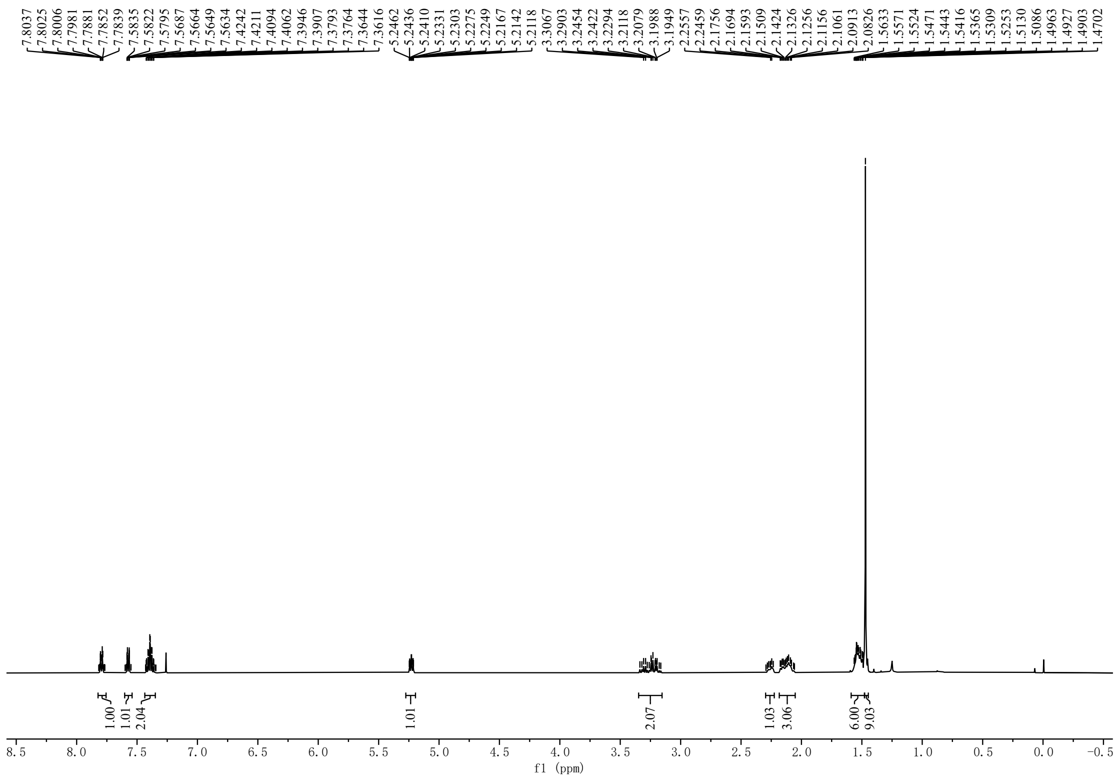

^{13}C NMR spectrum of **3o**

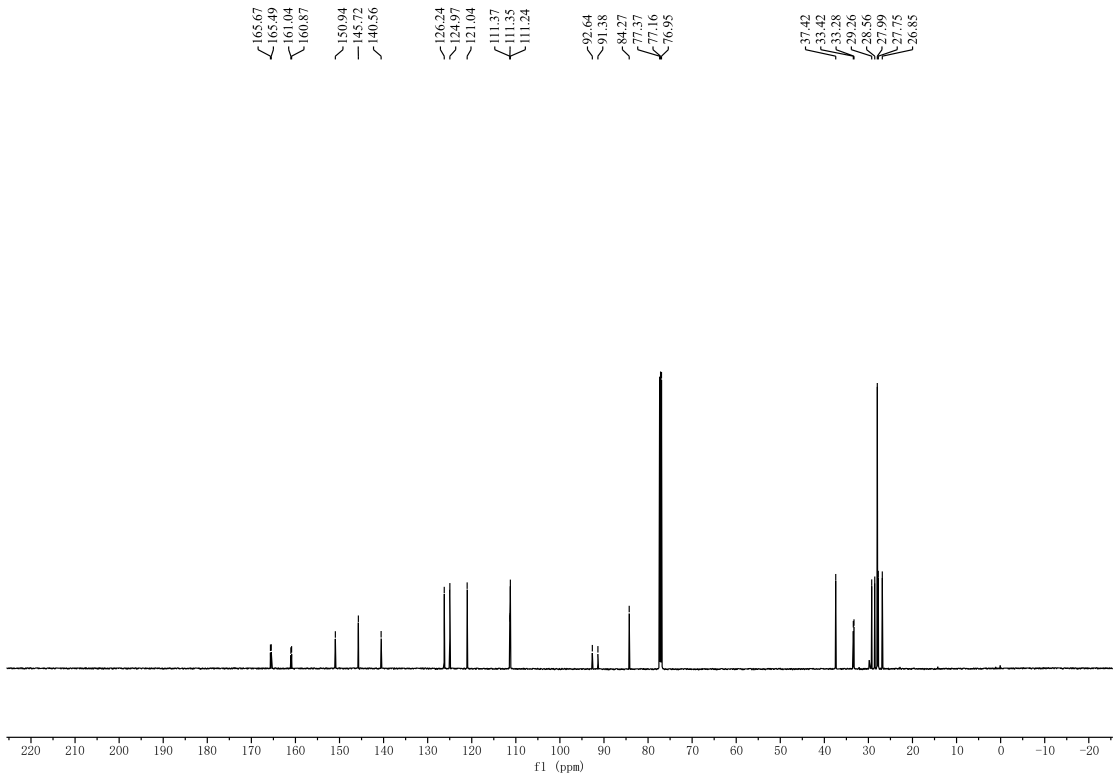

^{19}F NMR spectrum of **3o**

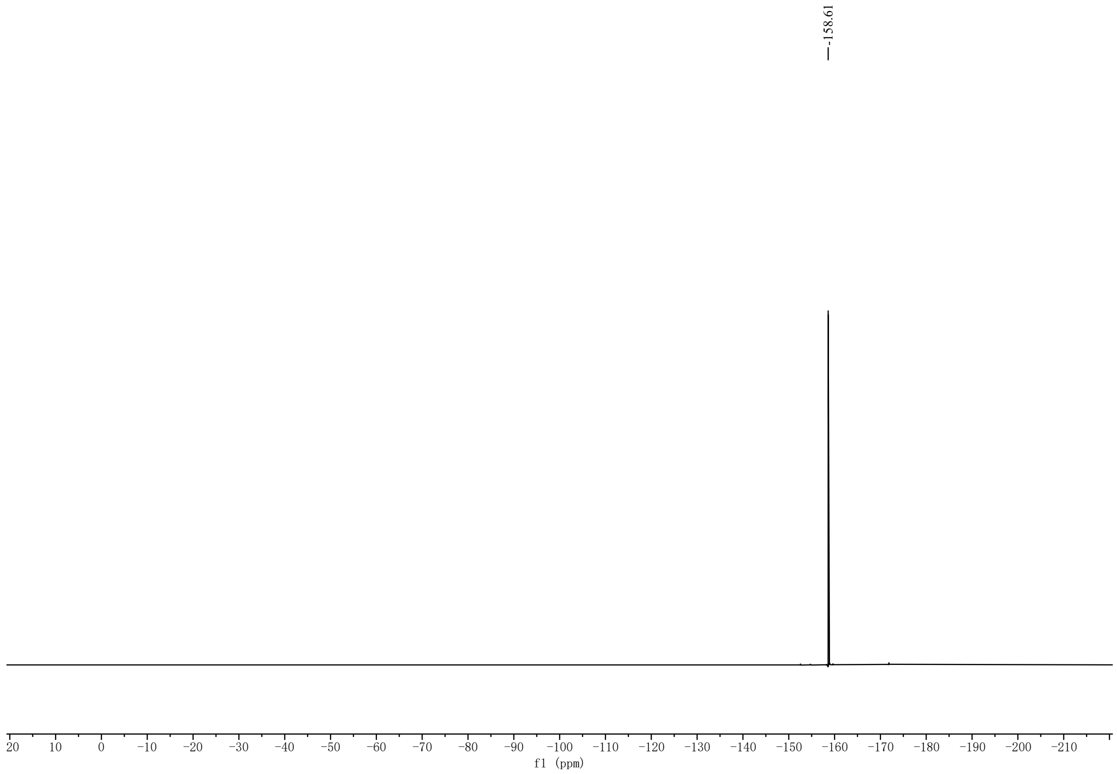

^{1}H NMR spectrum of **3p**

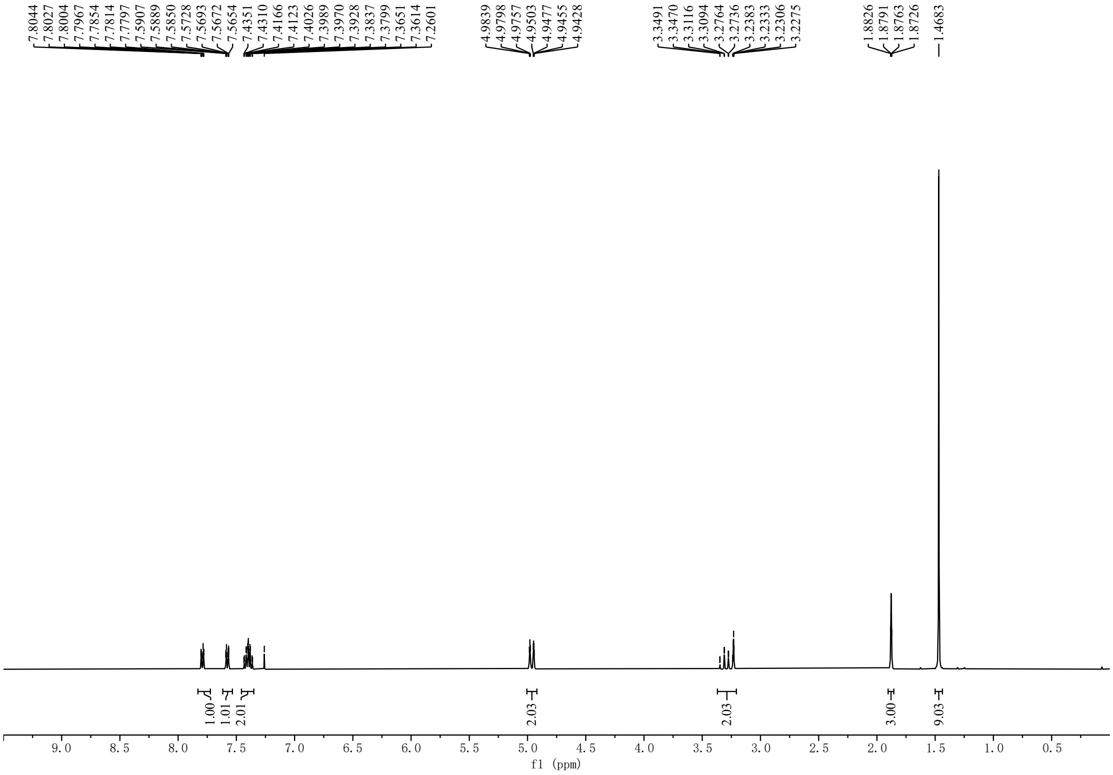

^{13}C NMR spectrum of **3p**

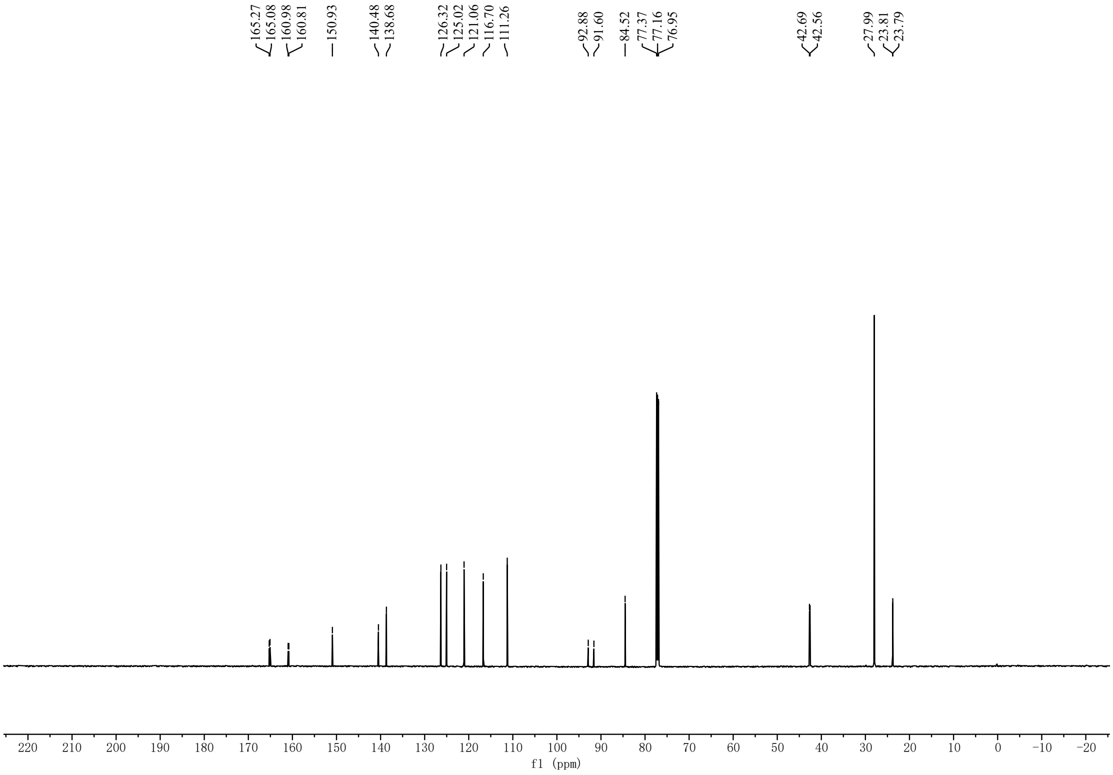

^{19}F NMR spectrum of **3p**

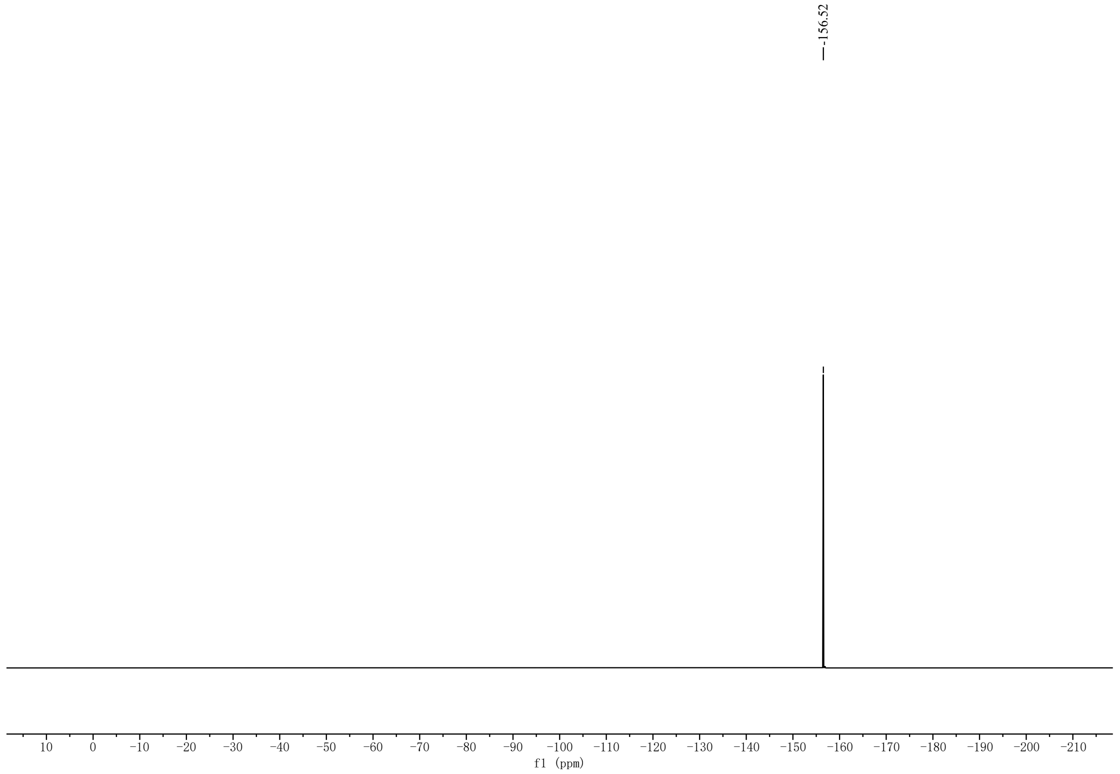

^{1}H NMR spectrum of **3q**

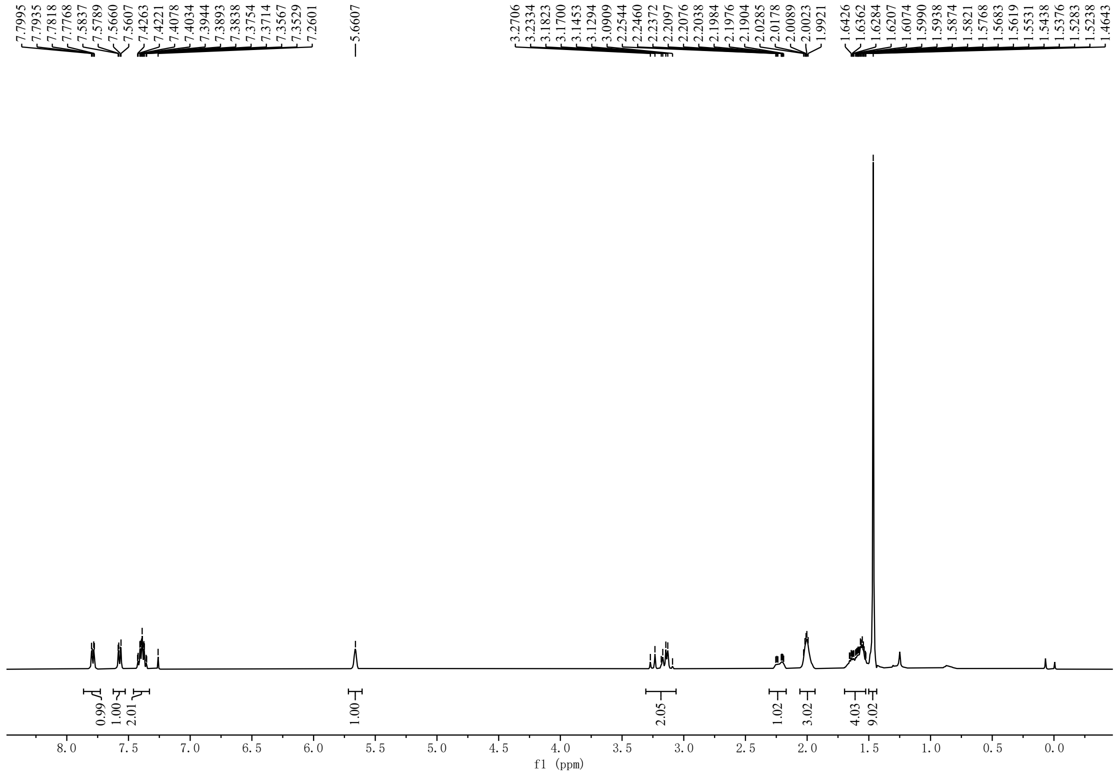

^{13}C NMR spectrum of **3q**

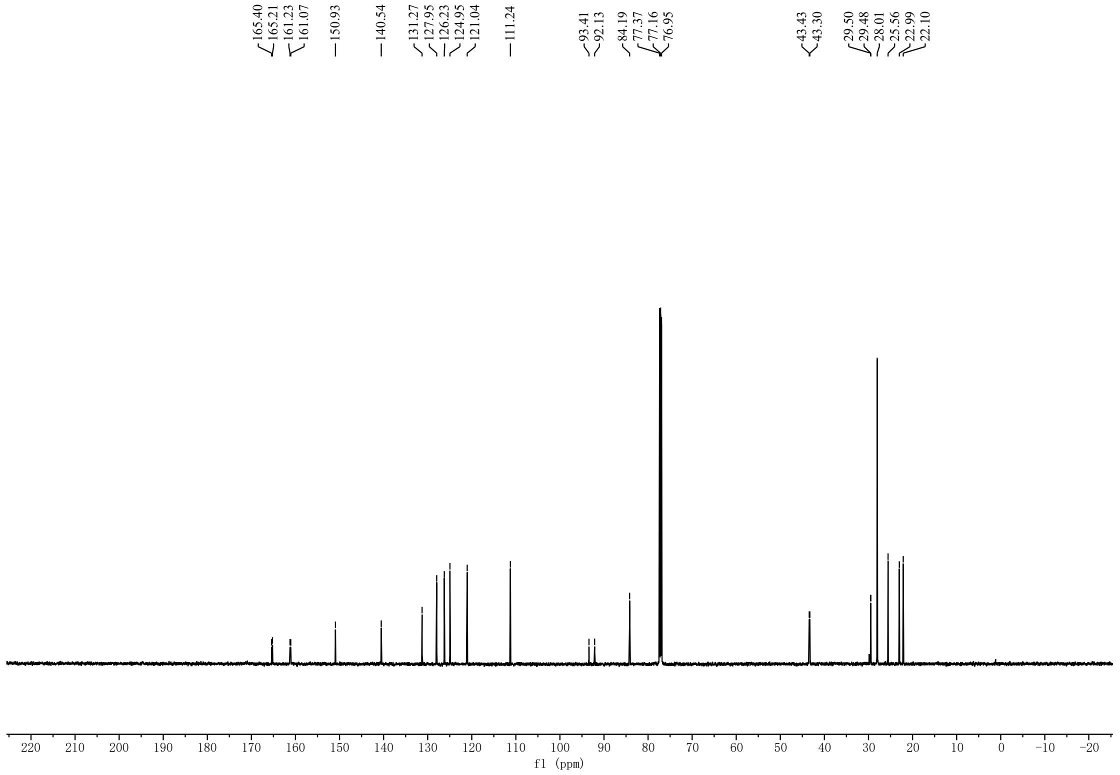

^{19}F NMR spectrum of **3q**

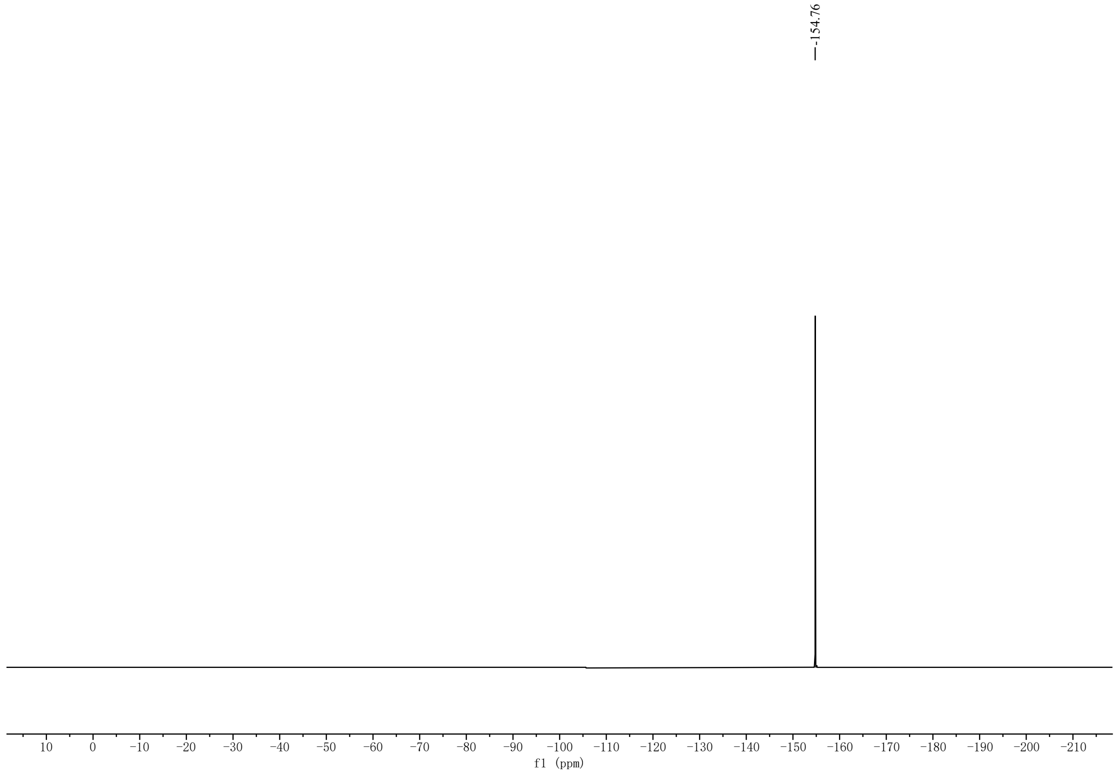

^{1}H NMR spectrum of **3r**

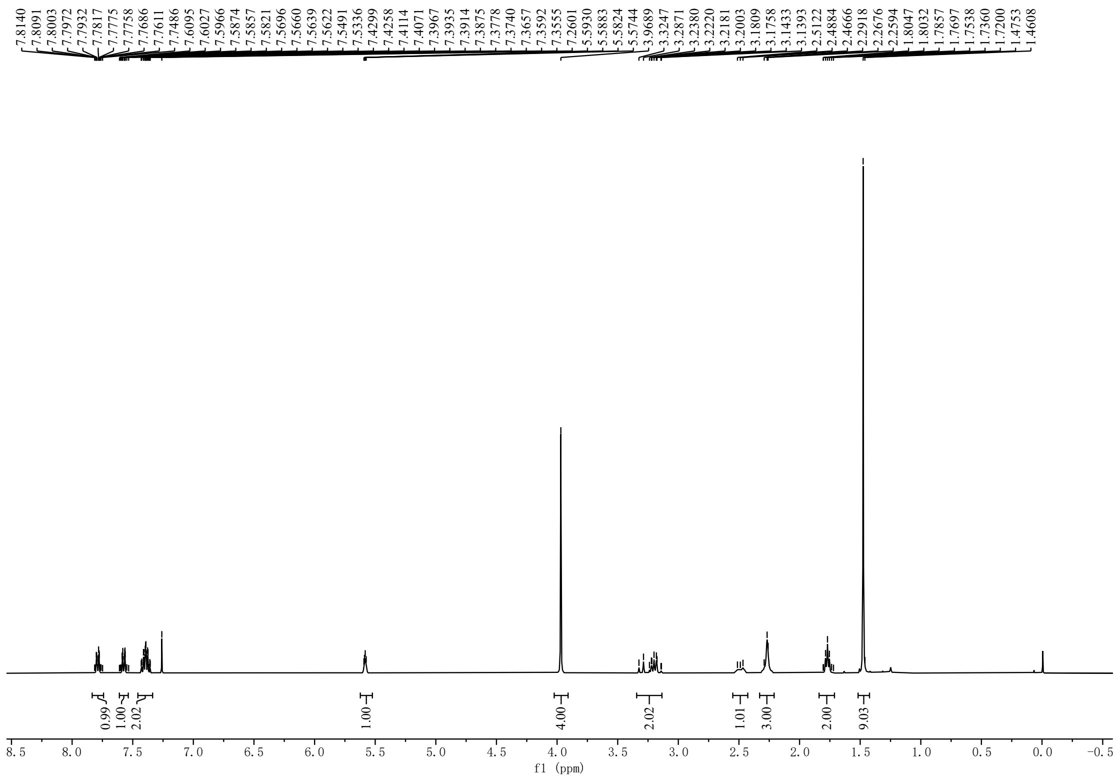

^{13}C NMR spectrum of **3r**

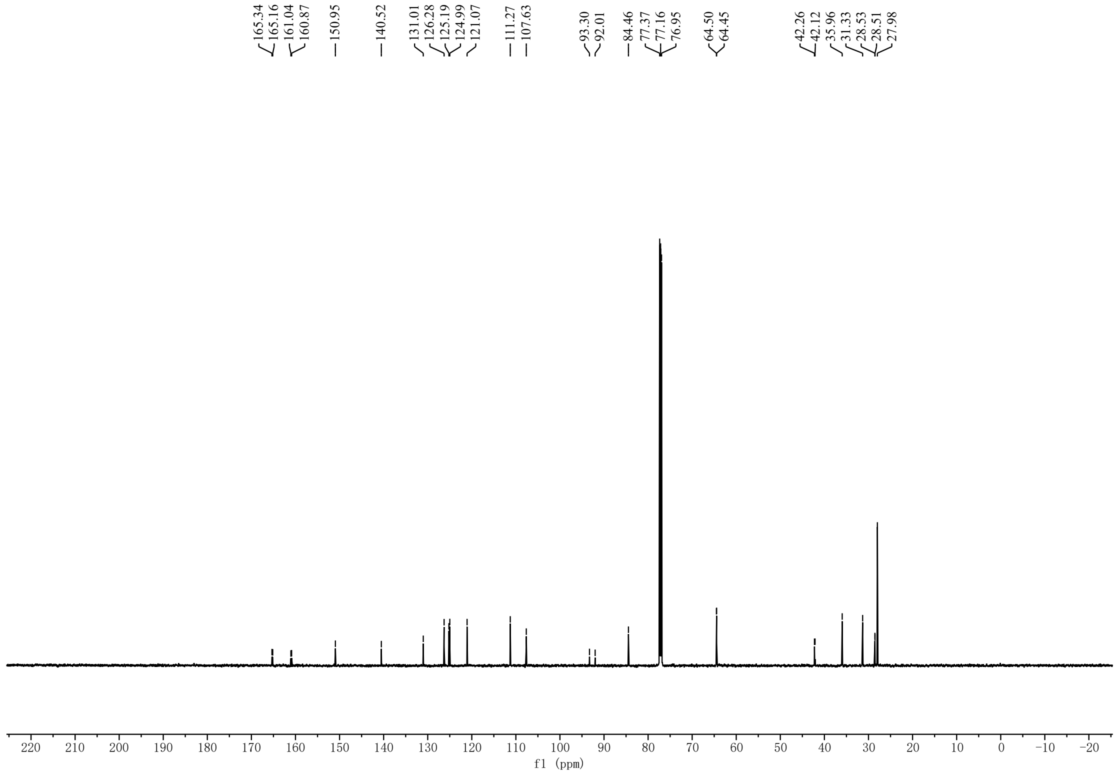

^{19}F NMR spectrum of **3r**

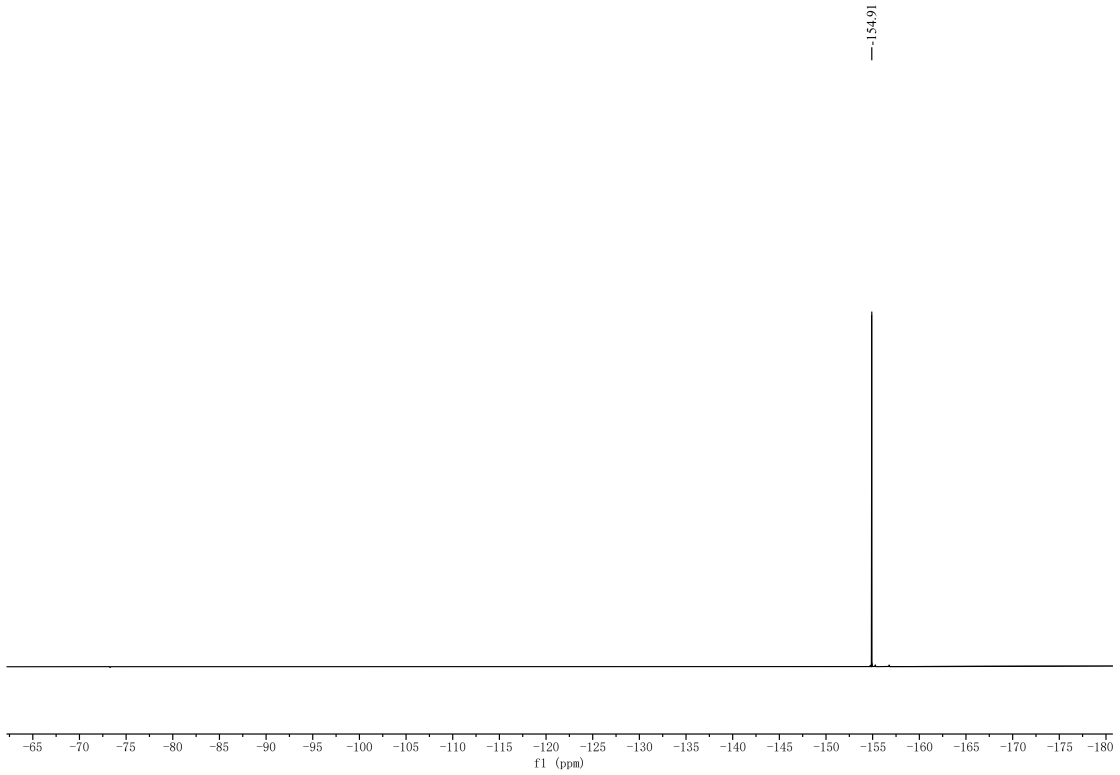

^{1}H NMR spectrum of **3s**

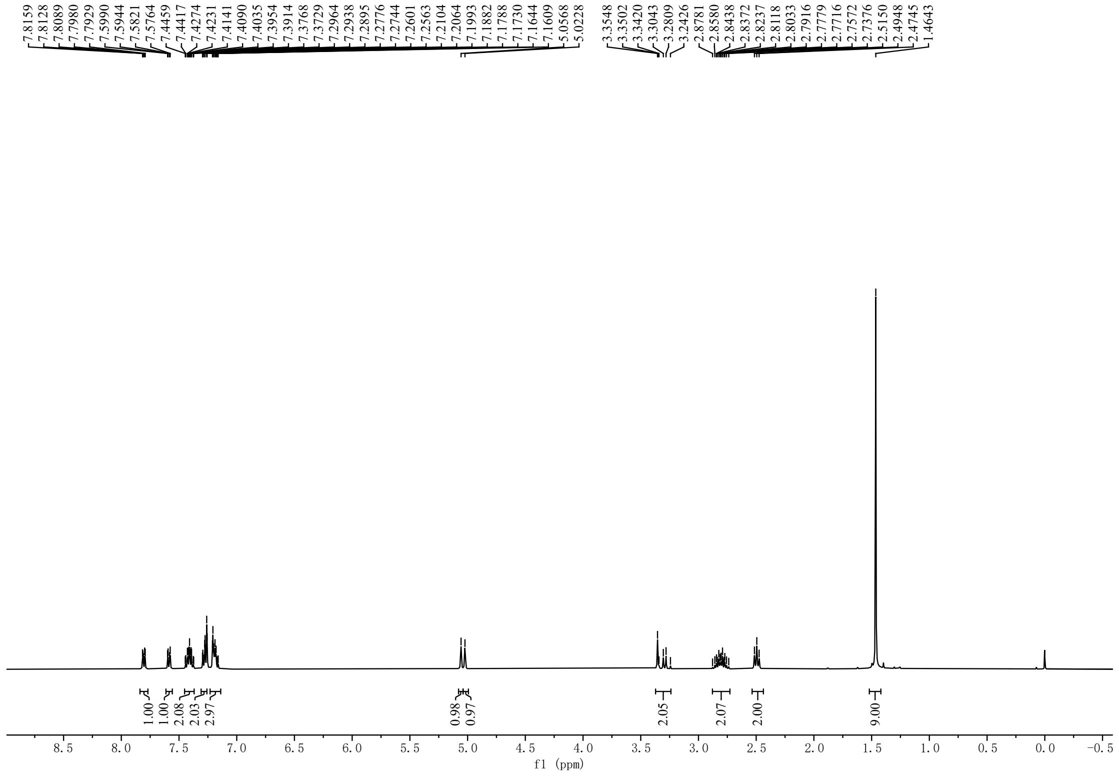

^{13}C NMR spectrum of **3s**

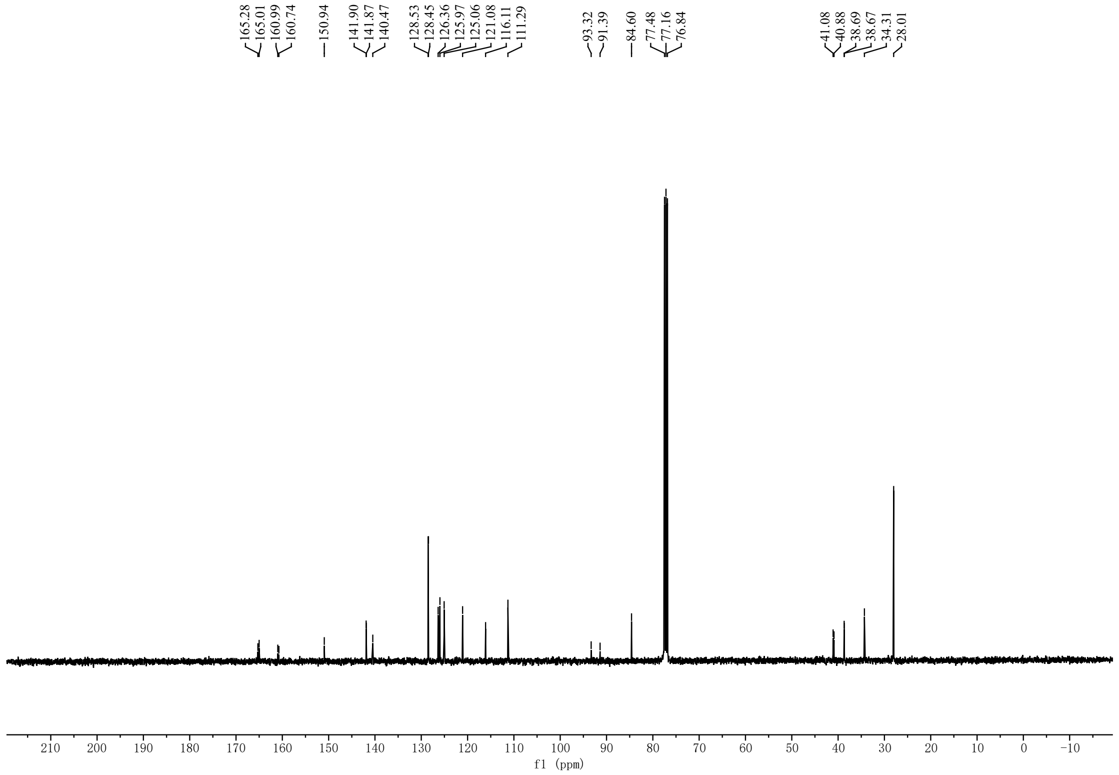

^{19}F NMR spectrum of **3s**

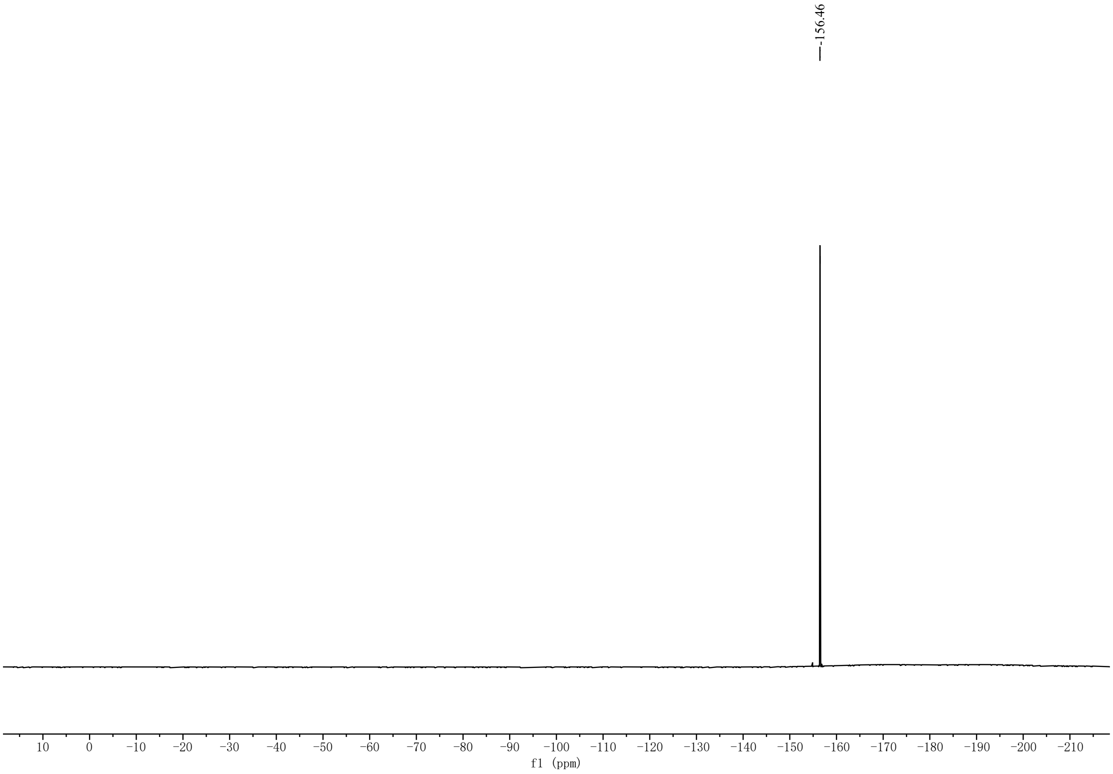

^{1}H NMR spectrum of **3t**

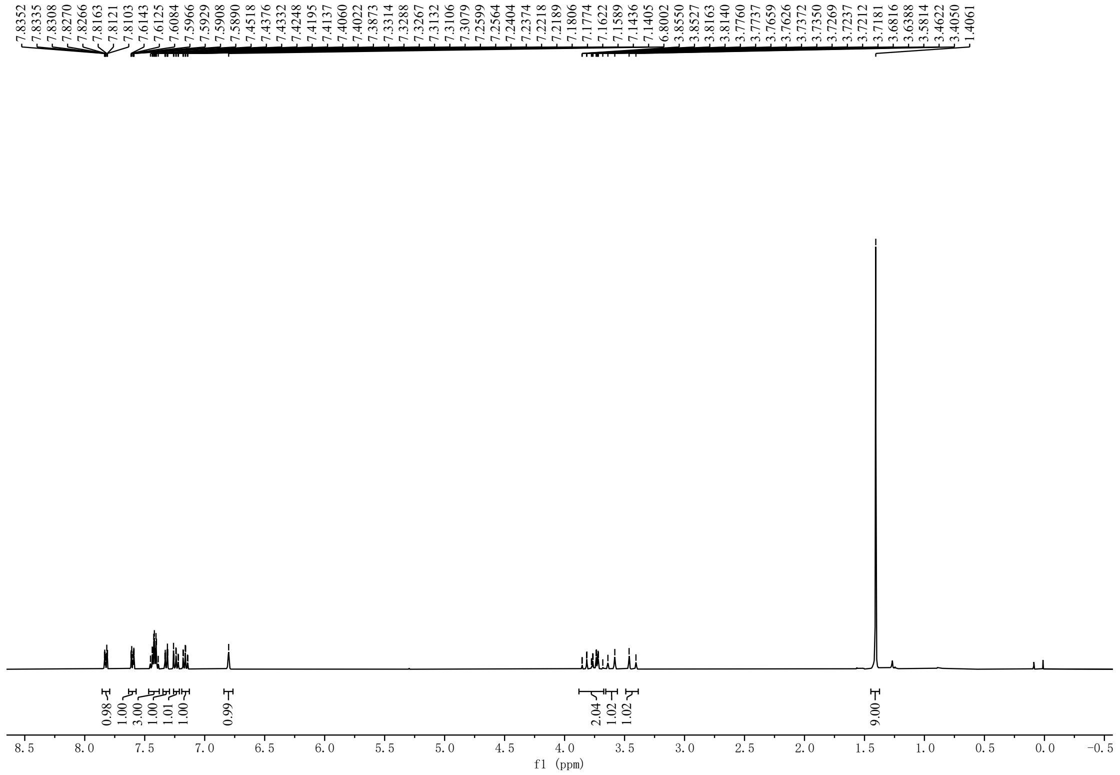

^{13}C NMR spectrum of **3t**

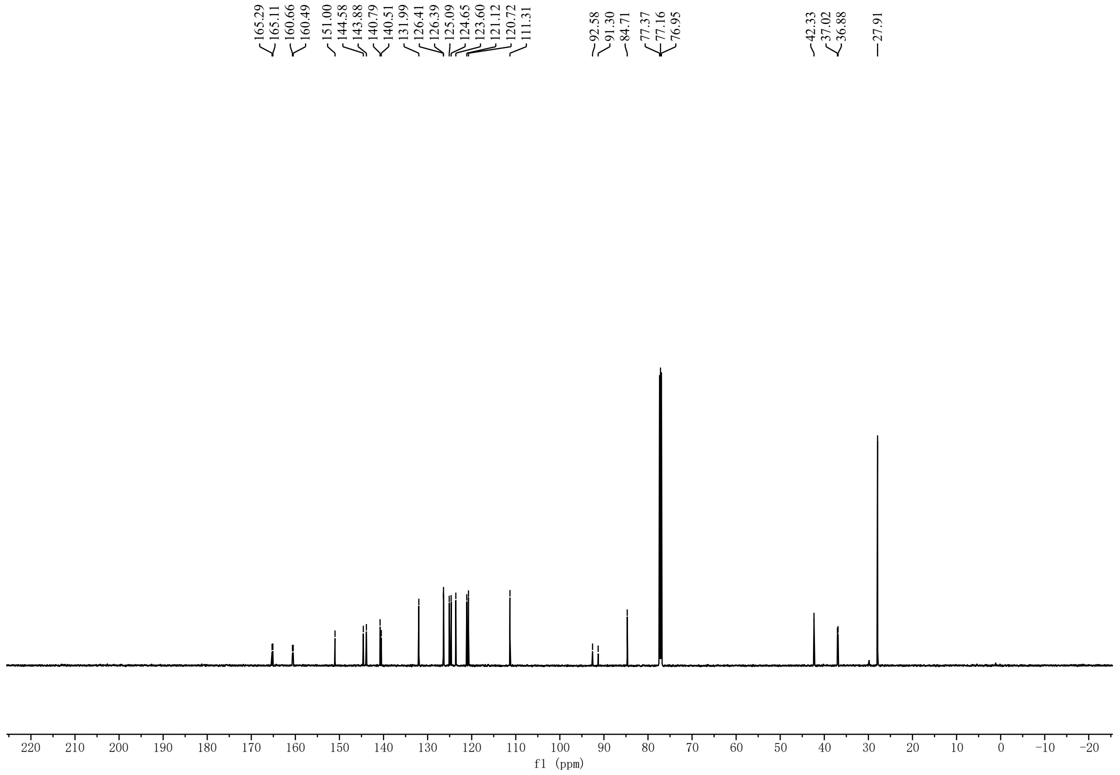

^{19}F NMR spectrum of **3t**

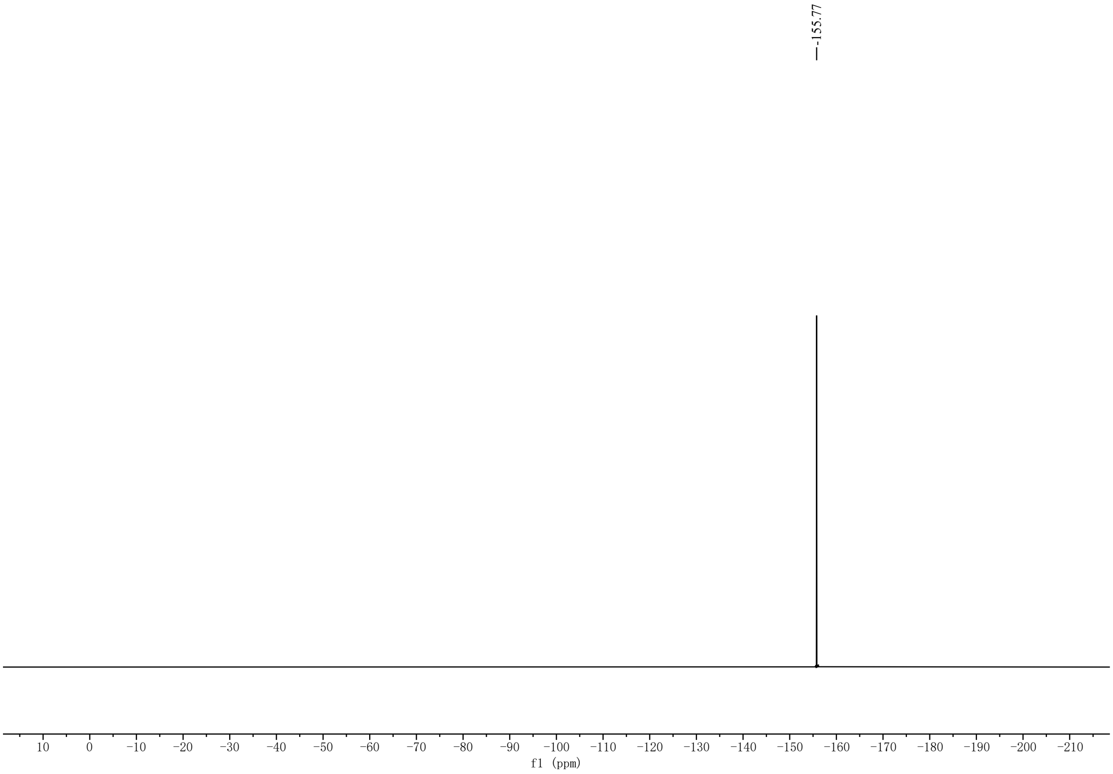

^{1}H NMR spectrum of **3u** (d.r. = 10 : 1)

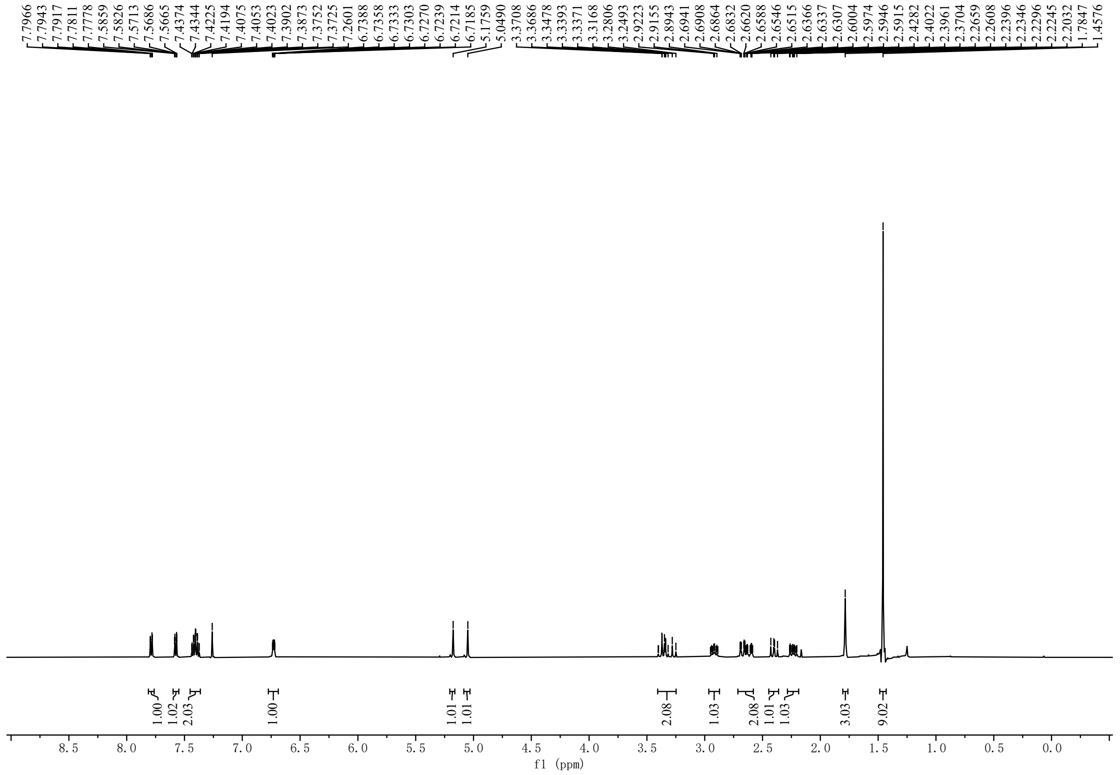

^{13}C NMR spectrum of **3u**

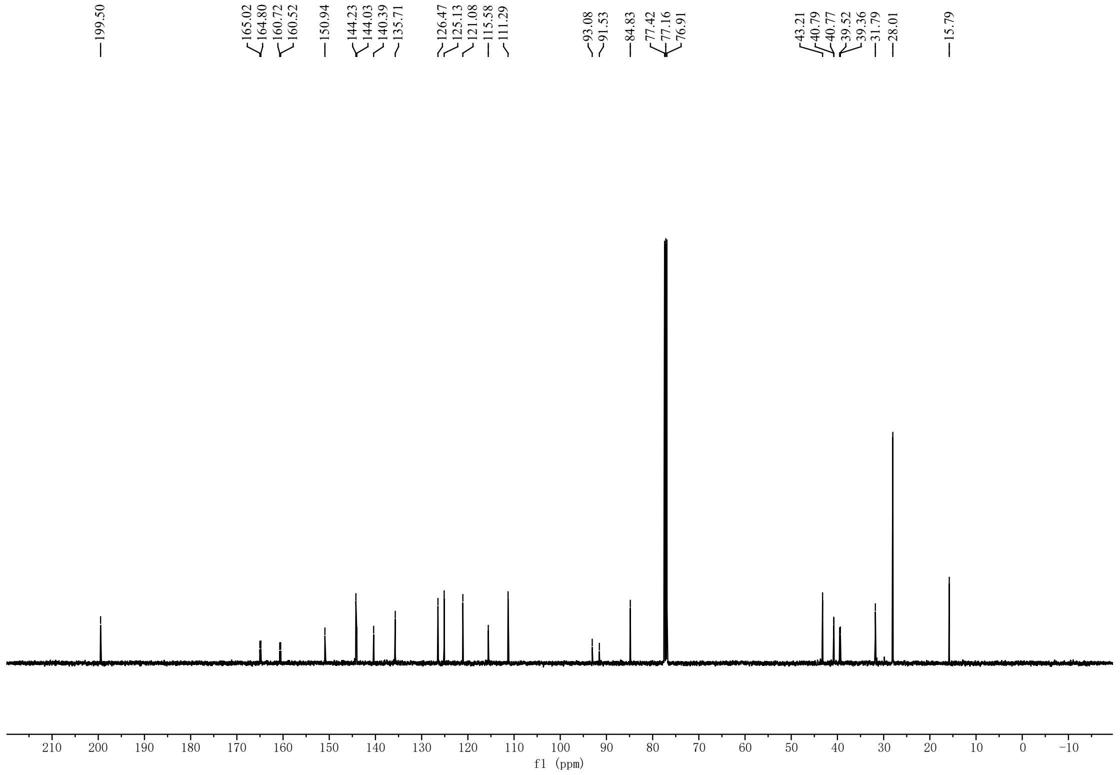

^{19}F NMR spectrum of **3u**

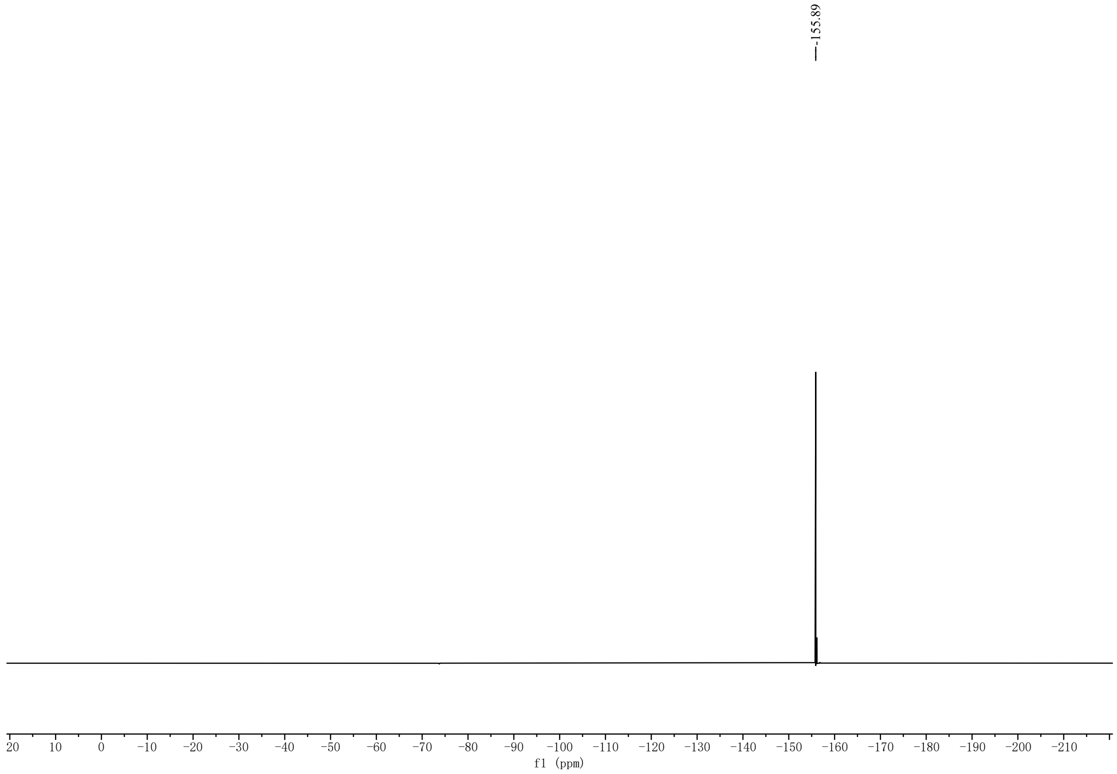

^{1}H **NMR** spectrum of **3v** (d.r. = 9 : 1)

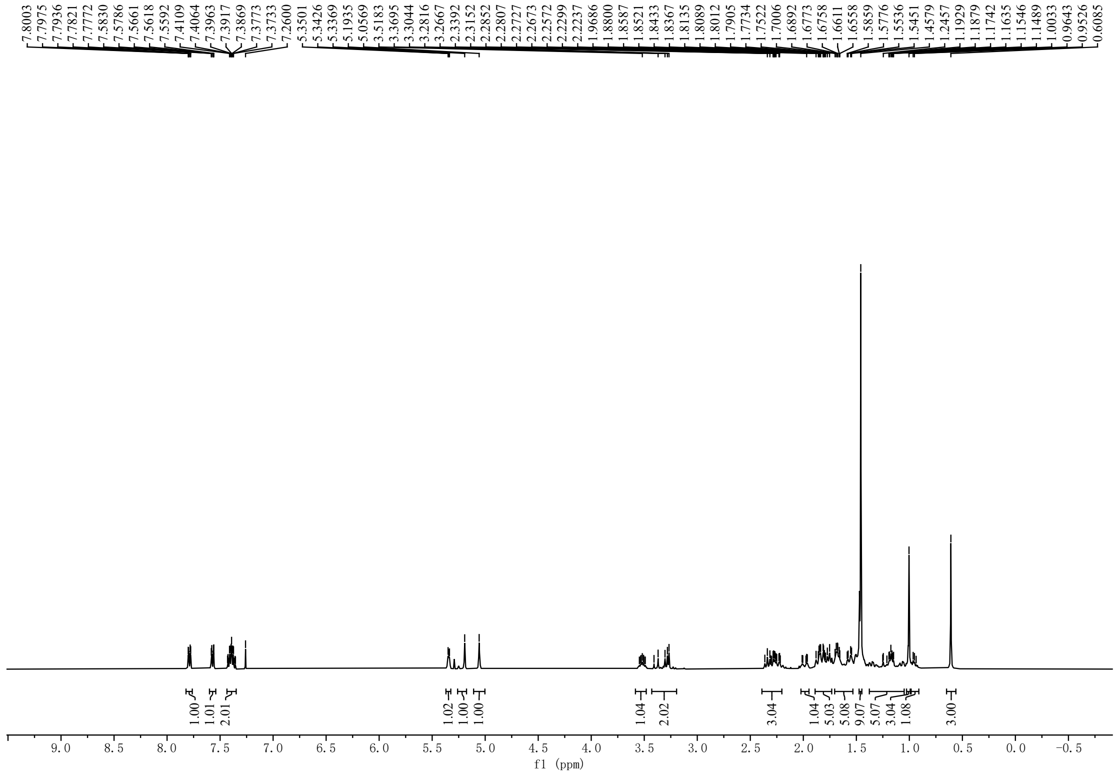

^{13}C **NMR** spectrum of **3v**

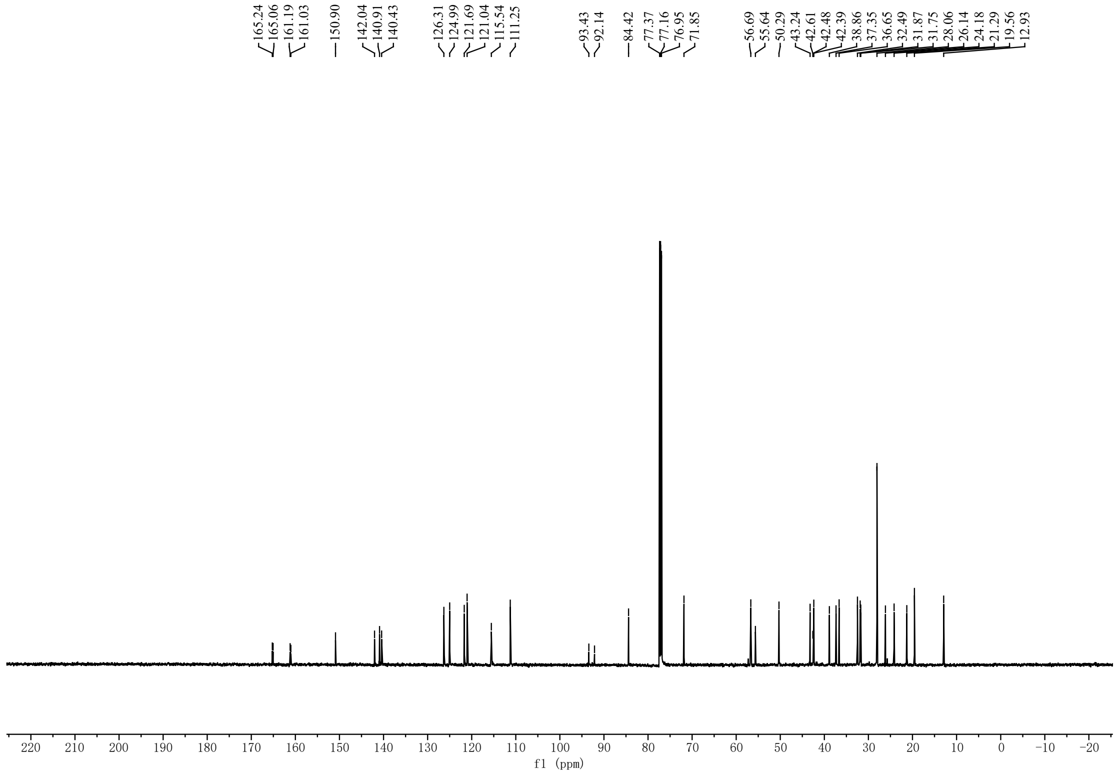

^{19}F NMR spectrum of **3v**

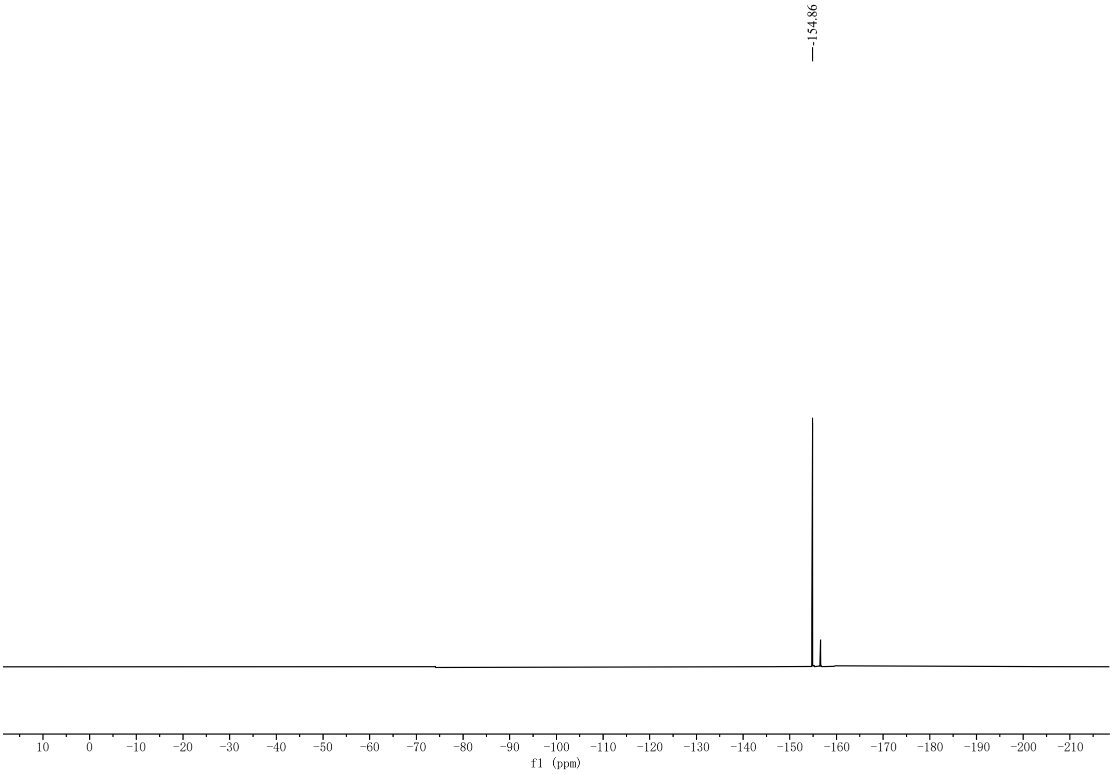

^{1}H NMR spectrum of **3w** (d.r. = 1 : 1)

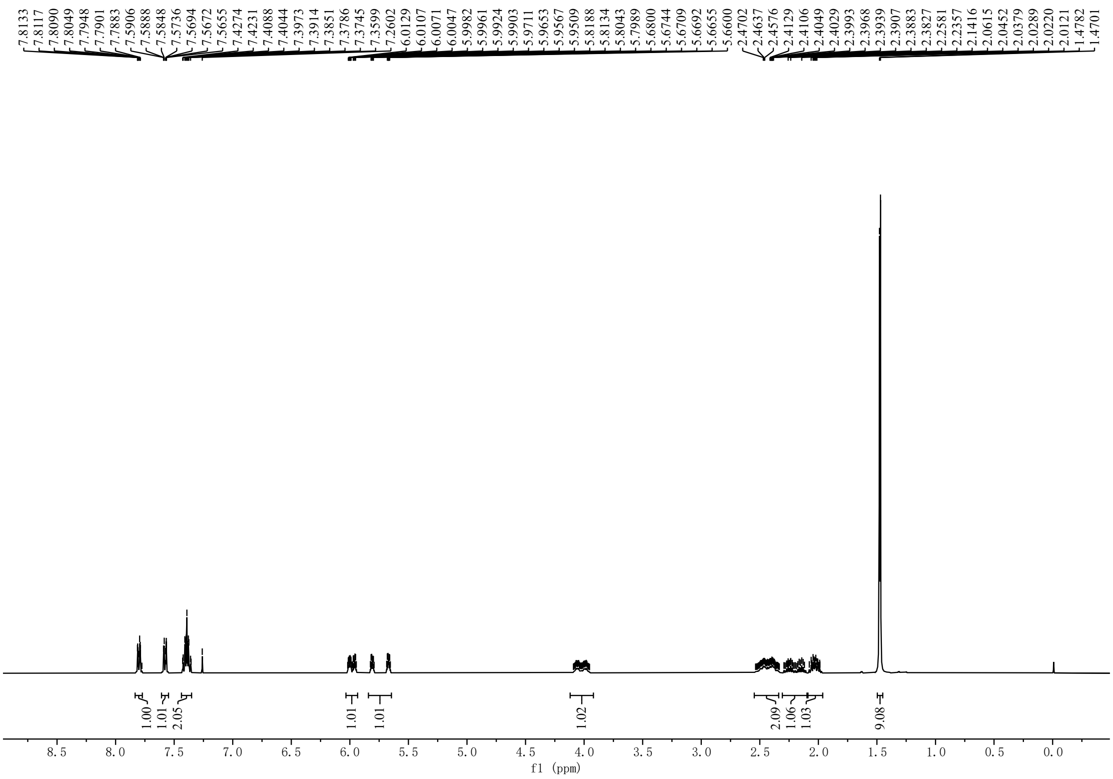

^{13}C NMR spectrum of **3w**

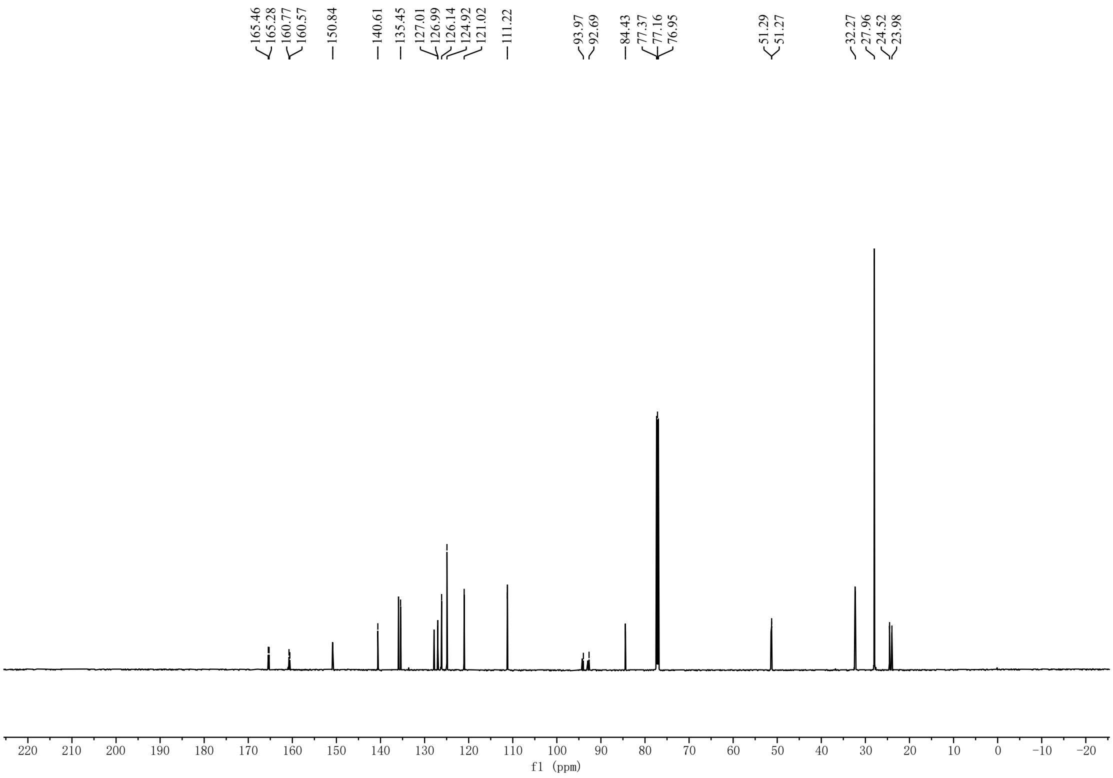

^{19}F NMR spectrum of **3w**

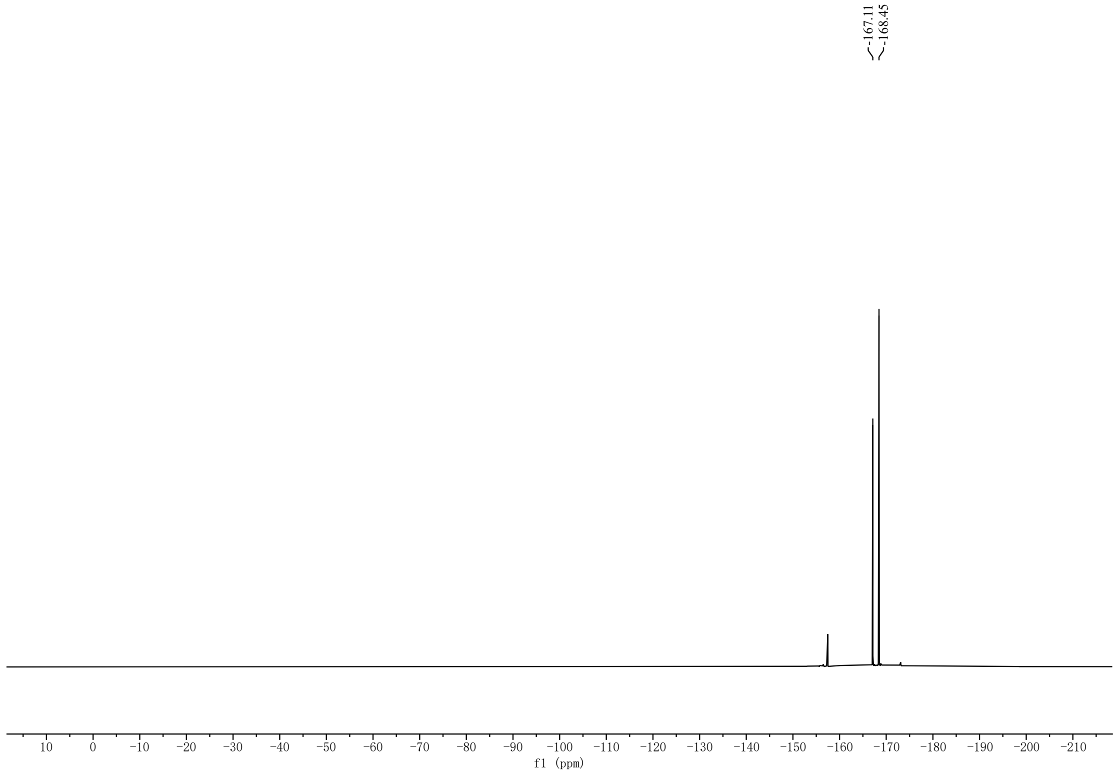

^{1}H NMR spectrum of 3x

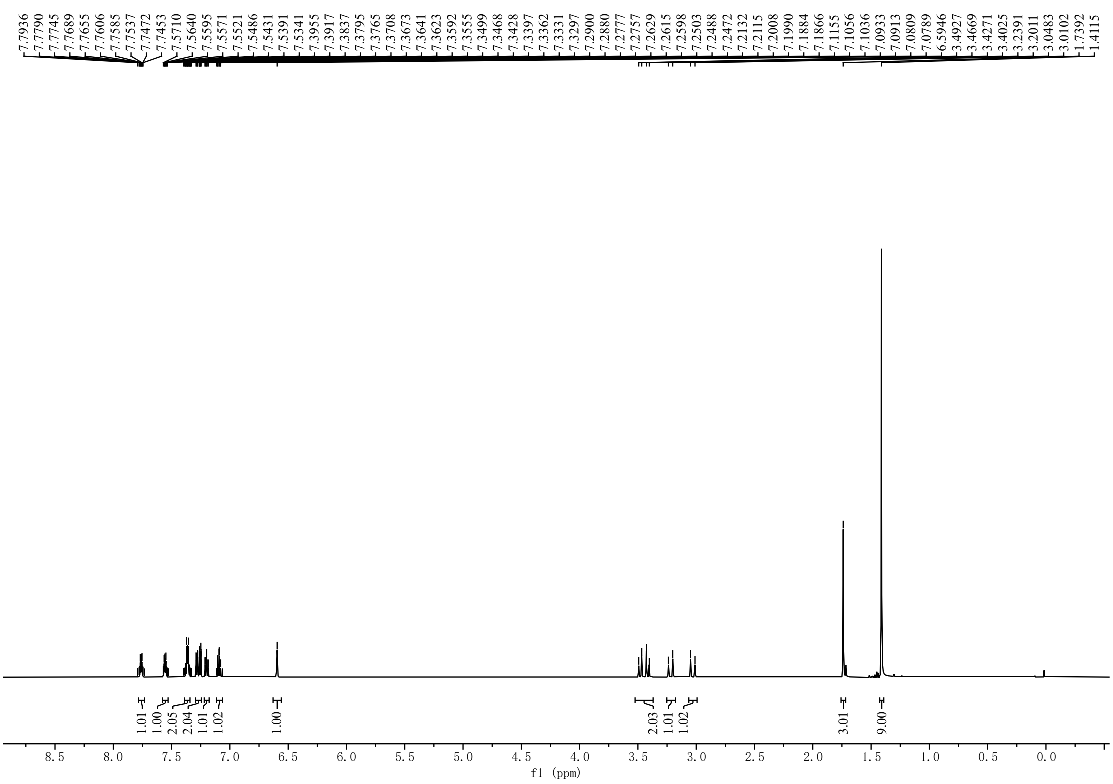

^{13}C NMR spectrum of 3x

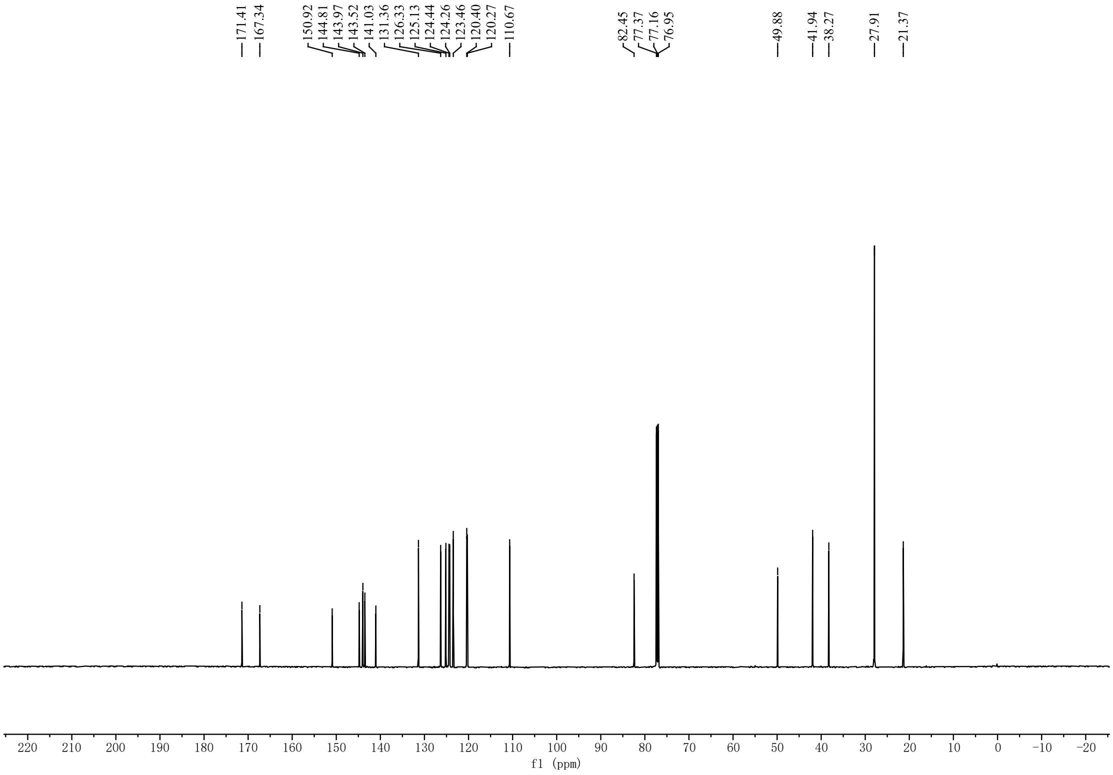

^{1}H NMR spectrum of **3y**

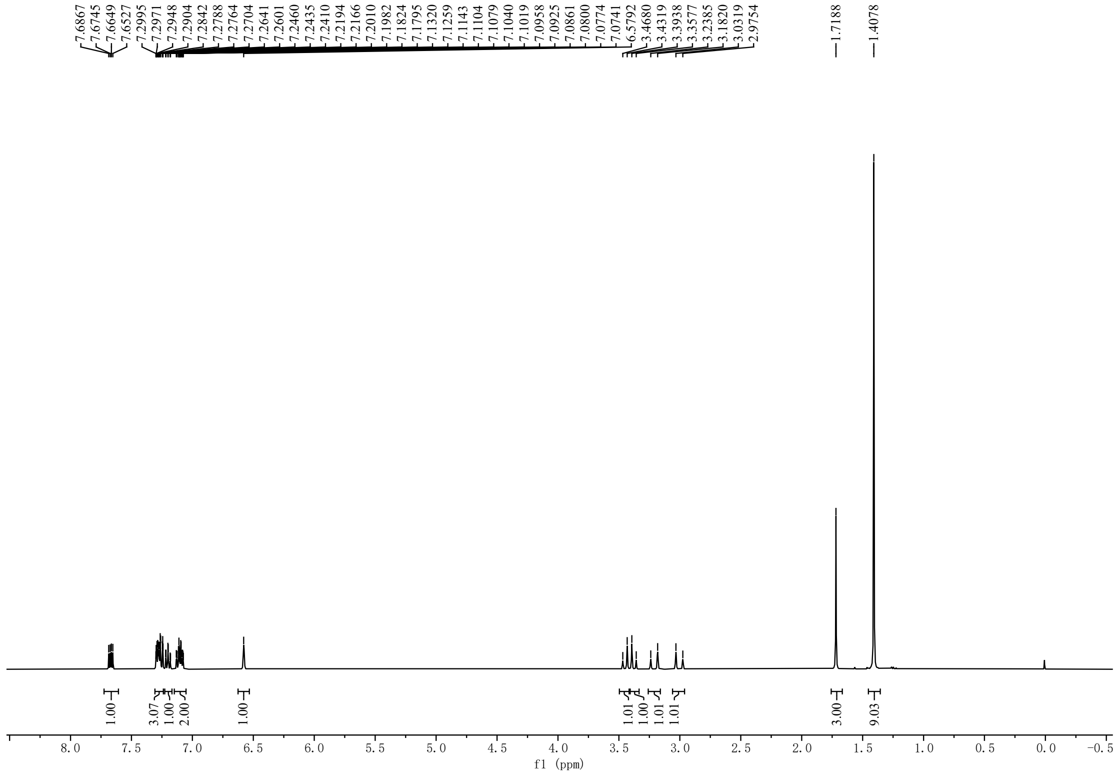

^{13}C NMR spectrum of **3y**

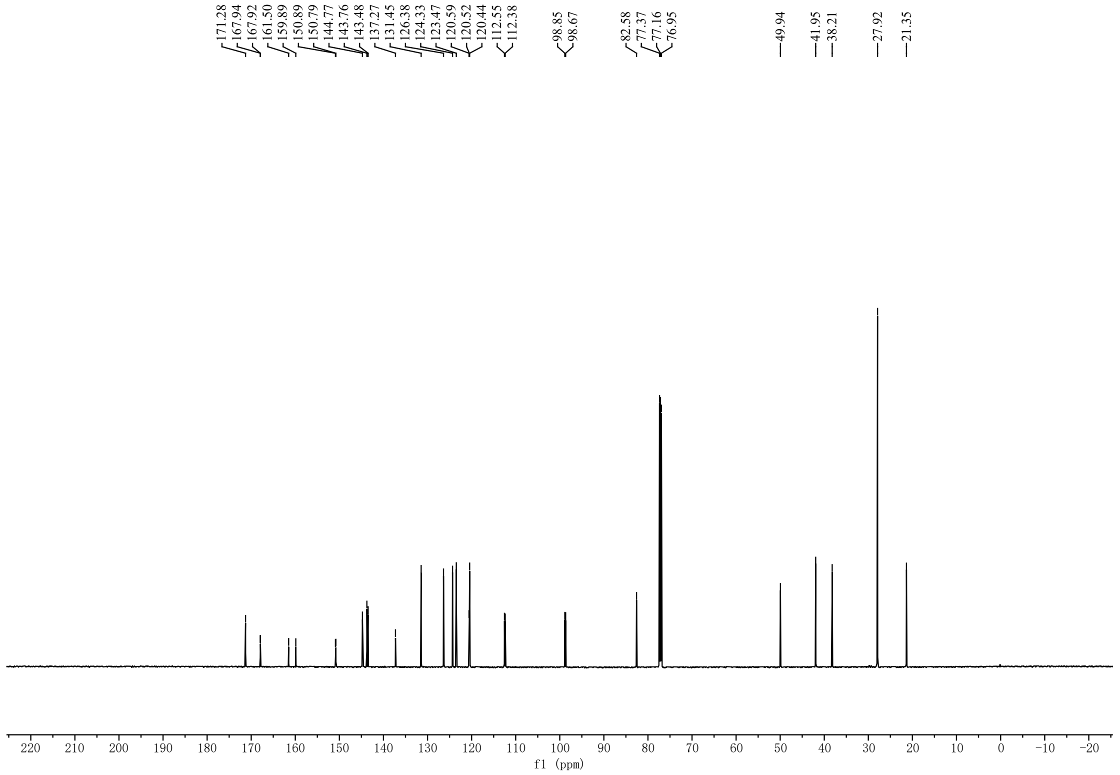

^{19}F NMR spectrum of **3y**

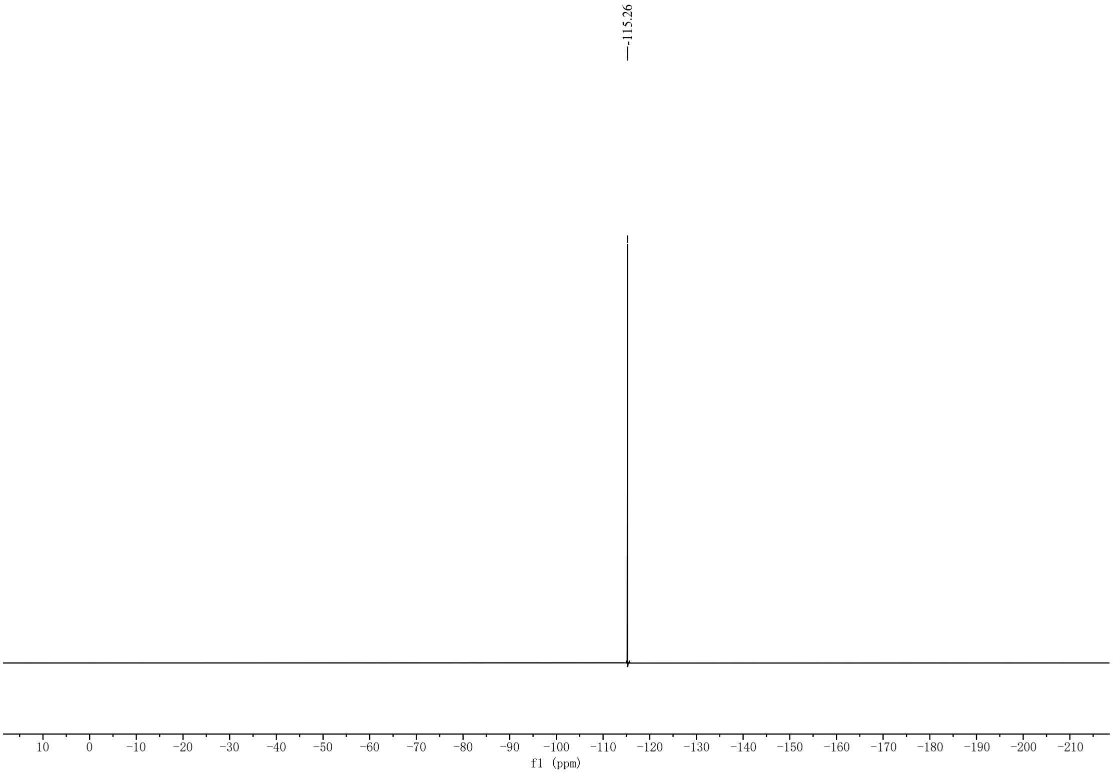

^{1}H NMR spectrum of **3y'**

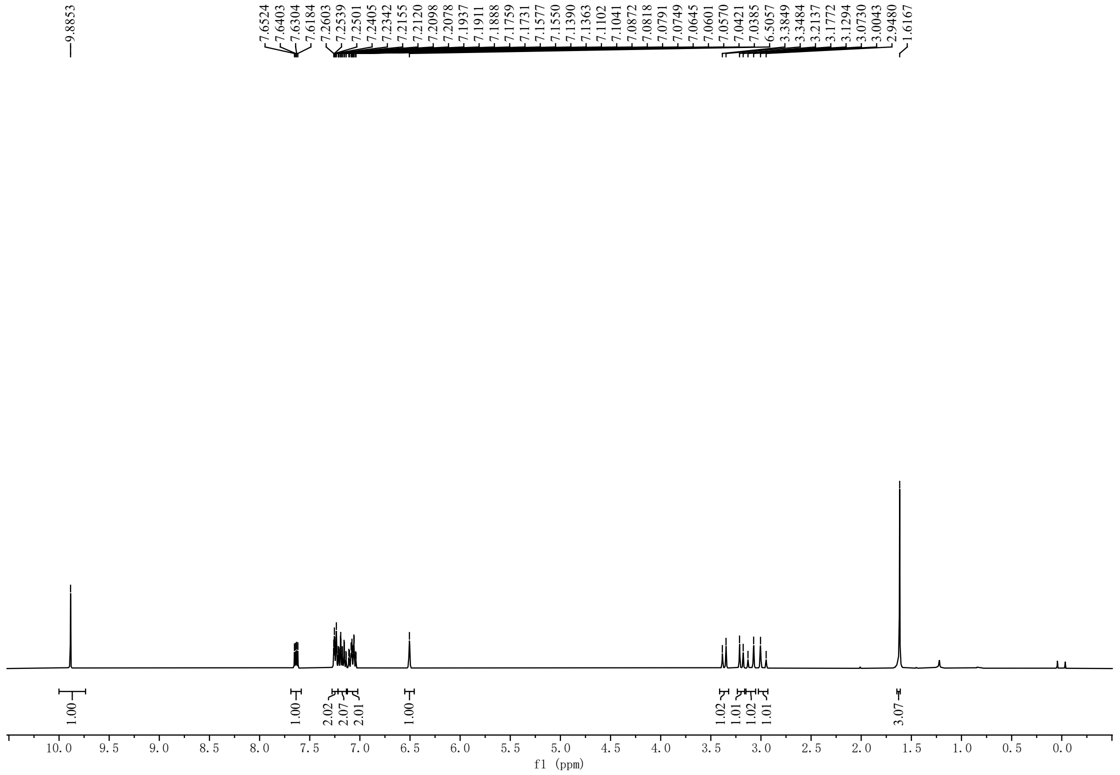

^{13}C NMR spectrum of **3y'**

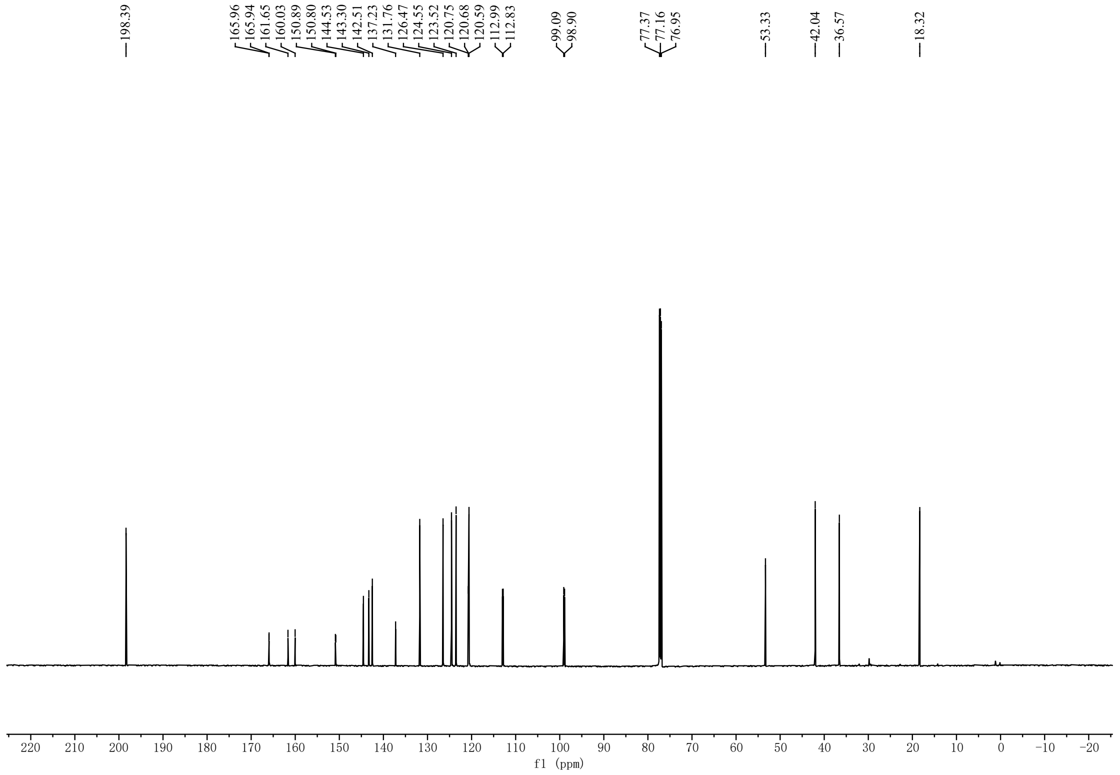

^{19}F NMR spectrum of **3y'**

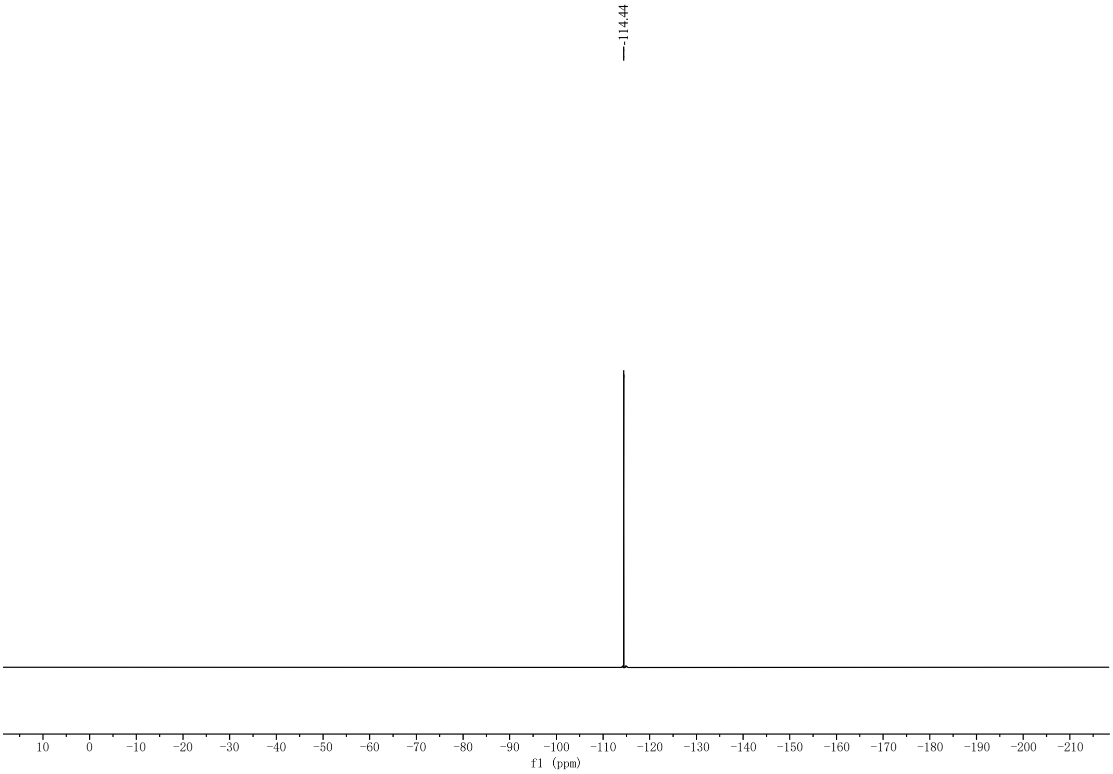

^{1}H NMR spectrum of **3z**

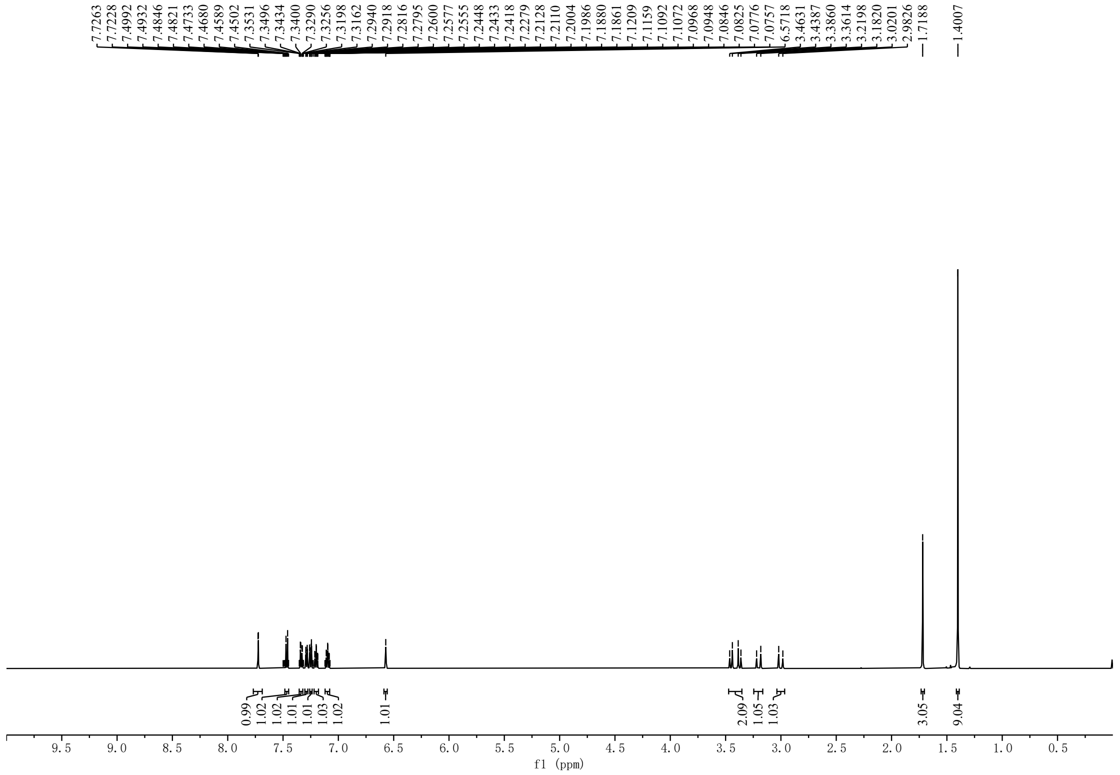

^{13}C NMR spectrum of **3z**

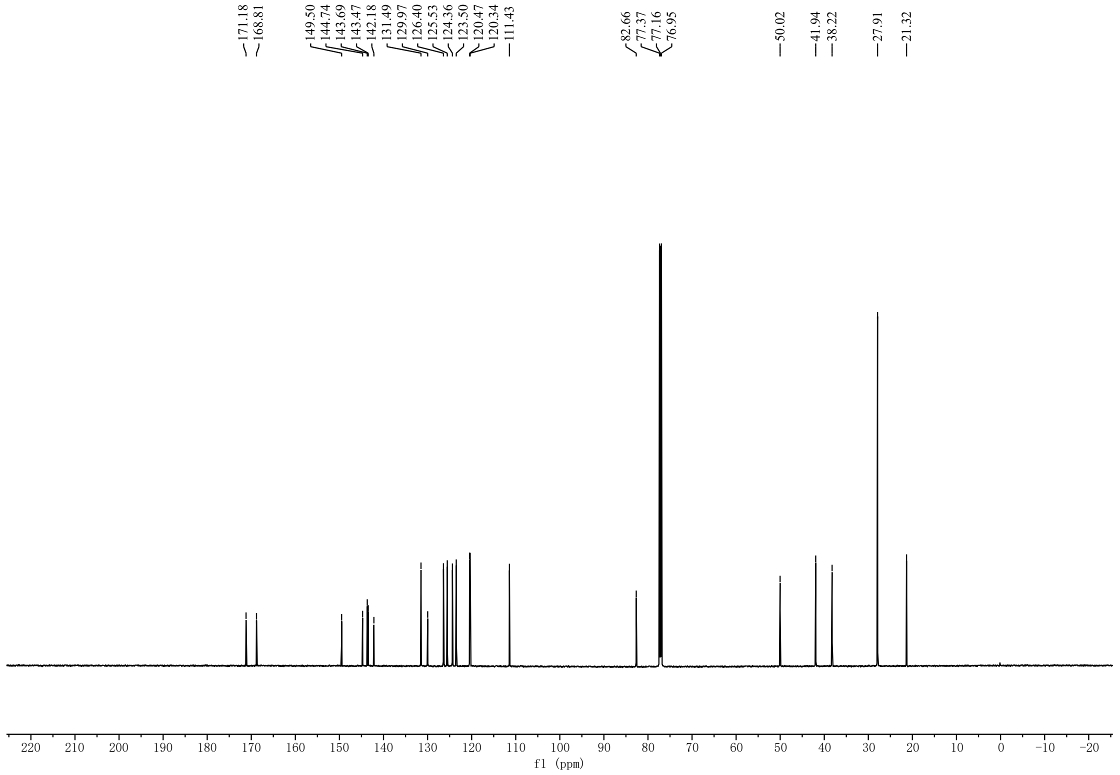

^{1}H NMR spectrum of **3aa**

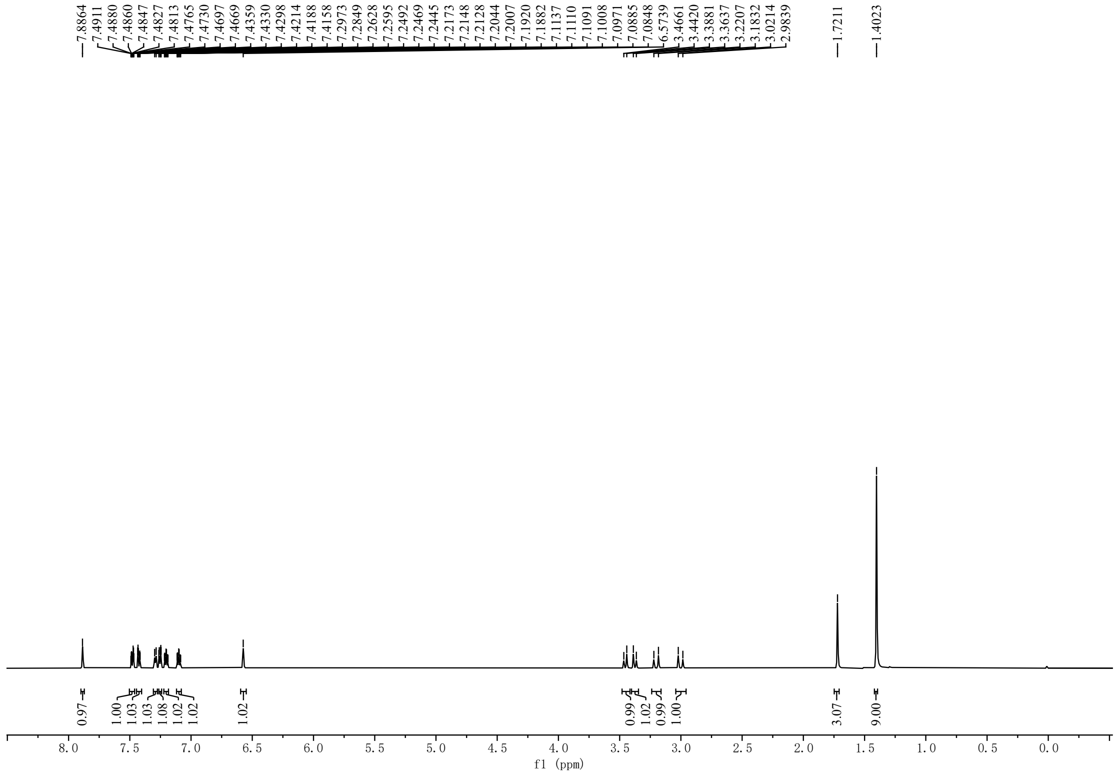

^{13}C NMR spectrum of **3aa**

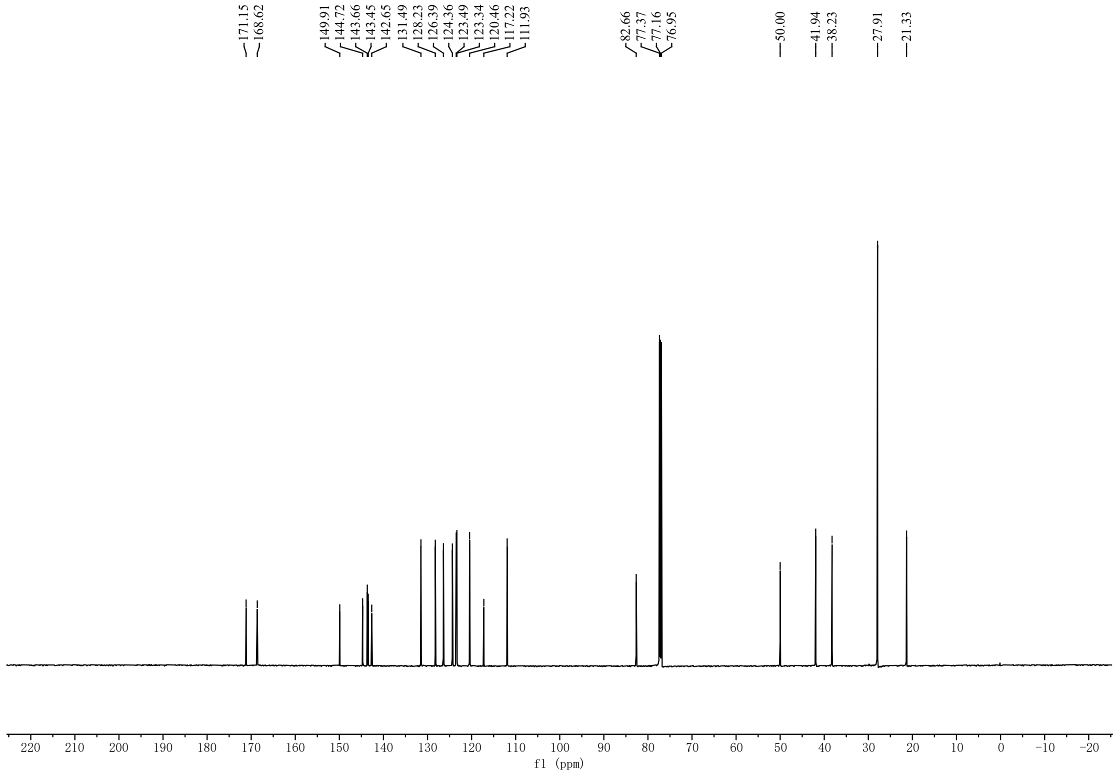

^{1}H NMR spectrum of **3ab**

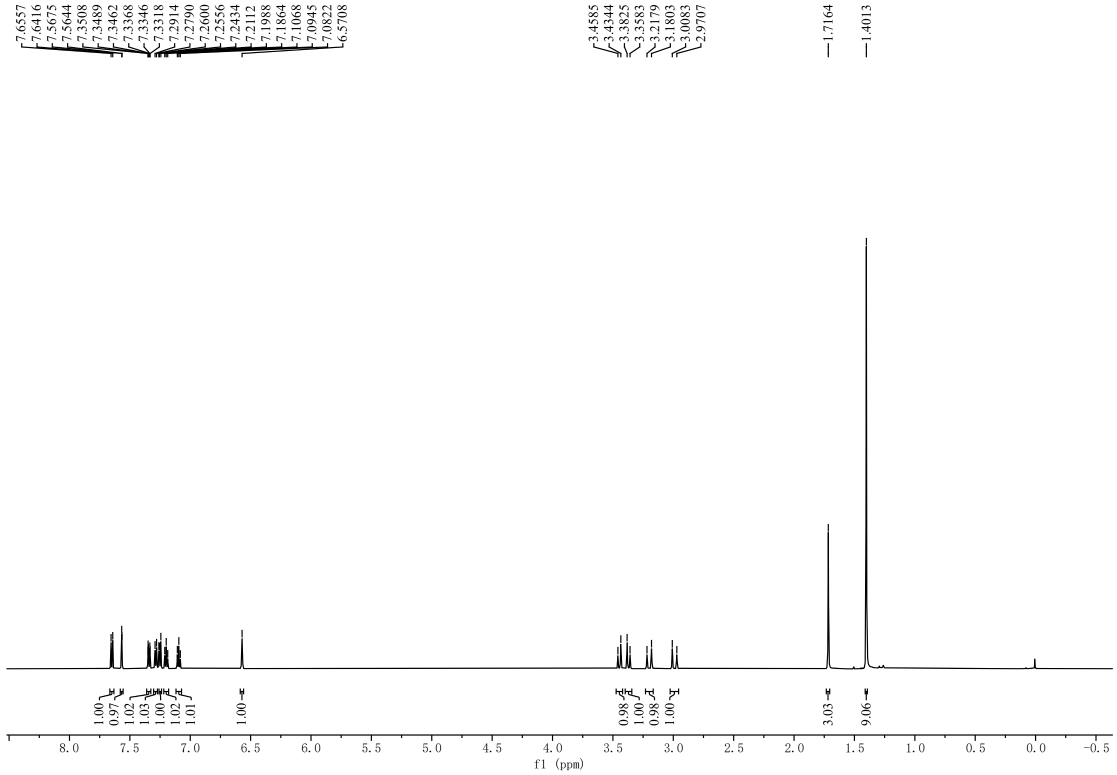

^{13}C NMR spectrum of **3ab**

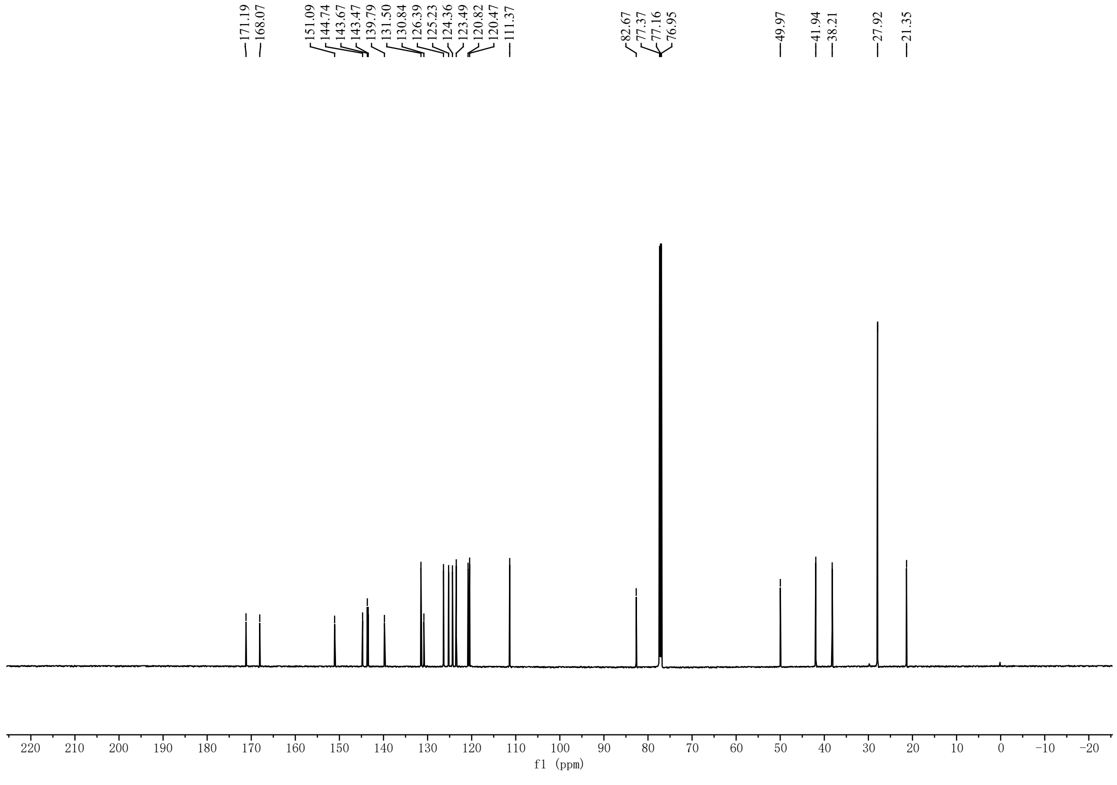

^{1}H NMR spectrum of **3ac**

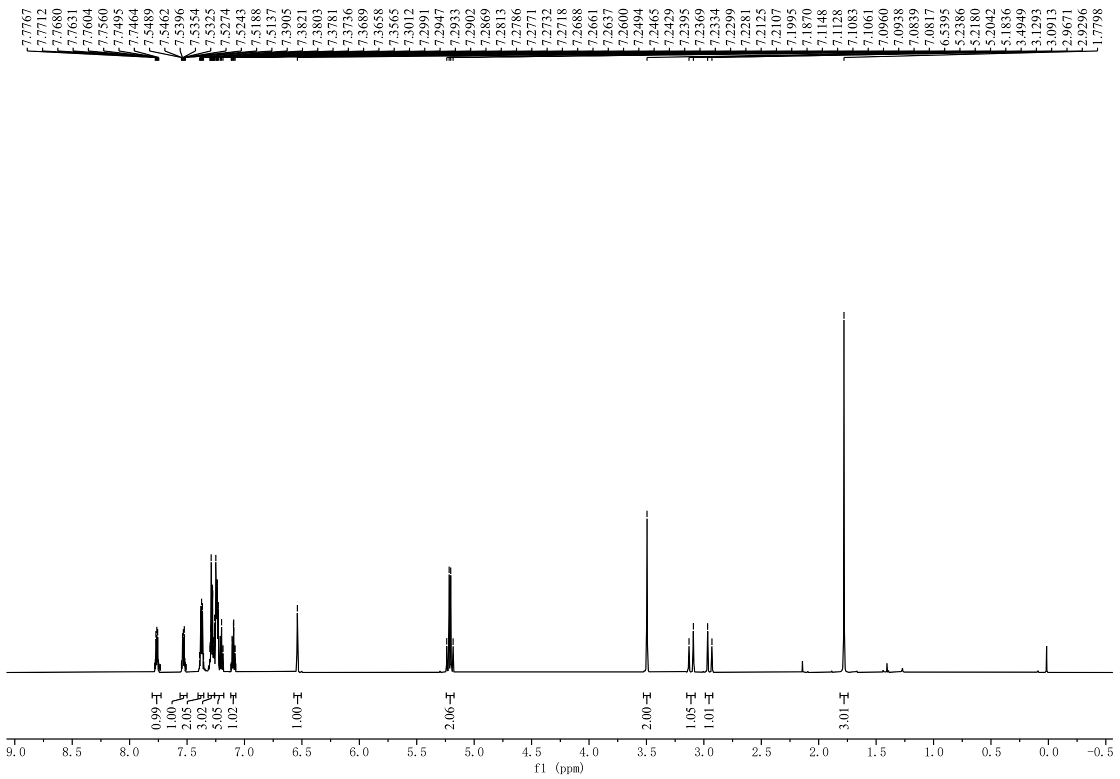

^{13}C NMR spectrum of **3ac**

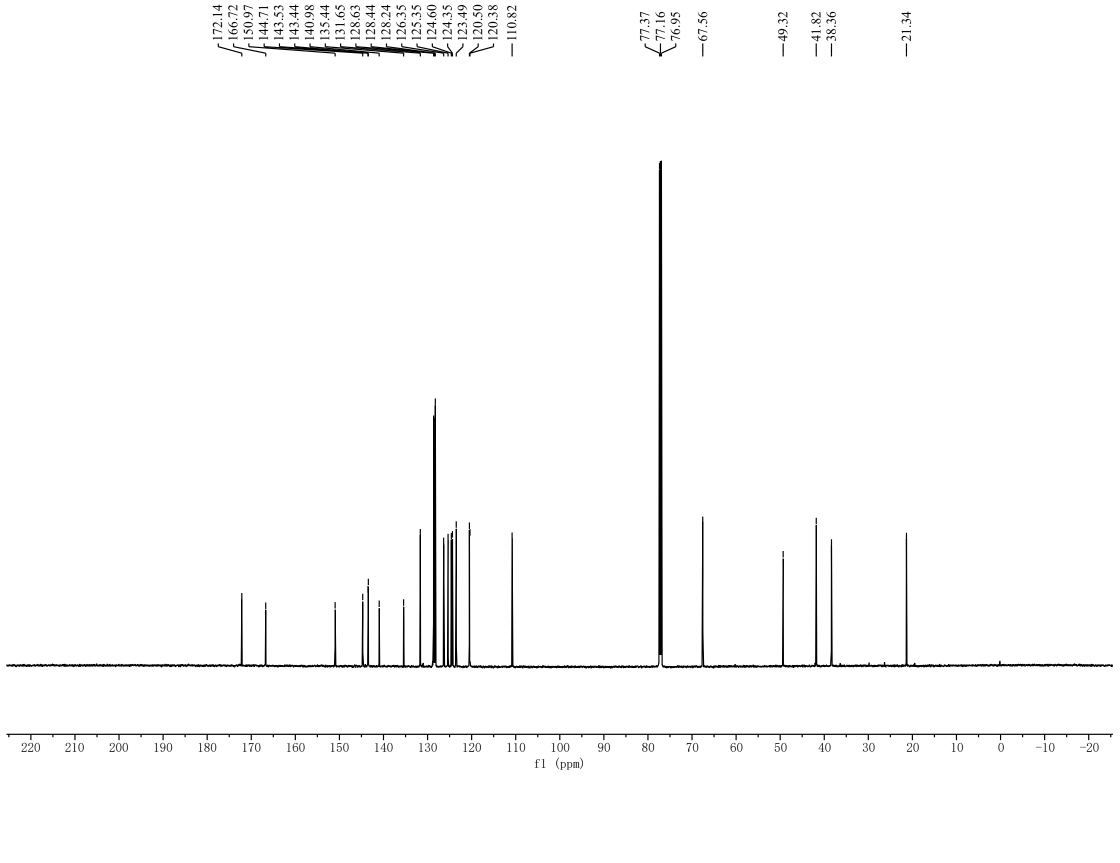

^{1}H NMR spectrum of **3ad**

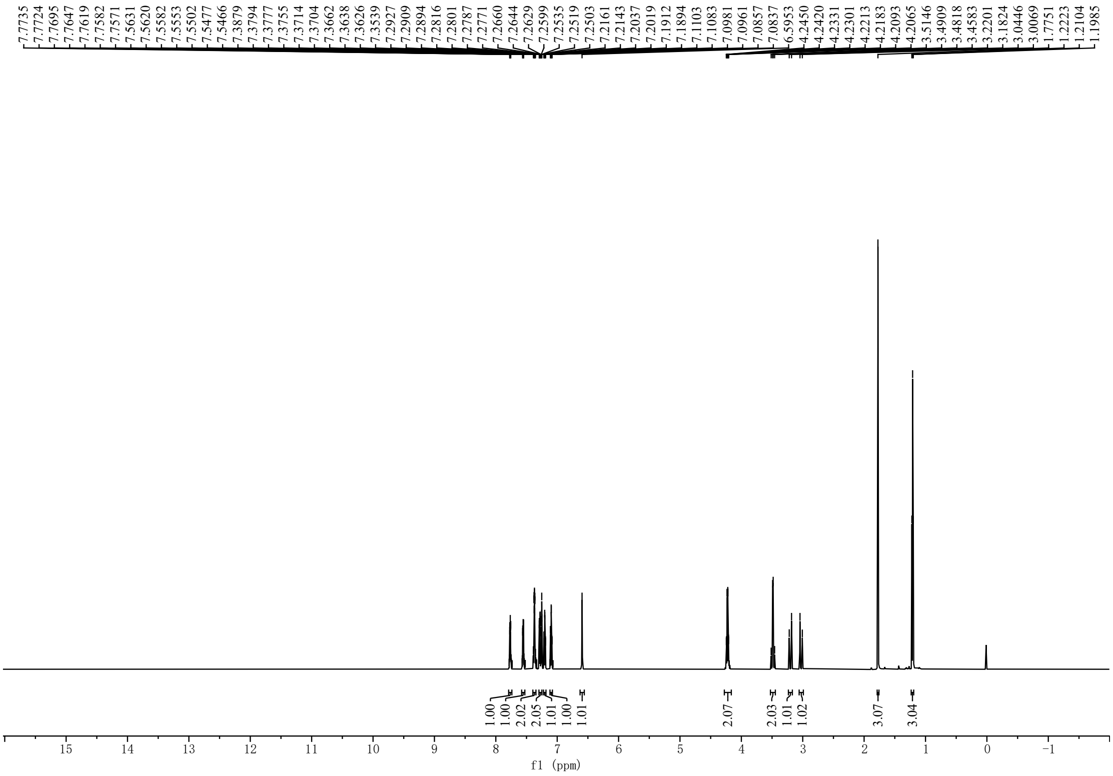

^{13}C NMR spectrum of **3ad**

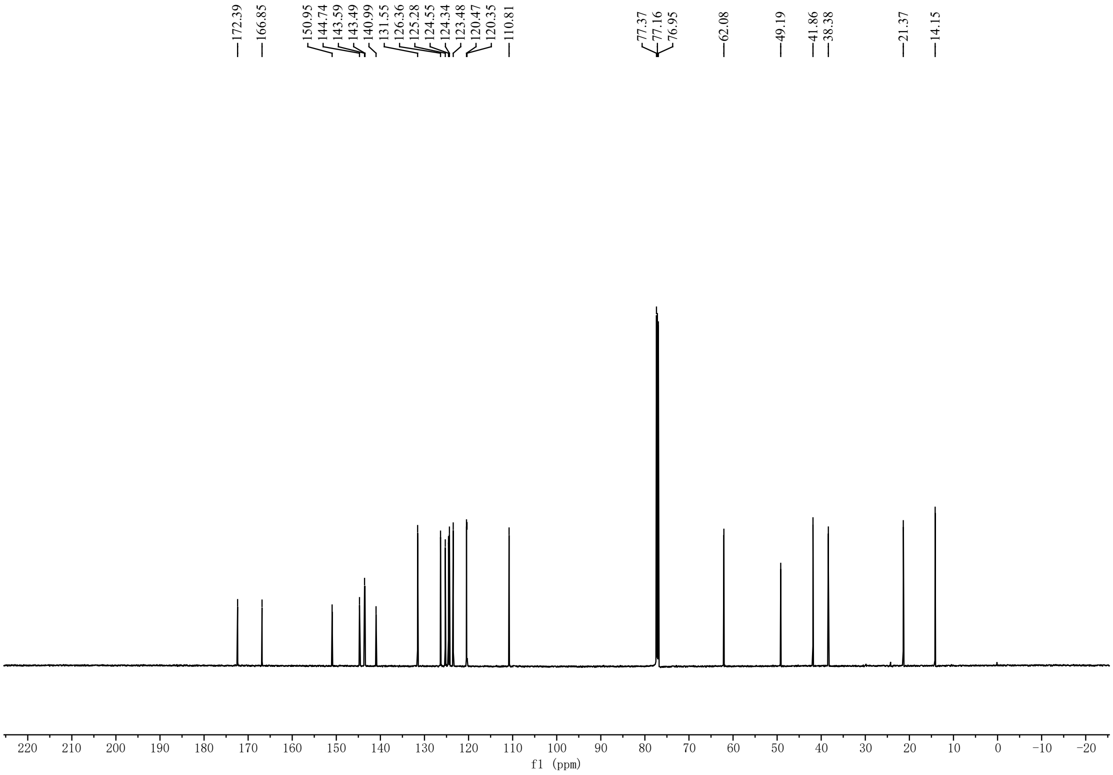

^{1}H NMR spectrum of **3ae**

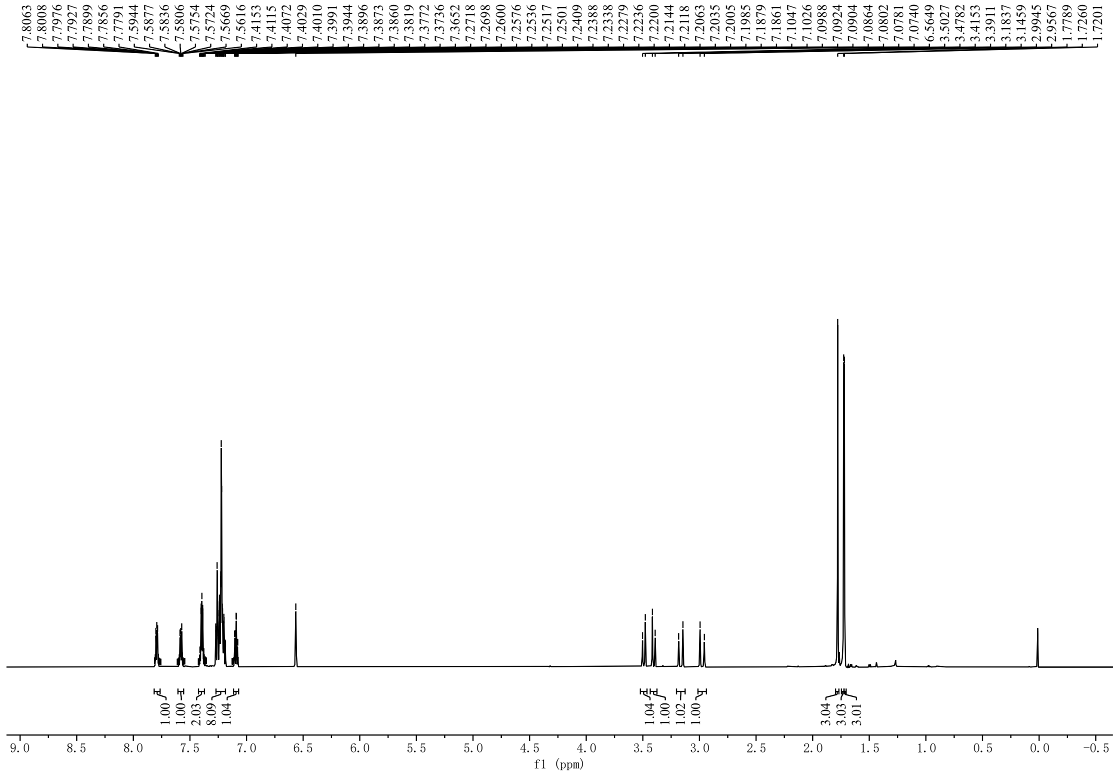

^{13}C NMR spectrum of **3ae**

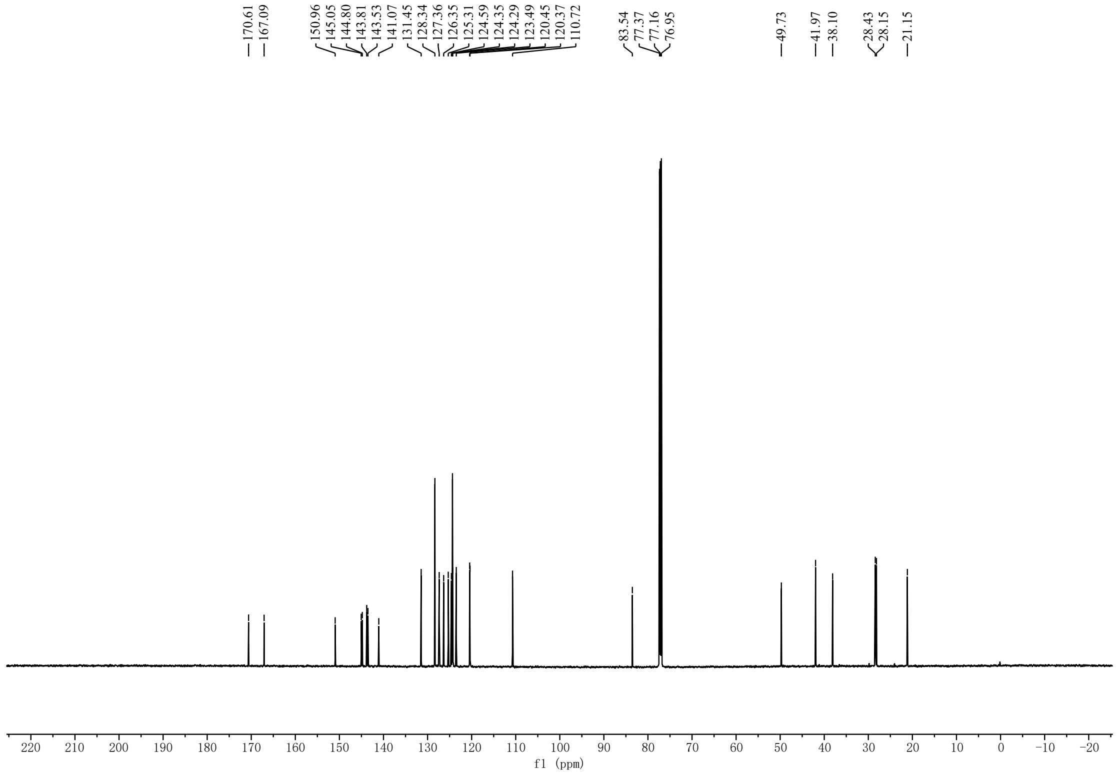

^{1}H NMR spectrum of **3af**

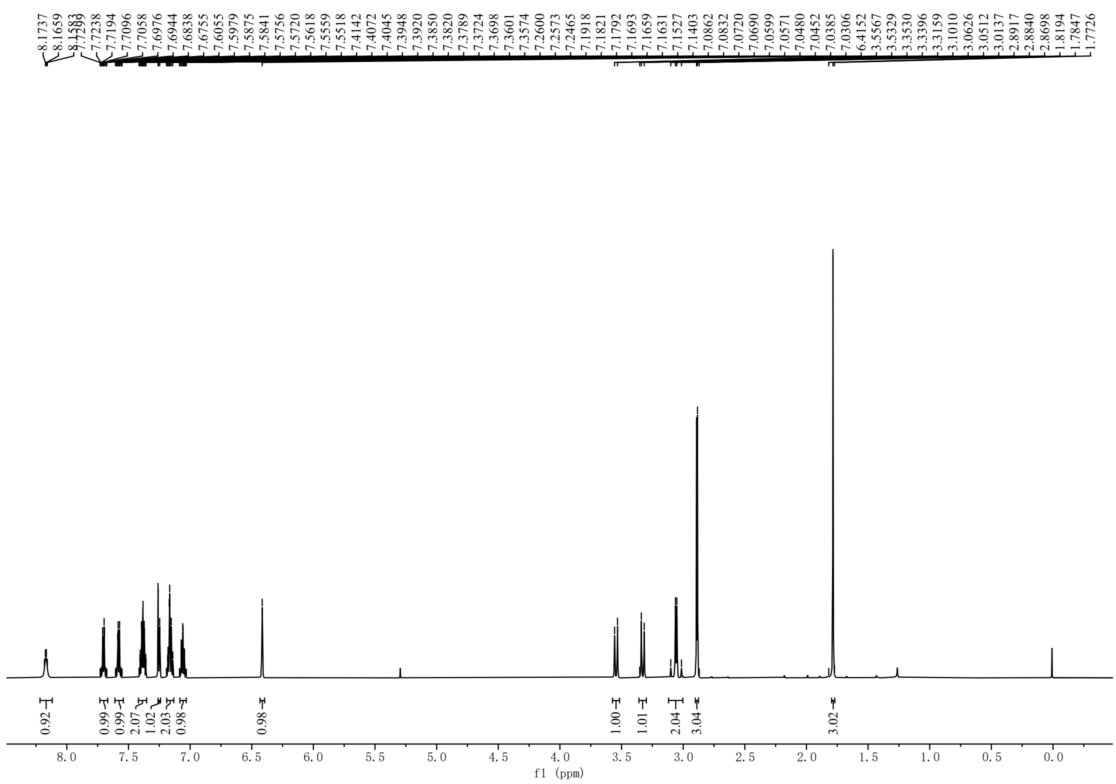

^{13}C NMR spectrum of **3af**

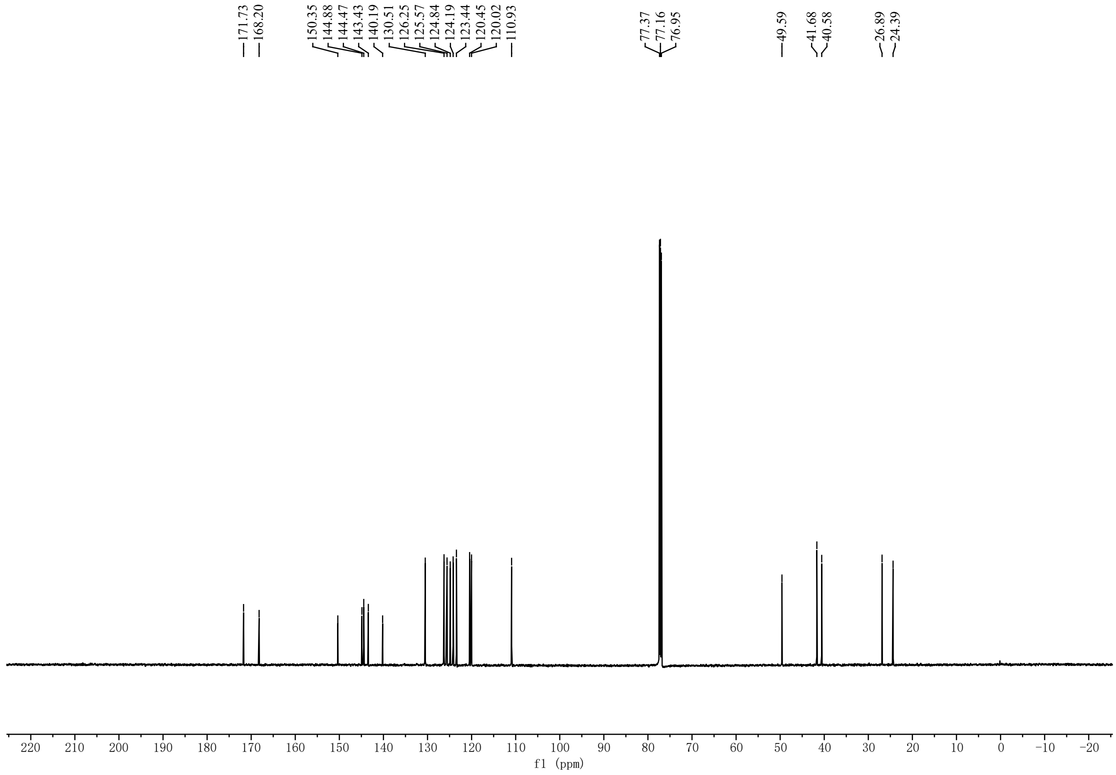

^{1}H NMR spectrum of **3ag**

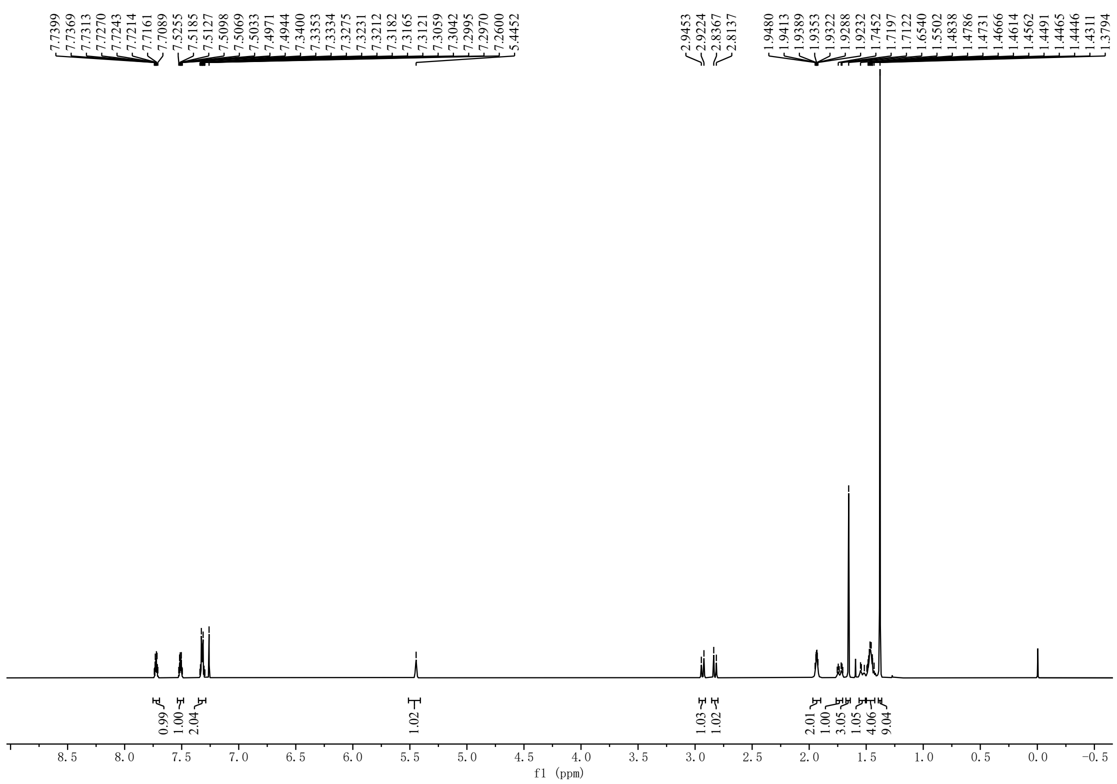

^{13}C NMR spectrum of **3ag**

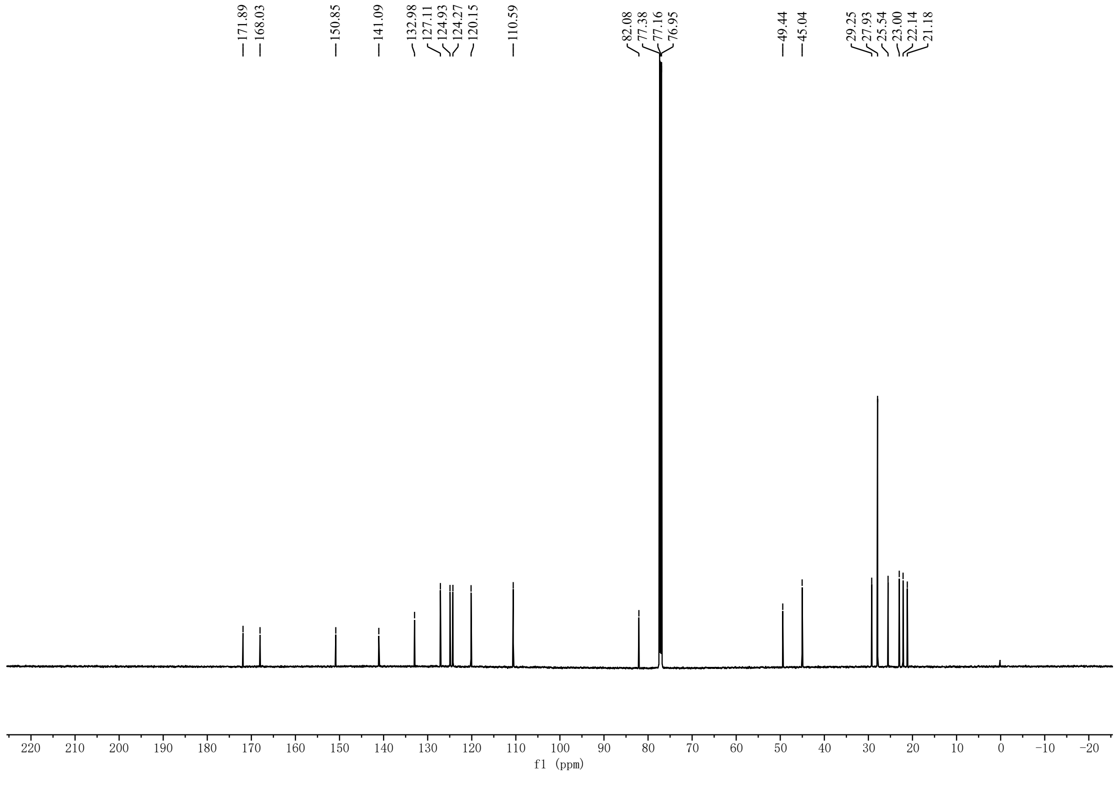

^{1}H NMR spectrum of **3ah**

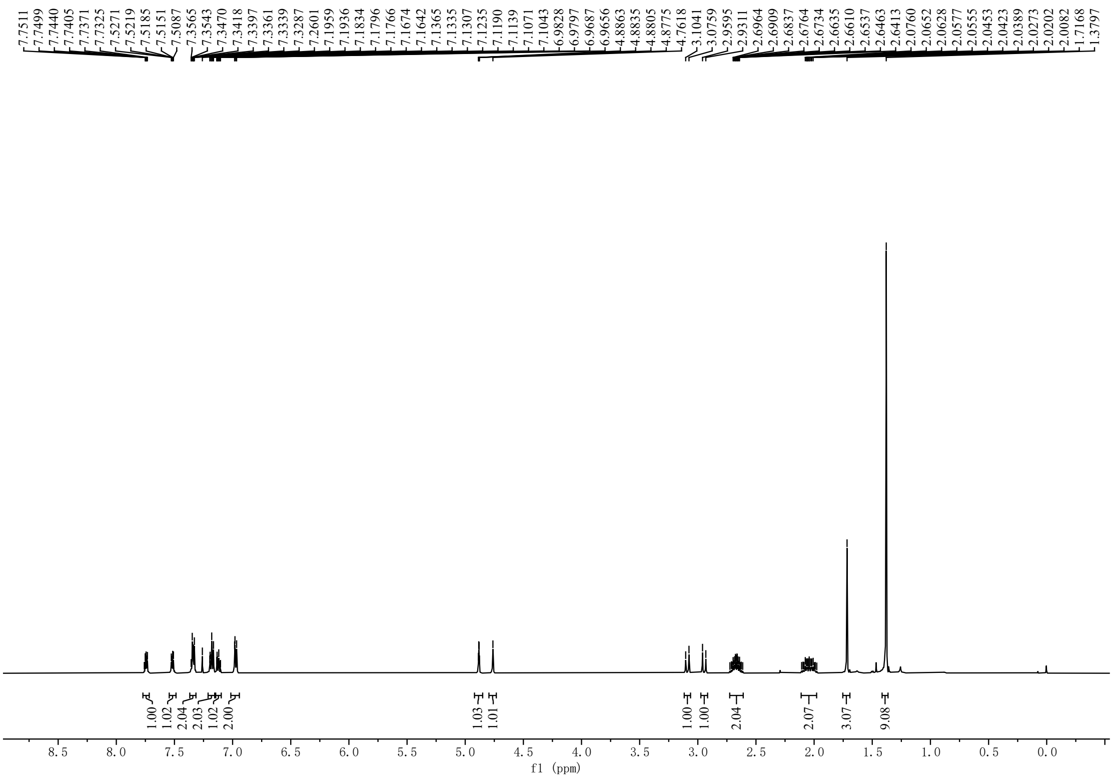

^{13}C NMR spectrum of **3ah**

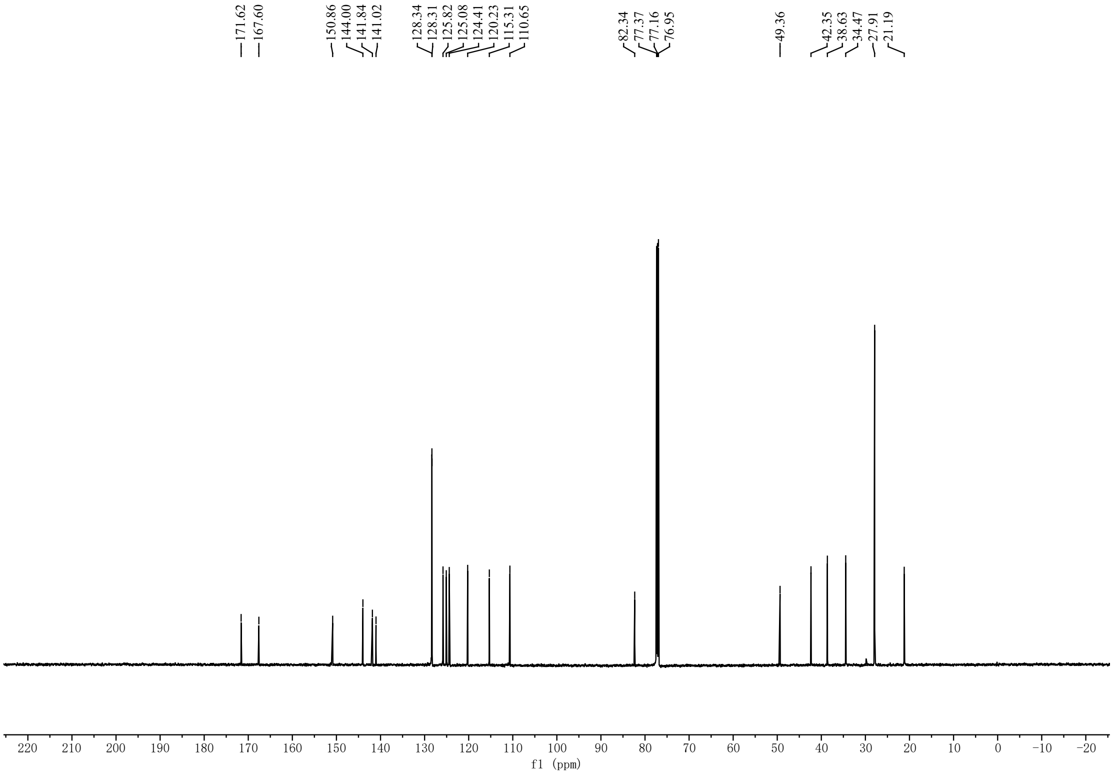

^{1}H NMR spectrum of **3ai**

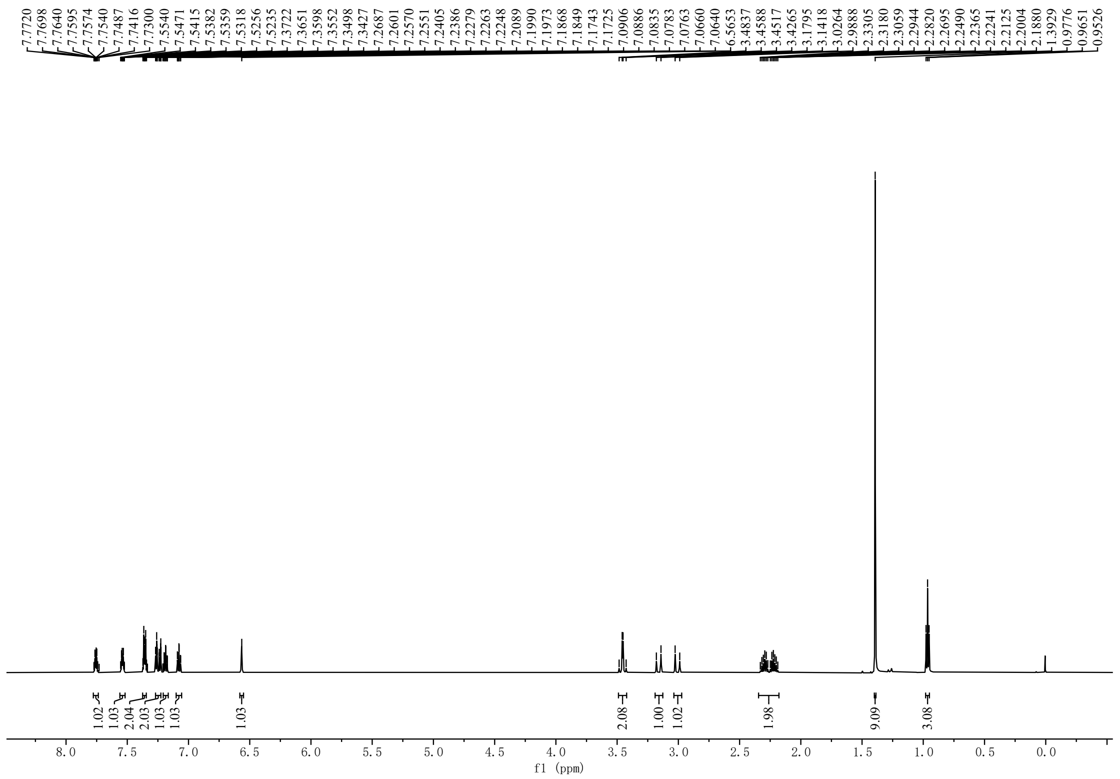

^{13}C NMR spectrum of **3ai**

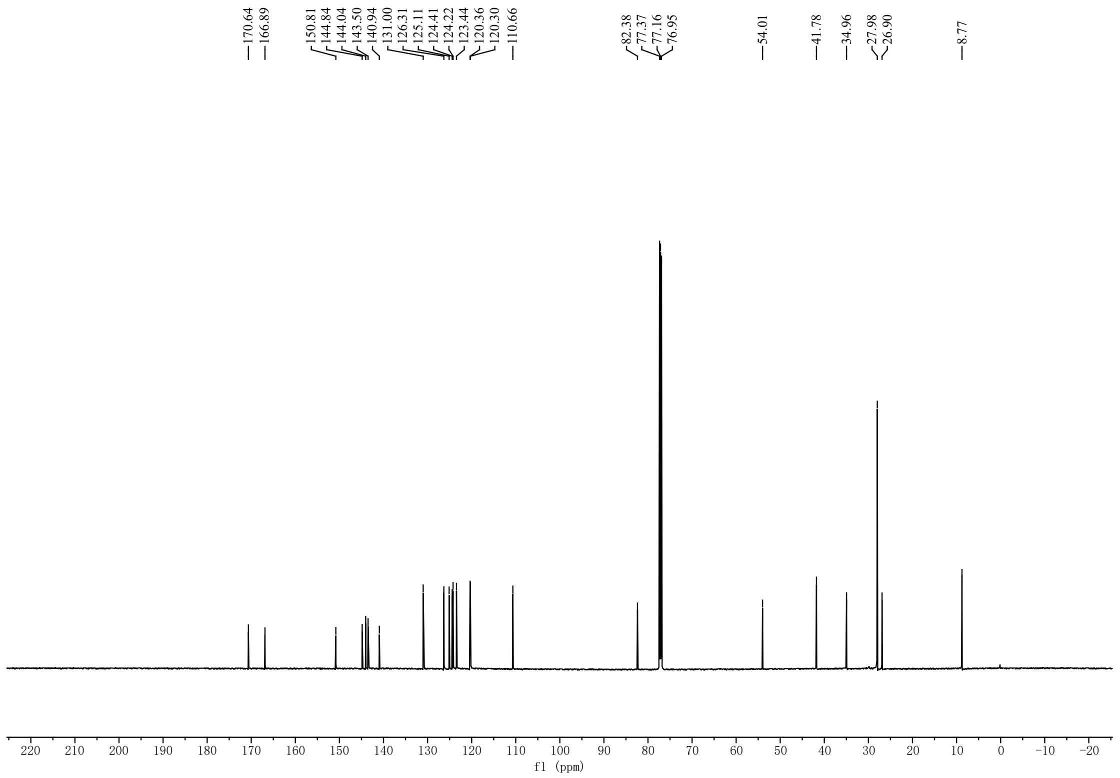

^{1}H NMR spectrum of **3aj**

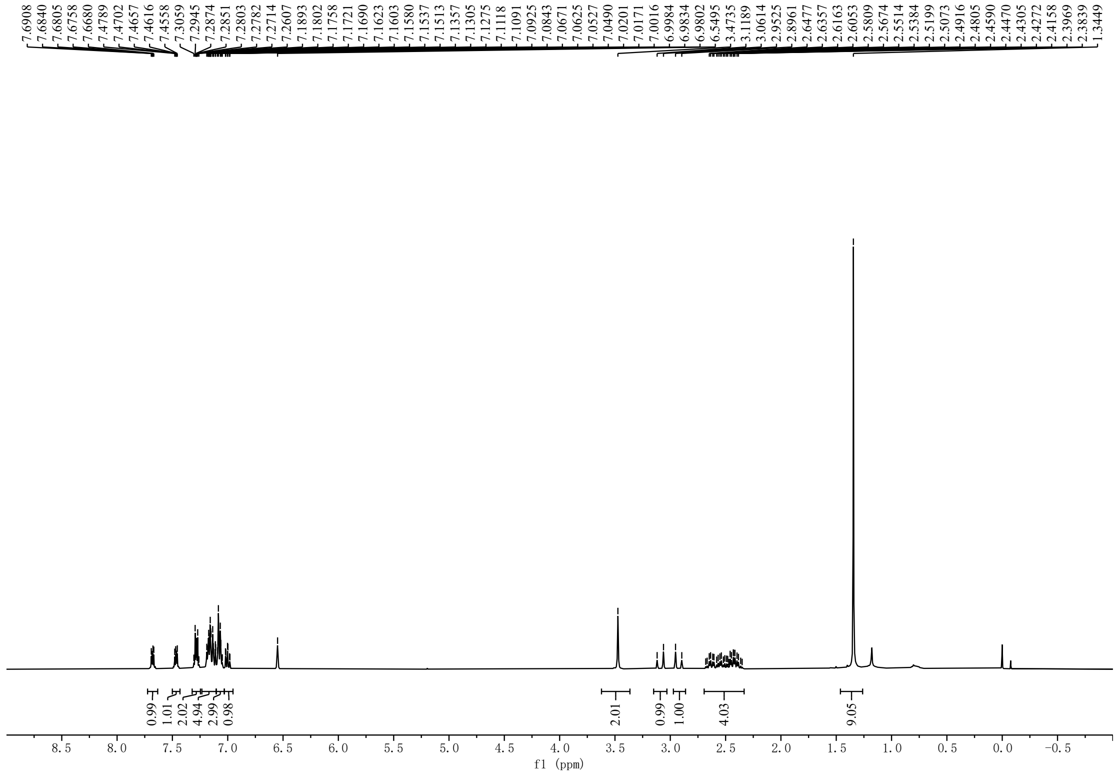

^{13}C NMR spectrum of **3aj**

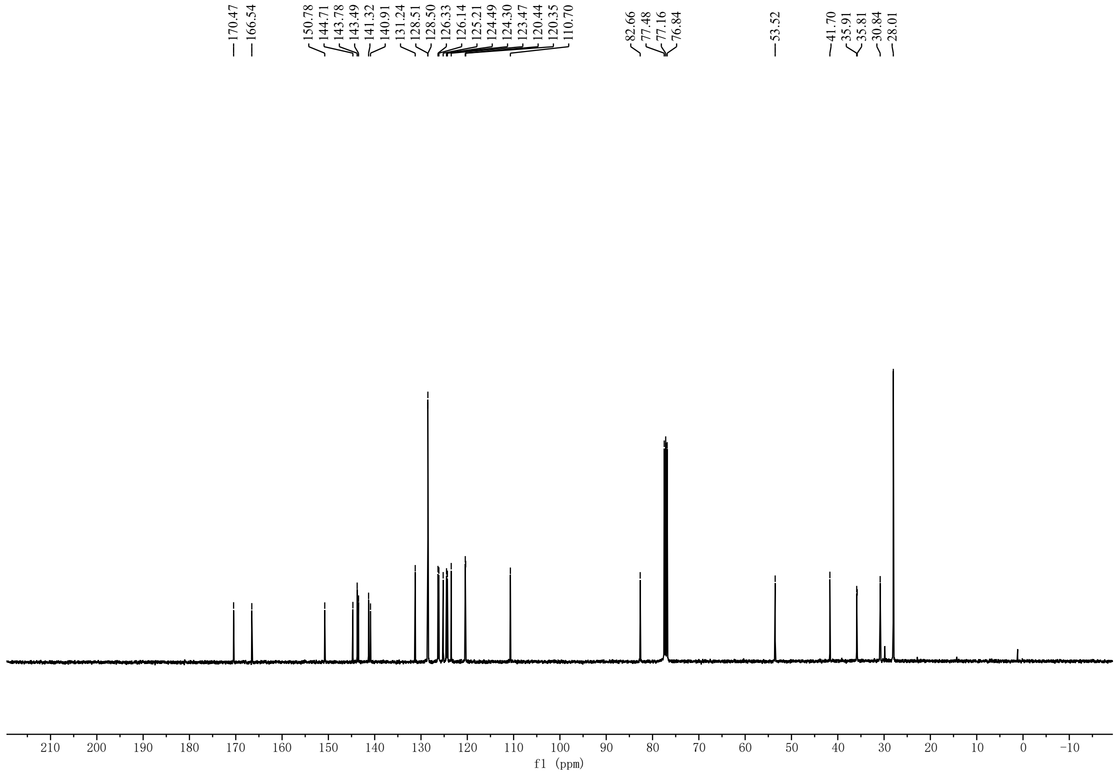

^{1}H NMR spectrum of **3ak**

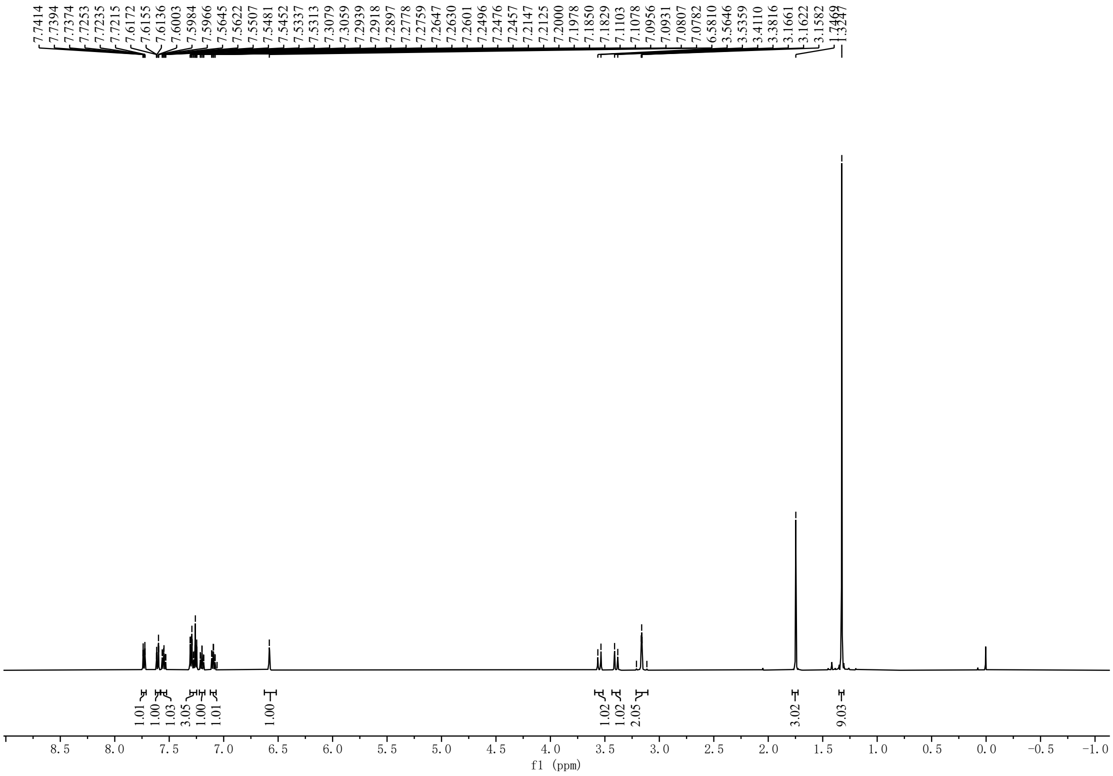

^{13}C NMR spectrum of **3ak**

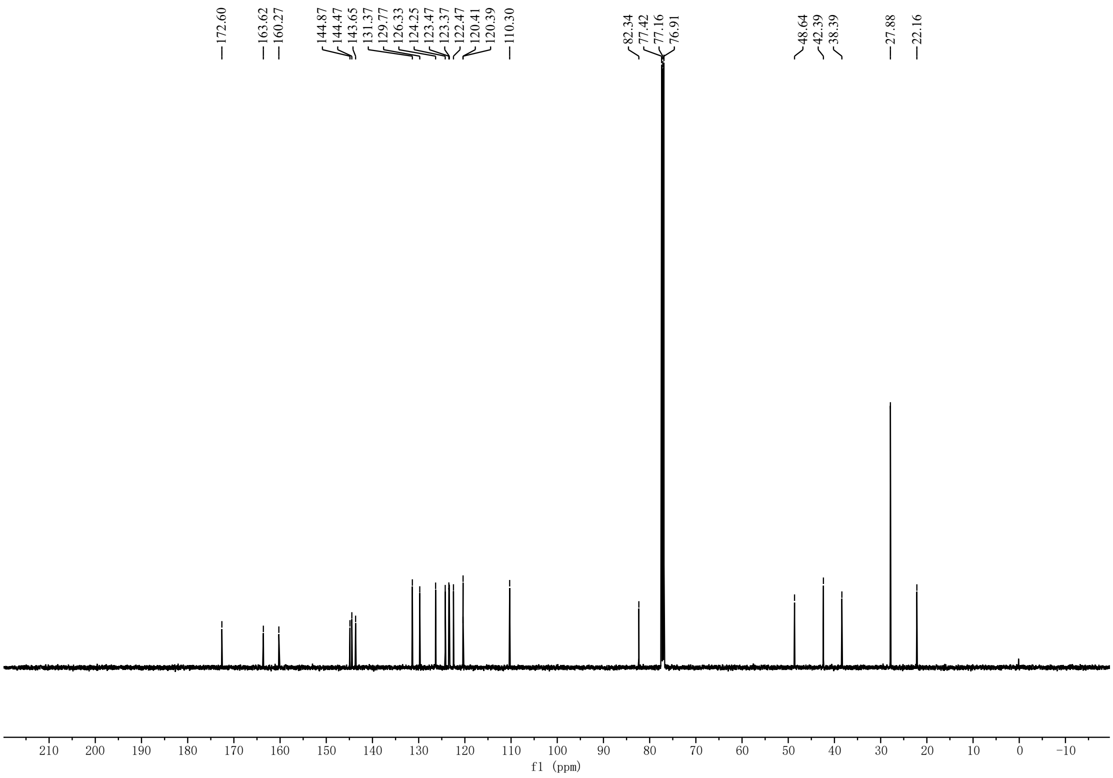

^{1}H NMR spectrum of **4a**

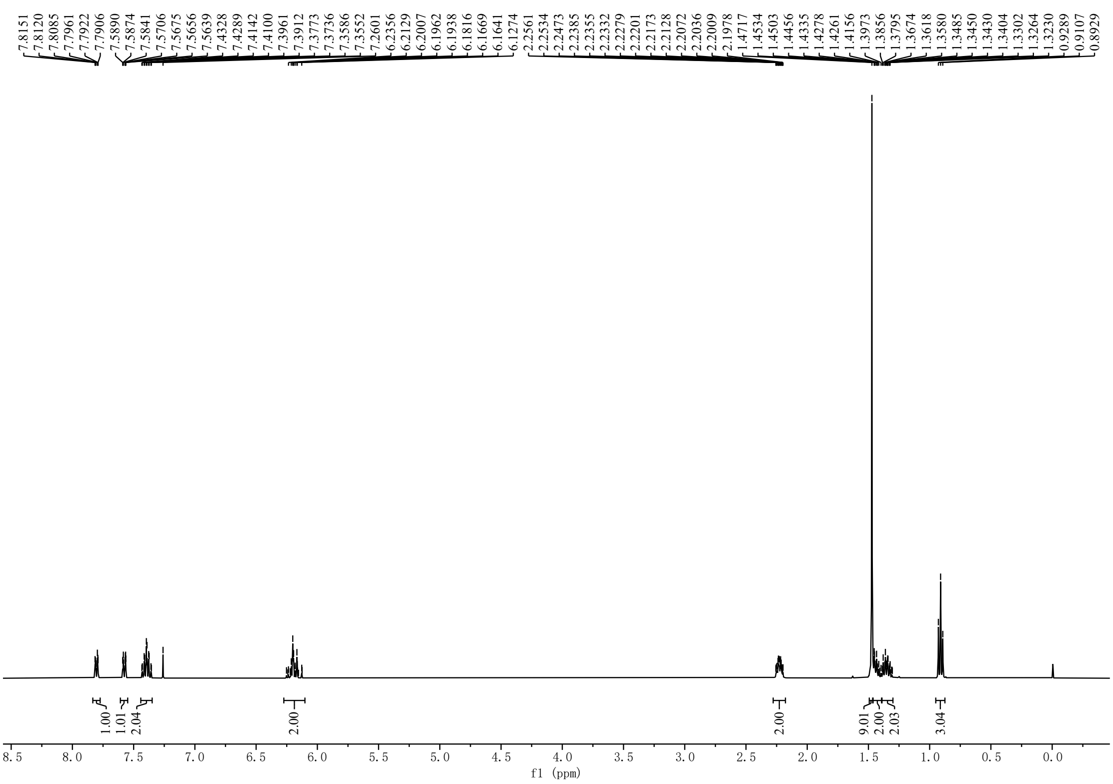

^{13}C NMR spectrum of **4a**

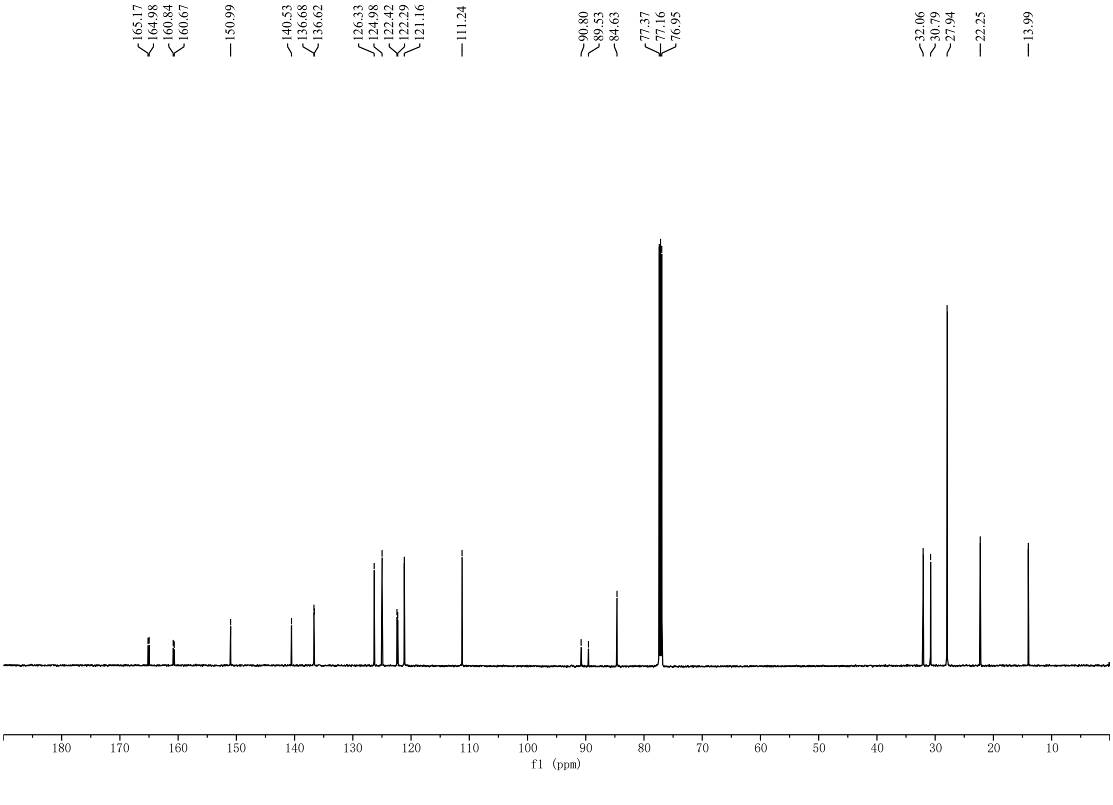

^{19}F NMR spectrum of **4a**

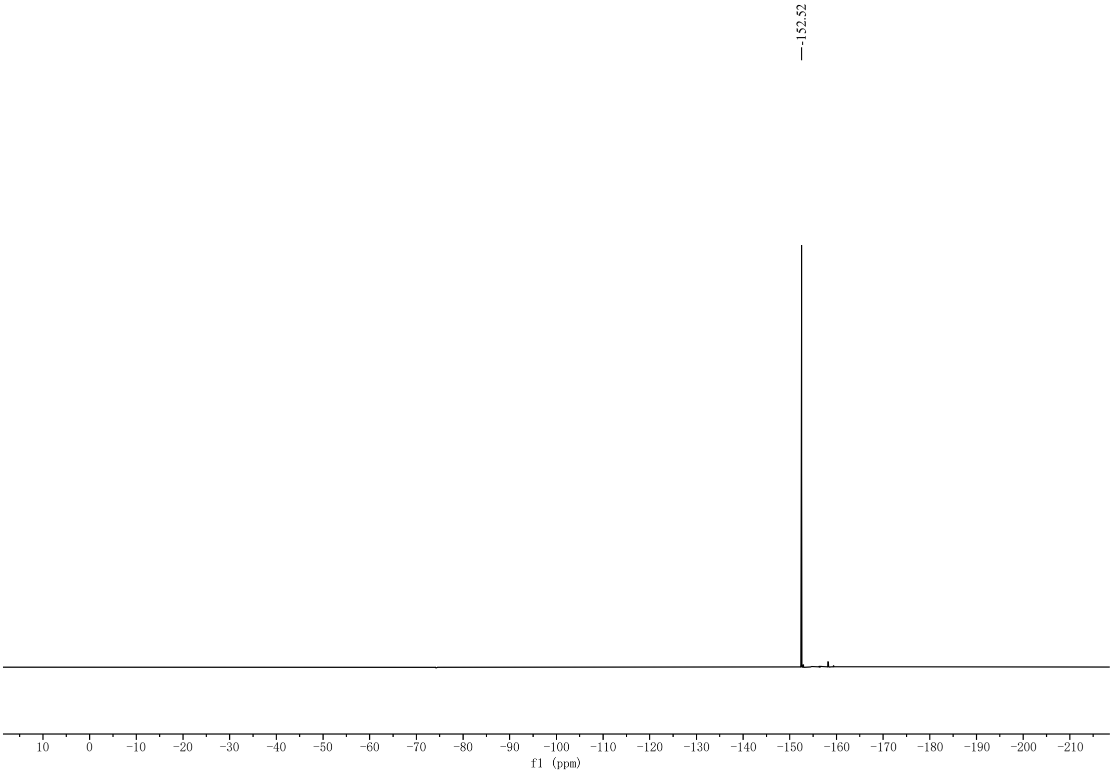

^{1}H NMR spectrum of **4b**

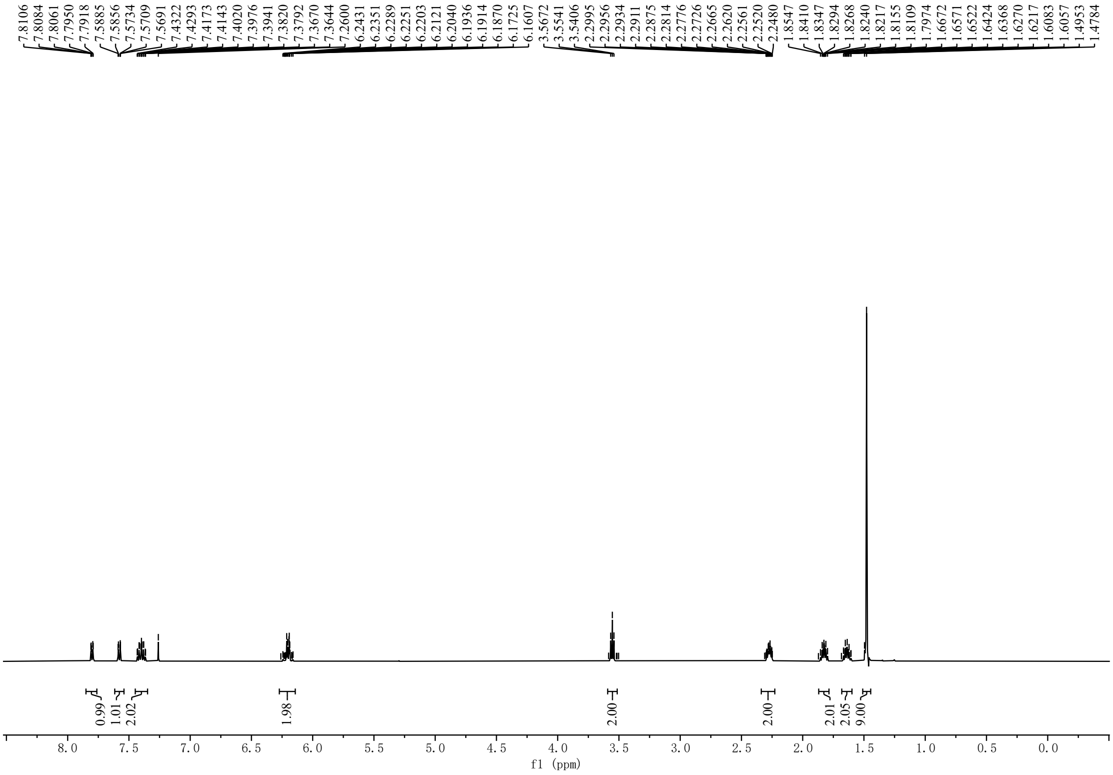

^{13}C NMR spectrum of **4b**

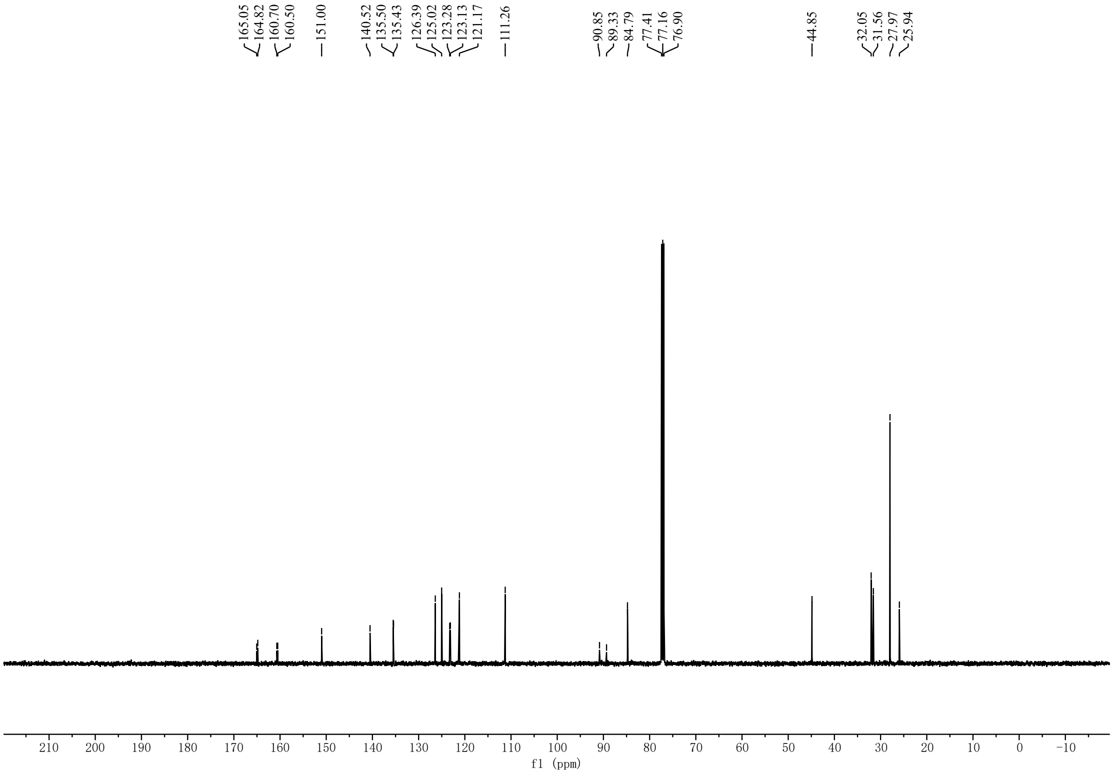

^{19}F NMR spectrum of **4b**

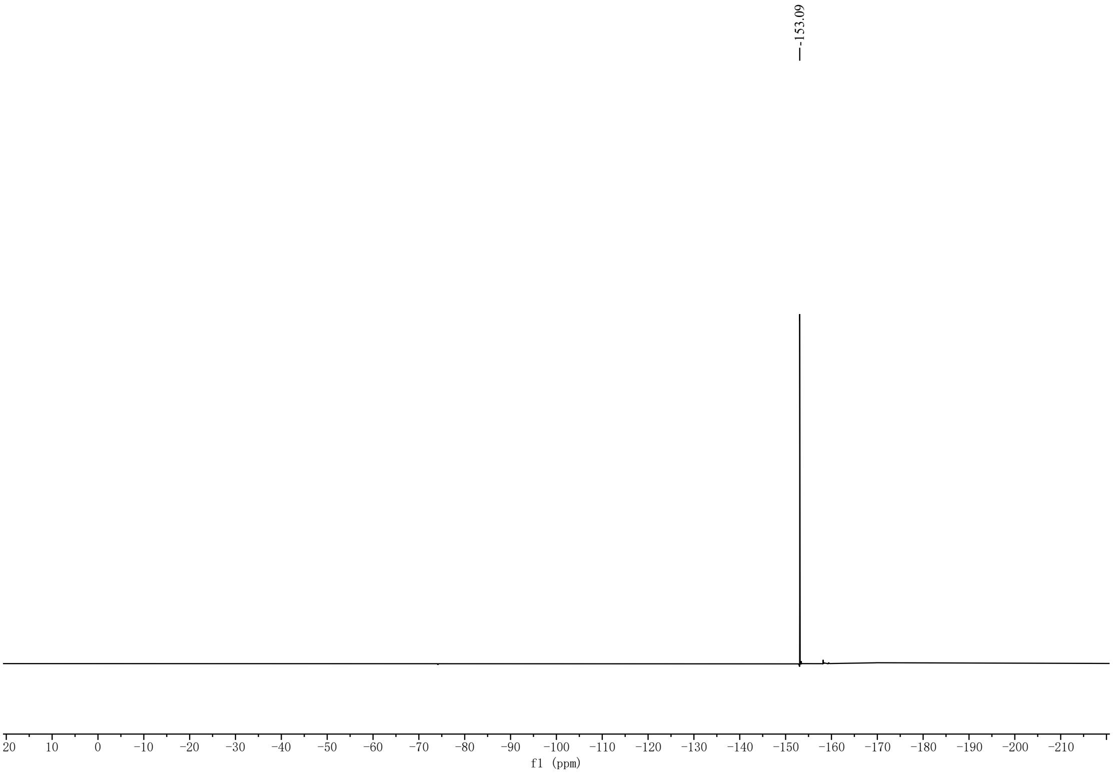

^{1}H NMR spectrum of **4c**

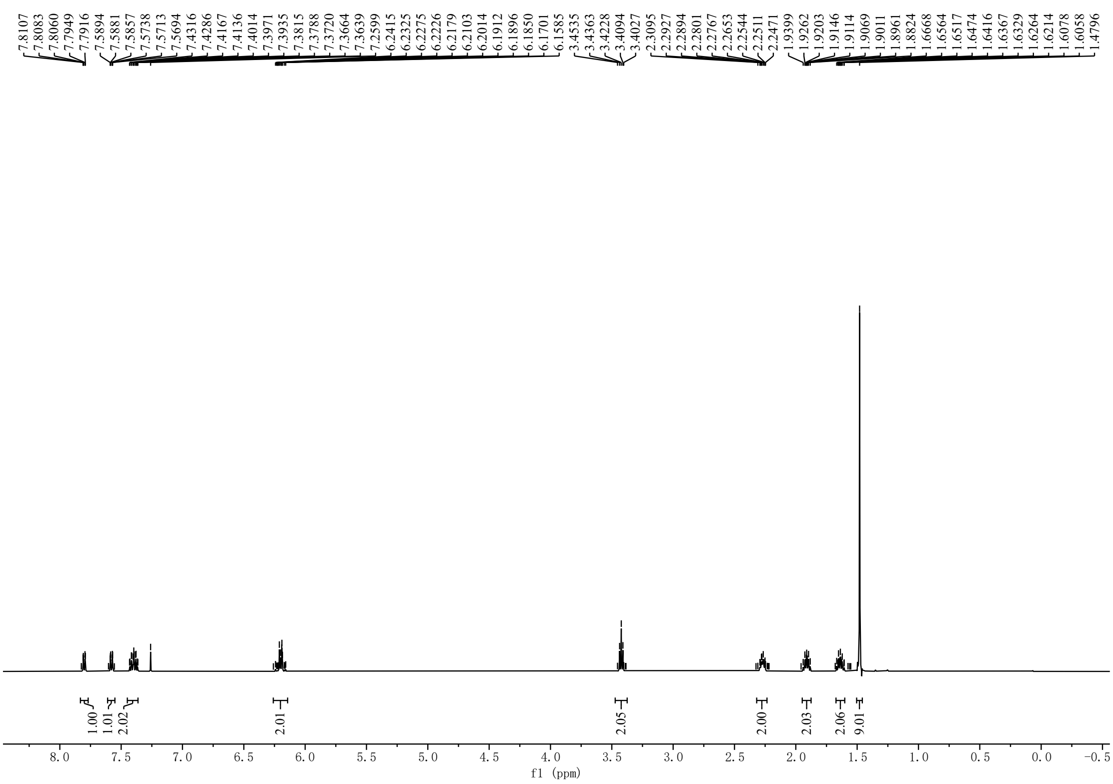

^{13}C NMR spectrum of **4c**

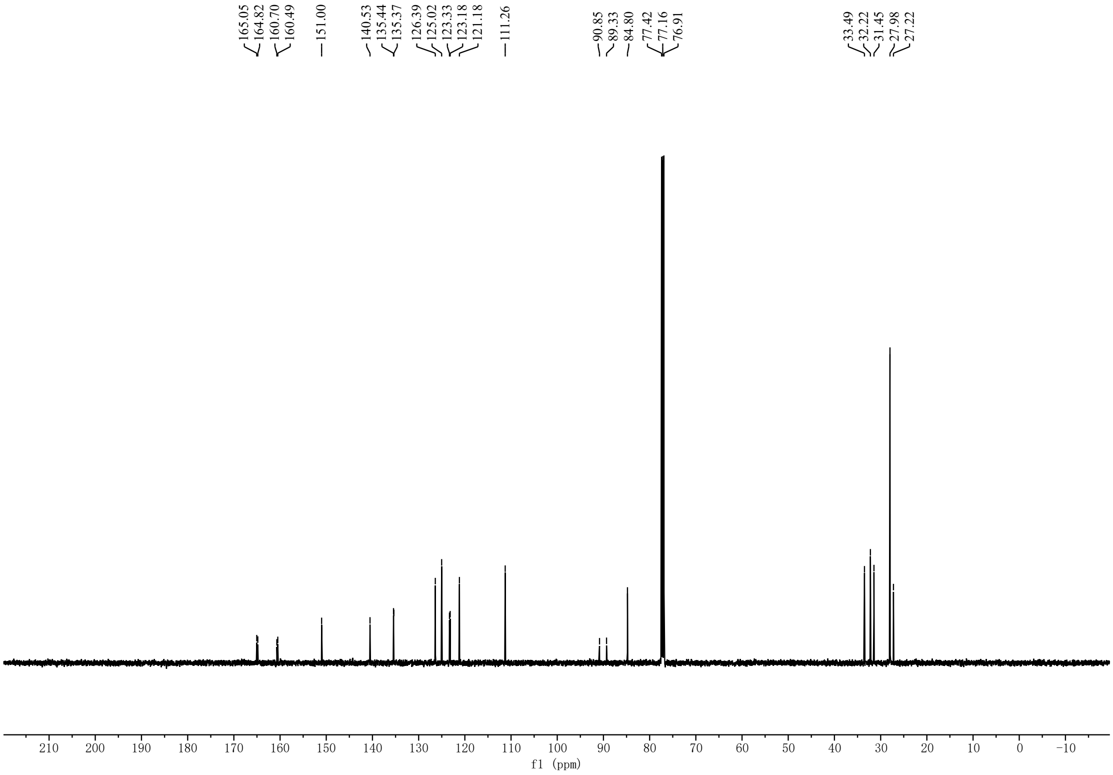

^{19}F NMR spectrum of **4c**

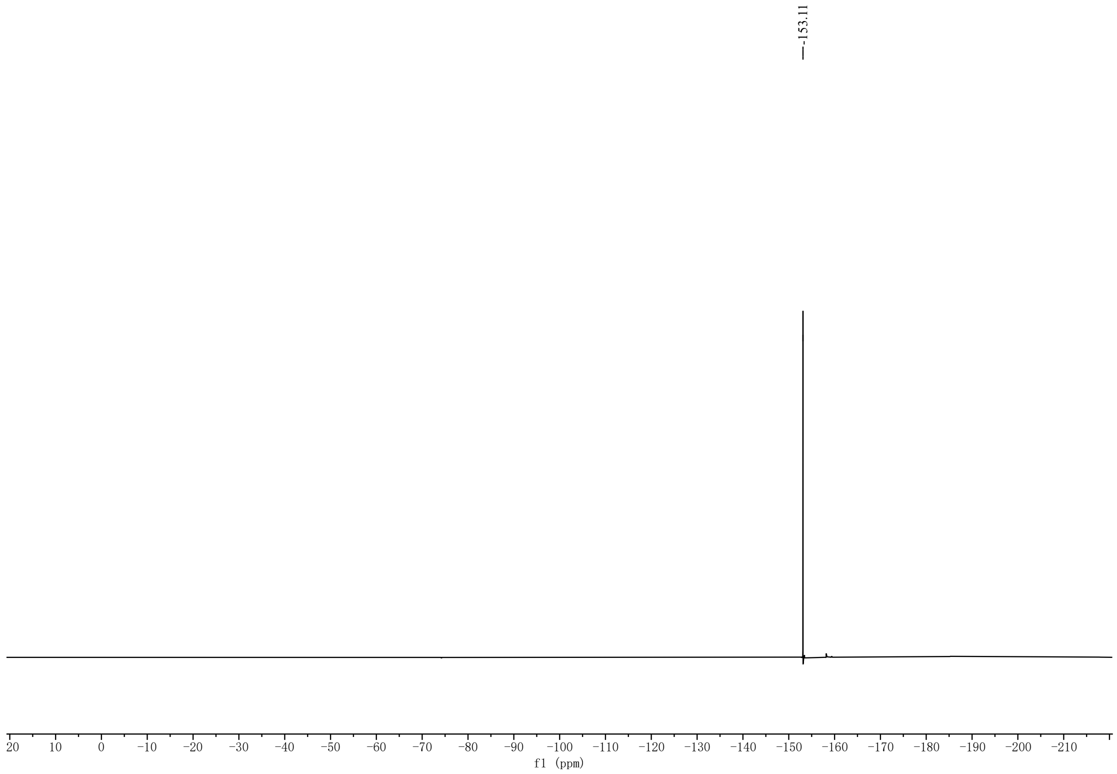

^{1}H NMR spectrum of **4d**

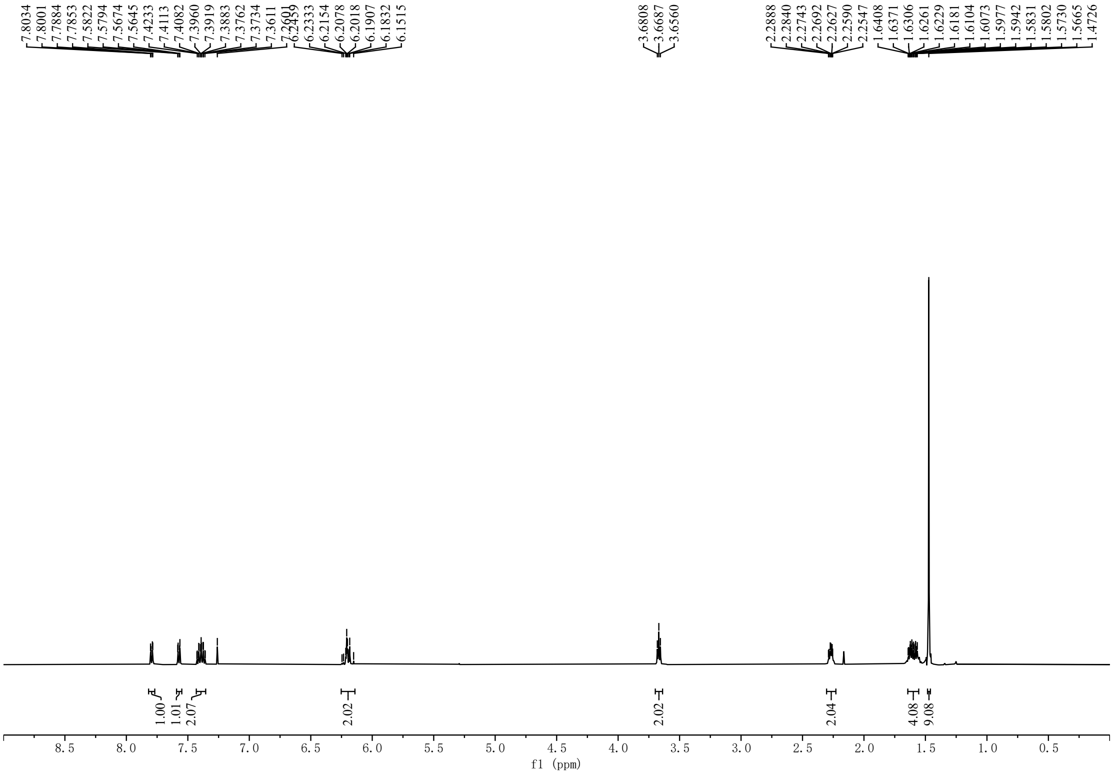

^{13}C NMR spectrum of **4d**

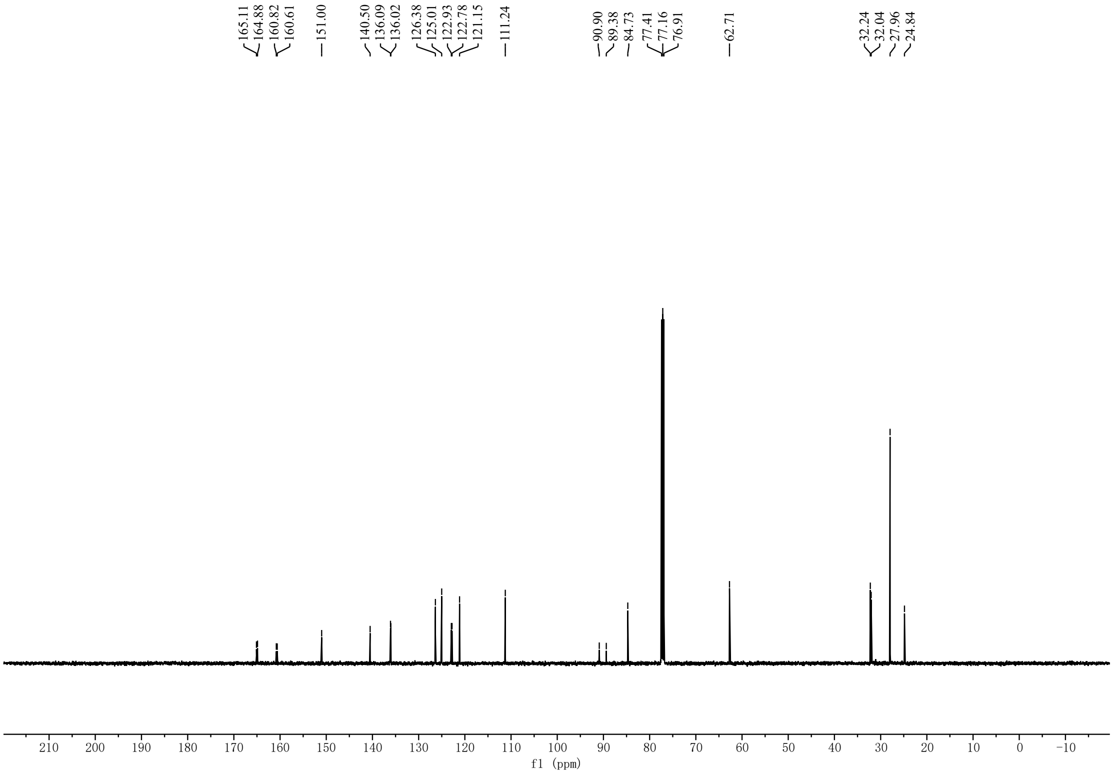

^{19}F NMR spectrum of **4d**

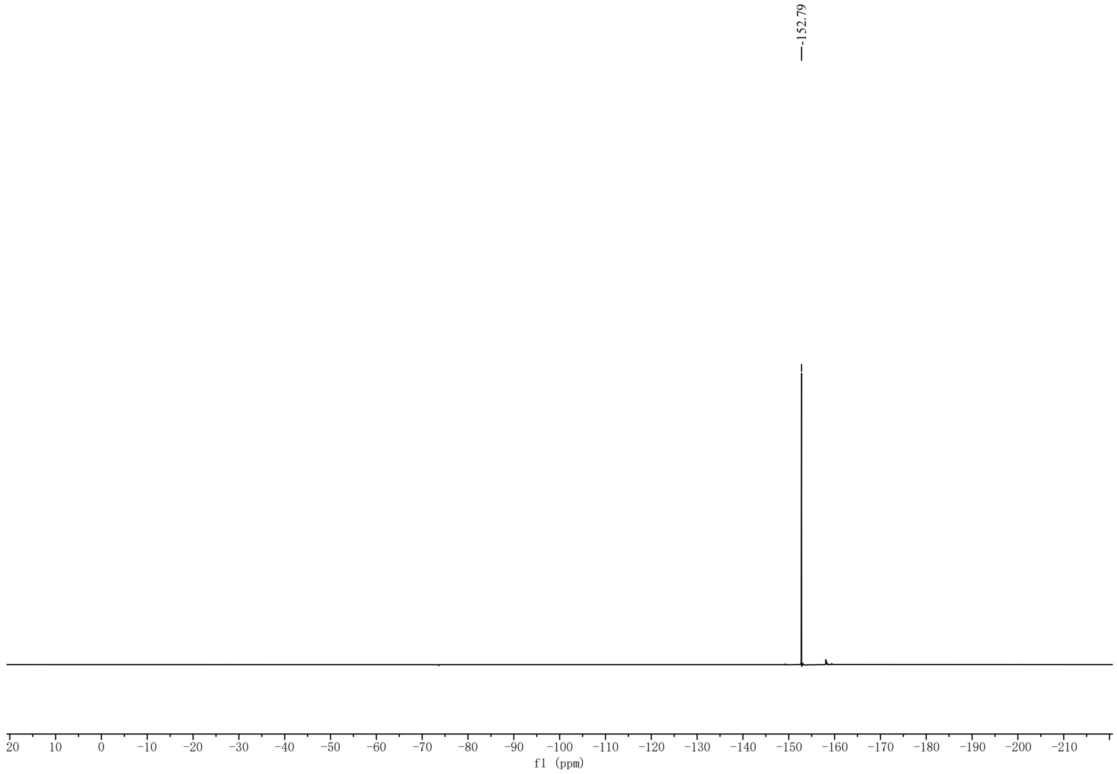

^{1}H NMR spectrum of **4e**

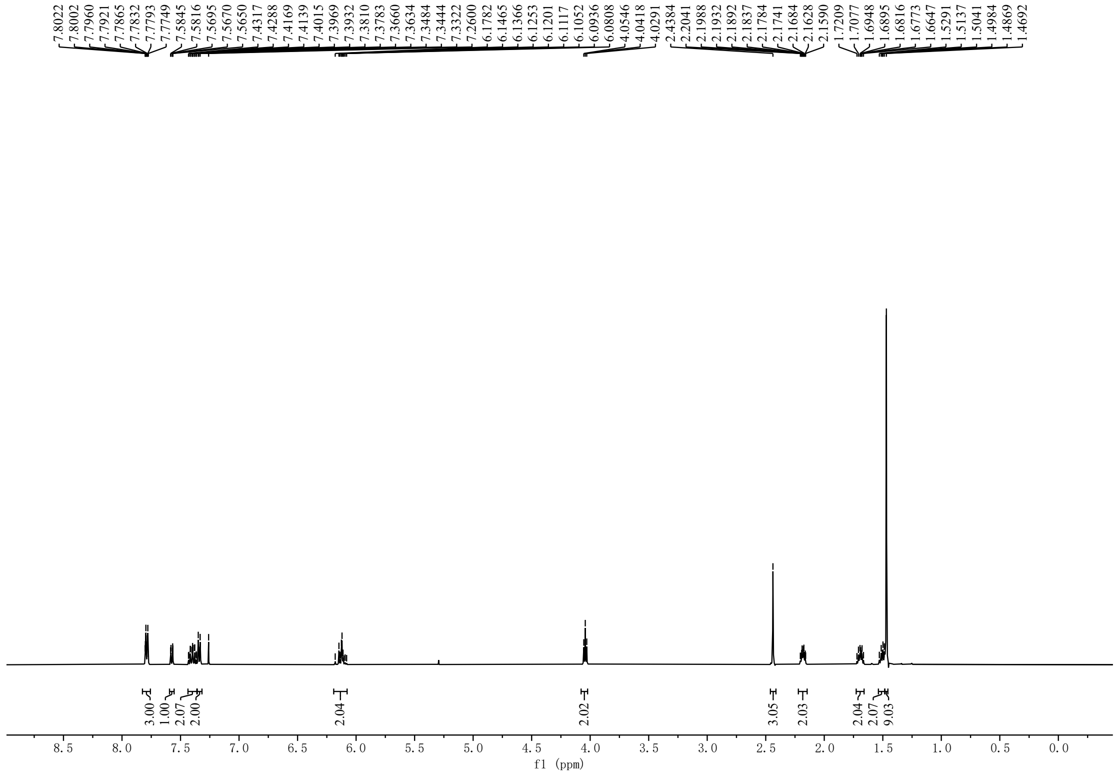

^{13}C NMR spectrum of **4e**

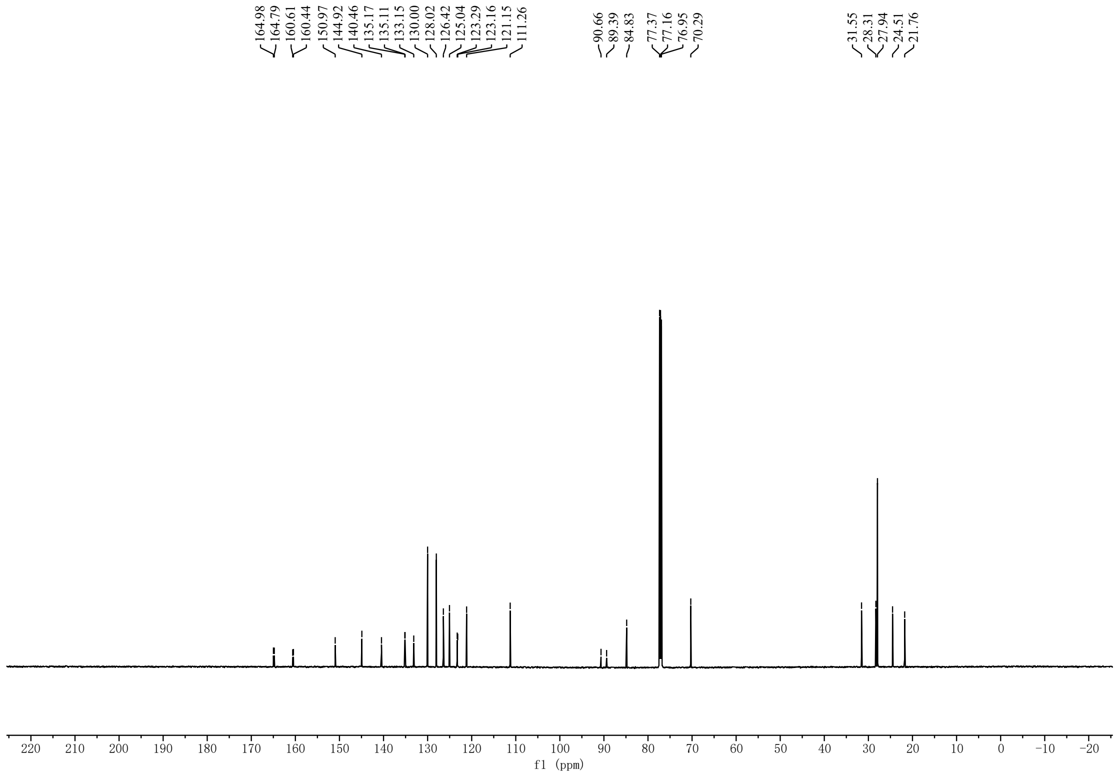

^{19}F NMR spectrum of **4e**

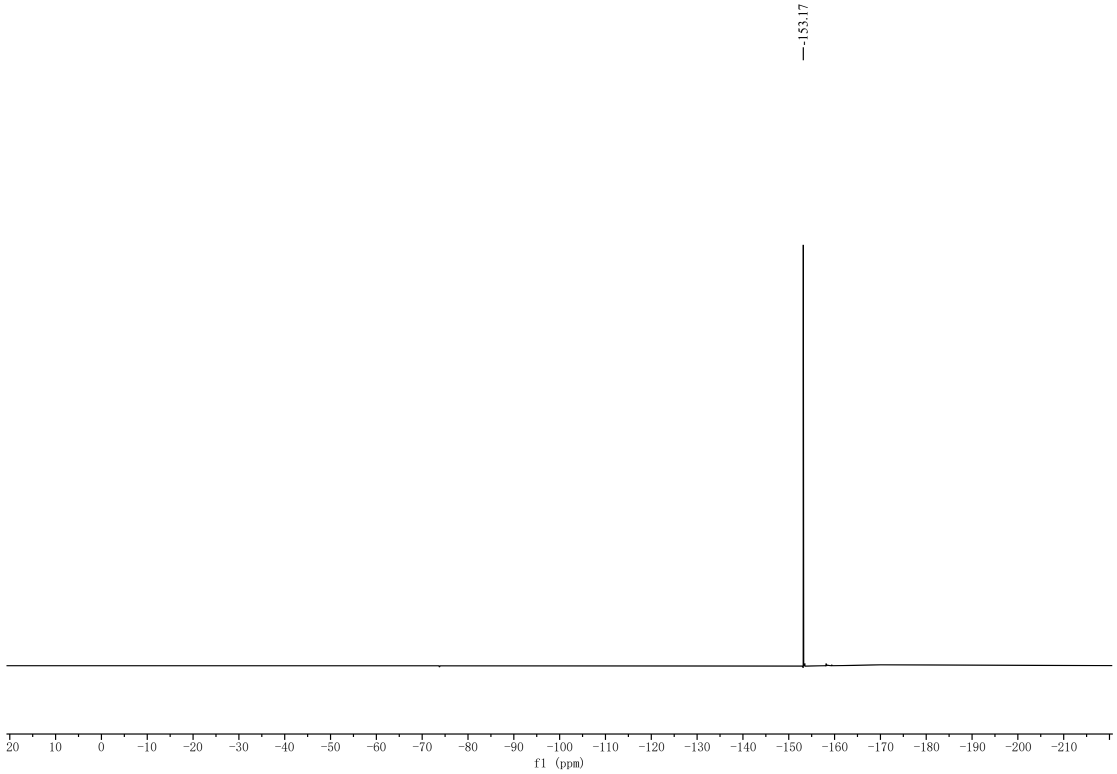

^{1}H NMR spectrum of **4f**

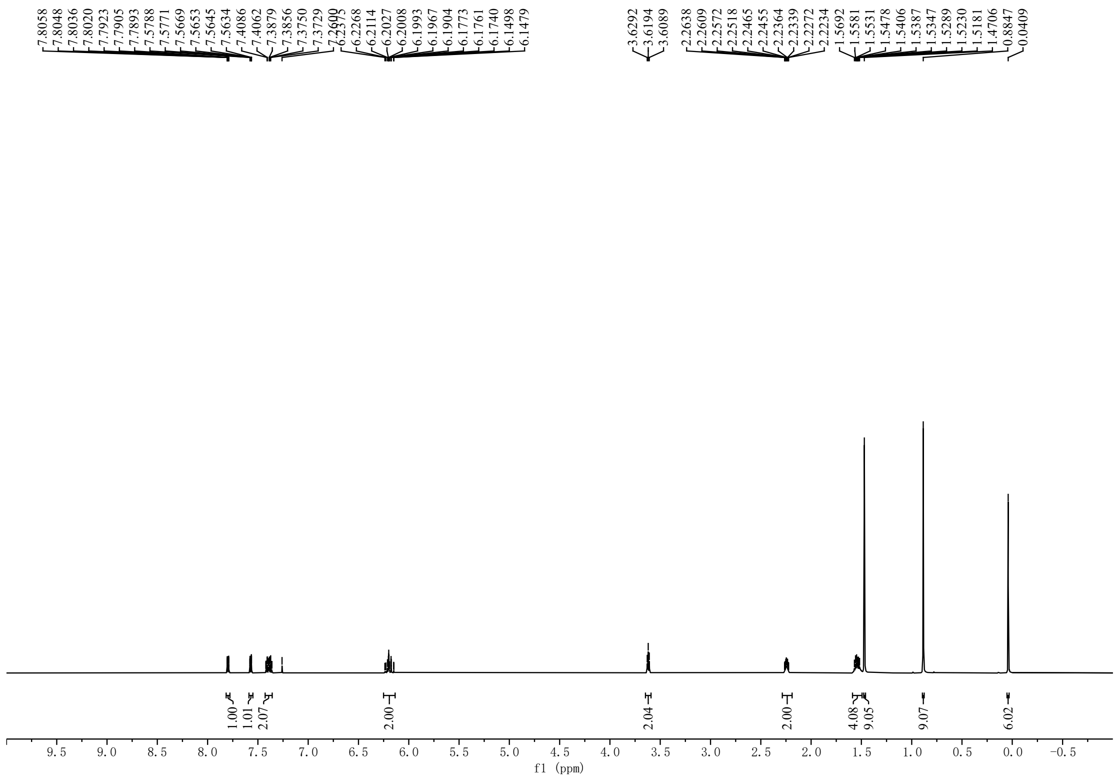

^{13}C NMR spectrum of **4f**

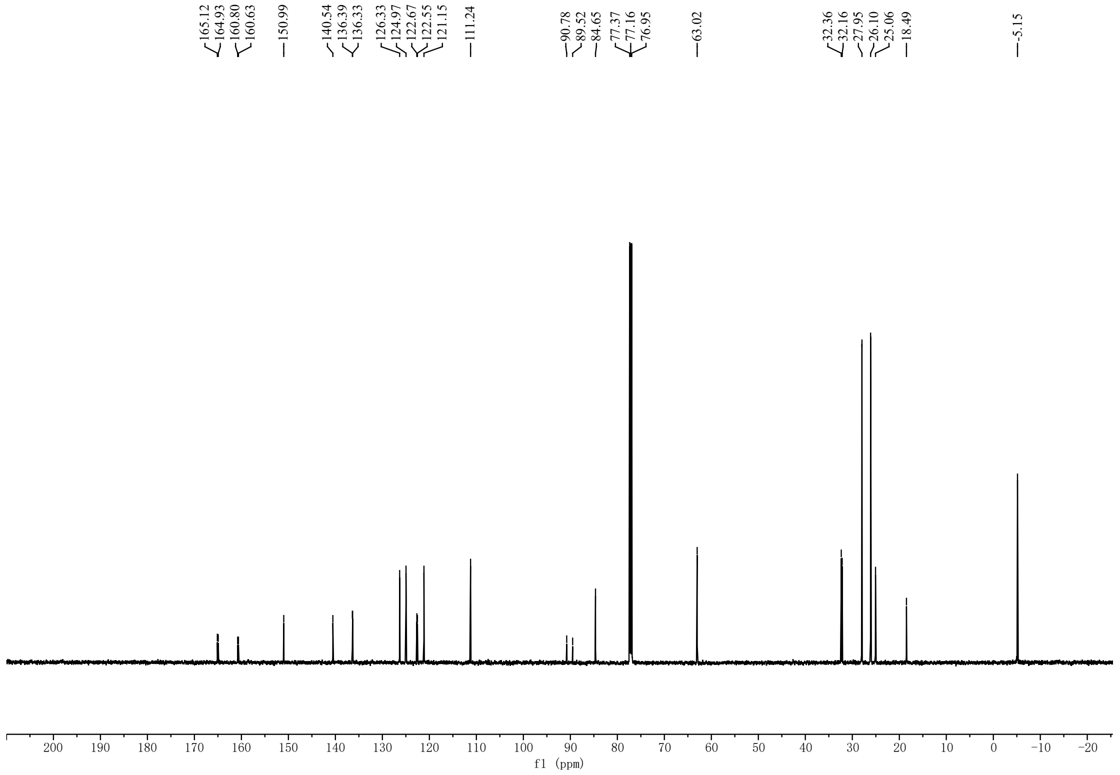

^{19}**F NMR** spectrum of **4f**

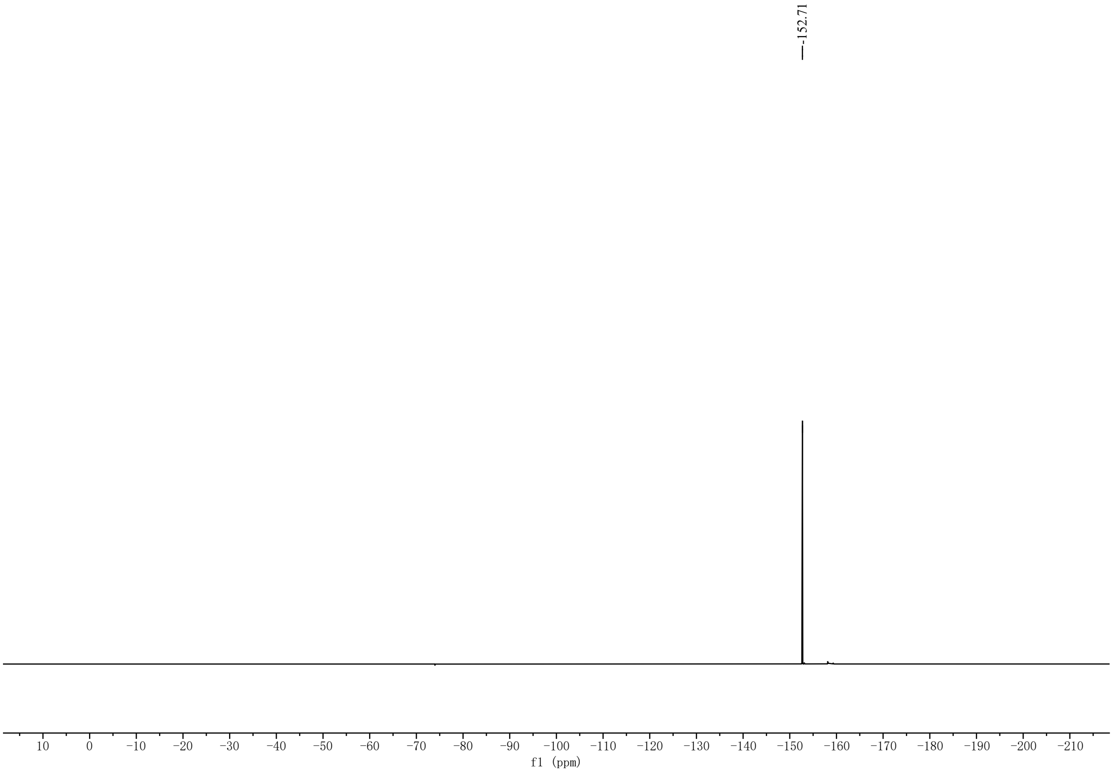

^{1}H NMR spectrum of **4g**

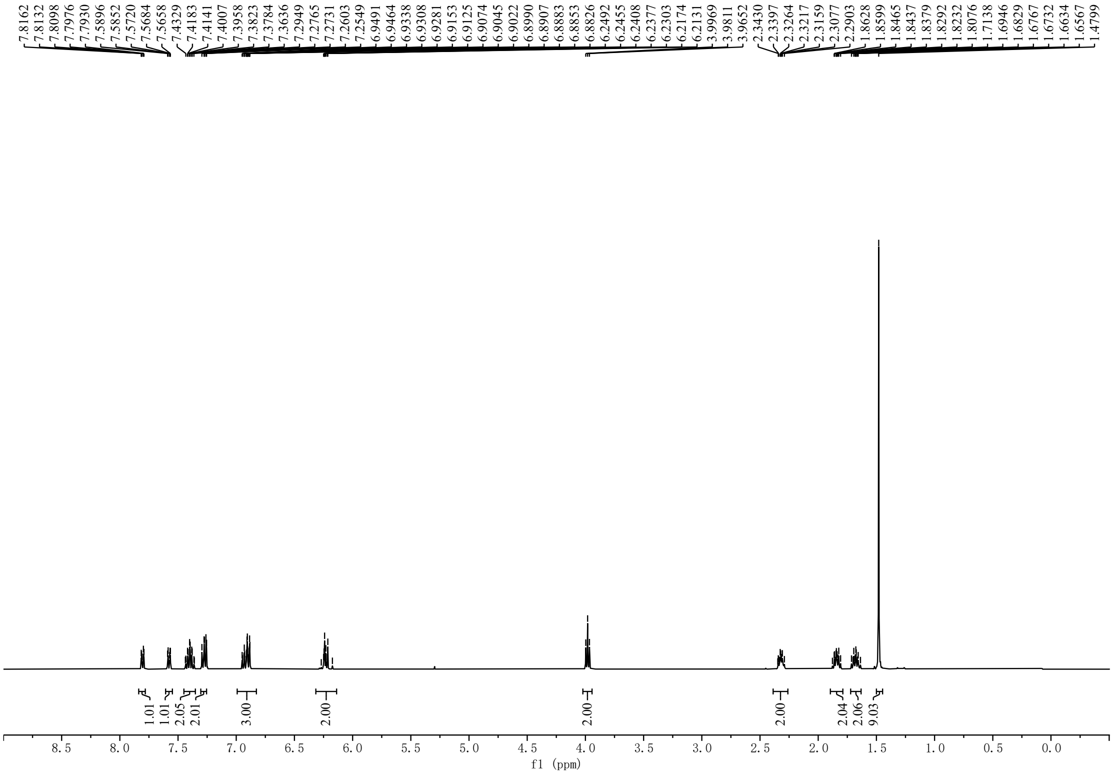

^{13}C NMR spectrum of **4g**

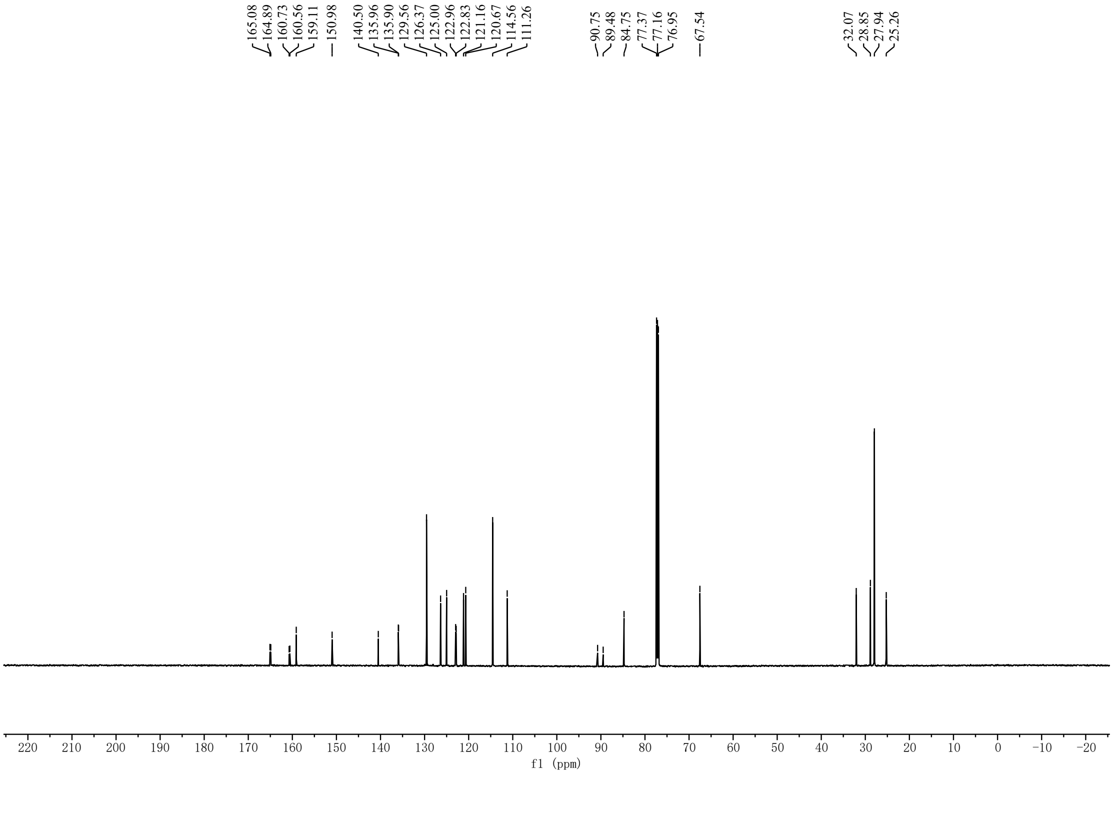

^{19}F NMR spectrum of **4g**

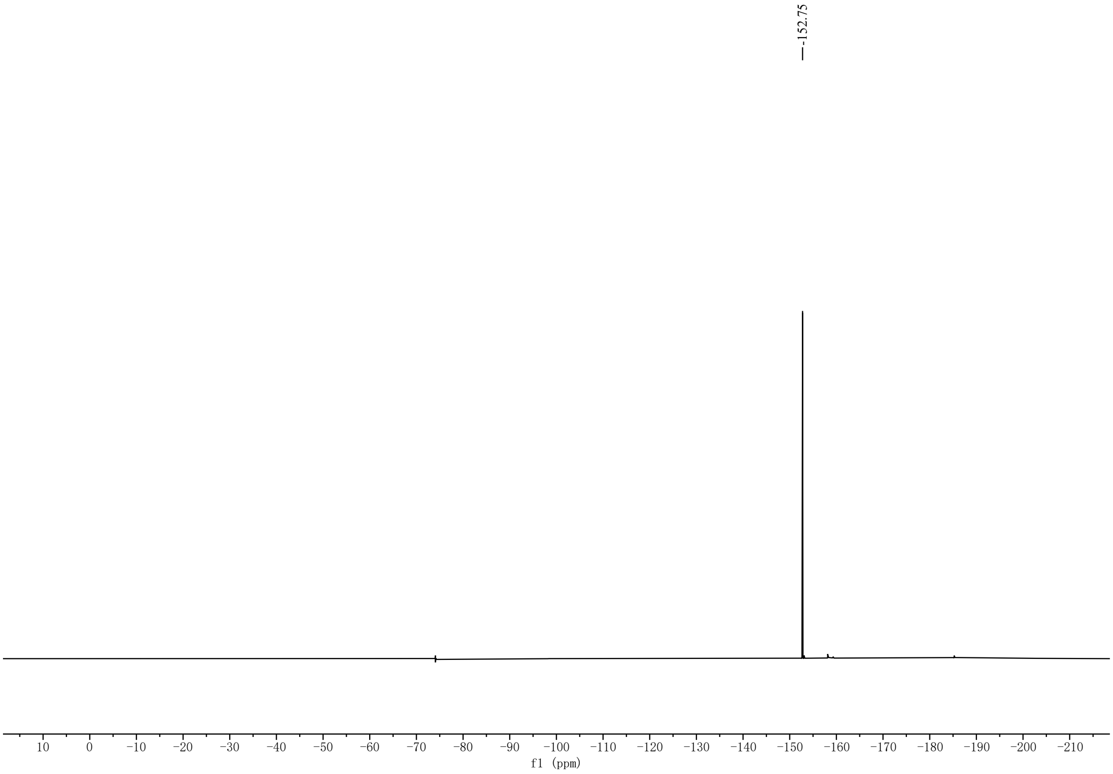

^{1}H NMR spectrum of **4h**

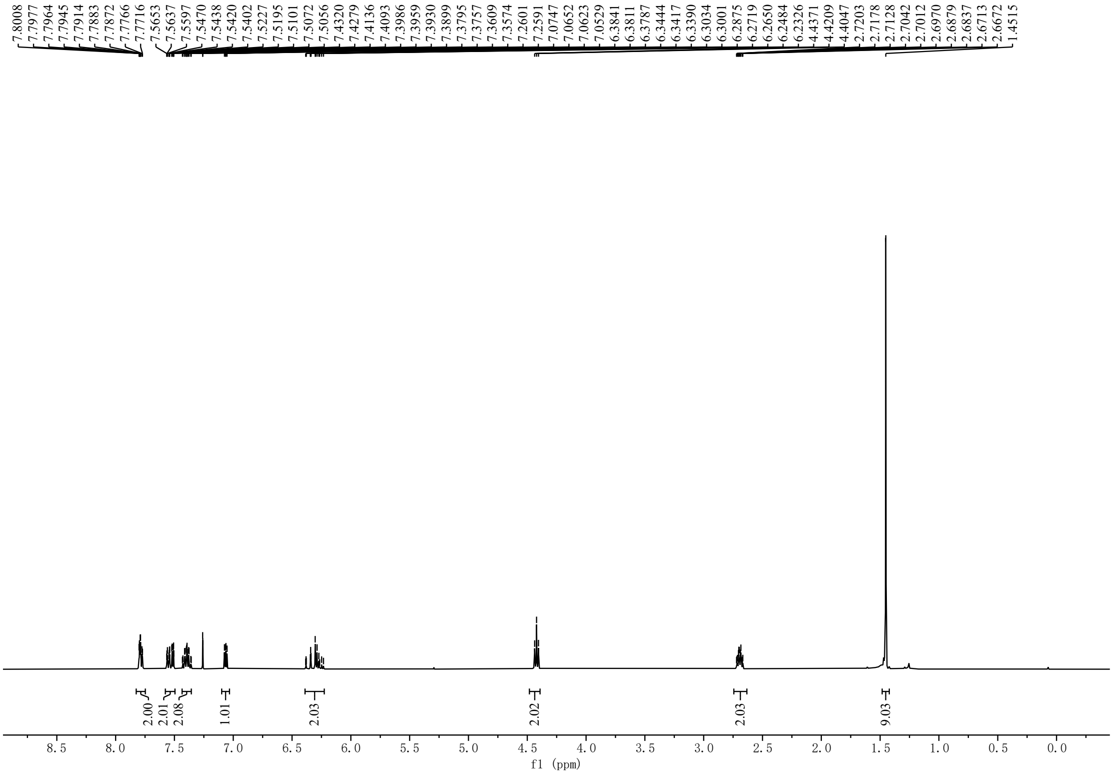

^{13}C NMR spectrum of **4h**

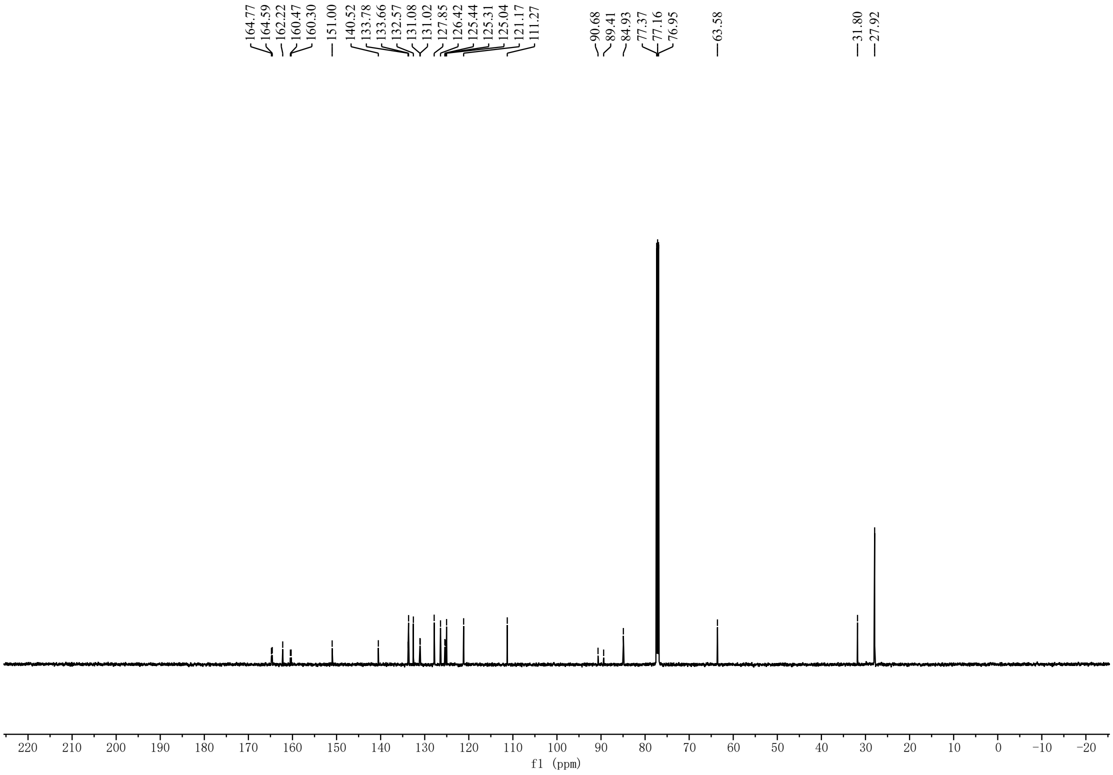

^{19}**F NMR** spectrum of **4h**

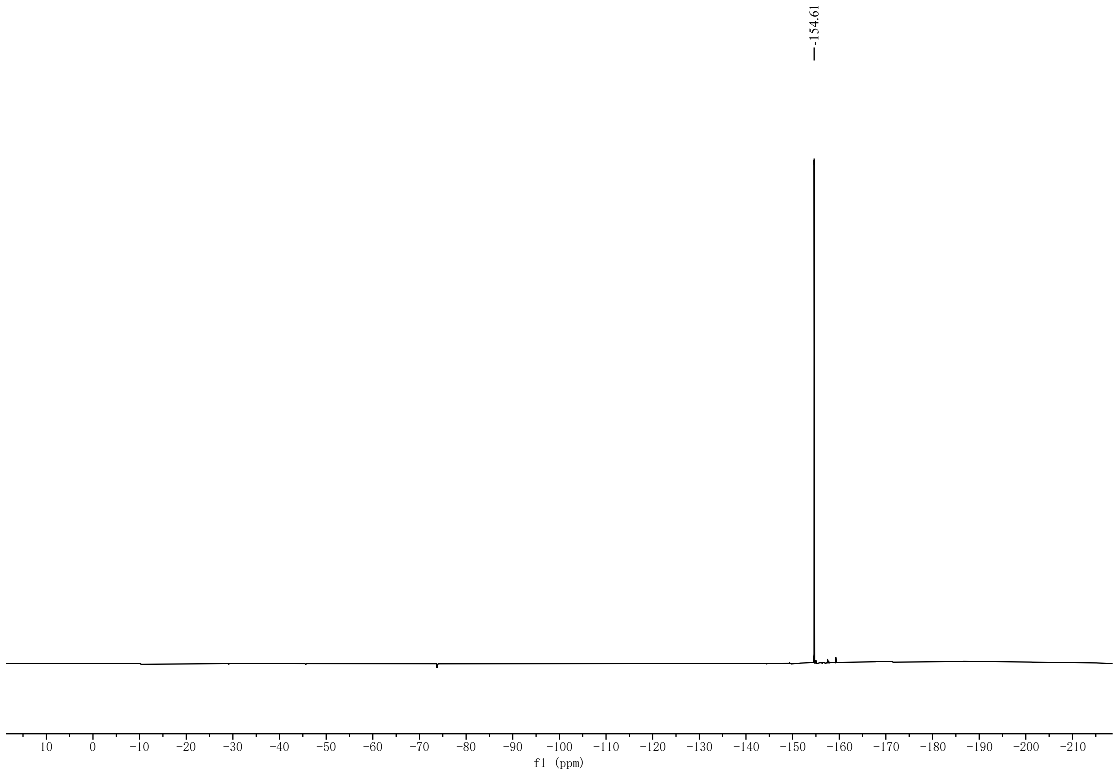

^{1}H NMR spectrum of **4i**

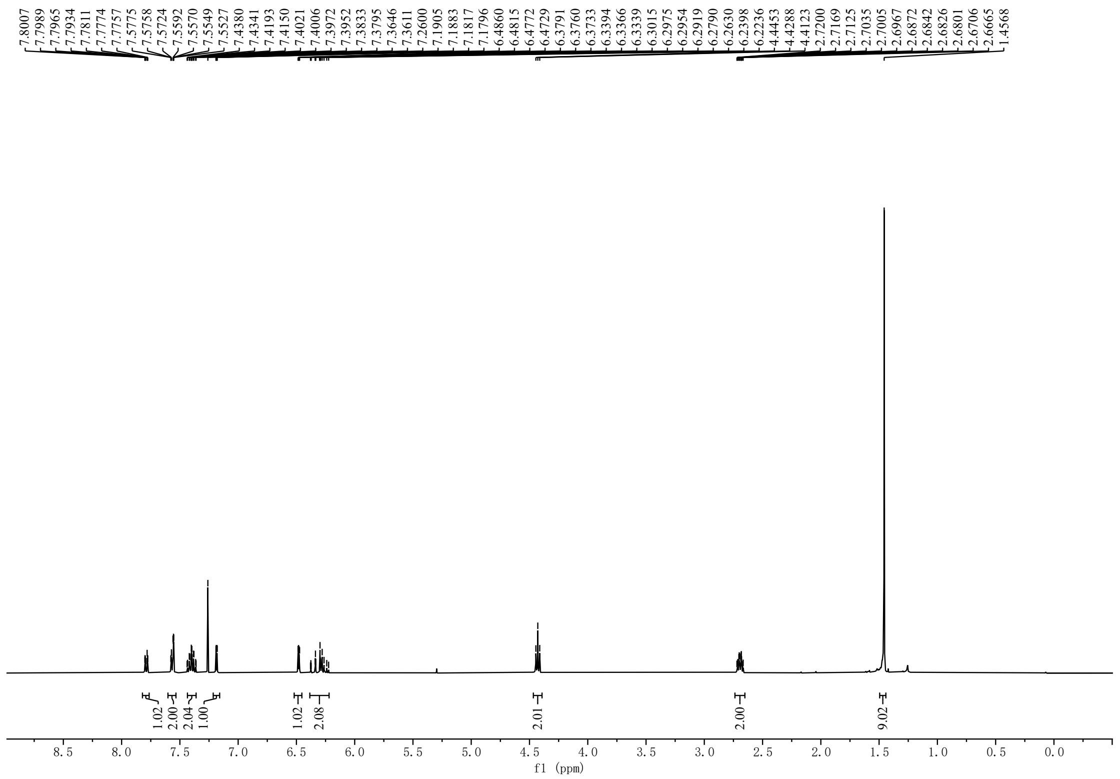

^{13}C NMR spectrum of **4i**

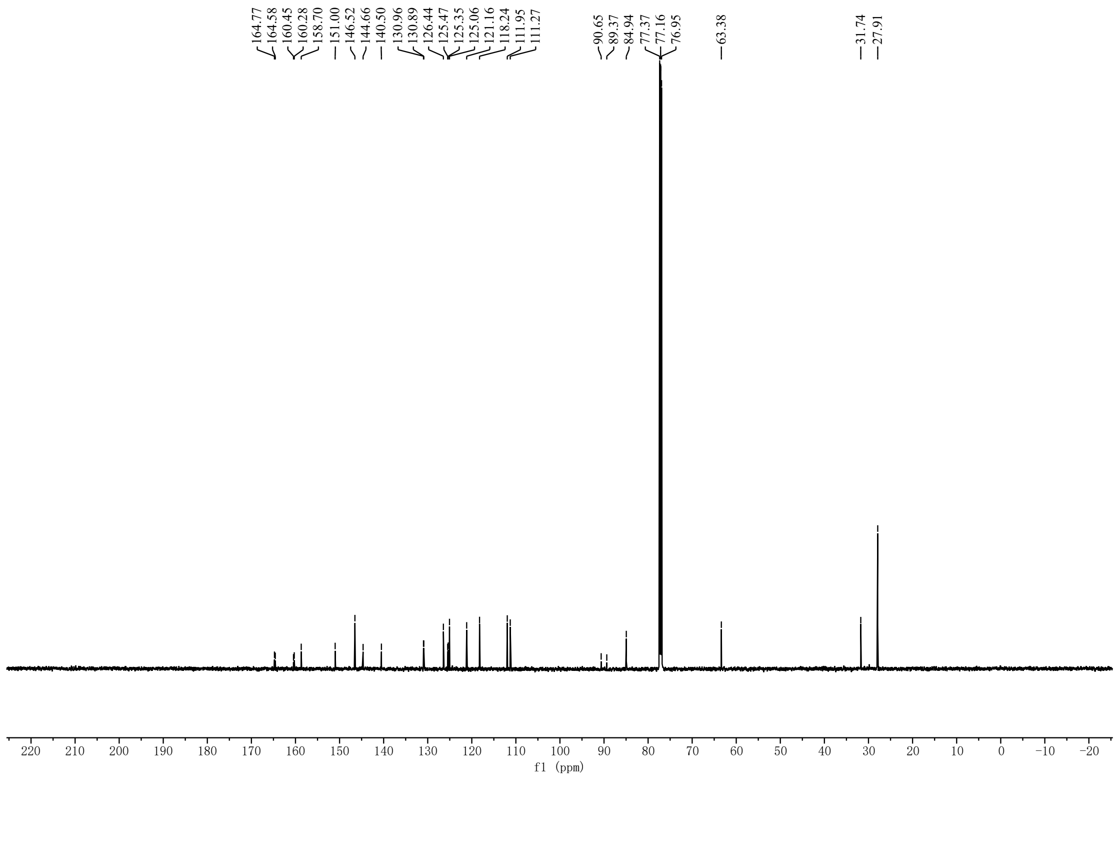

^{19}F NMR spectrum of **4i**

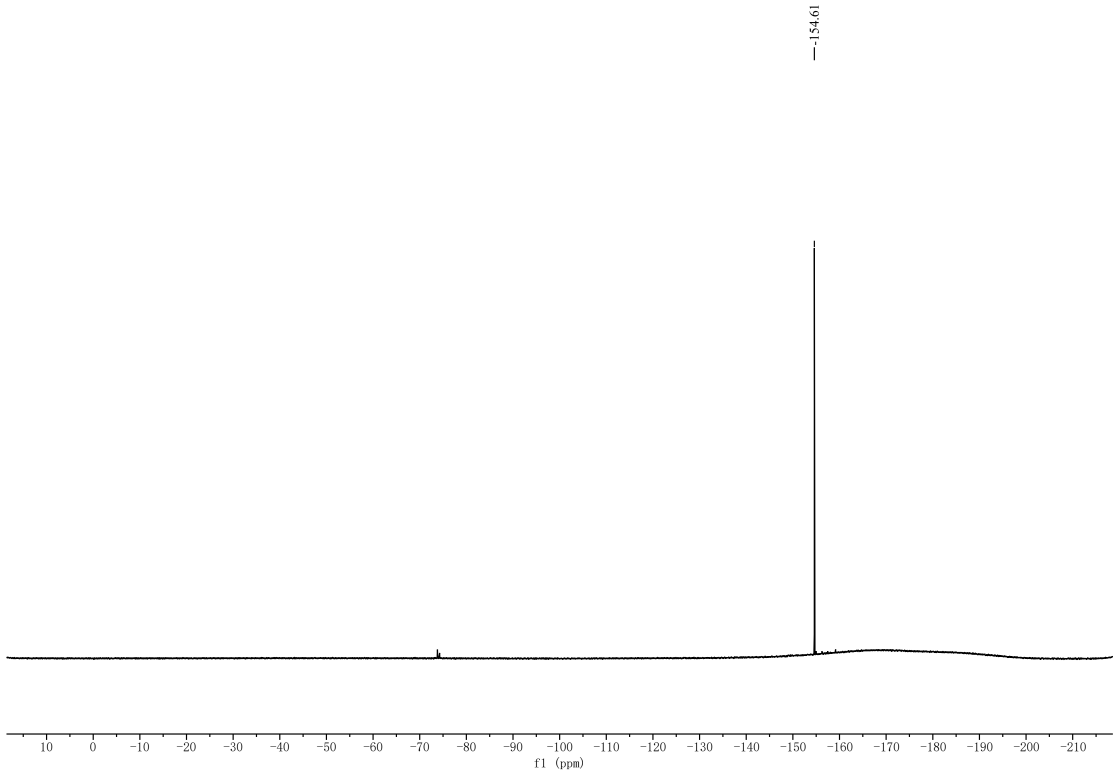

^{1}H NMR spectrum of **4j**

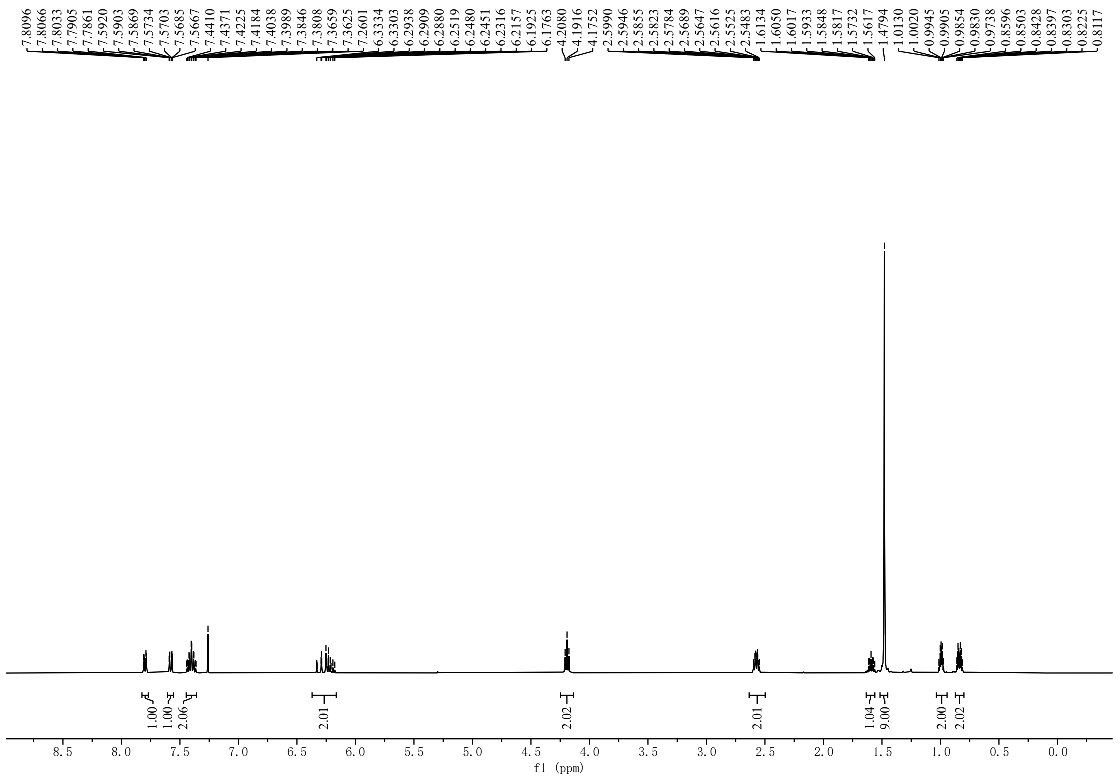

^{13}C NMR spectrum of **4j**

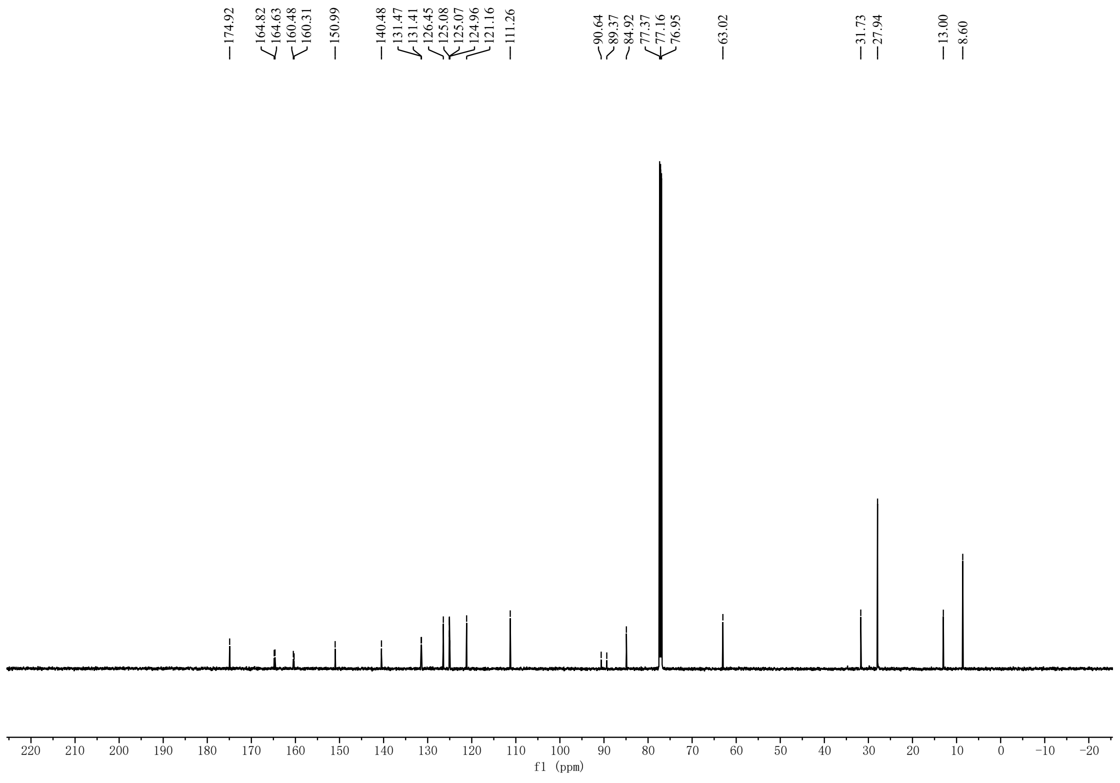

^{19}F **NMR** spectrum of **4j**

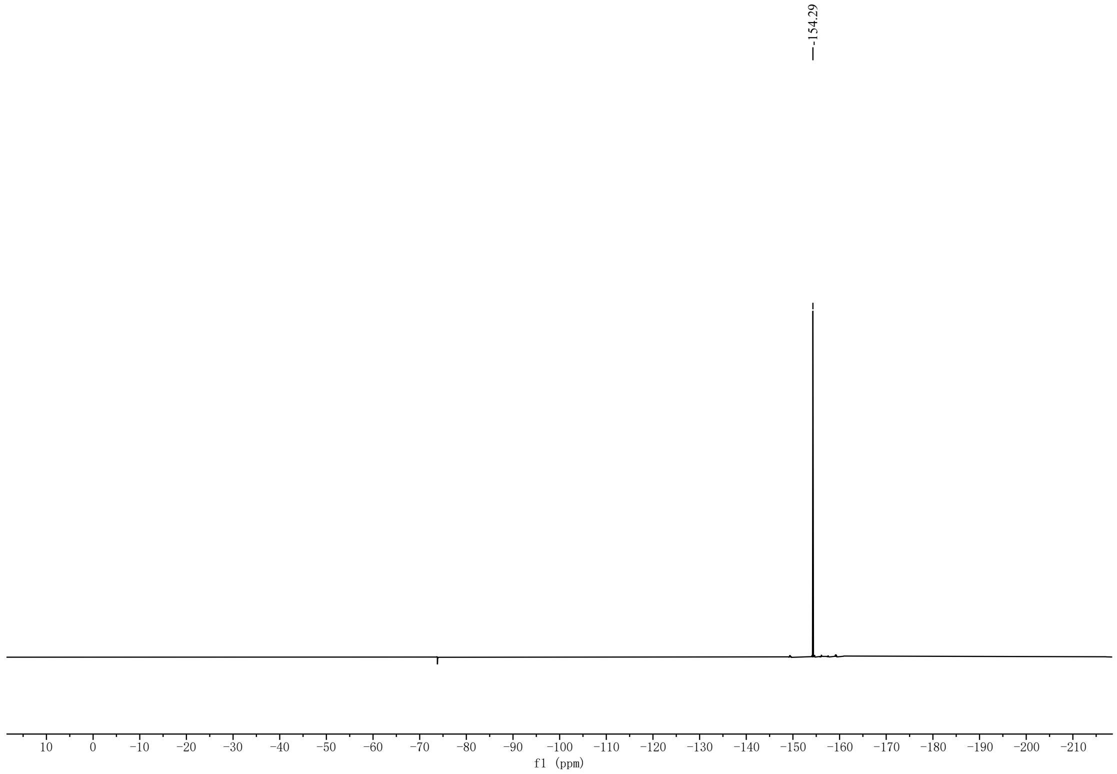

^{1}H NMR spectrum of **4k**

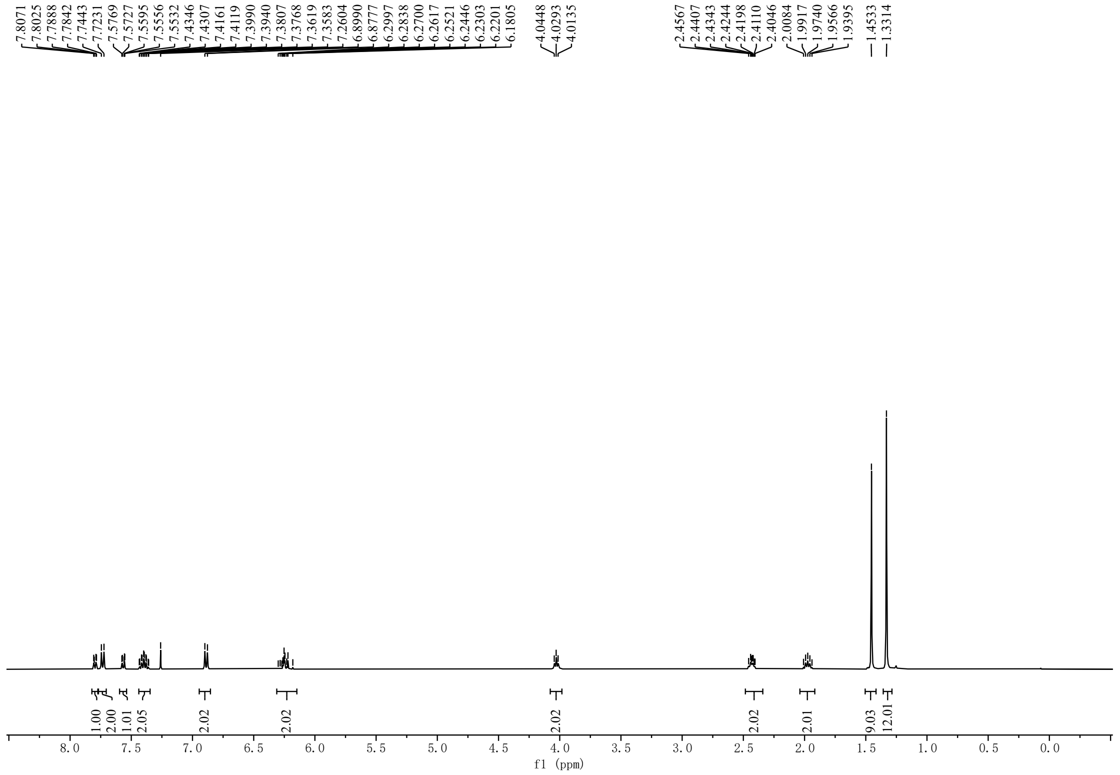

^{13}C NMR spectrum of **4k**

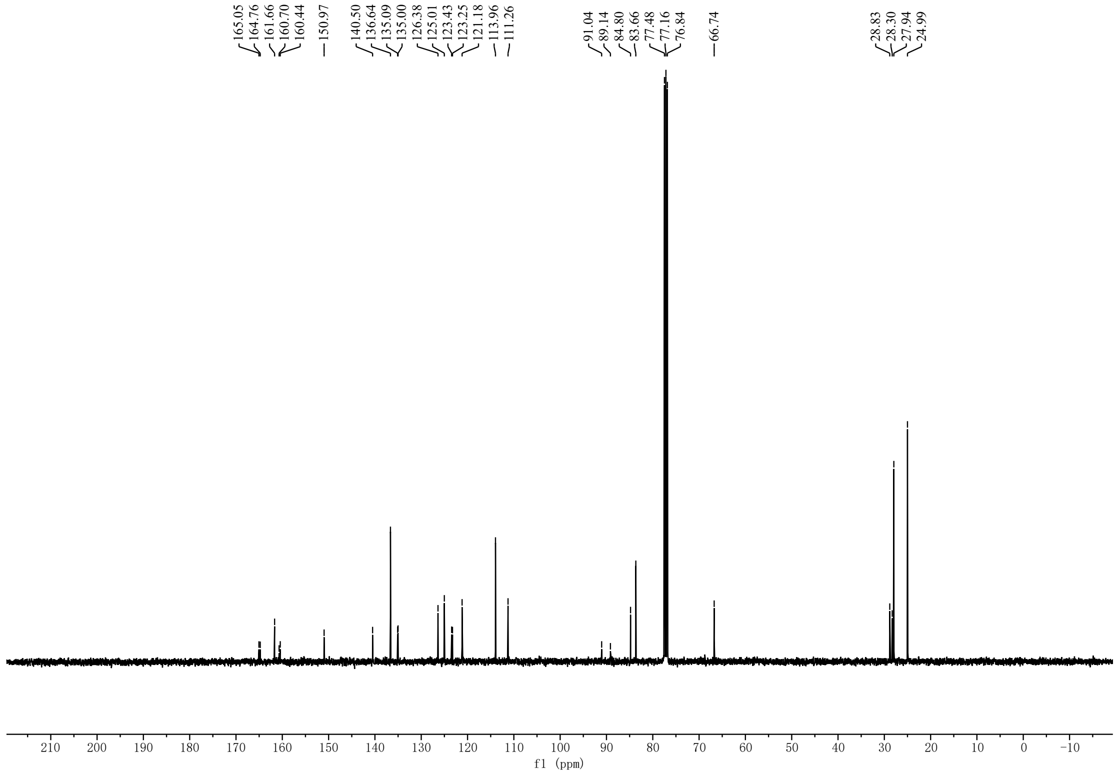

^{19}F NMR spectrum of **4k**

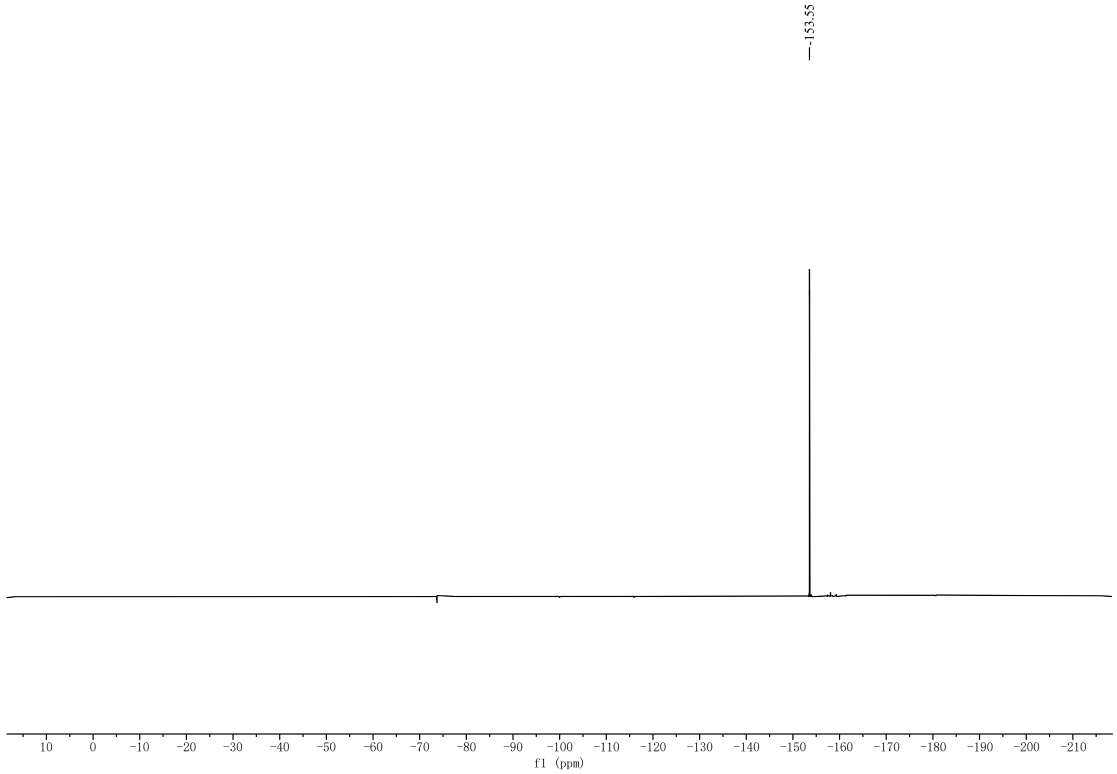

^{1}H NMR spectrum of **4l**

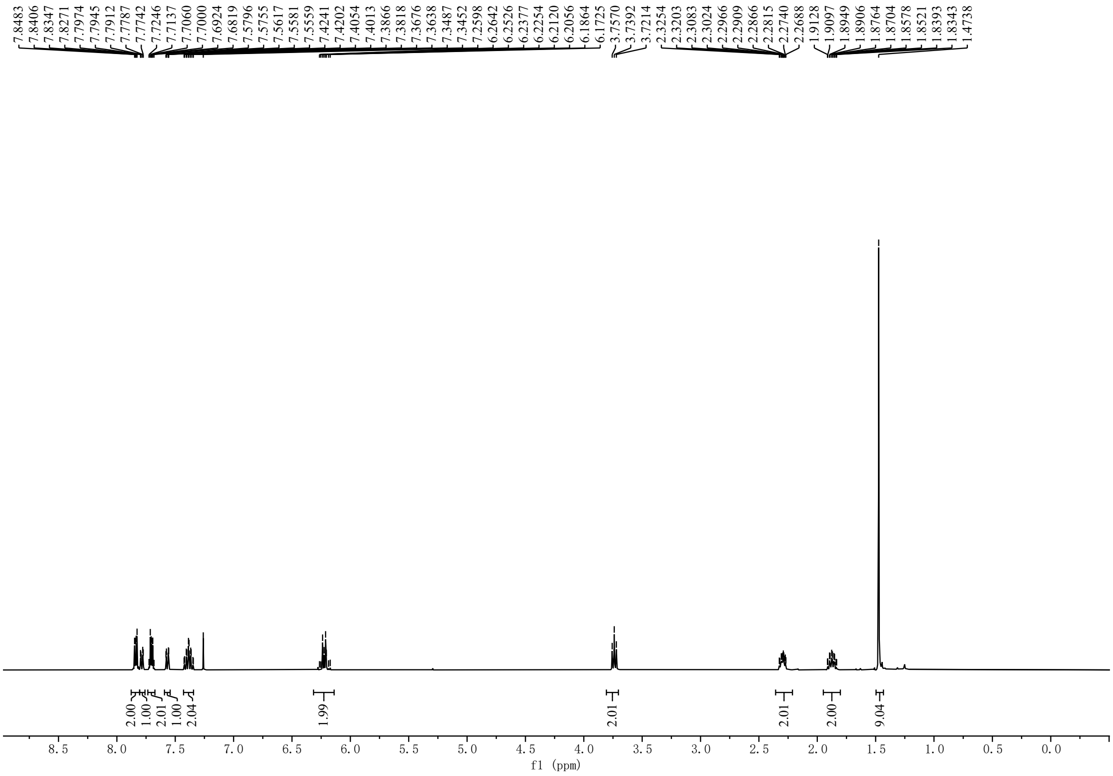

^{13}C NMR spectrum of **4l**

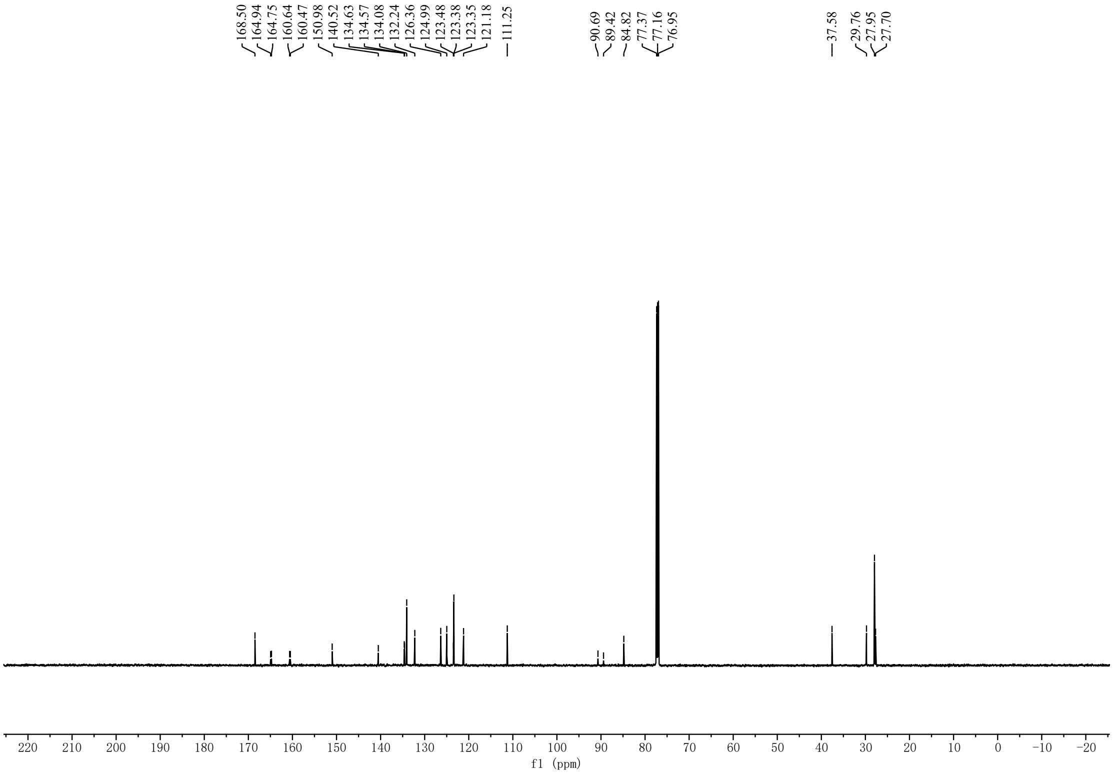

^{19}F NMR spectrum of **4l**

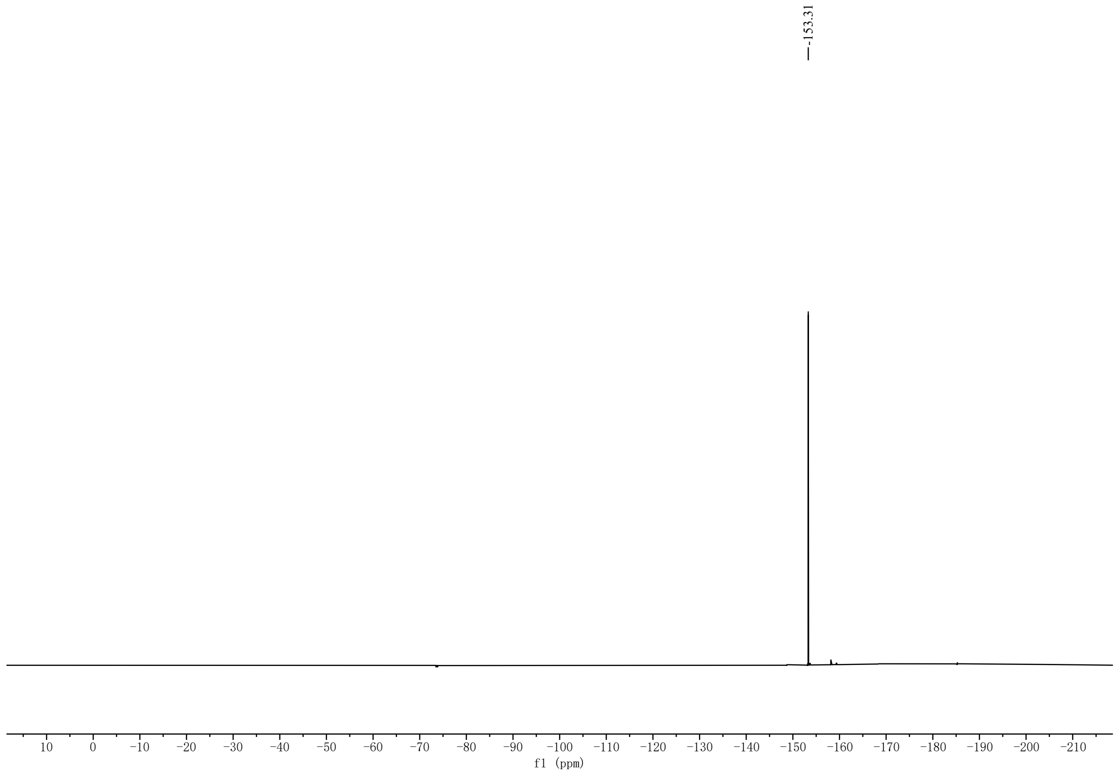

^{1}**H NMR** spectrum of **4m**

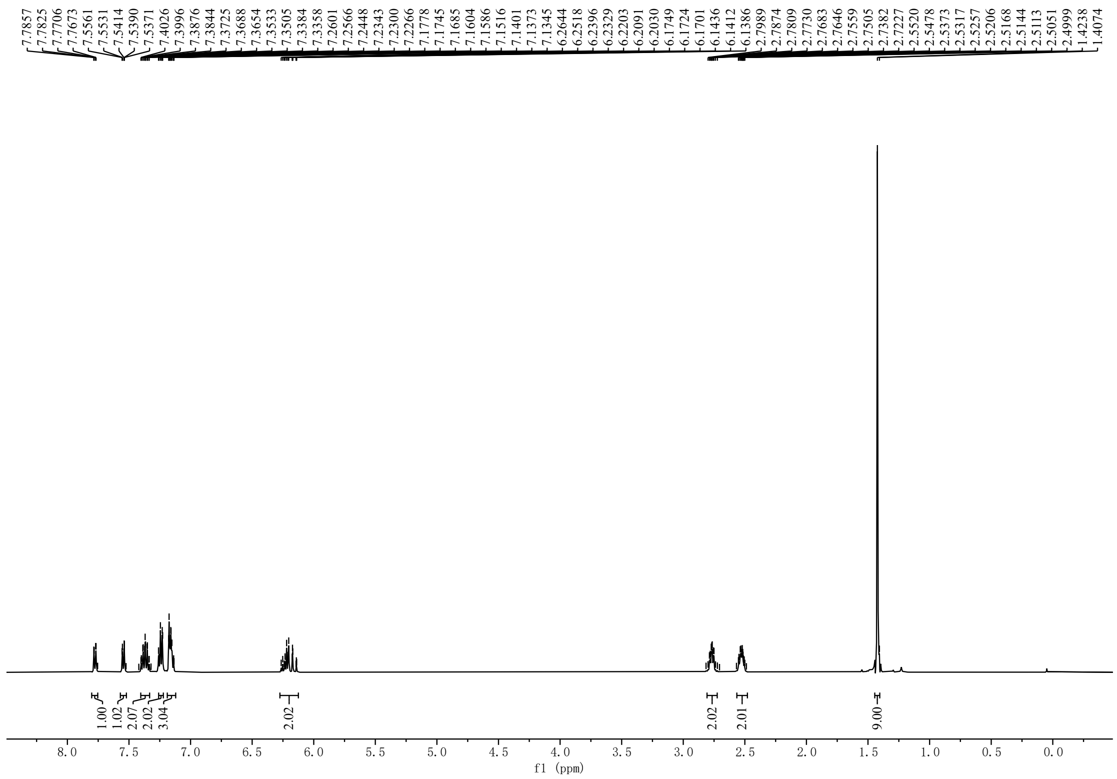

^{13}**C NMR** spectrum of **4m**

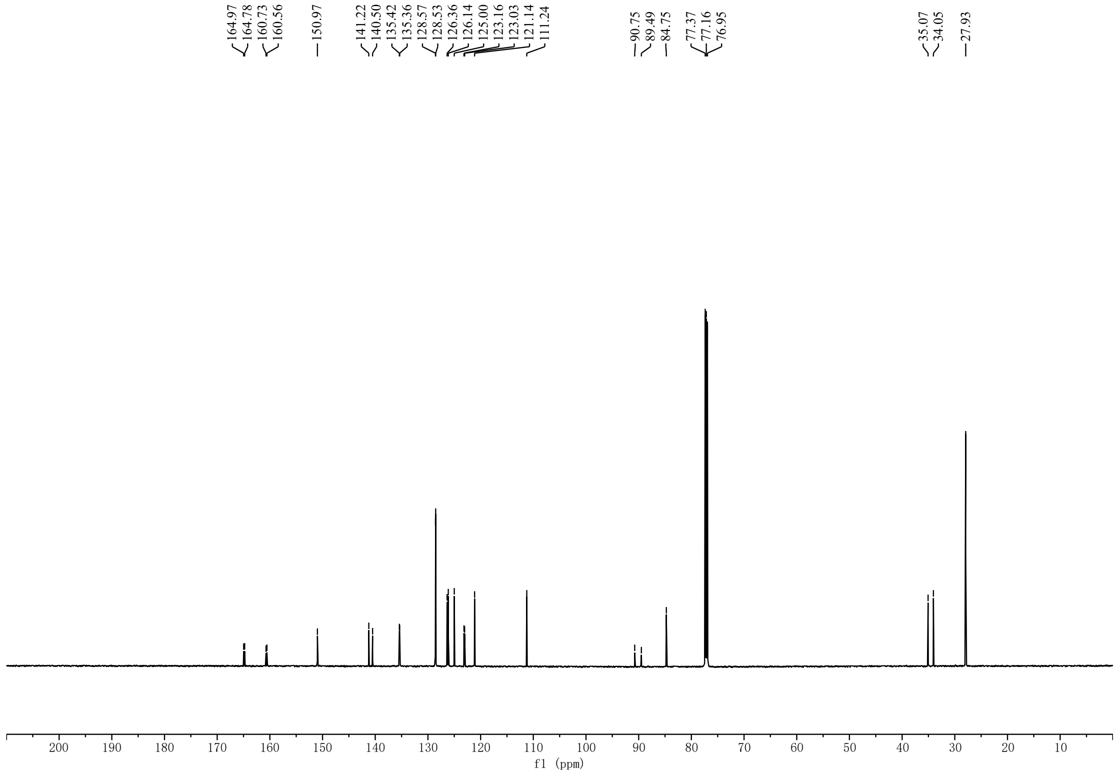

^{19}F NMR spectrum of **4m**

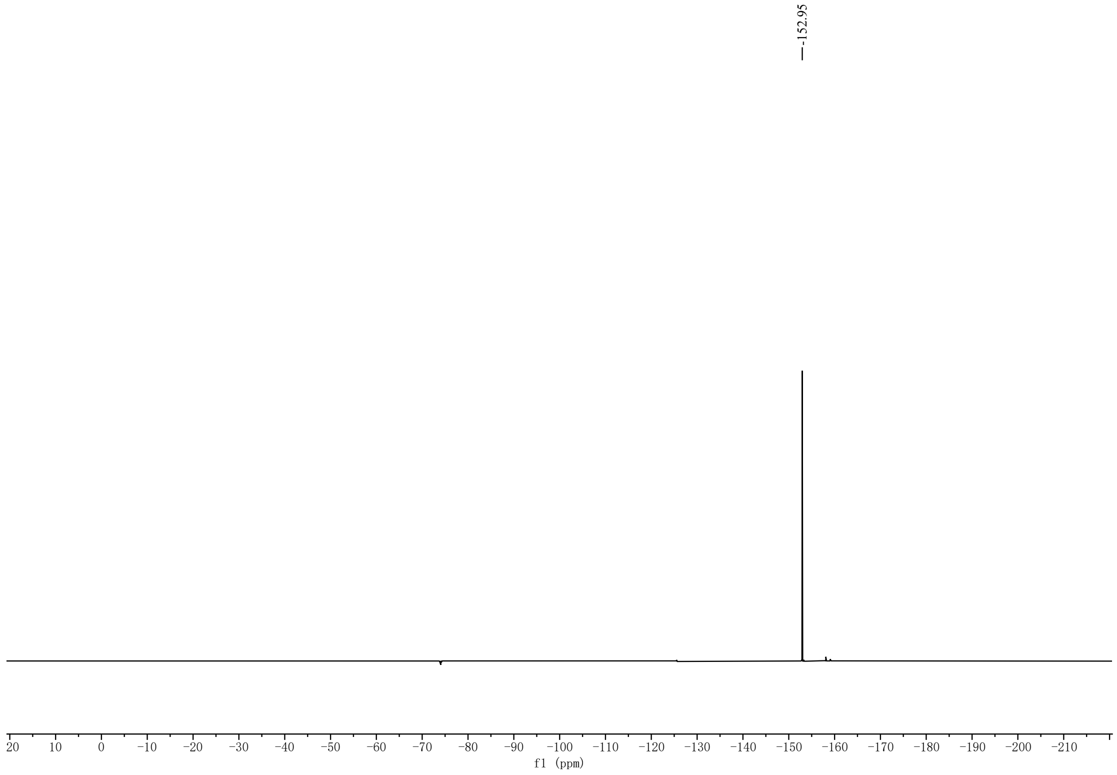

^{1}H NMR spectrum of **4n**

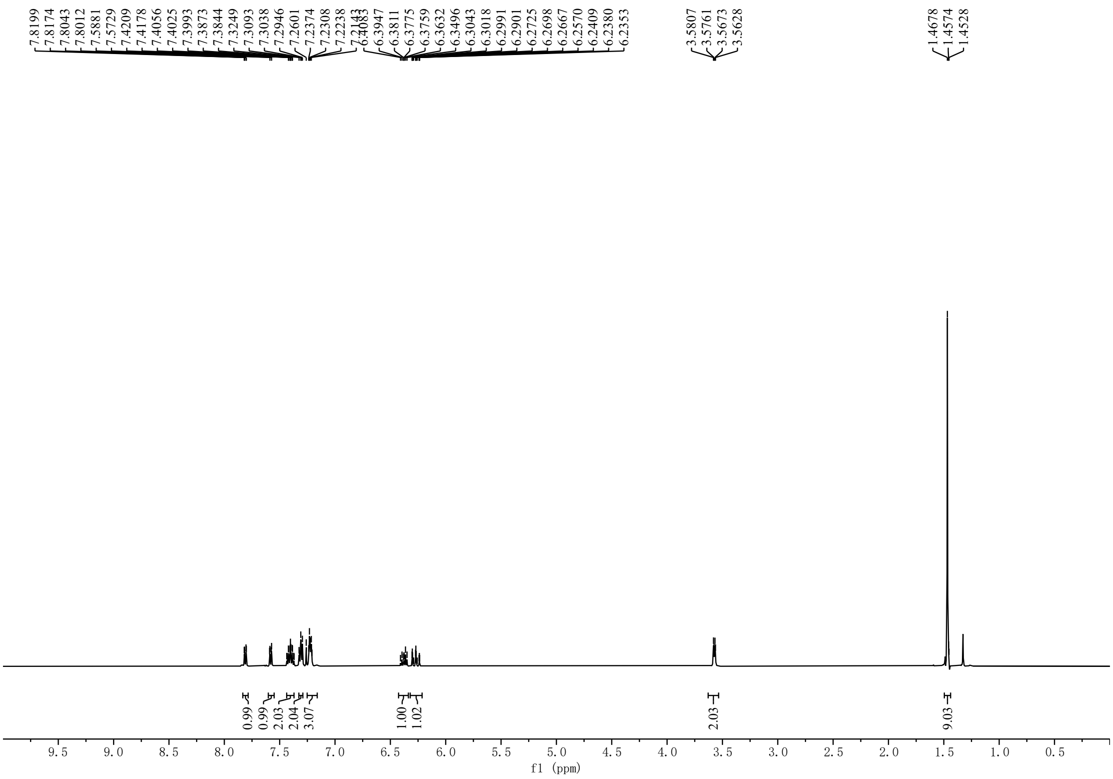

^{13}C NMR spectrum of **4n**

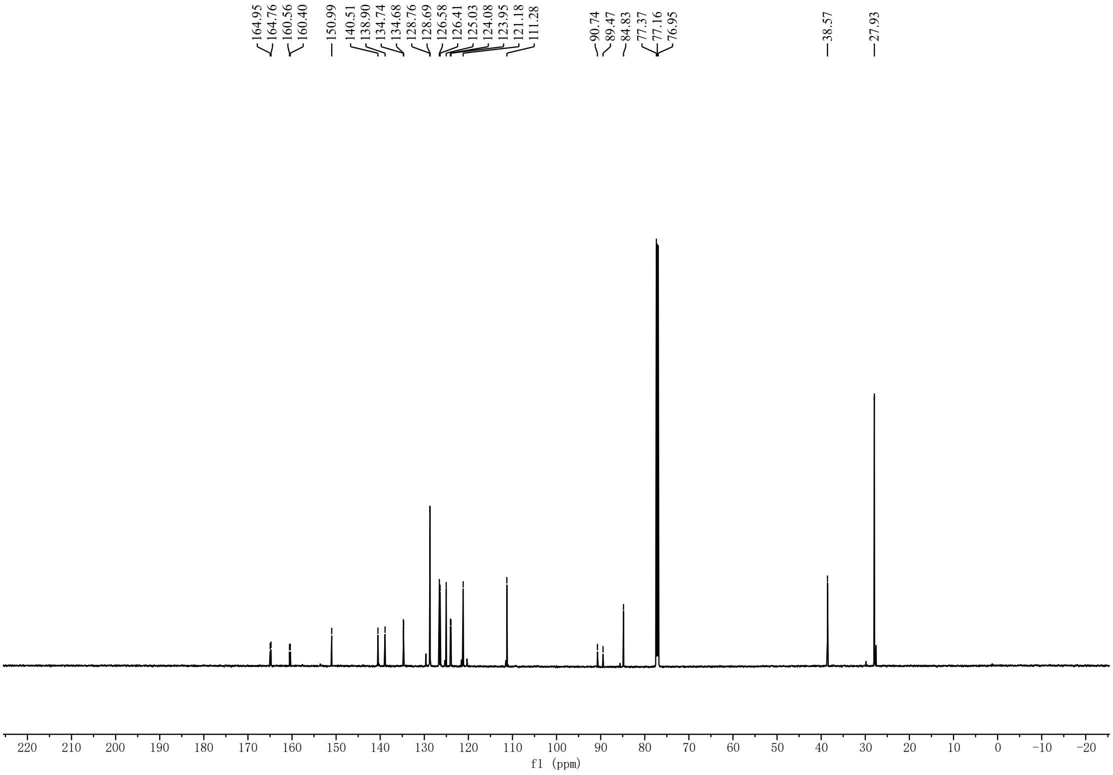

^{19}F NMR spectrum of **4n**

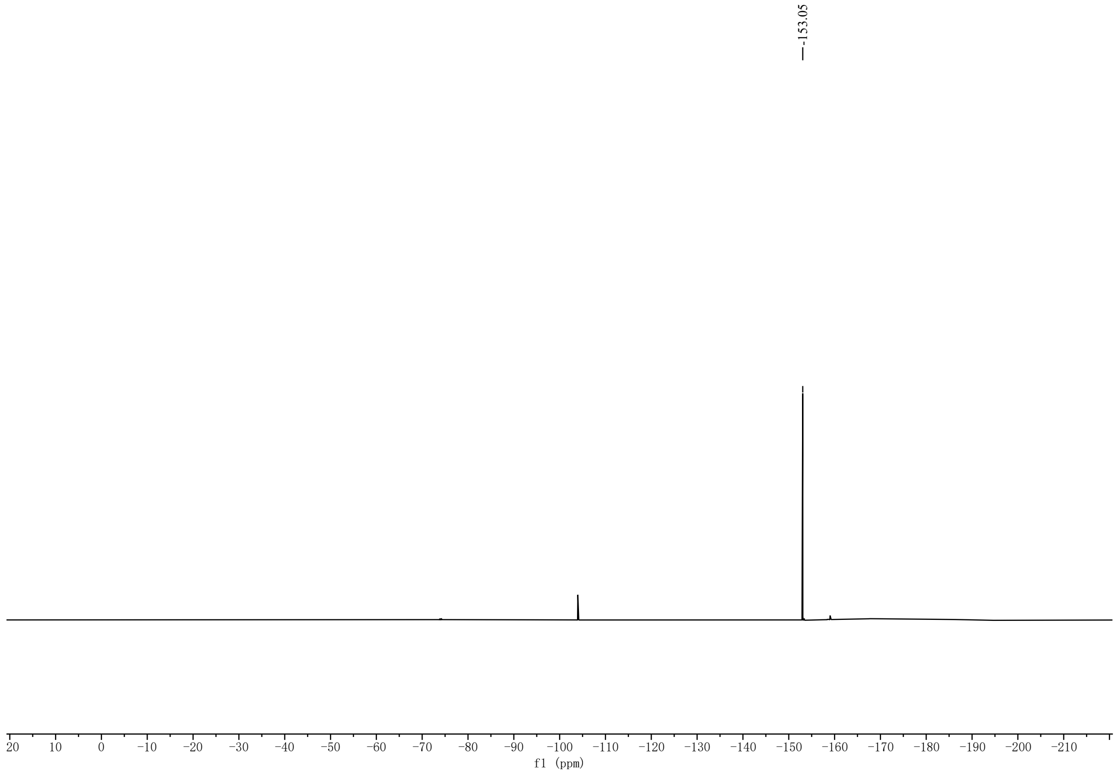

^{1}H NMR spectrum of **4o**

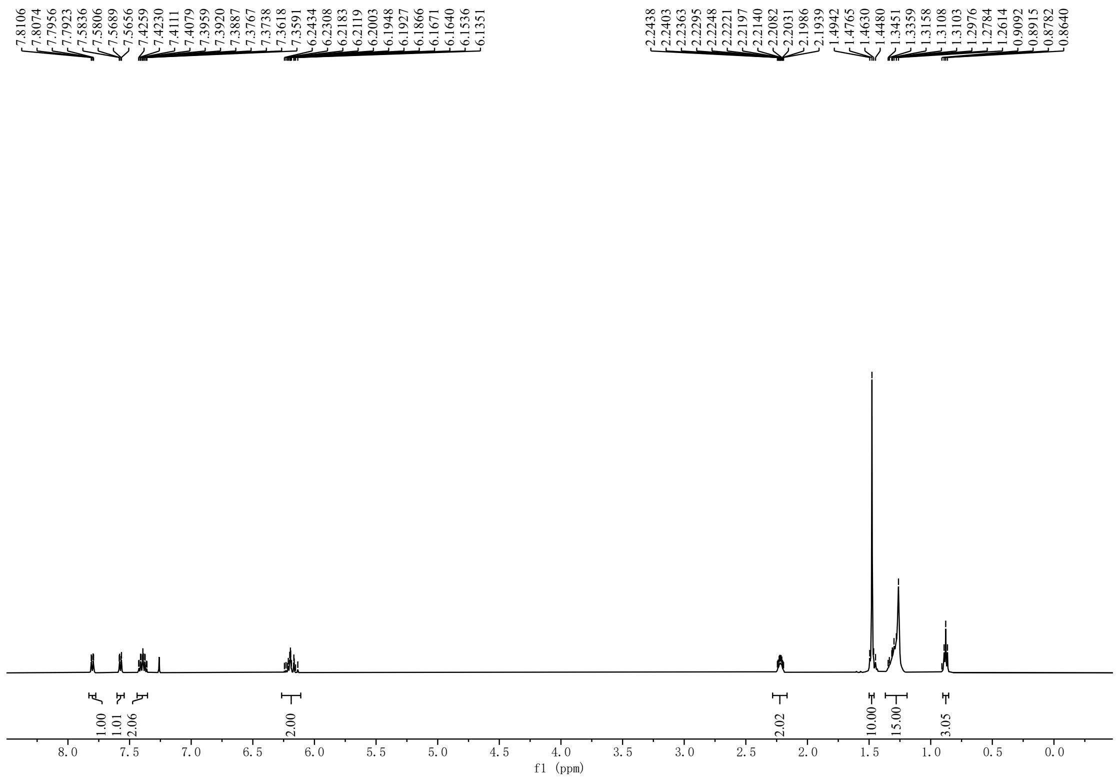

^{13}C NMR spectrum of **4o**

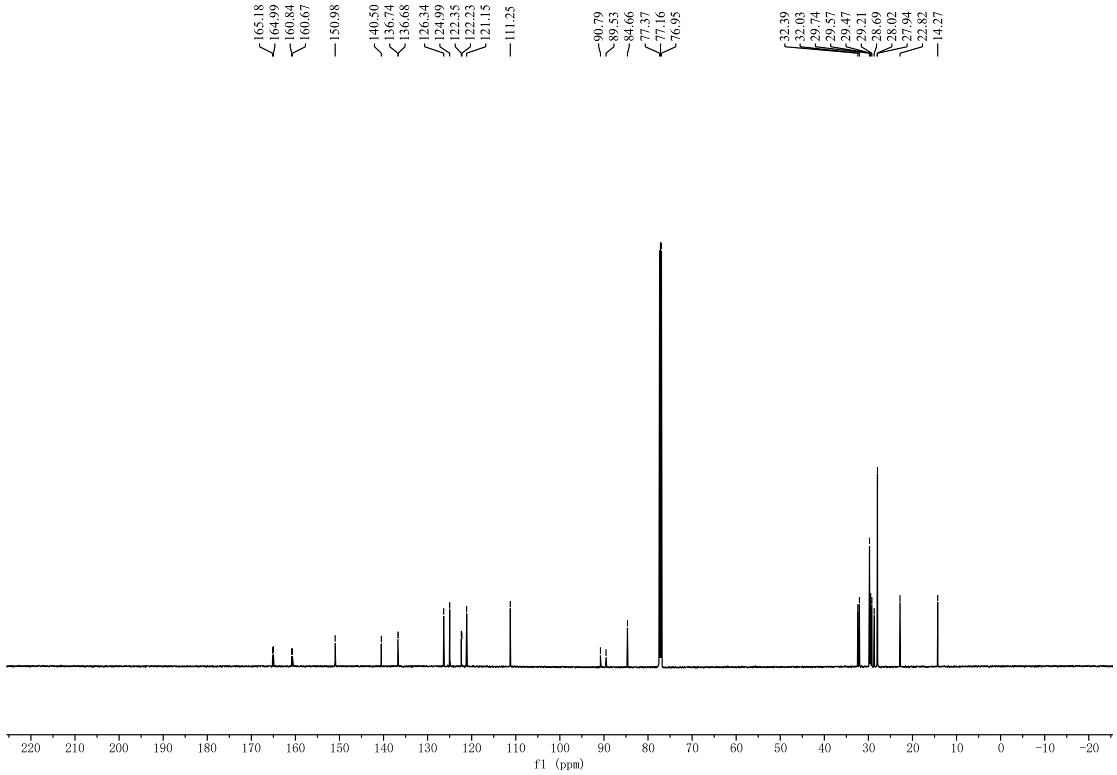

^{19}F NMR spectrum of **4o**

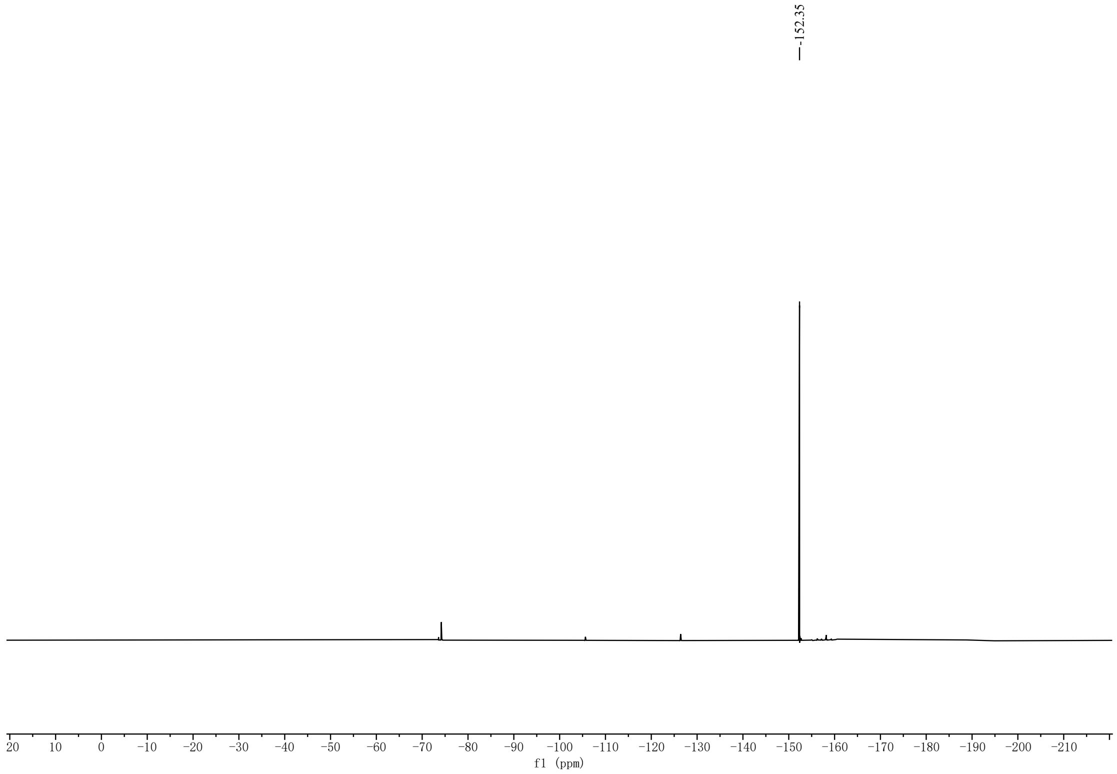

^{1}H NMR spectrum of **4p**

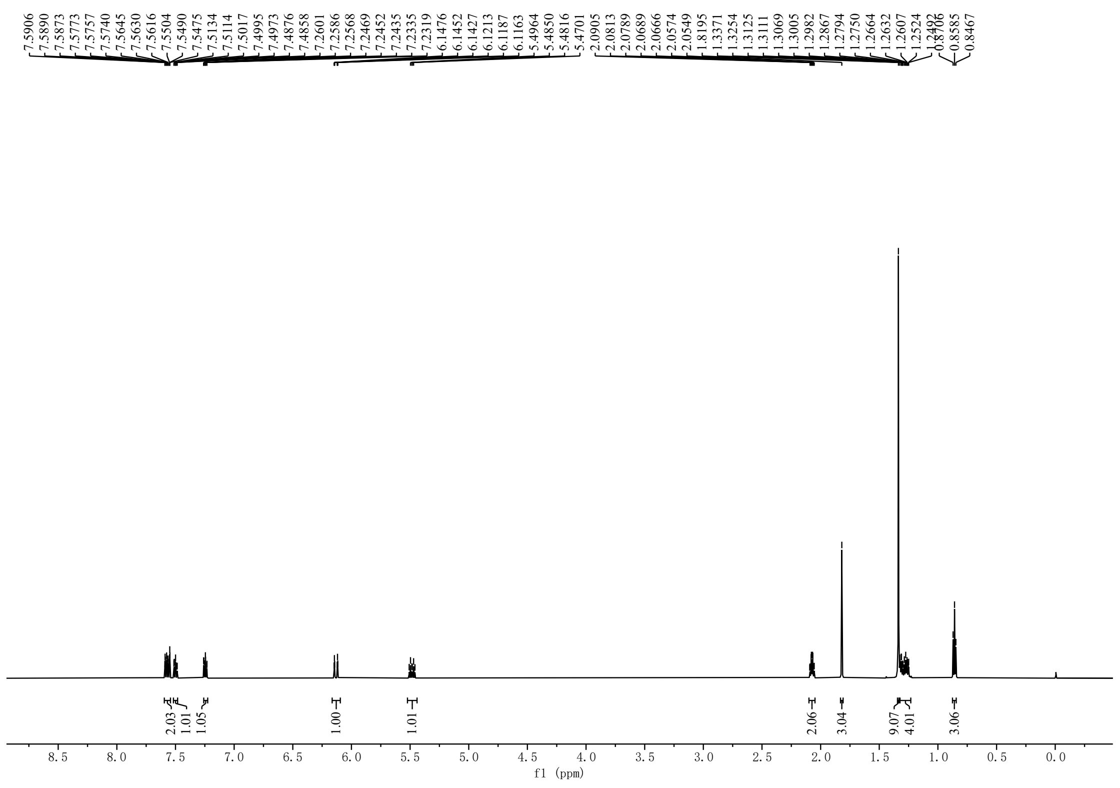

^{13}C NMR spectrum of **4p**

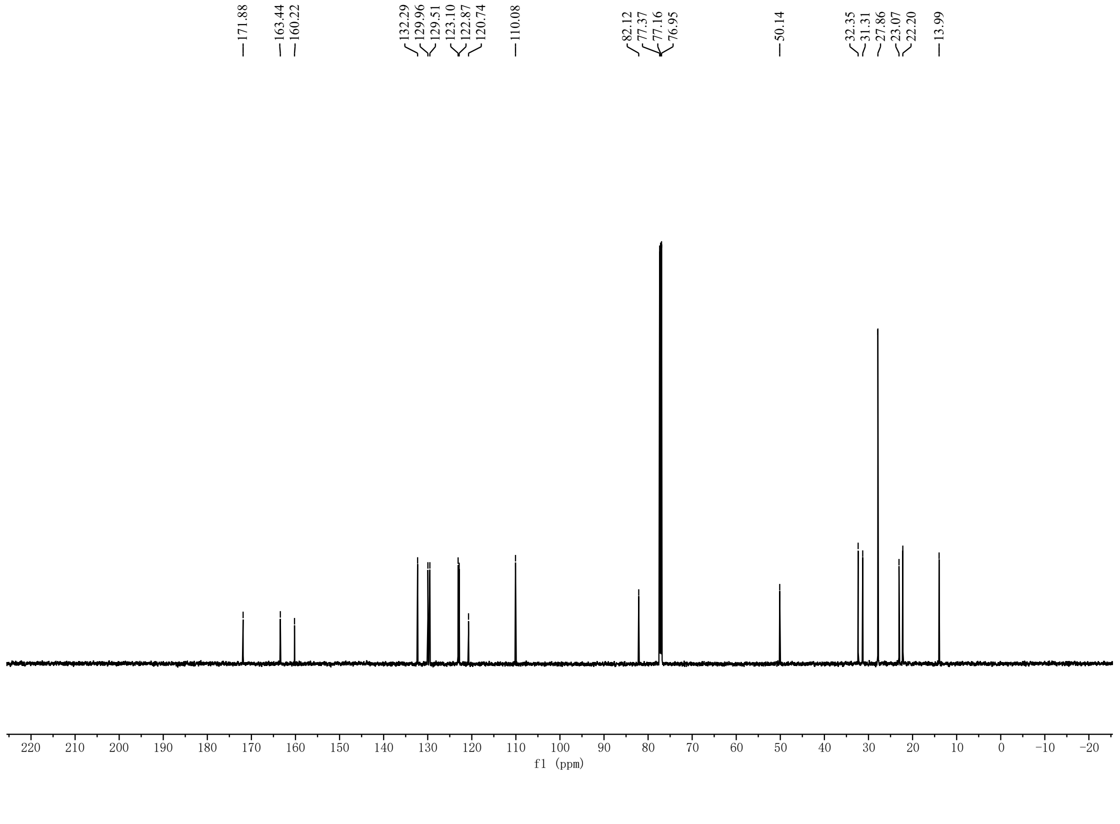

^{1}H NMR spectrum of **5a**

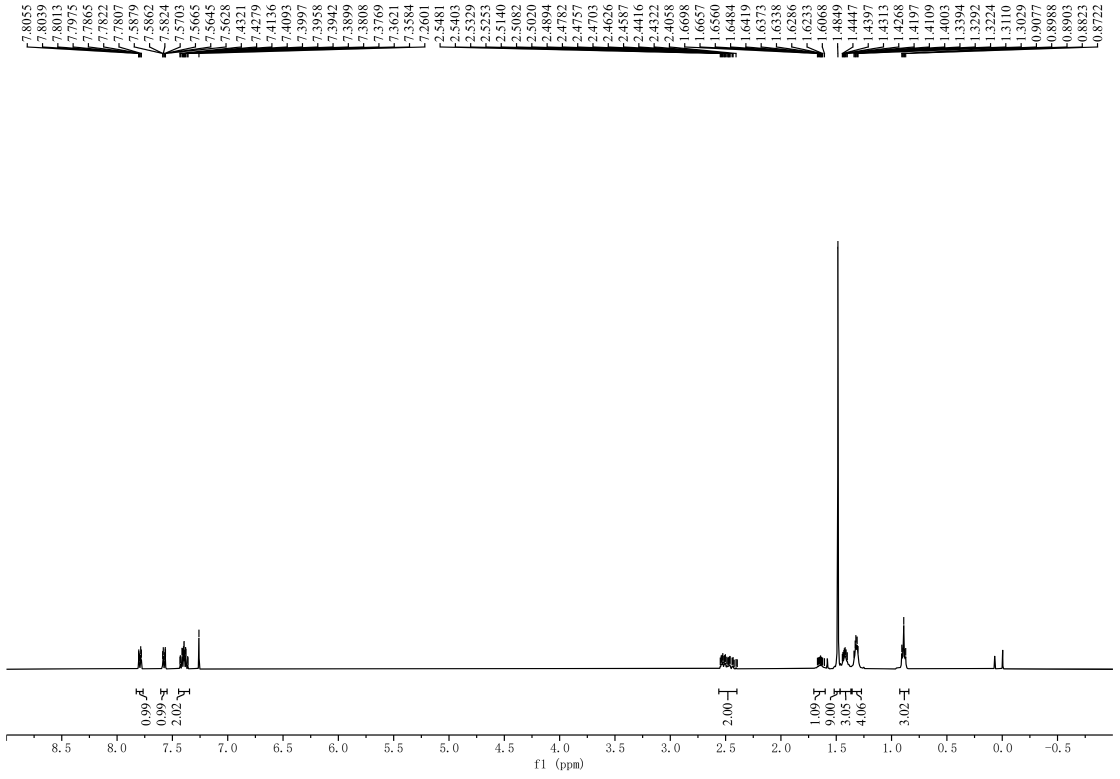

^{13}C NMR spectrum of **5a**

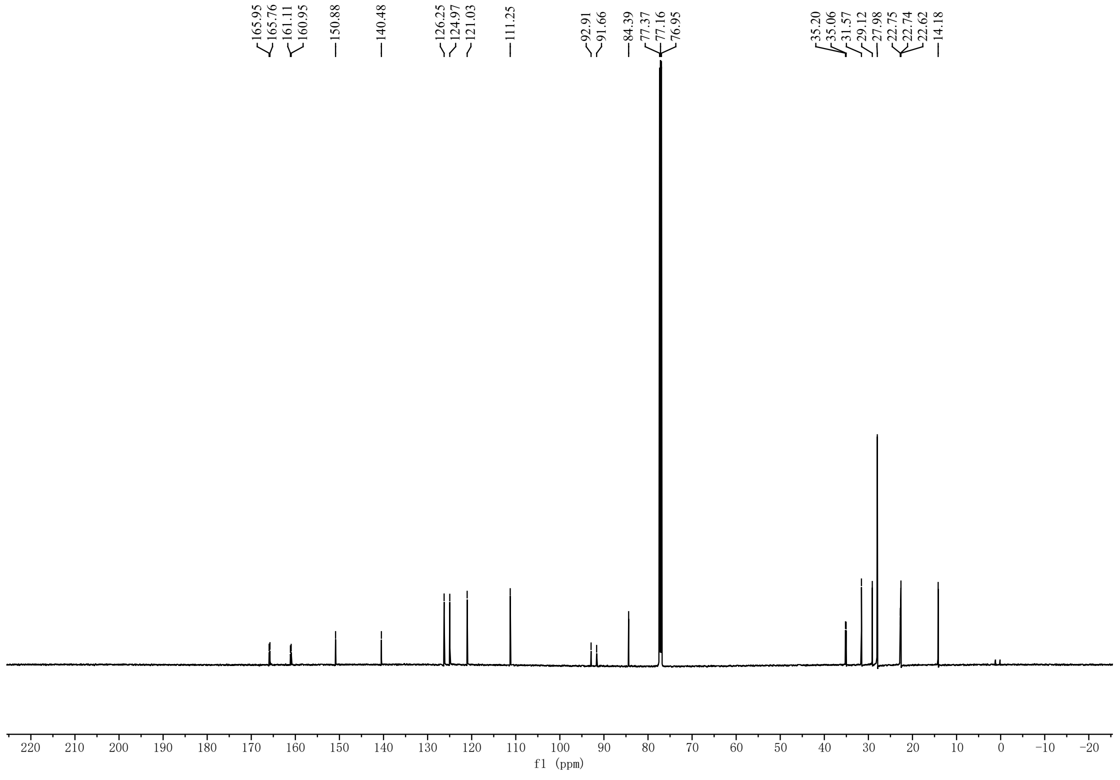

^{19}F NMR spectrum of **5a**

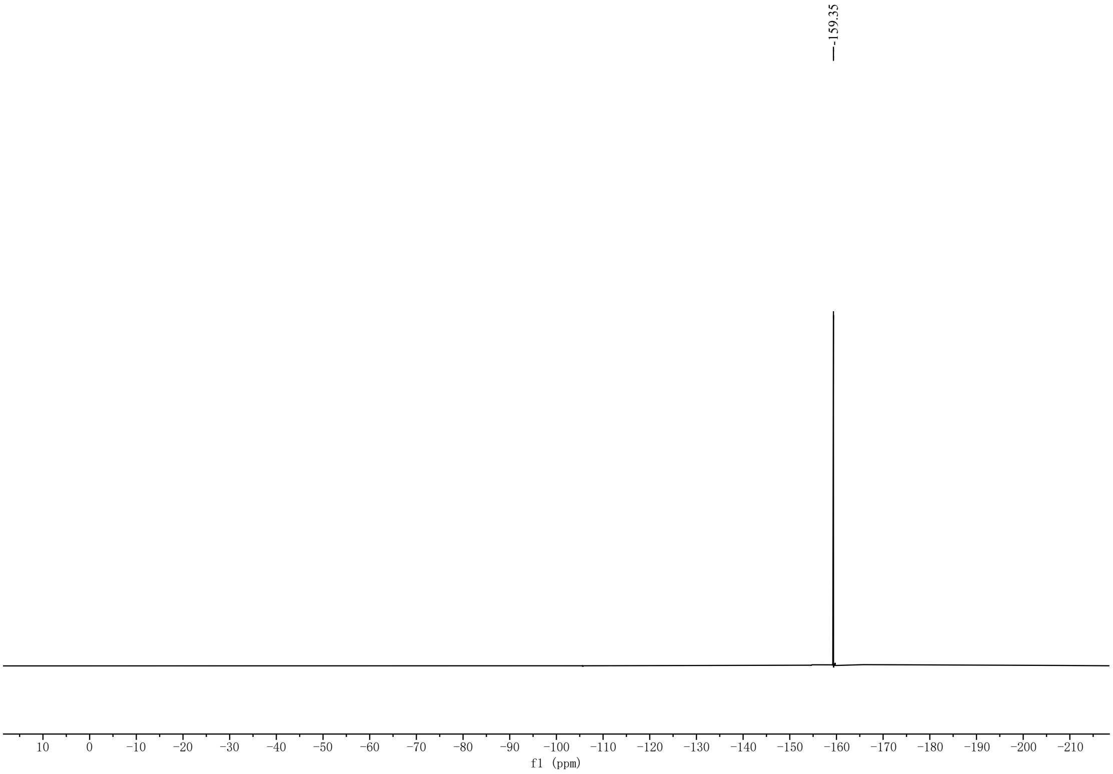

^{1}H NMR spectrum of **5b**

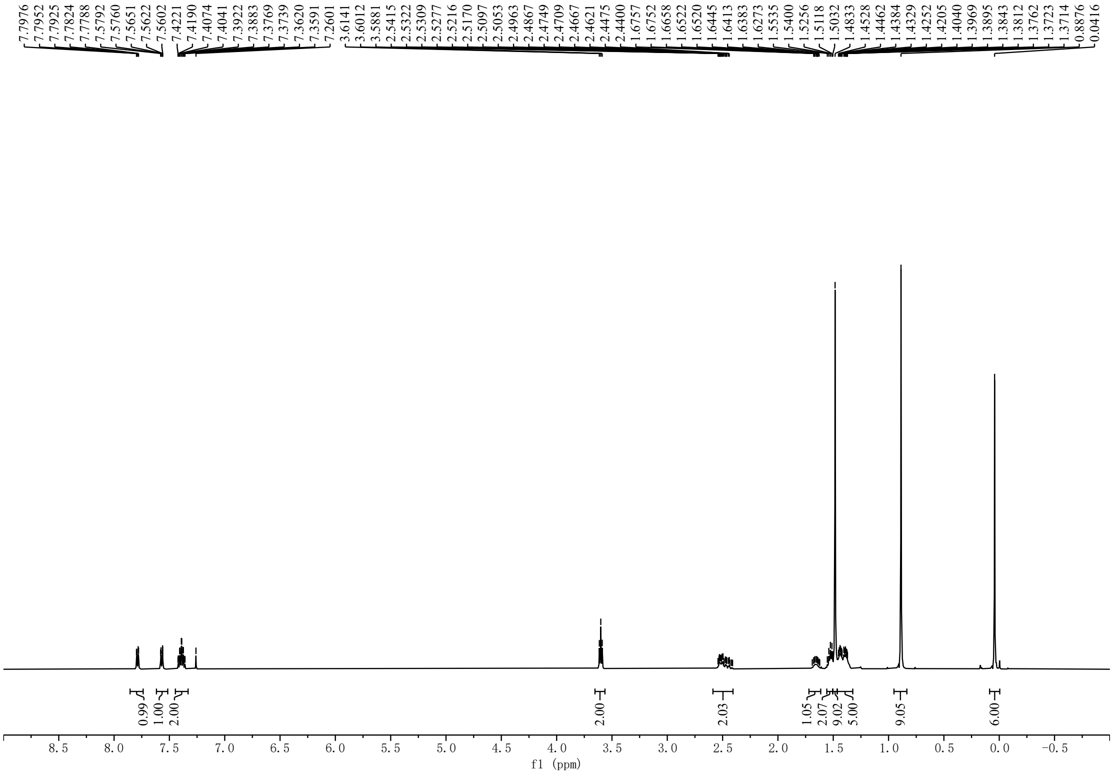

^{13}C NMR spectrum of **5b**

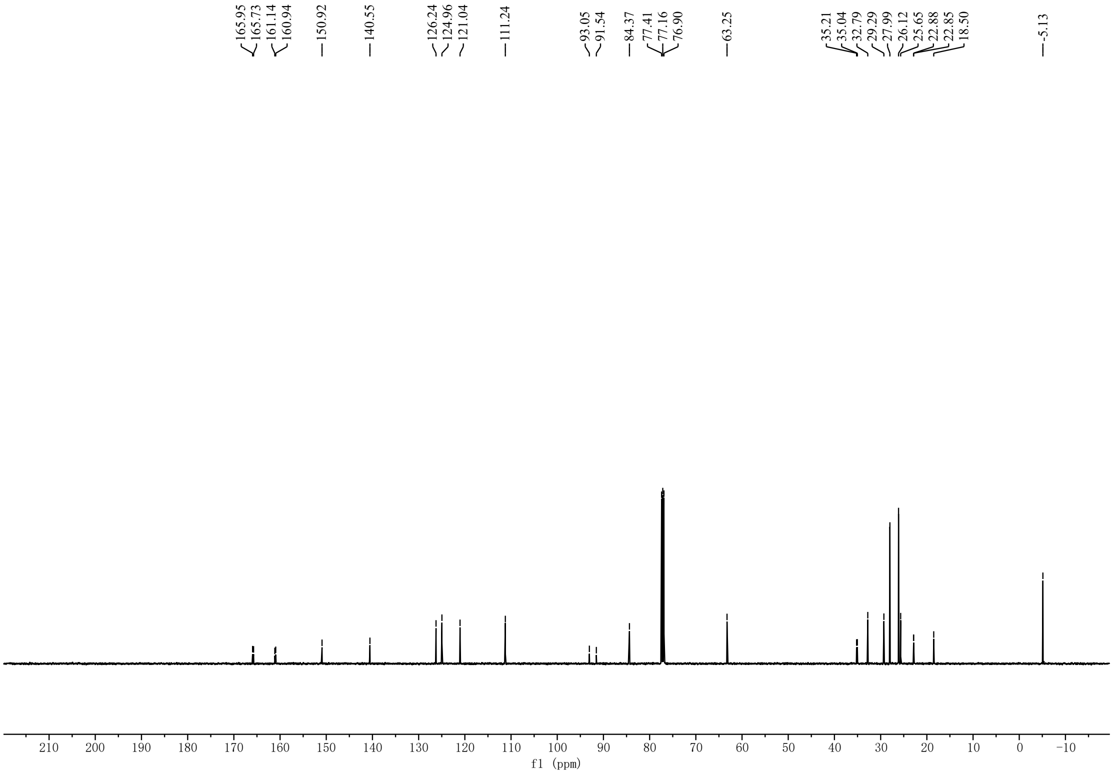

^{19}F NMR spectrum of **5b**

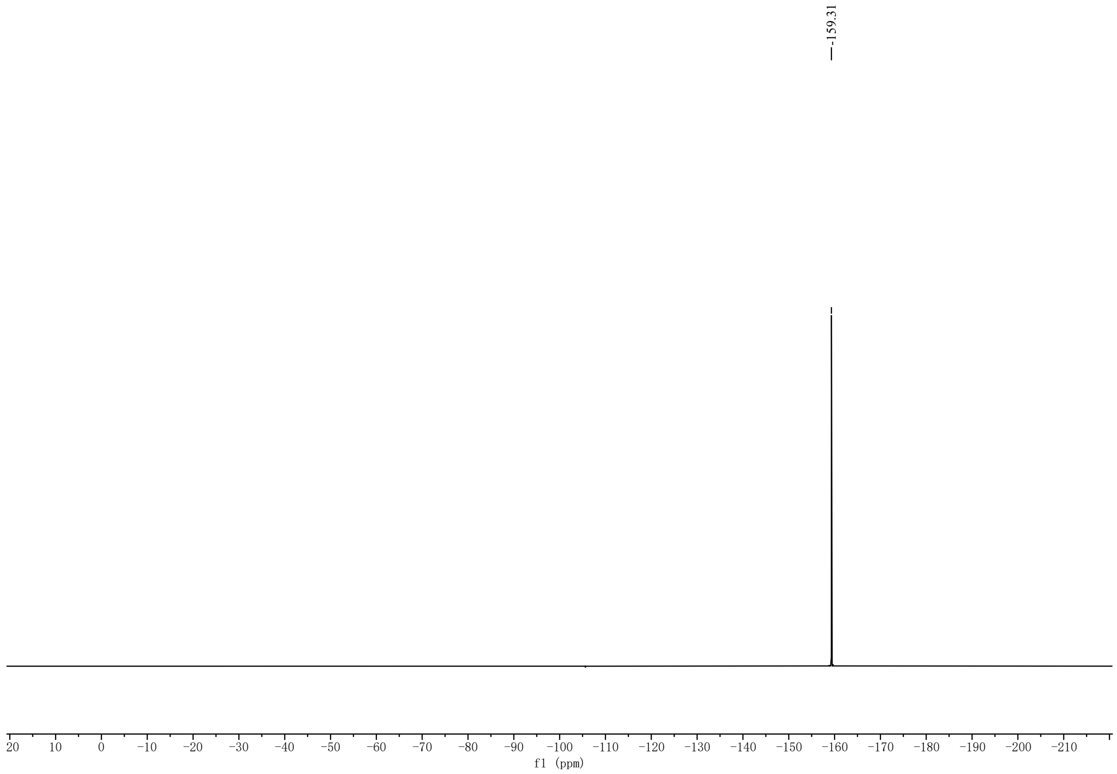

^{1}H NMR spectrum of **5c**

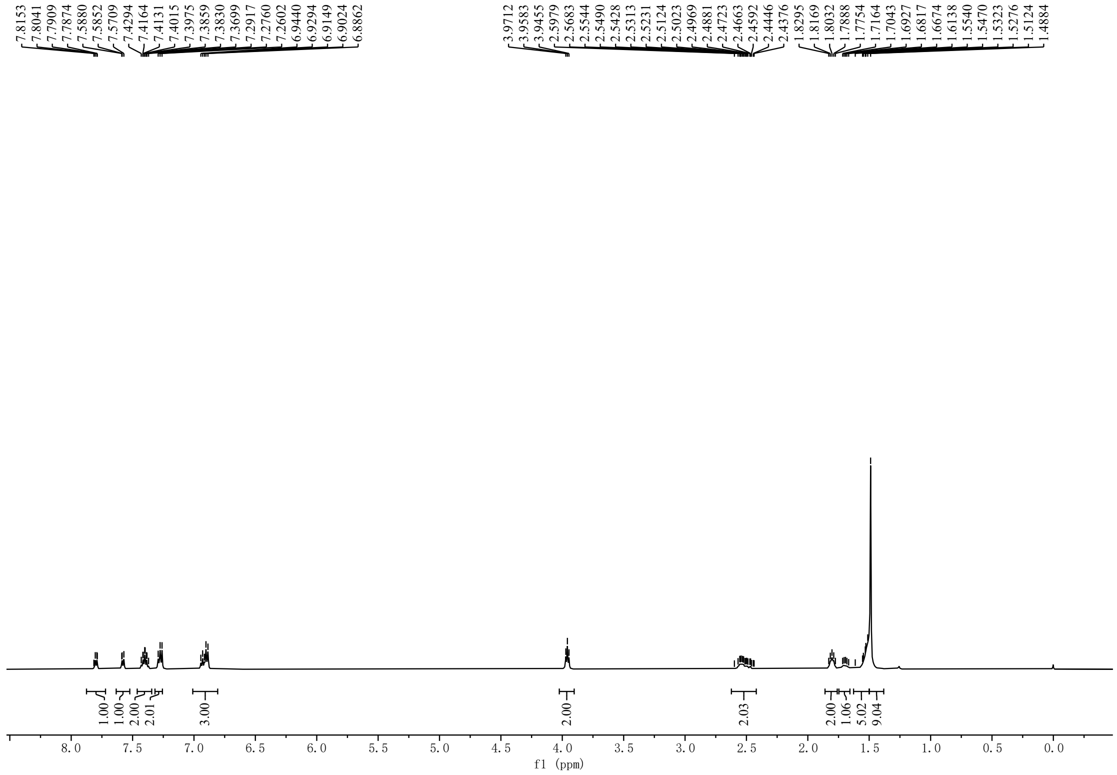

^{13}C NMR spectrum of **5c**

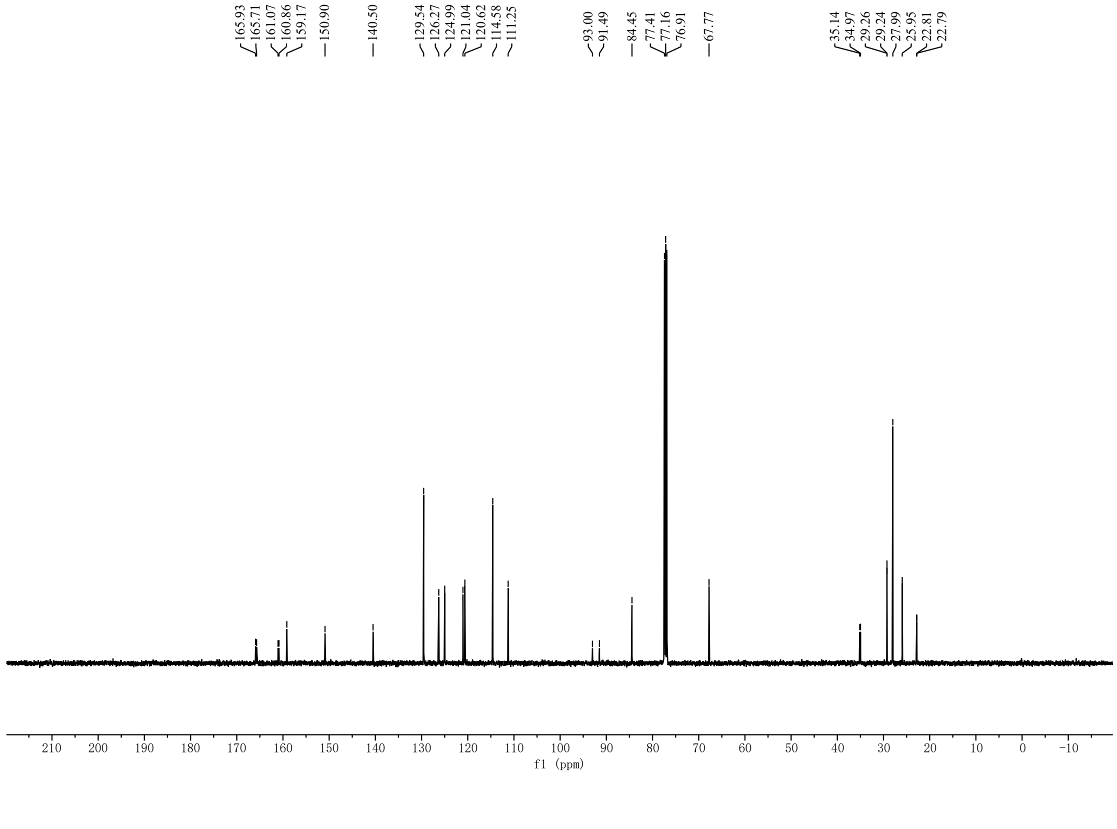

^{19}F NMR spectrum of **5c**

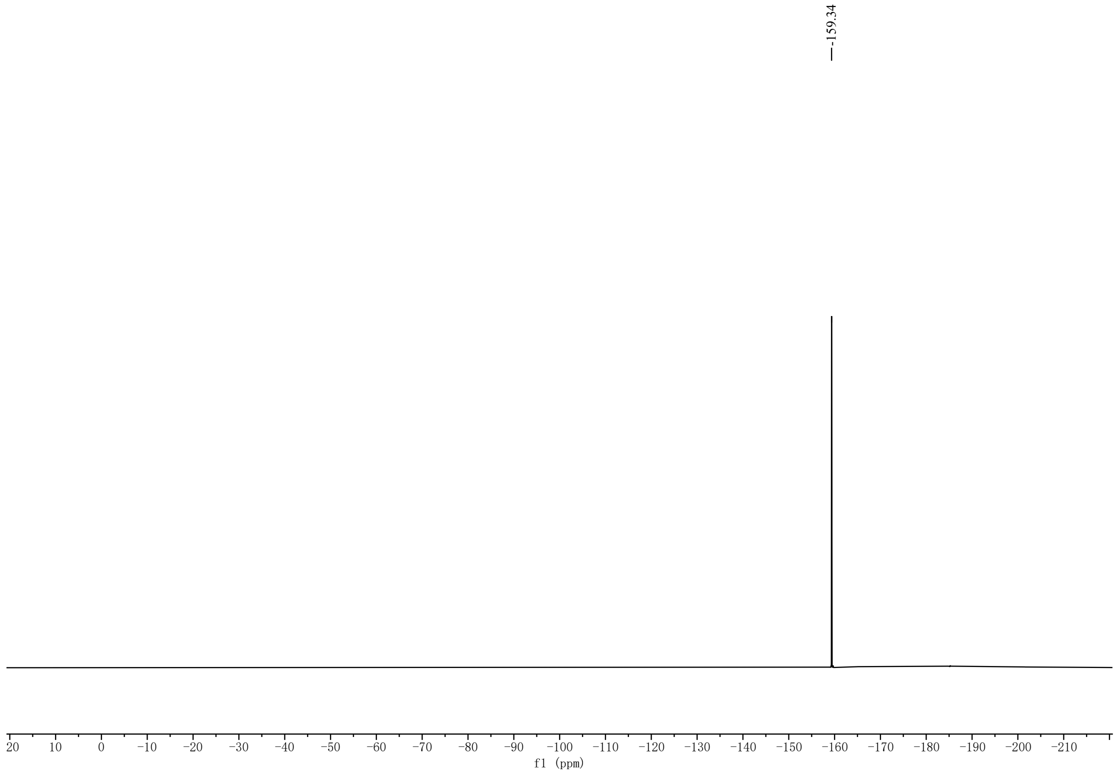

^{1}H NMR spectrum of **5d**

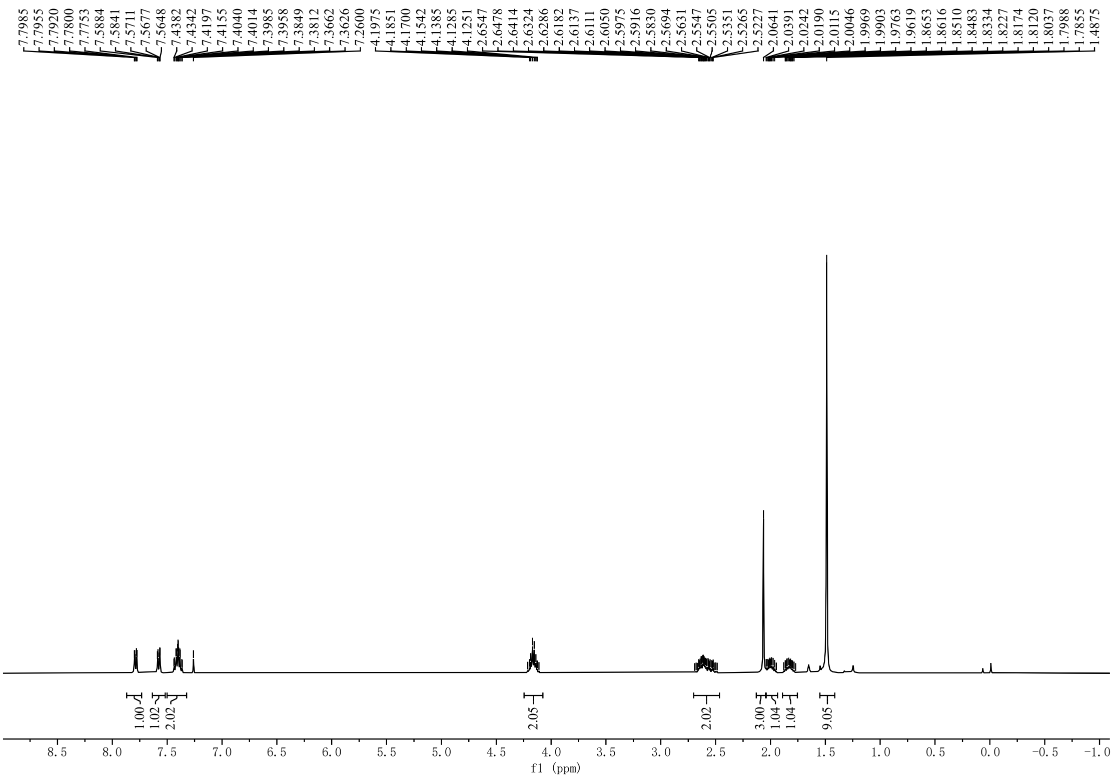

^{13}C NMR spectrum of **5d**

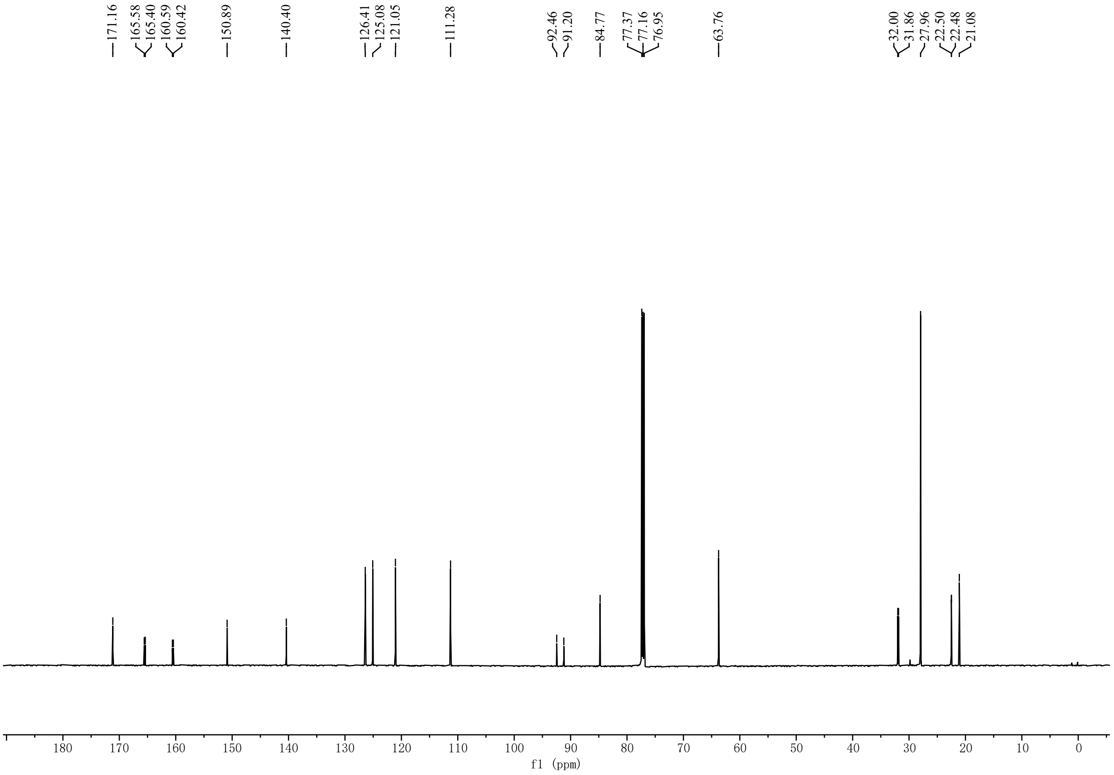

^{19}F NMR spectrum of **5d**

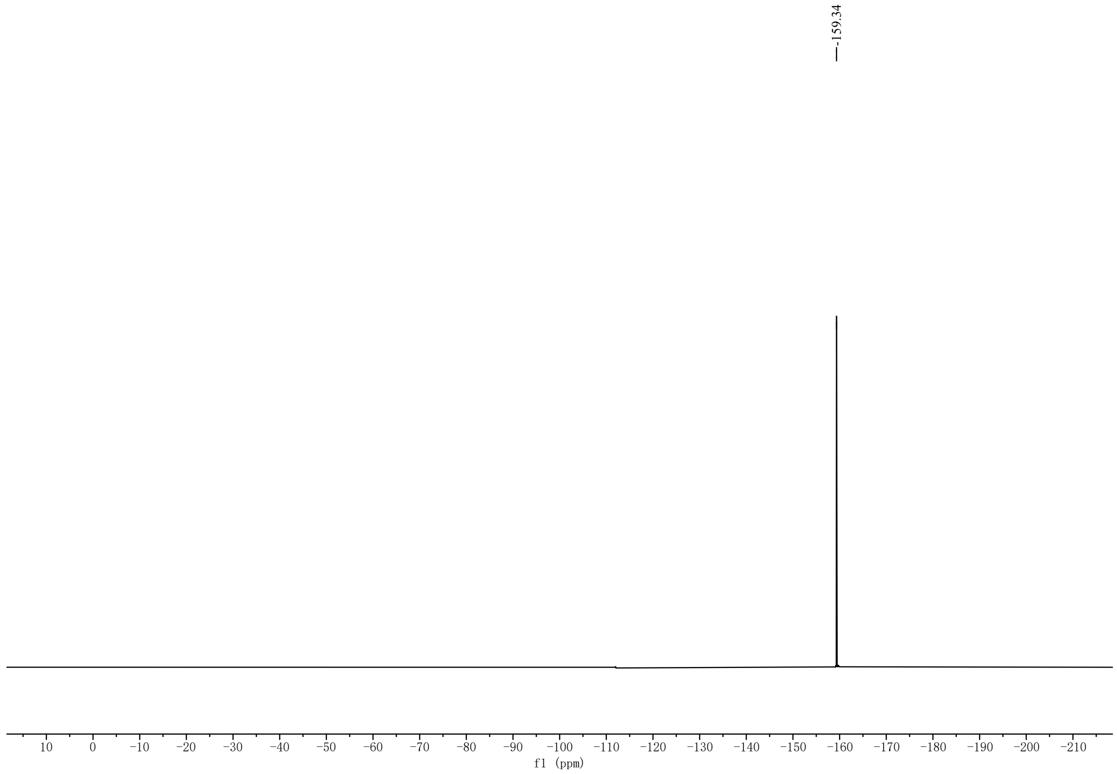

^{1}**H NMR** spectrum of **5e**

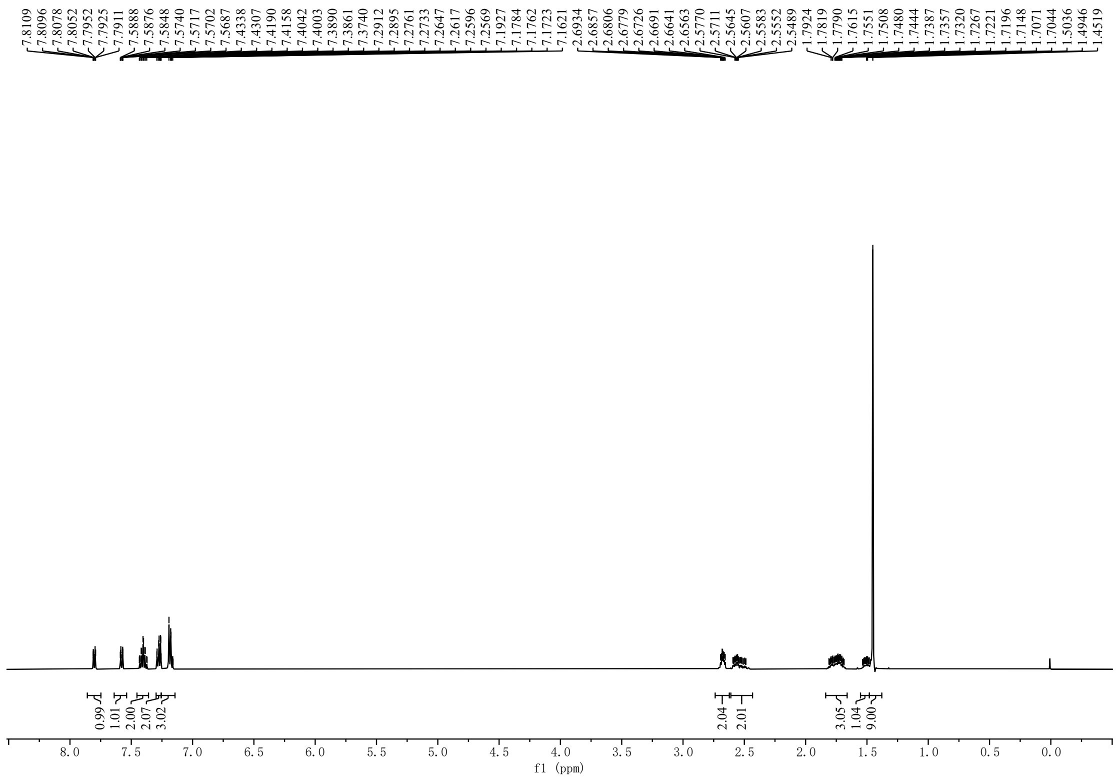

^{13}**C NMR** spectrum of **5e**

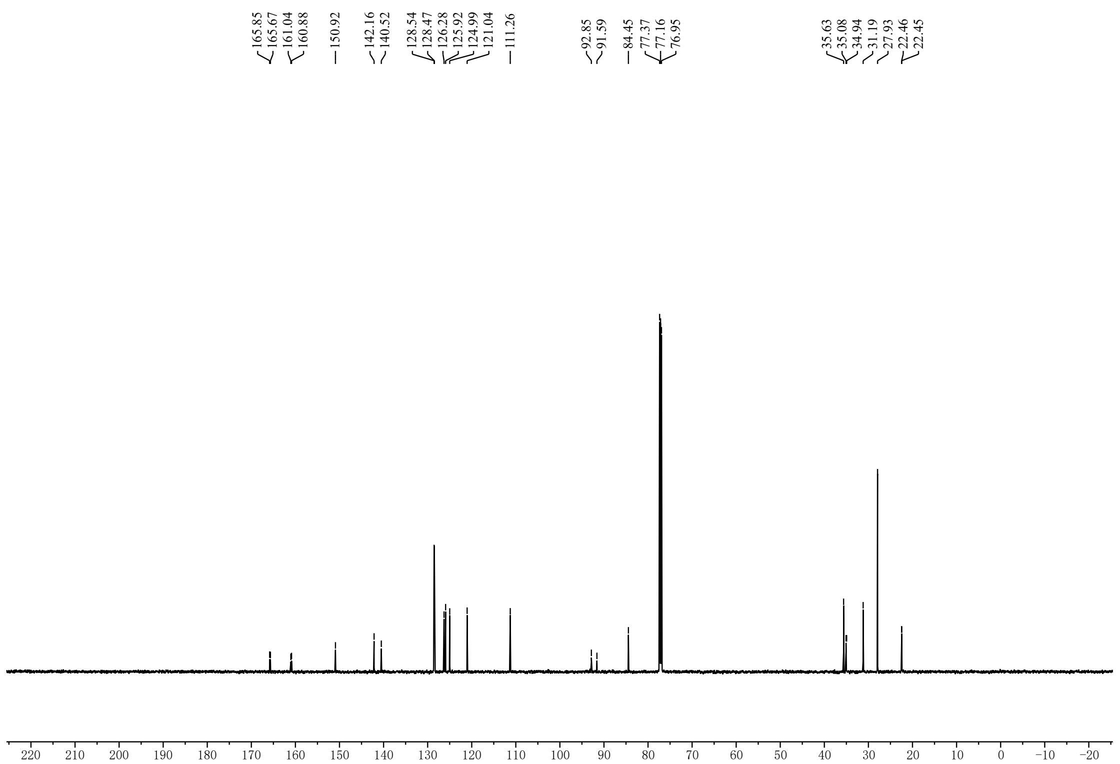

^{19}F NMR spectrum of **5e**

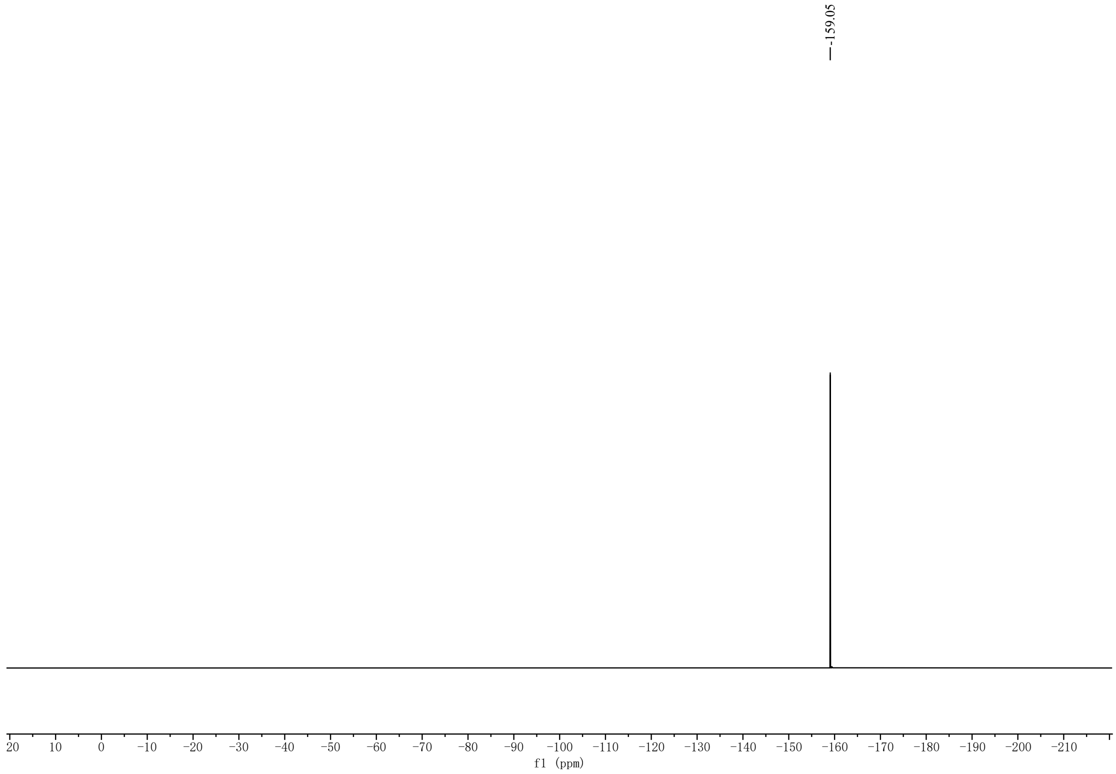

^{1}H NMR spectrum of **5f**

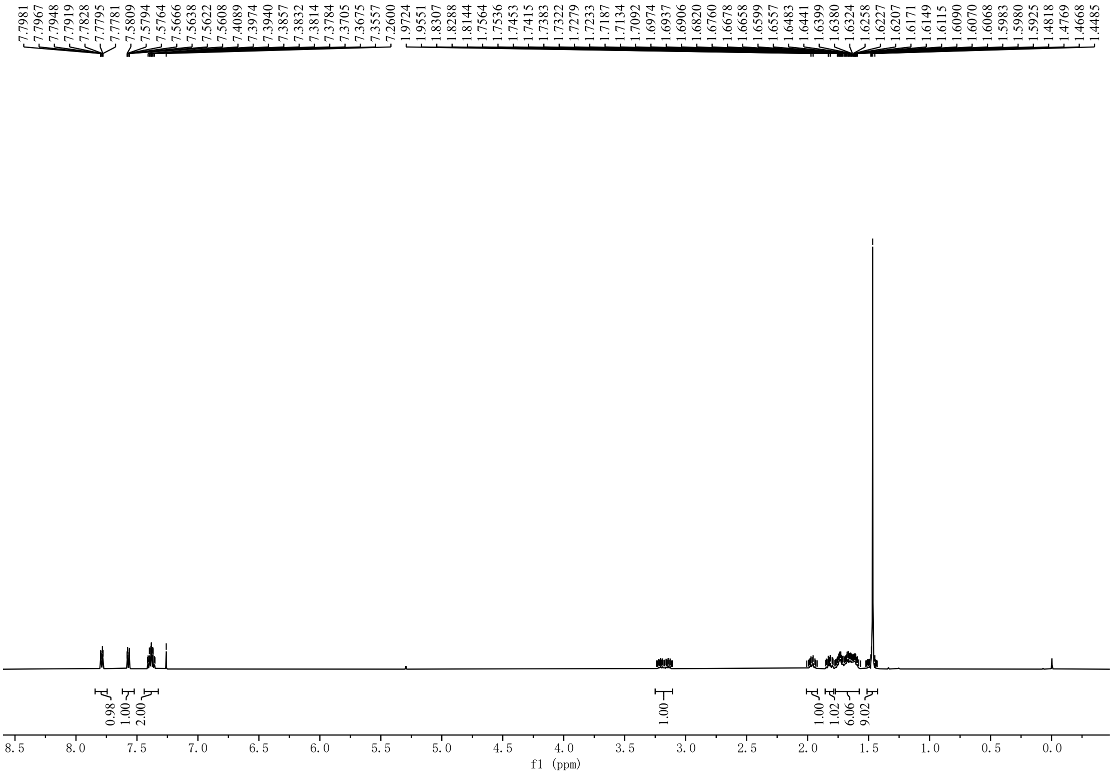

^{13}C NMR spectrum of **5f**

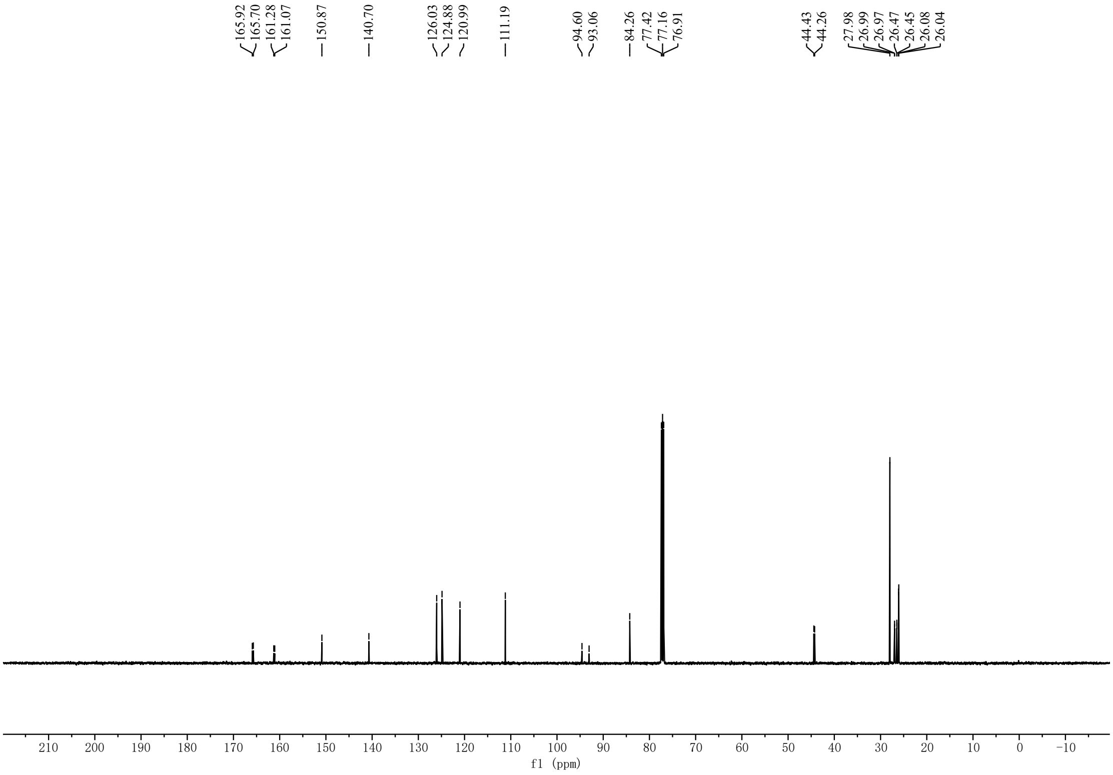

^{19}**F NMR** spectrum of **5f**

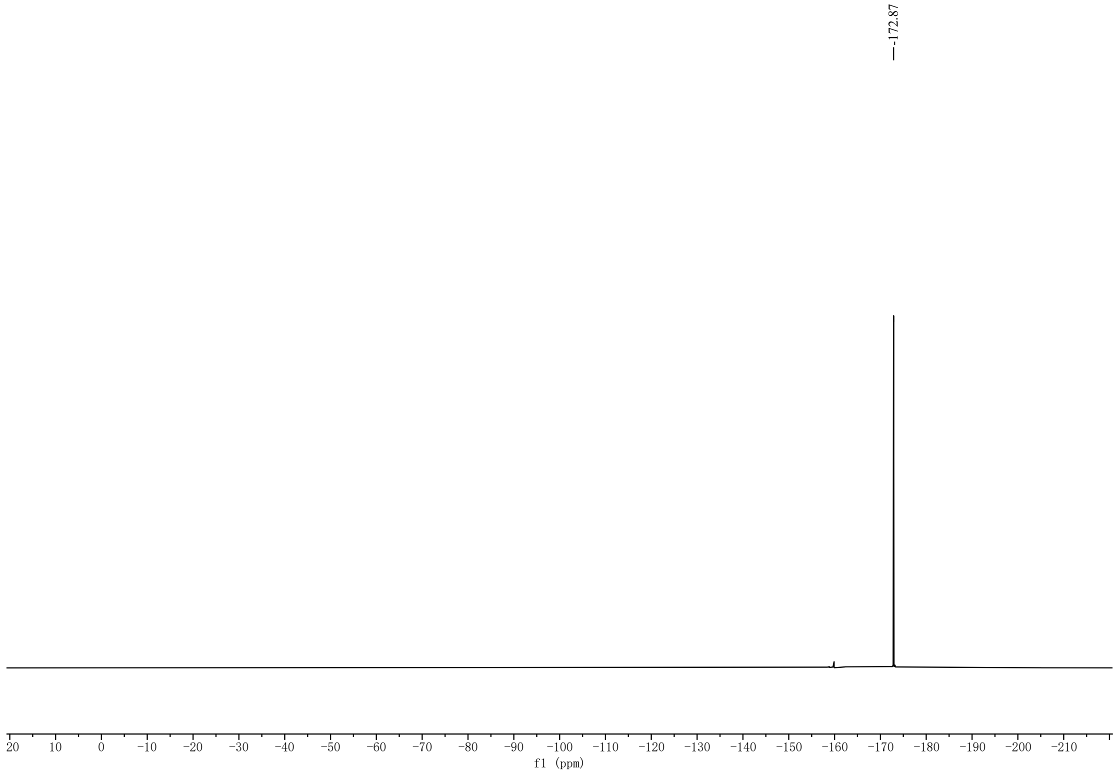

^{1}H NMR spectrum of **5g**

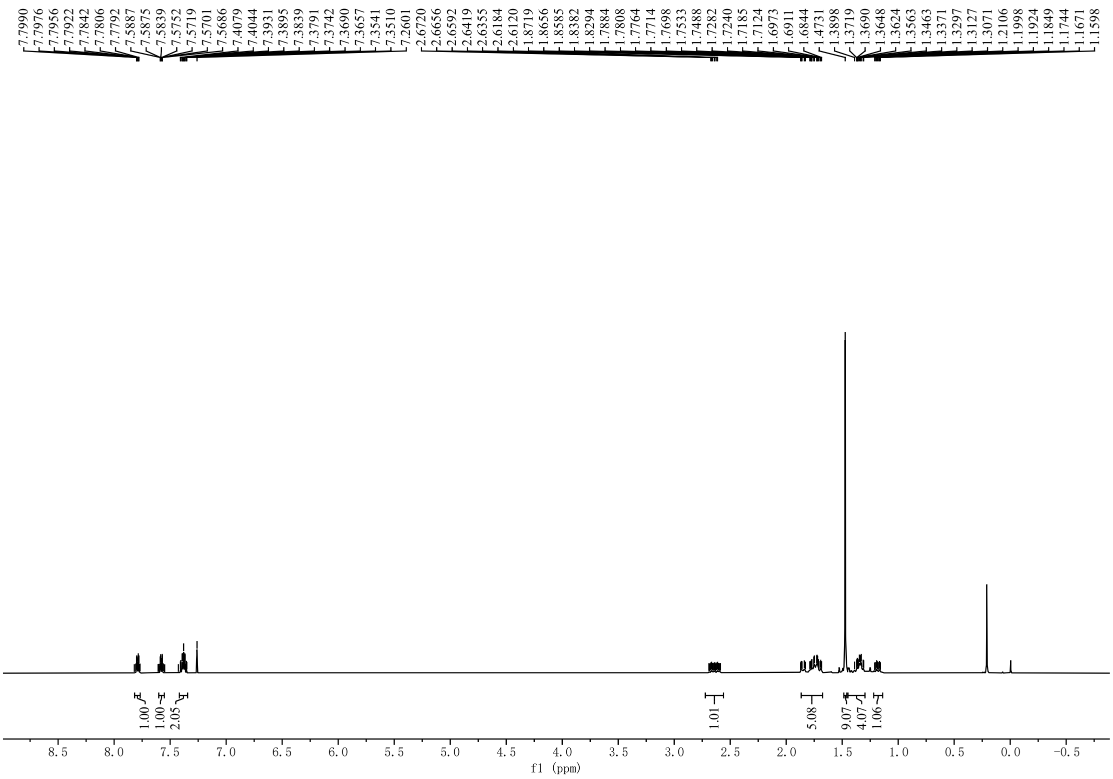

^{13}C NMR spectrum of **5g**

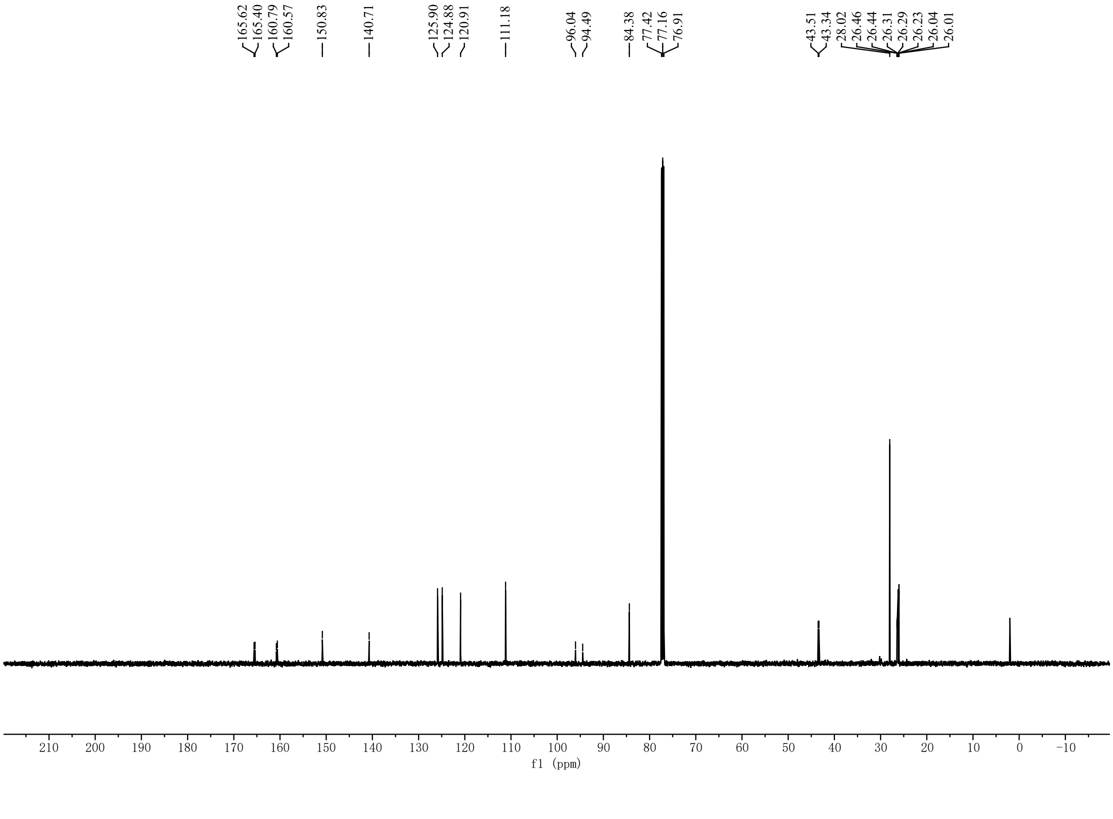

^{19}F NMR spectrum of **5g**

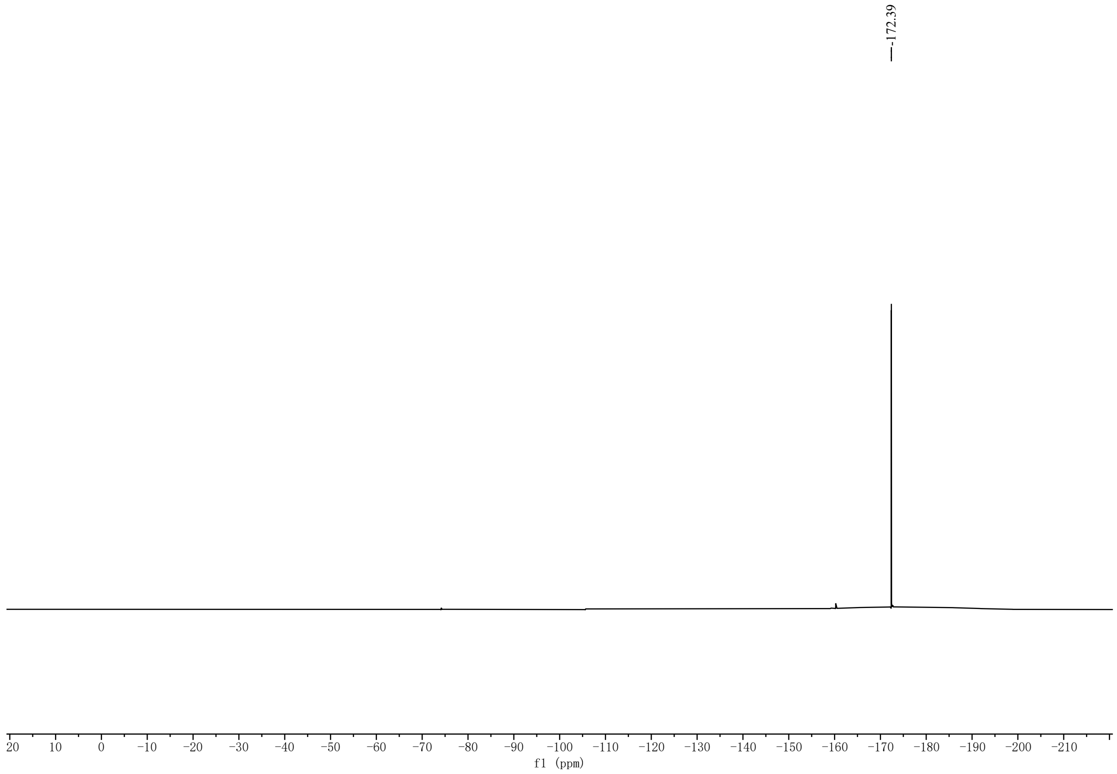

^{1}H NMR spectrum of **5h**

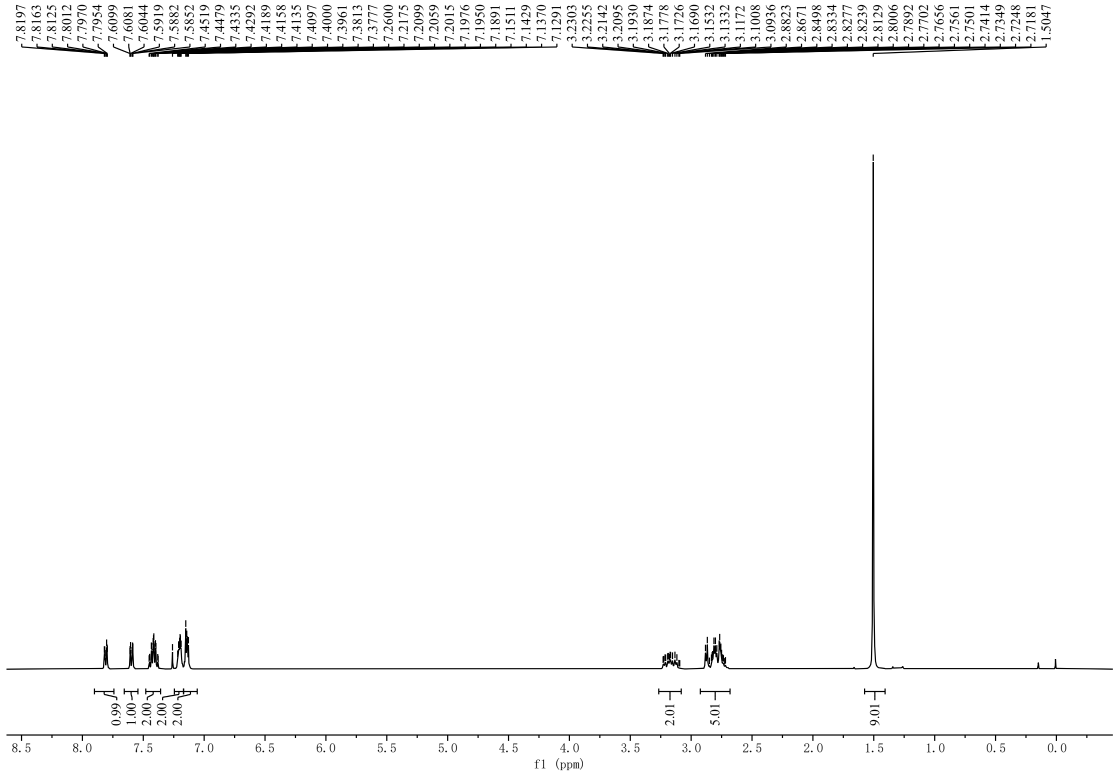

^{13}C NMR spectrum of **5h**

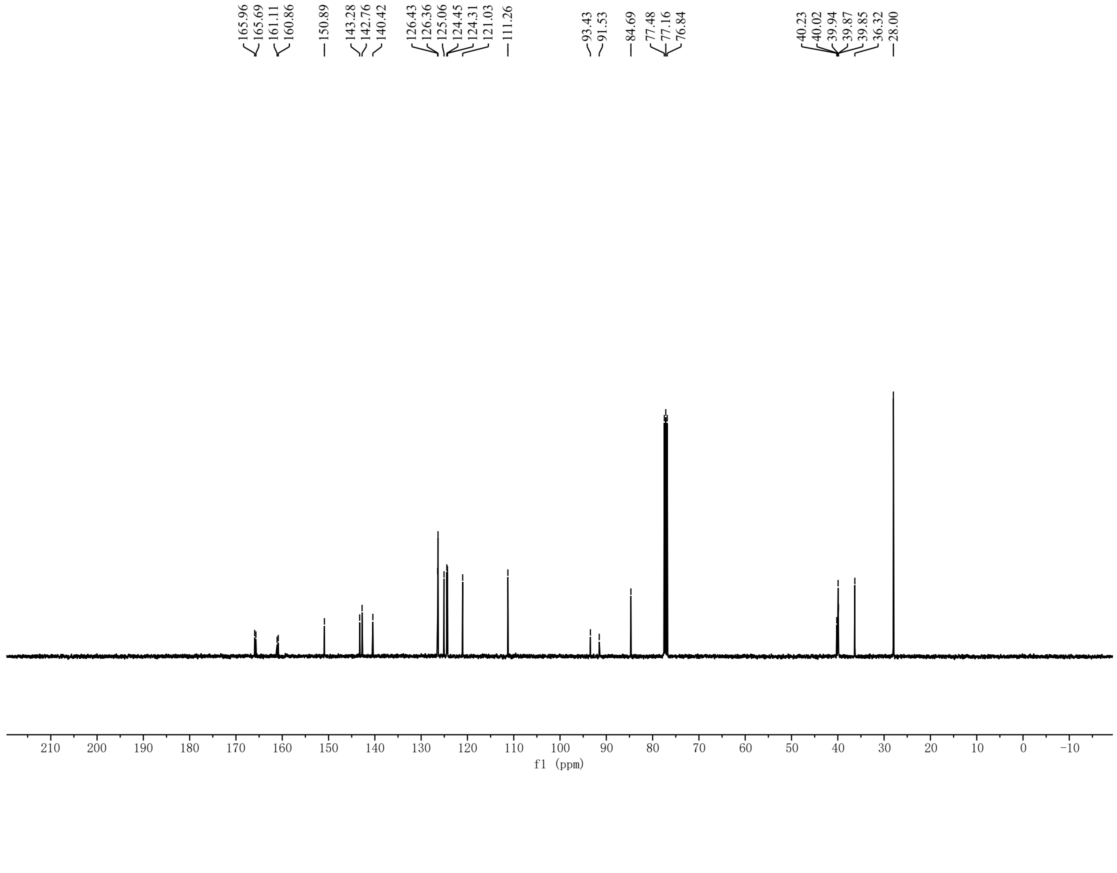

^{19}**F NMR** spectrum of **5h**

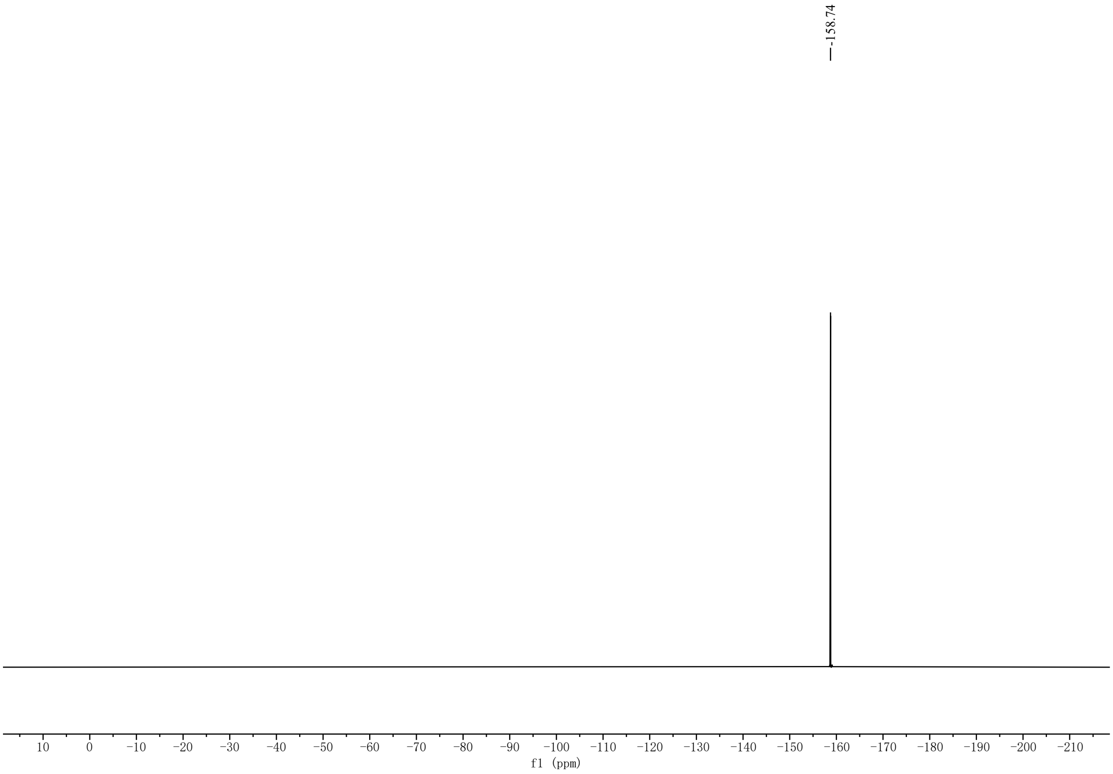

^{1}H NMR spectrum of **5i**

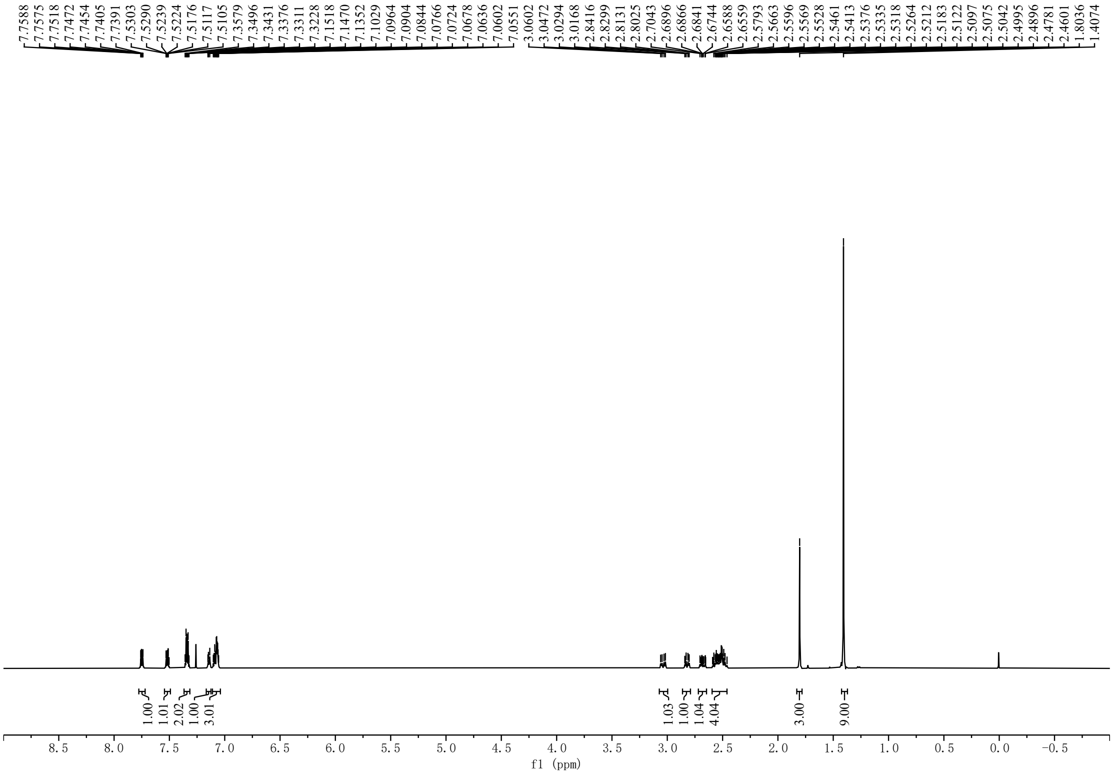

^{13}C NMR spectrum of **5i**

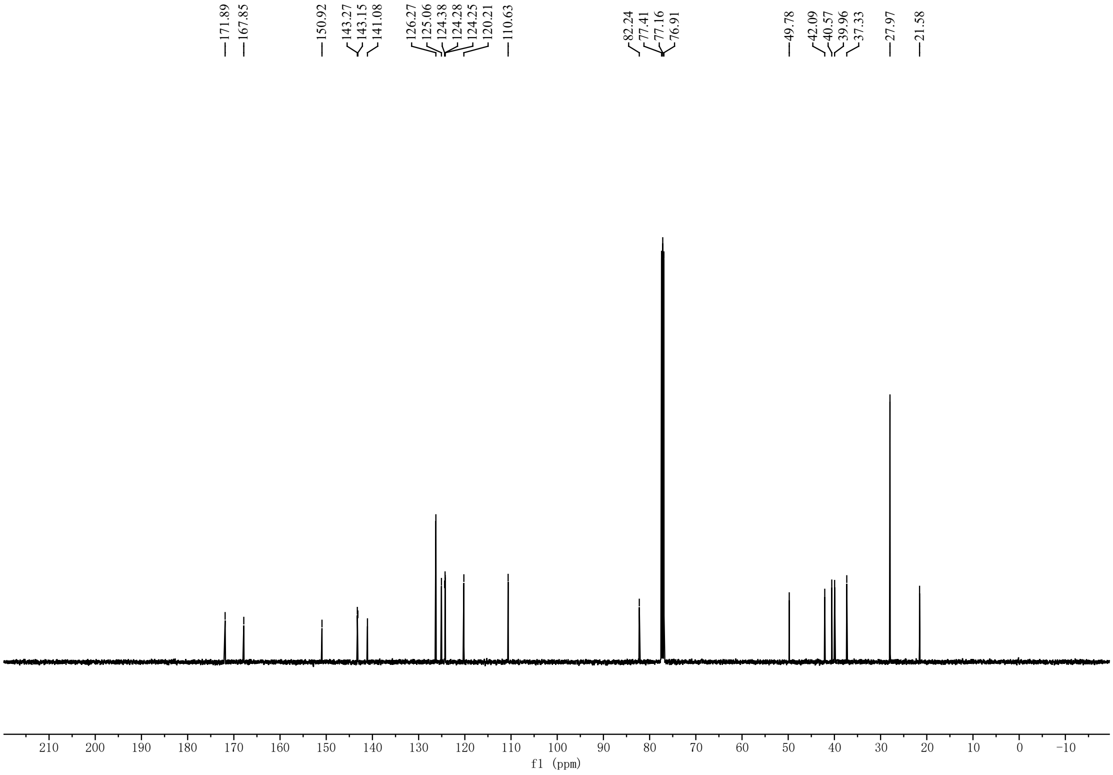

^{1}H NMR spectrum of **3al**

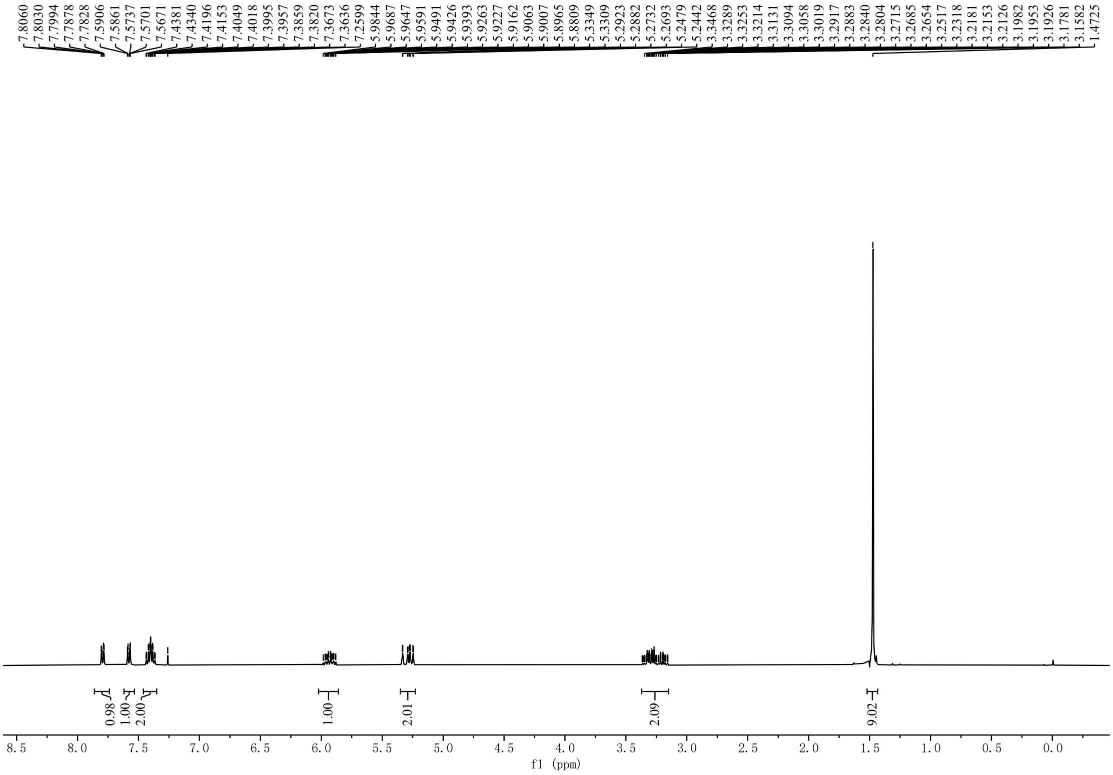

^{13}C NMR spectrum of **3al**

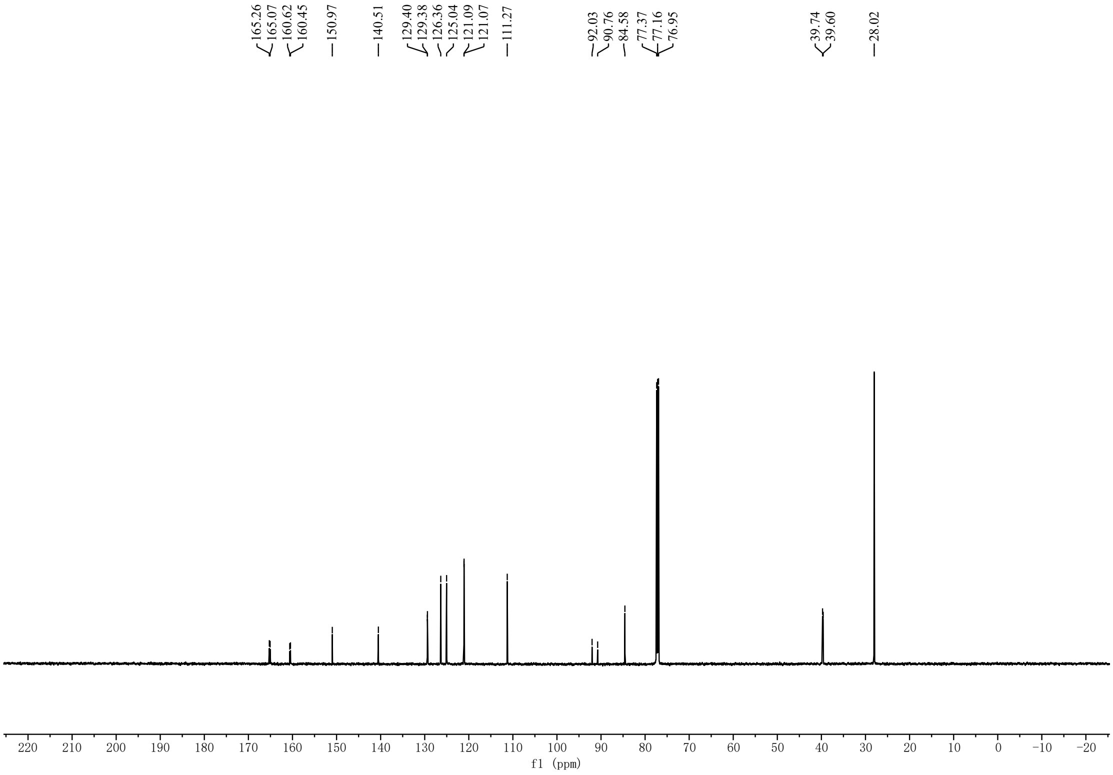

^{19}F NMR spectrum of **3al**

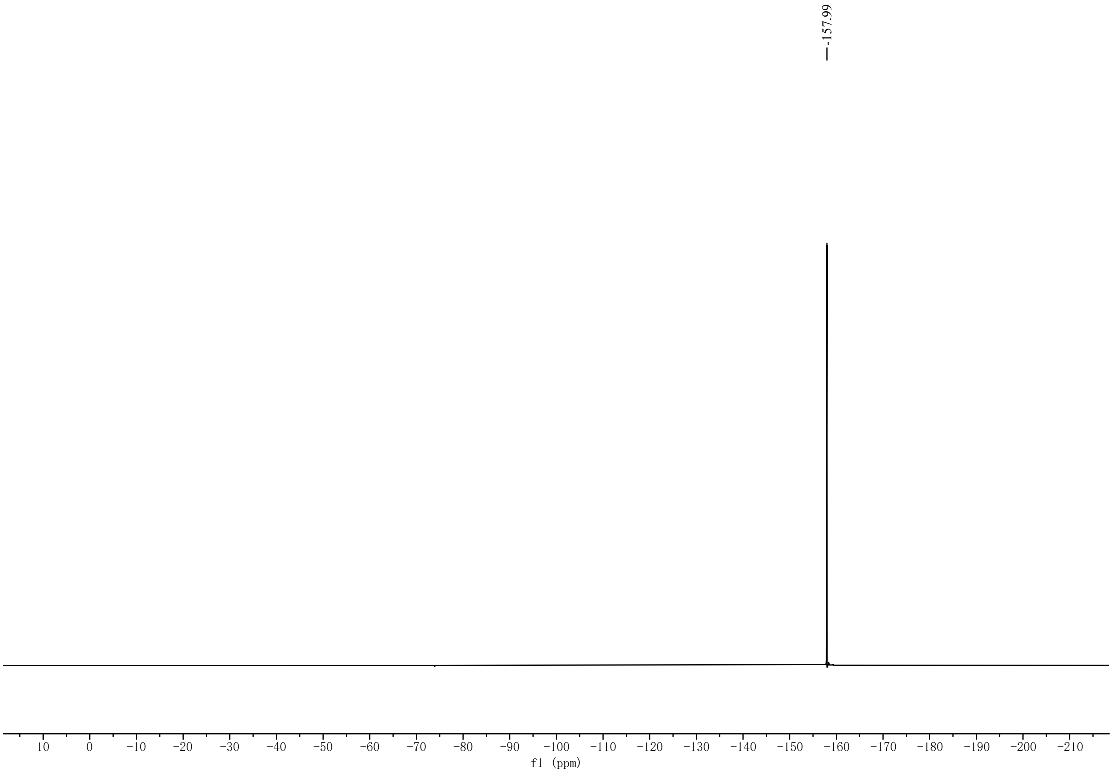

^{1}H NMR spectrum of **7**

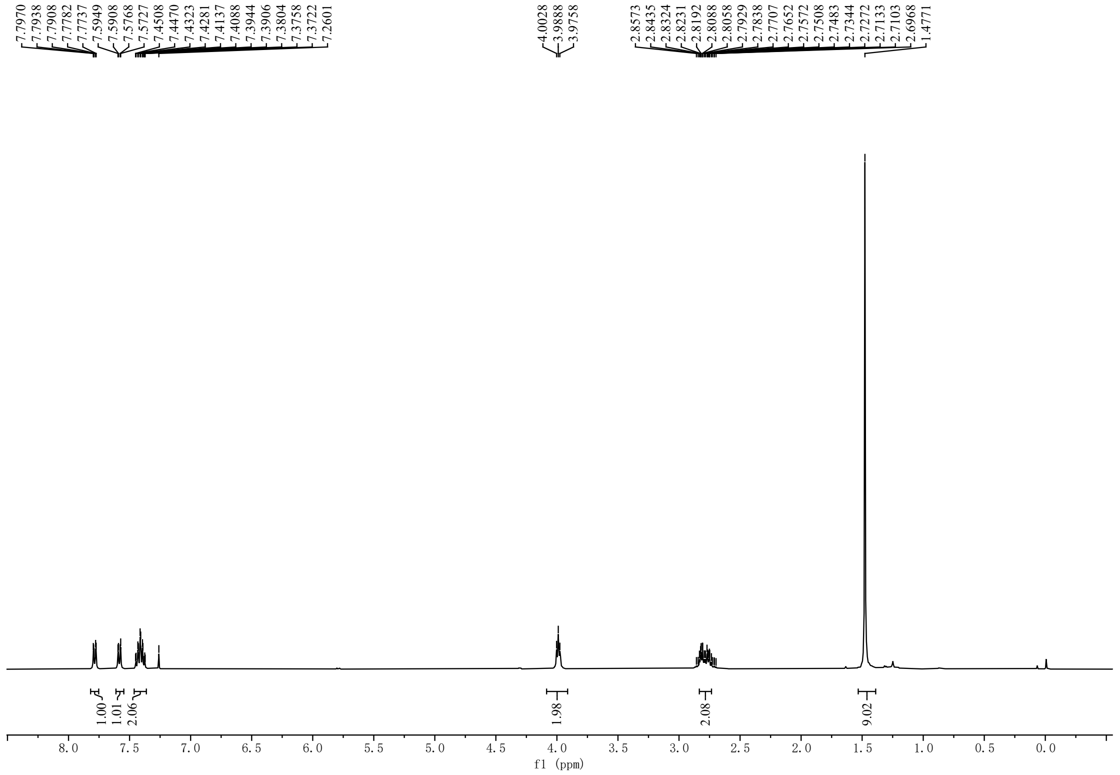

^{13}C NMR spectrum of **7**

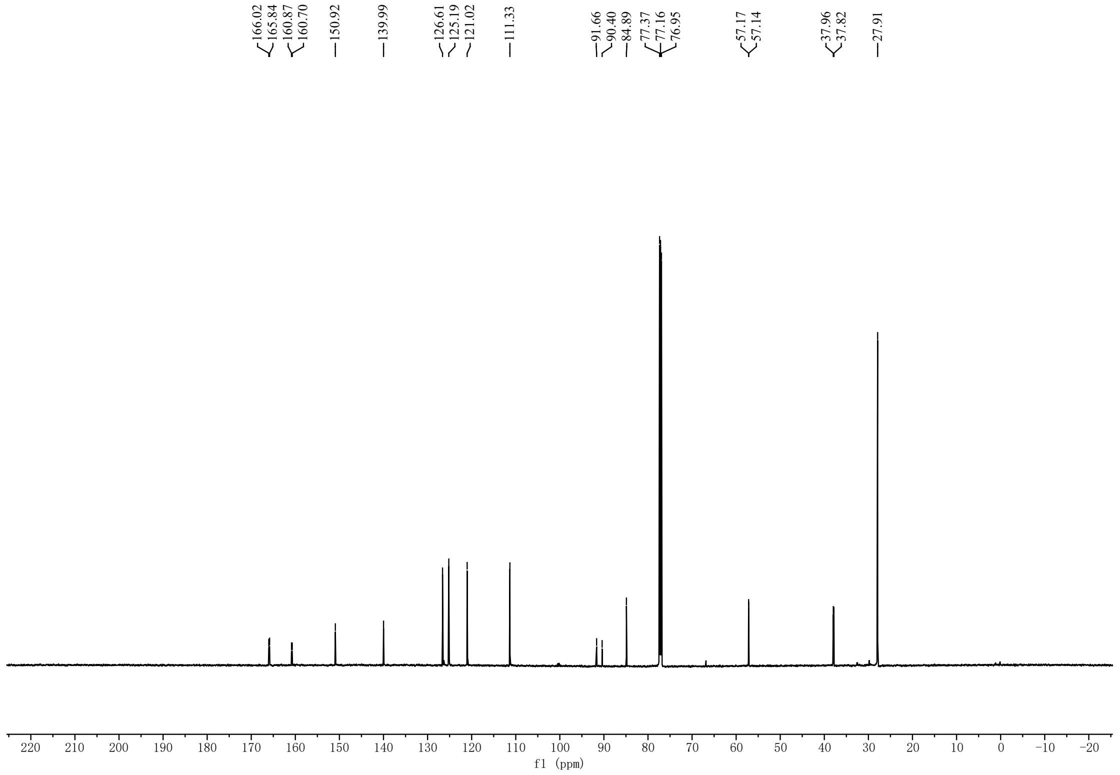

^{19}F NMR spectrum of **7**

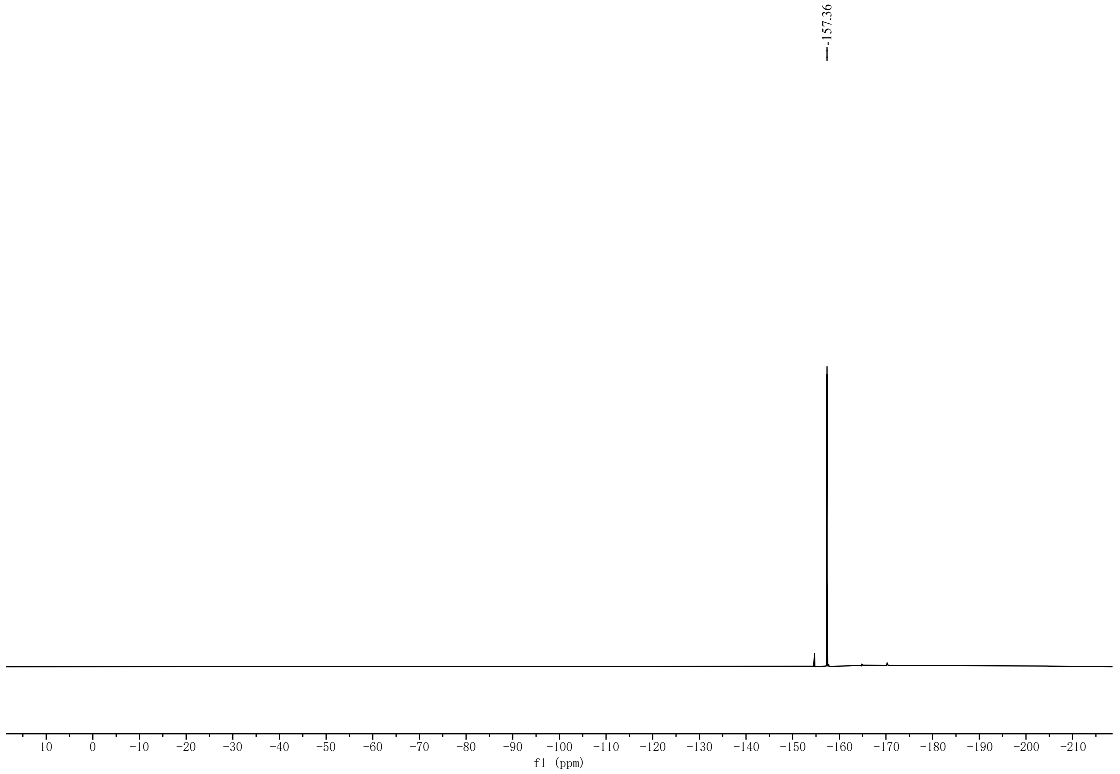

^{1}H NMR spectrum of **8**

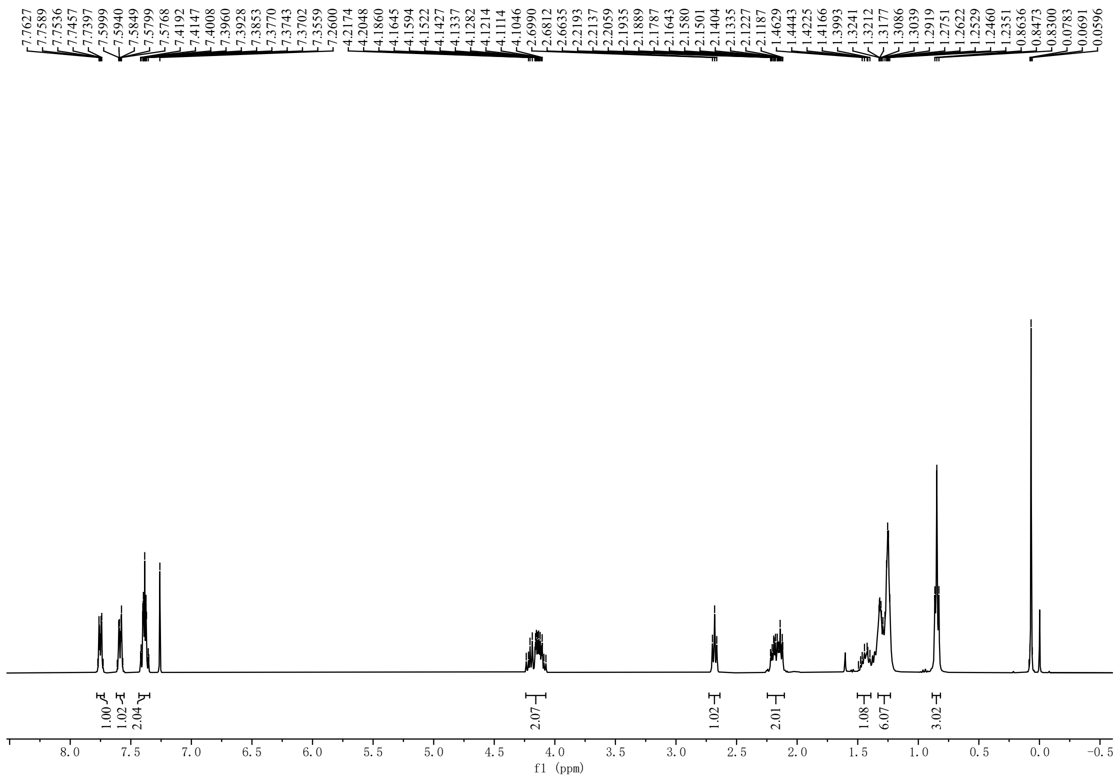

^{13}C NMR spectrum of **8**

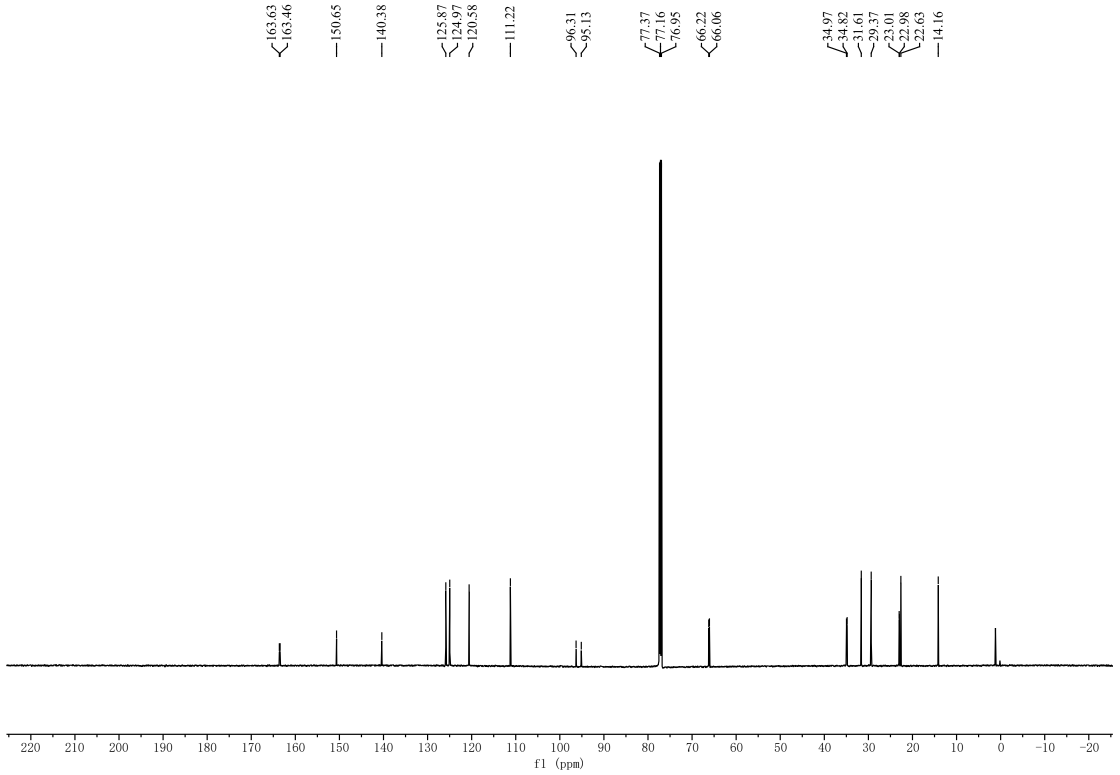

^{19}F NMR spectrum of **8**

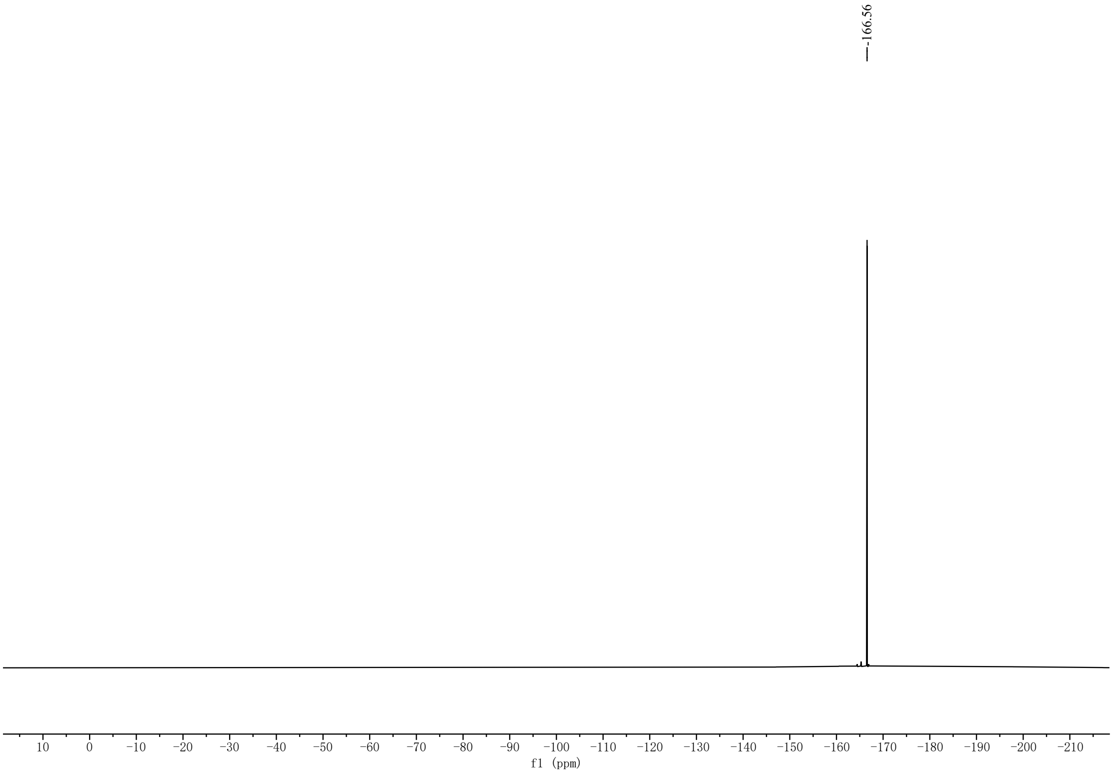

^{1}H NMR spectrum of **9**

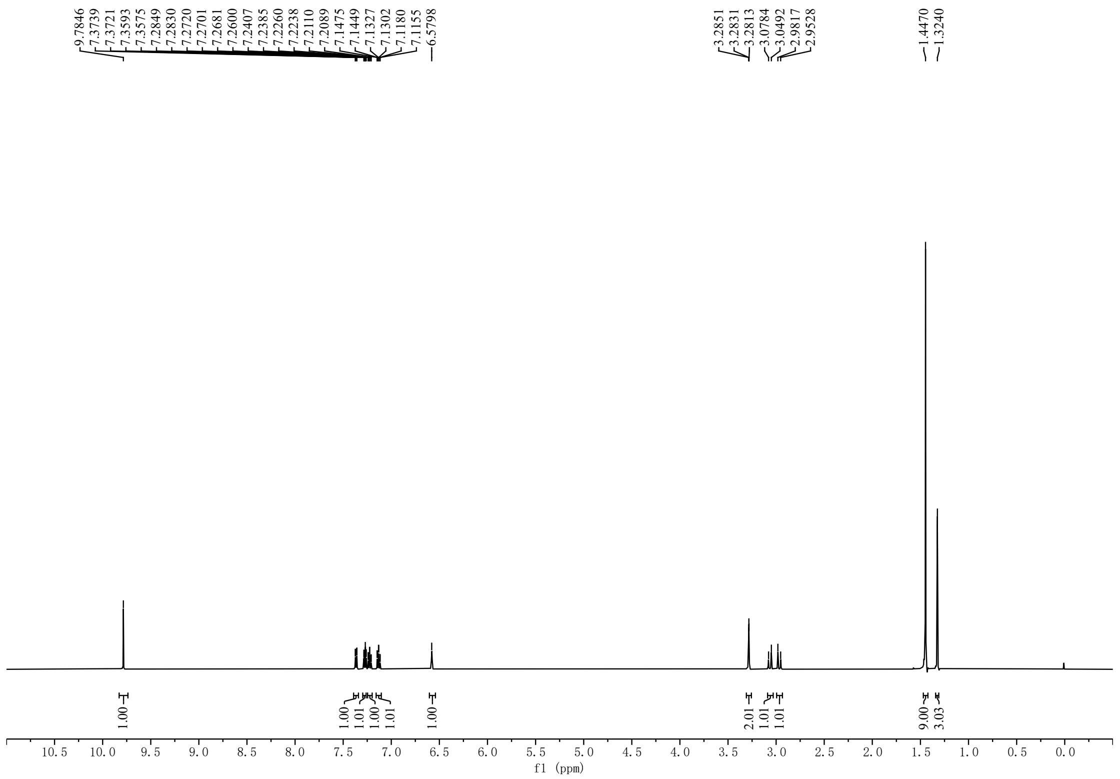

^{13}C NMR spectrum of **9**

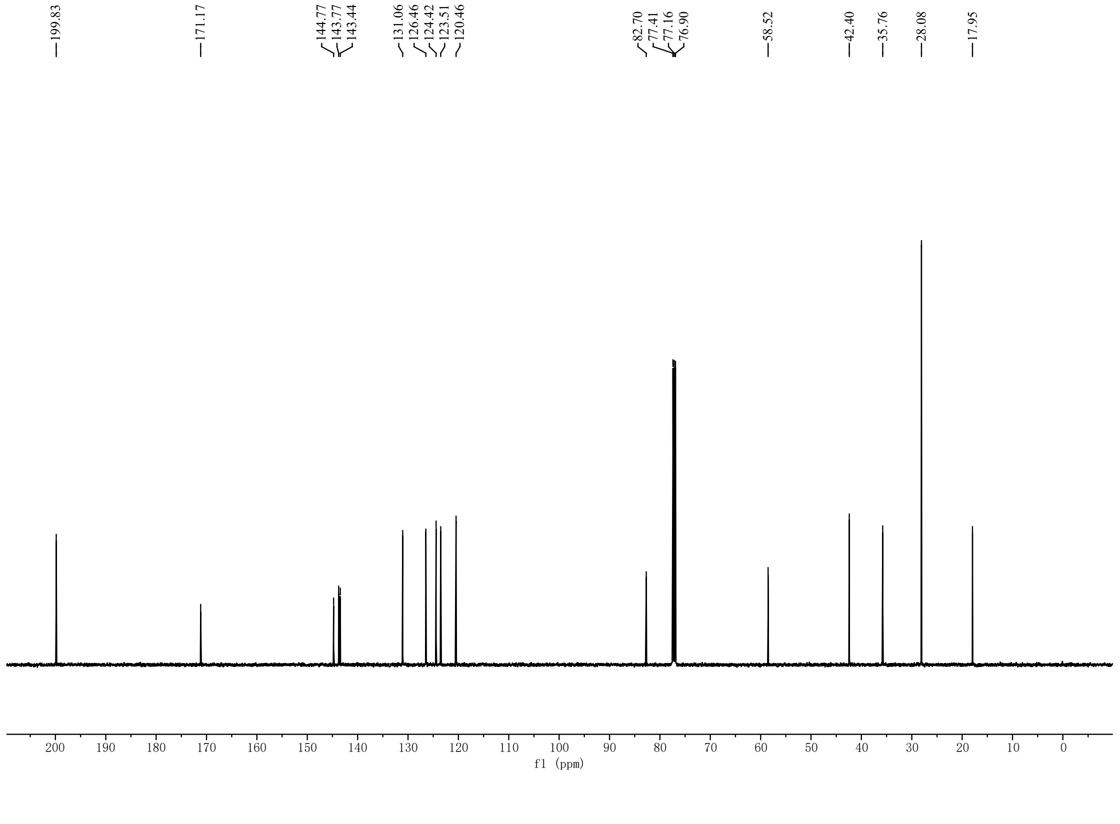

^{1}H NMR spectrum of **10**

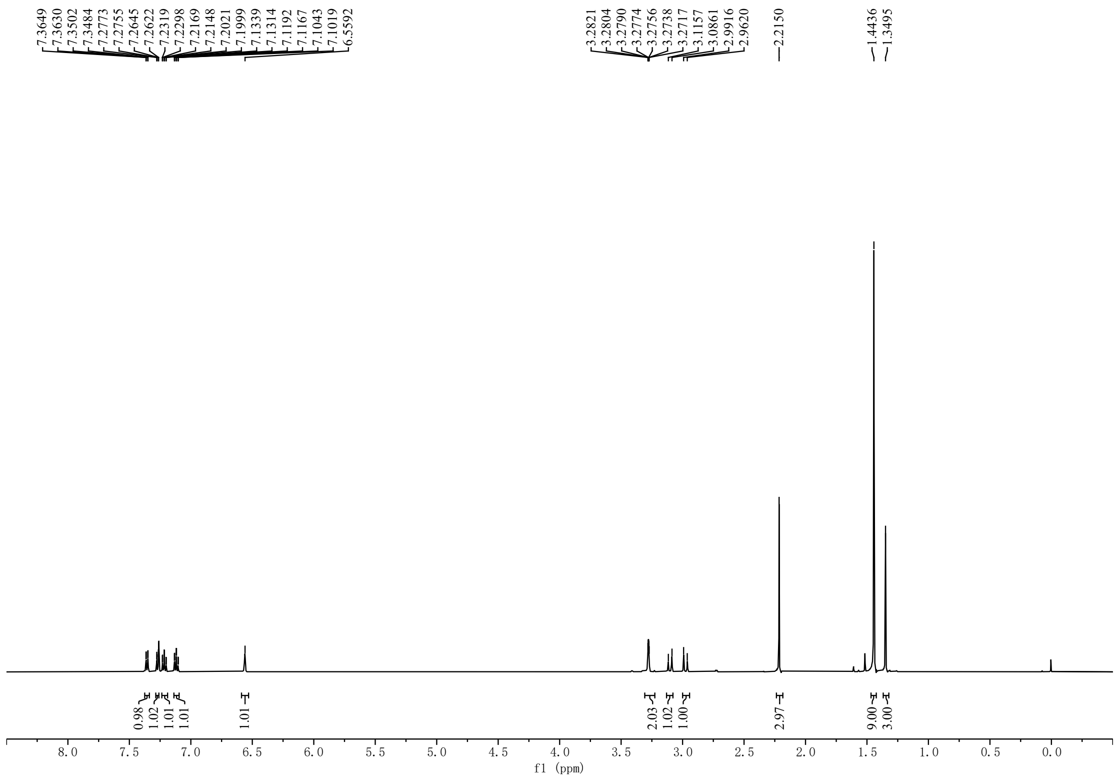

^{13}C NMR spectrum of **10**

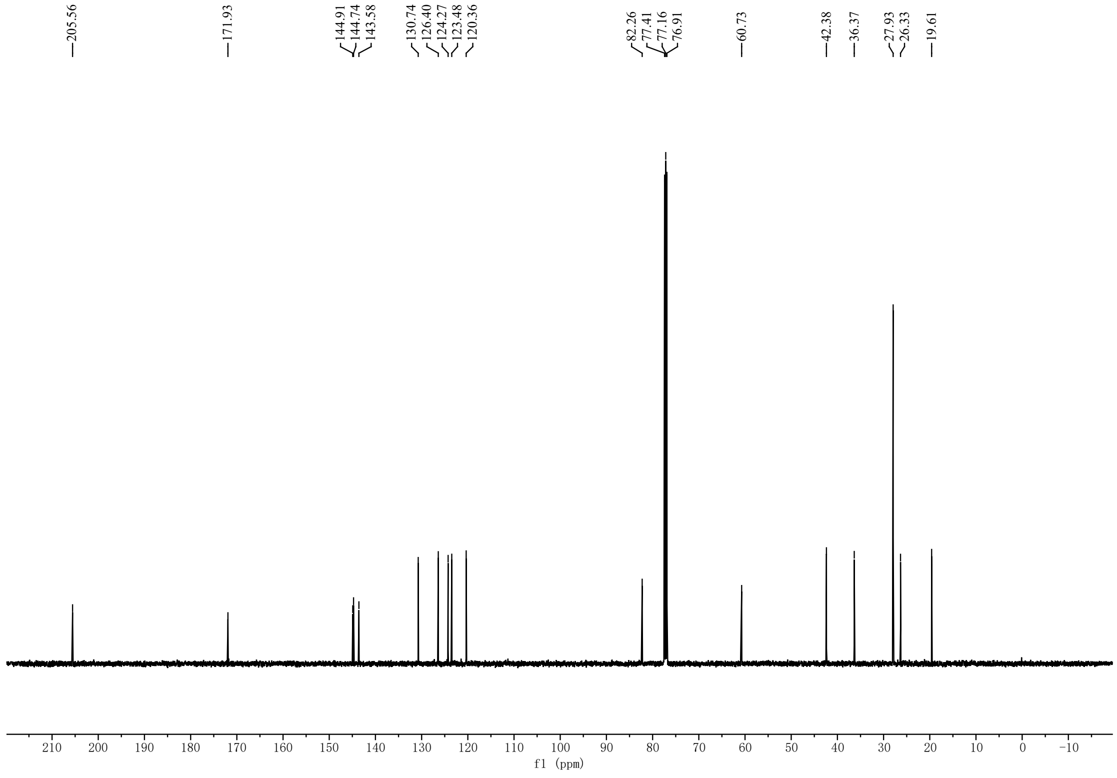

^{1}H NMR spectrum of **11**

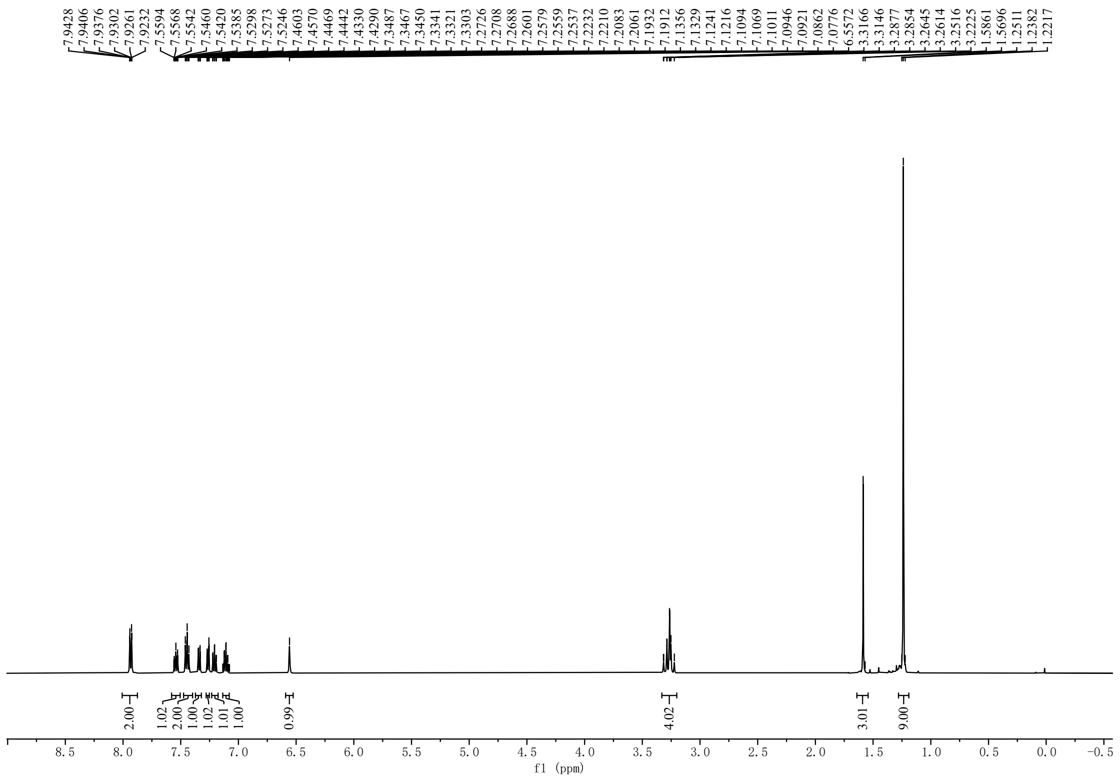

^{13}C NMR spectrum of **11**

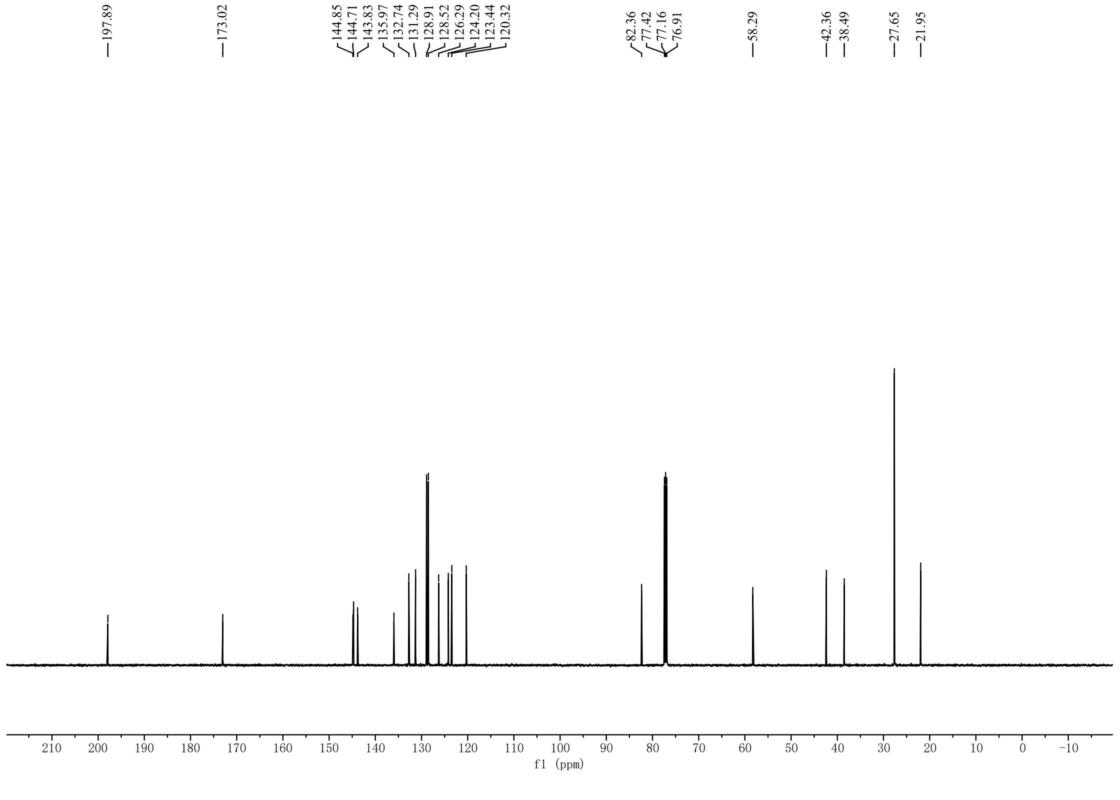

^{1}H NMR spectrum of **12**

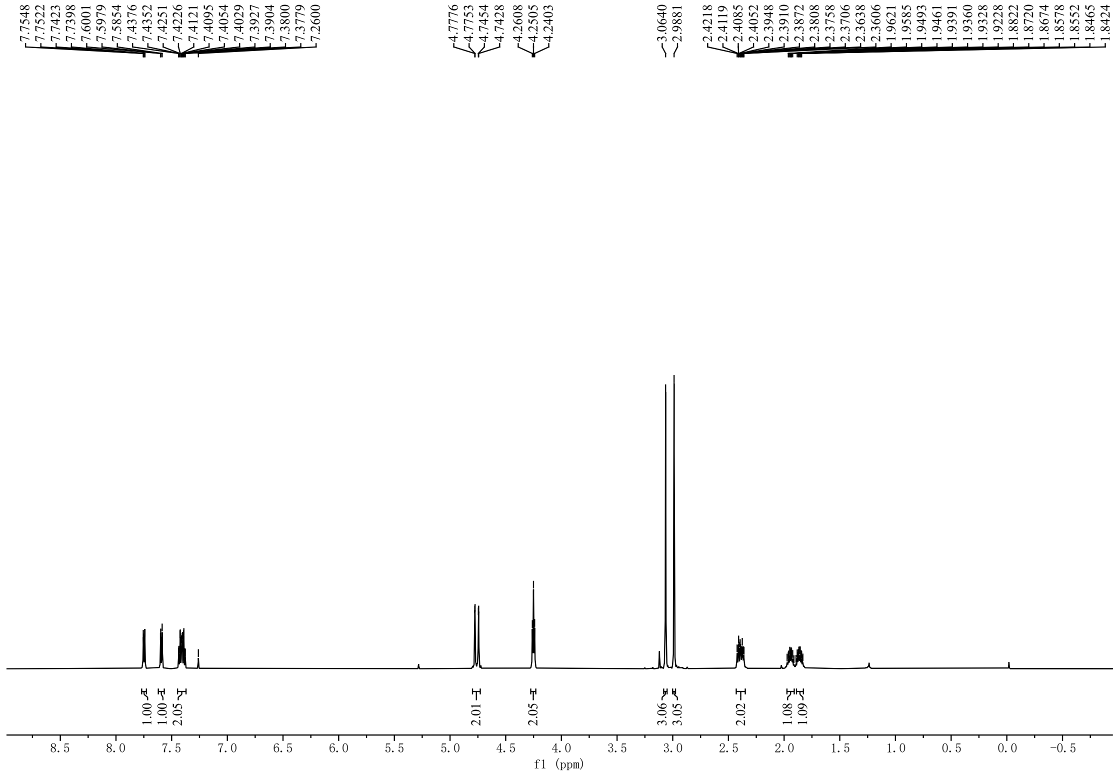

^{13}C NMR spectrum of **12**

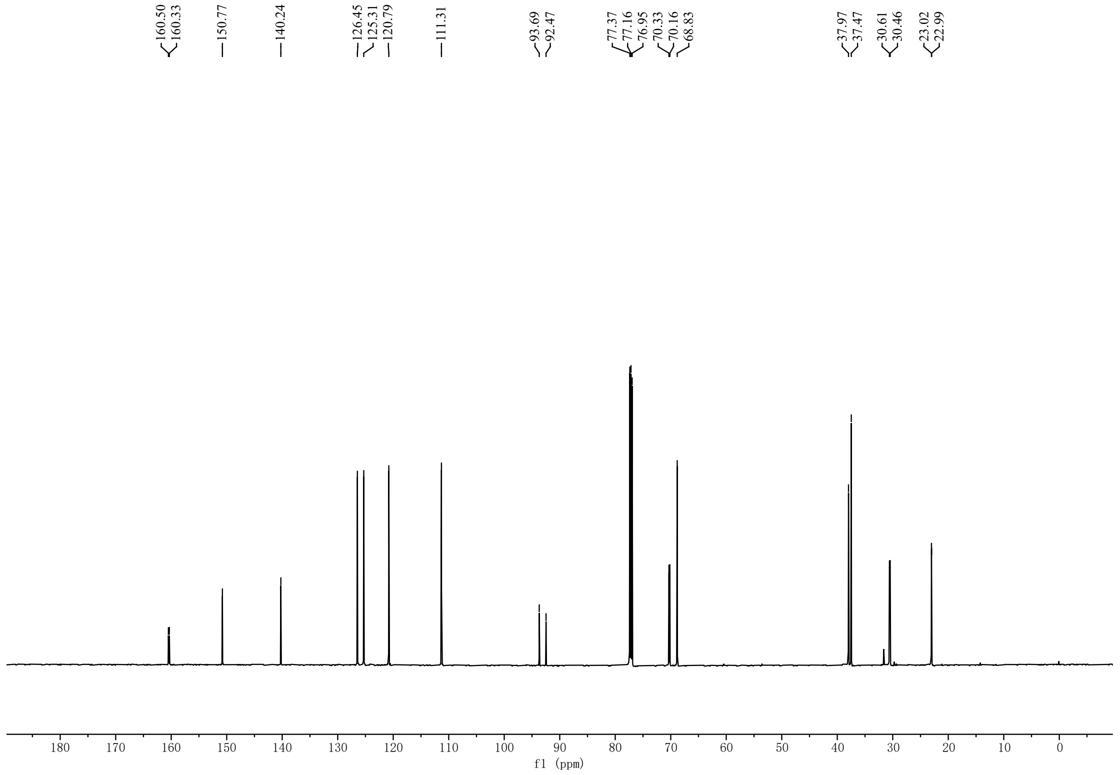

^{19}F NMR spectrum of **12**

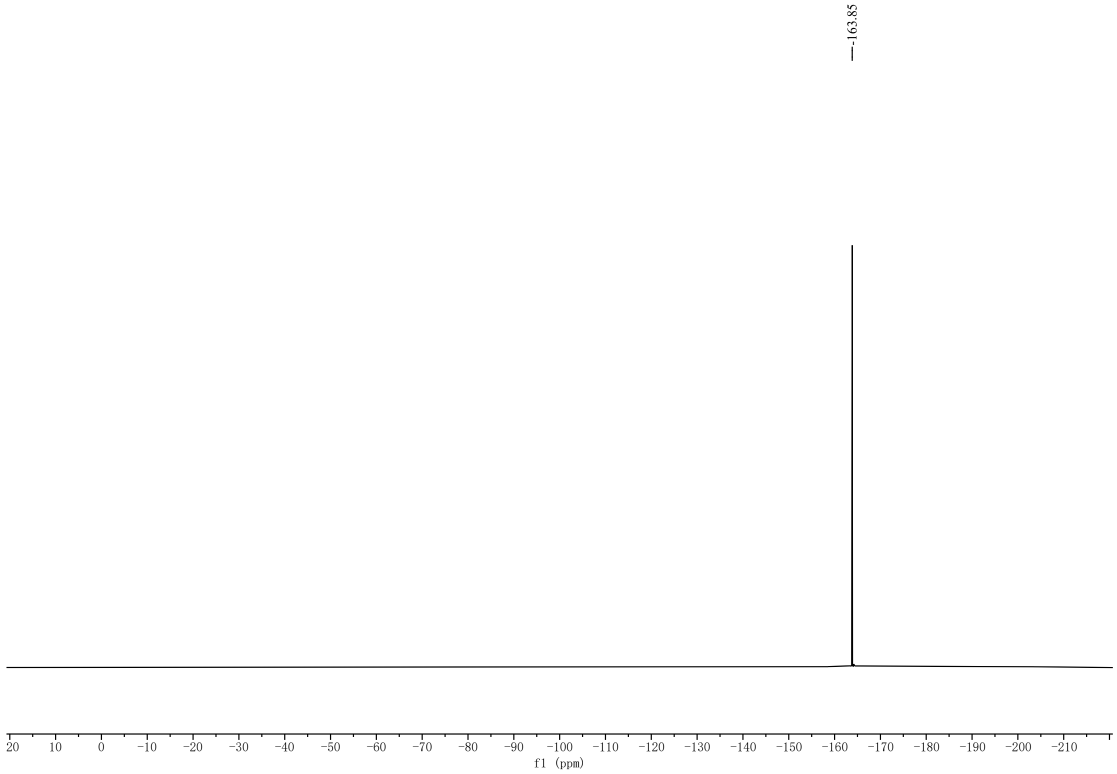

^{1}H NMR spectrum of **13**

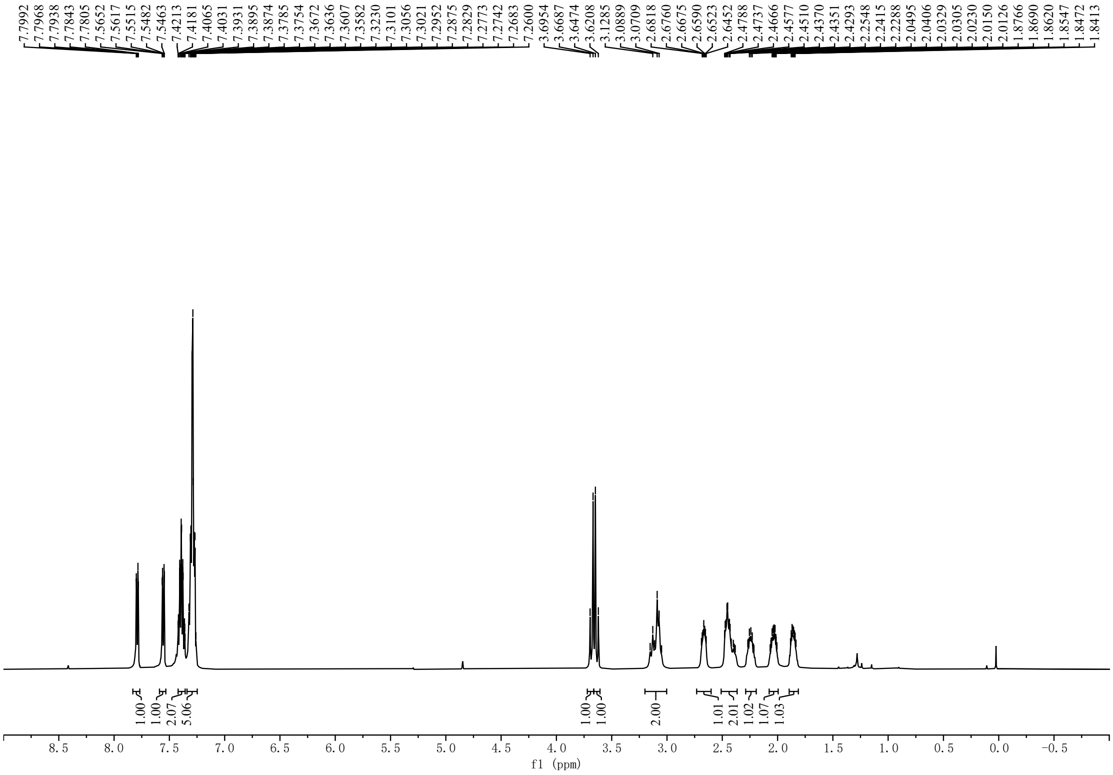

^{13}C NMR spectrum of **13**

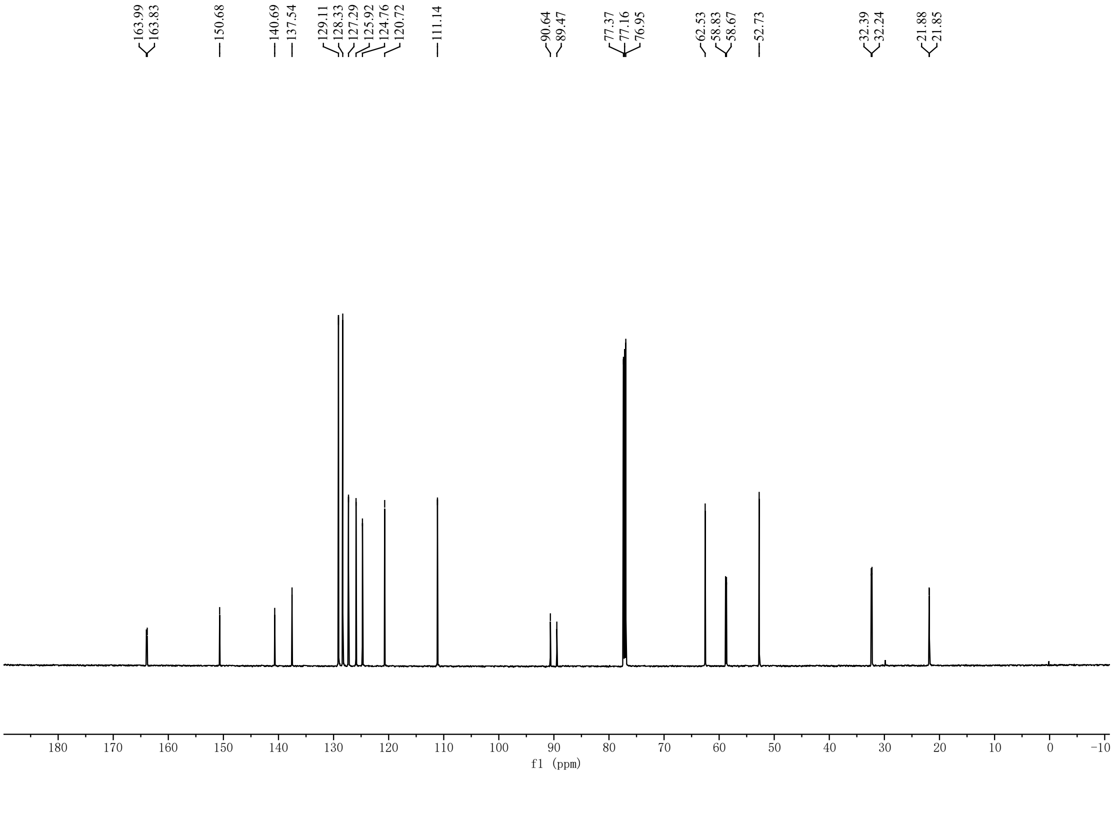

^{1}H NMR spectrum of **14**

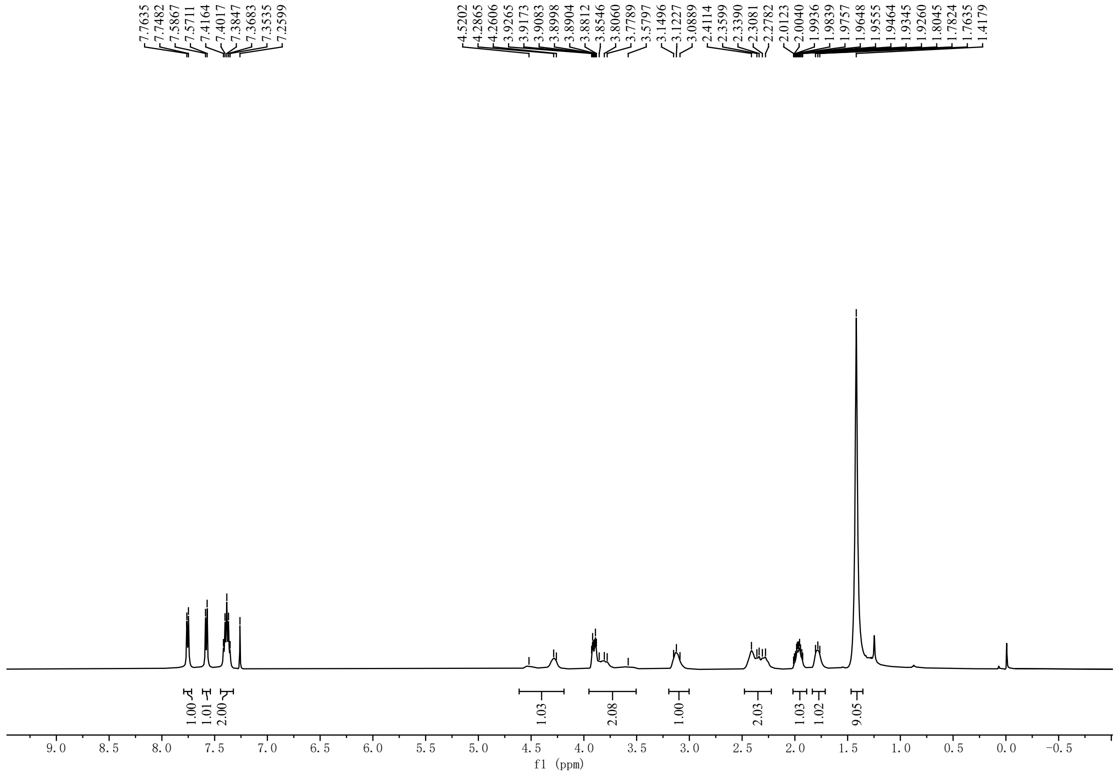

^{13}C NMR spectrum of **14**

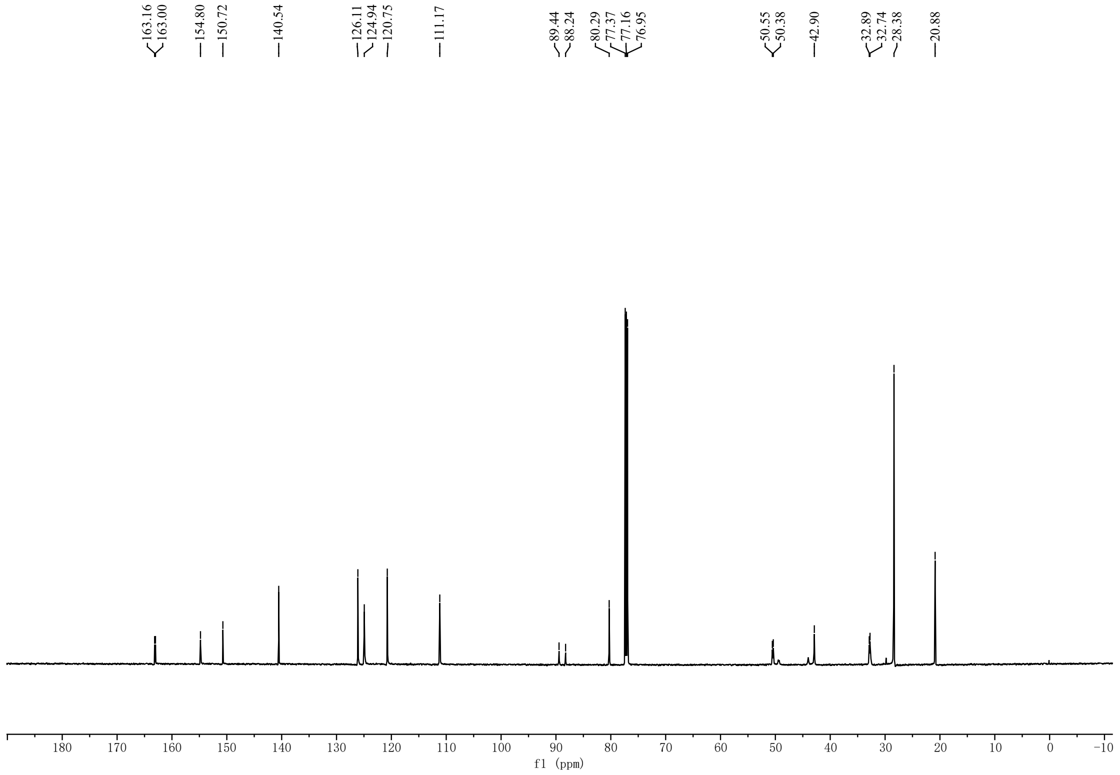

^{1}H NMR spectrum of **15**

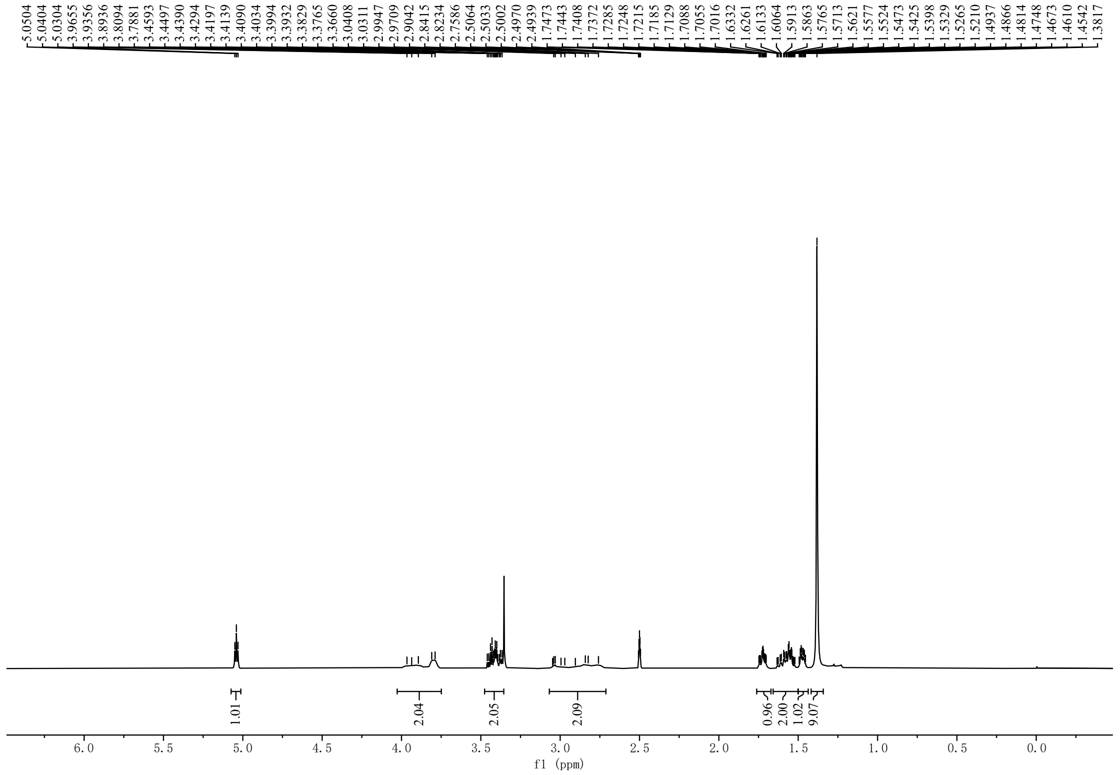

^{13}C NMR spectrum of **15**

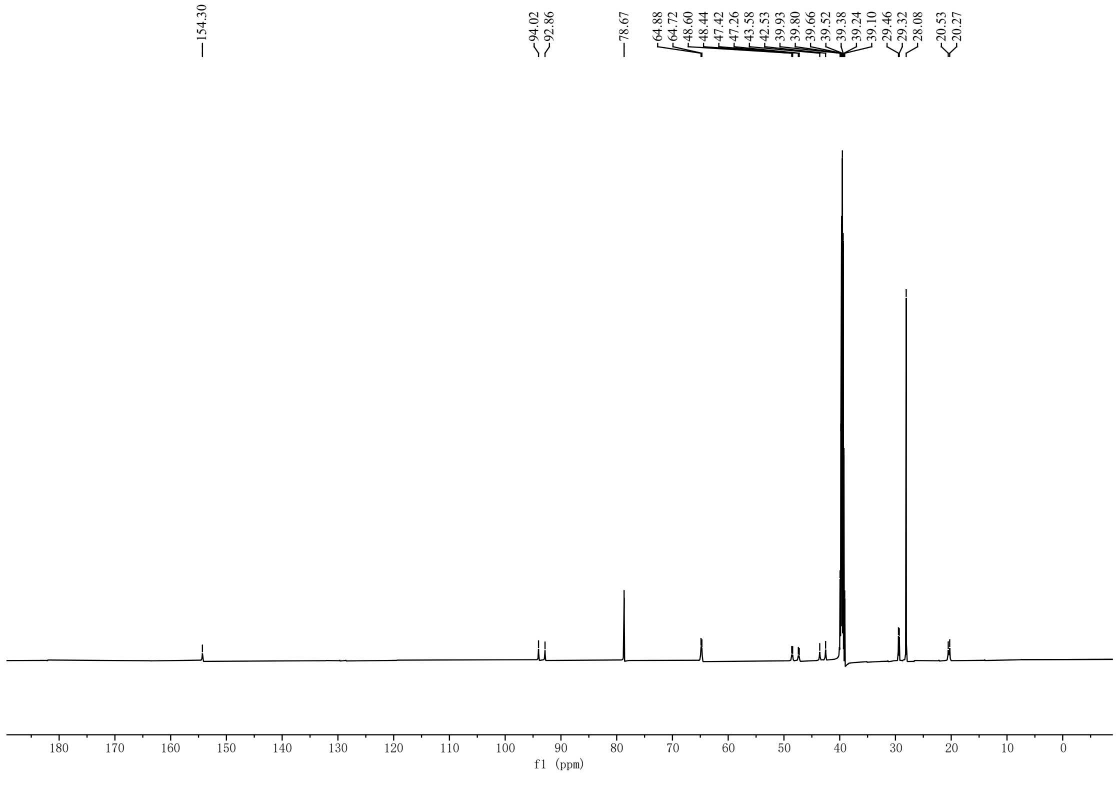

^{19}F NMR spectrum of **15**

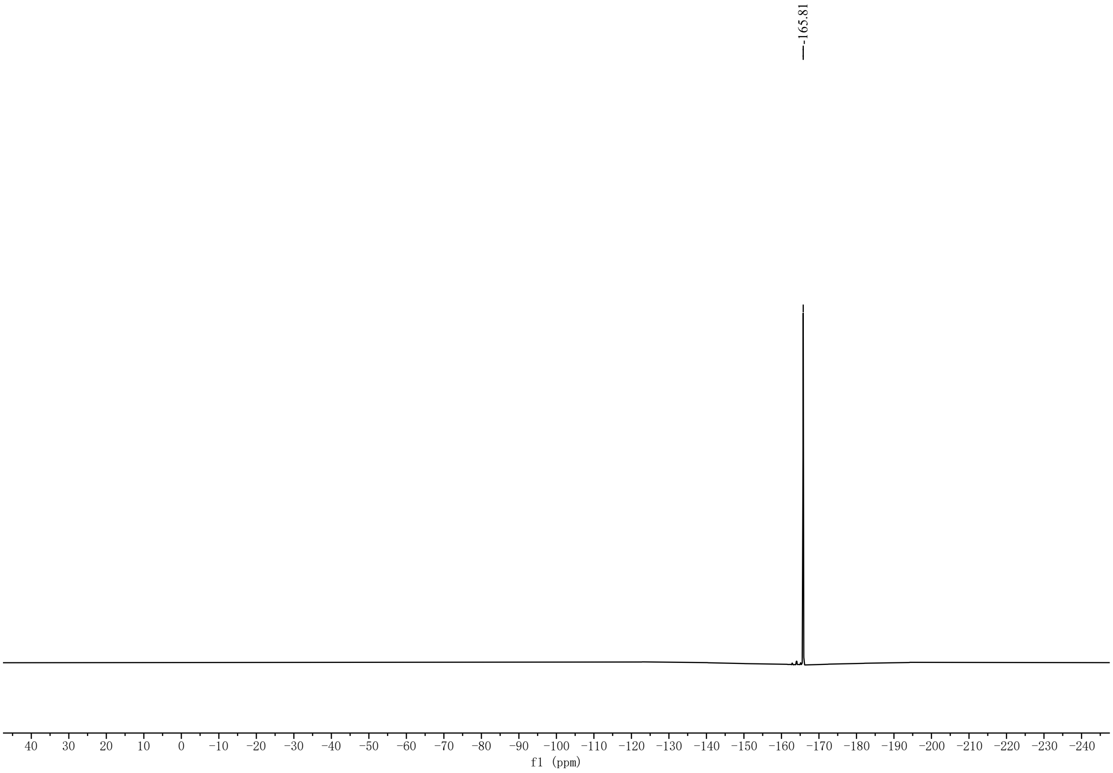

^{1}H NMR spectrum of **16**

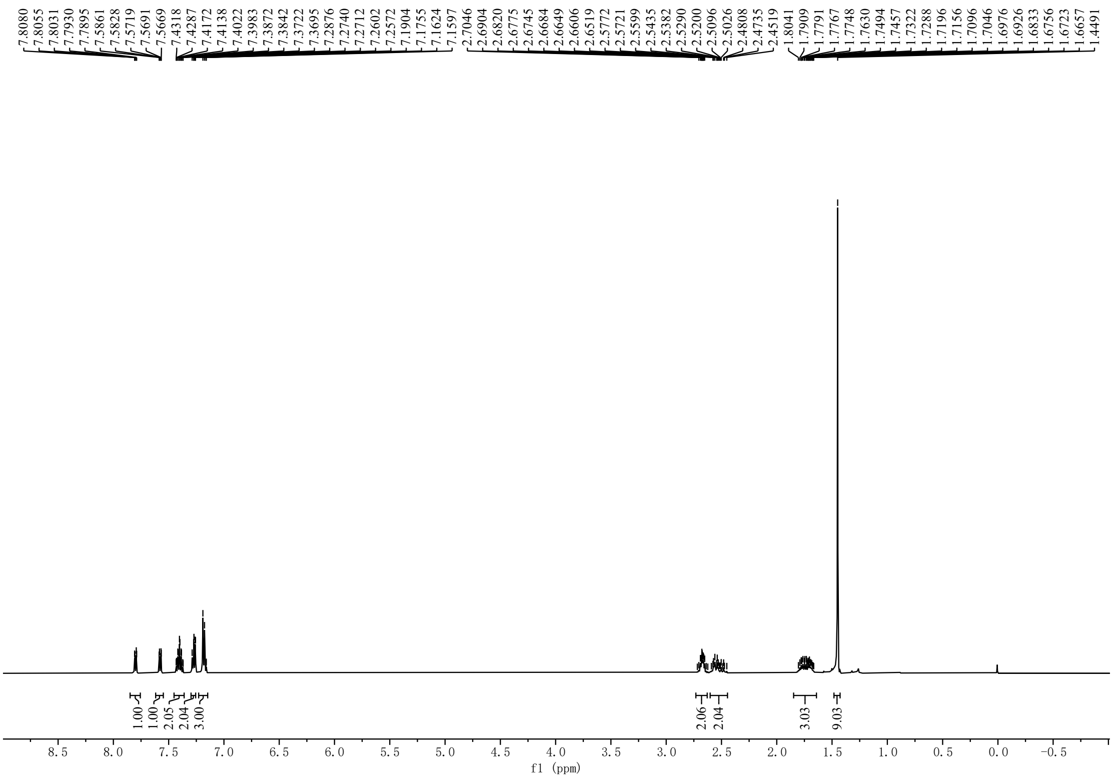

^{13}C NMR spectrum of **16**

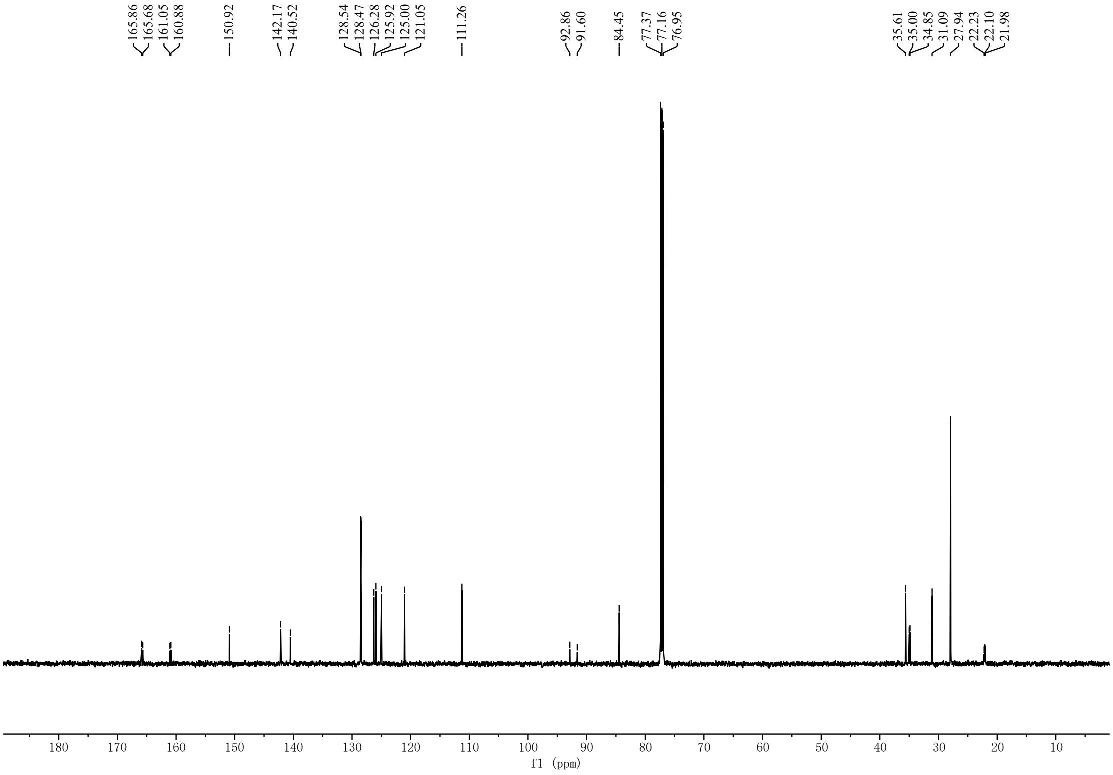

^{19}**F NMR** spectrum of **16**

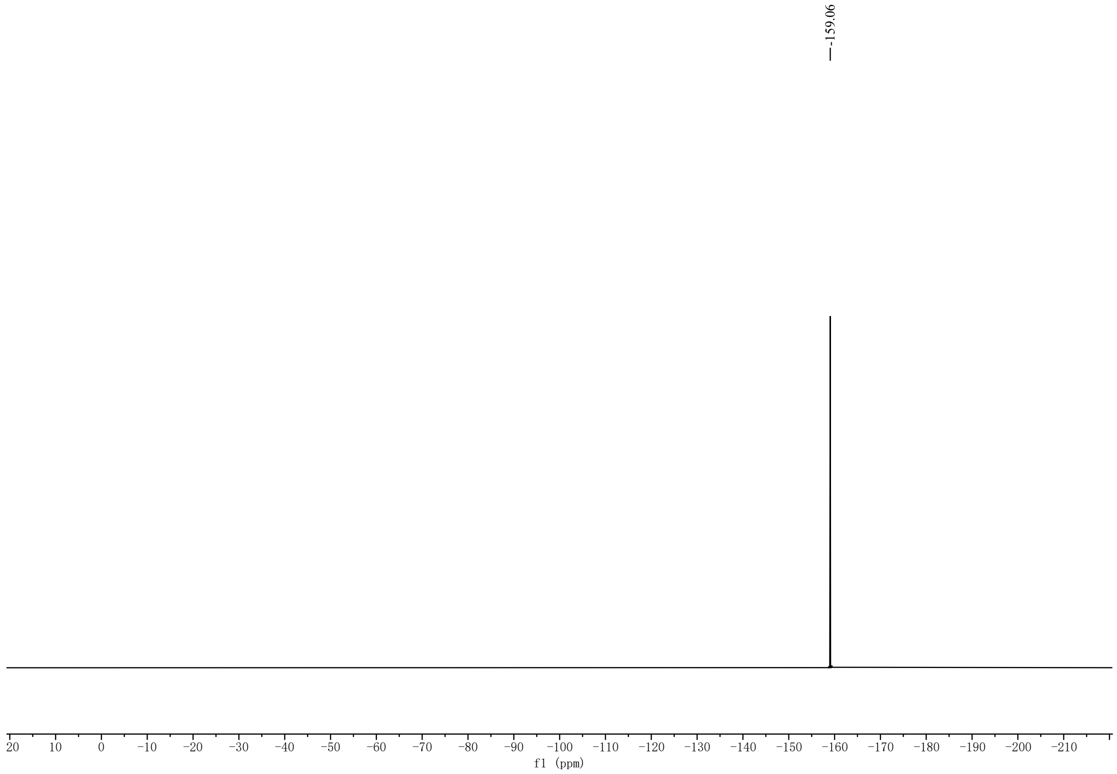

^{1}H NMR spectrum of **18**

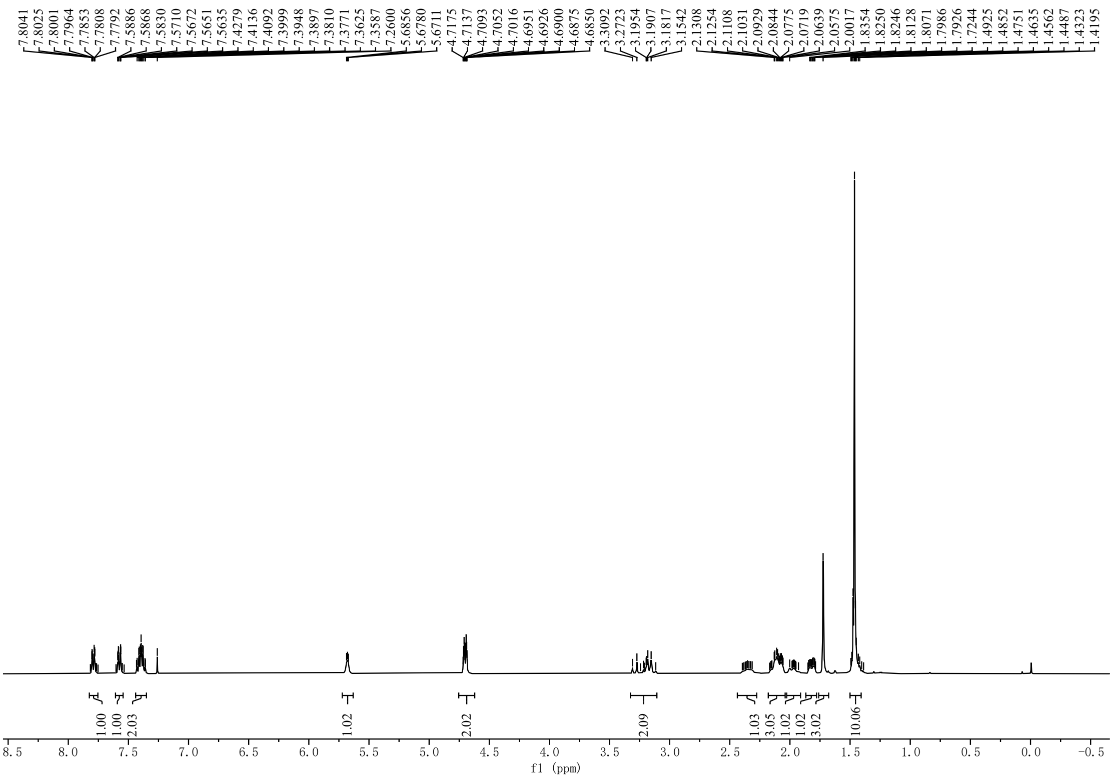

^{13}C NMR spectrum of **18**

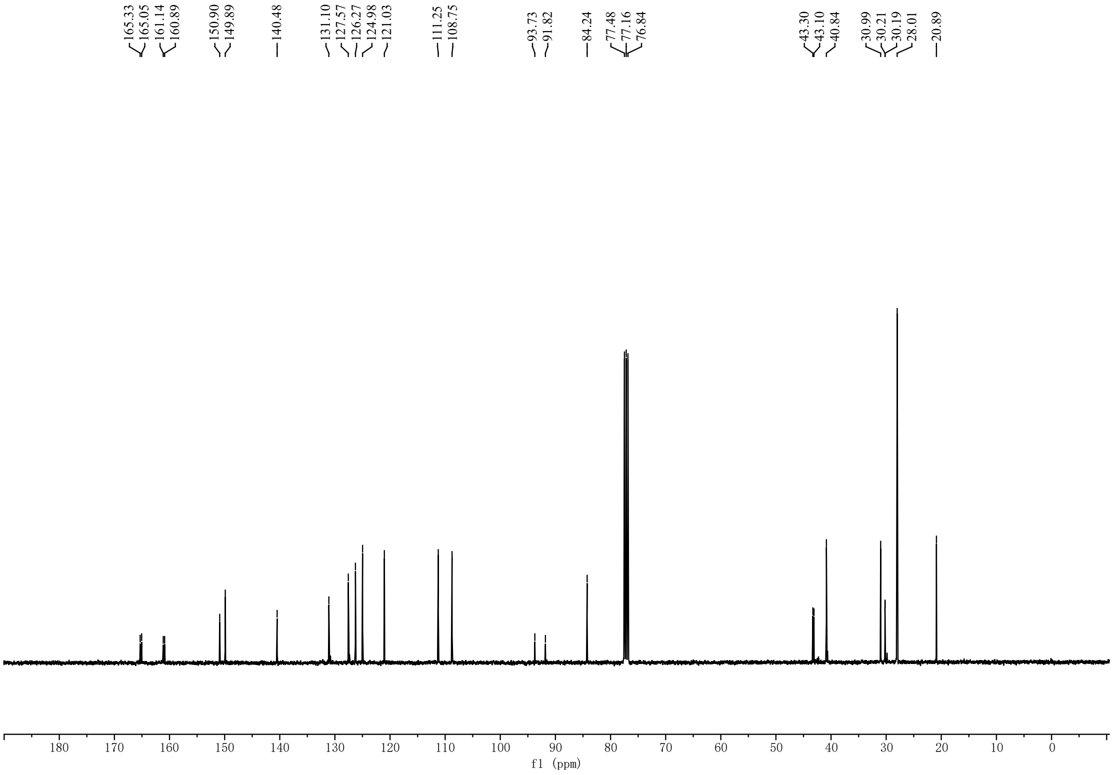

^{19}F NMR spectrum of **18**

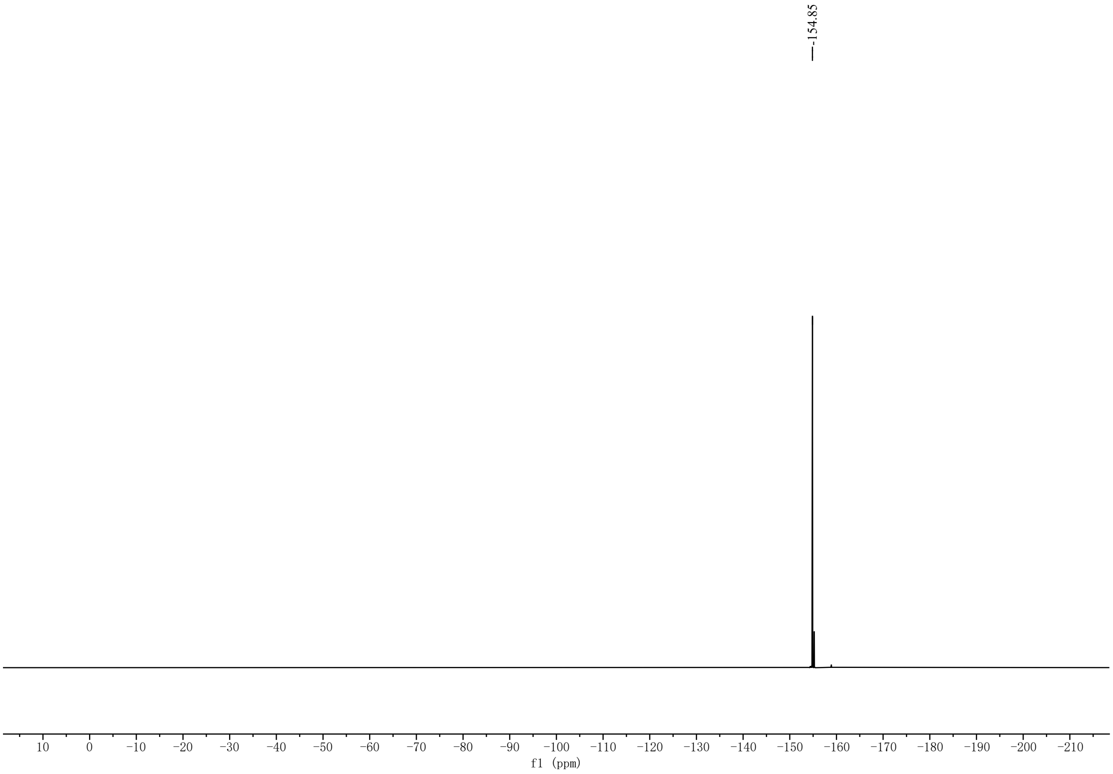

¹H NMR spectrum of **20** (The product was isolated as a 10:1 mixture of E/Z isomers)

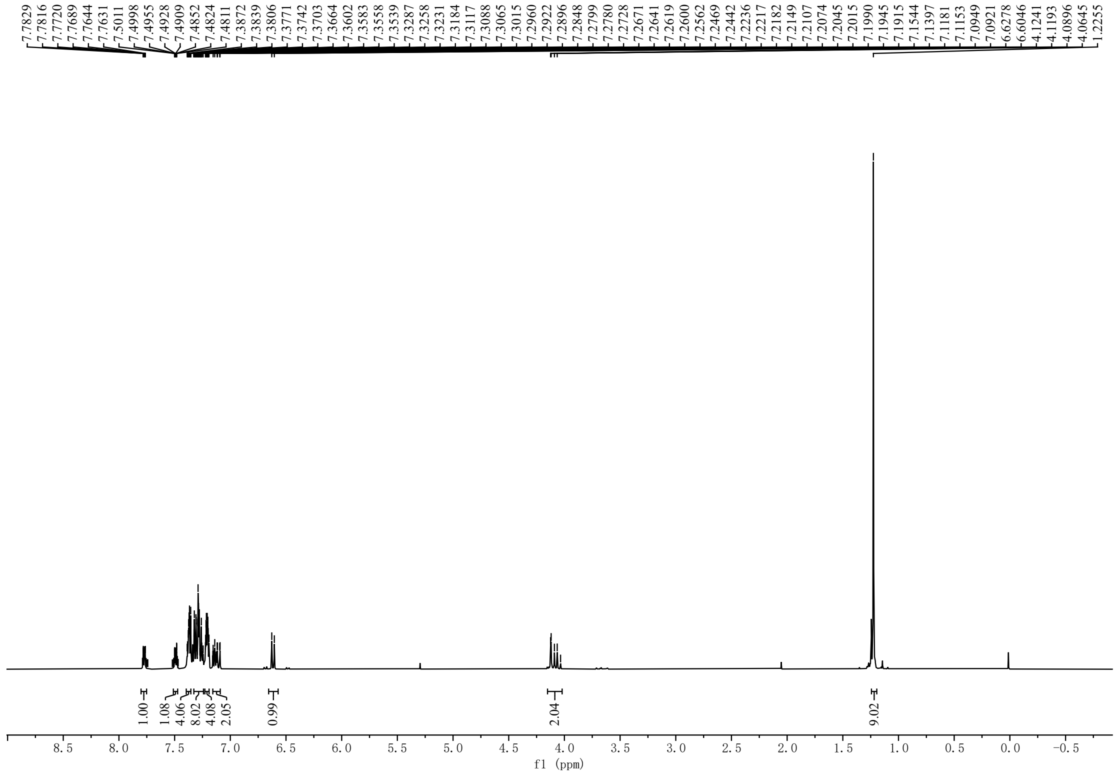

¹³C NMR spectrum of **20**

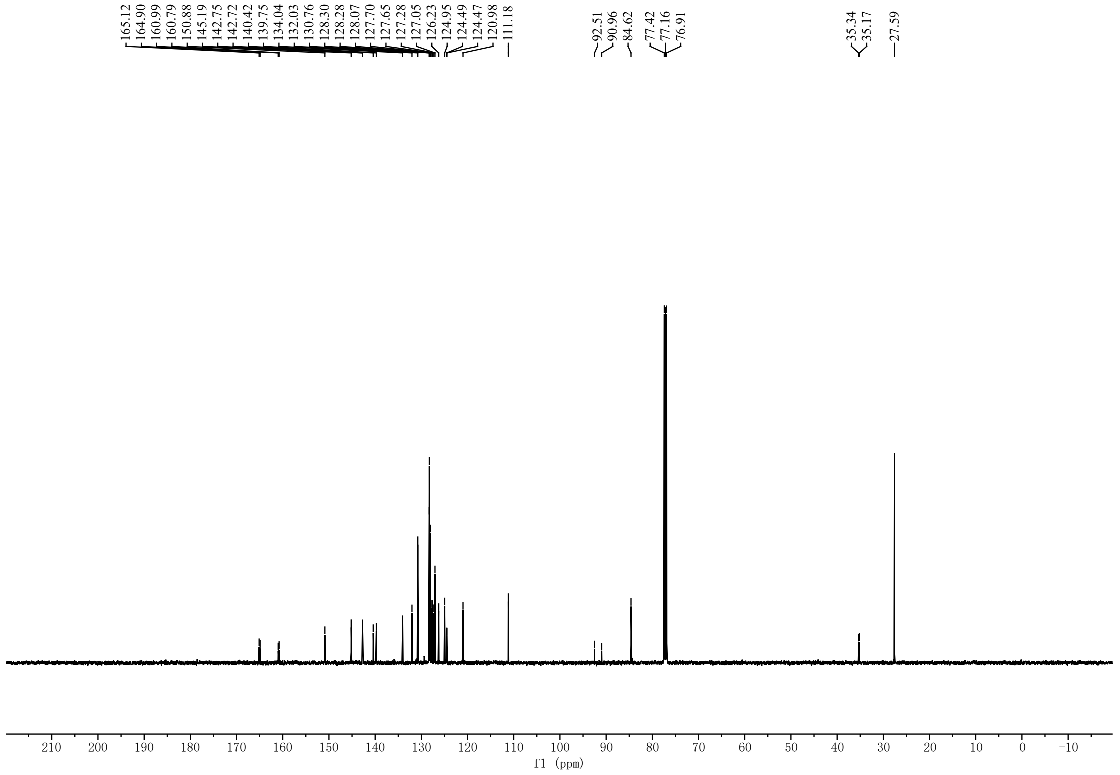

^{19}F NMR spectrum of **20**

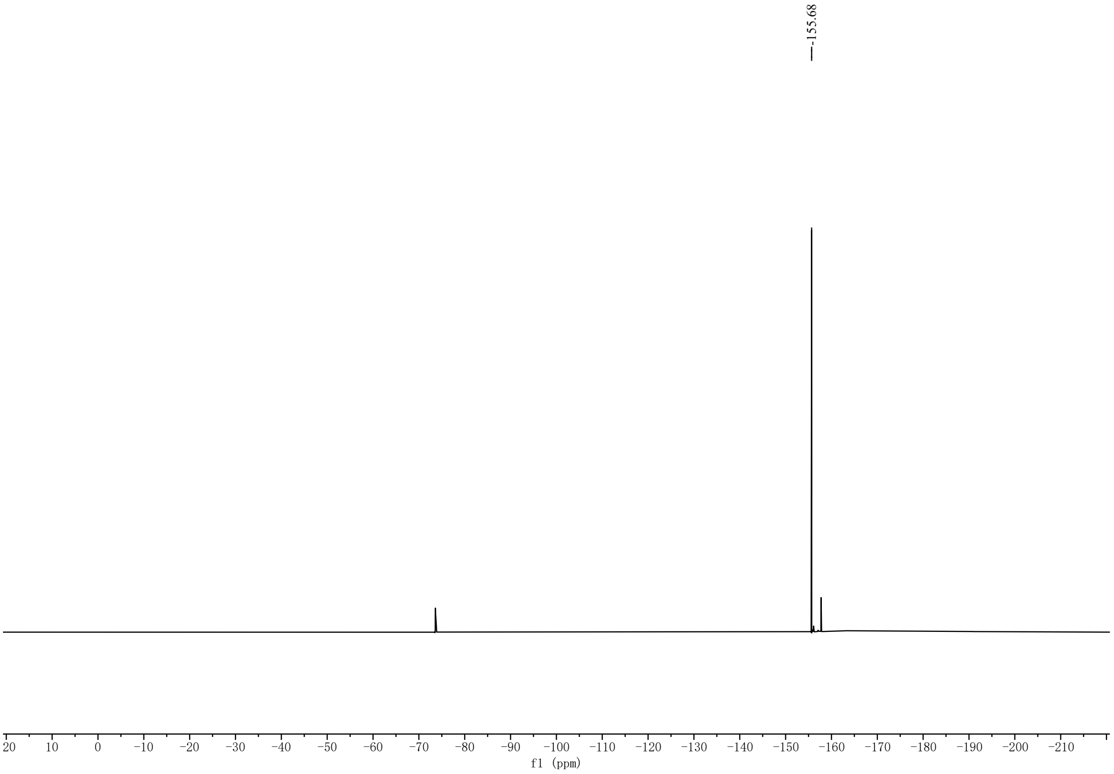

COSY spectrum of **20**

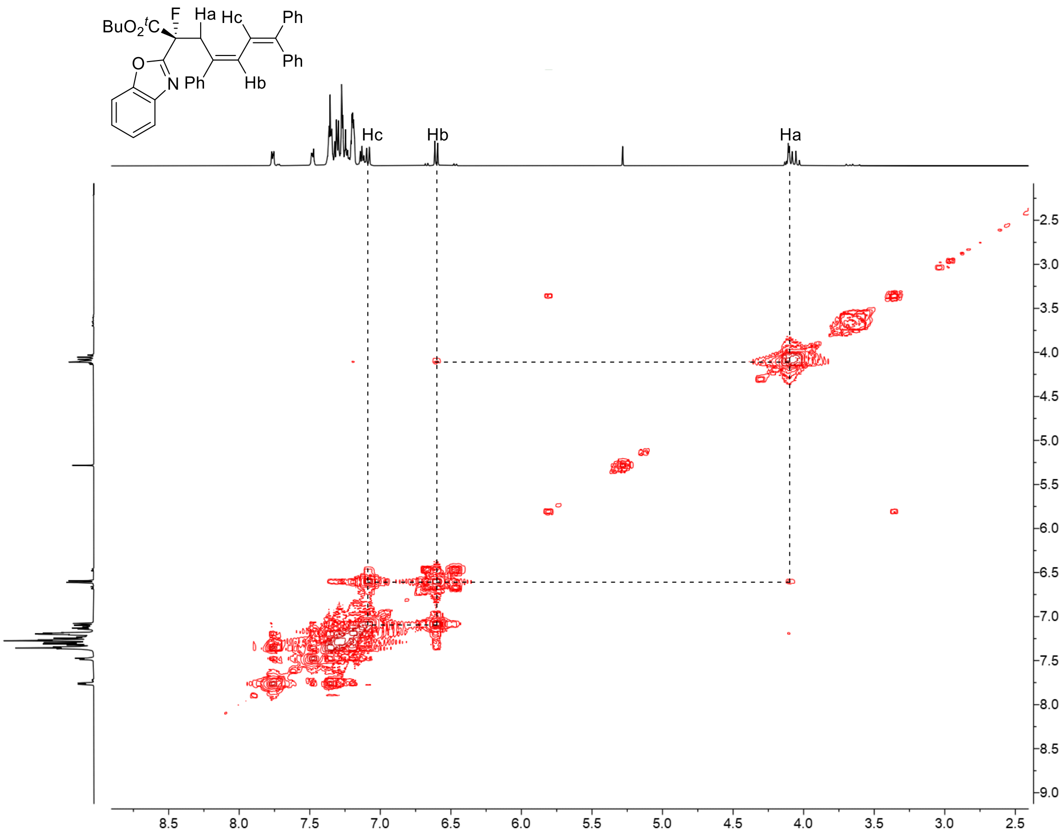

NOESY spectrum of **20**

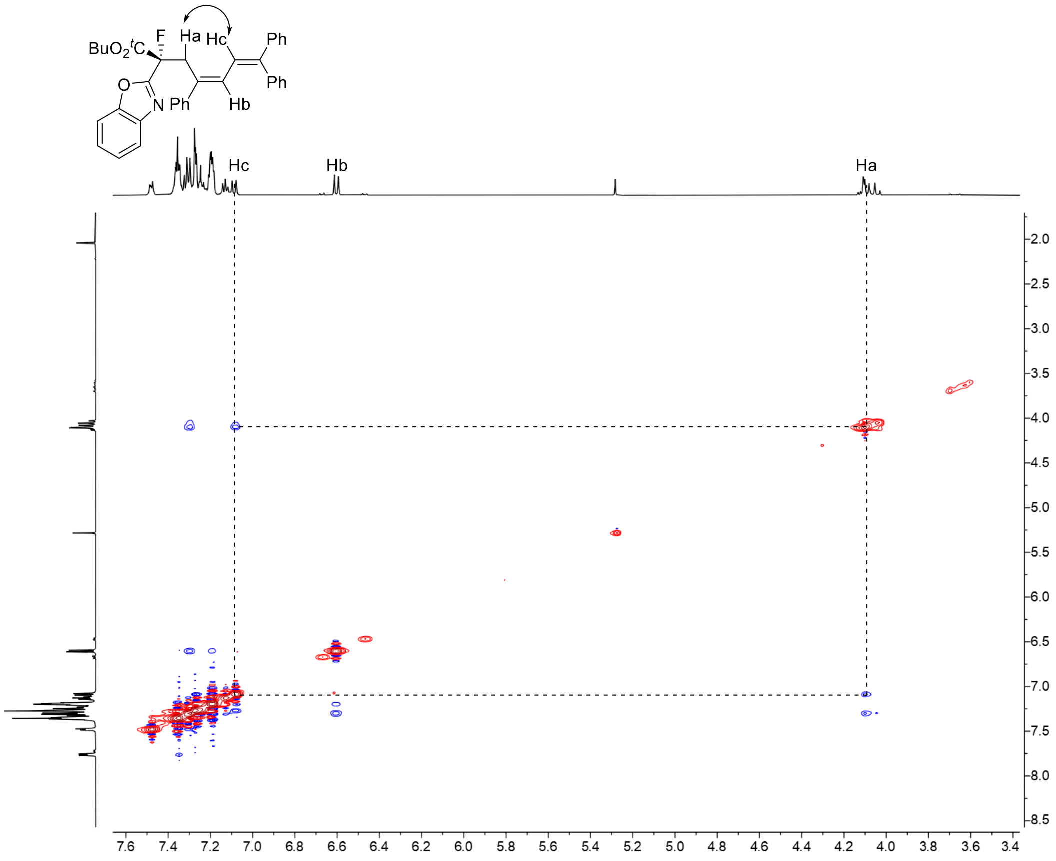

12. HPLC traces

Rac-**3a** (The product was isolated as a 6:1 mixture of E/Z isomers)

SAMPLE INFORMATION

Sample Name:	XT-8-38-1-RAC-1%-IG	Acquired By:	System
Sample Type:	Unknown	Sample Set Name:	20240913
Vial:	18	Acq. Method Set:	1% quanbo
Injection #:	1	Processing Method	XT 8 38 1 RAC
Injection Volume:	30.00 ul	Channel Name:	254.0nm
Run Time:	11.0 Minutes	Proc. Chnl. Descr.:	2998 PDA 254.0 nm (2998
Date Acquired:	9/13/2024 22:00:59 CST		
Date Processed:	9/13/2024 22:58:46 CST		

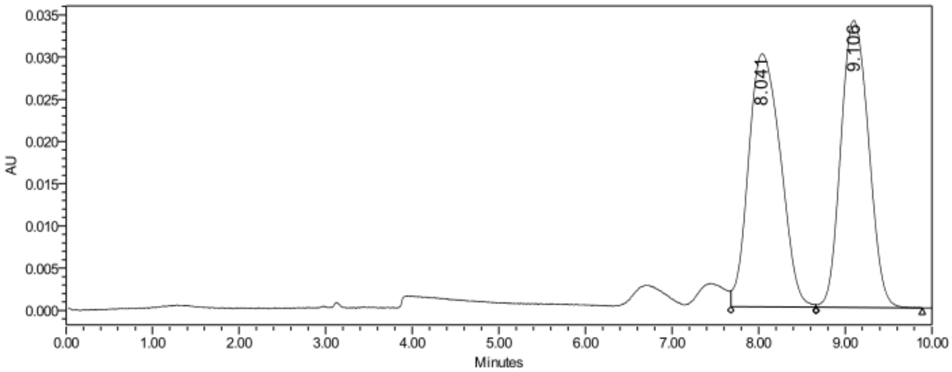

	RT	Area	% Area	Height
1	8.041	772339	50.56	29966
2	9.106	755163	49.44	33983

Asy-**3a**

SAMPLE INFORMATION

Sample Name:	XT-8-382-ASY-1%-IG	Acquired By:	System
Sample Type:	Unknown	Sample Set Name:	
Vial:	21	Acq. Method Set:	1% quanbo
Injection #:	1	Processing Method	XT 8 38 2 re ASY
Injection Volume:	20.00 ul	Channel Name:	254.0nm
Run Time:	60.0 Minutes	Proc. Chnl. Descr.:	2998 PDA 254.0 nm (2998
Date Acquired:	9/13/2024 21:44:02 CST		
Date Processed:	2/22/2025 21:05:21 CST		

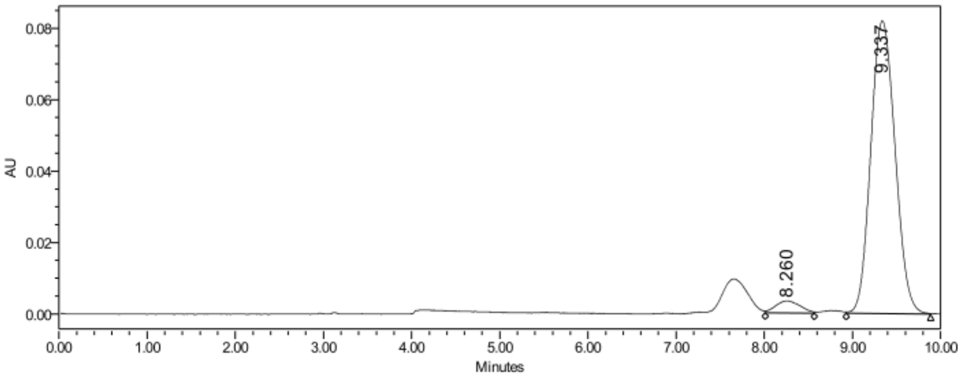

	RT	Area	% Area	Height
1	8.260	64215	3.94	3341
2	9.337	1565347	96.06	81967

Rac-**3b** (The product was isolated as a 6:1 mixture of E/Z isomers)

SAMPLE INFORMATION

Sample Name:	xt-7-194-1-RAC-2%-IG	Acquired By:	System
Sample Type:	Unknown	Sample Set Name	
Vial:	51	Acq. Method Set:	2% quanbo
Injection #:	1	Processing Method	xt 7 194 1 rac
Injection Volume:	10.00 ul	Channel Name:	254.0nm
Run Time:	60.0 Minutes	Proc. Chnl. Descr.:	2998 PDA 254.0 nm (2998
Date Acquired:	8/15/2024 22:05:15 CST		
Date Processed:	8/15/2024 23:05:32 CST		

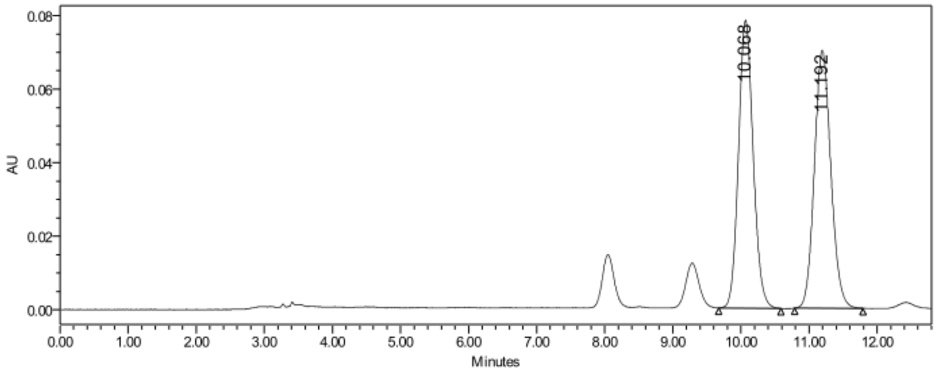

	RT	Area	% Area	Height
1	10.068	1176828	49.99	78295
2	11.192	1177071	50.01	70098

Asy-**3b**

SAMPLE INFORMATION

Sample Name:	xt-7-194-2-ASY-2%-IG	Acquired By:	System
Sample Type:	Unknown	Sample Set Name	20240815
Vial:	50	Acq. Method Set:	2% quanbo
Injection #:	1	Processing Method	xt 7 194 2 asy
Injection Volume:	10.00 ul	Channel Name:	252.2nm
Run Time:	15.0 Minutes	Proc. Chnl. Descr.:	2998 PDA 252.2 nm (2998
Date Acquired:	8/15/2024 22:25:24 CST		
Date Processed:	8/15/2024 23:07:18 CST		

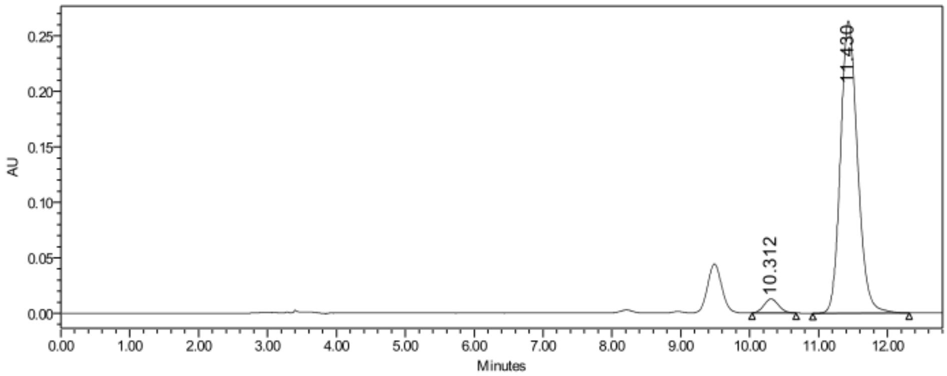

	RT	Area	% Area	Height
1	10.312	186928	3.94	12567
2	11.430	4555179	96.06	263628

Rac-**3c** (The product was isolated as a 6:1 mixture of E/Z isomers)

SAMPLE INFORMATION

Sample Name:	XT-7-192-1-rac-5%	Acquired By:	System
Sample Type:	Unknown	Sample Set Name	
Vial:	28	Acq. Method Set:	5% quanbo
Injection #:	1	Processing Method	XT 7 192 1 RAC
Injection Volume:	10.00 ul	Channel Name:	254.0nm
Run Time:	60.0 Minutes	Proc. Chnl. Descr.:	2998 PDA 254.0 nm (2998
Date Acquired:	9/14/2024 20:50:02 CST		
Date Processed:	9/17/2024 16:36:42 CST		

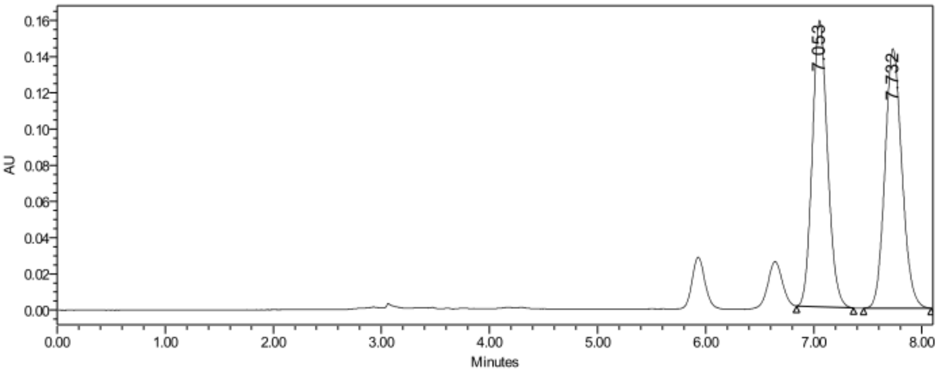

	RT	Area	% Area	Height
1	7.053	1556827	49.68	158302
2	7.732	1576931	50.32	143351

Asy-**3c**

SAMPLE INFORMATION

Sample Name:	XT-7-192-2-asy-5%-IG	Acquired By:	System
Sample Type:	Unknown	Sample Set Name	
Vial:	100	Acq. Method Set:	5% quanbo
Injection #:	1	Processing Method	XT 7 192 2 ASY
Injection Volume:	30.00 ul	Channel Name:	254.0nm
Run Time:	60.0 Minutes	Proc. Chnl. Descr.:	2998 PDA 254.0 nm (2998
Date Acquired:	9/17/2024 16:25:06 CST		
Date Processed:	9/17/2024 16:35:03 CST		

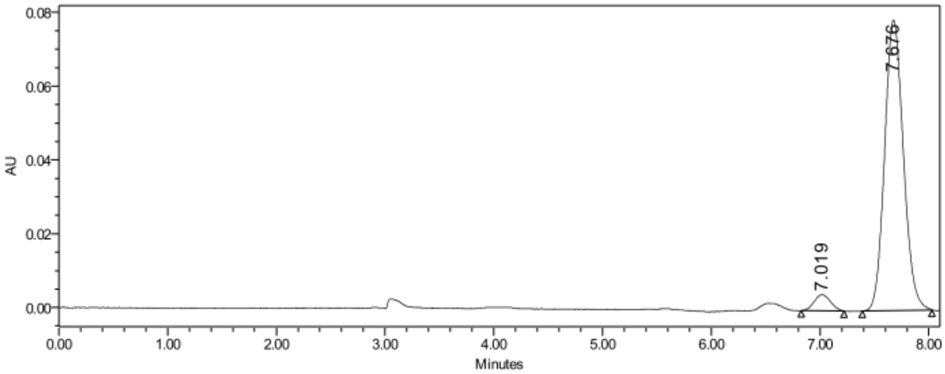

	RT	Area	% Area	Height
1	7.019	45882	4.64	4394
2	7.676	942803	95.36	78661

Rac-**3d** (The product was isolated as an 8:1 mixture of E/Z isomers)

SAMPLE INFORMATION

Sample Name:	xt-8-9-1-RAC-10%-IG	Acquired By:	System
Sample Type:	Unknown	Sample Set Name:	
Vial:	42	Acq. Method Set:	10%qb
Injection #:	1	Processing Method:	XT 8 9 1 RAC
Injection Volume:	10.00 ul	Channel Name:	233.0nm
Run Time:	60.0 Minutes	Proc. Chnl. Descr.:	2998 PDA 233.0 nm (2998
Date Acquired:	8/28/2024 10:14:55 PM CST		
Date Processed:	8/28/2024 10:57:26 PM CST		

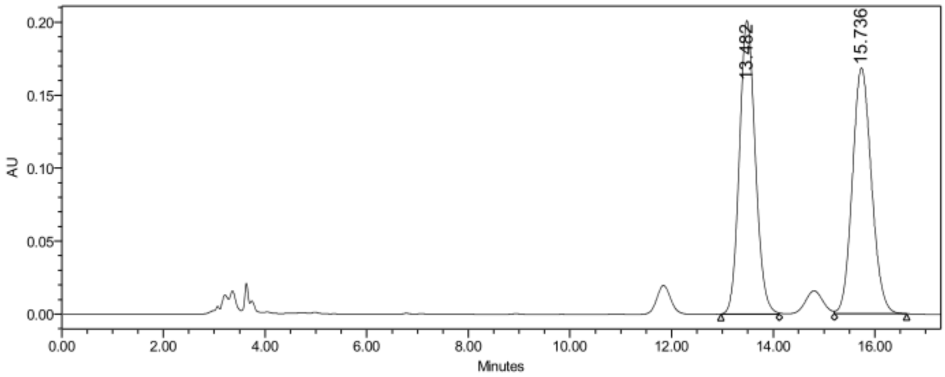

	RT	Area	% Area	Height
1	13.482	4404046	50.12	200954
2	15.736	4383648	49.88	168512

Asy-**3d**

SAMPLE INFORMATION

Sample Name:	xt-8-9-2-ASY-10%-IG	Acquired By:	System
Sample Type:	Unknown	Sample Set Name:	20240828
Vial:	68	Acq. Method Set:	10%qb
Injection #:	1	Processing Method:	XT 8 9 2 ASY
Injection Volume:	20.00 ul	Channel Name:	233.0nm
Run Time:	20.0 Minutes	Proc. Chnl. Descr.:	2998 PDA 233.0 nm (2998
Date Acquired:	8/28/2024 10:35:26 PM CST		
Date Processed:	8/28/2024 10:55:14 PM CST		

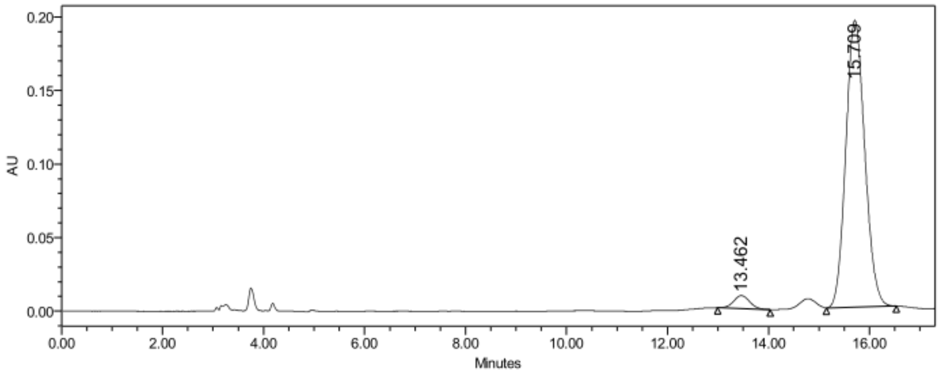

	RT	Area	% Area	Height
1	13.462	192631	3.67	8837
2	15.709	5051069	96.33	195001

Rac-**3e** (The product was isolated as a 5:1 mixture of E/Z isomers)

SAMPLE INFORMATION

Sample Name:	xt-8-3-1-RAC-30%-IG	Acquired By:	System
Sample Type:	Unknown	Sample Set Name	
Vial:	62	Acq. Method Set:	30%quanbo
Injection #:	1	Processing Method	xt 8 3 1 rac
Injection Volume:	10.00 ul	Channel Name:	254.0nm
Run Time:	60.0 Minutes	Proc. Chnl. Descr.:	2998 PDA 254.0 nm (2998
Date Acquired:	8/22/2024 20:39:59 CST		
Date Processed:	8/22/2024 21:38:09 CST		

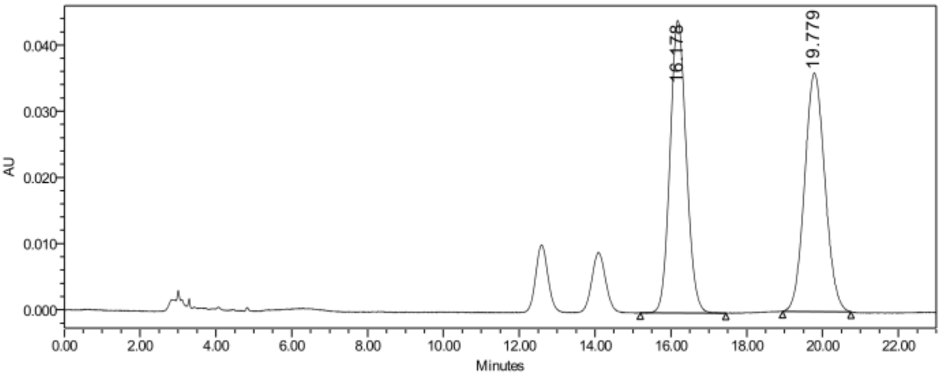

	RT	Area	% Area	Height
1	16.178	1313113	49.45	44173
2	19.779	1342503	50.55	36064

Asy-**3e**

SAMPLE INFORMATION

Sample Name:	xt-8-3-2-asy-30%-IG	Acquired By:	System
Sample Type:	Unknown	Sample Set Name	
Vial:	86	Acq. Method Set:	30%quanbo
Injection #:	1	Processing Method	xt 8 3 2 asy
Injection Volume:	10.00 ul	Channel Name:	254.0nm
Run Time:	60.0 Minutes	Proc. Chnl. Descr.:	2998 PDA 254.0 nm (2998
Date Acquired:	8/22/2024 21:08:56 CST		
Date Processed:	8/22/2024 21:36:25 CST		

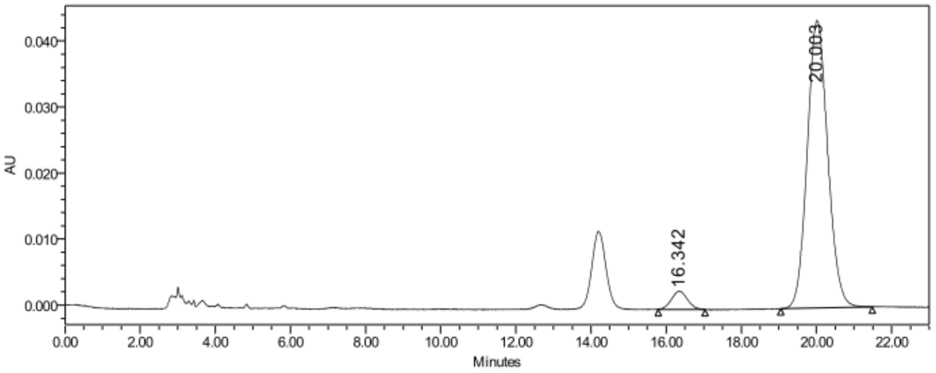

	RT	Area	% Area	Height
1	16.342	80847	4.65	2787
2	20.003	1656966	95.35	43580

Rac-**3f** (The product was isolated as a 9:1 mixture of E/Z isomers)

SAMPLE INFORMATION

Sample Name:	xt-8-18-1-rac-2%-IG	Acquired By:	System
Sample Type:	Unknown	Sample Set Name	20240903
Vial:	65	Acq. Method Set:	2% quanbo
Injection #:	1	Processing Method	xt 8 18 1 rac
Injection Volume:	20.00 ul	Channel Name:	254.0nm
Run Time:	8.0 Minutes	Proc. Chnl. Descr.:	2998 PDA 254.0 nm (2998
Date Acquired:	9/3/2024 11:37:13 CST		
Date Processed:	9/4/2024 20:08:46 CST		

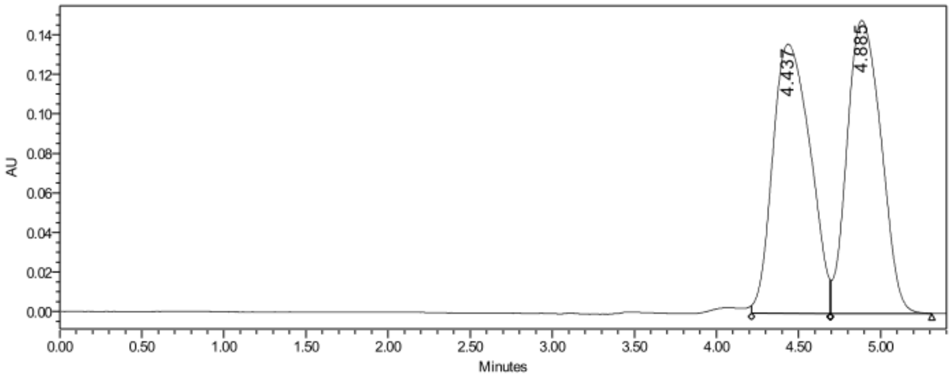

	RT	Area	% Area	Height
1	4.437	2158408	50.18	136133
2	4.885	2143330	49.82	148144

Asy-**3f**

SAMPLE INFORMATION

Sample Name:	xt-8-18-2-asy-2%-IG	Acquired By:	System
Sample Type:	Unknown	Sample Set Name	
Vial:	15	Acq. Method Set:	2% quanbo
Injection #:	1	Processing Method	xt 8 18 2 asy
Injection Volume:	20.00 ul	Channel Name:	254.0nm
Run Time:	60.0 Minutes	Proc. Chnl. Descr.:	2998 PDA 254.0 nm (2998
Date Acquired:	9/4/2024 19:56:38 CST		
Date Processed:	9/4/2024 20:06:46 CST		

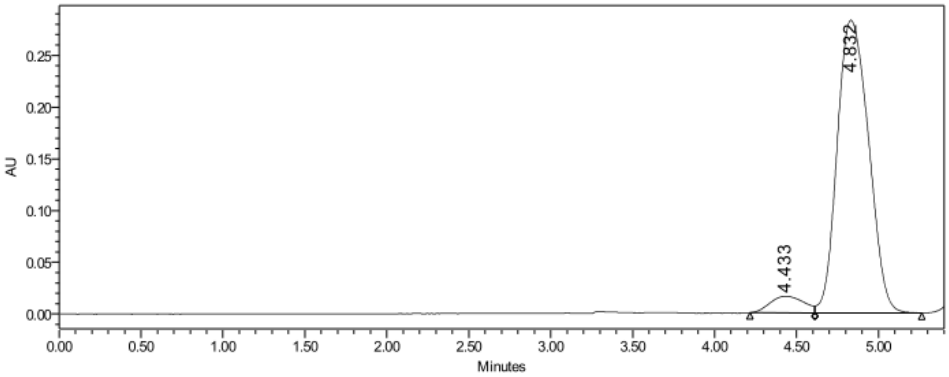

	RT	Area	% Area	Height
1	4.433	227574	5.69	15943
2	4.832	3770527	94.31	282908

Rac-**3g** (The product was isolated as a 5:1 mixture of E/Z isomers)

SAMPLE INFORMATION

Sample Name:	xt-8-7-1-rac-2%-IG	Acquired By:	System
Sample Type:	Unknown	Sample Set Name	20240823
Vial:	1	Acq. Method Set:	2% quanbo
Injection #:	1	Processing Method	xt 8 7 1 rac
Injection Volume:	10.00 ul	Channel Name:	254.0nm
Run Time:	20.0 Minutes	Proc. Chnl. Descr.:	2998 PDA 254.0 nm (2998
Date Acquired:	8/23/2024 10:59:38 CST		
Date Processed:	8/23/2024 11:22:58 CST		

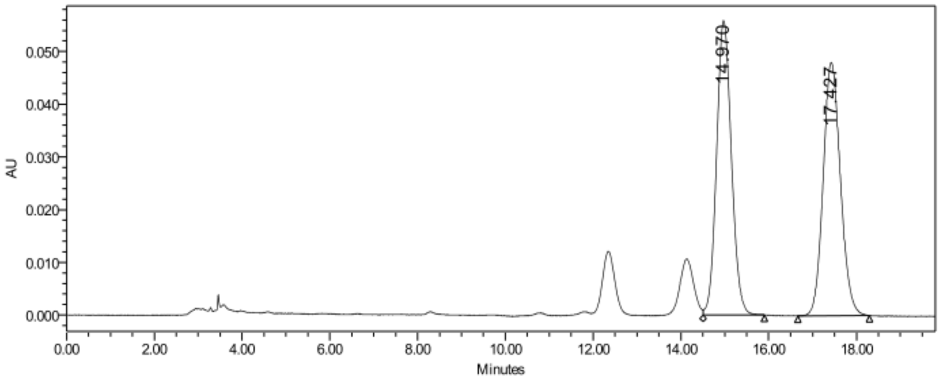

	RT	Area	% Area	Height
1	14.970	1312864	50.01	55846
2	17.427	1312241	49.99	48023

Asy-**3g**

SAMPLE INFORMATION

Sample Name:	xt-8-7-2-asy-2%-IG	Acquired By:	System
Sample Type:	Unknown	Sample Set Name	
Vial:	102	Acq. Method Set:	2% quanbo
Injection #:	1	Processing Method	xt 8 7 2 asy
Injection Volume:	20.00 ul	Channel Name:	254.0nm
Run Time:	60.0 Minutes	Proc. Chnl. Descr.:	2998 PDA 254.0 nm (2998
Date Acquired:	8/23/2024 10:33:30 CST		
Date Processed:	8/23/2024 11:24:39 CST		

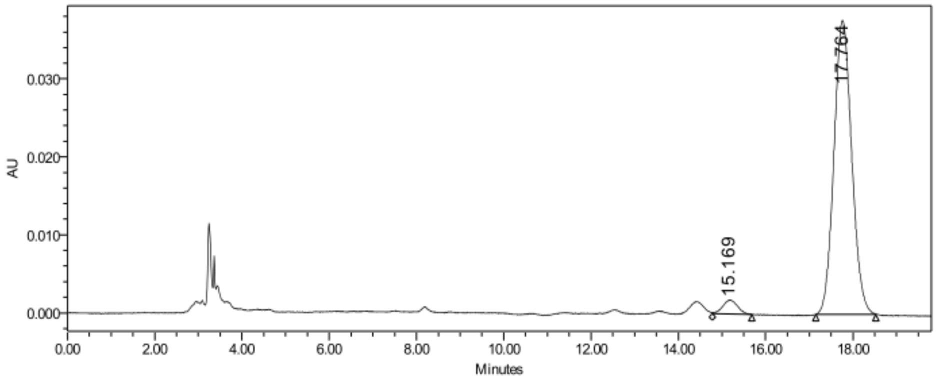

	RT	Area	% Area	Height
1	15.169	42468	3.83	1796
2	17.764	1066950	96.17	37677

Rac-**3h**

SAMPLE INFORMATION

Sample Name:	xt-8-28-1-rac-20%-IG	Acquired By:	System
Sample Type:	Unknown	Sample Set Name	
Vial:	84	Acq. Method Set:	20% quanbo
Injection #:	1	Processing Method	xt 8 28 1 rac
Injection Volume:	10.00 ul	Channel Name:	254.0nm
Run Time:	60.0 Minutes	Proc. Chnl. Descr.:	2998 PDA 254.0 nm (2998
Date Acquired:	9/5/2024 20:01:04 CST		
Date Processed:	9/5/2024 21:18:47 CST		

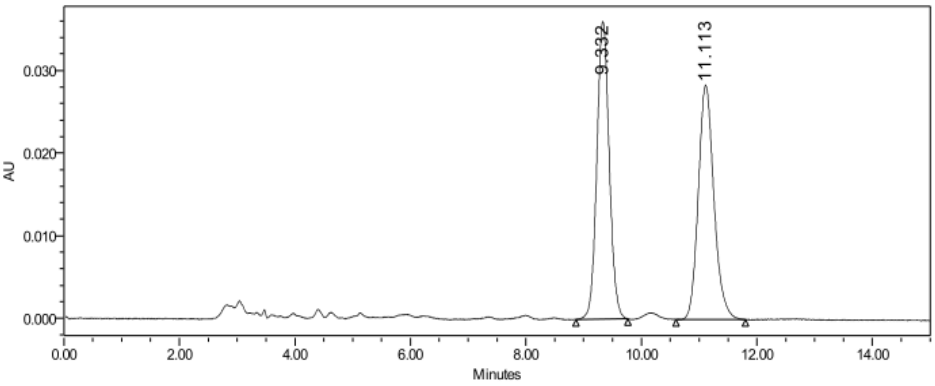

	RT	Area	% Area	Height
1	9.332	535736	50.12	36035
2	11.113	533235	49.88	28382

Asy-**3h**

SAMPLE INFORMATION

Sample Name:	xt-8-28-2-ASY-20%-IG	Acquired By:	System
Sample Type:	Unknown	Sample Set Name	
Vial:	31	Acq. Method Set:	20% quanbo
Injection #:	1	Processing Method	xt 8 28 2 asy
Injection Volume:	10.00 ul	Channel Name:	254.0nm
Run Time:	60.0 Minutes	Proc. Chnl. Descr.:	2998 PDA 254.0 nm (2998
Date Acquired:	9/5/2024 21:01:37 CST		
Date Processed:	9/5/2024 21:17:27 CST		

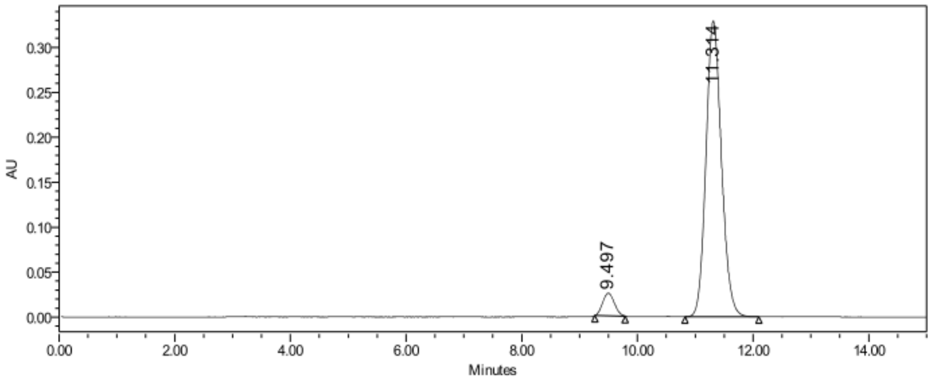

	RT	Area	% Area	Height
1	9.497	354011	5.48	25226
2	11.314	6107128	94.52	329300

Rac-**3i**

SAMPLE INFORMATION

Sample Name:	xt-8-26-1-rac-20%IG	Acquired By:	System
Sample Type:	Unknown	Sample Set Name:	02040905
Vial:	75	Acq. Method Set:	20%qb
Injection #:	1	Processing Method:	XT 8 26 1 RAC
Injection Volume:	10.00 ul	Channel Name:	254.0nm
Run Time:	20.0 Minutes	Proc. Chnl. Descr.:	2998 PDA 254.0 nm (2998
Date Acquired:	9/5/2024 5:59:16 PM CST		
Date Processed:	9/5/2024 8:06:57 PM CST		

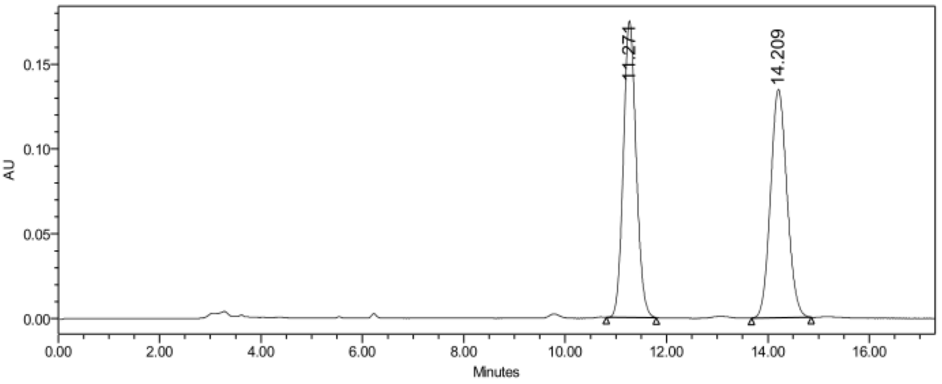

	RT	Area	% Area	Height
1	11.271	3052517	50.25	174836
2	14.209	3021835	49.75	134723

Asy-**3i**

SAMPLE INFORMATION

Sample Name:	xt-8-26-2-ASY-20%IG	Acquired By:	System
Sample Type:	Unknown	Sample Set Name:	02040905
Vial:	74	Acq. Method Set:	20%qb
Injection #:	1	Processing Method:	XT 8 26 2 ASY
Injection Volume:	10.00 ul	Channel Name:	254.0nm
Run Time:	20.0 Minutes	Proc. Chnl. Descr.:	2998 PDA 254.0 nm (2998
Date Acquired:	9/5/2024 5:38:36 PM CST		
Date Processed:	9/5/2024 8:05:21 PM CST		

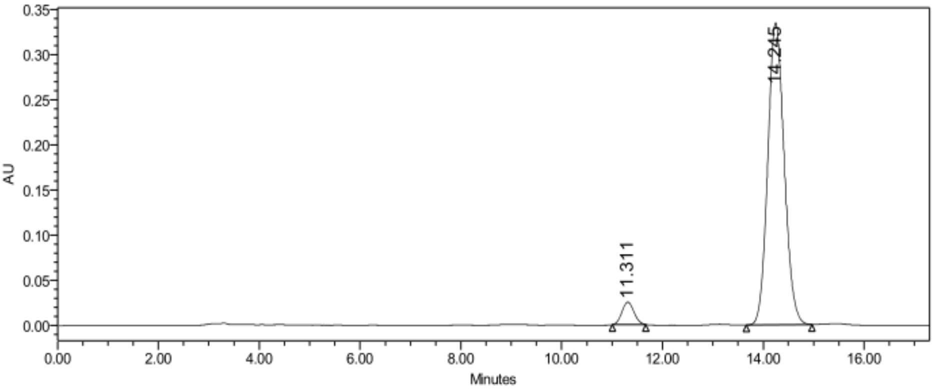

	RT	Area	% Area	Height
1	11.311	416487	5.25	25012
2	14.245	7524126	94.75	334489

Rac-**3j**

SAMPLE INFORMATION

Sample Name:	XT-8-32-1-RAC-20%-IG	Acquired By:	System
Sample Type:	Unknown	Sample Set Name:	20240910
Vial:	37	Acq. Method Set:	20%qb
Injection #:	1	Processing Method:	XT 8 32 1 RAC
Injection Volume:	20.00 ul	Channel Name:	254.0nm
Run Time:	15.0 Minutes	Proc. Chnl. Descr.:	2998 PDA 254.0 nm (2998
Date Acquired:	9/10/2024 10:29:34 PM CST		
Date Processed:	9/11/2024 9:08:45 AM CST		

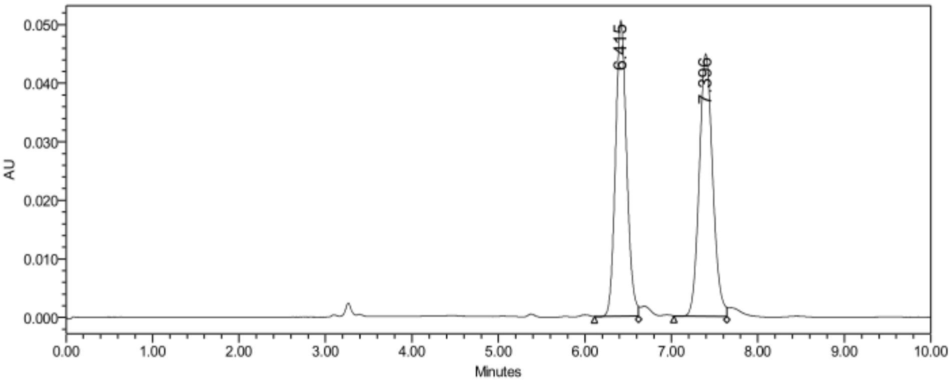

	RT	Area	% Area	Height
1	6.415	455385	48.56	50504
2	7.396	482354	51.44	44786

Asy-**3j**

SAMPLE INFORMATION

Sample Name:	xt-8-32-2-asy-20%-IG	Acquired By:	System
Sample Type:	Unknown	Sample Set Name	20240909
Vial:	70	Acq. Method Set:	20% quanbo
Injection #:	1	Processing Method	xt 8 32 2 asy
Injection Volume:	10.00 ul	Channel Name:	254.0nm
Run Time:	10.0 Minutes	Proc. Chnl. Descr.:	2998 PDA 254.0 nm (2998
Date Acquired:	9/9/2024 22:11:49 CST		
Date Processed:	9/9/2024 22:33:02 CST		

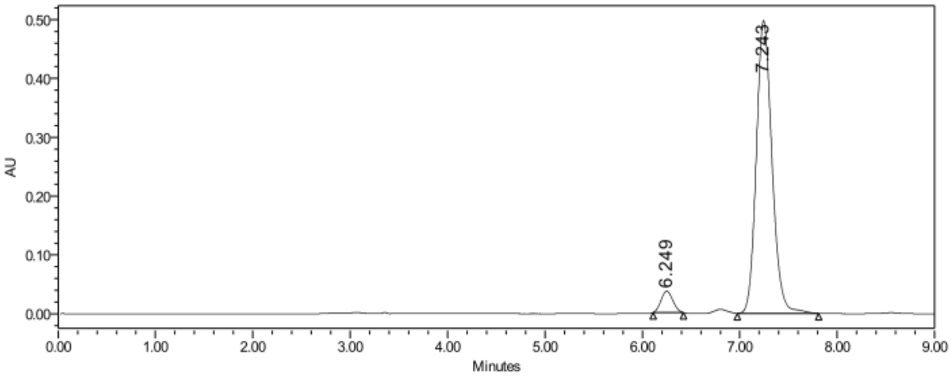

	RT	Area	% Area	Height
1	6.249	307822	5.23	36416
2	7.243	5572499	94.77	497941

Rac-**3k** (The product was isolated as a 5:1 mixture of E/Z isomers)

SAMPLE INFORMATION

Sample Name:	XT-8-36-1-RAC-15%-IE	Acquired By:	System
Sample Type:	Unknown	Sample Set Name:	20240913
Vial:	28	Acq. Method Set:	15% quanbo
Injection #:	1	Processing Method	XT 8 36 1 RAC
Injection Volume:	20.00 ul	Channel Name:	254.0nm
Run Time:	32.0 Minutes	Proc. Chnl. Descr.:	2998 PDA 254.0 nm (2998
Date Acquired:	9/13/2024 11:33:05 CST		
Date Processed:	9/13/2024 23:05:19 CST		

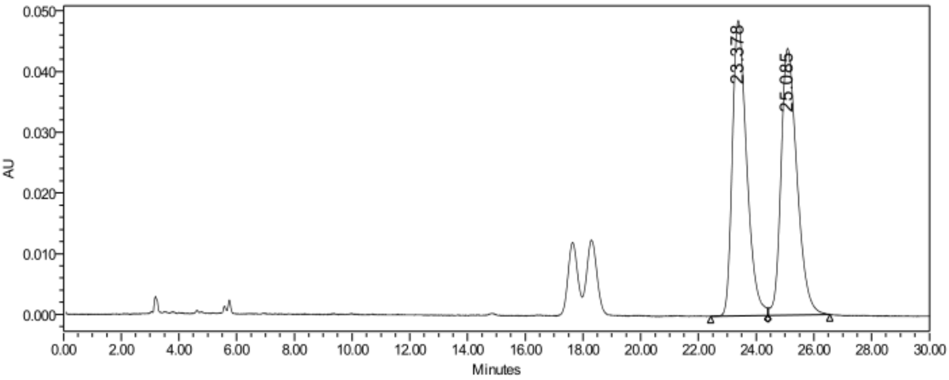

	RT	Area	% Area	Height
1	23.378	1680611	49.92	48620
2	25.085	1686130	50.08	43932

Asy-**3k**

SAMPLE INFORMATION

Sample Name:	XT-8-36-2-ASY-15%-IE	Acquired By:	System
Sample Type:	Unknown	Sample Set Name:	
Vial:	26	Acq. Method Set:	15% quanbo
Injection #:	1	Processing Method	XT 8 36 2 ASY
Injection Volume:	20.00 ul	Channel Name:	254.0nm
Run Time:	60.0 Minutes	Proc. Chnl. Descr.:	2998 PDA 254.0 nm (2998
Date Acquired:	9/13/2024 15:09:34 CST		
Date Processed:	9/13/2024 23:03:41 CST		

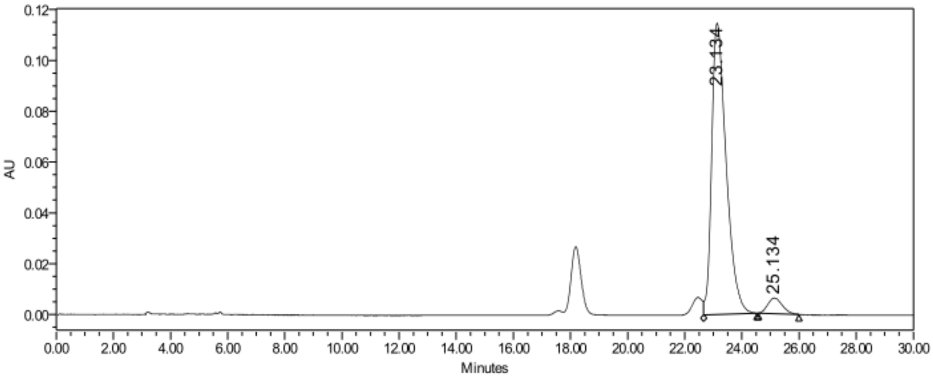

	RT	Area	% Area	Height
1	23.134	4045770	94.91	114505
2	25.134	216764	5.09	6195

Rac-**3l**

SAMPLE INFORMATION

Sample Name:	xt-8-24-1-rac-20%-IG	Acquired By:	System
Sample Type:	Unknown	Sample Set Name	
Vial:	99	Acq. Method Set:	20% quanbo
Injection #:	1	Processing Method	xt 8 24 1 rac
Injection Volume:	20.00 ul	Channel Name:	254.0nm
Run Time:	60.0 Minutes	Proc. Chnl. Descr.:	2998 PDA 254.0 nm (2998
Date Acquired:	9/3/2024 21:11:02 CST		
Date Processed:	9/5/2024 20:12:18 CST		

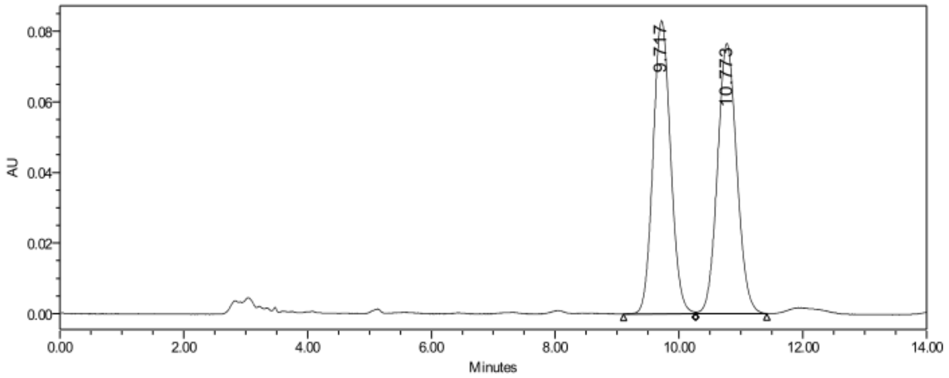

	RT	Area	% Area	Height
1	9.717	1625170	49.30	83155
2	10.773	1671532	50.70	76567

Asy-**3l**

SAMPLE INFORMATION

Sample Name:	xt-8-24-2-asy-20%-IG	Acquired By:	System
Sample Type:	Unknown	Sample Set Name	20240903
Vial:	18	Acq. Method Set:	20% quanbo
Injection #:	1	Processing Method	xt 8 24 2 asy
Injection Volume:	10.00 ul	Channel Name:	254.0nm
Run Time:	14.0 Minutes	Proc. Chnl. Descr.:	2998 PDA 254.0 nm (2998
Date Acquired:	9/3/2024 21:27:52 CST		
Date Processed:	9/5/2024 20:10:07 CST		

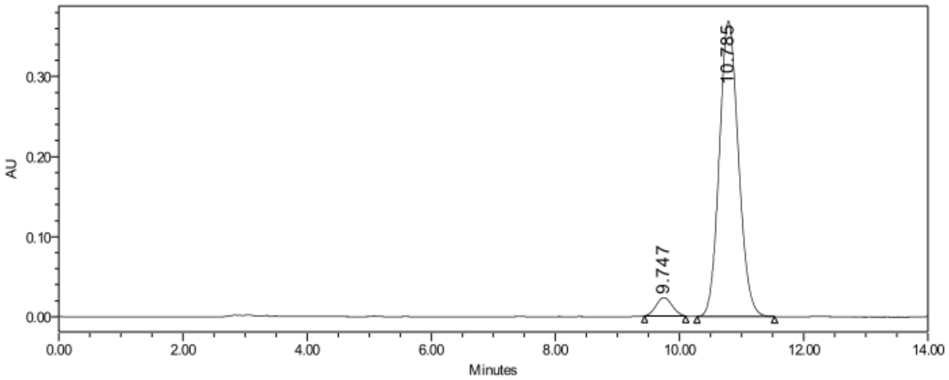

	RT	Area	% Area	Height
1	9.747	410976	4.92	22830
2	10.785	7948851	95.08	369326

Rac-**3m**

SAMPLE INFORMATION

Sample Name:	xt-7-184-1-rac-2%-IG	Acquired By:	System
Sample Type:	Unknown	Sample Set Name	20240801
Vial:	37	Acq. Method Set:	2% quanbo
Injection #:	1	Processing Method	xt 7 184 1 rac
Injection Volume:	10.00 ul	Channel Name:	254.0nm
Run Time:	16.0 Minutes	Proc. Chnl. Descr.:	2998 PDA 254.0 nm (2998
Date Acquired:	8/1/2024 11:50:32 CST		
Date Processed:	8/1/2024 14:38:50 CST		

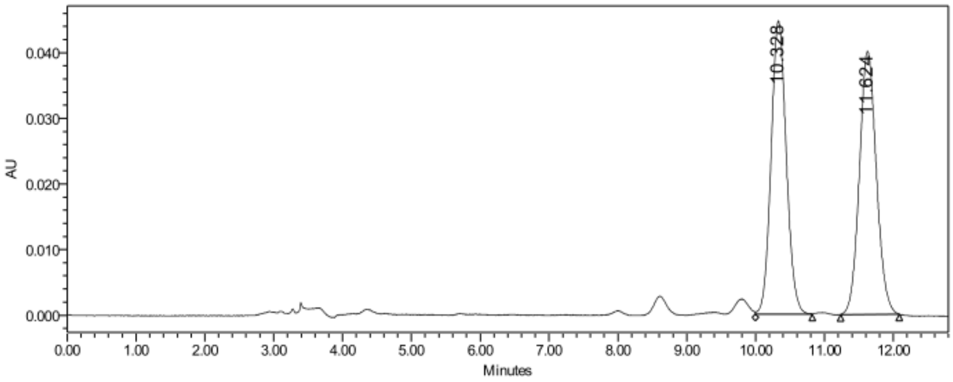

	RT	Area	% Area	Height
1	10.328	685055	49.88	44681
2	11.624	688349	50.12	40099

Asy-**3m**

SAMPLE INFORMATION

Sample Name:	xt-7-180-1-asy-2%-IG	Acquired By:	System
Sample Type:	Unknown	Sample Set Name	20240801
Vial:	35	Acq. Method Set:	2% quanbo
Injection #:	1	Processing Method	xt 7 180 1 asy
Injection Volume:	10.00 ul	Channel Name:	254.0nm
Run Time:	16.0 Minutes	Proc. Chnl. Descr.:	2998 PDA 254.0 nm (2998
Date Acquired:	8/1/2024 11:17:09 CST		
Date Processed:	8/1/2024 14:36:44 CST		

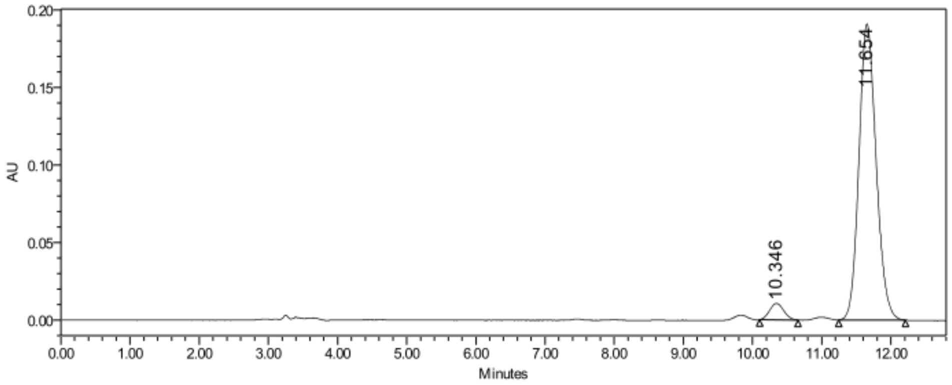

	RT	Area	% Area	Height
1	10.346	152167	4.40	10596
2	11.654	3305844	95.60	190926

Rac-**3n**

SAMPLE INFORMATION

Sample Name:	xt-7-188-1-rac-2%-IG	Acquired By:	System
Sample Type:	Unknown	Sample Set Name:	20240803
Vial:	111	Acq. Method Set:	2%qb
Injection #:	1	Processing Method:	XT 7 188 1 rac
Injection Volume:	10.00 ul	Channel Name:	254.0nm
Run Time:	16.0 Minutes	Proc. Chnl. Descr.:	2998 PDA 254.0 nm (2998
Date Acquired:	8/3/2024 4:19:58 PM CST		
Date Processed:	8/21/2024 10:32:01 PM CST		

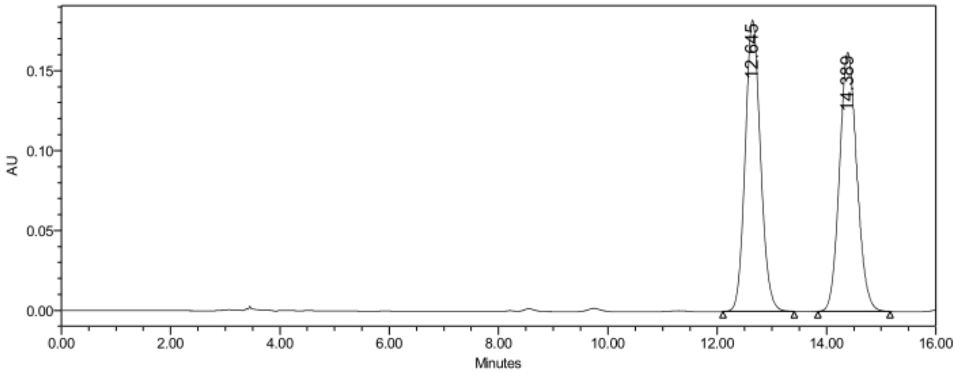

	RT	Area	% Area	Height
1	12.645	3570106	49.99	182318
2	14.389	3572226	50.01	162189

Asy-**3n**

SAMPLE INFORMATION

Sample Name:	xt-7-188-2-asy-2%-IG	Acquired By:	System
Sample Type:	Unknown	Sample Set Name:	20240803
Vial:	112	Acq. Method Set:	2%qb
Injection #:	1	Processing Method:	XT 7 188 2 ASY
Injection Volume:	10.00 ul	Channel Name:	254.0nm
Run Time:	16.0 Minutes	Proc. Chnl. Descr.:	2998 PDA 254.0 nm (2998
Date Acquired:	8/3/2024 4:36:41 PM CST		
Date Processed:	8/21/2024 10:40:12 PM CST		

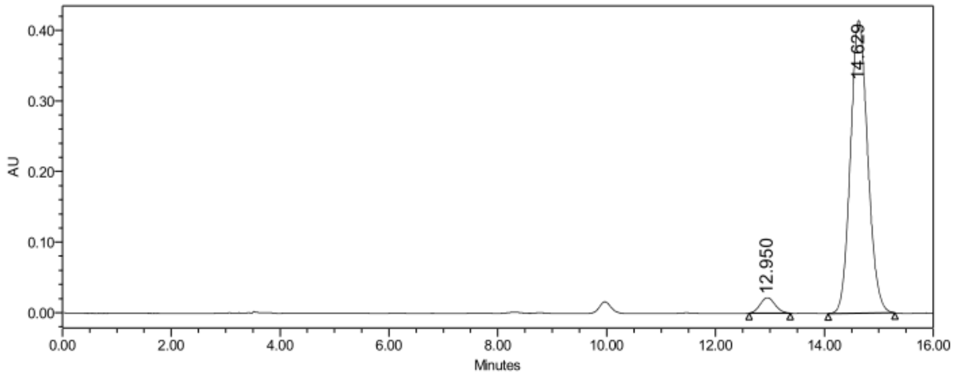

	RT	Area	% Area	Height
1	12.950	415396	4.37	21864
2	14.629	9094183	95.63	414819

Rac-**3o**

SAMPLE INFORMATION

Sample Name:	XT-8-64-1-rac-2%-IG	Acquired By:	System
Sample Type:	Unknown	Sample Set Name	
Vial:	55	Acq. Method Set:	2% quanbo
Injection #:	1	Processing Method	XT 8 64 1 RAC
Injection Volume:	10.00 ul	Channel Name:	254.0nm
Run Time:	60.0 Minutes	Proc. Chnl. Descr.:	2998 PDA 254.0 nm (2998
Date Acquired:	9/30/2024 17:17:24 CST		
Date Processed:	10/1/2024 20:08:19 CST		

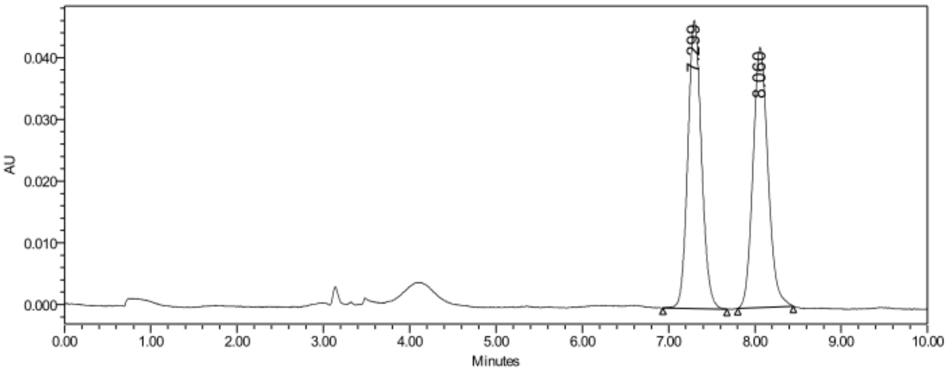

	RT	Area	% Area	Height
1	7.299	510063	49.98	46659
2	8.060	510411	50.02	42172

Asy-**3o**

SAMPLE INFORMATION

Sample Name:	XT-8-64-2-asy-2%-IG	Acquired By:	System
Sample Type:	Unknown	Sample Set Name	
Vial:	36	Acq. Method Set:	2% quanbo
Injection #:	1	Processing Method	XT 8 64 2 ASY
Injection Volume:	30.00 ul	Channel Name:	254.0nm
Run Time:	60.0 Minutes	Proc. Chnl. Descr.:	2998 PDA 254.0 nm (2998
Date Acquired:	10/1/2024 19:54:17 CST		
Date Processed:	10/1/2024 20:06:08 CST		

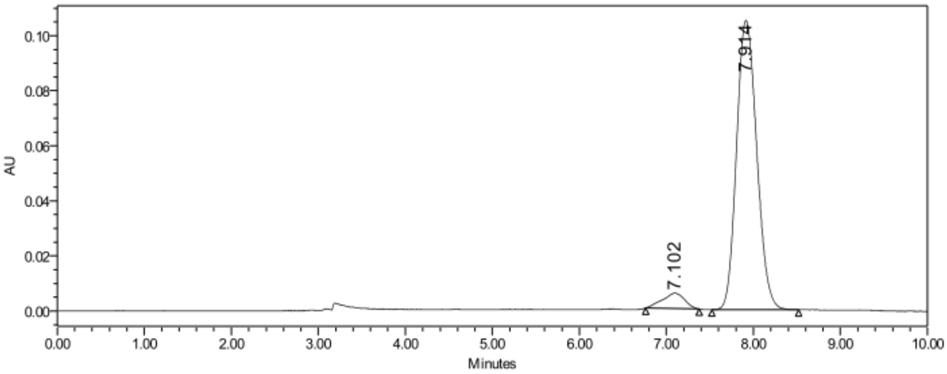

	RT	Area	% Area	Height
1	7.102	99539	5.56	5600
2	7.914	1689132	94.44	105136

Rac-**3p**

SAMPLE INFORMATION

Sample Name:	XT-8-66-1-RAC-2%-IG	Acquired By:	System
Sample Type:	Unknown	Sample Set Name	
Vial:	29	Acq. Method Set:	2% quanbo
Injection #:	1	Processing Method	XT 8 66 1 RAC
Injection Volume:	10.00 ul	Channel Name:	254.0nm
Run Time:	60.0 Minutes	Proc. Chnl. Descr.:	2998 PDA 254.0 nm (2998
Date Acquired:	9/28/2024 11:01:34 CST		
Date Processed:	9/28/2024 11:33:44 CST		

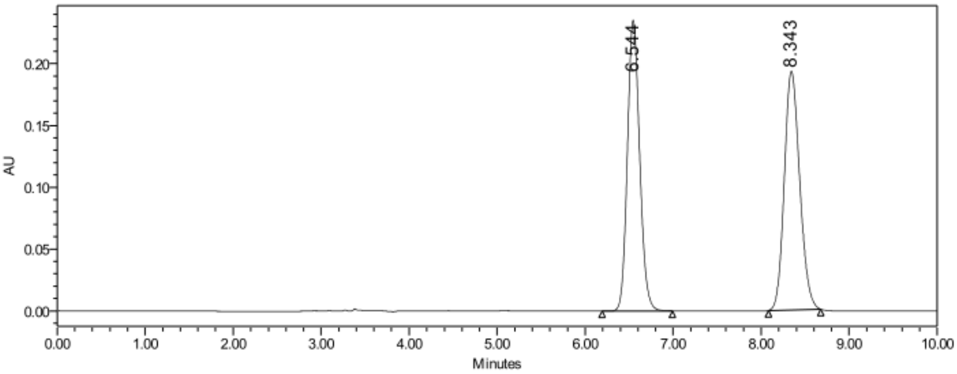

	RT	Area	% Area	Height
1	6.544	2288327	49.24	235192
2	8.343	2359253	50.76	193167

Asy-**3p**

SAMPLE INFORMATION

Sample Name:	XT-8-66-2-ASY-2%-IG	Acquired By:	System
Sample Type:	Unknown	Sample Set Name	0928
Vial:	64	Acq. Method Set:	2% quanbo
Injection #:	1	Processing Method	XT 8 66 2 ASY
Injection Volume:	10.00 ul	Channel Name:	254.0nm
Run Time:	11.0 Minutes	Proc. Chnl. Descr.:	2998 PDA 254.0 nm (2998
Date Acquired:	9/28/2024 11:19:39 CST		
Date Processed:	9/28/2024 11:32:34 CST		

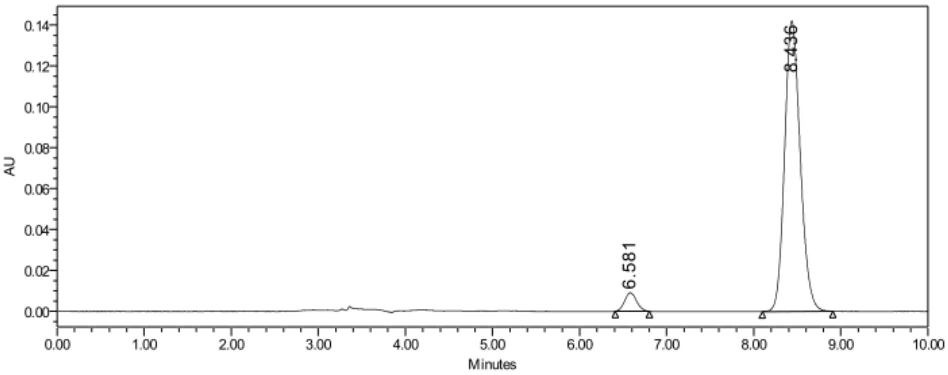

	RT	Area	% Area	Height
1	6.581	85891	4.61	9055
2	8.436	1777317	95.39	142144

Rac-**3q**

SAMPLE INFORMATION

Sample Name:	xt-8-60-1-RAC-2%-IG	Acquired By:	System
Sample Type:	Unknown	Sample Set Name:	
Vial:	53	Acq. Method Set:	2%qb
Injection #:	1	Processing Method:	XT 8 60 1 RAC
Injection Volume:	10.00 ul	Channel Name:	254.0nm
Run Time:	60.0 Minutes	Proc. Chnl. Descr.:	2998 PDA 254.0 nm (2998
Date Acquired:	9/25/2024 4:51:12 PM CST		
Date Processed:	9/25/2024 5:22:29 PM CST		

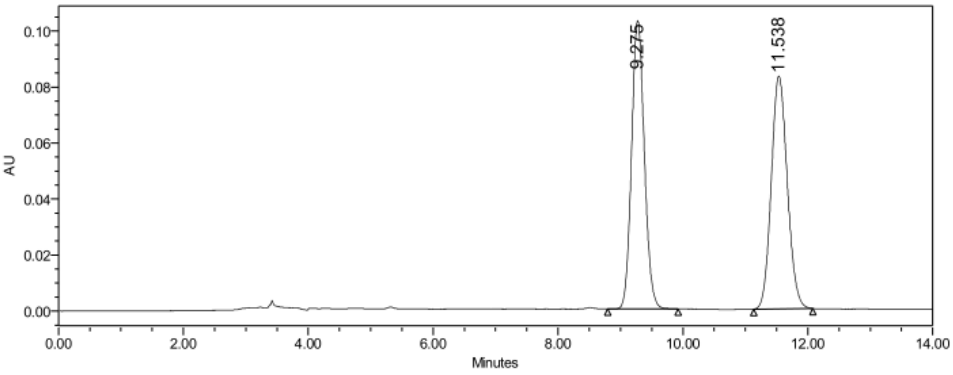

	RT	Area	% Area	Height
1	9.275	1453485	49.62	103030
2	11.538	1475837	50.38	83153

Asy-**3q**

SAMPLE INFORMATION

Sample Name:	xt-8-60-2-ASY-2%-IG	Acquired By:	System
Sample Type:	Unknown	Sample Set Name:	
Vial:	86	Acq. Method Set:	2%qb
Injection #:	1	Processing Method:	XT 8 60 2 ASY
Injection Volume:	10.00 ul	Channel Name:	254.0nm
Run Time:	60.0 Minutes	Proc. Chnl. Descr.:	2998 PDA 254.0 nm (2998
Date Acquired:	9/25/2024 5:09:09 PM CST		
Date Processed:	9/25/2024 5:23:59 PM CST		

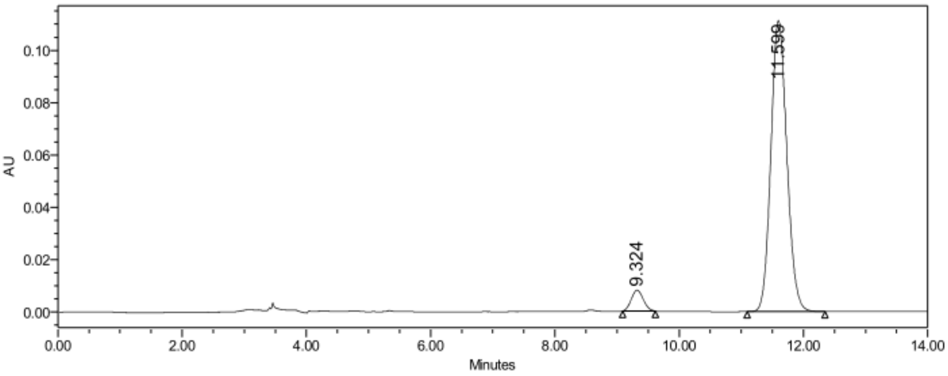

	RT	Area	% Area	Height
1	9.324	106808	5.14	7968
2	11.599	1972243	94.86	111240

Rac-**3r**

SAMPLE INFORMATION

Sample Name:	xt-8-57-1-RAC-20%-IG	Acquired By:	System
Sample Type:	Unknown	Sample Set Name:	
Vial:	95	Acq. Method Set:	20%qb
Injection #:	1	Processing Method:	XT 8 57 1 RAC
Injection Volume:	10.00 ul	Channel Name:	254.0nm
Run Time:	60.0 Minutes	Proc. Chnl. Descr.:	2998 PDA 254.0 nm (2998
Date Acquired:	9/25/2024 7:33:15 PM CST		
Date Processed:	9/25/2024 8:00:58 PM CST		

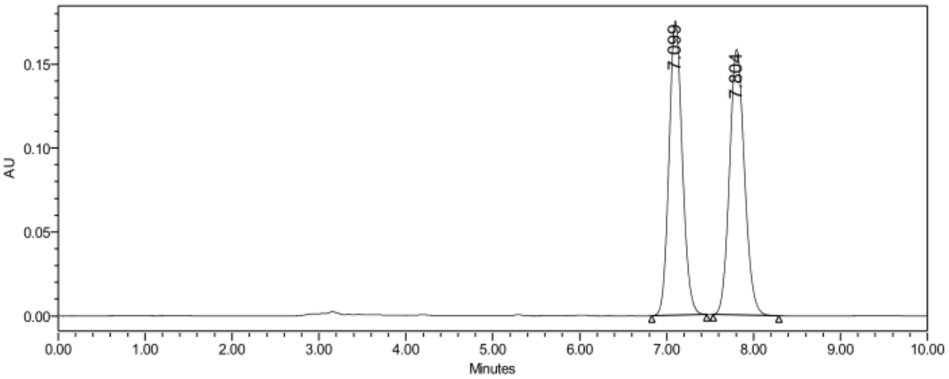

	RT	Area	% Area	Height
1	7.099	1867377	49.54	175568
2	7.804	1902011	50.46	158276

Asy-**3r**

SAMPLE INFORMATION

Sample Name:	xt-8-57-2-ASY-20%-IG	Acquired By:	System
Sample Type:	Unknown	Sample Set Name:	
Vial:	108	Acq. Method Set:	20%qb
Injection #:	1	Processing Method:	XT 8 57 2 ASY
Injection Volume:	10.00 ul	Channel Name:	254.0nm
Run Time:	60.0 Minutes	Proc. Chnl. Descr.:	2998 PDA 254.0 nm (2998
Date Acquired:	9/25/2024 7:46:17 PM CST		
Date Processed:	9/25/2024 7:59:01 PM CST		

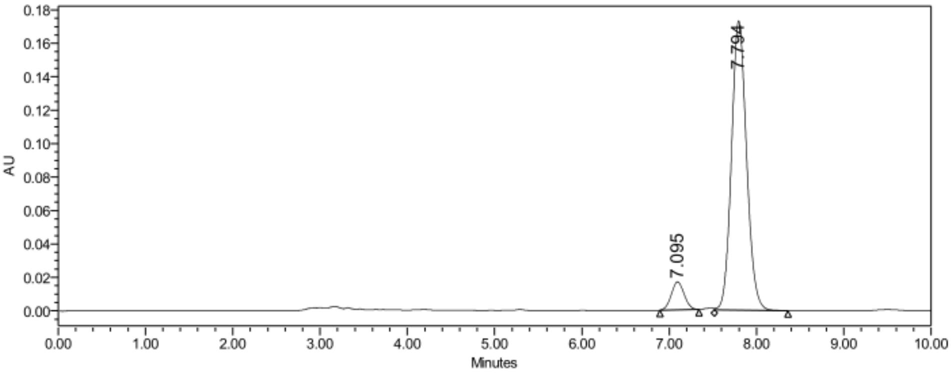

	RT	Area	% Area	Height
1	7.095	172198	7.66	16773
2	7.794	2076763	92.34	172975

Rac-**3s**

SAMPLE INFORMATION

Sample Name:	XT-8-48-1-rac-1%-IG	Acquired By:	System
Sample Type:	Unknown	Sample Set Name:	0105
Vial:	52	Acq. Method Set:	1% quanbo
Injection #:	1	Processing Method	XT 8 48 1 RAC RE
Injection Volume:	10.00 ul	Channel Name:	254.0nm
Run Time:	18.0 Minutes	Proc. Chnl. Descr.:	2998 PDA 254.0 nm (2998
Date Acquired:	1/5/2025 20:26:11 CST		
Date Processed:	1/5/2025 21:08:53 CST		

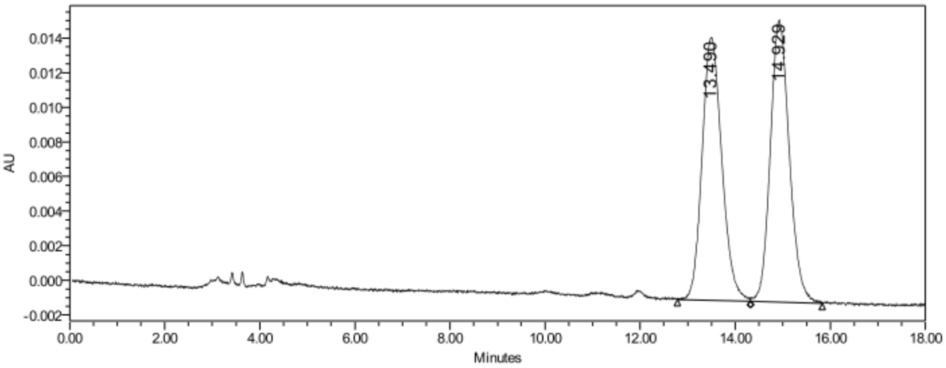

	RT	Area	% Area	Height
1	13.490	436826	49.29	15183
2	14.929	449398	50.71	16291

Asy-**3s**

SAMPLE INFORMATION

Sample Name:	XT-8-48-2-asy-1%-IG	Acquired By:	System
Sample Type:	Unknown	Sample Set Name:	0105
Vial:	53	Acq. Method Set:	1% quanbo
Injection #:	1	Processing Method	xt8 48 2 asy re
Injection Volume:	10.00 ul	Channel Name:	254.0nm
Run Time:	18.0 Minutes	Proc. Chnl. Descr.:	2998 PDA 254.0 nm (2998
Date Acquired:	1/5/2025 20:44:51 CST		
Date Processed:	1/5/2025 21:07:26 CST		

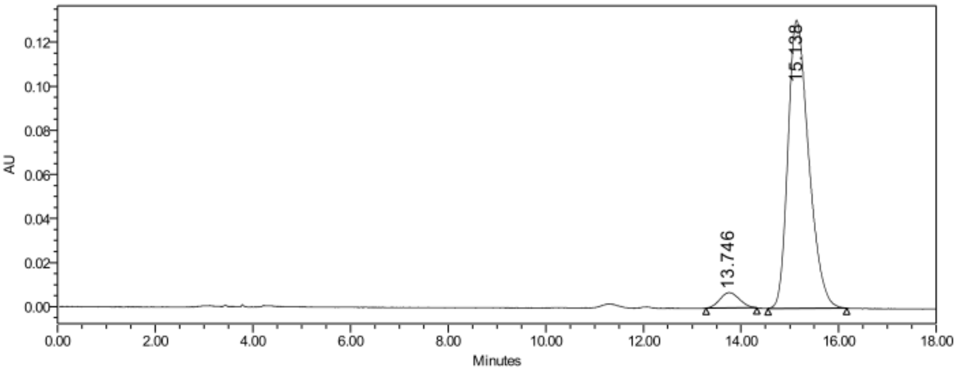

	RT	Area	% Area	Height
1	13.746	190365	4.70	6994
2	15.138	3862096	95.30	130716

Rac-**3t**

SAMPLE INFORMATION			
Sample Name:	xt-8-55-1-RAC-2%-IE	Acquired By:	System
Sample Type:	Unknown	Sample Set Name:	
Vial:	59	Acq. Method Set:	2%qb
Injection #:	1	Processing Method:	XT 8 55 1 RAC
Injection Volume:	10.00 ul	Channel Name:	254.0nm
Run Time:	60.0 Minutes	Proc. Chnl. Descr.:	2998 PDA 254.0 nm (2998
Date Acquired:	9/21/2024 3:42:33 PM CST		
Date Processed:	9/26/2024 5:13:05 PM CST		

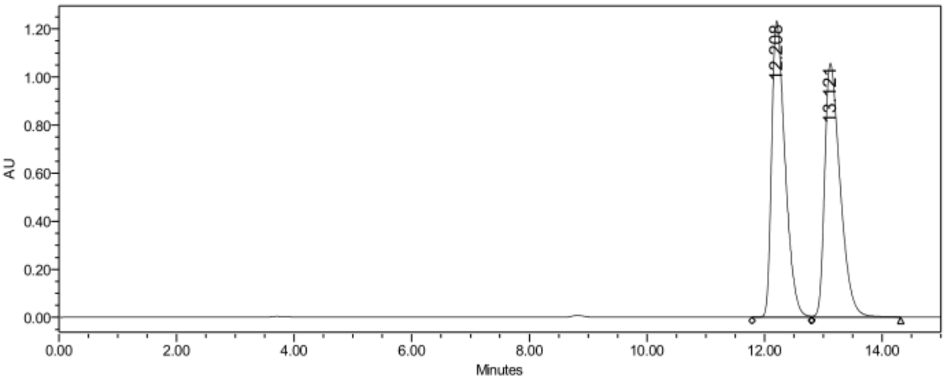

	RT	Area	% Area	Height
1	12.208	19509432	50.15	1232637
2	13.121	19390387	49.85	1055203

Asy-**3t**

SAMPLE INFORMATION			
Sample Name:	xt-8-55-2-ASY-2%-IE	Acquired By:	System
Sample Type:	Unknown	Sample Set Name:	
Vial:	79	Acq. Method Set:	2%qb
Injection #:	1	Processing Method:	XT 8 55 2 ASY
Injection Volume:	10.00 ul	Channel Name:	254.0nm
Run Time:	60.0 Minutes	Proc. Chnl. Descr.:	2998 PDA 254.0 nm (2998
Date Acquired:	9/21/2024 4:01:35 PM CST		
Date Processed:	9/26/2024 5:11:15 PM CST		

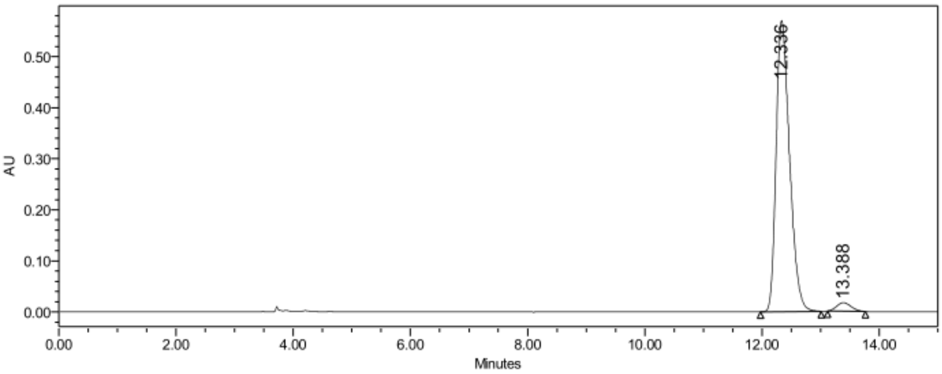

	RT	Area	% Area	Height
1	12.336	9027727	96.94	570404
2	13.388	285059	3.06	16638

Rac-**3w-1** (The product was isolated as a 6:1 mixture of E/Z isomers)

SAMPLE INFORMATION

Sample Name:	XT-8-92-1-RAC-2%-IC	Acquired By:	System
Sample Type:	Unknown	Sample Set Name	
Vial:	100	Acq. Method Set:	2% quanbo
Injection #:	1	Processing Method	XT 8 92 1 1 RAC
Injection Volume:	30.00 ul	Channel Name:	254.0nm
Run Time:	60.0 Minutes	Proc. Chnl. Descr.:	2998 PDA 254.0 nm (2998
Date Acquired:	10/17/2024 21:04:45 CST		
Date Processed:	10/17/2024 21:23:28 CST		

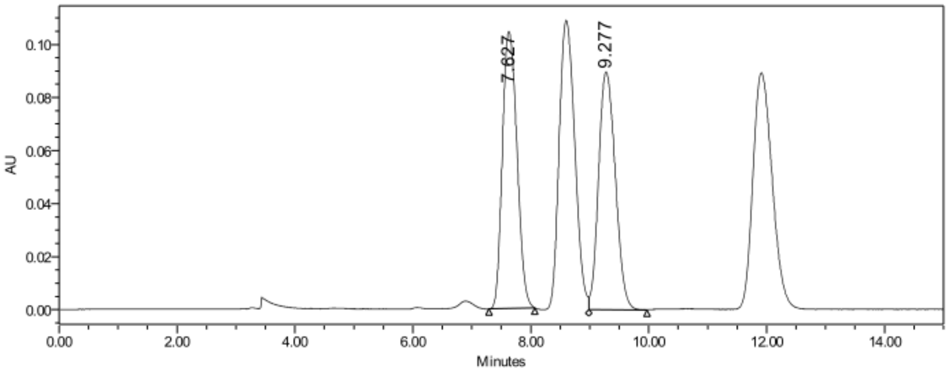

	RT	Area	% Area	Height
1	7.627	1784811	50.35	104441
2	9.277	1760182	49.65	89670

Asy-**3w-1**

SAMPLE INFORMATION

Sample Name:	XT-8-92-2-ASY-2%-IC	Acquired By:	System
Sample Type:	Unknown	Sample Set Name	
Vial:	68	Acq. Method Set:	2% quanbo
Injection #:	1	Processing Method	XT 8 92 1 1 ASY
Injection Volume:	30.00 ul	Channel Name:	254.0nm
Run Time:	60.0 Minutes	Proc. Chnl. Descr.:	2998 PDA 254.0 nm (2998
Date Acquired:	10/17/2024 19:20:00 CST		
Date Processed:	10/17/2024 21:26:21 CST		

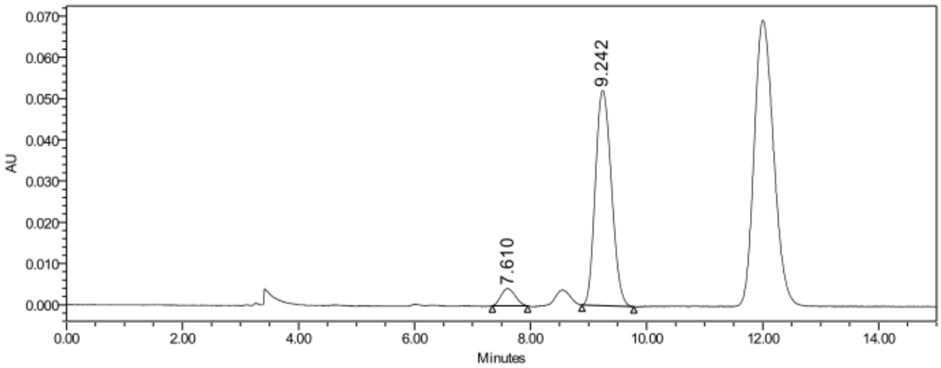

	RT	Area	% Area	Height
1	7.610	71554	6.66	4216
2	9.242	1002681	93.34	52154

Rac-**3w-2** (The product was isolated as a 6:1 mixture of E/Z isomers)

SAMPLE INFORMATION

Sample Name:	XT-8-92-1-RAC-2%-IC	Acquired By:	System
Sample Type:	Unknown	Sample Set Name	
Vial:	100	Acq. Method Set:	2% quanbo
Injection #:	1	Processing Method	XT 8 92 1 2 RAC
Injection Volume:	30.00 ul	Channel Name:	254.0nm
Run Time:	60.0 Minutes	Proc. Chnl. Descr.:	2998 PDA 254.0 nm (2998
Date Acquired:	10/17/2024 21:04:45 CST		
Date Processed:	10/17/2024 21:25:05 CST		

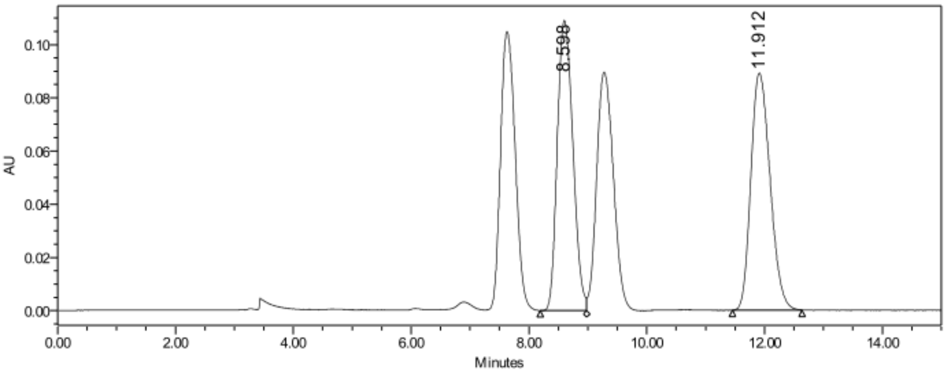

	RT	Area	% Area	Height
1	8.598	2005514	49.89	109062
2	11.912	2014248	50.11	89142

Asy-**3w-2**

SAMPLE INFORMATION

Sample Name:	XT-8-92-2-ASY-2%-IC	Acquired By:	System
Sample Type:	Unknown	Sample Set Name	
Vial:	68	Acq. Method Set:	2% quanbo
Injection #:	1	Processing Method	XT 8 92 1 2 ASY
Injection Volume:	30.00 ul	Channel Name:	254.0nm
Run Time:	60.0 Minutes	Proc. Chnl. Descr.:	2998 PDA 254.0 nm (2998
Date Acquired:	10/17/2024 19:20:00 CST		
Date Processed:	10/17/2024 21:27:30 CST		

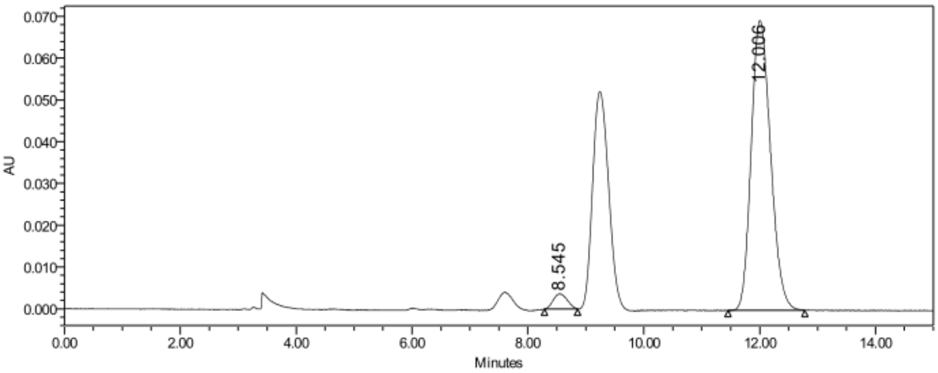

	RT	Area	% Area	Height
1	8.545	61525	3.68	3655
2	12.006	1611136	96.32	69357

Rac-**3x**

SAMPLE INFORMATION

Sample Name:	xt-7-119-3-5%-rac-IG	Acquired By:	System
Sample Type:	Unknown	Sample Set Name	0626
Vial:	44	Acq. Method Set:	5% quanbo
Injection #:	1	Processing Method	XT 7 119 3 RAC
Injection Volume:	10.00 ul	Channel Name:	254.0nm
Run Time:	9.0 Minutes	Proc. Chnl. Descr.:	2998 PDA 254.0 nm (2998
Date Acquired:	6/26/2024 15:52:07 CST		
Date Processed:	6/26/2024 16:03:55 CST		

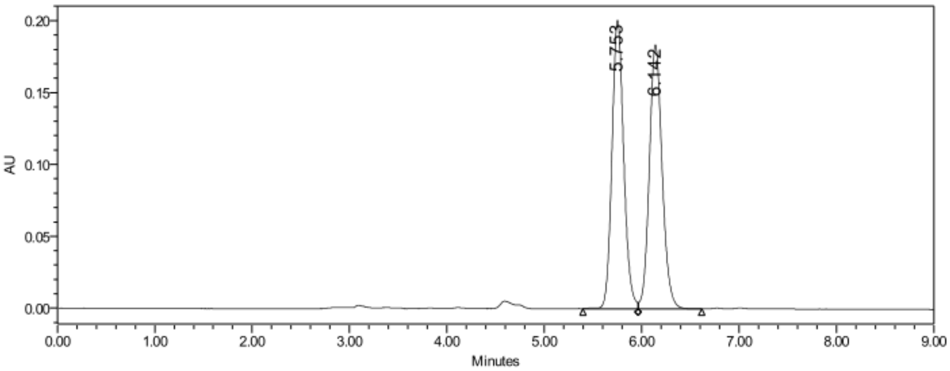

	RT	Area	% Area	Height
1	5.753	1686546	49.96	200522
2	6.142	1689185	50.04	183681

Asy-**3x**

SAMPLE INFORMATION

Sample Name:	xt-7-133-2-5%-asy-IG	Acquired By:	System
Sample Type:	Unknown	Sample Set Name	0626
Vial:	43	Acq. Method Set:	5% quanbo
Injection #:	1	Processing Method	XT 7 133 2 ASY
Injection Volume:	10.00 ul	Channel Name:	254.0nm
Run Time:	9.0 Minutes	Proc. Chnl. Descr.:	2998 PDA 254.0 nm (2998
Date Acquired:	6/26/2024 15:42:27 CST		
Date Processed:	6/26/2024 16:06:32 CST		

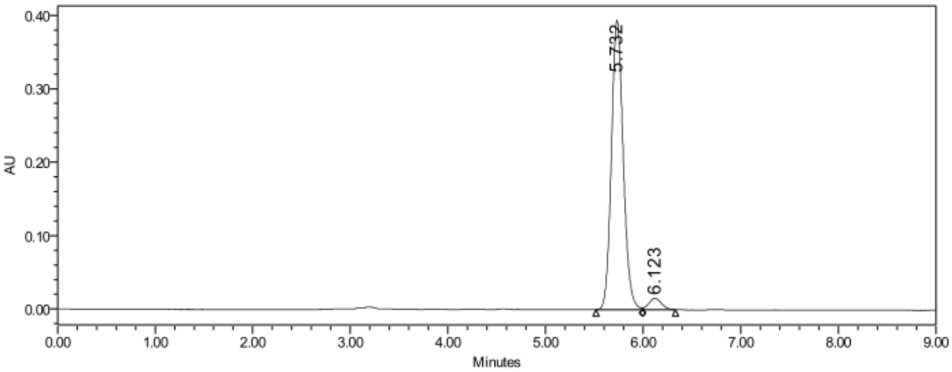

	RT	Area	% Area	Height
1	5.732	3315494	95.97	394162
2	6.123	139336	4.03	15413

Rac-**3y'**

SAMPLE INFORMATION

Sample Name:	xt-8-86-1-RAC-20%-IG	Acquired By:	System
Sample Type:	Unknown	Sample Set Name:	
Vial:	83	Acq. Method Set:	20%qb
Injection #:	1	Processing Method:	XT 8 86 1 RAC
Injection Volume:	30.00 ul	Channel Name:	254.0nm
Run Time:	60.0 Minutes	Proc. Chnl. Descr.:	2998 PDA 254.0 nm (2998
Date Acquired:	10/19/2024 8:22:50 PM CST		
Date Processed:	10/19/2024 9:11:00 PM CST		

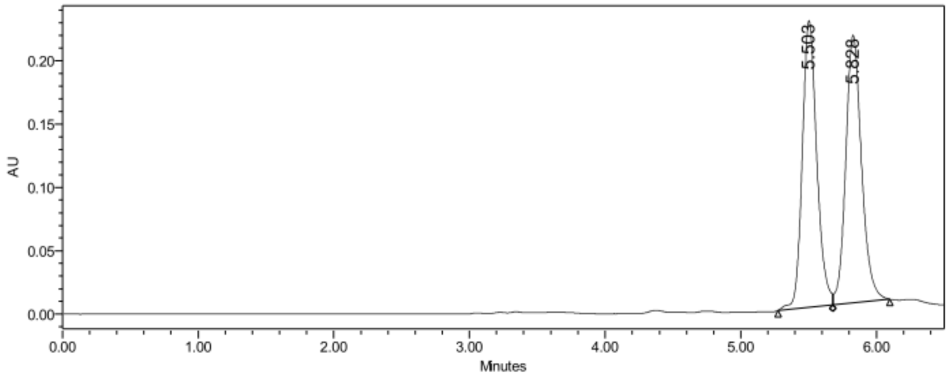

	RT	Area	% Area	Height
1	5.503	1682440	49.58	226380
2	5.828	1711060	50.42	211399

Asy-**3y'**

SAMPLE INFORMATION

Sample Name:	xt-8-86-3-20%-IG	Acquired By:	System
Sample Type:	Unknown	Sample Set Name:	
Vial:	82	Acq. Method Set:	20%qb
Injection #:	1	Processing Method:	XT 8 86 2 ASY
Injection Volume:	20.00 ul	Channel Name:	254.0nm
Run Time:	60.0 Minutes	Proc. Chnl. Descr.:	2998 PDA 254.0 nm (2998
Date Acquired:	10/19/2024 7:59:11 PM CST		
Date Processed:	10/19/2024 9:12:12 PM CST		

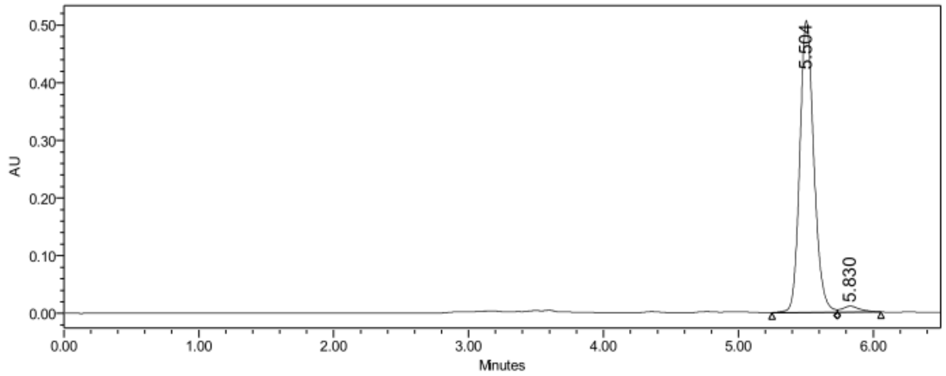

	RT	Area	% Area	Height
1	5.504	3677596	97.47	506690
2	5.830	95565	2.53	10409

Rac-**3z**

SAMPLE INFORMATION

Sample Name:	XT-8-115-1-rac-2%0.5flow-IG	Acquired By:	System
Sample Type:	Unknown	Sample Set Name	
Vial:	74	Acq. Method Set:	2% quanbo05 flow
Injection #:	1	Processing Method	XT 8 115 1 RAC
Injection Volume:	10.00 ul	Channel Name:	254.0nm
Run Time:	60.0 Minutes	Proc. Chnl. Descr.:	2998 PDA 254.0 nm (2998
Date Acquired:	10/31/2024 19:57:03 CST		
Date Processed:	10/31/2024 20:16:45 CST		

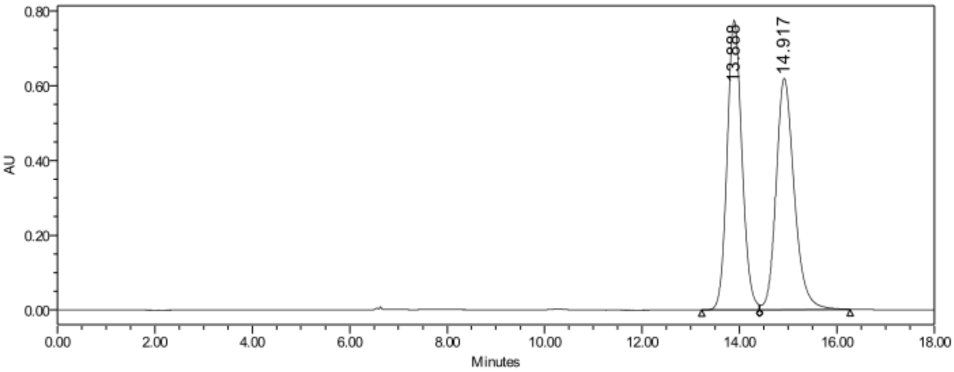

	RT	Area	% Area	Height
1	13.888	16453662	50.55	775236
2	14.917	16093005	49.45	618922

Asy-**3z**

SAMPLE INFORMATION

Sample Name:	XT-8-115-2-asy-2%0.5flow-IG	Acquired By:	System
Sample Type:	Unknown	Sample Set Name	
Vial:	75	Acq. Method Set:	2% quanbo05 flow
Injection #:	1	Processing Method	XT 8 115 2 ASY
Injection Volume:	20.00 ul	Channel Name:	254.0nm
Run Time:	60.0 Minutes	Proc. Chnl. Descr.:	2998 PDA 254.0 nm (2998
Date Acquired:	10/31/2024 19:37:16 CST		
Date Processed:	10/31/2024 20:14:58 CST		

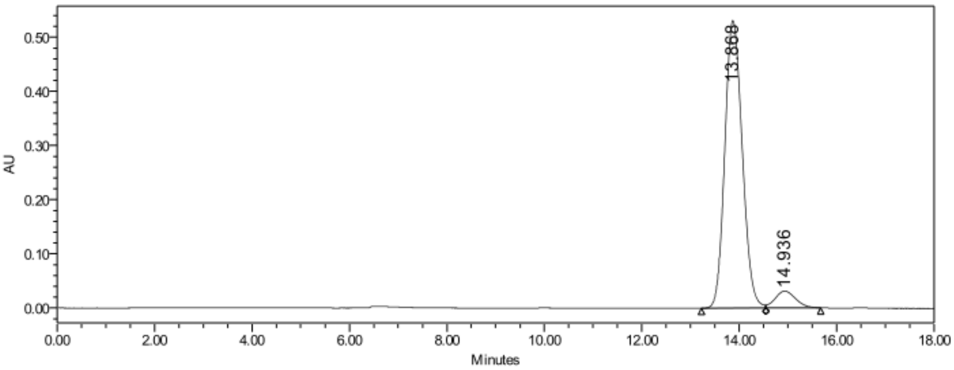

	RT	Area	% Area	Height
1	13.868	13297795	93.59	531126
2	14.936	910625	6.41	31147

Rac-**3aa**

SAMPLE INFORMATION

Sample Name:	XT-8-81-1-rac-2%-IE	Acquired By:	System
Sample Type:	Unknown	Sample Set Name	1012
Vial:	2	Acq. Method Set:	2% quanbo
Injection #:	1	Processing Method	XT 8 81 1 RAC
Injection Volume:	10.00 ul	Channel Name:	254.0nm
Run Time:	20.0 Minutes	Proc. Chnl. Descr.:	2998 PDA 254.0 nm (2998
Date Acquired:	10/12/2024 11:03:37 CST		
Date Processed:	10/14/2024 15:35:10 CST		

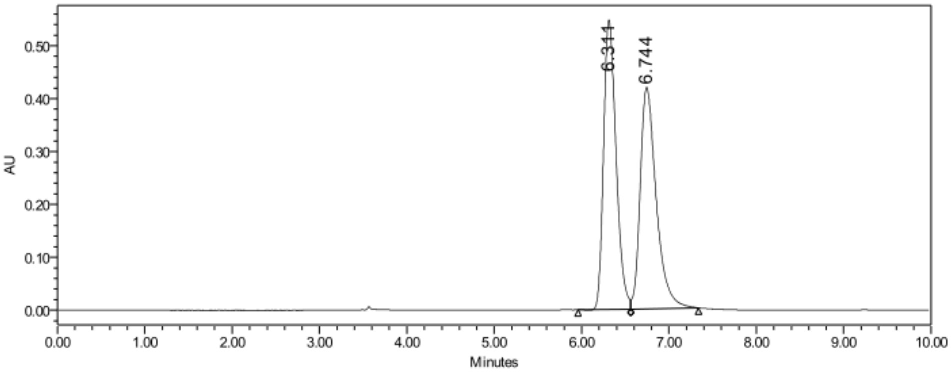

	RT	Area	% Area	Height
1	6.311	5392035	50.25	546935
2	6.744	5337811	49.75	418180

Asy-**3aa**

SAMPLE INFORMATION

Sample Name:	XT-8-81-3-ASY-2%-IE	Acquired By:	System
Sample Type:	Unknown	Sample Set Name	
Vial:	6	Acq. Method Set:	2% quanbo
Injection #:	1	Processing Method	XT 8 81 3 re ASY
Injection Volume:	20.00 ul	Channel Name:	254.0nm
Run Time:	60.0 Minutes	Proc. Chnl. Descr.:	2998 PDA 254.0 nm (2998
Date Acquired:	10/14/2024 15:19:47 CST		
Date Processed:	2/25/2025 19:01:03 CST		

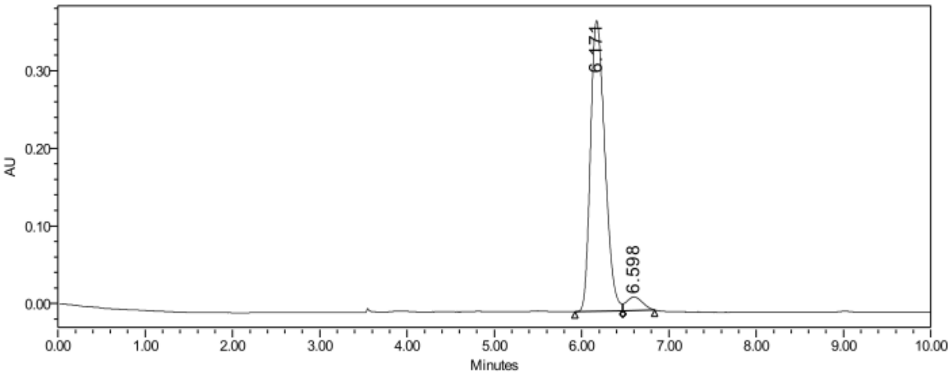

	RT	Area	% Area	Height
1	6.171	4282421	95.08	374581
2	6.598	221790	4.92	17324

Rac-**3ab**

SAMPLE INFORMATION

Sample Name:	XT-8-75-1-RAC-1%-IG	Acquired By:	System
Sample Type:	Unknown	Sample Set Name	
Vial:	92	Acq. Method Set:	1% quanbo
Injection #:	1	Processing Method	XT 8 75 1 RAC
Injection Volume:	10.00 ul	Channel Name:	254.0nm
Run Time:	60.0 Minutes	Proc. Chnl. Descr.:	2998 PDA 254.0 nm (2998
Date Acquired:	10/10/2024 10:56:28 CST		
Date Processed:	10/10/2024 16:02:39 CST		

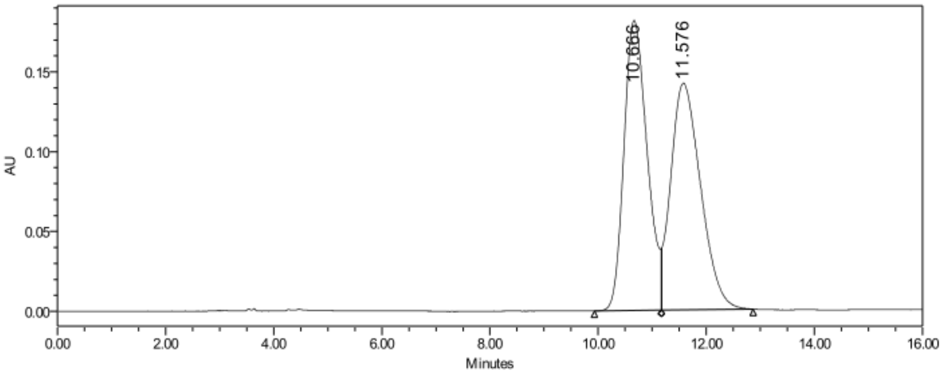

	RT	Area	% Area	Height
1	10.666	5430731	49.82	181712
2	11.576	5470348	50.18	142099

Asy-**3ab**

SAMPLE INFORMATION

Sample Name:	XT-8-77-1-ASY-1%-IG	Acquired By:	System
Sample Type:	Unknown	Sample Set Name	1010
Vial:	116	Acq. Method Set:	1% quanbo
Injection #:	1	Processing Method	XT 8 77 1 asy
Injection Volume:	10.00 ul	Channel Name:	254.0nm@1
Run Time:	16.0 Minutes	Proc. Chnl. Descr.:	2998 PDA 254.0 nm (2998
Date Acquired:	10/10/2024 11:17:21 CST		
Date Processed:	10/10/2024 16:00:53 CST		

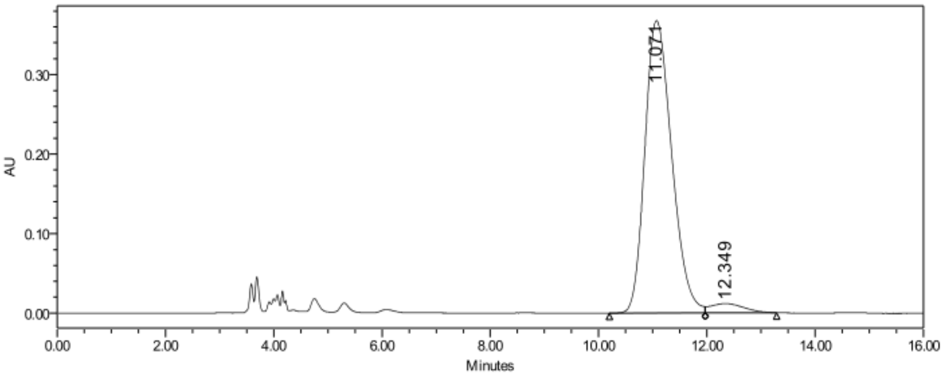

	RT	Area	% Area	Height
1	11.071	12646665	96.19	367809
2	12.349	501317	3.81	11719

Rac-**3ac**

SAMPLE INFORMATION

Sample Name:	XT-8-89-2-rac-8%-IG	Acquired By:	System
Sample Type:	Unknown	Sample Set Name	
Vial:	73	Acq. Method Set:	8% quanbo
Injection #:	1	Processing Method	XT 8 89 2 RAC
Injection Volume:	10.00 ul	Channel Name:	241.0nm
Run Time:	60.0 Minutes	Proc. Chnl. Descr.:	2998 PDA 241.0 nm (2998
Date Acquired:	10/22/2024 20:07:59 CST		
Date Processed:	10/22/2024 20:41:56 CST		

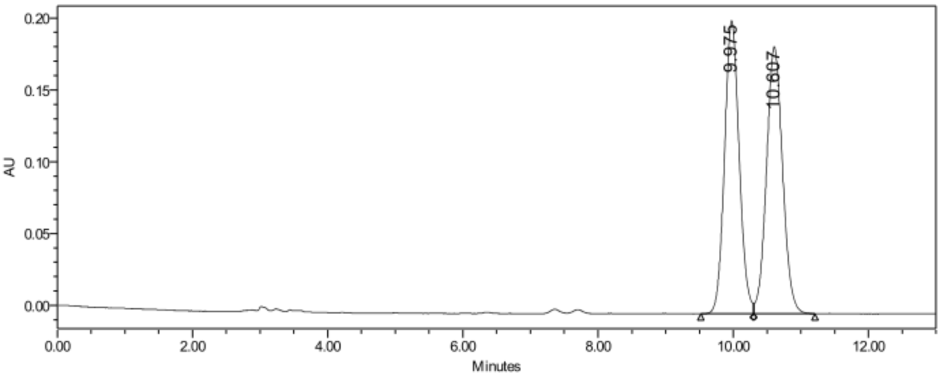

	RT	Area	% Area	Height
1	9.975	3086594	50.12	204132
2	10.607	3071331	49.88	186046

Asy-**3ac**

SAMPLE INFORMATION

Sample Name:	XT-8-101-1-asy-8%-IG	Acquired By:	System
Sample Type:	Unknown	Sample Set Name	
Vial:	117	Acq. Method Set:	8% quanbo
Injection #:	1	Processing Method	XT 8 101 1 ASY
Injection Volume:	30.00 ul	Channel Name:	241.0nm
Run Time:	60.0 Minutes	Proc. Chnl. Descr.:	2998 PDA 241.0 nm (2998
Date Acquired:	10/22/2024 20:24:27 CST		
Date Processed:	10/22/2024 20:40:15 CST		

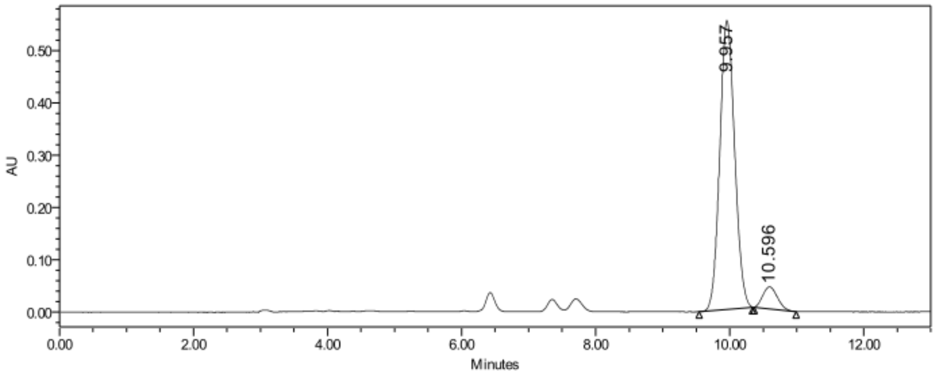

	RT	Area	% Area	Height
1	9.957	8546701	93.12	552156
2	10.596	631545	6.88	41804

Rac-**3ad**

SAMPLE INFORMATION

Sample Name:	xt-8-101-2-RAC-5%-IG	Acquired By:	System
Sample Type:	Unknown	Sample Set Name:	
Vial:	94	Acq. Method Set:	5%qb
Injection #:	1	Processing Method:	xt 8 101 2 rac
Injection Volume:	10.00 ul	Channel Name:	254.0nm
Run Time:	60.0 Minutes	Proc. Chnl. Descr.:	2998 PDA 254.0 nm (2998
Date Acquired:	10/31/2024 9:41:01 AM CST		
Date Processed:	10/31/2024 9:56:10 AM CST		

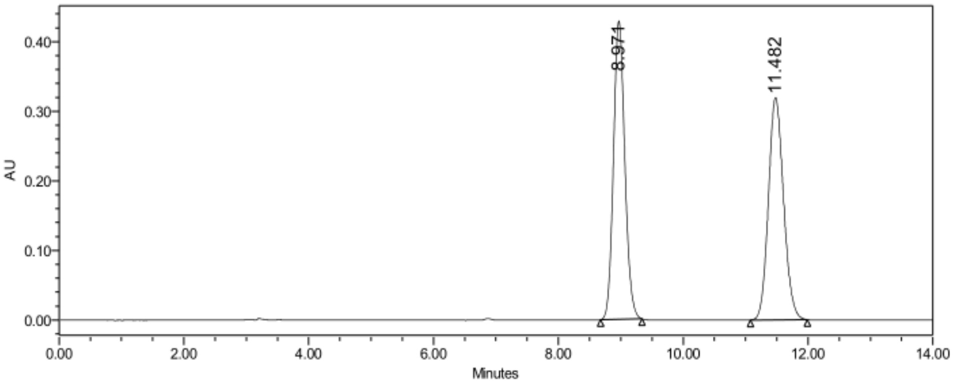

	RT	Area	% Area	Height
1	8.971	5425851	50.25	429233
2	11.482	5372242	49.75	319556

Asy-**3ad**

SAMPLE INFORMATION

Sample Name:	xt-8-101-3-5%-IG	Acquired By:	System
Sample Type:	Unknown	Sample Set Name:	
Vial:	60	Acq. Method Set:	5%qb
Injection #:	1	Processing Method:	XT 8 101 3 ASY
Injection Volume:	20.00 ul	Channel Name:	254.0nm
Run Time:	60.0 Minutes	Proc. Chnl. Descr.:	2998 PDA 254.0 nm (2998
Date Acquired:	10/31/2024 9:20:41 AM CST		
Date Processed:	10/31/2024 9:51:27 AM CST		

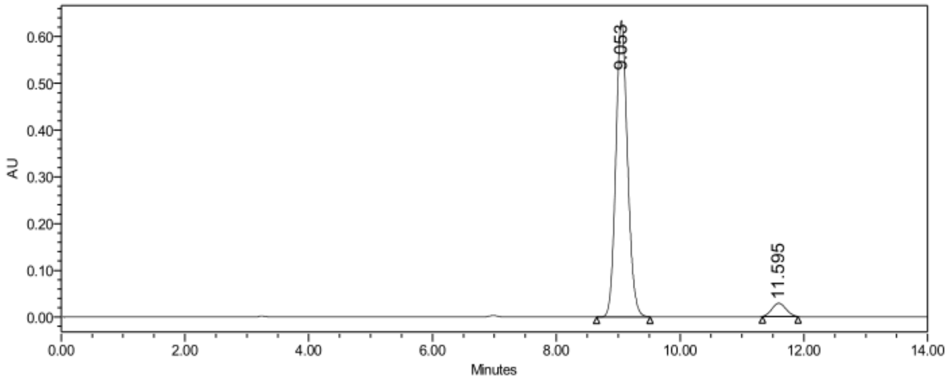

	RT	Area	% Area	Height
1	9.053	8308994	94.93	634192
2	11.595	443967	5.07	28191

Rac-**3ae**

SAMPLE INFORMATION			
Sample Name:	xt-8-102-1-2%05flow-AD	Acquired By:	System
Sample Type:	Unknown	Sample Set Name:	
Vial:	59	Acq. Method Set:	2%qb 05fiow
Injection #:	1	Processing Method:	xt 8 102 1 rac
Injection Volume:	10.00 ul	Channel Name:	254.0nm
Run Time:	60.0 Minutes	Proc. Chnl. Descr.:	2998 PDA 254.0 nm (2998
Date Acquired:	10/25/2024 8:44:14 PM CST		
Date Processed:	10/28/2024 5:15:59 PM CST		

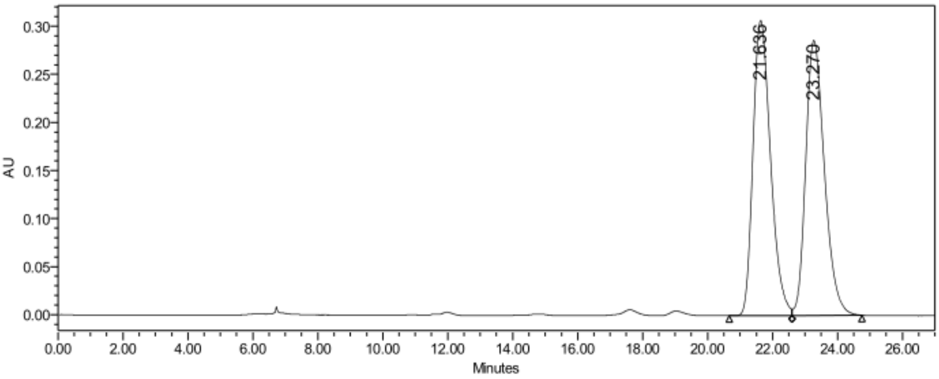

	RT	Area	% Area	Height
1	21.636	11571722	50.11	306936
2	23.270	11522094	49.89	285997

Asy-**3ae**

SAMPLE INFORMATION			
Sample Name:	xt-8-108-3-2%0.5flow-IG	Acquired By:	System
Sample Type:	Unknown	Sample Set Name:	
Vial:	10	Acq. Method Set:	2%qb 05fiow
Injection #:	1	Processing Method:	xt 8 108 3 asy
Injection Volume:	10.00 ul	Channel Name:	254.0nm
Run Time:	60.0 Minutes	Proc. Chnl. Descr.:	2998 PDA 254.0 nm (2998
Date Acquired:	10/27/2024 6:24:14 PM CST		
Date Processed:	10/28/2024 5:17:37 PM CST		

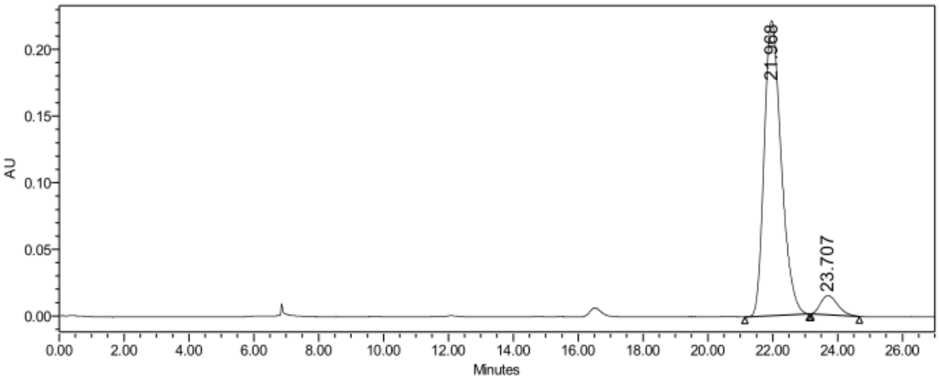

	RT	Area	% Area	Height
1	21.968	8088378	93.92	221367
2	23.707	523862	6.08	14380

Rac-**3af**

SAMPLE INFORMATION

Sample Name:	xt-8-89-1-RAC-30%-IG	Acquired By:	System
Sample Type:	Unknown	Sample Set Name:	
Vial:	76	Acq. Method Set:	30%qb
Injection #:	1	Processing Method:	xt 8 89 1 rac
Injection Volume:	10.00 ul	Channel Name:	254.0nm
Run Time:	60.0 Minutes	Proc. Chnl. Descr.:	2998 PDA 254.0 nm (2998
Date Acquired:	10/27/2024 5:29:44 PM CST		
Date Processed:	10/27/2024 5:45:54 PM CST		

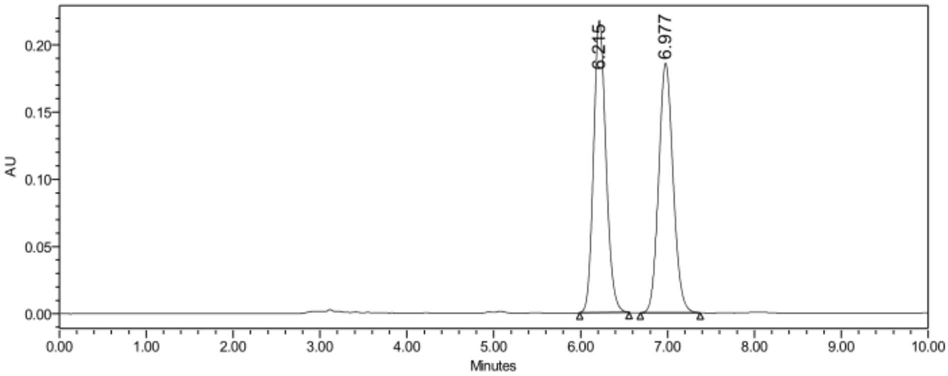

	RT	Area	% Area	Height
1	6.215	2167211	50.17	217445
2	6.977	2152463	49.83	185768

Asy-**3af**

SAMPLE INFORMATION

Sample Name:	xt-8-108-4-30%-IG	Acquired By:	System
Sample Type:	Unknown	Sample Set Name:	
Vial:	50	Acq. Method Set:	30%qb
Injection #:	1	Processing Method:	XT 8 108 4 ASY
Injection Volume:	20.00 ul	Channel Name:	254.0nm
Run Time:	60.0 Minutes	Proc. Chnl. Descr.:	2998 PDA 254.0 nm (2998
Date Acquired:	10/27/2024 5:13:10 PM CST		
Date Processed:	10/27/2024 5:48:04 PM CST		

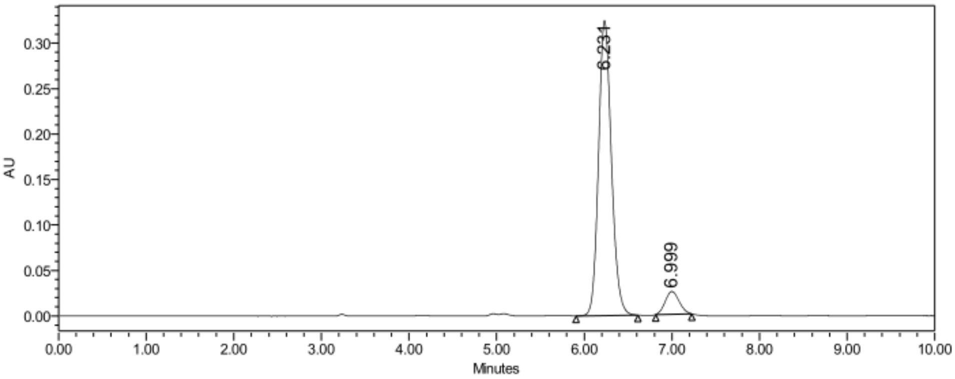

	RT	Area	% Area	Height
1	6.231	3322369	92.26	324508
2	6.999	278817	7.74	25121

Rac-**3ag**

SAMPLE INFORMATION

Sample Name:	xt-8-149-1-rac-2%-IE	Acquired By:	System
Sample Type:	Unknown	Sample Set Name	1130
Vial:	16	Acq. Method Set:	2% quanbo
Injection #:	1	Processing Method	XT 8 149 1 RAC
Injection Volume:	30.00 ul	Channel Name:	254.0nm
Run Time:	10.0 Minutes	Proc. Chnl. Descr.:	2998 PDA 254.0 nm (2998
Date Acquired:	11/30/2024 11:56:28 CST		
Date Processed:	11/30/2024 14:22:43 CST		

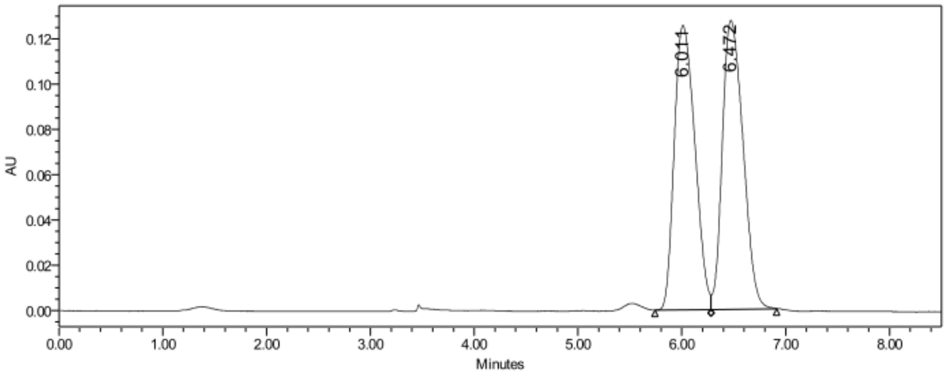

	RT	Area	% Area	Height
1	6.011	1737036	49.51	125655
2	6.472	1771150	50.49	127460

Asy-**3ag**

SAMPLE INFORMATION

Sample Name:	xt-8-149-2-asy-2%-IE	Acquired By:	System
Sample Type:	Unknown	Sample Set Name	1130
Vial:	15	Acq. Method Set:	2% quanbo
Injection #:	1	Processing Method	XT 8 149 2 ASY
Injection Volume:	30.00 ul	Channel Name:	254.0nm
Run Time:	10.0 Minutes	Proc. Chnl. Descr.:	2998 PDA 254.0 nm (2998
Date Acquired:	11/30/2024 11:45:40 CST		
Date Processed:	11/30/2024 14:20:57 CST		

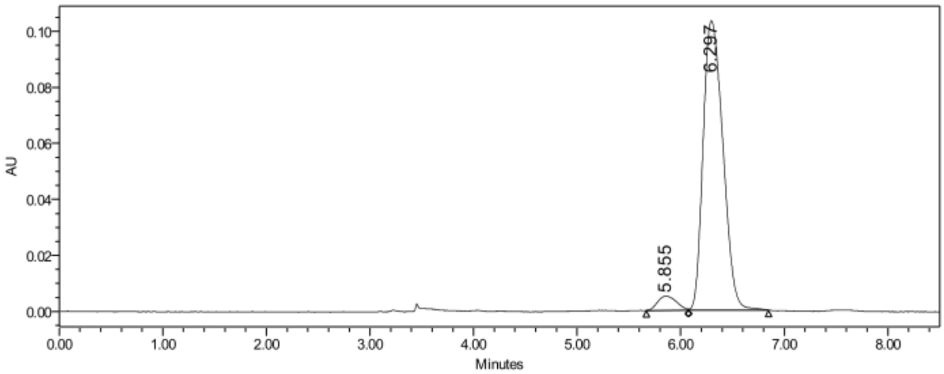

	RT	Area	% Area	Height
1	5.855	64785	4.51	5101
2	6.297	1372642	95.49	103401

Rac-**3ah**

SAMPLE INFORMATION

Sample Name:	xt-8-160-3-rac-2%0.5flow-IG	Acquired By:	System
Sample Type:	Unknown	Sample Set Name:	1217
Vial:	76	Acq. Method Set:	2%qb 05fiow
Injection #:	1	Processing Method:	XT 8 160 3 RAC
Injection Volume:	10.00 ul	Channel Name:	254.0nm
Run Time:	20.0 Minutes	Proc. Chnl. Descr.:	2998 PDA 254.0 nm (2998
Date Acquired:	12/17/2024 5:17:56 PM CST		
Date Processed:	12/17/2024 7:10:43 PM CST		

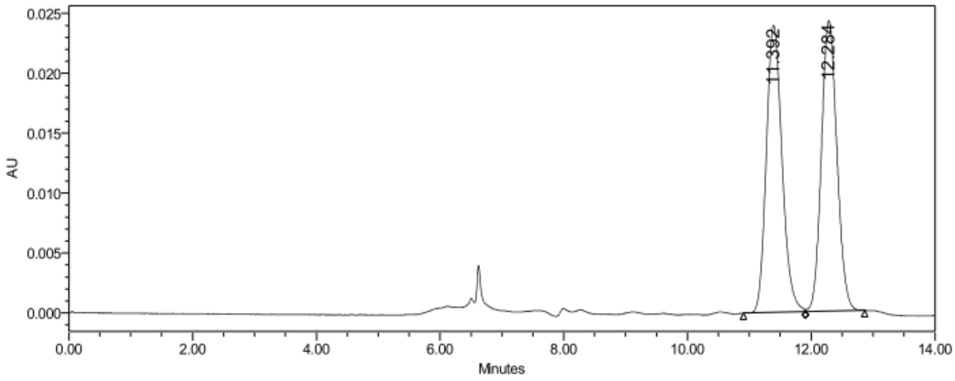

	RT	Area	% Area	Height
1	11.392	422125	49.83	23968
2	12.284	425051	50.17	24257

Asy-**3ah**

SAMPLE INFORMATION

Sample Name:	xt-8-160-2-2%0.5flow-IG	Acquired By:	System
Sample Type:	Unknown	Sample Set Name:	
Vial:	105	Acq. Method Set:	2%qb 05fiow
Injection #:	1	Processing Method:	XT 8 160 2 ASY
Injection Volume:	30.00 ul	Channel Name:	254.0nm
Run Time:	60.0 Minutes	Proc. Chnl. Descr.:	2998 PDA 254.0 nm (2998
Date Acquired:	12/15/2024 7:13:06 PM CST		
Date Processed:	12/15/2024 7:37:04 PM CST		

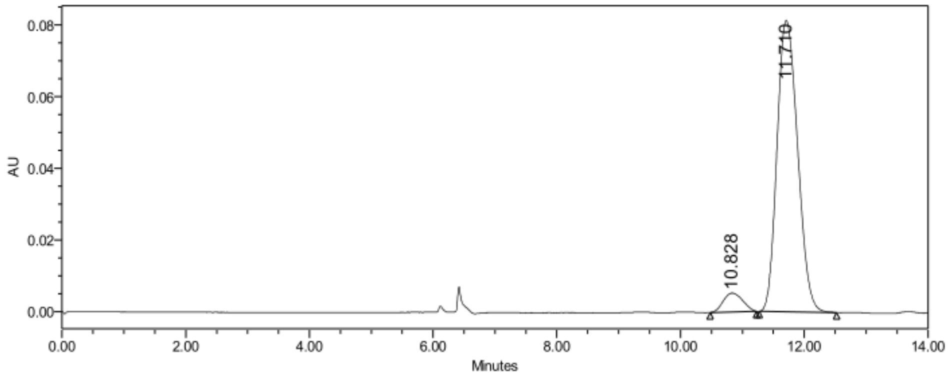

	RT	Area	% Area	Height
1	10.828	114610	5.71	5273
2	11.710	1891630	94.29	81411

Rac-**3ai**

SAMPLE INFORMATION

Sample Name:	xt-8-75-2-RAC-2%-IG	Acquired By:	System
Sample Type:	Unknown	Sample Set Name:	
Vial:	100	Acq. Method Set:	2%qb
Injection #:	1	Processing Method:	xt 8 75 2 rac
Injection Volume:	10.00 ul	Channel Name:	254.0nm
Run Time:	60.0 Minutes	Proc. Chnl. Descr.:	2998 PDA 254.0 nm (2998
Date Acquired:	10/27/2024 5:54:04 PM CST		
Date Processed:	10/27/2024 6:12:02 PM CST		

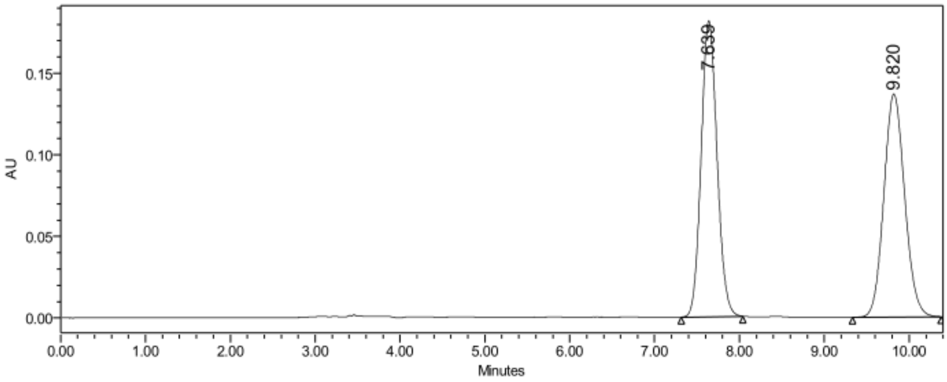

	RT	Area	% Area	Height
1	7.639	2354998	50.17	181645
2	9.820	2339355	49.83	136835

Asy-**3ai**

SAMPLE INFORMATION

Sample Name:	XT-8-178-1-2%-IG	Acquired By:	System
Sample Type:	Unknown	Sample Set Name:	
Vial:	64	Acq. Method Set:	2% quanbo
Injection #:	1	Processing Method	XT 8 178 1 ASY
Injection Volume:	30.00 ul	Channel Name:	254.0nm
Run Time:	60.0 Minutes	Proc. Chnl. Descr.:	2998 PDA 254.0 nm (2998
Date Acquired:	12/31/2024 19:20:05 CST		
Date Processed:	2/22/2025 20:51:28 CST		

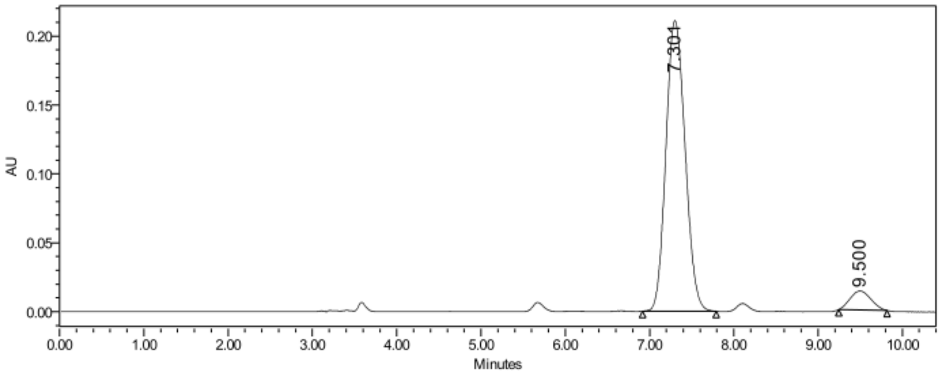

	RT	Area	% Area	Height
1	7.301	3348110	93.38	211064
2	9.500	237527	6.62	13804

Rac-**3aj**

SAMPLE INFORMATION			
Sample Name:	xt-8-65-1-rac-2%-IG	Acquired By:	System
Sample Type:	Unknown	Sample Set Name:	
Vial:	45	Acq. Method Set:	2%qb
Injection #:	1	Processing Method:	XT 8 65 1 RAC
Injection Volume:	10.00 ul	Channel Name:	254.0nm
Run Time:	60.0 Minutes	Proc. Chnl. Descr.:	2998 PDA 254.0 nm (2998
Date Acquired:	10/30/2024 3:15:44 PM CST		
Date Processed:	10/30/2024 4:31:58 PM CST		

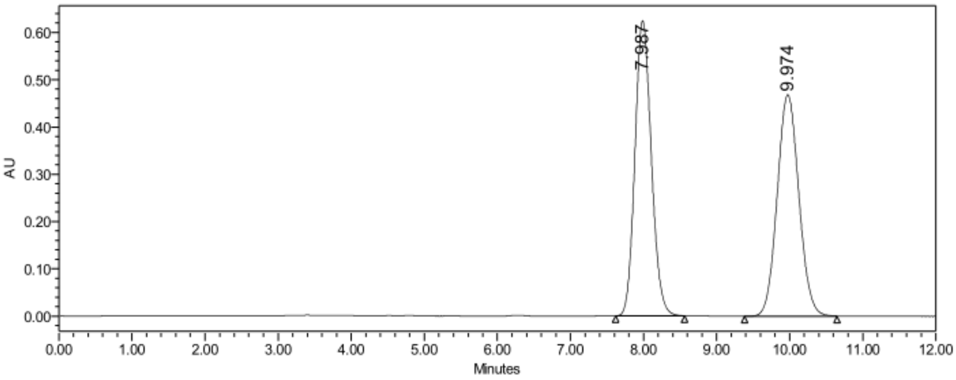

	RT	Area	% Area	Height
1	7.987	9686265	50.19	624774
2	9.974	9611461	49.81	468830

Asy-**3aj**

SAMPLE INFORMATION			
Sample Name:	XT-8-174-1-ASY-2%-IG	Acquired By:	System
Sample Type:	Unknown	Sample Set Name	0104
Vial:	82	Acq. Method Set:	2% quanbo
Injection #:	1	Processing Method	XT 8 174 1 ASY
Injection Volume:	30.00 ul	Channel Name:	254.0nm
Run Time:	14.0 Minutes	Proc. Chnl. Descr.:	2998 PDA 254.0 nm (2998
Date Acquired:	1/4/2025 17:39:13 CST		
Date Processed:	2/25/2025 18:53:49 CST		

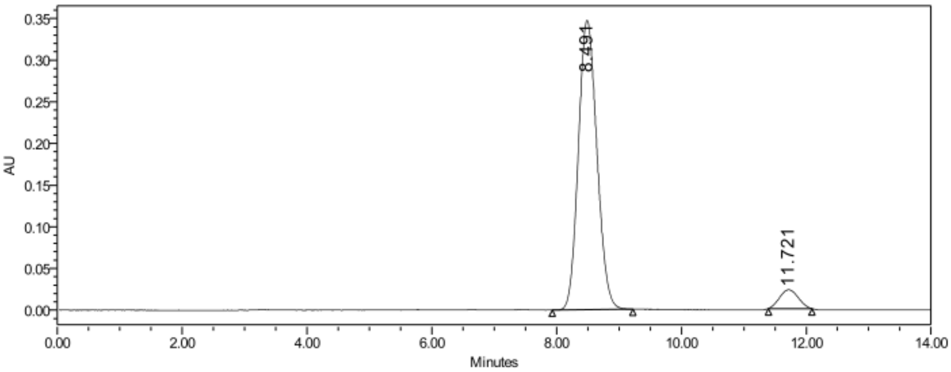

	RT	Area	% Area	Height
1	8.491	7144239	93.81	347263
2	11.721	471153	6.19	22775

Rac-**3ak**

SAMPLE INFORMATION

Sample Name:	xt-8-176-3-RAC-2%-IG	Acquired By:	System
Sample Type:	Unknown	Sample Set Name:	
Vial:	110	Acq. Method Set:	2%qb
Injection #:	1	Processing Method:	xt 8 176 3 rac
Injection Volume:	10.00 ul	Channel Name:	254.0nm
Run Time:	60.0 Minutes	Proc. Chnl. Descr.:	2998 PDA 254.0 nm (2998
Date Acquired:	1/3/2025 7:38:14 PM CST		
Date Processed:	2/22/2025 4:26:59 PM CST		

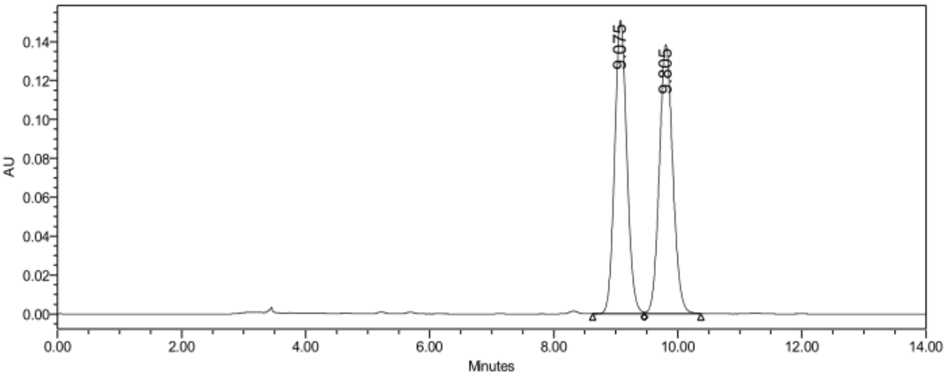

	RT	Area	% Area	Height
1	9.075	2072634	49.92	150934
2	9.805	2079012	50.08	138271

Asy-**3ak**

SAMPLE INFORMATION

Sample Name:	xt-8-177-1-ASY-2%-IG	Acquired By:	System
Sample Type:	Unknown	Sample Set Name:	
Vial:	7	Acq. Method Set:	2%qb
Injection #:	1	Processing Method:	xt 8 177 1 asy
Injection Volume:	10.00 ul	Channel Name:	254.0nm
Run Time:	60.0 Minutes	Proc. Chnl. Descr.:	2998 PDA 254.0 nm (2998
Date Acquired:	1/3/2025 7:54:04 PM CST		
Date Processed:	2/22/2025 4:25:12 PM CST		

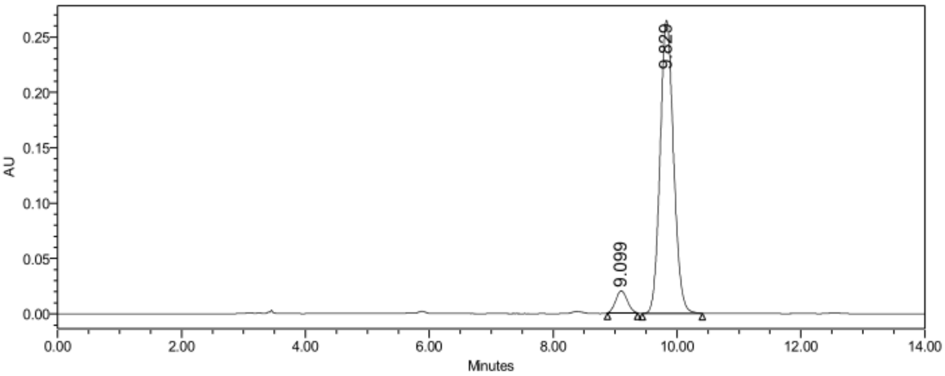

	RT	Area	% Area	Height
1	9.099	256767	6.05	19836
2	9.829	3984403	93.95	264629

Rac-**4a**

SAMPLE INFORMATION

Sample Name:	XT-8-79-1-rac-2%-IG	Acquired By:	System
Sample Type:	Unknown	Sample Set Name	
Vial:	31	Acq. Method Set:	2% quanbo
Injection #:	1	Processing Method	XT 8 79 1 RAC
Injection Volume:	10.00 ul	Channel Name:	254.0nm
Run Time:	60.0 Minutes	Proc. Chnl. Descr.:	2998 PDA 254.0 nm (2998
Date Acquired:	10/10/2024 15:37:46 CST		
Date Processed:	10/10/2024 19:15:12 CST		

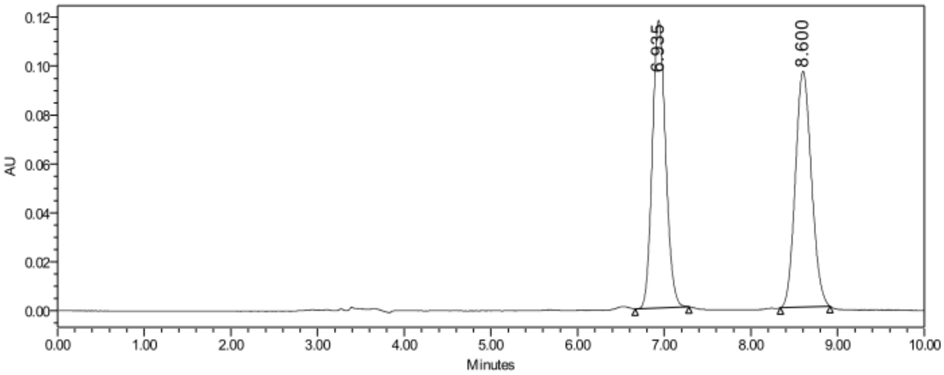

	RT	Area	% Area	Height
1	6.935	1237971	49.98	117556
2	8.600	1239164	50.02	96438

Asy-**4a**

SAMPLE INFORMATION

Sample Name:	XT-8-79-2-asy-2%-IG	Acquired By:	System
Sample Type:	Unknown	Sample Set Name	
Vial:	57	Acq. Method Set:	2% quanbo
Injection #:	1	Processing Method	XT 8 79 2 ASY
Injection Volume:	10.00 ul	Channel Name:	254.0nm
Run Time:	60.0 Minutes	Proc. Chnl. Descr.:	2998 PDA 254.0 nm (2998
Date Acquired:	10/10/2024 15:52:36 CST		
Date Processed:	10/10/2024 19:13:41 CST		

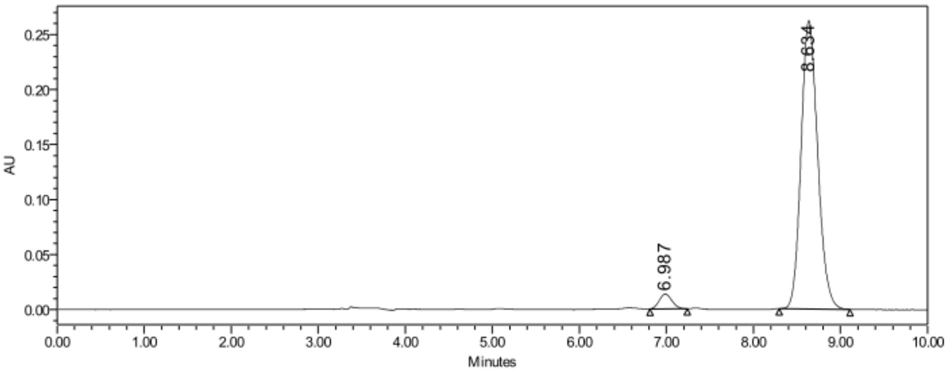

	RT	Area	% Area	Height
1	6.987	135397	3.76	13395
2	8.634	3464515	96.24	261977

Rac-**4b**

SAMPLE INFORMATION

Sample Name:	xt-7-193-1-rac-5%-IG	Acquired By:	System
Sample Type:	Unknown	Sample Set Name:	20240715
Vial:	91	Acq. Method Set:	5%qb
Injection #:	1	Processing Method:	xt 7 193 1 rac
Injection Volume:	10.00 ul	Channel Name:	254.0nm
Run Time:	13.0 Minutes	Proc. Chnl. Descr.:	2998 PDA 254.0 nm (2998
Date Acquired:	8/15/2024 5:16:58 PM CST		
Date Processed:	8/15/2024 7:23:07 PM CST		

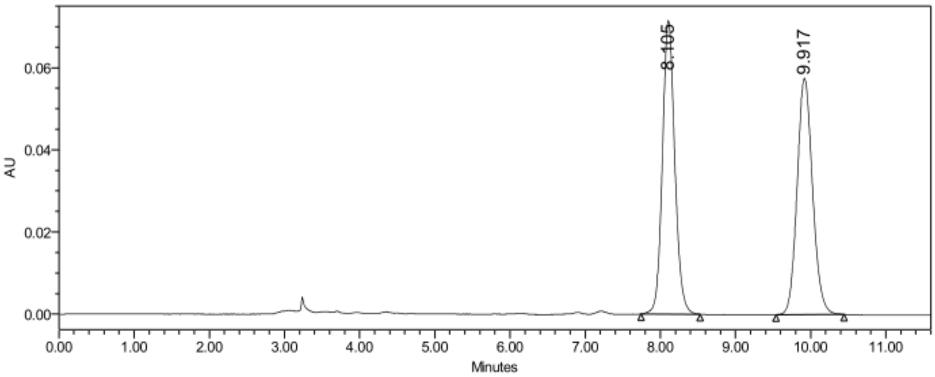

	RT	Area	% Area	Height
1	8.105	820574	49.95	71477
2	9.917	822321	50.05	57577

Asy-**4b**

SAMPLE INFORMATION

Sample Name:	xt-7-193-2-asy-5%-IG	Acquired By:	System
Sample Type:	Unknown	Sample Set Name:	20240715
Vial:	93	Acq. Method Set:	5%qb
Injection #:	1	Processing Method:	XT 7 193 2 ASY
Injection Volume:	10.00 ul	Channel Name:	254.0nm
Run Time:	13.0 Minutes	Proc. Chnl. Descr.:	2998 PDA 254.0 nm (2998
Date Acquired:	8/15/2024 5:44:21 PM CST		
Date Processed:	8/15/2024 7:24:23 PM CST		

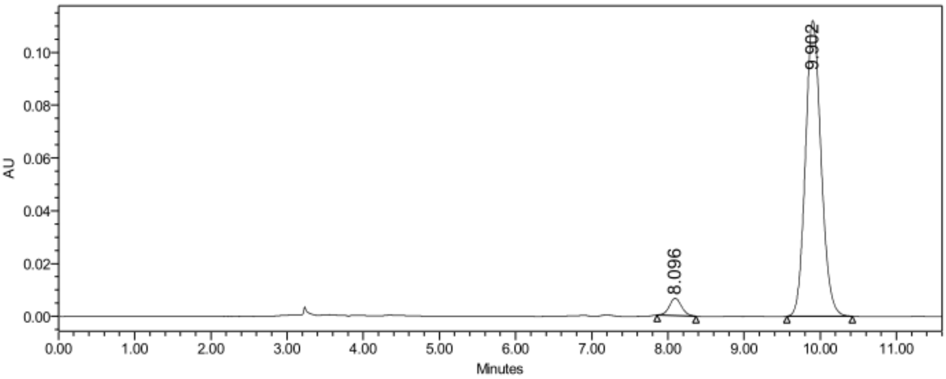

	RT	Area	% Area	Height
1	8.096	72443	4.32	6535
2	9.902	1602684	95.68	112050

Rac-**4c**

SAMPLE INFORMATION

Sample Name:	xt-7-191-1-rac-5%-IG	Acquired By:	System
Sample Type:	Unknown	Sample Set Name:	
Vial:	90	Acq. Method Set:	5%qb
Injection #:	1	Processing Method:	xt 7 191 1 rac
Injection Volume:	10.00 ul	Channel Name:	254.0nm
Run Time:	60.0 Minutes	Proc. Chnl. Descr.:	2998 PDA 254.0 nm (2998
Date Acquired:	8/15/2024 4:49:20 PM CST		
Date Processed:	8/15/2024 7:22:09 PM CST		

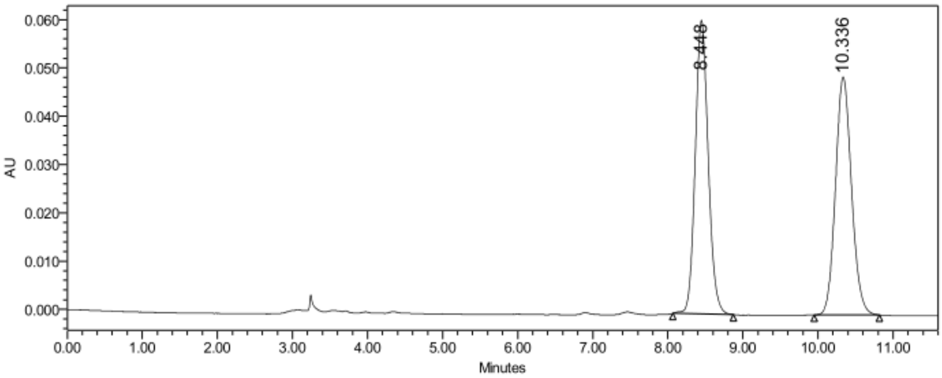

	RT	Area	% Area	Height
1	8.448	727049	49.87	60795
2	10.336	730743	50.13	49283

Asy-**4c**

SAMPLE INFORMATION

Sample Name:	xt-7-191-2-asy-5%-IG	Acquired By:	System
Sample Type:	Unknown	Sample Set Name:	20240715
Vial:	92	Acq. Method Set:	5%qb
Injection #:	1	Processing Method:	XT 7 191 2 ASY
Injection Volume:	10.00 ul	Channel Name:	254.0nm
Run Time:	13.0 Minutes	Proc. Chnl. Descr.:	2998 PDA 254.0 nm (2998
Date Acquired:	8/15/2024 5:30:39 PM CST		
Date Processed:	8/15/2024 7:20:07 PM CST		

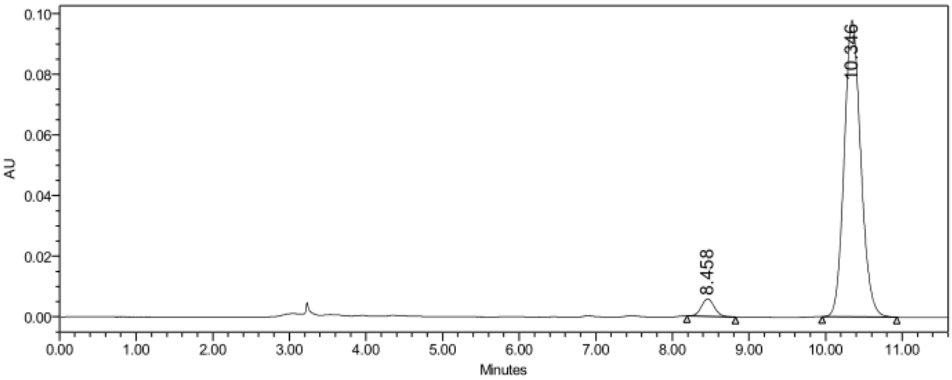

	RT	Area	% Area	Height
1	8.458	66535	4.32	5739
2	10.346	1472787	95.68	97788

Rac-**4d**

SAMPLE INFORMATION

Sample Name:	xt-8-8-1-rac-10%-IG	Acquired By:	System
Sample Type:	Unknown	Sample Set Name	
Vial:	56	Acq. Method Set:	10% quanbo
Injection #:	1	Processing Method	xt 8 8 1 rac
Injection Volume:	10.00 ul	Channel Name:	254.0nm
Run Time:	60.0 Minutes	Proc. Chnl. Descr.:	2998 PDA 254.0 nm (2998
Date Acquired:	8/25/2024 21:38:37 CST		
Date Processed:	8/25/2024 22:27:28 CST		

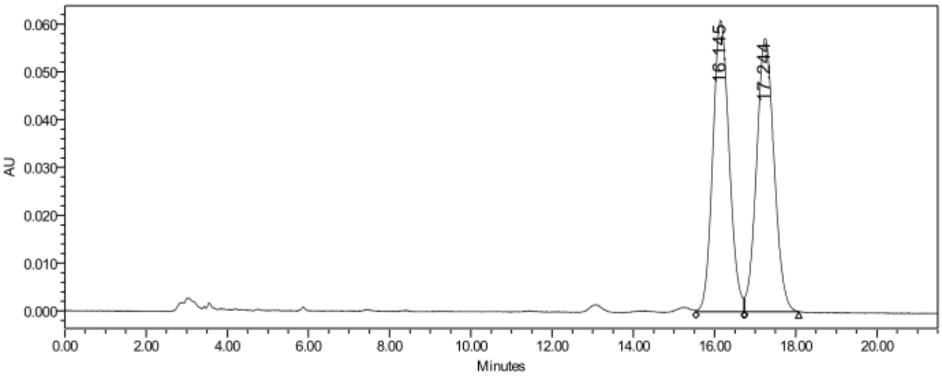

	RT	Area	% Area	Height
1	16.145	1701816	49.78	60924
2	17.244	1716836	50.22	57148

Asy-**4d**

SAMPLE INFORMATION

Sample Name:	xt-8-8-2-asy-10%-IG	Acquired By:	System
Sample Type:	Unknown	Sample Set Name	20240825
Vial:	85	Acq. Method Set:	10% quanbo
Injection #:	1	Processing Method	xt 8 8 2 asy
Injection Volume:	10.00 ul	Channel Name:	254.0nm
Run Time:	22.0 Minutes	Proc. Chnl. Descr.:	2998 PDA 254.0 nm (2998
Date Acquired:	8/25/2024 22:02:57 CST		
Date Processed:	8/25/2024 22:26:06 CST		

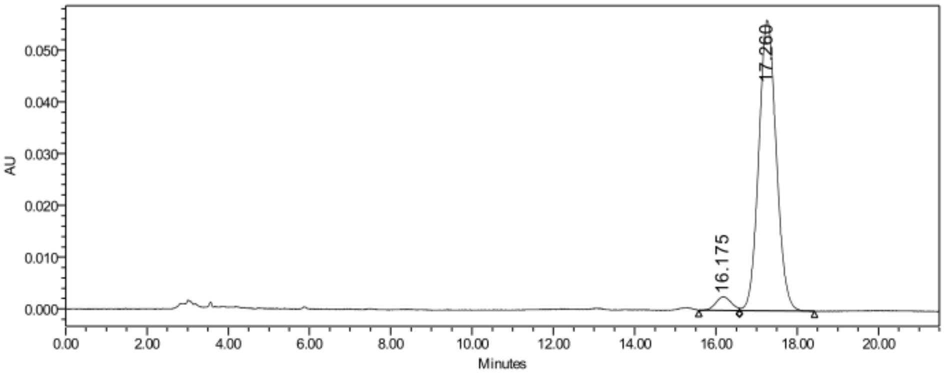

	RT	Area	% Area	Height
1	16.175	68573	3.92	2607
2	17.260	1679781	96.08	56100

Rac-**4e**

SAMPLE INFORMATION

Sample Name:	xt-8-2-1-RAC-30%-IG	Acquired By:	System
Sample Type:	Unknown	Sample Set Name	
Vial:	46	Acq. Method Set:	30%quanbo
Injection #:	1	Processing Method	xt 8 2 1 rac
Injection Volume:	10.00 ul	Channel Name:	254.0nm
Run Time:	60.0 Minutes	Proc. Chnl. Descr.:	2998 PDA 254.0 nm (2998
Date Acquired:	8/17/2024 16:44:31 CST		
Date Processed:	8/21/2024 22:40:39 CST		

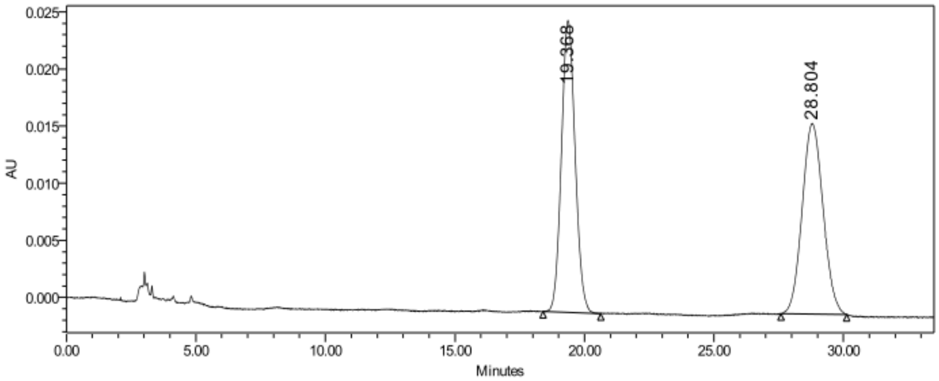

	RT	Area	% Area	Height
1	19.368	940659	49.99	25554
2	28.804	941065	50.01	16698

Asy-**4e**

SAMPLE INFORMATION

Sample Name:	xt-8-2-2-asy-30%-IG	Acquired By:	System
Sample Type:	Unknown	Sample Set Name	20240817
Vial:	47	Acq. Method Set:	30%quanbo
Injection #:	1	Processing Method	xt 8 2 2 asy
Injection Volume:	10.00 ul	Channel Name:	254.0nm
Run Time:	35.0 Minutes	Proc. Chnl. Descr.:	2998 PDA 254.0 nm (2998
Date Acquired:	8/17/2024 17:20:44 CST		
Date Processed:	8/21/2024 22:44:16 CST		

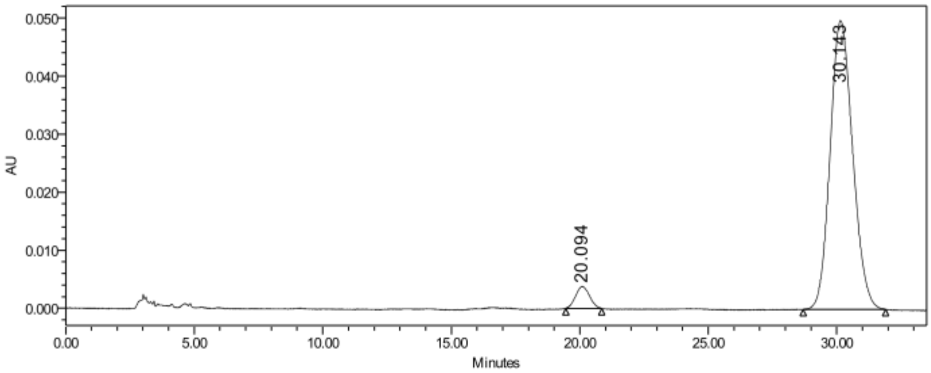

	RT	Area	% Area	Height
1	20.094	142833	4.49	3815
2	30.143	3037001	95.51	49803

Rac-**4f**

SAMPLE INFORMATION

Sample Name:	xt-8-17-1-rac-2%-IG	Acquired By:	System
Sample Type:	Unknown	Sample Set Name	
Vial:	36	Acq. Method Set:	2% quanbo
Injection #:	1	Processing Method	xt 8 17 1 rac
Injection Volume:	10.00 ul	Channel Name:	243.0nm
Run Time:	60.0 Minutes	Proc. Chnl. Descr.:	2998 PDA 243.0 nm (2998
Date Acquired:	8/30/2024 16:03:35 CST		
Date Processed:	8/30/2024 16:43:26 CST		

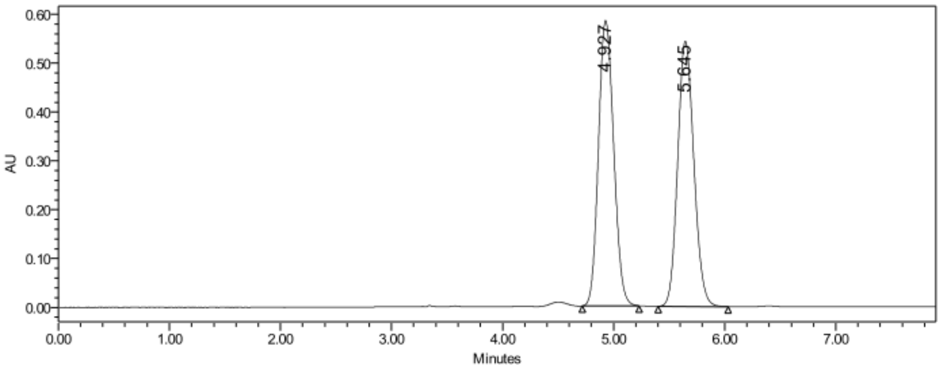

	RT	Area	% Area	Height
1	4.927	5556476	50.12	584321
2	5.645	5530098	49.88	542869

Asy-**4f**

SAMPLE INFORMATION

Sample Name:	xt-8-17-2-ASY-2%-IG	Acquired By:	System
Sample Type:	Unknown	Sample Set Name	
Vial:	51	Acq. Method Set:	2% quanbo
Injection #:	1	Processing Method	xt 8 17 2 asy
Injection Volume:	10.00 ul	Channel Name:	243.0nm
Run Time:	8.0 Minutes	Proc. Chnl. Descr.:	2998 PDA 243.0 nm (2998
Date Acquired:	8/30/2024 16:15:00 CST		
Date Processed:	8/30/2024 16:41:41 CST		

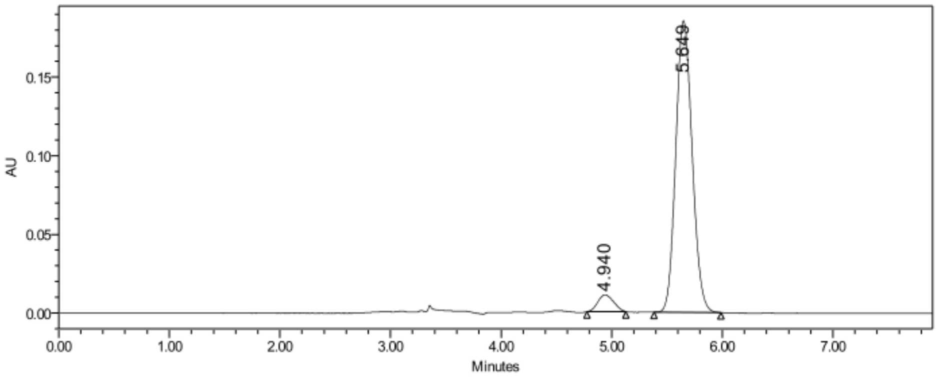

	RT	Area	% Area	Height
1	4.940	106682	5.25	10680
2	5.649	1924219	94.75	185594

Rac-**4g**

SAMPLE INFORMATION

Sample Name:	xt-8-6-1-RAC-5%-IG	Acquired By:	System
Sample Type:	Unknown	Sample Set Name	
Vial:	50	Acq. Method Set:	5% quanbo
Injection #:	1	Processing Method	xt 8 6 1 rac
Injection Volume:	10.00 ul	Channel Name:	254.0nm
Run Time:	60.0 Minutes	Proc. Chnl. Descr.:	2998 PDA 254.0 nm (2998
Date Acquired:	8/20/2024 21:37:32 CST		
Date Processed:	8/21/2024 22:50:20 CST		

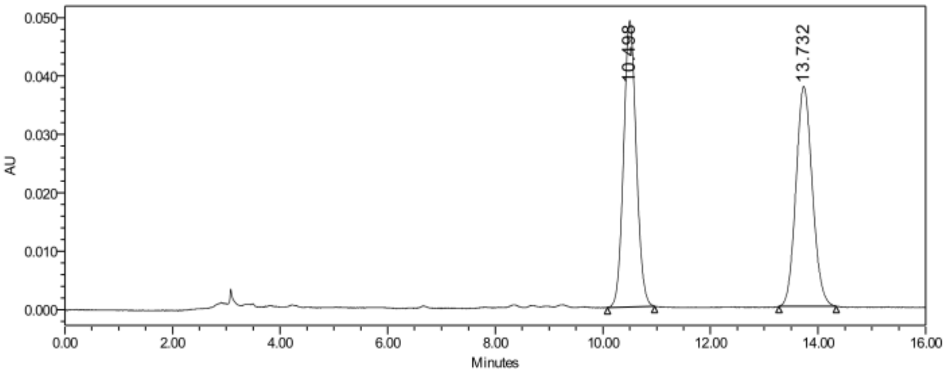

	RT	Area	% Area	Height
1	10.498	800046	50.04	49009
2	13.732	798632	49.96	37694

Asy-**4g**

SAMPLE INFORMATION

Sample Name:	xt-8-6-2-asy-5%-IG	Acquired By:	System
Sample Type:	Unknown	Sample Set Name	20240820
Vial:	83	Acq. Method Set:	5% quanbo
Injection #:	1	Processing Method	xt 8 6 2 asy
Injection Volume:	10.00 ul	Channel Name:	254.0nm
Run Time:	16.0 Minutes	Proc. Chnl. Descr.:	2998 PDA 254.0 nm (2998
Date Acquired:	8/20/2024 22:21:25 CST		
Date Processed:	8/21/2024 22:49:13 CST		

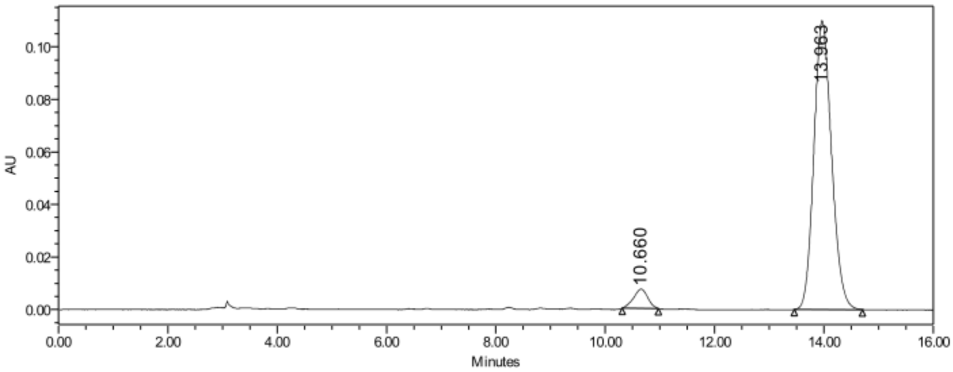

	RT	Area	% Area	Height
1	10.660	131788	5.07	7315
2	13.963	2468403	94.93	110038

Rac-**4h**

SAMPLE INFORMATION

Sample Name:	xt-8-27-1-rac-20%-IG	Acquired By:	System
Sample Type:	Unknown	Sample Set Name	
Vial:	65	Acq. Method Set:	20% quanbo
Injection #:	1	Processing Method	xt 8 27 1 rac
Injection Volume:	10.00 ul	Channel Name:	254.0nm
Run Time:	60.0 Minutes	Proc. Chnl. Descr.:	2998 PDA 254.0 nm (2998
Date Acquired:	9/1/2024 20:08:49 CST		
Date Processed:	9/5/2024 20:23:23 CST		

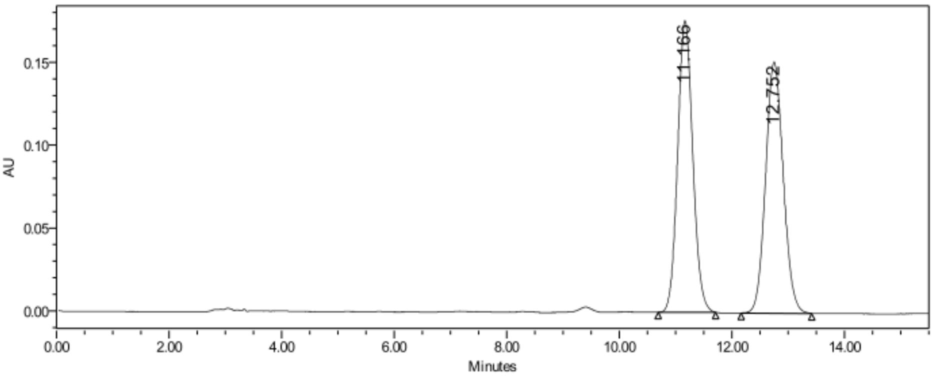

	RT	Area	% Area	Height
1	11.166	3290562	50.03	176070
2	12.752	3286408	49.97	151625

Asy-**4h**

SAMPLE INFORMATION

Sample Name:	xt-8-27-2-asy-20%-IG	Acquired By:	System
Sample Type:	Unknown	Sample Set Name	
Vial:	95	Acq. Method Set:	20% quanbo
Injection #:	1	Processing Method	xt 8 27 2 asy
Injection Volume:	10.00 ul	Channel Name:	254.0nm
Run Time:	60.0 Minutes	Proc. Chnl. Descr.:	2998 PDA 254.0 nm (2998
Date Acquired:	9/1/2024 20:26:42 CST		
Date Processed:	9/5/2024 20:17:50 CST		

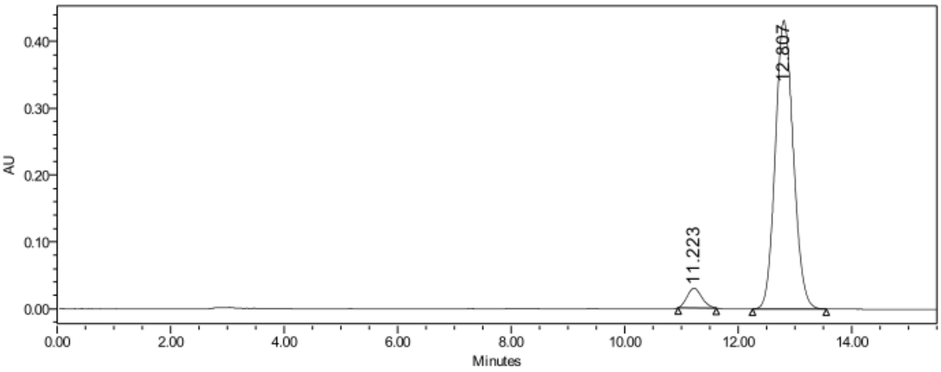

	RT	Area	% Area	Height
1	11.223	524492	5.25	29314
2	12.807	9463885	94.75	432051

Rac-**4i**

SAMPLE INFORMATION

Sample Name:	xt-8-25-1-rac-20%-IG	Acquired By:	System
Sample Type:	Unknown	Sample Set Name	
Vial:	23	Acq. Method Set:	20% quanbo
Injection #:	1	Processing Method	xt 8 25 1 rac
Injection Volume:	10.00 ul	Channel Name:	254.0nm
Run Time:	60.0 Minutes	Proc. Chnl. Descr.:	2998 PDA 254.0 nm (2998
Date Acquired:	9/1/2024 19:31:15 CST		
Date Processed:	9/5/2024 20:15:50 CST		

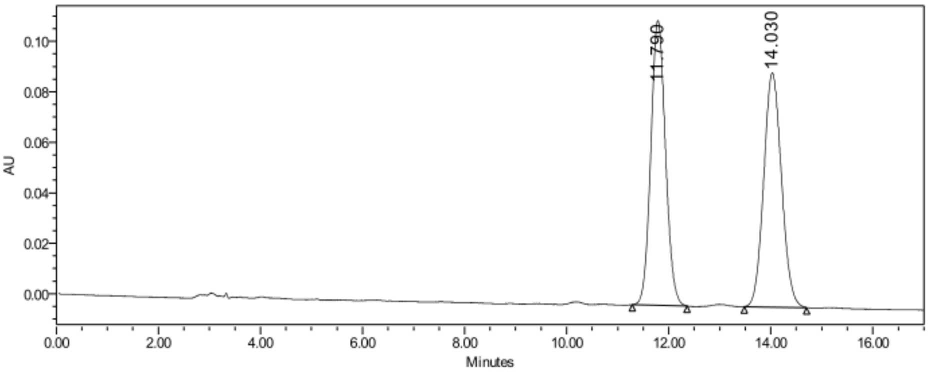

	RT	Area	% Area	Height
1	11.790	2243482	50.11	112905
2	14.030	2233465	49.89	92721

Asy-**4i**

SAMPLE INFORMATION

Sample Name:	xt-8-25-2-asy-20%-IG	Acquired By:	System
Sample Type:	Unknown	Sample Set Name	
Vial:	31	Acq. Method Set:	20% quanbo
Injection #:	1	Processing Method	xt 8 25 2 asy
Injection Volume:	10.00 ul	Channel Name:	254.0nm
Run Time:	60.0 Minutes	Proc. Chnl. Descr.:	2998 PDA 254.0 nm (2998
Date Acquired:	9/1/2024 19:49:57 CST		
Date Processed:	9/5/2024 20:14:18 CST		

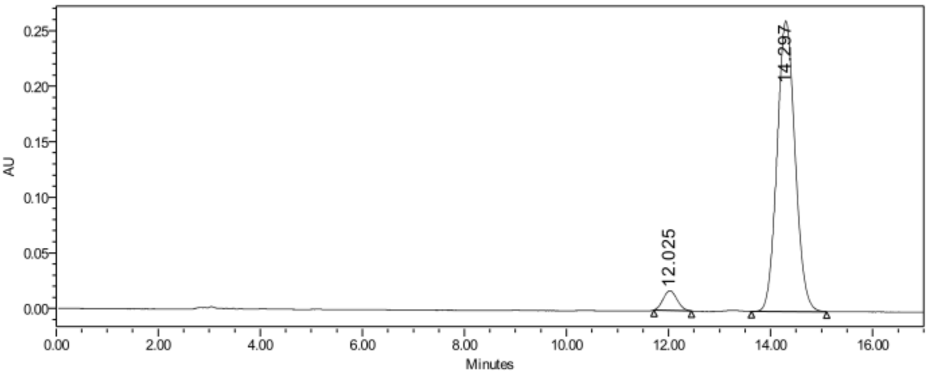

	RT	Area	% Area	Height
1	12.025	337262	4.99	17729
2	14.297	6420187	95.01	261873

Rac-**4j**

SAMPLE INFORMATION

Sample Name:	xt-8-31-1-RAC-20%-IG	Acquired By:	System
Sample Type:	Unknown	Sample Set Name	
Vial:	27	Acq. Method Set:	20% quanbo
Injection #:	1	Processing Method	xt 8 31 1 rac
Injection Volume:	20.00 ul	Channel Name:	254.0nm
Run Time:	60.0 Minutes	Proc. Chnl. Descr.:	2998 PDA 254.0 nm (2998
Date Acquired:	9/6/2024 22:39:45 CST		
Date Processed:	9/7/2024 20:50:47 CST		

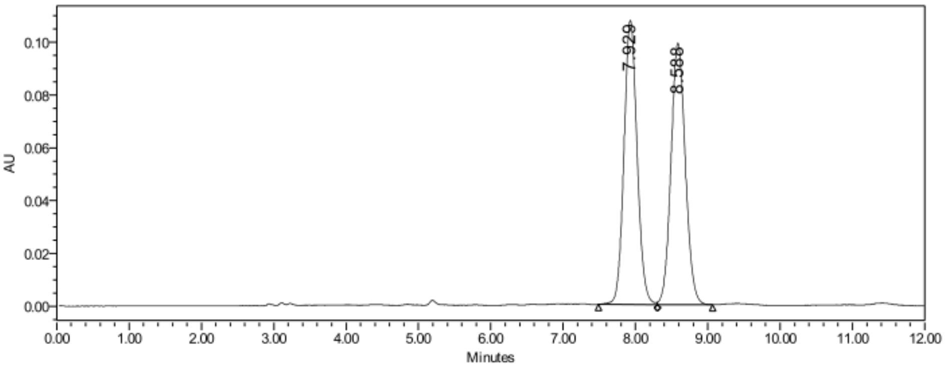

	RT	Area	% Area	Height
1	7.929	1361912	49.95	107670
2	8.588	1364614	50.05	99120

Asy-**4j**

SAMPLE INFORMATION

Sample Name:	xt-8-31-2-ASY-20%-IG	Acquired By:	System
Sample Type:	Unknown	Sample Set Name	20240906
Vial:	15	Acq. Method Set:	20% quanbo
Injection #:	1	Processing Method	xt 8 31 2 asy
Injection Volume:	10.00 ul	Channel Name:	254.0nm
Run Time:	12.0 Minutes	Proc. Chnl. Descr.:	2998 PDA 254.0 nm (2998
Date Acquired:	9/6/2024 21:59:40 CST		
Date Processed:	9/7/2024 20:49:15 CST		

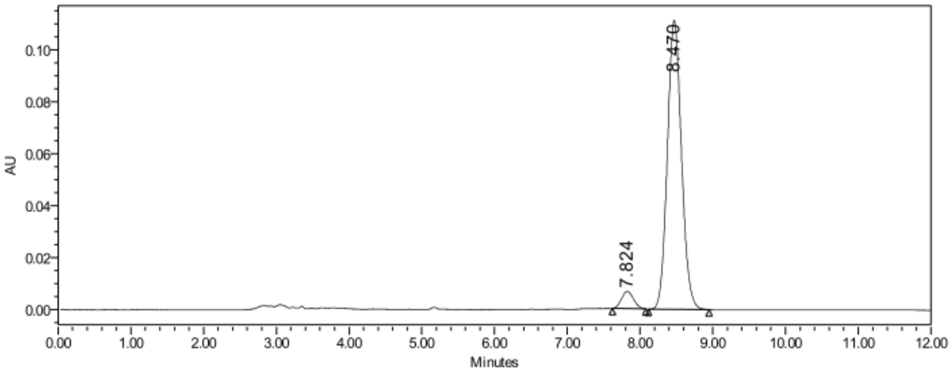

	RT	Area	% Area	Height
1	7.824	76290	4.87	6648
2	8.470	1490592	95.13	111296

Rac-**4k**

SAMPLE INFORMATION

Sample Name:	xt-8-46-1-RAC-20%-IG	Acquired By:	System
Sample Type:	Unknown	Sample Set Name:	
Vial:	48	Acq. Method Set:	20%qb
Injection #:	1	Processing Method:	XT 8 46 1 RAC
Injection Volume:	10.00 ul	Channel Name:	254.0nm
Run Time:	60.0 Minutes	Proc. Chnl. Descr.:	2998 PDA 254.0 nm (2998
Date Acquired:	9/17/2024 3:27:05 PM CST		
Date Processed:	9/17/2024 4:44:02 PM CST		

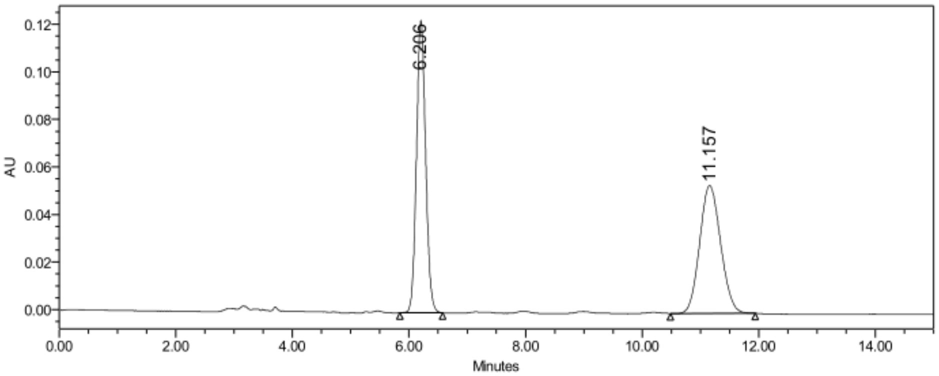

	RT	Area	% Area	Height
1	6.206	1341470	50.06	122914
2	11.157	1338207	49.94	53791

Asy-**4k**

SAMPLE INFORMATION

Sample Name:	xt-8-46-2-ASY-20%-IG	Acquired By:	System
Sample Type:	Unknown	Sample Set Name:	
Vial:	61	Acq. Method Set:	20%qb
Injection #:	1	Processing Method:	XT 8 46 2 ASY
Injection Volume:	30.00 ul	Channel Name:	254.0nm
Run Time:	60.0 Minutes	Proc. Chnl. Descr.:	2998 PDA 254.0 nm (2998
Date Acquired:	9/17/2024 3:44:22 PM CST		
Date Processed:	9/17/2024 4:42:37 PM CST		

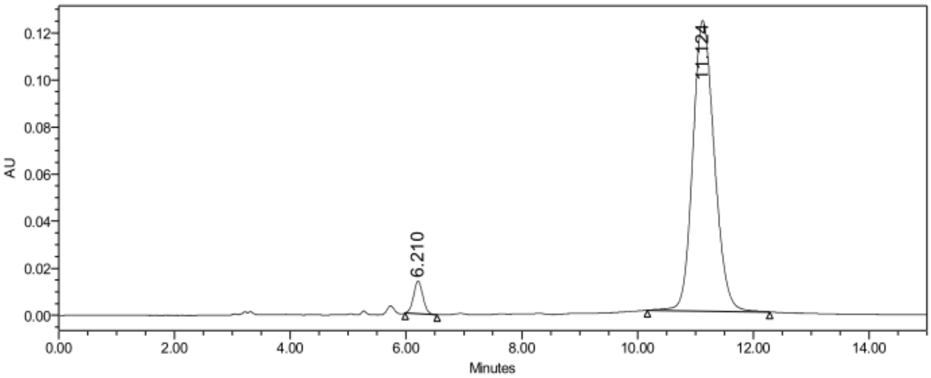

	RT	Area	% Area	Height
1	6.210	156058	4.69	13886
2	11.124	3174386	95.31	123446

Rac-**4l**

SAMPLE INFORMATION			
Sample Name:	xt-8-35-1-RAC-30%-IC	Acquired By:	System
Sample Type:	Unknown	Sample Set Name	
Vial:	1	Acq. Method Set:	30%quanbo
Injection #:	1	Processing Method	xt 8 35 1 rac
Injection Volume:	10.00 ul	Channel Name:	254.0nm
Run Time:	60.0 Minutes	Proc. Chnl. Descr.:	2998 PDA 254.0 nm (2998
Date Acquired:	9/7/2024 19:17:31 CST		
Date Processed:	9/7/2024 20:10:19 CST		

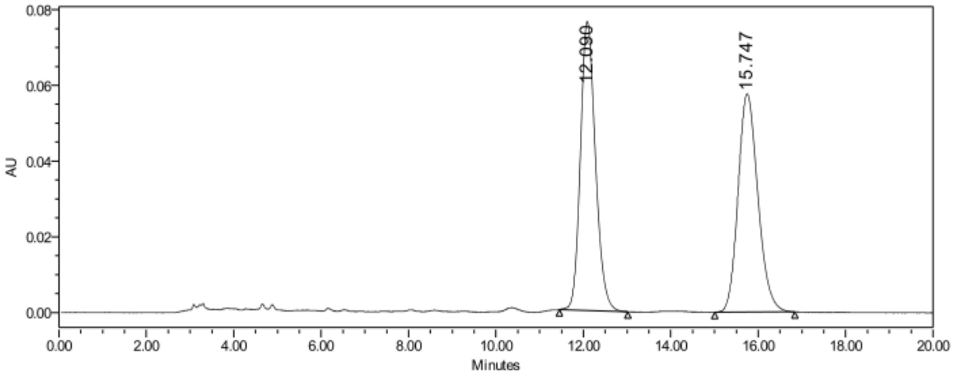

	RT	Area	% Area	Height
1	12.090	1833233	50.07	76441
2	15.747	1828308	49.93	57693

Asy-**4l**

SAMPLE INFORMATION			
Sample Name:	xt-8-35-2-ASY-30%-IC	Acquired By:	System
Sample Type:	Unknown	Sample Set Name	20240907
Vial:	36	Acq. Method Set:	30%quanbo
Injection #:	1	Processing Method	xt 8 35 2 asy
Injection Volume:	10.00 ul	Channel Name:	254.0nm
Run Time:	20.0 Minutes	Proc. Chnl. Descr.:	2998 PDA 254.0 nm (2998
Date Acquired:	9/7/2024 19:38:59 CST		
Date Processed:	9/7/2024 20:08:57 CST		

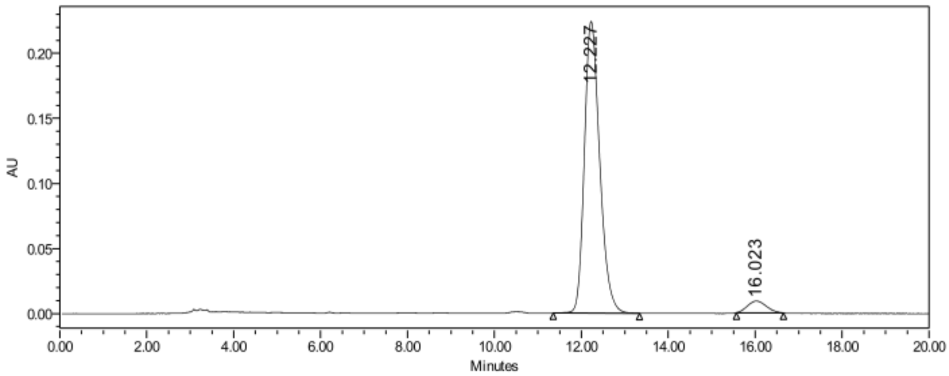

	RT	Area	% Area	Height
1	12.227	5518775	95.34	224344
2	16.023	269813	4.66	9054

Rac-**4m**

SAMPLE INFORMATION

Sample Name:	xt-7-184-2-rac-2%-IG	Acquired By:	System
Sample Type:	Unknown	Sample Set Name	20240801
Vial:	38	Acq. Method Set:	2% quanbo
Injection #:	1	Processing Method	xt 7 184 2 rac
Injection Volume:	10.00 ul	Channel Name:	254.0nm
Run Time:	16.0 Minutes	Proc. Chnl. Descr.:	2998 PDA 254.0 nm (2998
Date Acquired:	8/1/2024 12:07:13 CST		
Date Processed:	8/1/2024 14:31:55 CST		

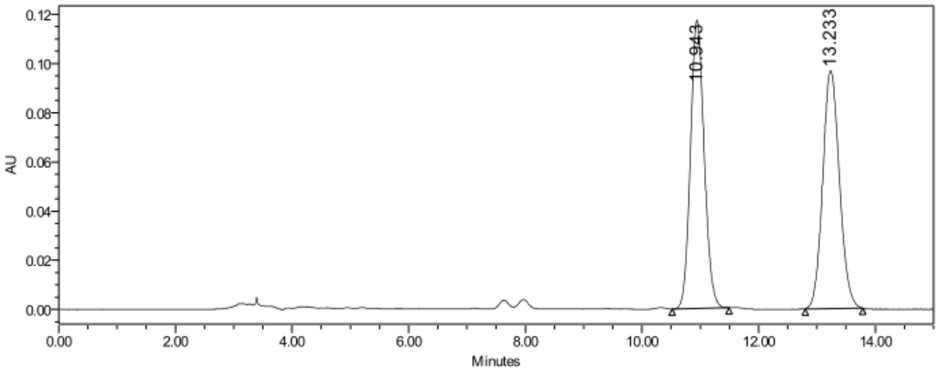

	RT	Area	% Area	Height
1	10.943	1922851	49.92	117283
2	13.233	1929176	50.08	96833

Asy-**4m**

SAMPLE INFORMATION

Sample Name:	xt-7-181-1-asy-2%-IG	Acquired By:	System
Sample Type:	Unknown	Sample Set Name	20240801
Vial:	36	Acq. Method Set:	2% quanbo
Injection #:	1	Processing Method	xt 7 181 1 asy
Injection Volume:	10.00 ul	Channel Name:	254.0nm
Run Time:	16.0 Minutes	Proc. Chnl. Descr.:	2998 PDA 254.0 nm (2998
Date Acquired:	8/1/2024 11:33:52 CST		
Date Processed:	8/1/2024 14:34:42 CST		

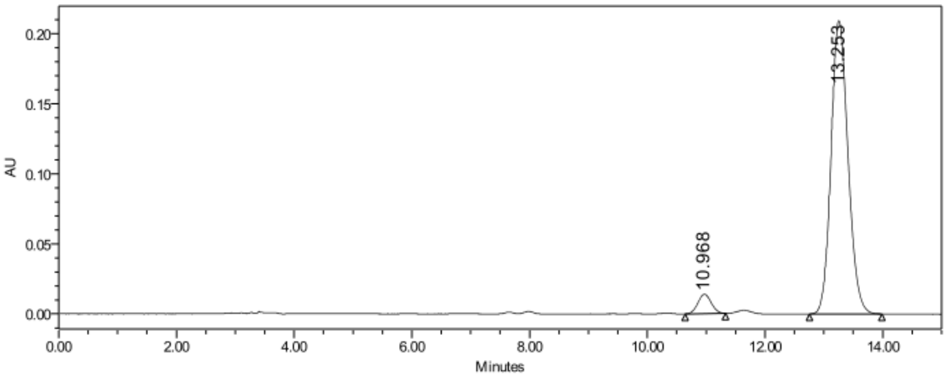

	RT	Area	% Area	Height
1	10.968	219543	4.96	13873
2	13.253	4209168	95.04	209489

Rac-**4n**

SAMPLE INFORMATION

Sample Name:	xt-7-187-1-rac-2%-IG	Acquired By:	System
Sample Type:	Unknown	Sample Set Name:	20240803
Vial:	114	Acq. Method Set:	2%qb
Injection #:	1	Processing Method:	XT 7 187 1 rac
Injection Volume:	10.00 ul	Channel Name:	254.0nm
Run Time:	18.0 Minutes	Proc. Chnl. Descr.:	2998 PDA 254.0 nm (2998
Date Acquired:	8/3/2024 5:36:10 PM CST		
Date Processed:	8/21/2024 10:35:24 PM CST		

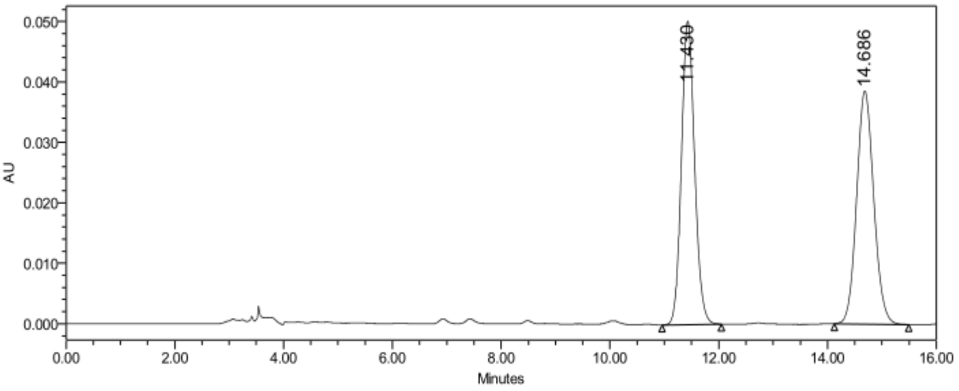

	RT	Area	% Area	Height
1	11.430	845063	50.05	50241
2	14.686	843379	49.95	38553

Asy-**4n**

SAMPLE INFORMATION

Sample Name:	xt-7-187-2-asy-2%-IG	Acquired By:	System
Sample Type:	Unknown	Sample Set Name:	20240803
Vial:	113	Acq. Method Set:	2%qb
Injection #:	1	Processing Method:	XT 7 187 2 ASY
Injection Volume:	10.00 ul	Channel Name:	254.0nm
Run Time:	18.0 Minutes	Proc. Chnl. Descr.:	2998 PDA 254.0 nm (2998
Date Acquired:	8/3/2024 5:17:16 PM CST		
Date Processed:	8/21/2024 10:37:38 PM CST		

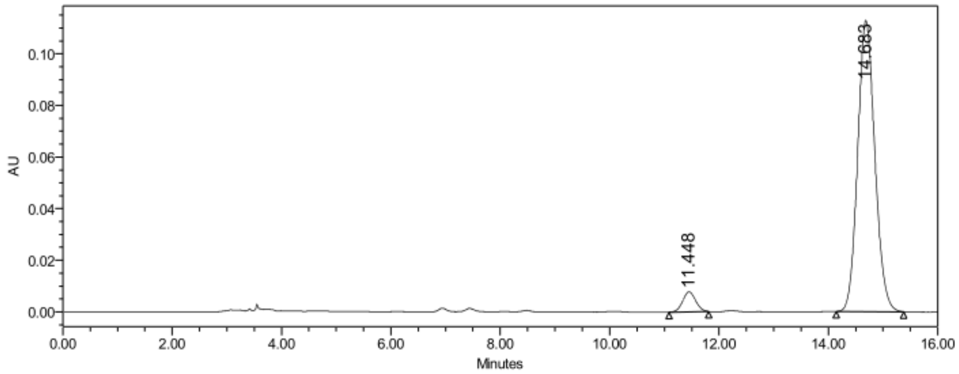

	RT	Area	% Area	Height
1	11.448	127869	4.96	7780
2	14.683	2451798	95.04	112875

Rac-**4o**

SAMPLE INFORMATION			
Sample Name:	xt-7-183-1-rac-1%-0.7flow-IG	Acquired By:	System
Sample Type:	Unknown	Sample Set Name:	20240802
Vial:	47	Acq. Method Set:	1%qb 07flow
Injection #:	1	Processing Method:	XT 7 183 1 rac
Injection Volume:	10.00 ul	Channel Name:	243.0nm
Run Time:	14.0 Minutes	Proc. Chnl. Descr.:	2998 PDA 243.0 nm (2998
Date Acquired:	8/2/2024 3:58:35 PM CST		
Date Processed:	8/21/2024 10:28:55 PM CST		

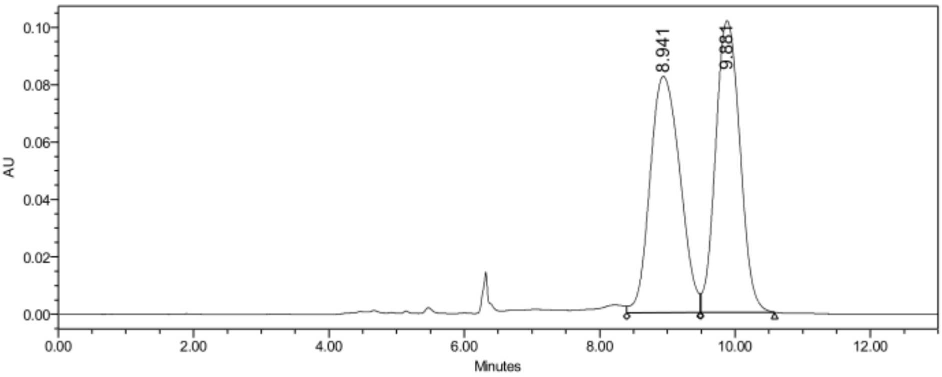

	RT	Area	% Area	Height
1	8.941	2542019	49.95	82447
2	9.881	2546996	50.05	101714

Asy-**4o**

SAMPLE INFORMATION			
Sample Name:	xt-7-183-2-asy-1%-0.7flow-IG	Acquired By:	System
Sample Type:	Unknown	Sample Set Name:	20240802
Vial:	46	Acq. Method Set:	1%qb 07flow
Injection #:	1	Processing Method:	XT 7 183 2 ASY
Injection Volume:	10.00 ul	Channel Name:	243.0nm
Run Time:	14.0 Minutes	Proc. Chnl. Descr.:	2998 PDA 243.0 nm (2998
Date Acquired:	8/2/2024 3:43:55 PM CST		
Date Processed:	8/21/2024 10:26:13 PM CST		

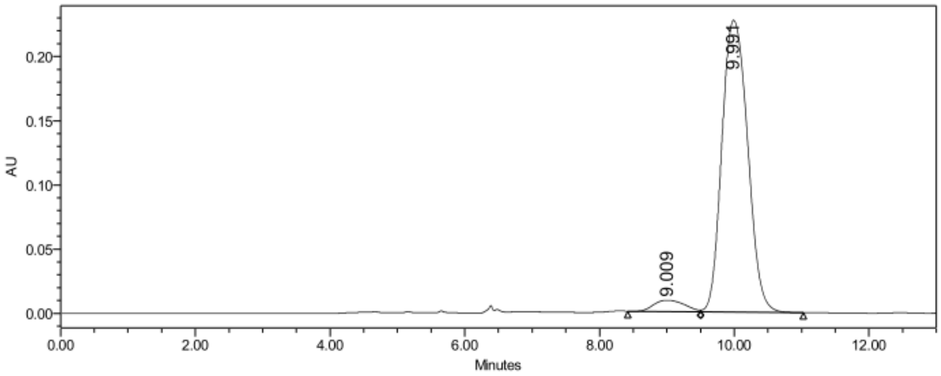

	RT	Area	% Area	Height
1	9.009	279559	4.42	8792
2	9.991	6050240	95.58	227214

Rac-**4p**

SAMPLE INFORMATION

Sample Name:	XT-9-22-rac-2%-IC	Acquired By:	System
Sample Type:	Unknown	Sample Set Name:	
Vial:	102	Acq. Method Set:	2% quanbo
Injection #:	1	Processing Method	XT 9 22 RAC
Injection Volume:	30.00 ul	Channel Name:	230.0nm
Run Time:	60.0 Minutes	Proc. Chnl. Descr.:	2998 PDA 230.0 nm (2998
Date Acquired:	2/14/2025 21:36:41 CST		
Date Processed:	2/14/2025 21:57:42 CST		

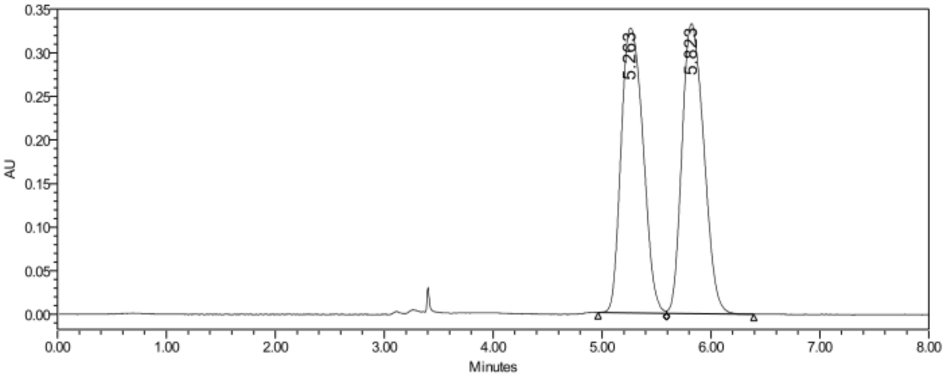

	RT	Area	% Area	Height
1	5.263	4588155	49.82	326667
2	5.823	4621444	50.18	332578

Asy-**4p**

SAMPLE INFORMATION

Sample Name:	XT-9-22-5-asy-2%-IC	Acquired By:	System
Sample Type:	Unknown	Sample Set Name:	
Vial:	103	Acq. Method Set:	2% quanbo
Injection #:	1	Processing Method	XT 9 22 5 ASY
Injection Volume:	30.00 ul	Channel Name:	230.0nm
Run Time:	60.0 Minutes	Proc. Chnl. Descr.:	2998 PDA 230.0 nm (2998
Date Acquired:	2/14/2025 21:47:27 CST		
Date Processed:	2/14/2025 21:58:02 CST		

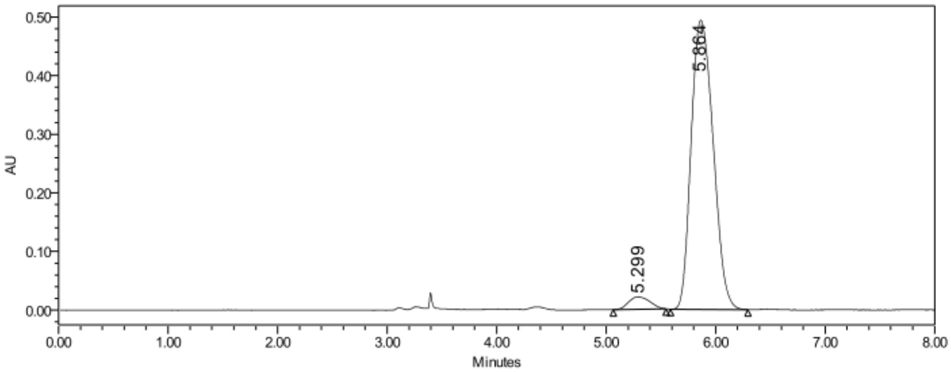

	RT	Area	% Area	Height
1	5.299	280204	3.92	20866
2	5.864	6868462	96.08	492807

Rac-**5a**

SAMPLE INFORMATION

Sample Name:	XT-8-137-2-RAC-2%-IG	Acquired By:	System
Sample Type:	Unknown	Sample Set Name:	
Vial:	62	Acq. Method Set:	2% quanbo
Injection #:	1	Processing Method	XT 8 137 2 RAC
Injection Volume:	30.00 ul	Channel Name:	254.0nm
Run Time:	60.0 Minutes	Proc. Chnl. Descr.:	2998 PDA 254.0 nm (2998
Date Acquired:	1/9/2025 15:34:34 CST		
Date Processed:	1/9/2025 15:45:07 CST		

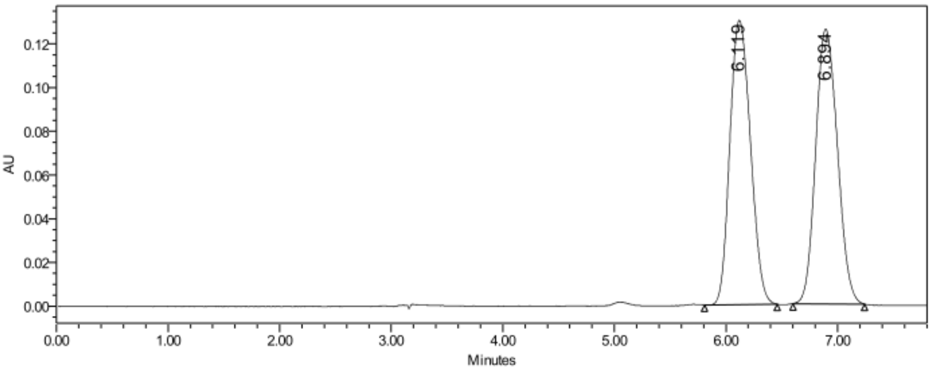

	RT	Area	% Area	Height
1	6.119	1670948	49.54	130046
2	6.894	1701704	50.46	125686

Asy-**5a**

SAMPLE INFORMATION

Sample Name:	XT-8-194-2-ASY-2%-IG	Acquired By:	System
Sample Type:	Unknown	Sample Set Name:	
Vial:	27	Acq. Method Set:	2% quanbo
Injection #:	1	Processing Method	XT 8 194 2 ASY
Injection Volume:	30.00 ul	Channel Name:	254.0nm
Run Time:	60.0 Minutes	Proc. Chnl. Descr.:	2998 PDA 254.0 nm (2998
Date Acquired:	1/9/2025 15:16:06 CST		
Date Processed:	1/9/2025 15:43:34 CST		

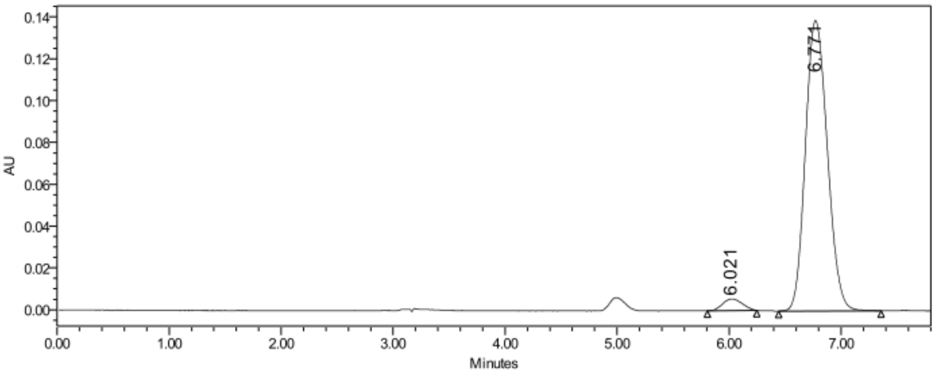

	RT	Area	% Area	Height
1	6.021	66813	3.40	5487
2	6.771	1899074	96.60	138884

Rac-**5b**

SAMPLE INFORMATION

Sample Name:	xt-9-16-2-rac-2%0.5flow-IG	Acquired By:	System
Sample Type:	Unknown	Sample Set Name:	
Vial:	16	Acq. Method Set:	2%qb 05fiow
Injection #:	1	Processing Method:	xt 9 16 2 rac
Injection Volume:	20.00 ul	Channel Name:	254.0nm
Run Time:	60.0 Minutes	Proc. Chnl. Descr.:	2998 PDA 254.0 nm (2998
Date Acquired:	1/18/2025 8:14:13 PM CST		
Date Processed:	1/18/2025 9:45:32 PM CST		

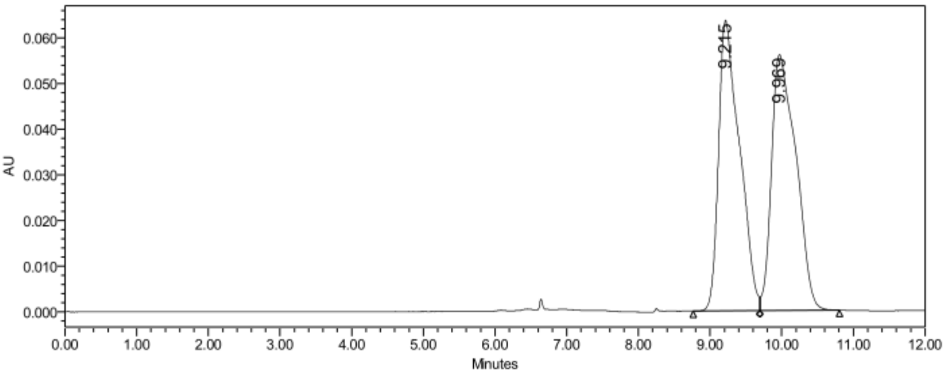

	RT	Area	% Area	Height
1	9.215	1298822	49.59	63556
2	9.969	1320174	50.41	56005

Asy-**5b**

SAMPLE INFORMATION

Sample Name:	xt-9-16-1-asy-2%0.5FLOW-IG	Acquired By:	System
Sample Type:	Unknown	Sample Set Name:	0118
Vial:	107	Acq. Method Set:	2%qb 05fiow
Injection #:	1	Processing Method:	xt 9 16 1 asy
Injection Volume:	30.00 ul	Channel Name:	254.0nm
Run Time:	20.0 Minutes	Proc. Chnl. Descr.:	2998 PDA 254.0 nm (2998
Date Acquired:	1/18/2025 5:31:37 PM CST		
Date Processed:	1/18/2025 9:47:32 PM CST		

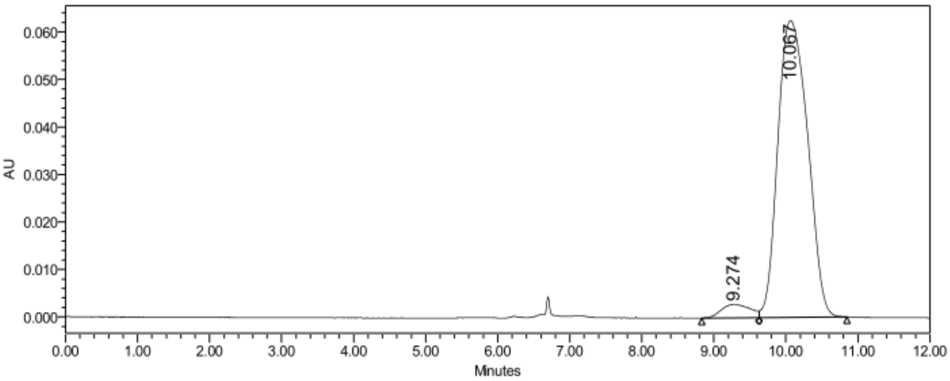

	RT	Area	% Area	Height
1	9.274	78877	4.20	2794
2	10.067	1798899	95.80	62502

Rac-**5c**

SAMPLE INFORMATION

Sample Name:	xt-9-18-2-rac-5%-IG	Acquired By:	System
Sample Type:	Unknown	Sample Set Name:	
Vial:	109	Acq. Method Set:	5%qb
Injection #:	1	Processing Method:	xt 9 18 2 rac
Injection Volume:	30.00 ul	Channel Name:	254.0nm
Run Time:	60.0 Minutes	Proc. Chnl. Descr.:	2998 PDA 254.0 nm (2998
Date Acquired:	1/18/2025 7:29:28 PM CST		
Date Processed:	1/18/2025 9:48:25 PM CST		

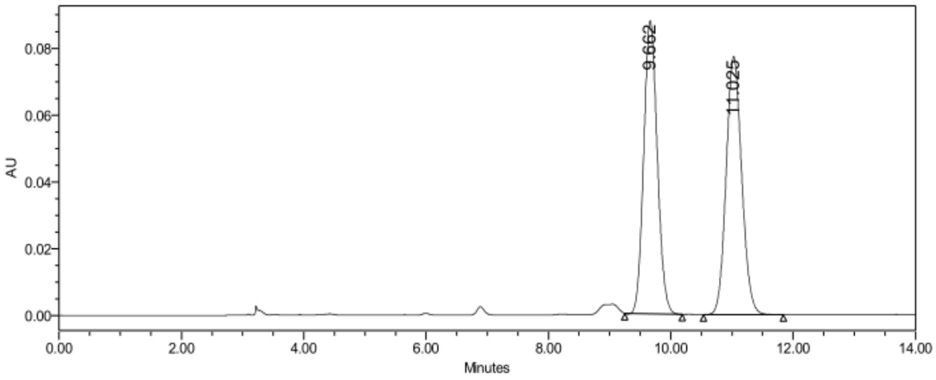

	RT	Area	% Area	Height
1	9.662	1347155	49.64	87802
2	11.025	1366642	50.36	77323

Asy-**5c**

SAMPLE INFORMATION

Sample Name:	xt-9-18-1-asy-5%-IG	Acquired By:	System
Sample Type:	Unknown	Sample Set Name:	
Vial:	108	Acq. Method Set:	5%qb
Injection #:	1	Processing Method:	xt 9 18 1 asy
Injection Volume:	10.00 ul	Channel Name:	254.0nm
Run Time:	60.0 Minutes	Proc. Chnl. Descr.:	2998 PDA 254.0 nm (2998
Date Acquired:	1/18/2025 7:12:53 PM CST		
Date Processed:	1/18/2025 9:49:40 PM CST		

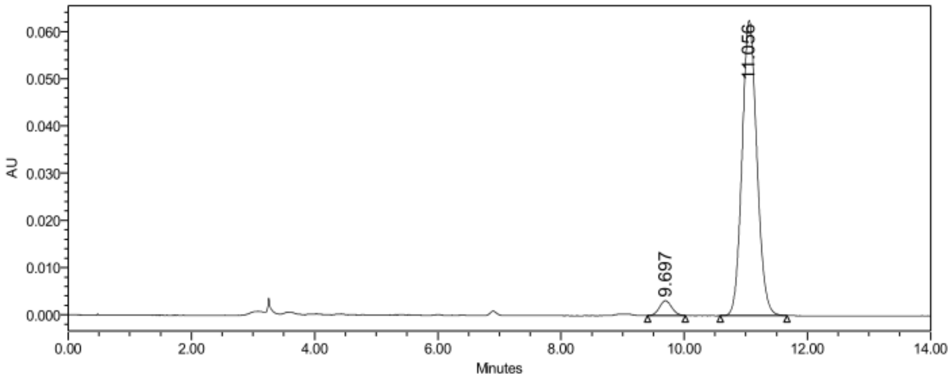

	RT	Area	% Area	Height
1	9.697	44317	4.06	3164
2	11.056	1048098	95.94	62500

Rac-**5d**

SAMPLE INFORMATION

Sample Name:	xt-9-69-1-RAC-5%-IG	Acquired By:	System
Sample Type:	Unknown	Sample Set Name:	
Vial:	27	Acq. Method Set:	5%qb
Injection #:	1	Processing Method:	XT 9 69 1 RAC
Injection Volume:	10.00 ul	Channel Name:	254.0nm
Run Time:	60.0 Minutes	Proc. Chnl. Descr.:	2998 PDA 254.0 nm (2998
Date Acquired:	4/19/2025 7:55:55 PM CST		
Date Processed:	4/19/2025 8:23:22 PM CST		

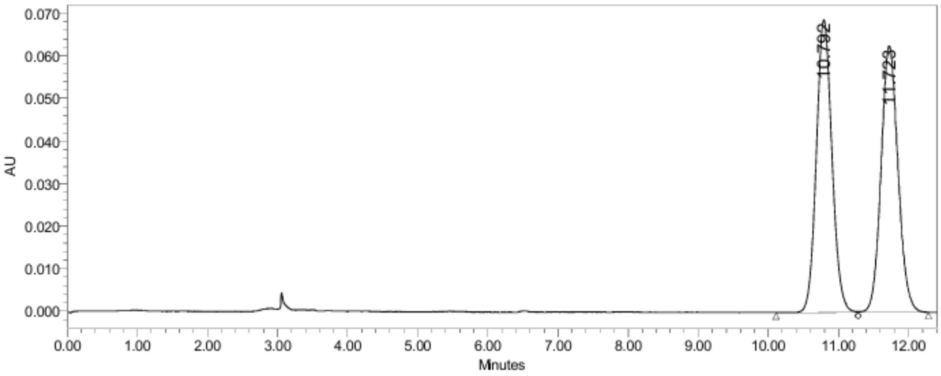

	RT	Area	% Area	Height
1	10.792	1061840	50.16	68727
2	11.723	1055027	49.84	62578

Asy-**5d**

SAMPLE INFORMATION

Sample Name:	xt-9-69-1-ASY-5%-IG	Acquired By:	System
Sample Type:	Unknown	Sample Set Name:	
Vial:	26	Acq. Method Set:	5%qb
Injection #:	1	Processing Method:	XT 9 69 1 ASY
Injection Volume:	30.00 ul	Channel Name:	254.0nm
Run Time:	60.0 Minutes	Proc. Chnl. Descr.:	2998 PDA 254.0 nm (2998
Date Acquired:	4/19/2025 7:39:35 PM CST		
Date Processed:	4/19/2025 8:21:17 PM CST		

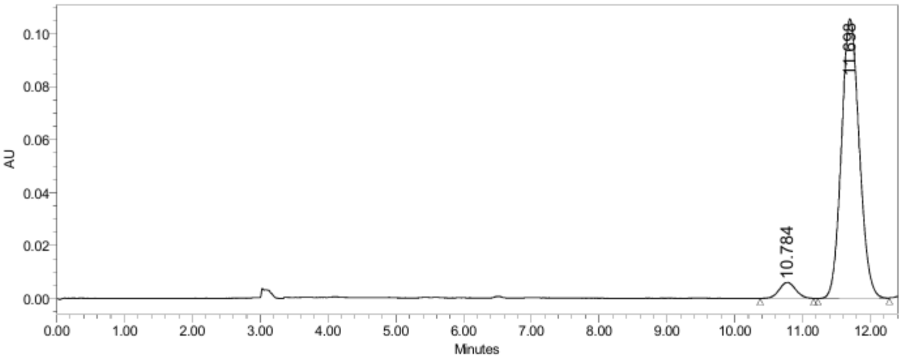

	RT	Area	% Area	Height
1	10.784	101182	4.99	6035
2	11.698	1925204	95.01	105510

Rac-**5e**

SAMPLE INFORMATION

Sample Name:	XT-9-2-2-RAC-2%-IG	Acquired By:	System
Sample Type:	Unknown	Sample Set Name:	0111
Vial:	23	Acq. Method Set:	2% quanbo
Injection #:	1	Processing Method	XT 9 2 2 RAC
Injection Volume:	30.00 ul	Channel Name:	271.0nm
Run Time:	15.0 Minutes	Proc. Chnl. Descr.:	2998 PDA 271.0 nm (2998
Date Acquired:	1/11/2025 21:40:48 CST		
Date Processed:	1/18/2025 23:01:19 CST		

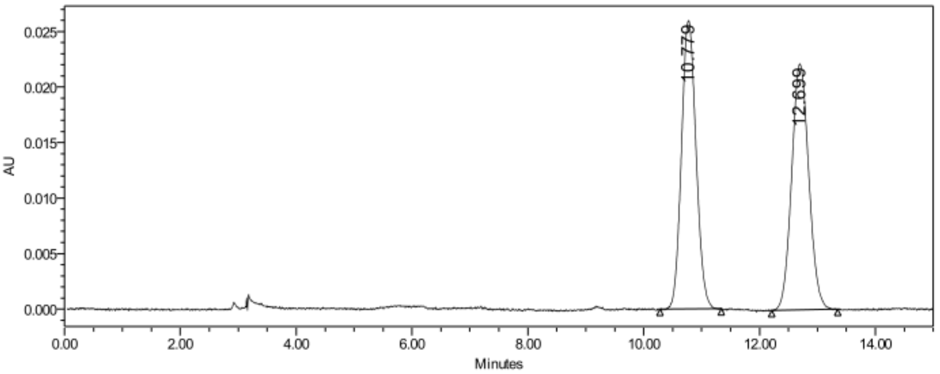

	RT	Area	% Area	Height
1	10.779	454218	49.92	25981
2	12.699	455588	50.08	22172

Asy-**5e**

SAMPLE INFORMATION

Sample Name:	XT-9-2-1-ASY-2%-IG	Acquired By:	System
Sample Type:	Unknown	Sample Set Name:	0111
Vial:	22	Acq. Method Set:	2% quanbo
Injection #:	1	Processing Method	XT 9 2 1 ASY
Injection Volume:	10.00 ul	Channel Name:	271.0nm
Run Time:	15.0 Minutes	Proc. Chnl. Descr.:	2998 PDA 271.0 nm (2998
Date Acquired:	1/11/2025 21:24:58 CST		
Date Processed:	1/18/2025 23:00:25 CST		

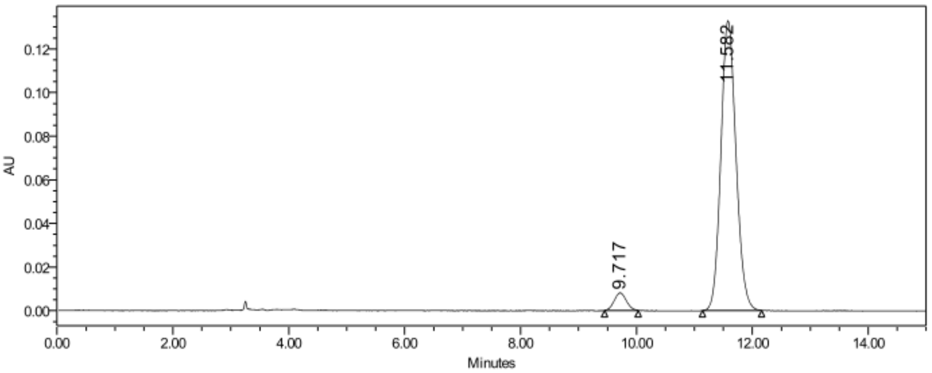

	RT	Area	% Area	Height
1	9.717	114906	4.53	8057
2	11.582	2419120	95.47	132890

Rac-**5f**

SAMPLE INFORMATION

Sample Name:	xt-9-6-2-rac-2%-IG	Acquired By:	System
Sample Type:	Unknown	Sample Set Name:	0113
Vial:	2	Acq. Method Set:	2%qb
Injection #:	1	Processing Method:	xt 9 6 2 rac
Injection Volume:	30.00 ul	Channel Name:	240.0nm
Run Time:	15.0 Minutes	Proc. Chnl. Descr.:	2998 PDA 240.0 nm (2998
Date Acquired:	1/13/2025 10:12:19 AM CST		
Date Processed:	1/18/2025 11:07:44 PM CST		

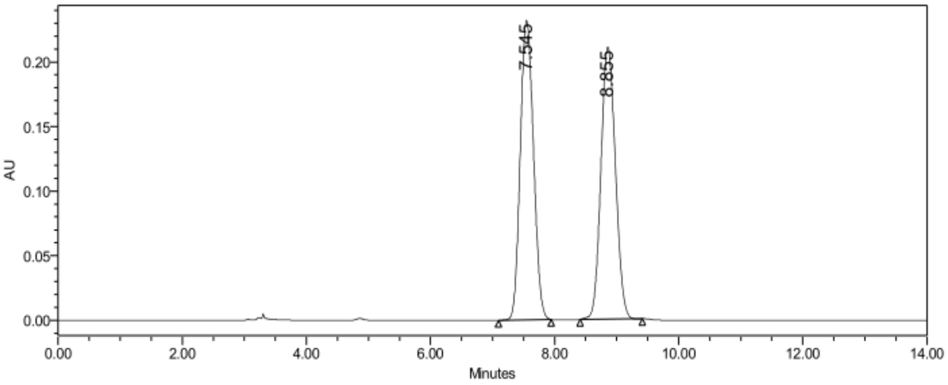

	RT	Area	% Area	Height
1	7.545	3418774	49.99	231856
2	8.855	3420692	50.01	210645

Asy-**5f**

SAMPLE INFORMATION

Sample Name:	xt-9-6-1-asy-2%-IG	Acquired By:	System
Sample Type:	Unknown	Sample Set Name:	0113
Vial:	1	Acq. Method Set:	2%qb
Injection #:	1	Processing Method:	xt 9 6 1 asy
Injection Volume:	10.00 ul	Channel Name:	240.0nm
Run Time:	15.0 Minutes	Proc. Chnl. Descr.:	2998 PDA 240.0 nm (2998
Date Acquired:	1/13/2025 9:56:28 AM CST		
Date Processed:	1/18/2025 11:06:55 PM CST		

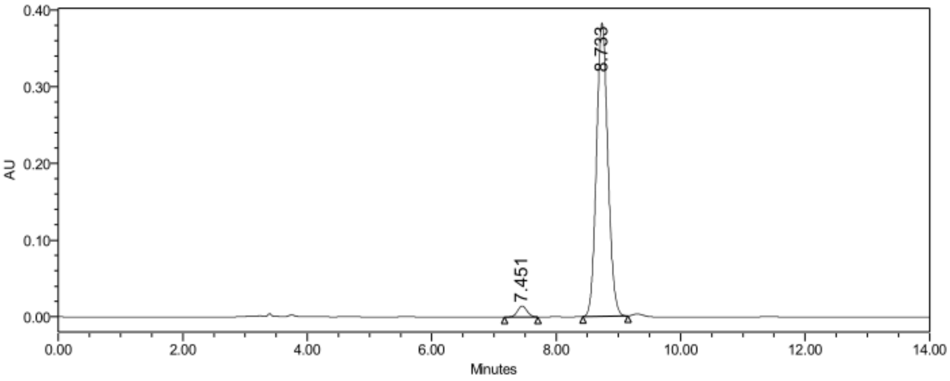

	RT	Area	% Area	Height
1	7.451	154364	3.07	14424
2	8.733	4865927	96.93	382420

Rac-**5g**

SAMPLE INFORMATION

Sample Name:	XT-9-29-2-RAC-2%-IG	Acquired By:	System
Sample Type:	Unknown	Sample Set Name:	
Vial:	28	Acq. Method Set:	2% quanbo
Injection #:	1	Processing Method	XT 9 29 2 RAC
Injection Volume:	30.00 ul	Channel Name:	240.0nm
Run Time:	60.0 Minutes	Proc. Chnl. Descr.:	2998 PDA 240.0 nm (2998
Date Acquired:	2/18/2025 19:10:18 CST		
Date Processed:	2/18/2025 19:24:26 CST		

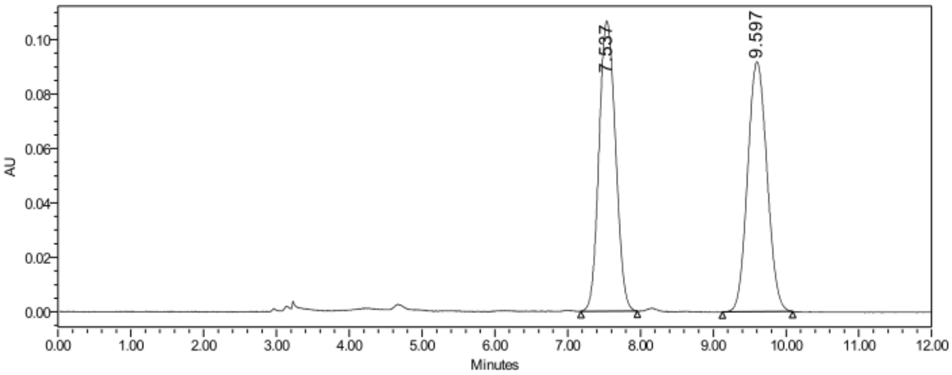

	RT	Area	% Area	Height
1	7.537	1679527	49.84	106737
2	9.597	1690638	50.16	91844

Asy-**5g**

SAMPLE INFORMATION

Sample Name:	XT-9-32-1-asy-2%-IG	Acquired By:	System
Sample Type:	Unknown	Sample Set Name:	
Vial:	26	Acq. Method Set:	2% quanbo
Injection #:	1	Processing Method	XT 9 32 1 ASY
Injection Volume:	30.00 ul	Channel Name:	240.0nm
Run Time:	60.0 Minutes	Proc. Chnl. Descr.:	2998 PDA 240.0 nm (2998
Date Acquired:	2/17/2025 19:36:06 CST		
Date Processed:	2/18/2025 19:23:11 CST		

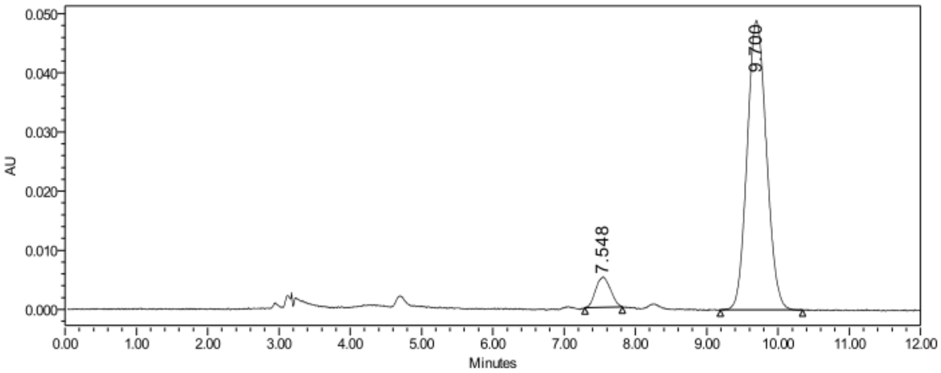

	RT	Area	% Area	Height
1	7.548	73719	7.40	5137
2	9.700	921951	92.60	48963

Rac-**5h**

SAMPLE INFORMATION

Sample Name:	xt-9-14-4-rac-5%-IG	Acquired By:	System
Sample Type:	Unknown	Sample Set Name:	
Vial:	57	Acq. Method Set:	5%qb
Injection #:	1	Processing Method:	xt 9 14 4 rac
Injection Volume:	30.00 ul	Channel Name:	254.0nm
Run Time:	60.0 Minutes	Proc. Chnl. Descr.:	2998 PDA 254.0 nm (2998
Date Acquired:	1/16/2025 10:27:05 PM CST		
Date Processed:	1/16/2025 10:40:23 PM CST		

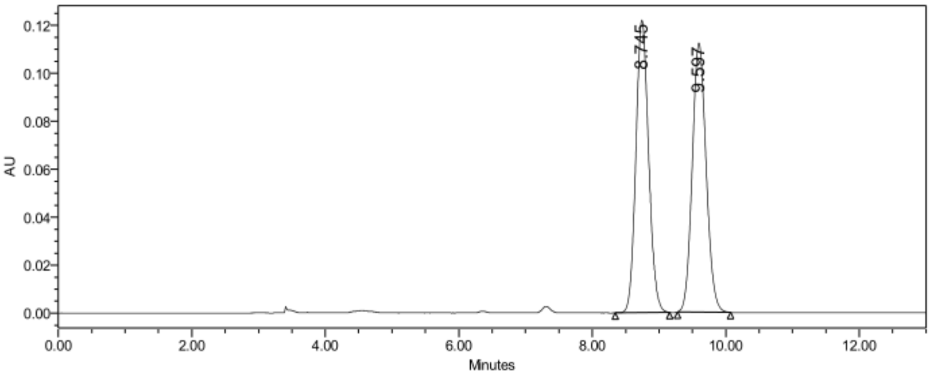

	RT	Area	% Area	Height
1	8.745	1619902	49.87	121949
2	9.597	1628649	50.13	112116

Asy-**5h**

SAMPLE INFORMATION

Sample Name:	xt-9-14-3-asy-5%-IG	Acquired By:	System
Sample Type:	Unknown	Sample Set Name:	
Vial:	44	Acq. Method Set:	5%qb
Injection #:	1	Processing Method:	xt 9 14 3 asy
Injection Volume:	10.00 ul	Channel Name:	254.0nm
Run Time:	60.0 Minutes	Proc. Chnl. Descr.:	2998 PDA 254.0 nm (2998
Date Acquired:	1/16/2025 10:07:09 PM CST		
Date Processed:	1/16/2025 10:41:55 PM CST		

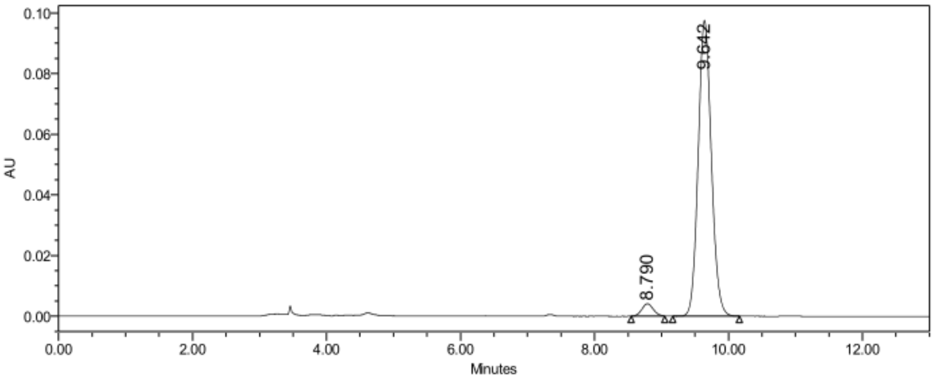

	RT	Area	% Area	Height
1	8.790	48485	3.50	4059
2	9.642	1336989	96.50	97546

Rac-**5i**

SAMPLE INFORMATION

Sample Name:	xt-8-161-6-RAC-2%-IE	Acquired By:	System
Sample Type:	Unknown	Sample Set Name:	
Vial:	107	Acq. Method Set:	2%qb
Injection #:	1	Processing Method:	XT 8 161 6 RAC
Injection Volume:	30.00 ul	Channel Name:	254.0nm
Run Time:	60.0 Minutes	Proc. Chnl. Descr.:	2998 PDA 254.0 nm (2998
Date Acquired:	12/22/2024 7:31:46 PM CST		
Date Processed:	12/22/2024 8:00:44 PM CST		

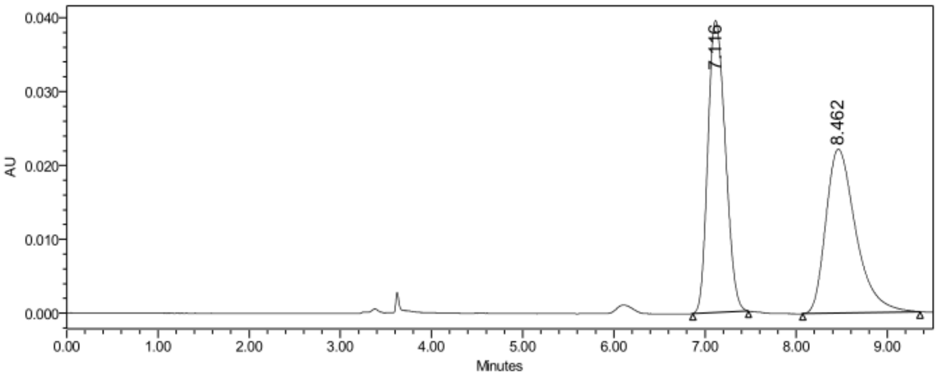

	RT	Area	% Area	Height
1	7.116	525625	50.93	39532
2	8.462	506336	49.07	22203

Asy-**5i**

SAMPLE INFORMATION

Sample Name:	xt-8-161-5-ASY-2%-IE	Acquired By:	System
Sample Type:	Unknown	Sample Set Name:	
Vial:	19	Acq. Method Set:	2%qb
Injection #:	1	Processing Method:	XT 8 161 5 ASY
Injection Volume:	30.00 ul	Channel Name:	254.0nm
Run Time:	60.0 Minutes	Proc. Chnl. Descr.:	2998 PDA 254.0 nm (2998
Date Acquired:	12/22/2024 7:48:45 PM CST		
Date Processed:	12/22/2024 8:02:00 PM CST		

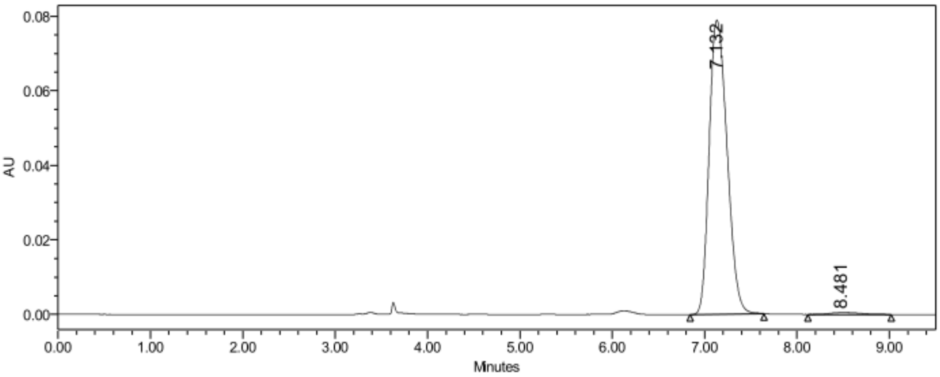

	RT	Area	% Area	Height
1	7.132	1061915	98.91	78916
2	8.481	11742	1.09	553

Rac-**3al**

SAMPLE INFORMATION			
Sample Name:	XT-8-58-1-RAC-2%-IG	Acquired By:	System
Sample Type:	Unknown	Sample Set Name	
Vial:	84	Acq. Method Set:	2% quanbo
Injection #:	1	Processing Method	XT 8 58 1 RAC
Injection Volume:	10.00 ul	Channel Name:	254.0nm
Run Time:	60.0 Minutes	Proc. Chnl. Descr.:	2998 PDA 254.0 nm (2998
Date Acquired:	9/25/2024 20:47:11 CST		
Date Processed:	9/26/2024 17:06:19 CST		

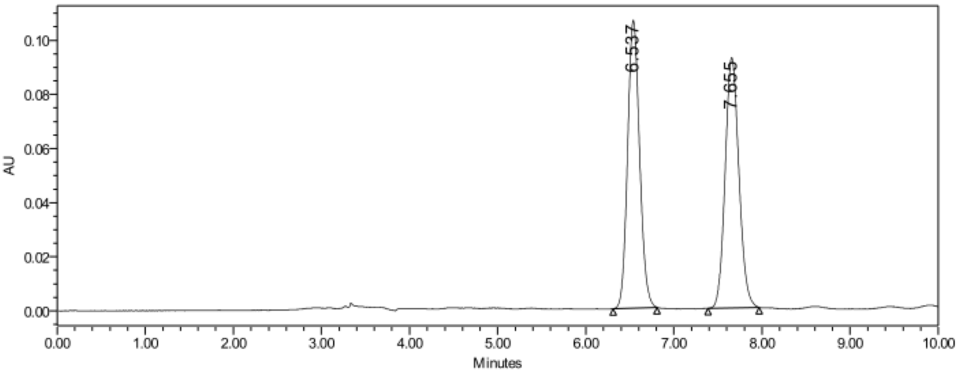

	RT	Area	% Area	Height
1	6.537	1012687	50.08	106372
2	7.655	1009274	49.92	92532

Asy-**3al**

SAMPLE INFORMATION			
Sample Name:	XT-8-157-1-ASY-2%-IG	Acquired By:	System
Sample Type:	Unknown	Sample Set Name	
Vial:	52	Acq. Method Set:	2% quanbo
Injection #:	1	Processing Method	XT 8 157 1 ASY
Injection Volume:	30.00 ul	Channel Name:	254.0nm
Run Time:	60.0 Minutes	Proc. Chnl. Descr.:	2998 PDA 254.0 nm (2998
Date Acquired:	12/11/2024 15:14:50 CST		
Date Processed:	12/11/2024 15:30:10 CST		

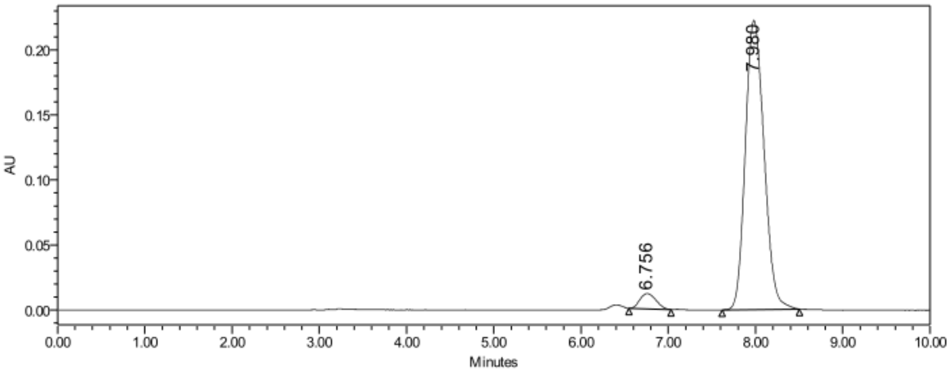

	RT	Area	% Area	Height
1	6.756	151310	4.38	11778
2	7.980	3300782	95.62	222441

Rac-**6**

SAMPLE INFORMATION

Sample Name:	XT-8-137-2-RAC-2%-IG	Acquired By:	System
Sample Type:	Unknown	Sample Set Name:	
Vial:	62	Acq. Method Set:	2% quanbo
Injection #:	1	Processing Method	XT 8 137 2 RAC
Injection Volume:	30.00 ul	Channel Name:	254.0nm
Run Time:	60.0 Minutes	Proc. Chnl. Descr.:	2998 PDA 254.0 nm (2998
Date Acquired:	1/9/2025 15:34:34 CST		
Date Processed:	1/9/2025 15:45:07 CST		

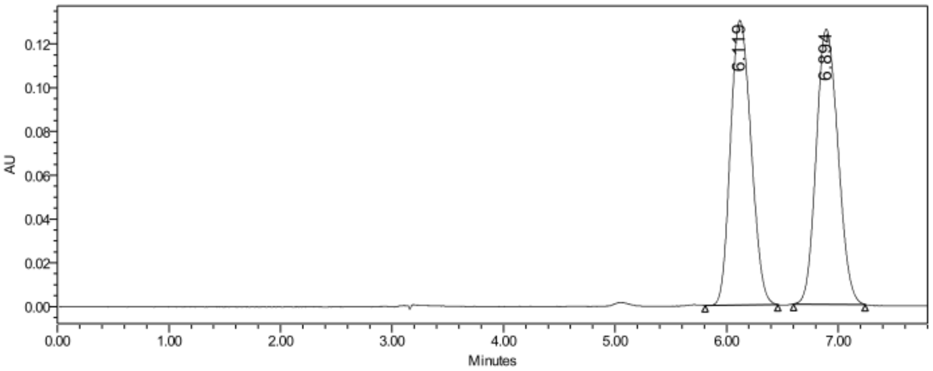

	RT	Area	% Area	Height
1	6.119	1670948	49.54	130046
2	6.894	1701704	50.46	125686

Asy-**6**

SAMPLE INFORMATION

Sample Name:	xt-8-137-1-ASY-2%-IG	Acquired By:	System
Sample Type:	Unknown	Sample Set Name:	1123
Vial:	54	Acq. Method Set:	2%qb
Injection #:	1	Processing Method:	XT 8 137 1 ASY
Injection Volume:	30.00 ul	Channel Name:	254.0nm
Run Time:	11.0 Minutes	Proc. Chnl. Descr.:	2998 PDA 254.0 nm (2998
Date Acquired:	11/23/2024 11:42:41 AM CST		
Date Processed:	11/25/2024 10:28:53 PM CST		

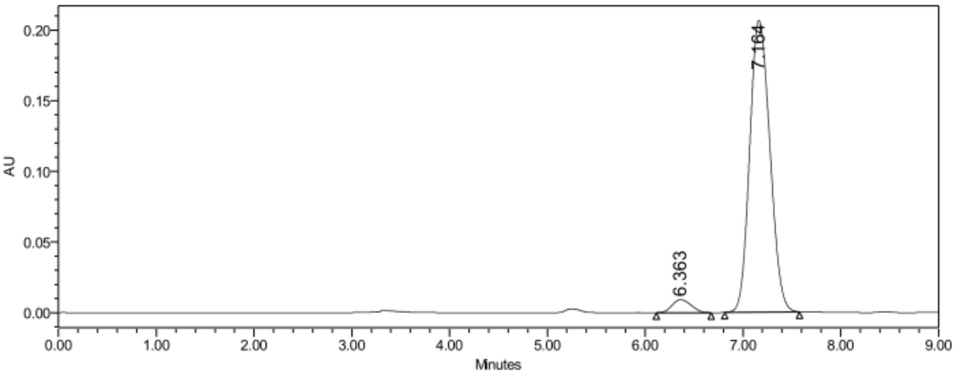

	RT	Area	% Area	Height
1	6.363	124003	4.01	9150
2	7.164	2965934	95.99	206356

Rac-**7**

SAMPLE INFORMATION

Sample Name:	xt-8-140-2-RAC-8%-IG	Acquired By:	System
Sample Type:	Unknown	Sample Set Name:	1125
Vial:	55	Acq. Method Set:	8%qb
Injection #:	1	Processing Method:	XT 8 140 2 RAC
Injection Volume:	10.00 ul	Channel Name:	254.0nm
Run Time:	17.0 Minutes	Proc. Chnl. Descr.:	2998 PDA 254.0 nm (2998
Date Acquired:	11/25/2024 9:20:04 PM CST		
Date Processed:	11/25/2024 10:27:55 PM CST		

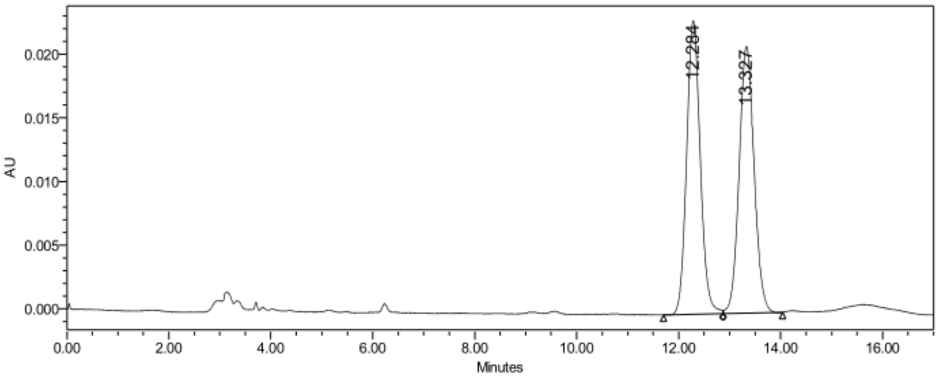

	RT	Area	% Area	Height
1	12.284	428619	49.99	23068
2	13.327	428720	50.01	20972

Asy-**7**

SAMPLE INFORMATION

Sample Name:	xt-8-140-3-ASY-8%-IG	Acquired By:	System
Sample Type:	Unknown	Sample Set Name:	1125
Vial:	93	Acq. Method Set:	8%qb
Injection #:	1	Processing Method:	XT 8 140 3 ASY
Injection Volume:	20.00 ul	Channel Name:	254.0nm
Run Time:	17.0 Minutes	Proc. Chnl. Descr.:	2998 PDA 254.0 nm (2998
Date Acquired:	11/25/2024 9:38:04 PM CST		
Date Processed:	11/25/2024 10:26:47 PM CST		

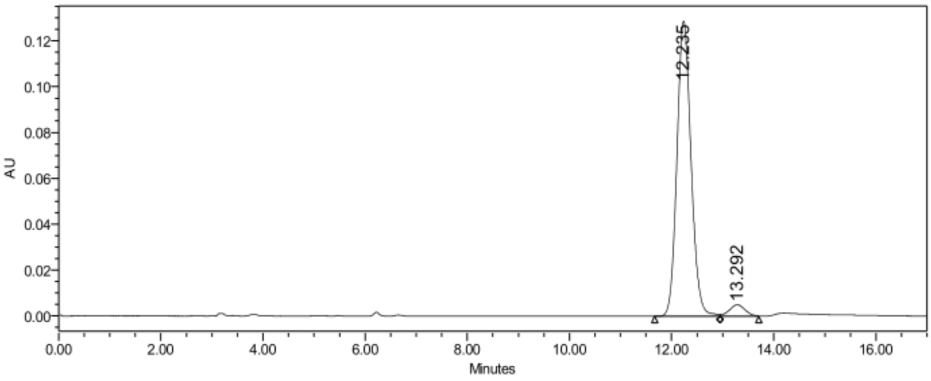

	RT	Area	% Area	Height
1	12.235	2478831	95.98	128892
2	13.292	103706	4.02	4931

Rac-**8**

SAMPLE INFORMATION

Sample Name:	XT-8-136-2-H2-RAC-8%-IG	Acquired By:	System
Sample Type:	Unknown	Sample Set Name	
Vial:	31	Acq. Method Set:	8% quanbo
Injection #:	1	Processing Method	XT 8 136 2 RAC
Injection Volume:	10.00 ul	Channel Name:	254.0nm
Run Time:	60.0 Minutes	Proc. Chnl. Descr.:	2998 PDA 254.0 nm (2998
Date Acquired:	11/28/2024 14:47:51 CST		
Date Processed:	11/28/2024 15:05:21 CST		

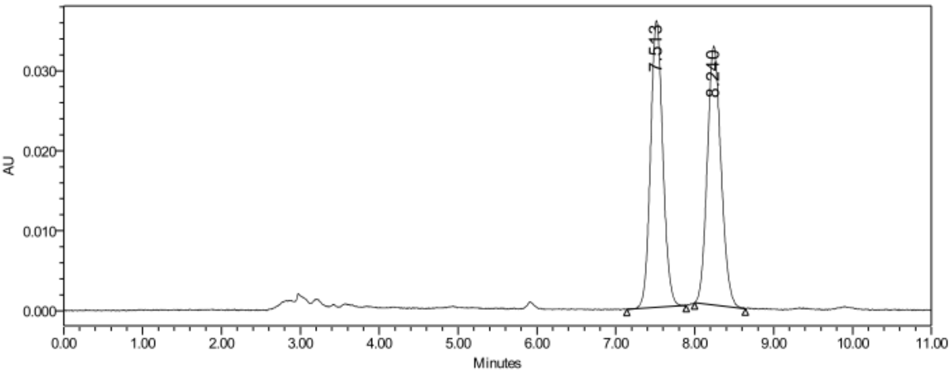

	RT	Area	% Area	Height
1	7.513	387556	49.66	35844
2	8.240	392908	50.34	32386

Asy-**8**

SAMPLE INFORMATION

Sample Name:	XT-8-136-3-H2-asy-8%-IG	Acquired By:	System
Sample Type:	Unknown	Sample Set Name	
Vial:	43	Acq. Method Set:	8% quanbo
Injection #:	1	Processing Method	XT 8 136 3 ASY
Injection Volume:	10.00 ul	Channel Name:	254.0nm
Run Time:	60.0 Minutes	Proc. Chnl. Descr.:	2998 PDA 254.0 nm (2998
Date Acquired:	11/28/2024 10:33:45 CST		
Date Processed:	11/28/2024 15:06:57 CST		

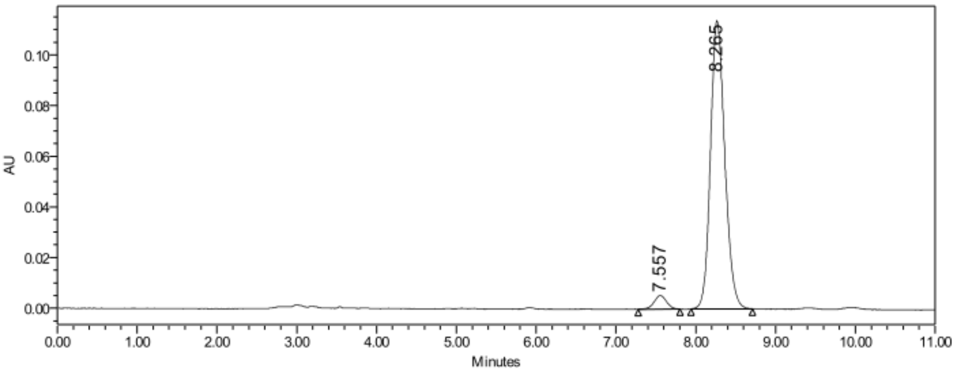

	RT	Area	% Area	Height
1	7.557	58341	3.96	5416
2	8.265	1414093	96.04	113833

Rac-**9**

SAMPLE INFORMATION

Sample Name:	xt-8-167-3-RAC-2%-IG	Acquired By:	System
Sample Type:	Unknown	Sample Set Name:	
Vial:	80	Acq. Method Set:	2%qb
Injection #:	1	Processing Method:	XT 8 167 3 RAC
Injection Volume:	10.00 ul	Channel Name:	254.0nm
Run Time:	60.0 Minutes	Proc. Chnl. Descr.:	2998 PDA 254.0 nm (2998
Date Acquired:	12/25/2024 2:40:46 PM CST		
Date Processed:	12/25/2024 2:54:46 PM CST		

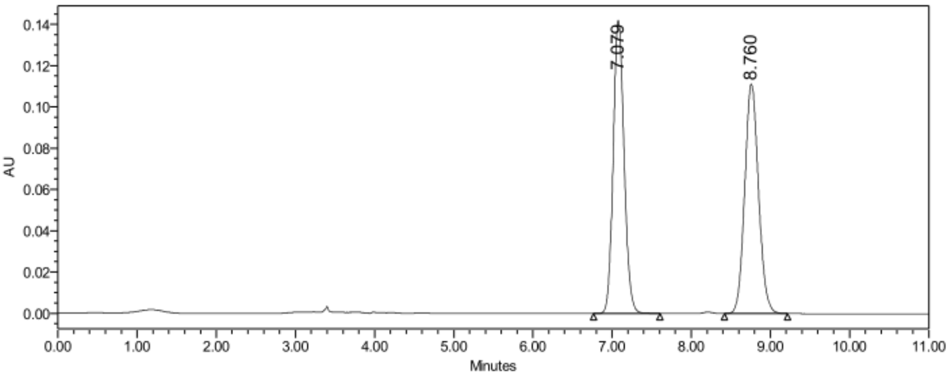

	RT	Area	% Area	Height
1	7.079	1347576	49.91	141993
2	8.760	1352625	50.09	111296

Asy-**9**

SAMPLE INFORMATION

Sample Name:	xt-8-167-4-ASY-2%-IG	Acquired By:	System
Sample Type:	Unknown	Sample Set Name:	1225
Vial:	53	Acq. Method Set:	2%qb
Injection #:	1	Processing Method:	XT 8 167 4 ASY
Injection Volume:	30.00 ul	Channel Name:	254.0nm
Run Time:	12.0 Minutes	Proc. Chnl. Descr.:	2998 PDA 254.0 nm (2998
Date Acquired:	12/25/2024 11:48:00 AM CST		
Date Processed:	12/25/2024 2:44:30 PM CST		

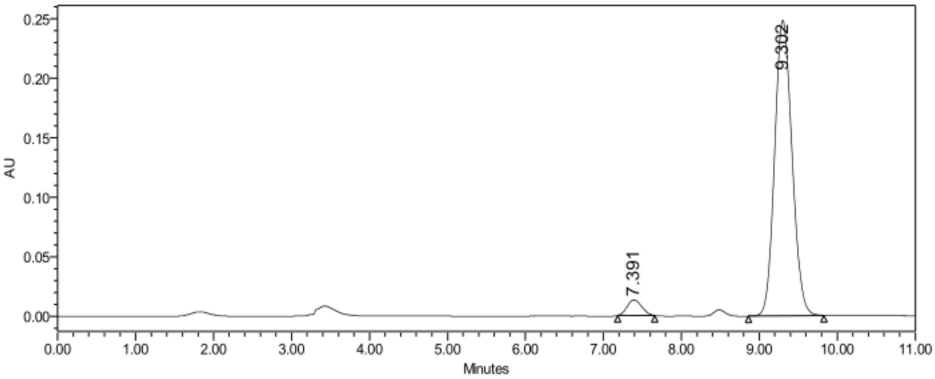

	RT	Area	% Area	Height
1	7.391	170202	4.22	13200
2	9.302	3861798	95.78	248353

Rac-**10**

SAMPLE INFORMATION

Sample Name:	xt-8-167-1-RAC-2%-IG	Acquired By:	System
Sample Type:	Unknown	Sample Set Name:	
Vial:	33	Acq. Method Set:	2%qb
Injection #:	1	Processing Method:	XT 8 167 1 RAC
Injection Volume:	20.00 ul	Channel Name:	254.0nm
Run Time:	60.0 Minutes	Proc. Chnl. Descr.:	2998 PDA 254.0 nm (2998
Date Acquired:	12/25/2024 11:18:47 AM CST		
Date Processed:	12/25/2024 2:41:56 PM CST		

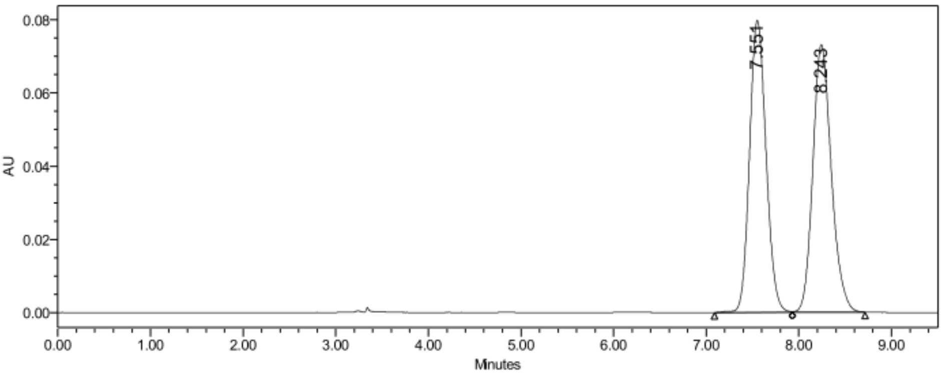

	RT	Area	% Area	Height
1	7.551	986979	49.25	79781
2	8.243	1016899	50.75	73099

Asy-**10**

SAMPLE INFORMATION

Sample Name:	xt-8-167-2-ASY-2%-IG	Acquired By:	System
Sample Type:	Unknown	Sample Set Name:	1225
Vial:	52	Acq. Method Set:	2%qb
Injection #:	1	Processing Method:	XT 8 167 2 ASY
Injection Volume:	30.00 ul	Channel Name:	254.0nm
Run Time:	12.0 Minutes	Proc. Chnl. Descr.:	2998 PDA 254.0 nm (2998
Date Acquired:	12/25/2024 11:35:11 AM CST		
Date Processed:	12/25/2024 2:40:23 PM CST		

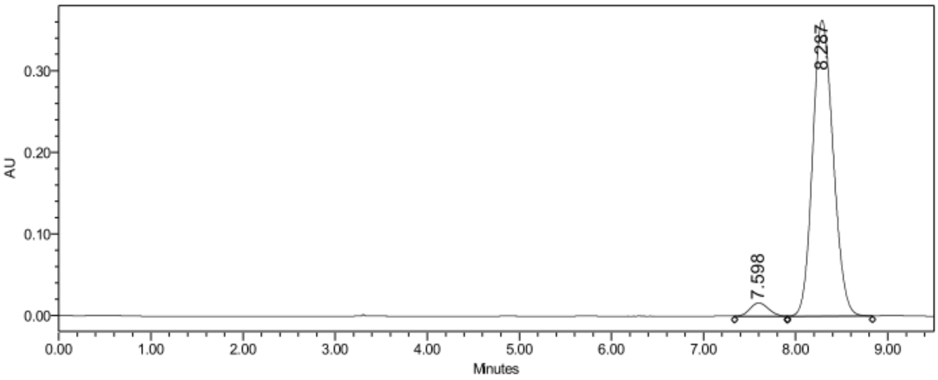

	RT	Area	% Area	Height
1	7.598	229020	3.91	16229
2	8.287	5627001	96.09	362582

Rac-**11**

SAMPLE INFORMATION

Sample Name:	XT-8-196-1-RAC-2%-IC	Acquired By:	System
Sample Type:	Unknown	Sample Set Name:	
Vial:	84	Acq. Method Set:	2% quanbo
Injection #:	1	Processing Method	XT 8 196 1 RAC
Injection Volume:	10.00 ul	Channel Name:	254.0nm
Run Time:	60.0 Minutes	Proc. Chnl. Descr.:	2998 PDA 254.0 nm (2998
Date Acquired:	1/11/2025 20:34:25 CST		
Date Processed:	2/22/2025 16:28:08 CST		

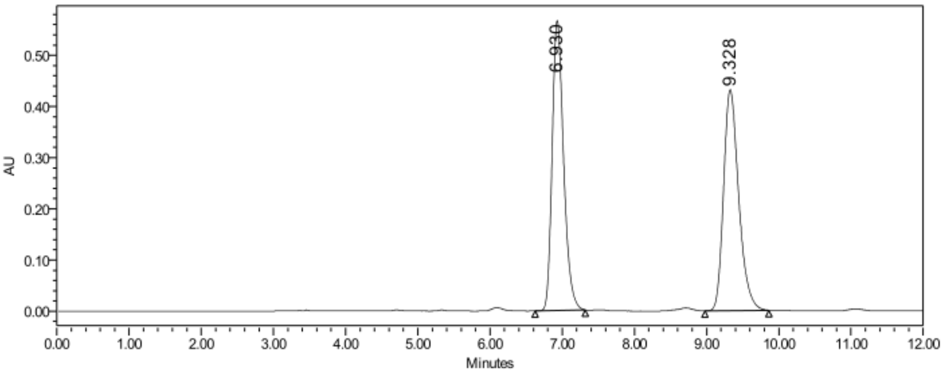

	RT	Area	% Area	Height
1	6.930	6270886	49.81	566916
2	9.328	6317489	50.19	431855

Asy-**11**

SAMPLE INFORMATION

Sample Name:	XT-8-196-2-ASY-2%-IC	Acquired By:	System
Sample Type:	Unknown	Sample Set Name:	
Vial:	113	Acq. Method Set:	2% quanbo
Injection #:	1	Processing Method	XT 8 196 2 ASY
Injection Volume:	10.00 ul	Channel Name:	254.0nm
Run Time:	60.0 Minutes	Proc. Chnl. Descr.:	2998 PDA 254.0 nm (2998
Date Acquired:	1/11/2025 20:49:07 CST		
Date Processed:	2/22/2025 16:26:26 CST		

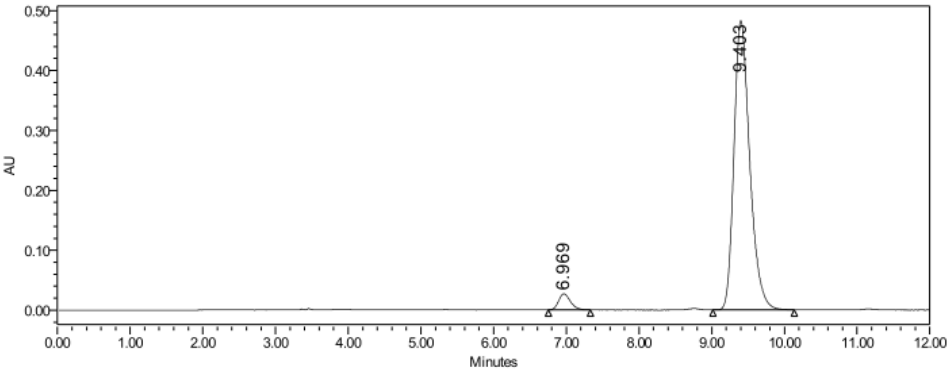

	RT	Area	% Area	Height
1	6.969	304967	4.02	27020
2	9.403	7283183	95.98	483025

Rac-**13**

SAMPLE INFORMATION			
Sample Name:	XT-9-69-3-rac-2%-IG	Acquired By:	System
Sample Type:	Unknown	Sample Set Name:	
Vial:	42	Acq. Method Set:	2% quanbo
Injection #:	1	Processing Method	XT 9 69 3 RAC
Injection Volume:	30.00 ul	Channel Name:	254.0nm
Run Time:	60.0 Minutes	Proc. Chnl. Descr.:	2998 PDA 254.0 nm (2998
Date Acquired:	4/27/2025 20:50:42 CST		
Date Processed:	4/27/2025 21:15:51 CST		

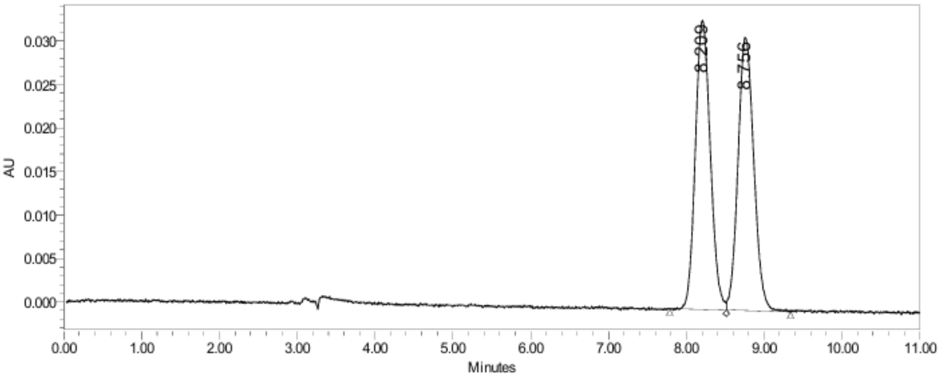

	RT	Area	% Area	Height
1	8.209	444020	50.04	33234
2	8.756	443375	49.96	31386

Asy-**13**

SAMPLE INFORMATION			
Sample Name:	XT-9-69-3-ASY-2%-IG	Acquired By:	System
Sample Type:	Unknown	Sample Set Name:	
Vial:	49	Acq. Method Set:	2% quanbo
Injection #:	1	Processing Method	XT 9 69 3 ASY
Injection Volume:	30.00 ul	Channel Name:	254.0nm
Run Time:	60.0 Minutes	Proc. Chnl. Descr.:	2998 PDA 254.0 nm (2998
Date Acquired:	4/27/2025 21:05:31 CST		
Date Processed:	4/27/2025 21:17:16 CST		

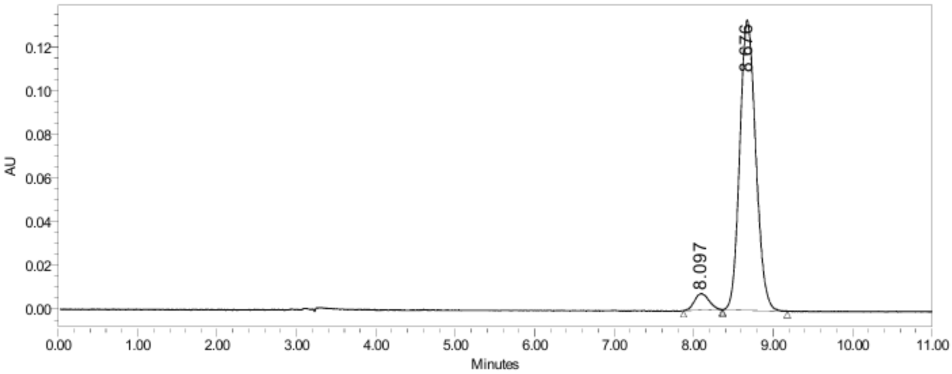

	RT	Area	% Area	Height
1	8.097	96904	4.98	7627
2	8.676	1850905	95.02	132765

Rac-**14**

SAMPLE INFORMATION

Sample Name:	XT-9-69-4-rac-5%-IG	Acquired By:	System
Sample Type:	Unknown	Sample Set Name	
Vial:	111	Acq. Method Set:	5% quanbo
Injection #:	1	Processing Method	xt 9 69 4 rac
Injection Volume:	30.00 ul	Channel Name:	245.0nm
Run Time:	60.0 Minutes	Proc. Chnl. Descr.:	2998 PDA 245.0 nm (2998
Date Acquired:	4/28/2025 20:17:18 CST		
Date Processed:	4/28/2025 21:38:18 CST		

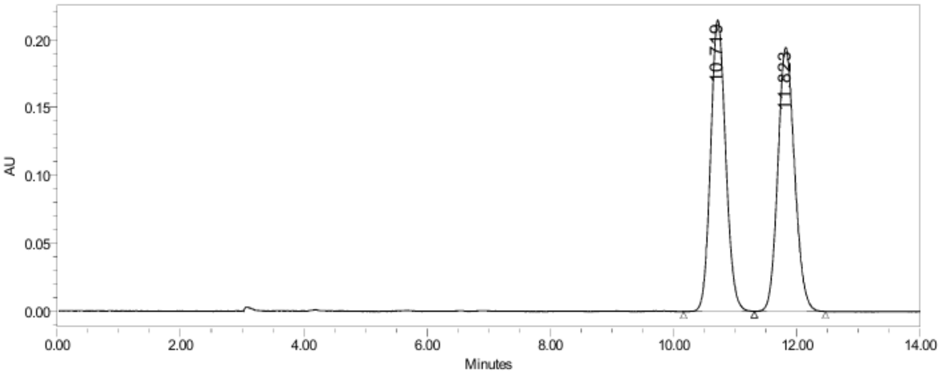

	RT	Area	% Area	Height
1	10.719	3678421	50.04	214880
2	11.823	3672807	49.96	194589

Asy-**14**

SAMPLE INFORMATION

Sample Name:	XT-9-69-4-asy-5%-IG	Acquired By:	System
Sample Type:	Unknown	Sample Set Name	
Vial:	112	Acq. Method Set:	5% quanbo
Injection #:	1	Processing Method	xt 9 69 4 asy
Injection Volume:	30.00 ul	Channel Name:	245.0nm
Run Time:	60.0 Minutes	Proc. Chnl. Descr.:	2998 PDA 245.0 nm (2998
Date Acquired:	4/28/2025 21:17:04 CST		
Date Processed:	4/28/2025 21:35:50 CST		

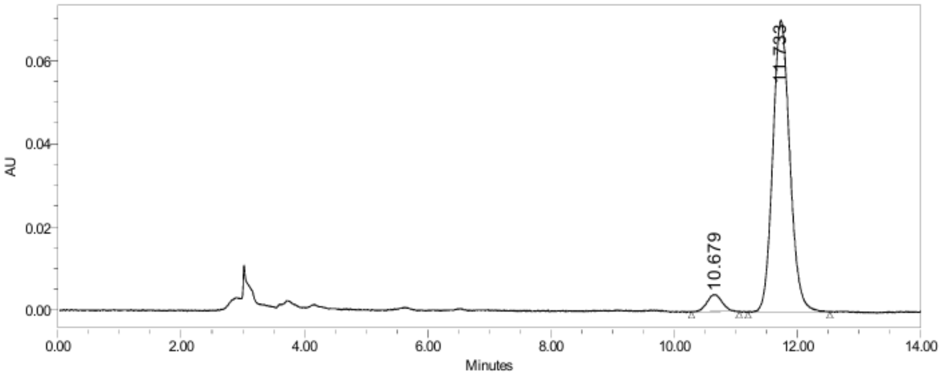

	RT	Area	% Area	Height
1	10.679	70390	4.95	4104
2	11.733	1350711	95.05	70178

Rac-**15'**

SAMPLE INFORMATION

Sample Name:	XT-9-69-6-RAC-10%-IG	Acquired By:	System
Sample Type:	Unknown	Sample Set Name	0506
Vial:	53	Acq. Method Set:	10% quanbo
Injection #:	1	Processing Method	XT 9 69 6 RAC
Injection Volume:	10.00 ul	Channel Name:	254.0nm
Run Time:	25.0 Minutes	Proc. Chnl. Descr.:	2998 PDA 254.0 nm (2998
Date Acquired:	5/6/2025 11:41:40 CST		
Date Processed:	5/6/2025 12:15:39 CST		

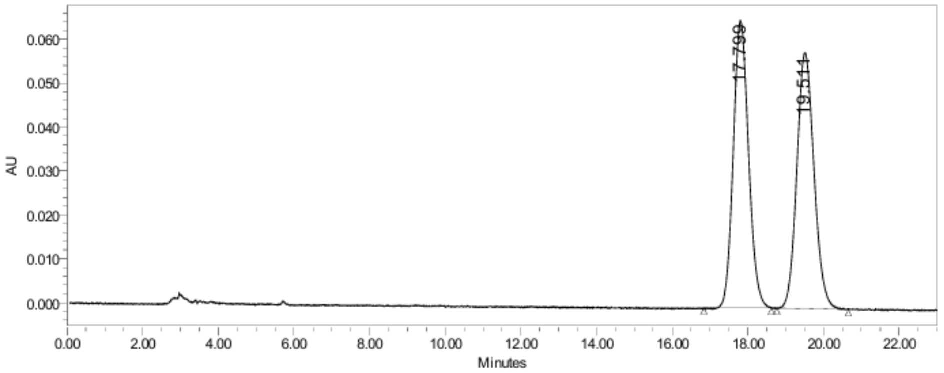

	RT	Area	% Area	Height
1	17.799	1832899	49.95	65503
2	19.511	1836496	50.05	58220

Asy-**15'**

SAMPLE INFORMATION

Sample Name:	XT-9-69-6-ASY-10%-IG	Acquired By:	System
Sample Type:	Unknown	Sample Set Name	0506
Vial:	52	Acq. Method Set:	10% quanbo
Injection #:	1	Processing Method	XT 9 69 6 ASY
Injection Volume:	10.00 ul	Channel Name:	254.0nm
Run Time:	25.0 Minutes	Proc. Chnl. Descr.:	2998 PDA 254.0 nm (2998
Date Acquired:	5/6/2025 11:16:00 CST		
Date Processed:	5/6/2025 12:13:05 CST		

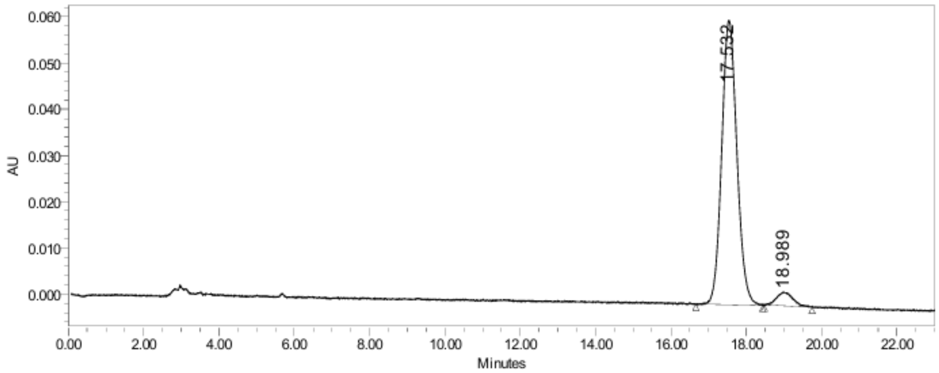

	RT	Area	% Area	Height
1	17.532	1717913	95.06	61502
2	18.989	89251	4.94	3053

Rac-**16**

SAMPLE INFORMATION

Sample Name:	XT-9-2-2-RAC-2%-IG	Acquired By:	System
Sample Type:	Unknown	Sample Set Name:	0111
Vial:	23	Acq. Method Set:	2% quanbo
Injection #:	1	Processing Method	XT 9 2 2 RAC
Injection Volume:	30.00 ul	Channel Name:	271.0nm
Run Time:	15.0 Minutes	Proc. Chnl. Descr.:	2998 PDA 271.0 nm (2998
Date Acquired:	1/11/2025 21:40:48 CST		
Date Processed:	1/18/2025 23:01:19 CST		

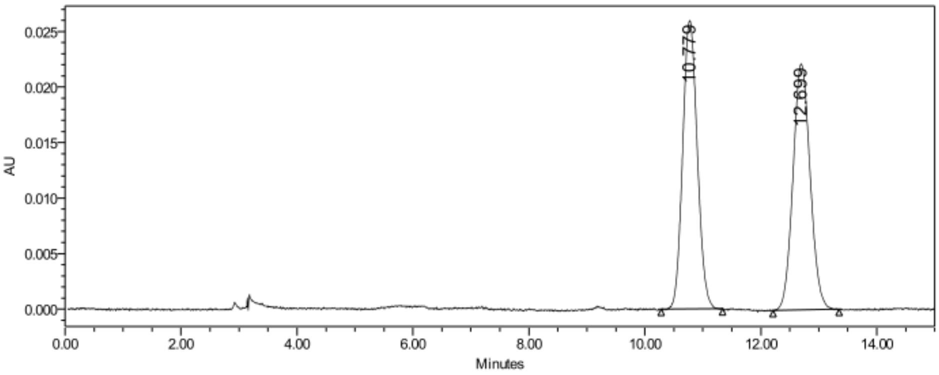

	RT	Area	% Area	Height
1	10.779	454218	49.92	25981
2	12.699	455588	50.08	22172

Asy-**16**

SAMPLE INFORMATION

Sample Name:	xt-9-4-2-D-2%-IG	Acquired By:	System
Sample Type:	Unknown	Sample Set Name:	
Vial:	33	Acq. Method Set:	2%qb
Injection #:	1	Processing Method:	xt 9 4 2 D asy
Injection Volume:	10.00 ul	Channel Name:	254.0nm
Run Time:	60.0 Minutes	Proc. Chnl. Descr.:	2998 PDA 254.0 nm (2998
Date Acquired:	2/12/2025 5:00:57 PM CST		
Date Processed:	2/16/2025 3:07:34 PM CST		

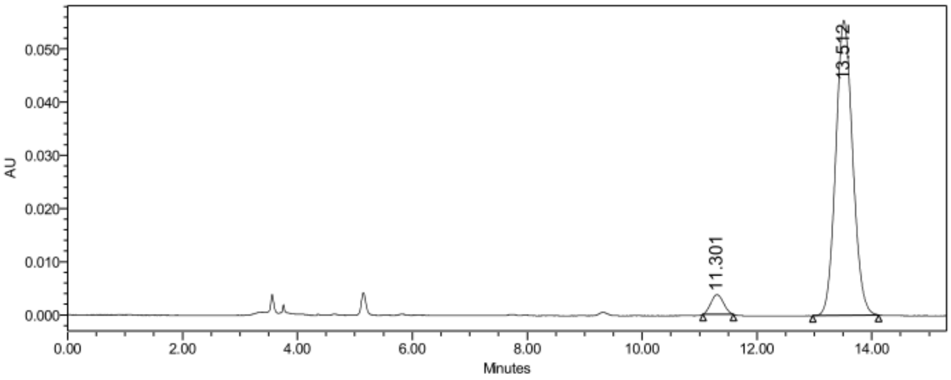

	RT	Area	% Area	Height
1	11.301	56420	4.74	3686
2	13.512	1134822	95.26	55432

Rac-**18**

SAMPLE INFORMATION

Sample Name:	XT-8-118-1-rac-2%-IG	Acquired By:	System
Sample Type:	Unknown	Sample Set Name:	
Vial:	63	Acq. Method Set:	2% quanbo
Injection #:	1	Processing Method	XT 8 118 1 RAC
Injection Volume:	10.00 ul	Channel Name:	254.0nm
Run Time:	60.0 Minutes	Proc. Chnl. Descr.:	2998 PDA 254.0 nm (2998
Date Acquired:	12/3/2024 14:54:59 CST		
Date Processed:	12/14/2024 20:54:26 CST		

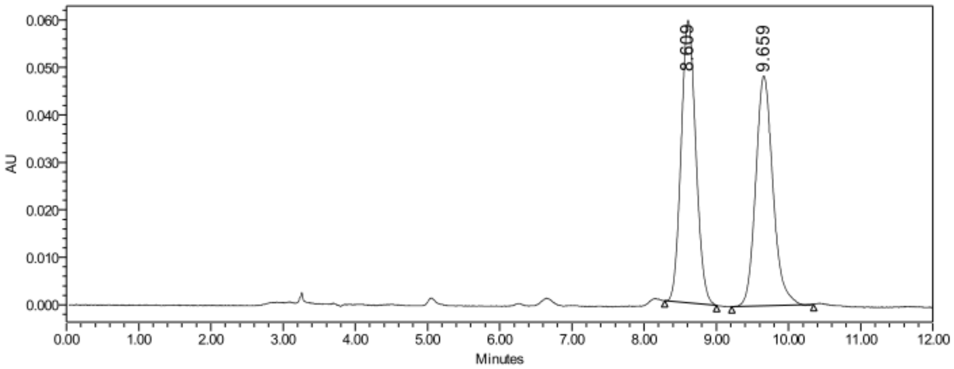

	RT	Area	% Area	Height
1	8.609	841635	51.16	59496
2	9.659	803422	48.84	48406

Asy-**18**

SAMPLE INFORMATION

Sample Name:	XT-8-118-1-ASY-2%-IG	Acquired By:	System
Sample Type:	Unknown	Sample Set Name:	
Vial:	64	Acq. Method Set:	2% quanbo
Injection #:	1	Processing Method	XT 8 118 1 ASY
Injection Volume:	10.00 ul	Channel Name:	254.0nm
Run Time:	60.0 Minutes	Proc. Chnl. Descr.:	2998 PDA 254.0 nm (2998
Date Acquired:	12/3/2024 15:56:11 CST		
Date Processed:	12/14/2024 20:51:58 CST		

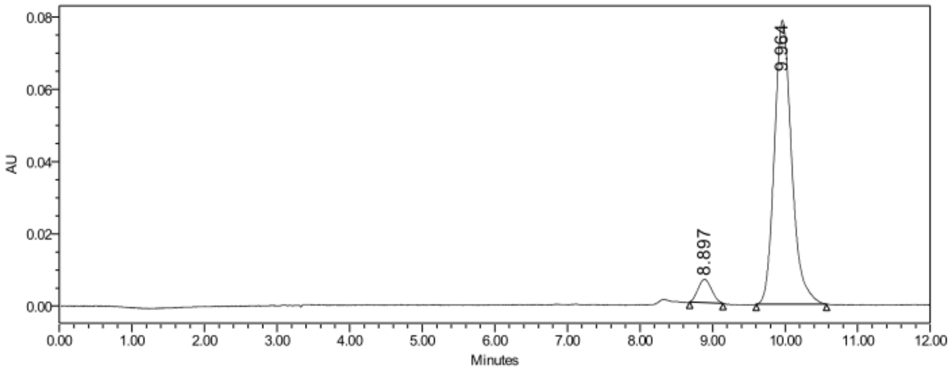

	RT	Area	% Area	Height
1	8.897	83128	5.98	6463
2	9.964	1307124	94.02	78643

Rac-**20**

SAMPLE INFORMATION

Sample Name:	xt-8-153-1-1-rac-2%-IC	Acquired By:	System
Sample Type:	Unknown	Sample Set Name:	
Vial:	83	Acq. Method Set:	2%qb
Injection #:	1	Processing Method:	XT 8 153 1 1 RAC
Injection Volume:	10.00 ul	Channel Name:	254.0nm
Run Time:	60.0 Minutes	Proc. Chnl. Descr.:	2998 PDA 254.0 nm (2998
Date Acquired:	12/9/2024 4:41:45 PM CST		
Date Processed:	12/14/2024 9:02:01 PM CST		

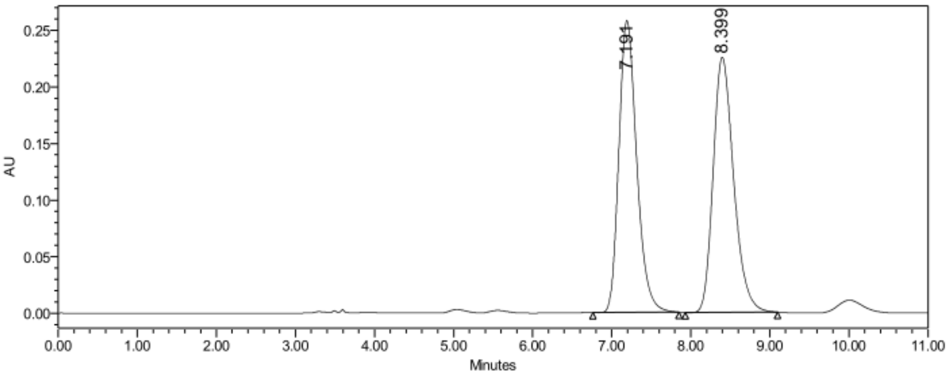

	RT	Area	% Area	Height
1	7.191	4038759	49.72	257827
2	8.399	4083632	50.28	225432

Asy-**20**

SAMPLE INFORMATION

Sample Name:	xt-8-118-3-ASY-2%-IC	Acquired By:	System
Sample Type:	Unknown	Sample Set Name:	1209
Vial:	111	Acq. Method Set:	2%qb
Injection #:	1	Processing Method:	XT 8 118 3 ASY
Injection Volume:	10.00 ul	Channel Name:	254.0nm
Run Time:	18.0 Minutes	Proc. Chnl. Descr.:	2998 PDA 254.0 nm (2998
Date Acquired:	12/9/2024 5:01:42 PM CST		
Date Processed:	12/14/2024 8:59:57 PM CST		

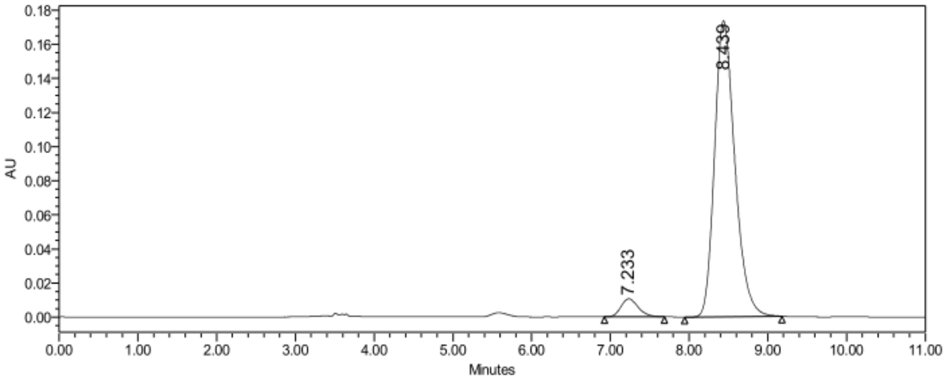

	RT	Area	% Area	Height
1	7.233	163825	5.00	10661
2	8.439	3115732	95.00	173654

13. References

1 Zhang, J., Zhu, W., Chen, Z., Zhang, Q. & Guo, C. Dual-Catalyzed Stereodivergent Electrooxidative Homocoupling of Benzoxazolyl Acetate. *J. Am. Chem. Soc.* **146**, 1522-1531 (2024).

2 Zhang, Q., Chang, X., Peng, L. & Guo, C. Asymmetric Lewis Acid Catalyzed Electrochemical Alkylation. *Angew. Chem. Int. Ed. Engl.* **58**, 6999-7003 (2019).

3 Qiao, J., Ci, R.-N., Gan, Q.-C., Huang, C., Liu, Z., Hu, H.-L., Ye, C., Chen, B., Tung, C.-H. & Wu, L.-Z. Amine-Free, Directing-Group-Free and Redox-Neutral α-Alkylation of Saturated Cyclic Ketones. *Angew. Chem. Int. Ed.* **62**, e202305679 (2023).

4 Yang, N., Mao, C., Zhang, H., Wang, P., Li, S., Xie, L. & Liao, S. FSO2 Radical-Initiated Tandem Addition Reaction of Two Different Olefins: A Facile Access to Multifunctional Aliphatic Sulfonyl Fluorides. *Org. Lett.* **25**, 4478-4482 (2023).

5 Li, X., He, S. & Song, Q. Diethylzinc-Mediated Radical 1,2-Addition of Alkenes and Alkynes. *Org. Lett.* **23**, 2994-2999 (2021).

6 Berg, N., Bergwinkl, S., Nuernberger, P., Horinek, D. & Gschwind, R. M. Extended Hydrogen Bond Networks for Effective Proton-Coupled Electron Transfer (PCET) Reactions: The Unexpected Role of Thiophenol and Its Acidic Channel in Photocatalytic Hydroamidations. *J. Am. Chem. Soc.* **143**, 724-735 (2021).

7 Curtis, N. R., Davies, S. H., Gray, M., Leach, S. G., Mckie, R. A., Vernon, L. E. & Walkington, A. J. Asymmetric Fluorination Approach to the Scalable Synthesis of a SYK Inhibitor. *Org. Process Res. Dev.* **19**, 865-871 (2015).

8 Hao, Y., Li, J., Cao, X., Meng, L., Wu, J., Yang, X., Li, Y., Liu, Z. & Gong, M. Origin of the Universal Potential-Dependent Organic Oxidation on Nickel Oxyhydroxide. *ACS Catal.* **13**, 2916-2927 (2023)

9 Frisch, M. J., Trucks, G. W., Schlegel, H. B., Scuseria, G. E., Robb, M. A., Cheeseman, J. R., Scalmani, G., Barone, V., Mennucci, B., Petersson, G. A., Nakatsuji, H., Caricato, M., Li, X., Hratchian, H. P., Izmaylov, A. F., Bloino, J., Zheng, G., Sonnenberg, J. L., Hada, M., Ehara, M., Toyota, K., Fukuda, R., Hasegawa, J., Ishida, M., Nakajima, T., Honda, Y., Kitao, O., Nakai, H., Vreven, T., Montgomery, J. A., Jr., Peralta, J. E., Ogliaro, F., Bearpark, M., Heyd, J. J., Brothers, E., Kudin, K. N., Staroverov, V. N., Kobayashi, R., Normand, J., Raghavachari, K., Rendell, A., Burant, J. C., Iyengar, S. S., Tomasi, J., Cossi, M., Rega, N., Millam, M. J., Klene, M., Knox, J. E., Cross, J. B., Bakken, V., Adamo, C., Jaramillo, J., Gomperts, R., Stratmann, R. E., Yazyev, O., Austin, A. J., Cammi, R., Pomelli, C., Ochterski, J. W., Martin, R. L., Morokuma, K., Zakrzewski, V. G., Voth, G. A., Salvador, P., Dannenberg, J. J., Dapprich, S., Daniels, A. D., Farkas, Ö ., Foresman, J. B., Ortiz, J. V., Cioslowski, J., Fox, D. J. Gaussian 16, Revision C.01, Gaussian, Inc., Wallingford CT. (2016).

10 Stephens, P. J., Devlin, F. J., Chabalowski, C. F. & Frisch, M. J. Ab Initio Calculation of Vibrational Absorption and Circular Dichroism Spectra Using Density Functional Force Fields. *J. Phys. Chem.* **98**, 11623-11627 (1994).

11 Grimme, S., Ehrlich, S. & Goerigk, L. Effect of the damping function in dispersion corrected density functional theory. *J. Comput. Chem.* **32**, 1456-1465 (2011).

12 Dolg, M., Wedig, U., Stoll, H. & Preuss, H. Energy-adjusted ab initio pseudopotentials for the first row transition elements. *J. Chem. Phys.* **86**, 866-872 (1987).

13 Hariharan, P. C. & Pople, J. A. The influence of polarization functions on molecular orbital hydrogenation energies. *Theor. Chim. Acta* **28**, 213-222 (1973).
14 Grimme, S. Supramolecular Binding Thermodynamics by Dispersion-Corrected Density Functional Theory. *Chem. Eur. J.* **18**, 9955-9964 (2012).
15 Lu, T. & Chen, Q. Shermo: A general code for calculating molecular thermochemistry properties. *Comput. Theor. Chem.* **1200**, 113249 (2021).
16 Chai, J.-D. & Head-Gordon, M. Long-range corrected hybrid density functionals with damped atom–atom dispersion corrections. *Phys. Chem. Chem. Phys.* **10**, 6615-6620 (2008).
17 Weigend, F. & Ahlrichs, R. Balanced basis sets of split valence, triple zeta valence and quadruple zeta valence quality for H to Rn: Design and assessment of accuracy. *Phys. Chem. Chem. Phys.* **7**, 3297-3305 (2005).
18 Marenich, A. V., Cramer, C. J. & Truhlar, D. G. Universal Solvation Model Based on Solute Electron Density and on a Continuum Model of the Solvent Defined by the Bulk Dielectric Constant and Atomic Surface Tensions. *J. Phys. Chem. B* **113**, 6378-6396 (2009).
19 Legault, C. Y. CYLview20, Université de Sherbrooke, http://www.cylview.org. (2020).
20 Li, J., Liu, M., Wei, B., Peng, L., Song, J. & Guo, C. Enantioselective Nickel-Electrocatalyzed Cross-Dehydrogenative α- and γ-Nitroalkylation. *J. Am. Chem. Soc.* **146**, 34043-34052 (2024).
21 Sohtome, Y., Nakamura, G., Muranaka, A., Hashizume, D., Lectard, S., Tsuchimoto, T., Uchiyama, M. & Sodeoka, M. Naked d-orbital in a centrochiral Ni(II) complex as a catalyst for asymmetric [3+2] cycloaddition. *Nat. Commun.* **8**, 14875 (2017).
22 Kochem, A., Kanso, H., Baptiste, B., Arora, H., Philouze, C., Jarjayes, O., Vezin, H., Luneau, D., Orio, M. & Thomas, F. Ligand Contributions to the Electronic Structures of the Oxidized Cobalt(II) salen Complexes. *Inorg. Chem.* **51**, 10557-10571 (2012).
23 Liu, Z., Zhang, L., Luo, S. & Qi, X. Dynamic Hydrogen Bonding Tuned Enantioselectivity Control in Cobaloxime-Chiral Amine Cooperative Catalysis. *Angew. Chem. Int. Ed.* **64**, e202506268 (2025).
24 Staroverov, V. N., Scuseria, G. E., Tao, J. & Perdew, J. P. Comparative assessment of a new nonempirical density functional: Molecules and hydrogen-bonded complexes. *J. Chem. Phys.* **119**, 12129-12137 (2003).
25 Yu, H. S., He, X. & Truhlar, D. G. MN15-L: A New Local Exchange-Correlation Functional for Kohn–Sham Density Functional Theory with Broad Accuracy for Atoms, Molecules, and Solids. *J. Chem. Theory Comput.* **12**, 1280-1293 (2016).
26 Zhang, D. & Truhlar, D. G. Spin Splitting Energy of Transition Metals: A New, More Affordable Wave Function Benchmark Method and Its Use to Test Density Functional Theory. *J. Chem. Theory Comput.* **16**, 4416-4428 (2020).
27 Cirera, J., Via-Nadal, M. & Ruiz, E. Benchmarking Density Functional Methods for Calculation of State Energies of First Row Spin-Crossover Molecules. *Inorg. Chem.* **57**, 14097-14105 (2018).
28 Soda, T., Kitagawa, Y., Onishi, T., Takano, Y., Shigeta, Y., Nagao, H., Yoshioka, Y. & Yamaguchi, K. Ab initio computations of effective exchange integrals for H–H, H–He–H and Mn2O2 complex: comparison of broken-symmetry approaches. *Chem. Phys. Lett.* **319**, 223-230 (2000).
29 Konezny, S. J., Doherty, M. D., Luca, O. R., Crabtree, R. H., Soloveichik, G. L. & Batista, V. S. Reduction of Systematic Uncertainty in DFT Redox Potentials of Transition-Metal Complexes. *J. Phys. Chem. C* **116**, 6349-6356 (2012).

30 Lu, T. & Chen, Q. Simple, Efficient, and Universal Energy Decomposition Analysis Method Based on Dispersion-Corrected Density Functional Theory. *J. Phys. Chem. A* **127**, 7023-7035 (2023).

31 Guo, Q., Mao, Y., Liu, J., Zhu, L., Hong, X. & Lu, Z. Asymmetric Hofmann–Löffler–Freytag-type reaction via a transient carbenium ion complex merging organocatalysis and photocatalysis. *Nat. Catal.* **8**, 448-456 (2025).
